# Assessment of Xenoestrogens in Jordanian Water System: Activity and Identification

**DOI:** 10.3390/toxics11010063

**Published:** 2023-01-09

**Authors:** Yazan Akkam, Derar Omari, Hassan Alhmoud, Mohammad Alajmi, Nosaibah Akkam, Islam Aljarrah

**Affiliations:** 1 Department of Medicinal Chemistry and Pharmacognosy, Faculty of Pharmacy, Yarmouk University, Irbid 21163, Jordan; 2 Department of Pharmaceutical Technology and Pharmaceutics, Faculty of Pharmacy, Yarmouk University, Irbid 21163, Jordan; 3 Faculty of Pharmacy, Jerash University, Irbid 26110, Jordan; 4 Department of Law and Science Department, Kuwait International Law School, Doha 93151, Kuwait; 5 Department of Anatomy and Cell Biology, Faculty of Medicine, Universität des Saarlandes, 66424 Hamburg, Germany

**Keywords:** xenoestrogens, water pollution, UPLC-MS, estrogen receptors, surface water, drinking water

## Abstract

Sex hormone disruptors (xenoestrogens) are a global concern due to their potential toxicity. However, to date, there has been no study to investigate the presence of xenoestrogen pollutants in the Jordanian water system. Samples in triplicates were collected from six locations in Jordan, including dams, surface water, tap or faucet water, and filtered water (drinking water—local company). Xenoestrogens were then extracted and evaluated with a yeast estrogen screen utilizing *Saccharomyces cerevisiae.* Later, possible pollutants were mined using ultrahigh-performance liquid chromatography (UPLC) coupled with a Bruker impact II Q-TOF-MS. Possible hits were identified using MetaboScape software (4000 compounds), which includes pesticide, pharmaceutical pollutant, veterinary drug, and toxic compound databases and a special library of 75 possible xenoestrogens. The presence of xenoestrogens in vegetable samples collected from two different locations was also investigated. The total estrogen equivalents according to the YES system were 2.9 ± 1.2, 9.5 ± 5, 2.5 ± 1.5, 1.4 ± 0.9 ng/L for King Talal Dam, As-Samra Wastewater Treatment Plant, King Abdullah Canal, and tap water, respectively. In Almujeb Dam and drinking water, the estrogenic activity was below the detection limit. Numbers of identified xenoestrogens were: As-Samra Wastewater Treatment Plant 27 pollutants, King Talal Dam 20 pollutants, Almujeb Dam 10 pollutants, King Abdullah Canal 16 pollutants, Irbid tap water 32 pollutants, Amman tap water 30 pollutants, drinking water 3 pollutants, and vegetables 7 pollutants. However, a large number of compounds remained unknown. Xenoestrogen pollutants were detected in all tested samples, but the total estrogenic capacities were within the acceptable range. The major source of xenoestrogen pollutants was agricultural resources. Risk evaluations for low xenoestrogen activity should be taken into account, and thorough pesticide monitoring systems and regular inspections should also be established.

## 1. Introduction

Water treatment and pollution are major concerns worldwide, i.e., heavy metals and (recently appearing) estrogen and xenoestrogen pollutants. Xenoestrogens are estrogen mimics [1]. Many xenoestrogens found in the waterways, both natural and synthetic, can mimic or disrupt the natural estrogens found in humans and animals [2,3,4]. Estrogenic chemicals of varying potency and persistence originate from agriculture, industry, humans, household products, and pharmaceuticals [5].

Xenoestrogens as well as estrogen pollutants are not completely removed during the process of sewage treatment and are carried over into the general aquatic environment. After ground passage, they can eventually be found in drinking water [6,7,8,9]. Although the concentration of these compounds is very low in the water, it has been identified as the main cause of hormonal disruption in wildlife [7,10,11].

Xenoestrogens are widely diffused in the environment, water, and in food, and thus a large portion of the human population is exposed to them worldwide [12]. Xenoestrogens have been linked to several human diseases, such as testicular dysgenesis syndrome [13], hypospadias [14], testicular cancer [15,16], breast cancer [17], endometriosis [18], birth defects [19], decreased sperm counts [20], and others [21,22,23,24].

The scarcity of water is the greatest challenge that Jordan faces. On a per capita basis, Jordan has one of the lowest levels of water resources in the world. In addition, the situation has been exacerbated by periodic massive influxes of refugees, worsening the imbalance between population and water. Despite the presence of some surface water supplies, collectible rain is the main source of water in Jordan via dams, rivers, lakes, and groundwater [25]. To collect rain, the water should run off across long-distance interactions and be exposed to various xenoestrogens.

The pollution of estrogen and xenoestrogen in Jordan should be taken seriously, especially after it has been detected and determined in several places around Jordan, including Mediterranean coastal water [26], Jordan Valley soil [27,28], and the Jordan River [28]. In one study, estrogen was detected in 85% of the samples along the Jordan River at risk concentrations [28]. However, to date, there has been no research to test the presence of sex hormone disruptors (xenoestrogen and xenoandrogen) pollutants in the Jordanian water system.

## 2. Materials and Methods

### 2.1. Chemicals

Chemicals were purchased from Sigma-Aldrich, and 17-β-estradiol stock solutions were prepared in methanol (1 µg/µL) and stored at −20°C.

### 2.2. Sample Locations

**Figure 1:**
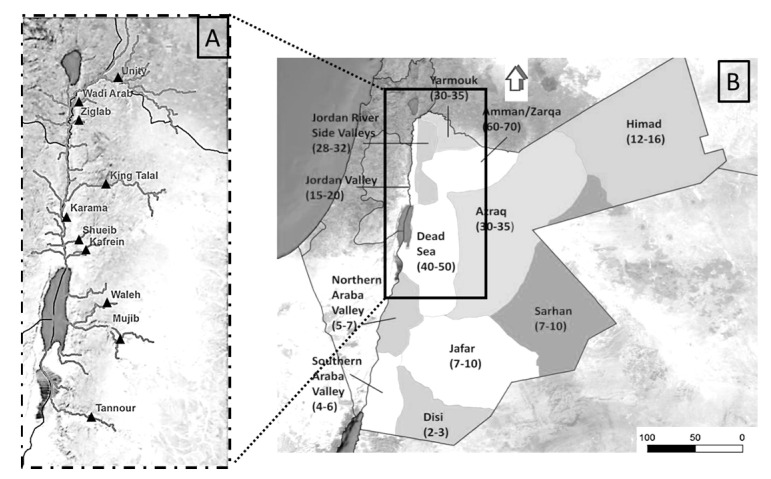
Summary of the Jordanian water system. (**A**) Surface water including dams, rivers, and streams. (**B**) Groundwater basins and their annual safe yield in millions of cubic meters [33].

**Figure 2:**
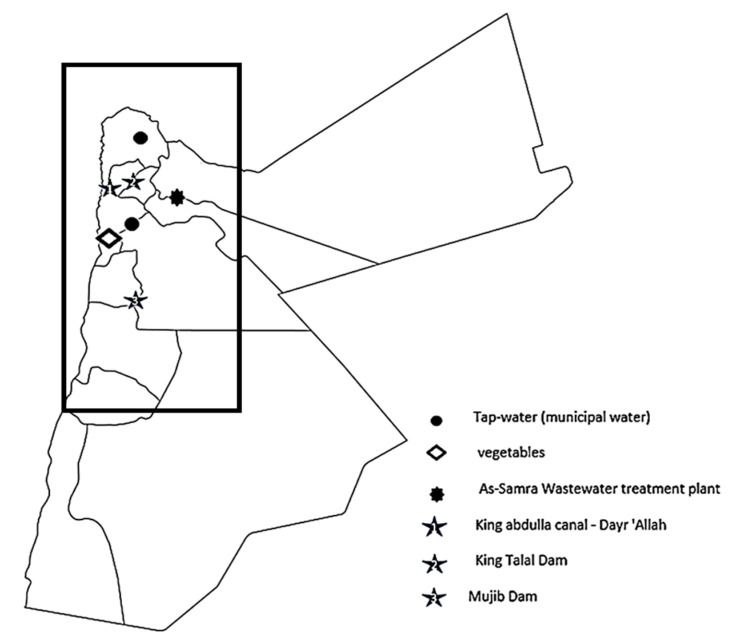
The distribution of sample collection sites.

The water system in Jordan is complex and composed of surface water (rivers, streams, dams) and groundwater basins (Figure 1). The Jordanian water system was discussed in detail previously [29,30,31,32]. Due to the government constraints that restrict access to water resources, the Ministry of Water and Irrigation determined the quantity and location of collected samples. Samples were collected in triplicate according to the following Table 1 and map (Figure 2).

### 2.3. Sample Collection

Water sample collection was conducted as described previously [34]. All glassware was washed twice with methanol, then distilled water, and baked at 180 °C for 4 h. Samples were collected in 2 L precleaned amber glass bottles containing 0.5 g of copper (II) nitrate and 6 mL of 3.6 M hydrochloric acid solution before being stored at 4 °C in the dark. To enhance the solubility of the lipophilic pollutants, 5% methanol was added to the samples. All samples were collected from the edge of the canal or dam from a deep point of around 1 m. Two other samples were also studied: tap water and drinking water. Tap water was collected from Irbid and Amman. The term “drinking water” refers to water sold at purifying stations that began with tap water. The vegetables were purchased from the local market in Irbid.

### 2.4. Sample Preparation

#### 2.4.1. Water

Samples were filtered through glass fiber or glass wool (pore sizes of 0.3–1.2 µm) before solid-phase extraction was carried out. The filtered samples were loaded with a flow rate of 5.5–6 mL/min into reactivated reversed-phase C-18 cartridges (8 mL methanol, and then 8 mL of water:methanol solution (95:5). Later, the cartridges were washed with 10 mL of methanol in water (1:1), followed by 10 mL of acetone in water (1:2).

There were two methods: one for the analysis using yeast estrogen assay (YES) and the other for liquid chromatography–mass spectrometry (LC-MS) analysis.

Samples for YES: 100 µL DMSO was added, and the acetone and methanol were evaporated under a gentle stream of nitrogen.

Samples for LC-MS screening: samples were dried under nitrogen gas (99.99%), reconstituted in 100 μL methanol, completed to 50 mL by acetonitrile, and then centrifuged at 4000 rpm for 2.0 min. Finally, 1.0 mL of the sample was transferred to the autosampler.

#### 2.4.2. Vegetables

The sample preparation was conducted via salting-out assisted liquid–liquid extraction (SALLE) as described previously [35]. Five milliliters of the sample solution was spiked with a standard solution containing the target analytes and transferred to a 15 mL screw-capped test tube. The pH of the solution was adjusted to 7.4 by adding 0.1 M NaOH, followed by 2.40 mL acetonitrile and 1.6 g NaCl. Following that, the solution was gently shaken for 2 min before being centrifuged at 4000 rpm for 5 min to cause phase separation. The upper organic phase was then carefully withdrawn with a 1 mL microsyringe. This volume was approximately 100 ± 25 µL, which was poured into a vial to avoid an anomalous peak in the HPLC chromatogram. A nitrogen stream was blown at this stage to dry it at room temperature. The final residue was reconstituted to a volume of 100 µL using a mobile phase, shaken for 2 min, and filtered through a 0.2 µm nylon filter before being injected into the HPLC system.

### 2.5. Yeast Screening Assays

Assays were conducted as described previously [36,37,38,39] using yeast-based microplate assay (XenoScreen YES/YAS, Xenometrix, Switzerland). Briefly, minimal medium and medium components were prepared according to the manual. Cells were incubated with serially diluted substances and positive control (17-β estradiol for YES assay) for 48 h at 32 °C in the presence of a substrate for β-galactosidase synthesis. All tested samples and the color change and growth of yeast were quantitatively measured using a BioTek Synergy HTX microplate reader at 570 and 690 nm wavelength, respectively. The results were evaluated in terms of the agonistic and antagonistic effects of estrogen. In addition, the cytotoxic effect was evaluated for the test compound by testing the optical density of each well (λ = 690 nm). Assessment of estrogen–androgen activity was performed for the samples at eight dilution levels. Each assay was repeated three times.

### 2.6. Data Analysis

Data analysis was conducted as described previously [40]. Dose–response curves were graphed, and then EC50 and IC50 values were calculated for those compounds that exhibited a complete dose–response curve. The agonistic endocrine activity was considered if the tested sample had an induction of at least 10% of the difference between the maximum E2 response and solvent control in agonist assay. The E2 equivalents corresponding to the measured concentrations were calculated as follows: 5 ng/L of E2 is equal to 1 relative activity in the YES assay. One-way ANOVA was performed for the analysis of the variance first, followed by Dunn’s test (if a significant difference was found). Statistical analysis was conducted using GraphPad Prism 5.00 (GraphPad Software, San Diego, CA, USA.

### 2.7. LC-MS/MS Analysis

All samples were analyzed using LC-MS/MS. Assays were conducted as described previously [36]. For analysis, Elute UHPLC coupled with a Bruker impact II QTOFMS (Bremen, Germany) was used. Chromatographic separation was performed using Bruker solo 2.0C-18 UHPLC column (100 mm × 2.1 mm × 2.0 μm) at a flow rate of 0.5 mL/min and a column temperature of 40 C. The solvents were (A) water with 0.05% formic acid and (B) acetonitrile. A linear gradient from 5% to 80% B over 27 min, followed by two min 95% B. The total analysis time was 35 min in positive and 35 min in negative mode and the injection volume was 3 µL.

The instrument was operated using the Ion Source Apollo II Ion Funnel electrospray source. The capillary voltage was 2500 V, the nebulizer gas was 2.0 bar, the dry gas (nitrogen) flow was 8 L/min and the dry temperature was 200 °C. The mass accuracy was ˂1 ppm, the mass resolution was 50,000 FSR (full-sensitivity resolution) and the TOF repetition rate was up to 20 kHz.

Standards for identification of ms/z with high-resolution Bruker TOF MS and the exact retention time of each analyst after chromatographic separation was used. Later, all possible hits were identified using MetaboScape software (more than 4000 compounds) which includes a list of potential pharmaceutical pollutants, a list of potential veterinary drug pollutants, a list of potential insecticides, pesticides, and herbicides pollutants, and an additional special library (constructed from chemicals reported in the literature) of possible xenoestrogens. All pollutants are listed in Supplementary Material S1–S4. The detection of xenoestrogens in tap water were used as a model for the LC-MS experiment.

## 3. Results and Discussion

### 3.1. Study Area

Due to the governmental restriction from the Ministry of Water and Irrigation, the research was restricted to designated sites: two dams (King Talal Dam and Almujeb Dam), one wastewater treatment plant (As-Samra), and one canal (King Abdullah canal). King Talal Dam’s (the largest dam in Jordan) main purpose is to retain winter rainfall and treated wastewater processed at the As-Samra Wastewater Treatment Plant and is used for domestic, agricultural, and industrial uses and to control floods, improve drainage, and collect water from rivers and streams [41,42], while Almujeb Dam only collects rainwater and is used for domestic, industrial supply and irrigation [43]. As-Samra Wastewater Treatment Plant was built to improve the quality of water in Jordan. It treats wastewater released from the Zarqa River Basin. Moreover, the facility treats an average flow of 267,000 m^3^ of wastewater, serving a population of 2.2 million living in the Amman and Zarqa areas [44]. King Abdullah Canal is Jordan’s most important source of drinking and agricultural water. The canal is approximately 110 km long, with a head discharge capacity of 20 m^3^/s and a tail discharge capacity of 6 m^3^/s. [45]. Dair Alla Station is responsible for pumping water from the canal to Amman [45].

### 3.2. Estrogenicity in Water Samples

The estrogenic activity via yeast screening assay of each water sample measured as 17β-estradiol relative estrogenicity is summarized in Table 2. The relative estrogenic activities of all samples were comparable to those of other yeast assays [28,46,47,48].

#### 3.2.1. Tap Samples

Jordan’s primary sources of tap water are surface water and groundwater wells [49]. For example, 45% of tap water in Amman comes from the Disi aquifer, 29% from the Zay treatment plant, which uses water from the King Abdullah Canal, 16% from the Zara treatment plant, which uses water from the Almoujib Dam, and 10% from a network of subterranean wells [50].

The relative estrogenicity of tap water (1.4 ± 0.9 ng/L) is lower than that reported in other countries [51,52,53,54]. According to reports, such concentrations do not constitute an immediate, acute health risk to the community [51,55]. However, the potential long-term impact of xenoestrogens on human health and the environment at low concentrations is yet to be elucidated [56]. Hence, xenoestrogens have been categorized as an “unquantified risk” [55]. The World Health Organization (WHO) has reported that xenoestrogens in such low concentrations are potentially less harmful to human health, but emphasized the necessity to limit the existence of such compounds in the water [56,57].

In the LC-MS experiment, a high-resolution database included exact mass data for parent ions, adducts, fragment ions as well as isotopic pattern and retention time. Figure S1 represents the base peak chromatogram (all MS and bbCID) for samples from both Irbid and Amman. The complete spectrum list of (Dissect and bbCID) for both Amman and Irbid are shown in Tables S1 and S2, respectively. However, only 34 compounds were identified as potential xenoestrogen or estrogen disturbances (Table 3). It is worth mentioning that around 100 compounds are still unknown in each sample.

**Figure 3:**
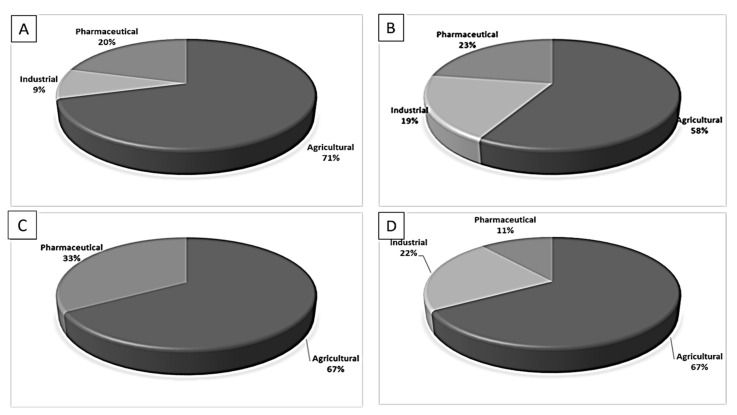
The distribution of potential sources of detected xenoestrogens in: (**A**) tap water, (**B**) open surface water, (**C**) drinking water, and (**D**) vegetables. The percentage in each sector represents the ratio of the overall number of xenoestrogens in a particular sector to the total number of xenoestrogens across all sectors.

The potential source of each xenoestrogen (as shown in Table 3) was classified into three main categories: agricultural, industrial, and pharmaceutical. In tap water, agricultural sources accounted for the majority of xenoestrogens (>70%), which include pesticides and fungicides, as shown in Figure 3. Between Amman and Irbid, minor variations were spotted, mostly in the pharmaceutical sector (such as sunscreens). According to the chemical structures of identified xenoestrogens, 94% of them were either aromatic or polycyclic with low water solubility, with only prochloraz and pregabalin exceptions. Moreover, only three of the identified xenoestrogens contained steroid rings (betamethasone, hydrocortisone, and protopanaxadiol).

The presence of xenoestrogens might result from either direct contact with water at the source, pipeline, or reservoir, or from by-products of water treatment. Jordan’s water supply network is plagued by issues such as seepage inside distribution pipe systems and intrusions (illegal use). In both circumstances, an interaction between xenoestrogens and tap water is feasible [31]. It appears that the majority of the purposeful sabotage of the water network occurred in agricultural areas, which may explain the prevalence of pesticides and fungicides. Furthermore, water losses in the Jordanian system were estimated at 50% [31].

In Jordan, water is disinfected using various disinfectants (such as chlorine), which may cause interactions with organic matter to produce disinfection by-products (halocarbon compounds) [92]. Natural organic matter (NOM) is an enormously complicated mixture of organic molecules with widely varying physical and chemical properties. NOM is both a natural occurrence in the environment and a by-product of human activity. NOM is present in particle, colloidal, and dissolved states in all ground and surface waters, including rainwater [93]. Furthermore, the infrequent usage of pipelines facilitates such reactions, particularly at the periphery networks (6 h per week). Seven halocarbon xenoestrogens were identified in the tap water samples (Table 3).

#### 3.2.2. Surface Water

The data show that open-source water has estrogenic activity of 2.5–9.5 ng/L and is not far from most countries in the world, including bordered countries [28,46,47,48]. It has been reported that the estrogenic activity of surface water typically should be below 25 ng/L, including the effluent of the water treatment plant [94,95].

Raw wastewater has different characteristics in Jordan from most other countries. According to reports, Jordan’s wastewater is very strong, highly salinized, contains heavy metals, and contains toxic organic compounds. Furthermore, Jordan’s raw wastewater contains large organic contents resulting from low home water consumption and industrial waste [96].

The estrogenic activity of the effluent of As-Samra treatment plant was 9.5 ± 5 ng/L. This value is not far from recorded around the world. For instance, the estrogenic activity was 0.4–6.6 ng/L in England, 5–10.6 ng/L in the USA, 5.6–11 ng/L in Germany, 2.9–7.5 ng/L in Italy, 17.9 ng/L in Switzerland and 29–65 ng/L in Brazil [95,97,98]. According to reports, estrogenic activity in treatment plant effluent is deemed high enough to cause issues with public health if it exceeds 15 ng/L [99]. Hence, there are no xenoestrogen threats from the water treatment plant. However, it has been reported that As-Samra treatment plant needs further improvements [100]. More about the concentration of estrogenic activity in the surface water is found in a review [101].

The distribution of detected xenoestrogens sources in the open-source water is shown in Figure 3. Still, agricultural resources are the main source of xenoestrogens with 58%. The potential xenoestrogens in each site are summarized in Table 4; nonetheless, more than 100 compounds remained unidentified in each sample. The number of detected xenoestrogens was 12, 18, 23, and 30 for Almujeb, King Abdullah Canal, King Talal Dam, and As-Samra treatment plant, respectively. According to the chemical structures of xenoestrogens, 97% of them are aromatic with low water solubility. Only pregabalin is the exception. Moreover, no xenoestrogens containing steroid rings were detected in the samples, which implies there is no direct pollution from pharmaceutical drugs.

It is clear that Almujeb water has the least detected compounds and that may be explained by the location, where the dam and collected water are far from human pollution, such as heavy traffic and industrial areas [102]. Therefore, the estrogenic activity was below the detection limit.

On the other hand, King Talal Dam has easy access to people and is located in a crowded place surrounded by farms and agricultural areas. Furthermore, the dam retained the treated wastewater processed in the As-Samra Wastewater Treatment Plant. Hence, the pollutants from residents around the dam, the farms, and the treated wastewater augment the pollution in King Talal Dam. This may explain the presence of herbicides, pharmaceutical compounds, and natural products.

It has previously been claimed that some chemicals, such as phenolic compounds, may be released from the pharmaceutical sector or other companies engaged and located in King Talal Dam’s catchment area [103]. Moreover, it has been reported that more than 100 organic compounds were detected using GC-MS at various concentrations in King Talal Dam. The reported compounds fit well with our findings [103]. Furthermore, it has been reported that King Talal Dam has alarming biological pollution [103].

In case of King Abdullah Canal, residents toss waste and trash into and around it, and the presence of intermittent gatherings of migrant workers who live in the neighborhood exacerbates the problem. The continuous dumping of waste and its accumulation may explain the presence of such pollutants in the canal. Moreover, previous incidents of contamination have been reported [29]. It has been reported that the water canal is not safe for domestic use and needs further treatment, especially in the lower part of the canal [45].

A possible explanation for the low level of xenoestrogens in all samples is that the sample collections were conducted during the winter, which may dilute the xenoestrogens. In addition, degradation, evaporation, and adsorption are potential mechanisms contributing to lowering the activity of xenoestrogens [44].

**Table 4:** Detected xenoestrogens in open surface water.

Expected Compound	As-Samra	King Talal Dam	Almujeb Dam	King Abdullah Canal	Origin ^3^	Ref. ^2^	Chemical Structure ^1^
3 5 6-Trichloro-2-pyridinol (TCPy)	Yes	Yes	Yes	Yes	Pesticide	[104]	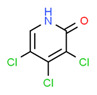
Isoferulic acid	Yes	-	-	-	Natural products	[105]	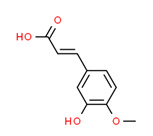
Acacetin	Yes	Yes	Yes	-	Natural products	[106]	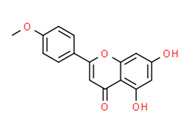
5-Hydroxy Mebendazole	Yes	-	-	-	Pharmaceutical	[107]	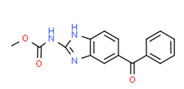
Acetamiprid	-	-	-	Yes	Pesticide	[108]	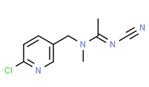
Alpha-zearalenol	Yes	Yes	Yes	Yes	Natural product	[109]	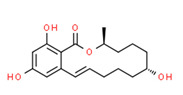
Bisphenol A	Yes	Yes	-	Yes	Polycarbonate plastic	[65]	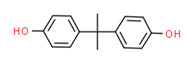
Caffeic Acid	Yes	-	Yes	-	Natural products	[110]	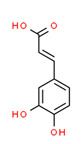
carbamazepine	Yes	Yes	-	Yes	Pharmaceutical	[111]	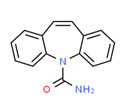
Carbendazim	Yes	-	-	-	Fungicide	[67]	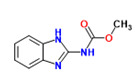
Cotinine	Yes	-	-	-	Metabolite	[112]	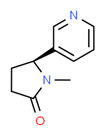
Diazinon	-	Yes	-	-	Pesticide	[113]	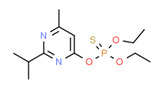
Dibutylphthalate	Yes	Yes	Yes	Yes	Plasticizer	[114]	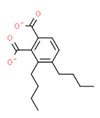
Dicofol	Yes	Yes	-	-	Pesticide	[115]	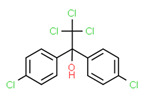
Fenamiphos	Yes	Yes	-	Yes	Pesticide	[116]	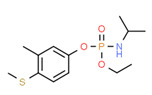
Ferimzone	Yes	Yes	Yes	Yes	Pesticide	[117]	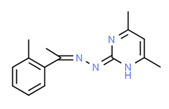
Ferutinine	Yes	Yes	Yes	Yes	Natural product	[75]	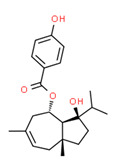
Halofenozide	Yes	Yes	-	Yes	Pesticide	[118]	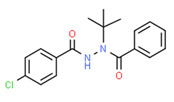
Hexazinone	Yes	Yes	Yes	Yes	Pesticide	[119]	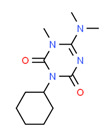
Isobavachin	Yes	Yes	Yes	Yes	Natural product	[120]	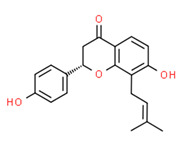
Isosakuranetin	Yes	Yes	-	Yes	Natural product	[121]	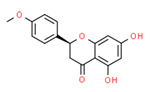
Levamisole	Yes	-	-	-	Pharmaceutical	[122]	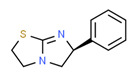
Lidocaine	Yes	-	-	-	Pharmaceutical	[123]	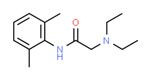
Nitenpyram	Yes	-	-	-	Pesticide	[124]	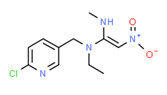
Phthalic acid	Yes	Yes	-	Yes	Plasticizer	[125]	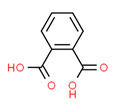
Phthalic Acid Bis(2 Ethylhexyl) Ester	Yes	Yes	-	Yes	Plastic-softening agent	[126]	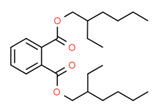
Pregabalin	Yes	Yes	-	-	Pharmaceutical	[85]	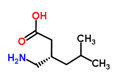
Salicylic acid	Yes	Yes	-	-	Pharmaceutical	[127]	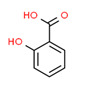
Tebuconazole	Yes	Yes	Yes	Yes	Pesticide	[128]	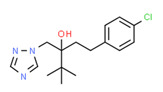

^1^ Marvin Sketch was used to draw the chemical structures. ^2^ Citation describing the estrogenic activity of the identified xenoestrogen. ^3^ Potential source of the xenoestrogen.

#### 3.2.3. Drinking Water

Regarding drinking water, private stations have more purification systems with additional filters: sand filter, carbon filter, iron removal filter, and microfilter. In addition, the stations perform sterilization using ultraviolet radiation. Hence, the potential estrogenicity was low, below the detection limit of the YES assay (1 ng/L). However, several compounds (potential xenoestrogens) were detected by LC-MS and identified by the MetaboScape database (Table 5).

Despite estrogenic activity not being detected in drinking water using YES, the existence of xenoestrogen cannot be ruled out, because its concentration was below the YES detection limit. More sensitive screening methods have recently been developed, which can detect levels as low as 14.7 pg/L of estrogen equivalents [129]. Estrogenic-disrupting substances have been found in drinking water all around the world [99], including the USA [130], Germany [131,132], India [133,134], Italy [132], Sweden [54,135], France [132] and Spain [136]. According to the Environmental Protection Agency (US-EPA), the estrogenic risk is significant when the estrogenic activity is greater than 1 ng/L [137].

Detected xenoestrogens are listed in Table 5. It is anticipated that xenoestrogens will originate from the several sources depicted in Figure 3. Agriculture was responsible for the production of 67% of all xenoestrogens.

Carvone is a monoterpene compound that is widely used as an insecticide, fungicide, antioxidant, and plant growth regulator [138]. For instance, it is currently used as a potato germination inhibitor [139]. Carvone is easily volatilized, so it is not a persistent component in soil or water. Furthermore, when exposed to light, the volatilized carvone will undergo photochemical reactions [138]. This finding may question the source of untreated water that is used in private stations.

Mercaptobenzothiazole, in addition to being utilized in pesticide manufacturing, is also employed as a sulfur vulcanization accelerator in the synthesis of rubber plumbing items such as gaskets and O-rings, which are essential parts of water networks [140]. Mercaptobenzothiazole could therefore have reached drinking water through private station pipelines during filtration or bottle packaging. Both (carvone and mercaptobenzothiazole) have been detected in drinking water around the world [80,140,141].

**Figure 4:**
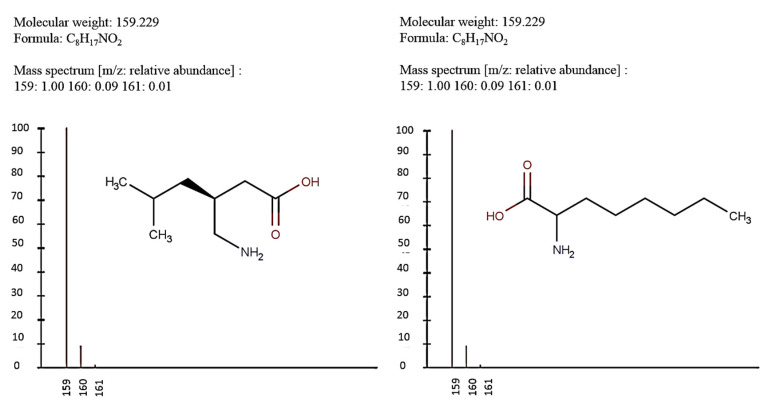
Elemental analysis of pregabalin (**left**) and DL-2-aminooctanoic acid (**right**), generated using Marvin Sketch.

The existence of pregabalin, which is used to treat neuropathic pain and convulsions, remains unknown. Pregabalin was detected in all water samples; however, its half-life has been reported to be 8 to 10 days in the aerobic environment [142], implying a potential error in the detection experiment. The detection system employs four different identification methods: mass accuracy, retention duration, diagnostic ions, and isotopic pattern. Pregabalin only satisfied the retention time and mass accuracy detection requirements. DL-2-aminooctanoic acid, also known as alpha-aminocaprylic acid, has the same molecular weight (159.229) and chemical formula (C_8_H_17_NO_2_) as pregabalin (Figure 4). Hence, it has identical MS data. According to FooDB (www.foodb.ca, accessed on 25 October 2022.), DL-2-aminooctanoic acid has been identified in various foods, including chicken and cow milk, and has been used as a potential biomarker for the consumption of these foods. As a result, it is more likely that the pollutant came from food metabolites rather than the anticonvulsant medicine, especially as DL-2-aminooctanoic acid is not found in the MetaboScape library. Although it is unlikely to have pregabalin in drinking water as explained previously, it has been detected in several rivers around the world [143].

The identification of xenoestrogen contaminants in the water does not always imply direct estrogenic activity, because the mass spectrometry detects traces of xenoestrogens and the estrogenic activity is concentration-dependent [144]. Although more than 700 xenoestrogens have been identified in drinking water [8], only 11 xenoestrogen are regulated by the United States Environmental Protection Agency (USEPA) [8]. As such, scientists pay attention to the fact that xenoestrogens are often found in the raw water that is used to make drinking water, and calls are made for more government control and regulations [55,145].

#### 3.2.4. Vegetables

The presence of xenoestrogens in vegetables has been previously reported [146]. Consequently, vegetable samples were utilized to investigate the potential transfer of xenoestrogens from irrigation water to vegetables. Vegetable samples were not used in the YES experiment because plants contain phytoestrogens, which are known to interact with estrogen receptors [147]. Detected xenoestrogen in vegetables (tomato and cucumber) is summarized in Table 6, which was also detected in open surface water. The majority of vegetables are cultivated in the Jordan Valley, and farmers use the King Abdullah Canal and King Talal Dam water for irrigation, so either the xenoestrogen in the canal water contaminated the crops or the pesticides used by farmers contaminated both the crops and the King Abdullah Canal. Agricultural resources are responsible for 67% of the xenoestrogens in vegetables, as illustrated in Figure 3. The other 23% may come from industrial pollution during cultivation and shipping processes, as dibutyl phthalate was identified in the samples. All detected xenoestrogens in vegetables samples were aromatic with low water solubility Table 6.

The *Ferula* genus (Umbelliferae) contains the phytoestrogen ferutinin, which has a modest estrogenic activity [148] and is native to Jordan [149]. Therefore, it was probably the surrounding plants that allowed it to reach the crops. Moreover, ferutinin was detected in all water samples (except drinking water).

Butylparaben—a preservative used in cosmetic products—is not prone to photodegradation and is highly stable against sunlight; however, it is suitable for biodegradation and sorption [150]. The expected source is direct contamination from the surrounding area, not from water, as it was not detected in any water samples. Moreover, butylparaben has weak estrogenic activity [150].

Dibutyl phthalate (DBP) is a plasticizer used to improve the flexibility of plastic products, specifically polyvinyl chloride, which is used in the synthesis of packaging/greenhouse films, wires, pipes, and all flooring materials [151]. Moreover, phthalate plasticizers are not chemically bound to the polymer structure and have a high probability of being released into the environment [152]. Hence, DBP may contaminate the crops directly during farming (plastic greenhouse), cultivation (plastic pipelines), and shipping (plastic packaging). In addition, there is another possible source that DBP came from water, as DBP was detected in all surface samples from water used for irrigation. Dibutyl phthalate has moderate estrogenic activity [153].

Alpha-zearalenol (α-ZEA) is a metabolite of mycotoxin zearalenone that is widespread, particularly in pathogens (*Fusarium* species) of small grain cereals and corn, and might be developed under poor storage conditions [154]. Furthermore, zearalenone is thermostable and is not degraded by processing, such as milling, extrusion, storage, or heating [155]. Alpha-zearalenol is mainly formed in the liver and the small intestines of humans and animals. Therefore, it has been detected in animal body fluids such as milk and urine [155], and has also been identified in foods worldwide, such as cow’s milk-based infant formula [156], chicken heart [157], and fish meat [158]. Hence, the source of α-ZEA in vegetables mainly came from animals such as cows and chickens. This theory is supported by the fact that α-ZEA was detected in all open surface water samples. Alpha-zearalenol possesses 60 times the estrogenic activity of zearalenone [159].

Ferimzone is a systemic pyrimidine fungicide and it has been detected in surface water [160] and groundwater [161] worldwide, so the presence of ferimzone in samples was due to the direct use on the vegetables. Ferimzone has weak estrogenic activity [117], and it was detected in all surface waters.

Tebuconazole is an azole fungicide used in vegetables, citrus, and field crops [162]. Moreover, it has high photochemical stability, very slow photodegradation, and slow microbial-mediated degradation in soil [163,164]. Tebuconazole was detected in drinking water and groundwater [162,165]. Tebuconazole exhibits moderate estrogenic activity [166]. Direct application on the plant may the source of contamination in vegetables rather than the irrigation water.

3,5,6-Trichloro-2-pyridinol (TCP) is a metabolite of chlorpyrifos [104], which is an organophosphate insecticide. It has been reported that TCP can reach groundwater and surface water, and its half-life in the soil can reach 120 days [167]. Therefore, the direct application of TCP might be the source of contamination [142,143,144,153,154,155,156,157,158,159,160,161,162,163,164,165,166,167]. Moreover, it has weak estrogenic activity, being 2500 times less estrogenic than 17β-estradiol [104].

The source of xenoestrogens in the vegetable samples was predominantly from direct interaction throughout the farming process (cultivation, irrigation, packing), and the source of xenoestrogens discovered in water was most likely from agricultural resources rather than the other way around. Traces of pesticides have previously been detected in Jordanian fruit and vegetables [169,170]. Furthermore, some of these pesticides were also found in water and soil. As a result, the necessity for proper training and enforcement of good agricultural practices in the region was advised. Comprehensive pesticide monitoring systems and frequent inspections were also highlighted [170].

## 4. Conclusions

Xenoestrogens are persistent, prevalent substances in the environment that accumulate and may even be further activated by biotransformation, making them hazardous to animal and human health. Xenoestrogens were detected in all water and vegetable samples, including drinking water. However, the estrogenic activity was low and does not constitute an immediate, acute health risk to the community, though there is a necessity to limit the existence of such compounds in the water. The main source of xenoestrogens was agricultural resources (pesticides, insecticides, fungicides). Therefore, proper training and implementation of good agricultural practices should be established, and comprehensive pesticide monitoring systems and frequent inspections should also be enforced. This research may serve as a whistleblower on the estrogenic contamination in the Jordanian water system.

## 5. Limitations and Future Work

Six locations are insufficient to provide adequate data regarding xenoestrogen pollution in the Jordanian water system. The Ministry of Water and Irrigation restricted access to water resources and barred the collection of samples freely. As a result, the number of samples from each location was limited and restricted to certain sites. Furthermore, surface water samples were collected and prepared at the ministry labs, and the samples were subsequently analyzed in the university labs.

Future studies must be more comprehensive and include more samples from various locations at each site. Even though there were more than 4000 compounds in the MetaboScape database, still there were many unidentified compounds in the samples. Hence, future work might focus on specific families of xenoestrogens, where the xenoestrogens could be quantified.

## Figures and Tables

**Table 1:** Sample collection sites.

Name	Samples Collection by
King Talal Dam	MWI ^1^
Almujeb Dam	MWI
As-Samra Wastewater Treatment Plant	MWI
King Abdullah Canal: Dair Alla	MWI
Tap water	Researchers
Drinking water	Researchers

^1^ MWI: Ministry of Water and Irrigation.

**Table 2:** The estrogenic activity using the YES assay.

Name	Total 17β-Estradiol Equivalents (ng/L)
King Talal Dam	2.9 ± 1.2
Almujeb Dam	ND
As-Samra Wastewater Treatment Plant	9.5 ± 5
King Abdullah Canal: Dair Alla	2.5 ±1.5
Tap water	1.4 ± 0.9
Drinking water	ND

**Table 3:** Detected xenoestrogens in tap water.

Expected Compound	Irbid	Amman	Origin ^3^	Ref. ^2^	Chemical Structure ^1^
3-Methylcholanthrene	Yes	Yes	Pesticide/fungicide	[58]	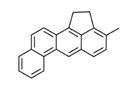
4-Hydroxybenzophenone	Yes	Yes	Intermediate of clomiphene	[59]	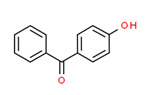
7,12-Dimethyl-benz(a)anthracene	Yes	Yes	Incomplete combustion of gasoline and coal	[60]	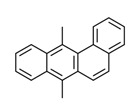
Aldrin	Yes	Yes	Banned insecticide	[61]	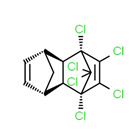
Benomyl	Yes	Yes	Fungicide	[62]	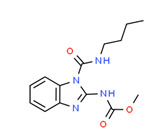
Benzophenone-3	-	Yes	Sunscreen agent	[63]	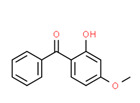
Betamethasone valerate	Yes	Yes	Corticosteroid	[64]	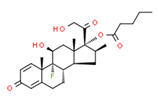
Bisphenol A	Yes	Yes	Manufacturing of various plastics	[65]	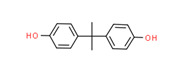
Carbanilide	Yes	Yes	Pesticide	[66]	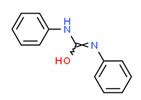
Carbendazim	Yes	Yes	Fungicide	[67]	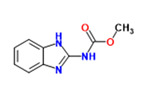
Carvone	Yes	Yes	Insect repellent	[68]	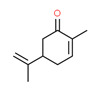
Cyhalothrin	Yes	Yes	Pesticide	[69]	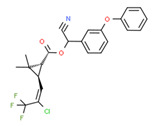
Cyprodinil	Yes	Yes	Fungicide	[70]	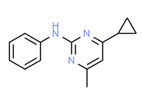
Dimethomorph	Yes	-	Fungicide	[71]	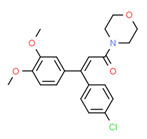
Dodemorph II	Yes	Yes	Pesticide	[72]	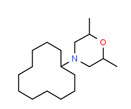
Fenarimol	Yes	Yes	Fungicide	[73]	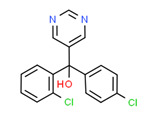
Fenitrothion	Yes	Yes	Pesticide	[74]	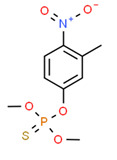
Ferutinin	-	Yes	Natural product	[75]	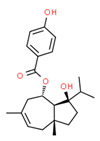
Fludioxonil	Yes	Yes	Fungicide	[76]	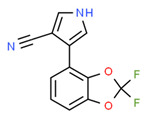
Hydrocortisone	Yes	Yes	Hormone cortisol	[77]	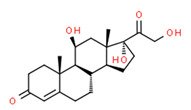
Imazalil	Yes	-	Pesticide	[78]	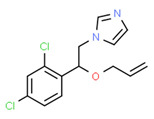
Isopentyl-4-methoxycinnamate	-	Yes	Sunscreen product	[79]	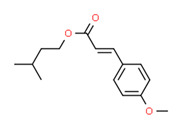
2-Mercaptobenzothiazole	-	Yes	Pesticide	[80]	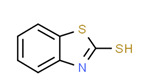
Metolachlor	Yes	Yes	Herbicide	[81]	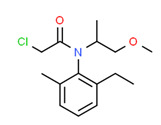
Octocrylene	-	Yes	Sunscreen product	[82]	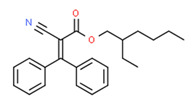
Para amino-benzoic acid	-	Yes	Sunscreen product	[83]	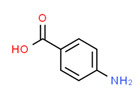
Permethrin	Yes	Yes	Insecticide	[84]	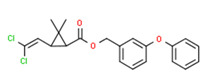
Pregabalin	Yes	Yes	Pharmaceuticals	[85]	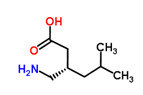
Prochloraz	Yes	Yes	Fungicide	[86]	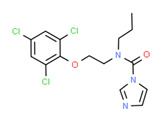
Propamocarb	Yes	Yes	Fungicide	[87]	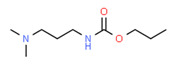
Protopanaxadiol	Yes	Yes	Natural product	[88]	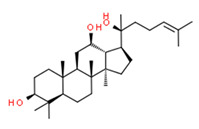
Pyrimethanil	Yes	Yes	Fungicide	[89]	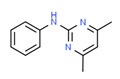
Temephos	Yes	Yes	Organophosphate larvicide	[90]	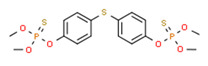
Tetramethrin	Yes	Yes	Insecticide	[91]	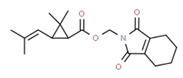

^1^ Marvin Sketch was used to draw the chemical structures. ^2^ Citation describing the estrogenic activity of the identified xenoestrogen. ^3^ Potential source of the xenoestrogen.

**Table 5:** Detected xenoestrogens in drinking water.

Expected Compound	Origin ^3^	Ref. ^2^	Chemical Structure ^1^
Carvone	Insect repellent	[68]	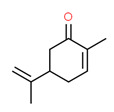
2-Mercaptobenzothiazole	Pesticide	[80]	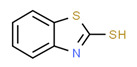
Pregabalin	Pharmaceutical	[85]	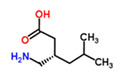

^1^ Marvin Sketch was used to draw the chemical structures. ^2^ Citation describing the estrogenic activity of the identified xenoestrogen. ^3^ Potential source of the xenoestrogen.

**Table 6:** Detected xenoestrogens in vegetables.

Expected Compound	Origin ^3^	Ref. ^2^	Chemical Structure ^1^
Ferutinin	Natural product	[75]	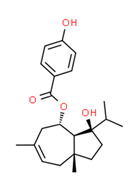
Butylparaben	Sunscreens	[168]	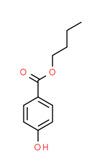
Dibutylphthalate	Plasticizer	[114]	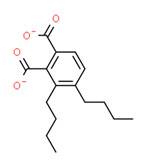
Alpha-zearalenol	Natural product	[109]	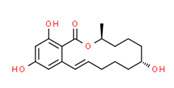
Ferimzone	Fungicide	[117]	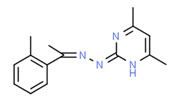
Tebuconazole	Fungicide	[128]	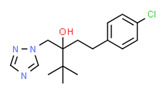
3 5 6-Trichloro-2-pyridinol	Pesticide	[104]	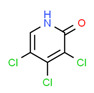

^1^ Marvin Sketch was used to draw the chemical structures. ^2^ Citation describing the estrogenic activity of the identified xenoestrogen. ^3^ Potential source of the xenoestrogen.

## Data Availability

The datasets used and/or analyzed during the current study are available from the corresponding author upon reasonable request.

## Supplementary Materials

The following supporting information can be downloaded at https://www.mdpi.com/article/10.3390/toxics11010063/s1. Figure S1: The base peak chromatogram for tap water samples from Irbid and Amman; Table S1: The complete spectrum list of (Dissect and bbCID) for Irbid’s tap water sample; Table S2: The complete spectrum list of (Dissect and bbCID) for Amman’s tap water. Supplementary Material S1: List of potential pharmaceutical pollutants (including psychoactive substances) and their metabolites—a total of 1659 compounds. The list includes the name and CAS number of each compound. Supplementary Material S2: List of potential veterinary drug pollutants—a total of 206 compounds. The list includes the name and CAS number of each compound. Supplementary Material S3: List of potential insecticides, pesticides, and herbicides pollutants—a total of 1060 compounds. The list includes the name and CAS number of each compound. Supplementary Material S4: List of potential xenoestrogens according to the literature—a total of 75 compounds. The list includes the name, chemical formula, molecular weight, and CAS number of each compound, as well as occurrence and use.
