# Supplementary material for: Assessment of Xenoestrogens in Jordanian Water System: Activity and Identification
Source: Toxics. 2023 Jan 9;11(1):63. doi: 10.3390/toxics11010063 (PMC9866086; doi:10.3390/toxics11010063)
Supplement: Supplementary file 1 [file toxics-11-00063-s001.zip › Figure S1.pdf]

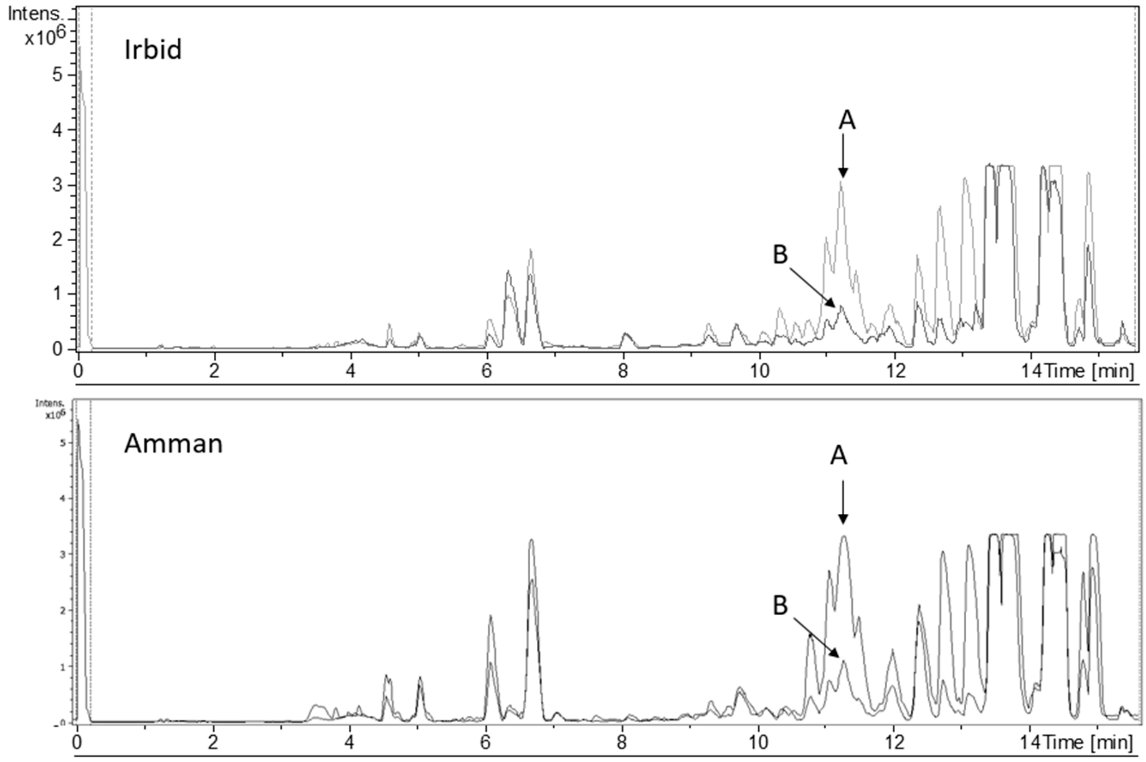

Figure S1. The base peak chromatogram for tap water samples from Irbid and Amman. A) All MS. B) Broadband collision-induced dissociation
