# Supplementary material for: Assessment of Xenoestrogens in Jordanian Water System: Activity and Identification
Source: Toxics. 2023 Jan 9;11(1):63. doi: 10.3390/toxics11010063 (PMC9866086; doi:10.3390/toxics11010063)
Supplement: Supplementary file 1 [file toxics-11-00063-s001.zip › Supplementary Material S1.pdf]

Supplementary Material S1

List of Potential Pharmaceutical pollutants (including psychoactive substances) and their metabolites

	Name	CAS Number
1	1-(2-Methoxyphenyl)piperazine	(35386-24-4)
2	1-(4-methoxyphenyl)piperazine. MeOPP	(38212-30-5)
3	10-Hydroxycarbamazepine	(29331-92-8)
4	17-alpha-Methyltestosterone	(58-18-4)
5	1-Hydroxymethyltriazolam. alpha-Hydroxy-Triazolam	(37115-45-0)
6	1-Hydroxymidazolam	(59468-90-5)
7	1-Piperonylpiperazine	(32231-06-4)
8	2 C-D	(24333-19-5)
9	2 C-P	(207740-22-5)
10	2-5-DMA	(2801-68-5)
11	25I-NBF	(919797-21-0)
12	25I-NBoMe	(919797-19-6)
13	2-Amino-5-chlorobenzophenone	(719-59-5)
14	2-Amino-5-nitrobenzophenone	(1775-95-7)
15	2-Aminoindane. 2-AI	(2975-41-9)
16	2-Benzyltetronic acid	(3734-22-3)
17	2C-B	(66142-81-2)
18	2C-B-FLY	(733720-95-1)
19	2C-C	(88441-14-9)
20	2-C-C-NBoMe . 25C-NBoMe	(1227608-02-7)
21	2C-E	(71539-34-9)
22	2C-H	(3600-86-0)
23	2C-I	(69587-11-7)
24	2CT-2	(207740-24-7)
25	2C-T-4	(207740-25-8)
26	2C-T-7	(207740-26-9)
27	2-DPMP (desoxypipradrole)	(519-74-4)
28	2-Fluoroamphetamin	(1716-60-5)
29	2-Fluoroethcathinone. 2-FEC	(na)
30	2-fluoromethcathinone	(1186137-35-8)
31	2-F-Methamphetamine	(1017176-48-5)
32	2-Hydroxyibuprofen	(51146-55-5)
33	2-MEC. 2-Methylethcathinone	(na)
34	2-MeO-Diphenidine. Methoxphenidine	(127529-46-8)
35	2-MMC. 2-Methylmethcathinone	(1246815-51-9.HCl)

36	2-OH-Ethylflurazepam	(19011-77-9)
37	2-Oxo-3-hydroxy-LSD	(na)
38	2-Phenethylamine	(64-04-0)
39	3.4.5-Trimethoxyamphetamine. TMA	(1082-88-8)
40	3.4-Dimethoxyphenethylamine. DMPEA	(120-20-7)
41	3.4-Dimethylethcathinone	(na)
42	3.4-Methylenedioxy-n-benzylcathinone	(1823274-68-5.HCl)
43	3.5-Diiodotyrosine	(66-02-4)
44	3-4-CTMP	(1400742-68-8)
45	3-4-DMA	(120-26-3)
46	3-4-DMMC	(1081772-06-6 HCl)
47	3-Ethylethcathinone	(na)
48	3-F-Amphetamine	(1626-71-7)
49	3-Fluorophenmetrazine	(1350768-28-3)
50	3-F-Methamphetamine	(na)
51	3-F-Methcathinone. 3-FMC	(1049677-77-1)
52	3-Hydroxyphenazepam	(70030-11-4)
53	3-MBZP	(MBZP 374898-00-7)
54	3-MEC. 3-Methylethcathinone	(na)
55	3-MeO-PCP	(72242-03-6)
56	3-Methylfentanyl	(42045-86-3)
57	3-Methylnorfentanyl	(na)
58	3-MMC. 3-Methylmethcathinone	(1246816-62-5.HCl)
59	3-OH-Bromazepam	(13132-73-5)
60	3-OH-Flubromazepam	(62559-65-8)
61	4-Acetamidoantipyrine (4-AAA)	(83-15-8)
62	4-Acetoxy-N-methyl-N-ethyltryptamine	(1445751-40-5)
63	4-AcO-DIPT	(na)
64	4-AcO-DMT. Psilacetin	(92292-84-7)
65	4-Aminoantipyrine (4-AAP)	(83-07-8)
66	4-Benzamidosalicylic acid	(13898-58-3)
67	4-BMC. Brephedrone	(486459-03-4)
68	4-CEC	(22198-75-0)
69	4-CMC. Clephedrone	(1225843-86-6)
70	4-Cyano CUMYL-BUTINACA	(1631074-54-8)
71	4-EMC. 4-Ethylmethcathinone	(1391053-87-4.HCl)
72	4-Ethylethcathinone	(1225619-32-8)
73	4-Ethylmethcathinone	(1225622-14-9)
74	4-Ethyl-N.N-Dimethylcathinone	(na)
75	4F-alpha-PEP. 4F-PV8	(na)
76	4F-alpha-PVP	(850352-62-4)

77	4-F-Amphetamine	(459-02-9)
78	4-Fluoroethcathinone	(1225625-74-0)
79	4-F-Methamphetamine	(52063-62-4 HCl)
80	4-F-Methcathinone. 4-FMC. Flephedrone	(7589-35-7)
81	4-Formylaminoantipyrine (4-FAA)	(1672-58-8)
82	4-MEC	(1225617-18-4)
83	4-MeO-PCP	(2201-35-6)
84	4-MePPP	(1313393-58-6. 28117-80-8)
85	4-Methylaminoantipyrine (4-MAA). 4-Methylaminophenazone	(519-98-2)
86	4-Methylbuphedrone	(HCl.1336911-98-8)
87	4-MeTMP. 4-Methylmethylphenidate	(191790-79-1)
88	4-MMA (Methylmethamphetamine)	(161697-16-1.HCl)
89	4-MTA	(14116-06-4)
90	4-OH MET. Metocin	(77872-41-4)
91	4-OH-DIPT	(63065-90-7)
92	5-(p-Methylphenyl)-5-phenylhydantoin (MPPH)	(51169-17-6)
93	5-Aminosalicylic acid	(89-57-6)
94	5-APB	(286834-80-8)
95	5-APDB	(152624-03-8)
96	5-Carboxybupranolol	(42242-69-3)
97	5-EAPB	(1445566-01-7)
98	5F-AMBICA	(na)
99	5-Fluoro AKB48 N-(4-hydroxypentyl) metabolite	(1843184-42-8)
100	5-fluoro MN-18 (AM2201 Indazol Carboxamide)	(1445581-91-8)
101	5-Fluoro PB-22 3-carboxyindole metabolite	(1432794-98-3)
102	5-fluoro PY-PICA	(na)
103	5F-PB-22. 5-fluoro QUPIC	(1400742-41-7)
104	5-IAI	(132367-76-1)
105	5-IT. 5-API	(3784-30-3)
106	5-MAPB	(1354631-77-8)
107	5-MeO-AMT	(1137-04-8)
108	5-MeO-DALT	(928822-98-4)
109	5-MeO-DIPT	(4021-34-5)
110	5-MeO-DMT	(1019-45-0)
111	5-MeO-MIPT	(96096-55-8)
112	5-MeOPP	(na)
113	5-MeOT	(608-07-1)
114	5-MeO-TMT	(67292-68-6)

115	6-Acetylcodeine	(6703-27-1)
116	6-APB	(286834-84-2)
117	6-APDB	(152623-93-3)
118	6-Chlorothymolsulfonic acid	(83732-70-1)
119	6-EAPB	(1632539-47-9)
120	6-Mercaptopurine	(50-44-2)
121	6-O-Monoacetylmorphine (MAM)	(2784-73-8)
122	7-Aminoclonazepam	(4959-17-5)
123	7-Aminodesmethylflunitrazepam	(894-76-8)
124	7-Aminoflunitrazepam	(34084-50-9)
125	7-Aminonitrazepam	(4928-02-3)
126	7-Hydroxymitragynine	(174418-82-7)
127	A-796.260	(895155-26-7)
128	A-834.735	(895155-57-4)
129	AB-005	(895155-25-6)
130	AB-CHMINACA	(1185887-21-1)
131	AB-FUBINACA	(1185282-01-2)
132	AB-FUBINACA metabolite	(1877243-60-1)
133	ABICA-5F	(na)
134	AB-PINACA	(1445752-09-9)
135	AB-PINACA 5Cl	(1801552-02-2)
136	AB-PINACA 5F	(na)
137	AB-PINACA metabolite (amide hydrolysis)	(na)
138	AB-Pinaca N-(5-OH-PENTYL) metabolite	(na)
139	Acebutolol	(37517-30-9)
140	Aceclidine	(827-61-2)
141	Acemetacin	(53164-05-9)
142	Acepromazine	(61-00-7)
143	Aceprometazine	(13461-01-3)
144	Acetaminodantrolene	(41515-09-7)
145	Acetazolamide	(59-66-5)
146	Acetiamine	(28008-04-0)
147	Acetildenafil	(831217-01-7)
148	Acetyl-Fentanyl	(3258-84-2)
149	Acetylvardenafil	(na)
150	Aciclovir	(59277-89-3)
151	Aconitine	(302-27-2)
152	Acrivastine	(87848-99-5)
153	Acrylfentanyl	(82003-75-6)
154	Actinoquinol	(15301-40-3)
155	ADB-5F	(1838134-16-9)

156	ADB-CHMICA	(na)
157	ADB-Chminaca	(1185887-13-1)
158	ADB-FUBINACA	(1445583-51-6)
159	ADBICA	(1445583-48-1)
160	ADBICA-5F	(na)
161	ADB-PINACA	(1633766-73-0)
162	ADB-PINACA-5F	(na)
163	Adenine	(73-24-5)
164	Adenosine	(58-61-7)
165	Adinazolam	(37115-32-5)
166	Adrenalone	(99-45-6)
167	Agomelatine	(138112-76-2)
168	AH-7921	(55154-30-8)
169	Ajmaline	(4360-12-7)
170	AKB48 (APINACA)	(1345973-53-6)
171	AKB48 N-(5-hydroxypentyl) metabolite	(1778734-77-2)
172	AKB48-5F (5F-APINACA)	(1400742-13-3)
173	Alfentanyl	(71195-58-9)
174	Alimemazine. Trimeprazine	(84-96-8)
175	Aliskiren	(173334-57-1)
176	Alizapride	(59338-93-1)
177	Allylescaline	(39201-75-7)
178	Almitrine	(27469-53-0)
179	alpha-ET	(2235-90-7)
180	alpha-Hydroxy-Alprazolam	(37115-43-8)
181	Alpha-methylfentanyl	(79704-88-4)
182	ALPHA-PBP. alpha-Pyrrolidinobutiophenone	(13415-82-2)
183	alpha-PEP	(na)
184	alpha-PHP	(13415-86-6)
185	alpha-PMP	(na)
186	alpha-POP / PV9	(na)
187	alpha-PPP	(19134-50-0)
188	Alpha-PVP	(14530-33-7)
189	ALPHA-PVP metabolite	(1797986-63-0.CF3COOH)
190	alpha-PVT	(1400742-66-6)
191	alpha-Pyrrolidinobutiophenone	(13415-54-8)
192	Alprazolam	(28981-97-7)
193	Alprenolol	(13655-52-2)
194	Alprostadile. Prostaglandin E1	(745-65-3)
195	Altretamine	(645-05-6)
196	Alypin	(963-07-5)

197	AM 1241	(444912-48-5)
198	AM-1220	(137642-54-7)
199	AM-1220-Azepane	(1348081-04-8)
200	AM-1248	(335160-66-2)
201	AM-2201	(335161-24-5)
202	AM-2232	(335161-19-8)
203	AM-2233	(444912-75-8)
204	AM-694	(335161-03-0)
205	Amantadine	(768-94-5)
206	AMB	(na)
207	AMB-5F	(1801552-03-3)
208	Ambroxol	(18683-91-5)
209	Amfepramone	(134-80-5)
210	Amidephrine	(3354-67-4)
211	Amiloride	(2016-88-8)
212	Aminoglutethimide	(125-84-8)
213	Aminophenazone. Amidopyrin	(58-15-1)
214	Aminopromazine	(58-37-7)
215	Aminorex	(2207-50-3)
216	Aminotadalafil	(385769-84-6)
217	Amiodarone	(1951-25-3)
218	Amisulpiride	(71675-85-9)
219	Amitriptyline	(50-48-6)
220	Amitriptylinoxide	(4317-14-0)
221	Amlodipine	(88150-42-9)
222	Amobarbital	(57-43-2)
223	Amorolfine	(78613-35-1)
224	Amoxapine	(14028-44-5)
225	Amphetamine	(300-62-9)
226	Amrinone	(60719-84-8)
227	AMT . Alpha-Methyltryptamine	(299-26-3)
228	Anabasine	(40774-73-0)
229	Anatabine	(581-49-7)
230	Antazoline	(91-75-8)
231	APICA	(1345973-50-3)
232	Apixaban	(503612-47-3)
233	Apophedrin (Phenylethanolamine)	(7568-93-6)
234	Apraclonidin	(66711-21-5)
235	Aprindine	(37640-71-4)
236	Apronalide	(528-92-7)
237	Arecoline	(63-75-2)

238	Argatroban	(74863-84-6)
239	Aripiprazole	(129722-12-9)
240	Asenapine	(65576-45-6)
241	Astemizole	(68844-77-9)
242	Atenolol	(29122-68-7)
243	Atomoxetine	(83015-26-3)
244	Atorvastatin	(134523-00-5)
245	Atropine	(51-55-8)
246	Avanafil	(330784-47-9)
247	Axeen (Proxabarbal. Proxibarbital)	(2537-29-3)
248	Azacyclonol	(115-46-8)
249	Azapropazone	(13539-59-8)
250	Azatadine	(3964-81-6)
251	Azelastine	(58581-89-8)
252	Azithromycin	(83905-01-5)
253	Baclofen	(1134-47-0)
254	Bambuterol	(81732-65-2)
255	Bamifylline	(2016-63-9)
256	Bamipine	(4945-47-5)
257	Barbital	(57-44-3)
258	Barverin	(1639-79-8)
259	BB-22	(1400742-42-8)
260	BDB	(42542-07-4)
261	Beclamide	(501-68-8)
262	Befunolol	(39552-01-7)
263	Benactyzine	(302-40-9)
264	Bendamustine	(16506-27-7)
265	Bendroflumethiazide	(73-48-3)
266	Benodanil	(15310-01-7)
267	Benorilate	(5003-48-5)
268	Benperidol	(2062-84-2)
269	Benproperine	(2156-27-6)
270	Benzatropine	(86-13-5)
271	Benzedrone	(1225617-75-3)
272	Benzethonium	(121-54-0)
273	Benzocaine	(94-09-7)
274	Benzoctamine	(17243-39-9)
275	Benzododecinium	(139-07-1)
276	Benzoxonium	(19379-90-9)
277	Benzoylecgonine	(519-09-5)
278	Benzthiazide	(91-33-8)

279	Benzyl Sildenafil	(na)
280	Benzylpiperazine. BZP	(2759-28-6)
281	Betaxolol	(63659-18-7)
282	Bethanidine	(55-73-2)
283	Bezafibrate	(41859-67-0)
284	Bicalutamide	(90357-06-5)
285	Biotin	(58-85-5)
286	Biperidene	(514-65-8)
287	Bisacodyl	(603-50-9)
288	Bisoprolol	(66722-44-9)
289	Bitrex (Denatonium)	(3734-33-6.benzoate)
290	bk-2C-B	(807631-09-0)
291	bk-MDDMA	(109367-07-9)
292	Boldenone	(846-48-0)
293	Bornaprine	(20448-86-6)
294	Brallobarbital	(561-86-4)
295	Brodifacoum	(56073-10-0)
296	Bromadiolone	(28772-56-7)
297	Bromazepam	(1812-30-2)
298	Bromhexine	(3572-43-8)
299	Bromocriptine	(25614-03-3)
300	Bromodragonfly	(502759-67-3)
301	Bromperidol	(10457-90-6)
302	Brompheniramine	(86-22-6)
303	Bromural (Bromisoval)	(496-67-3)
304	Brotizolam	(57801-81-7)
305	Brucine	(357-57-3)
306	Bucetin	(1083-57-4)
307	Budipine	(57982-78-2)
308	Bufexamac	(2438-72-4)
309	Bufotenin	(487-93-4)
310	Bulbocapnine	(298-45-3)
311	Bumetanide	(28395-03-1)
312	Bunitrolol	(34915-68-9)
313	Buphedrone	(408332-79-6)
314	Bupivacaine	(38396-39-3)
315	Bupranolol	(14556-46-8)
316	Buprenorphine	(52485-79-7)
317	Bupropion	(34911-55-2)
318	Buspirone	(36505-84-7)
319	Butaperazine	(653-03-2)

320	Butizide	(2043-38-1)
321	Butorphanol	(42408-82-2)
322	Butoxycaine	(3772-43-8)
323	Butylone	(17762-90-2)
324	Butylscopolamine	(149-64-4.Br)
325	Butyryl Fentanyl	(1169-70-6)
326	CAF-3	(na)
327	Cafaminol	(30924-31-3)
328	Caffeine	(58-08-2)
329	Calteridol	(121915-83-1)
330	Camfetamine	(92499-19-9)
331	Candesartan	(139481-59-7)
332	Candesartan cilexetil	(145040-37-5)
333	Cannabidiol	(13956-29-1)
334	Cannabinol	(521-35-7)
335	Canrenone	(976-71-6)
336	Caproylresorcinol	(70807-24-8)
337	Capsaicin	(404-86-4)
338	Carazolol	(57775-29-8)
339	Carbachol	(51-83-2)
340	Carbamazepine	(298-46-4)
341	Carbamazepine-10.11-epoxid	(36507-30-9)
342	Carbinoxamine	(486-16-8)
343	Carbodenafil	(944241-52-5)
344	Carboxyibuprofen	(15935-54-3)
345	Carbutamide	(339-43-5)
346	Carbuterol	(34866-47-2)
347	Carfentanyl	(59708-52-0)
348	Carisoprodol	(78-44-4)
349	Carteolol	(51781-06-7)
350	Carticaine (Articaine)	(23964-58-1)
351	Carvedilol	(72956-09-3)
352	Cathine	(492-39-7)
353	Cathinone	(71031-15-7)
354	CC-2201	(na)
355	Celiprolol	(56980-93-9)
356	Cerivastatin	(145599-86-6)
357	Cetirizine	(83881-51-0)
358	Chloramphenicol	(56-75-7)
359	Chlorazanil	(500-42-5)
360	Chlorbenzoxamine	(522-18-9)

361	Chlorcyclizine	(82-93-9)
362	Chlordiazepoxid	(58-25-3)
363	Chlormezanone	(80-77-3)
364	Chlorodenafil	(1058653-74-9)
365	Chloropretadalafil	(na)
366	Chloropyramine	(59-32-5)
367	Chloroquine	(54-05-7)
368	Chlorphenethazine	(2095-24-1)
369	Chlorpheniramine	(132-22-9)
370	Chlorpromazine	(50-53-3)
371	Chlorpropamide	(94-20-2)
372	Chlorprotixene	(113-59-7)
373	Chlortalidone	(77-36-1)
374	Cilazapril	(88768-40-5)
375	Cimetidine	(51481-61-9)
376	Cinchocaine	(85-79-0)
377	Cinnamyldenafil	(na)
378	Cinnarizine	(298-57-7)
379	Cinoxacin	(28657-80-9)
380	Ciprofloxacin	(85721-33-1)
381	Cisapride	(81098-60-4)
382	Citalopram	(59729-33-8)
383	Clemastine	(15686-51-8)
384	Clenbuterol	(37148-27-9)
385	Clibucaine	(15302-10-0)
386	Clidinium (I+)	(7020-55-5)
387	Climbazole	(38083-17-9)
388	Clobazam	(22316-47-8)
389	Clobenzepam	(1159-93-9)
390	Clobenzorex	(13364-32-4)
391	Clobetasone butyrate	(25122-57-0)
392	Clobutinol	(14860-49-2)
393	Clomethiazole	(533-45-9)
394	Clomipramine	(303-49-1)
395	Clonazepam	(1622-61-3)
396	Clonazolam	(33887-02-4)
397	Clonidine	(4205-90-7)
398	Clopamide	(636-54-4)
399	Clopenthixol	(982-24-1)
400	Clopidogrel	(113665-84-2)
401	Clothiapine	(2058-52-8)

402	Clotiazepam	(33671-46-4)
403	Clozapine	(5786-21-0)
404	Clozapine N-oxide	(34233-69-7)
405	Cocaethylene	(529-38-4)
406	Cocaine	(50-36-2)
407	Codeine	(76-57-3)
408	Colchicine	(64-86-8)
409	Corticosterone	(50-22-6)
410	Cortisone	(53-06-5)
411	Cotinine	(486-56-6)
412	Coumatetralyl	(5836-29-3)
413	CP 47-497	(70434-82-1)
414	CP 47-497-C8	(70434-92-3)
415	CP 55-940	(83002-04-4)
416	Croconazole	(77175-51-0)
417	Cumyl-PINACA	(1400742-15-5)
418	Cyamemazine	(3546-03-0)
419	Cyclamic acid	(100-88-9)
420	Cyclizine	(82-92-8)
421	Cyclobenzaprine	(303-53-7)
422	Cyclopentolate	(512-15-2)
423	Cyclopentynafil	(na)
424	Cyclovalone	(579-23-7)
425	Cyproheptadine	(129-03-3)
426	D2PM (diphenylprolinol)	(22348-32-9)
427	D3-6-O-Acetylmorphine	(136765-25-8)
428	D3-Atropin	(51-54-7)
429	D3-Benzoylecgonine	(115732-68-8)
430	D3-Chlorpromazine	(136765-28-1)
431	D3-Cocaethylene	(136765-30-5)
432	D3-Cocaine	(65266-73-1)
433	D3-Codeine	(70420-71-2)
434	D3-Cotinine	(110952-70-0)
435	D3-Doxepin	(347840-07-7.HCl)
436	D3-Ecgoninemethylester	(136765-34-9)
437	D3-EDDP	(136765-23-6.HClO4)
438	D3-Hydromorphone	(136765-37-2)
439	D3-LSD	(136765-38-3)
440	D3-Morphine	(67293-88-3)
441	D3-Oxycodone	(160227-46-3)
442	D3-Trimipramine	(136765-54-3)

443	D4-Clonazepam	(170082-15-2)
444	D4-Haloperidol	(136765-35-0)
445	D4-Lorazepam	(84344-15-0)
446	D4-Midazolam	(na)
447	D4-N-Desmethylflunitrazepam	(1397234-19-3)
448	D4-Nicotine	(350818-69-8)
449	D5-Amphetamine	(136765-27-0)
450	D5-Diazepam	(65854-76-4)
451	D5-Fentanyl	(118357-29-2)
452	D5-MBDB	(362043-98-9)
453	D5-MDA	(136765-42-9)
454	D5-MDEA	(160227-43-0)
455	D5-MDMA	(136765-43-0)
456	D5-Nordiazepam	(65891-80-7)
457	D5-Oxazepam	(65854-78-6)
458	D5-Temazepam	(136765-51-0)
459	D6-Dihydrocodeine	(na)
460	D6-Fluoxetine	(na)
461	D6-Zolpidem	(959605-90-4)
462	D7-7-Aminoflunitrazepam	(879894-27-6)
463	D7-Flunitrazepam	(1286448-08-5)
464	D9-Heroin	(1338713-49-7)
465	D9-Methadone	(1435933-74-6)
466	Dabigatran etexilate	(211915-06-9)
467	DALT	(60676-77-9)
468	Dapiprazole	(72822-12-9)
469	Dapoxetine	(119356-77-3)
470	Deacetyldiltiazem	(42399-40-6)
471	Debrisoquine	(1131-64-2)
472	Deflazacort	(14484-47-0)
473	Delorazepam	(2894-67-9)
474	Demeclocycline	(127-33-3)
475	Demoxepam	(963-39-3)
476	Denaverine	(3579-62-2)
477	Desalkylflurazepam	(2886-65-9)
478	Deschloroetizolam	(40054-73-7)
479	Deschloroketamine	(4631-27-0)
480	Desethylhydroxychloroquine	(4298-15-1)
481	Desipramine	(50-47-5)
482	Desloratadine	(100643-71-8)
483	Desmethoxyyangonin	(15345-89-8)

484	Desmethyl carbodenafil	(147676-79-7)
485	Desmethyl-Chlordiazepoxide	(7722-15-8)
486	Desmethylclobazam	(22316-55-8)
487	Desmethyl-Mirtazapine	(61337-68-6)
488	Desmethylnortriptyline	(4444-42-2)
489	Desmethylpromazine	(2095-20-7)
490	Desmethylsertraline	(87857-41-8)
491	Desomorphine	(427-00-9)
492	Desoxycortone 21-(3-phenylpropionate)	(14007-50-2)
493	Desoxycortone enantate	(64-85-7)
494	DET	(7558-72-7)
495	Detajmium	(33774-52-6)
496	Dexamethasone	(50-02-2)
497	Dexfenfluramine	(3239-44-9)
498	Dexmedetomidine	(113775-47-6)
499	Dextromethorphan	(125-71-3)
500	Dextropropoxyphene	(469-62-5)
501	Dextrorphan. O-Desmethyldextromethorphan	(125-73-5)
502	Diaveridine	(5355-16-8)
503	Diazepam	(439-14-5)
504	Dibenzepin	(4498-32-2)
505	Dibutylone (bk-MMBDB)	(802286-83-5)
506	Diclazepam	(2894-68-0)
507	Diclofenac	(15307-86-5)
508	Dicycloverine	(77-19-0)
509	Didesmethyl Sibutramine	(84467-54-9)
510	Dienogest	(65928-58-7)
511	Diethazine	(60-91-3)
512	Diethylcarbamazine	(90-89-1)
513	Digoxin	(20830-75-5)
514	Dihydrocodeine	(125-28-0)
515	Dihydroergocristine	(17479-19-5)
516	Dihydrokavain	(587-63-3)
517	Dihydromethysticin	(19902-91-1)
518	Dihydromorphine	(1421-28-9)
519	Diltiazem	(42399-41-7)
520	Dimethocain	(94-15-5)
521	Dimethyl Acetildenafil	(1290041-88-1)
522	Dimethyl Sildenafil	(1416130-63-6)
523	Dimethylcathinone. Metamfepramone	(15351-09-4)
524	Dimetindene	(5636-83-9)

525	Dimetotiazine	(7456-24-8)
526	Dimetridazole	(551-92-8)
527	Dinortramadol	(na)
528	Dioxethedrin	(497-75-6)
529	Dioxohongdenafil	(na)
530	Diphenhydramine	(58-73-1)
531	Diphenidine	(36794-52-2)
532	Diphenoxylate	(915-30-0)
533	Diphenylpyraline	(147-20-6)
534	Diponium	(58875-33-5)
535	Diprophylline	(479-18-5)
536	DiPT	(14780-24-6)
537	Dipyridamole	(58-32-2)
538	Disopyramide	(3737-09-5)
539	Dithio-desmethyl-carbodenafil	(na)
540	Dixyrazine	(2470-73-7)
541	DL-4-methylamphetamine	(41632-56-8).HCl)
542	DMAA	(105-41-9)
543	DMT . Dimethyltryptamine	(61-50-7)
544	DOB	(64638-07-9)
545	Dobutamine	(34368-04-2)
546	DOC	(123431-31-2)
547	DOET	(22004-32-6)
548	DOM	(15588-95-1)
549	Domperidone	(57808-66-9)
550	Donepezil	(120014-06-4)
551	Dopexamine	(86197-47-9)
552	Dorzolamide	(120279-96-1)
553	Dosulepin	(113-53-1)
554	Doxapram	(309-29-5)
555	Doxepin	(1668-19-5)
556	Doxylamine	(469-21-6)
557	DPT	(61-52-9)
558	Drazoxolon	(5707-69-7)
559	Drofenine	(1679-76-1)
560	Droperidol	(548-73-2)
561	Dropropizine	(17692-31-8)
562	Duloxetine	(116539-59-4)
563	E-10-Hydroxyamitriptyline	(64520-05-4)
564	EAM-2201	(1364933-60-7)
565	Ebastine	(90729-43-4)

566	Ecgonine methyl ester	(7143-09-1)
567	EDDP	(30223-73-5)
568	Edoxaban	(480449-70-5)
569	EG-018	(na)
570	Embutramide	(15687-14-6)
571	Emepronium	(3614-30-0)
572	Enalapril	(75847-73-3)
573	Enoximon	(77671-31-9)
574	Entacapone	(130929-57-6)
575	Ephedrine	(299-42-3)
576	Eprosartan	(133040-01-4)
577	Ergotamine	(113-15-5)
578	Erythromycin	(114-07-8)
579	Esculin	(531-75-9)
580	Esmolol	(103598-03-4)
581	Esomeprazole	(119141-88-7)
582	Estazolam	(29975-16-4)
583	Etaqualone	(7432-25-9)
584	EtG. Ethylglucuronide	(17685-04-0)
585	Ethambutol	(74-55-5)
586	Ethenzamide	(938-73-8)
587	Ethylcathinone	(51553-17-4)
588	Ethylmorphine	(76-58-4)
589	Ethylone	(1112937-64-0)
590	Ethylphenidate	(57413-43-1)
591	Etilefrine	(709-55-7)
592	Etizolam	(40054-69-1)
593	Etodroxizine	(17692-34-1)
594	EtS	(540-82-9)
595	Euthylone. bk-EBDB	(802855-66-9)
596	Exemestane	(107868-30-4)
597	FAB-144	(na)
598	Famotidine	(76824-35-6)
599	FDU-PB-22	(na)
600	Fedrilate	(23271-74-1)
601	Felodipine	(72509-76-3)
602	Fencamfamine	(2240-14-4)
603	Fendiline	(13042-18-7)
604	Fenethylline	(3736-08-1)
605	Fenfluramine	(458-24-2)
606	Fenofibrate	(49562-28-9)

607	Fenoterol	(13392-18-2)
608	Fenpipramide	(77-01-0)
609	Fenpiprane	(3329-14-4)
610	Fenproporex (NARL)	(15686-61-0)
611	Fentanyl	(437-38-7)
612	Fenticonazole	(72479-26-6)
613	Fexofenadine	(83799-24-0)
614	Flecainide	(54143-55-4)
615	Flibanserin	(167933-07-5)
616	Flocoumafen	(90035-08-8)
617	Floctafenine	(23779-99-9)
618	Fluanisone	(1480-19-9)
619	Flubromazepam	(2647-50-9)
620	Flubromazolam	(612526-40-6)
621	Fluconazole	(86386-73-4)
622	Fludrocortisone	(514-36-3)
623	Flumazenil	(78755-81-4)
624	Flunitrazepam	(1622-62-4)
625	Flunixin	(38677-85-9)
626	Fluoro methyl phenidate	(na)
627	Fluoxetine	(54910-89-3)
628	Flupentixol	(2709-56-0)
629	Fluphenazine	(69-23-8)
630	Flupirtine	(56995-20-1)
631	Flurazepam	(17617-23-1)
632	Fluspirilen	(1841-19-6)
633	Fluvastatin	(93957-54-1)
634	Fluvoxamine	(54739-18-3)
635	Fosinopril	(98048-97-6)
636	Frovatriptan	(158747-02-5)
637	FUB-144	(na)
638	FUB-AKB48	(na)
639	FUB-AMB	(na)
640	FUBIMINA	(na)
641	FUB-PB-22	(1800098-36-5)
642	Furanyl fentanyl	(101345-66-8)
643	Furazolidone	(67-45-8)
644	Gabapentin	(60142-96-3)
645	Galantamine	(357-70-0)
646	Gallopamil	(16662-47-8)
647	Gendenafil	(147676-66-2)

648	GHB	(591-81-1)
649	Glaphenine	(3820-67-5)
650	Glibenclamide	(10238-21-8)
651	Glibornuride	(26944-48-9)
652	Glimepiride	(93479-97-1)
653	Glipizide	(29094-61-9)
654	Granisetron	(109889-09-0)
655	Griseofulvin	(126-07-8)
656	Guaifenesin	(93-14-1)
657	Guanabenz	(5051-62-7)
658	Guanethidine	(55-65-2)
659	Guanoxan	(2165-19-7)
660	Halcinonide	(3093-35-4)
661	Haloperidol	(52-86-8)
662	Haloxyfop ethoxyethyl ester	(87237-48-7)
663	Harmaline	(304-21-2)
664	Harman	(486-84-0)
665	Harmine	(442-51-3)
666	Harpagoside	(19210-12-9)
667	HDMP-28. Methylnaphthidate	(231299-82-4)
668	Heroin	(561-27-3)
669	Hexobendine	(54-03-5)
670	HHMA	(15398-87-5)
671	Histamine	(51-45-6)
672	Histapyrrodine	(493-80-1)
673	Histidine	(71-00-1)
674	HMA	(na)
675	HMMA	(117652-28-5)
676	Homatropine	(87-00-3)
677	Homofenazine	(3833-99-6)
678	HomoSildenafil	(642928-07-2)
679	Hordenine	(539-15-1)
680	HTMPIPO-4	(1445751-38-1)
681	HU-210	(112830-95-2)
682	Hydrochlorothiazide	(58-93-5)
683	Hydrocodone	(125-29-1)
684	Hydrocortisone	(850-23-7)
685	Hydrocortisone 21-acetate	(50-03-3)
686	Hydromorphone	(466-99-9)
687	Hydroxy Acetildenafil	(147676-56-0)
688	Hydroxy Vardenafil	(224785-98-2)

689	Hydroxyalprazolam	(30896-57-2/4-OH or 37115-43-8/alpha-OH)
690	Hydroxybupropion (as (RS.RS)-cyclic Hemiketal)	(233600-52-7)
691	Hydroxychloroquine	(118-42-3)
692	Hydroxycotinine	(34834-67-8)
693	Hydroxyhomo Sildenafil	(139755-85-4)
694	Hydroxyquetiapine	(139079-39-3)
695	Hydroxythiohomo Sildenafil	(479073-82-0)
696	Hydroxyzine	(68-88-2)
697	Ibogaine	(83-74-9)
698	Ibuprofen	(15687-27-1)
699	Icariin	(489-32-7)
700	Imipramine	(50-49-7)
701	Indanazoline	(40507-78-6)
702	Indapamide	(26807-65-8)
703	Indinavir	(150378-17-9)
704	Indomethacin	(53-86-1)
705	Indoramin	(26844-12-2)
706	Iopodic acid	(5587-89-3)
707	Ipratropium	(22254-24-6)
708	Iprindol	(5560-72-5)
709	Irbesartan	(138402-11-6)
710	Isoaminile	(77-51-0)
711	Isoconazole	(24168-96-5)
712	Iso-LSD	(2126-78-5)
713	Isoniazide	(54-85-3)
714	Isopropylphenidate	(93148-46-0)
715	Isothipendyl	(482-15-5)
716	Isoxsuprine	(395-28-8)
717	Isradipine	(75695-93-1)
718	Ivabradine	(155974-00-8)
719	JWH-007	(155471-10-6)
720	JWH-015	(155471-08-2)
721	JWH-018	(209414-07-3)
722	JWH-018-1-Methyl-Hexyl	(155471-13-9)
723	JWH-018-6-Methoxy-Ind	(1427325-49-2)
724	JWH-018-Adamantoyl. AB-001	(1345973-49-0)
725	JWH-018-D9	(JWH-018. 209414-07-3)
726	JWH-018-M-2-OH-Ind	(1427325-32-3)
727	JWH-018-M-4-OH-Ind	(1307803-42-4)

728	JWH-018-M-5-OH-Ind	(1307803-43-5)
729	JWH-018-M-6-OH-Ind	(1307803-44-6)
730	JWH-018-M-7-OH-Ind	(1307803-45-7)
731	JWH-018-M-N-4-OH-Pentyl	(1320363-47-0)
732	JWH-018-M-N-5-OH-Pentyl	(335161-21-2)
733	JWH-018-M-N-Pentanoic acid	(1254475-87-0)
734	JWH-019	(209414-08-4)
735	JWH-019-(5-hydroxyindol) metabolite	(1379604-70-2)
736	JWH-020	(209414-09-5)
737	JWH-022	(209414-16-4)
738	JWH-073	(208987-48-8)
739	JWH-073-2-Methyl	(na)
740	JWH-073-3-Methyl	(na)
741	JWH-073-M-2-OH-Ind	(1427325-54-9)
742	JWH-073-M-3-OH-Butyl	(na)
743	JWH-073-M-4-OH-Butyl	(335161-14-3)
744	JWH-073-M-4-OH-Ind	(1307803-46-8)
745	JWH-073-M-6-OH-Ind	(1307803-48-0)
746	JWH-073-M-7-OH-Ind	(1307803-49-1)
747	JWH-073-M-N-Butanoic acid	(1307803-52-6)
748	JWH-073-N-(3-hydroxybutyl) metabolite-D5	(1413427-47-0)
749	JWH-081	(210179-46-7)
750	JWH-081-N-(5-hydroxypentyl) metabolite	(1427325-66-3)
751	JWH-122	(619294-47-2)
752	JWH-122-F-Pentyl	(1354631-24-5)
753	JWH-122-M-N-5-OH-Pentyl	(1379604-68-8)
754	JWH-147	(914458-20-1)
755	JWH-180	(824959-87-7)
756	JWH-200	(103610-04-4)
757	JWH-200-M-4-OH-Ind	(1427325-73-2)
758	JWH-201	(864445-47-6)
759	JWH-203	(864445-54-5)
760	JWH-210	(824959-81-1)
761	JWH-210-N-(4-hydroxypentyl) metabolite	(1427521-37-6)
762	JWH-250	(864445-43-2)
763	JWH-250-N-(4-hydroxypentyl) metabolite	(1427521-38-7)
764	JWH-251	(864445-39-6)
765	JWH-302	(864445-45-4)
766	JWH-307	(914458-26-7)
767	JWH-368	(914458-31-4)
768	JWH-370	(914458-22-3)

769	JWH-387	(1366067-59-5)
770	JWH-398	(1292765-18-4)
771	JWH-398-N-(5-hydroxypentyl) metabolite	(1379604-69-9)
772	JWH-412	(1364933-59-4)
773	JWH-412-5F. F2201	(1391485-39-4)
774	Kavain	(500-64-1)
775	Ketamine	(6740-88-1)
776	Ketazolam	(27223-35-4)
777	Ketobemidone	(469-79-4)
778	Ketoprofen	(22071-15-4)
779	Ketorolac	(74103-06-3)
780	Ketotifen	(34580-13-7)
781	Labetalol	(36894-69-6)
782	Lacosamide	(175481-36-4. 175481-37-5)
783	Lamotrigine	(84057-84-1)
784	Laudanosine	(2688-77-9)
785	Leflunomide	(75706-12-6)
786	Lercanidipine	(100427-26-7)
787	Letrozole	(112809-51-5)
788	Levamisole	(16595-80-5.HCl)
789	Levetiracetam	(102767-28-2)
790	Levobunolol	(47141-42-4)
791	Levocabastine	(79516-68-0)
792	Levodopa	(59-92-7)
793	Levomepromazine	(60-99-1)
794	Levomepromazinesulfoxide	(7052-08-6)
795	Levopropylhexedrine	(6192-97-8)
796	Lidocaine	(137-58-6)
797	Linagliptin	(668270-12-0)
798	Lisinopril	(83915-83-7)
799	Lisuride	(18016-80-3)
800	Lofepramine	(23047-25-8)
801	Lofexidine	(21498-08-8.HCl)
802	Lonazolac	(53808-88-1)
803	Loperamide	(53179-11-6)
804	Lopinavir	(192725-17-0)
805	Loprazolam	(61197-73-7)
806	Loratadine	(79794-75-5)
807	Lorazepam	(846-49-1)
808	Lormetazepam	(848-75-9)
809	Losartan	(114798-26-4)

810	Losartan Carboxylic Acid (EXP-3174)	(124750-92-1)
811	Lovastatin	(75330-75-5)
812	Loxapine	(1977-10-2)
813	LSD	(50-37-3)
814	Lurasidone	(367514-87-2)
815	M-144	(na)
816	MAB-CHMINACA	(na)
817	MA-CHMINACA	(na)
818	Malathion	(121-75-5)
819	MAM-2201	(1354631-24-5)
820	MAM2201 N-pentanoic acid metabolite	(1537889-09-0)
821	Maprotiline	(10262-69-8)
822	Mazindol	(22232-71-9)
823	MBDB	(103818-46-8)
824	m-CPP	(6640-24-0)
825	MDA	(4764-17-4)
826	MDAI	(132741-81-2)
827	MDAT	(101625-35-8)
828	MDDMA	(74698-50-3)
829	MDEA	(82801-81-8)
830	MDMA	(42542-10-9)
831	MDMB-CHMINACA	(1185888-32-7)
832	MDMB-FUBINACA	(na)
833	MDPBP	(24622-60-4)
834	MDPHP	(24622-61-5)
835	MDPPP	(24698-57-5)
836	MDPV	(687603-66-3)
837	Mebeverine	(3625-06-7)
838	Mebroqualone	(4260-20-2)
839	Meclofenamic acid	(644-62-2)
840	Meclonazepam	(58662-84-3)
841	Meclozine	(569-65-3)
842	Medazepam	(2898-12-6)
843	Mefenamic acid	(61-68-7)
844	Mefenorex	(17243-57-1)
845	Mefexamide	(1227-61-8)
846	Mefloquine	(53230-10-7)
847	Mefruside	(7195-27-9)
848	Meftetramine	(403860-66-2)
849	Melatonin	(73-31-4)
850	Melitracen	(5118-29-6)

851	Meloxicam	(71125-38-7)
852	Melperone	(3575-80-2)
853	Melphalan	(148-82-3)
854	Memantine	(19982-08-2)
855	Mephedrone	(1189805-46-6)
856	Mepindolol	(23694-81-7)
857	Mepirapim	(na)
858	Mepivacaine	(96-88-8)
859	Meprobamate	(57-53-4)
860	Meptazinol	(54340-58-8)
861	Mepyramine. Pyrilamine. Bromth	(91-84-9)
862	Mequitazine	(29216-28-2)
863	Mescaline	(54-04-6)
864	Mesoridazine	(5588-33-0)
865	Metaclazepam	(65517-27-3)
866	Metamizole (Dipyrone)	(68-89-3)
867	Metenolone acetate	(434-05-9)
868	Metformin	(657-24-9)
869	Methadone	(76-99-3)
870	Methamphetamine	(537-46-2)
871	Methanthelinium	(53-46-3)
872	Methaphenilene	(493-78-7)
873	Methaqualone	(72-44-6)
874	Methazolamide	(554-57-4)
875	Methcathinone	(5650-44-2)
876	Methedrone. 4MeOMC	(530-54-1)
877	Methfuroxam	(28730-17-8)
878	Methiopropamine	(7464-94-0)
879	Methocarbamol	(532-03-6)
880	Methohexital	(151-83-7)
881	Methoprotryne	(841-06-5)
882	Methotrexate	(59-05-2)
883	Methoxetamine. MXE	(1239943-76-0)
884	Methoxyphenamine	(93-30-1)
885	Methoxypiperamide	(na)
886	Methylephedrine	(552-79-4)
887	Methylergometrine	(113-42-8)
888	Methylone (MDMC)	(186028-79-5)
889	Methylphenidate	(113-45-1)
890	Methylscopolamine	(155-41-9)
891	Methylsynphrine	(na)

892	Methysergide	(361-37-5)
893	Methysticin	(495-85-2)
894	Metipranolol	(22664-55-7)
895	Metixene	(4969-02-2)
896	Metizolam (Desmethyletizolam)	(40054-68-0)
897	Metoclopramide	(364-62-5)
898	Metolazone	(17560-51-9)
899	Metoprolol	(37350-58-6)
900	Metronidazole	(443-48-1)
901	Metsulfuron-methyl	(74223-64-6)
902	Metyrapone	(54-36-4)
903	Mexedrone	(na)
904	Mexiletine	(31828-71-4)
905	Mianserine	(24219-97-4)
906	Mianserine-N-oxide	(62510-46-7)
907	Miconazole	(22916-47-8)
908	Midazolam	(59467-70-8)
909	Milnacipran	(92623-85-3)
910	Minocycline	(10118-90-8)
911	Minoxidil	(38304-91-5)
912	Mirodenafil	(862189-95-5)
913	Mirtazapine	(61337-67-5)
914	Mitragyna alkaloid	(na)
915	Mitragynine	(4098-40-2)
916	MIZOLASTINE	(108612-45-9)
917	MMB-2201 (AMB-PICA-5F)	(1616253-26-9)
918	MMB-CHMINACA. MDMB-CHMICA	(1971007-95-0)
919	MN-18	(1391484-80-2)
920	MN-25	(501926-82-5)
921	Moclobemide	(71320-77-9)
922	Modafinil	(68693-11-8)
923	Molindone	(7416-34-4)
924	Moperone	(1050-79-9)
925	Morphine	(57-27-2)
926	Morphine-3-beta-D-glucuronide	(20290-09-9)
927	Morphine-6-beta-D-glucuronide	(50444-03-6)
928	Moxaverine	(10539-19-2)
929	Moxisylyte	(54-32-0)
930	Moxonidine	(75438-57-2)
931	MPHP	(34138-58-4)
932	MPPP (3-desmethylprodine)	(13147-09-6)

933	MT-45	(57314-55-3.HCl)
934	N-(3-Methylbenzyl)piperazine	(5321-48-2)
935	N.N-Diethyl-m-toluamide. DEET	(134-62-3)
936	N.N-Diisobutyltryptamine	(15741-78-3)
937	Nabumetone	(42924-53-8)
938	Nadolol	(42200-33-9)
939	Naftidrofuryl	(31329-57-4)
940	Naftifine	(65472-88-0)
941	Nalbuphine	(20594-83-6)
942	Nalidixic acid	(389-08-2)
943	Nalmefene	(55096-26-9)
944	Nalorphine	(62-67-9)
945	Naloxone	(465-65-6)
946	Naltrexone	(16590-41-3)
947	Nandrolone	(434-22-0)
948	Nandrolone phenylpropionate	(62-90-8)
949	Naphazoline	(835-31-4)
950	Naphyrone	(850352-53-3)
951	Naproxen	(22204-53-1)
952	Nateglinide	(105816-04-4)
953	n-Butyl Tadalafil	(171596-31-9)
954	n-Desmethyl Sildenafil	(139755-82-1)
955	N-Desmethylflunitrazepam. Norflunitrazepam	(2558-30-7)
956	N-Desmethylolanzapine	(161696-76-0)
957	N-Desmethylpropafenone	(86383-21-3)
958	N-Desmethyltapentadol	(1300037-83-5)
959	Nebivolol	(99200-09-6)
960	NE-CHMIMO	(na)
961	Nefazodone	(83366-66-9)
962	Nefopam	(13669-70-0)
963	Neostigmine	(59-99-4)
964	N-Ethylamphetamine	(457-87-4)
965	N-Ethylbuphedrone	(1354631-28-9)
966	Nicardipine	(55985-32-5)
967	Nicotinamide	(98-92-0)
968	Nicotine	(54-11-5)
969	Nifedipine	(21829-25-4)
970	Nifenazone	(2139-47-1)
971	Niflumic acid	(4394-00-7)
972	Nifoxipam	(74723-10-7)
973	Nikethamide	(59-26-7)

974	Nilvadipine	(75530-68-6)
975	Nimetazepam	(2011-67-8)
976	Nimorazole	(6506-37-2)
977	Nisoldipine	(63675-72-9)
978	N-Isopropylsalicylamide	(551-35-9)
979	Nitrazepam	(146-22-5)
980	Nitrazolam	(28910-99-8)
981	Nizatidine	(76963-41-2)
982	NM-2201	(na)
983	NM-2-AI	(24445-44-1)
984	N-MBZP	(na)
985	N-Methyl Pregabalin	(1155843-61-0)
986	NMT	(61-49-4)
987	NNEI	(1338925-11-3)
988	NNEI-5Cl	(na)
989	NNEI-5F	(1445580-60-8)
990	Nomifensine	(24526-64-5)
991	NorAcetildenafil	(949091-38-7)
992	Norbuprenorphine	(78715-23-8)
993	Norchlorprothixene	(51382-91-3)
994	Norcitalopram	(144025-14-9)
995	Norclomipramine	(303-48-0)
996	Norclozapine	(6104-71-8)
997	Norcocaine	(18717-72-1)
998	Norcodeine	(467-15-2)
999	Nordextropropoxyphene	(3376-94-1)
1000	Nordiazepam	(1088-11-5)
1001	Nordiltiazem	(na)
1002	Nordoxepin	(1225-56-5)
1003	Norephedrine (Cathin. Phenylpropanolamine)	(14838-15-4)
1004	Norethisterone	(68-22-4)
1005	Norethisterone acetate	(51-98-9)
1006	Norfenefrine	(536-21-0)
1007	Norfentanyl	(1609-66-1)
1008	Norfloxacin	(70458-96-7)
1009	Norfluoxetine	(56161-73-0)
1010	Norketamine	(35211-10-0)
1011	Norlevomepromazine	(61733-92-4. HCl)
1012	Nor-LSD.Nor-iso-LSD	(35779-43-2 nor-LSD. Nor-iso-LSD na)
1013	Normephedrone	(31952-47-3)

1014	Normethadone	(467-85-6)
1015	Normianserine	(71936-92-0)
1016	Normorphine	(466-97-7)
1017	Norneosildenafil	(371959-09-0)
1018	Nororphenadrine. Tofenacin. Elamol	(15301-93-6)
1019	Noroxycodone	(57664-96-7)
1020	Norsibutramine	(na)
1021	Norsildenafil	(na)
1022	NorTadalafil	(171596-36-4)
1023	Nortetrazepam	(10379-11-0)
1024	Nortilidine	(38677-94-0)
1025	Nortramadol	(75377-45-6)
1026	Nortrimipramine	(2293-21-2)
1027	Nortriptyline	(72-69-5)
1028	Norvenlafaxine	(149289-30-5)
1029	Norverapamil	(67018-85-3)
1030	Noscapine	(128-62-1)
1031	NPB-22	(1445579-61-2)
1032	NPB-22-5F	(1445579-79-2)
1033	N-Propylamphetamine	(51799-32-7)
1034	Nuarimol	(63284-71-9)
1035	Obidoxime	(7683-36-5)
1036	Ocfentanil	(101343-69-5)
1037	oCpp (1-2 chloropiperazine)	(41202-32-8)(oCPP HCl)
1038	O-Desmethyldinortramadol	(na)
1039	O-Desmethylnortramadol	(na)
1040	O-Desmethylsulpiride	(67381-52-6)
1041	O-Desmethyltramadol	(73986-53-5)
1042	O-Desmethylvenlafaxine. Desvenlafaxine	(93413-62-8)
1043	Ofloxacin	(82419-36-1)
1044	Olanzapine	(132539-06-1)
1045	Olsalazine	(15772-48-2)
1046	Omeprazole	(73590-58-6)
1047	Ondansetron	(99614-02-5)
1048	Opipramol	(909-39-7)
1049	ORG 27569	(868273-06-7)
1050	Orlistat	(96829-58-2)
1051	Ornidazole	(16773-42-5)
1052	Orphenadrine	(83-98-7)
1053	Oseltamivir	(196618-13-0)
1054	Oxatomide	(60607-34-3)

1055	Oxazepam	(604-75-1)
1056	Oxcarbazepine	(28721-07-5)
1057	Oxeladin	(468-61-1)
1058	Oxetacaine	(126-27-2)
1059	Oxitropium	(30286-75-0)
1060	Oxohongdenafil	(na)
1061	Oxomemazine	(3689-50-7)
1062	Oxprenolol	(6452-71-7)
1063	Oxybuprocaine	(99-43-4)
1064	Oxybutynin	(5633-20-5)
1065	Oxycodone	(76-42-6)
1066	Oxyfedrine	(15687-41-9)
1067	Oxymetazoline	(1491-59-4)
1068	Oxymorphone	(76-41-5)
1069	Oxymorphone-D3	(145225-03-2)
1070	Oxypendyl	(17297-82-4)
1071	Oxypertine	(153-87-7)
1072	Oxyphencyclimine	(125-53-1)
1073	Oxytetracycline	(79-57-2)
1074	Paliperidone (9-OH-Risperidone)	(144598-75-4)
1075	Pantoprazole	(102625-70-7)
1076	Papaverine	(58-74-2)
1077	Paracetamol	(103-90-2)
1078	Para-fluorofentanyl	(90736-23-5)
1079	Paroxetine	(61869-08-7)
1080	Paynantheine (Mitragyna alkaloid)	(23496-41-5)
1081	PB-22	(1400742-17-7)
1082	PB-22 3-carboxyindole metabolite	(727421-73-0)
1083	PB-22 N-(5-hydroxypentyl) metabolite	(na)
1084	PCN-5F	(na)
1085	Pemoline	(2152-34-3)
1086	Penbutolol	(38363-40-5)
1087	Penfluridol	(26864-56-2)
1088	Pentazocine	(359-83-1)
1089	Pentedrone	(879722-57-3)
1090	Pentifylline	(1028-33-7)
1091	Pentobarbital	(76-74-4)
1092	Pentoxifylline	(6493-05-6)
1093	Pentoxyverine	(77-23-6)
1094	Pentylone	(698963-77-8)
1095	Perazine	(84-97-9)

1096	Pergolide	(66104-22-1)
1097	Periciazine	(2622-26-6)
1098	Perphenazine	(58-39-9)
1099	Pethidine	(57-42-1)
1100	p-FBF	(244195-31-1)
1101	Phenacetin	(62-44-2)
1102	Phenazepam	(51753-57-2)
1103	Phenazocine	(127-35-5)
1104	Phenazone	(60-80-0)
1105	Phenazopyridine	(94-78-0)
1106	Phencyclidine	(77-10-1)
1107	Phendimetrazine	(634-03-7)
1108	Phenformin	(114-86-3)
1109	Pheniramine	(86-21-5)
1110	Phenmetrazine	(134-49-6)
1111	Phenobarbital	(50-06-6)
1112	Phenolphthalein	(77-09-8)
1113	Phenprocoumon	(435-97-2)
1114	Phentermine	(122-09-8)
1115	Phentolamine	(50-60-2)
1116	Phenylbutazone	(50-33-9)
1117	Phenylephrine	(59-42-7)
1118	Phenylpiracetam	(77472-70-9)
1119	Phenyltoloxamine	(92-12-6)
1120	Phenytoin	(57-41-0)
1121	Pholcodine	(509-67-1)
1122	Pholedrine	(370-14-9)
1123	Phthalylsulfathiazole	(85-73-4)
1124	P-hydroxyamphetamine	(1518-86-1)
1125	P-Hydroxymesocarb	(72460-70-9)
1126	P-Hydroxymethamphetamine	(6114-26-7)
1127	Physostigmine	(57-47-6)
1128	Pilocarpine	(92-13-7)
1129	Pimozide	(2062-78-4)
1130	Pindolol	(13523-86-9)
1131	Pioglitazone	(111025-46-8)
1132	Pipamperone	(1893-33-0)
1133	Piperiacetildenafil	(147676-50-4)
1134	Pipotiazine	(39860-99-6)
1135	Pipradrol	(467-60-7)
1136	Piprozolin	(17243-64-0)

1137	Piracetam	(7491-74-9)
1138	Pirenzepine	(28797-61-7)
1139	Piretanide	(55837-27-9)
1140	Piritramide	(302-41-0)
1141	Pirmenol	(68252-19-7)
1142	Piroxicam	(36322-90-4)
1143	Pitofenone	(54063-52-4)
1144	Pizotifen	(15574-96-6)
1145	PMA	(64-13-1)
1146	PMMA	(3398-68-3)
1147	Practolol	(6673-35-4)
1148	Prajmaline	(35080-11-6)
1149	Pramipexole	(104632-26-0)
1150	Pravadoline. WIN-48-098	(92623-83-1)
1151	Prazepam	(2955-38-6)
1152	Prazosin	(19216-56-9)
1153	Prednisolone	(50-24-8)
1154	Prednisone	(53-03-2)
1155	Pregabalin	(148553-50-8)
1156	Prenylamine	(390-64-7)
1157	Prilocaine	(721-50-6)
1158	Primaquine	(90-34-6)
1159	Primidone	(125-33-7)
1160	Proadifen	(302-33-0)
1161	Procainamide	(51-06-9)
1162	Procaine	(59-46-1)
1163	Prochlorperazine	(58-38-8)
1164	Procyclidine	(77-37-2)
1165	Progesterone	(57-83-0)
1166	Proguanil	(500-92-5)
1167	Prolintane	(493-92-5)
1168	Promazine	(58-40-2)
1169	Promethazine	(60-87-7)
1170	Prometryn	(7287-19-6)
1171	Propafenone	(54063-53-5)
1172	Propantheline	(50-34-0. 298-50-0)
1173	Propiconazole	(60207-90-1)
1174	Propionylpromazine	(3568-24-9)
1175	Propipocaine	(3670-68-6)
1176	Propiverine	(60569-19-9)
1177	Propoxyphenyl sildenafil	(877777-10-1)

1178	Propoxyphenyl-Thiohydroxyhomosildenafil	(479073-90-0)
1179	Propranolol	(525-66-6)
1180	Propyphenazone	(479-92-5)
1181	Proquazone	(22760-18-5)
1182	Prothipendyl	(303-69-5)
1183	Protionamide	(14222-60-7)
1184	Protriptyline	(438-60-8)
1185	Proxyphylline	(603-00-9)
1186	Pseudoephedrine	(90-82-4)
1187	PseudoVardenafil	(224788-34-5)
1188	Psilocin	(520-53-6)
1189	PV8. alpha-PHPP	(13415-55-9 .HCl)
1190	PX-1	(na)
1191	PX-2	(na)
1192	Pyranocoumarin	(518-20-7)
1193	Pyrazolam	(39243-02-2)
1194	Pyribenzamine (Tripelenamine. Azaron)	(91-81-6)
1195	Pyridoxine	(65-23-6)
1196	Pyrimethamine	(58-14-0)
1197	Pyritinol	(1098-97-1)
1198	Pyrovalerone	(3563-49-3)
1199	Pyrvinium	(548-84-5)
1200	Quetiapine	(111974-69-7)
1201	Quinagolide	(87056-78-8)
1202	Quinapril	(85441-61-8)
1203	Quinidine	(56-54-2)
1204	Quinine	(130-95-0)
1205	Ramifenazone	(3615-24-5)
1206	Ramipril	(87333-19-5)
1207	Ranitidine	(66357-35-5)
1208	Rasagiline	(136236-51-6)
1209	Raubasine	(483-04-5)
1210	RCS-4	(1345966-78-0)
1211	RCS-4-M-5-COOH-Pentyl	(1427521-39-8)
1212	RCS-4-M-5-OH-Pentyl	(1379604-66-6)
1213	RCS-4-ortho	(1345966-76-8)
1214	RCS-8	(1345970-42-4)
1215	Reboxetine	(98769-81-4)
1216	Remifentanyl	(132875-61-7)
1217	Remoxipride	(117591-79-4)
1218	Repaglinide	(135062-02-1)

1219	Reproterol	(54063-54-6)
1220	Reserpine	(50-55-5)
1221	RH-34	(1028307-48-3)
1222	Riluzole	(1744-22-5)
1223	Rimonabant	(168273-06-1)
1224	Risperidone	(106266-06-2)
1225	Ritalinic acid	(19395-41-6)
1226	Ritodrine	(26652-09-5)
1227	Ritonavir	(155213-67-5)
1228	Rivaroxaban	(366789-02-8)
1229	Rivastigmine	(123441-03-2)
1230	Rizatriptan	(145202-66-0)
1231	Rocuronium	(119302-91-9)
1232	Ropinirole	(91374-21-9)
1233	Ropivacaine	(84057-95-4)
1234	Rosiglitazone	(122320-73-4)
1235	Rosuvastatin	(287714-41-4)
1236	Salbutamol	(18559-94-9)
1237	Salicylamide	(65-45-2)
1238	Salmeterol	(89365-50-4)
1239	Salvinorin A	(83729-01-5)
1240	Scopolamine	(51-34-3)
1241	SDB-005	(na)
1242	SDB-005-5F	(na)
1243	SDB-006	(695213-59-3)
1244	SDB-006 N-phenyl	(1430634-87-9)
1245	SDB-006-5F	(na)
1246	Sebuthylazine	(7286-69-3)
1247	Secbutabarbital	(125-40-6)
1248	Secobarbital	(76-73-3)
1249	Selegiline	(14611-51-9)
1250	Sertindole	(106516-24-9)
1251	Sertraline	(79617-96-2)
1252	Sibutramine	(106650-56-0)
1253	Sildenafil	(139755-83-2)
1254	Simvastatin	(79902-63-9)
1255	Sitagliptin	(486460-32-6)
1256	Solifenacin	(242478-37-1)
1257	Sotalol	(3930-20-9)
1258	Speciociliatine (Mitragyna alkaloid)	(14382-79-7)
1259	Speciogynine (Mitragyna alkaloid)	(4697-67-0)

1260	Spirapril	(83647-97-6)
1261	Stanozolol	(10418-03-8)
1262	Strychnine	(57-24-9)
1263	STS-135	(1354631-26-7)
1264	Sufentanil	(56030-54-7)
1265	Sulfabenzamide	(127-71-9)
1266	Sulfaclomide	(4015-18-3)
1267	Sulfadiazine	(68-35-9)
1268	Sulfadoxine	(2447-57-6)
1269	Sulfaethidole	(94-19-9)
1270	Sulfaguanidine	(57-67-0)
1271	Sulfalene	(152-47-6)
1272	Sulfamerazine	(127-79-7)
1273	Sulfamethazine	(57-68-1)
1274	Sulfamethizole	(144-82-1)
1275	Sulfamethoxazole	(723-46-6)
1276	Sulfamethoxypyridazine	(80-35-3)
1277	Sulfapyridine	(144-83-2)
1278	Sulfaquinoxaline	(59-40-5)
1279	Sulfasalazine	(599-79-1)
1280	Sulfathiazole	(72-14-0)
1281	Sulfinpyrazone	(57-96-5)
1282	Sulindac	(38194-50-2)
1283	Sulpiride	(15676-16-1)
1284	Sulthiame	(61-56-3)
1285	Sumatriptan	(103628-46-2)
1286	Suxibuzone	(27470-51-5)
1287	Synephrine	(94-07-5)
1288	Tacrine	(321-64-2)
1289	Tadalafil	(171596-29-5)
1290	Talinolol	(57460-41-0)
1291	Tamoxifen	(10540-29-1)
1292	Tapentadol	(175591-23-8)
1293	Telithromycin	(191114-48-4)
1294	Telmisartan	(144701-48-4)
1295	Temazepam	(846-50-4)
1296	Terazosin	(63590-64-7)
1297	Terbinafine	(91161-71-6)
1298	Terbutaline	(23031-25-6)
1299	Terconazole	(67915-31-5)
1300	Terfenadine	(50679-08-8)

1301	Terodiline	(15793-40-5)
1302	Tertatolol	(34784-64-0)
1303	Testosterone benzoate	(2088-71-3)
1304	Tetracaine	(94-24-6)
1305	Tetracycline	(60-54-8)
1306	Tetrazepam	(10379-14-3)
1307	Tetroxoprim	(53808-87-0)
1308	Tetryzoline	(84-22-0)
1309	TFMPP	(15532-75-9)
1310	THC	(1972-08-3)
1311	THC-COOH. 11-COOH-THC. 11-nor-9-Carboxy-THC	(56354-06-4)
1312	THC-OH. 11-OH-THC	(36557-05-8)
1313	Thebacon	(466-90-0)
1314	Thebaine	(115-37-7)
1315	Theobromine	(83-67-0)
1316	Theophylline	(58-55-9)
1317	Thiethylperazine	(1420-55-9)
1318	Thiodimethyl Sildenafil	(856190-47-1)
1319	Thioguanine	(154-42-7)
1320	Thiohomo Sildenafil	(479073-80-8)
1321	Thiopental	(76-75-5)
1322	Thiopropazate	(84-06-0)
1323	Thioproperazine	(316-81-4)
1324	Thioridazine	(50-52-2)
1325	Thioridazine-5-sulfoxide	(7776-05-8)
1326	Thiosildenafil	(479073-79-5)
1327	Thiothinone. bk-MPA	(24065-17-6)
1328	Thiothixene	(5591-45-7)
1329	THJ	(na)
1330	THJ-018	(1364933-55-0)
1331	THJ-2201	(1801552-01-1)
1332	THJ-5F	(na)
1333	TH-PVP	(na)
1334	Thymopentin	(69558-55-0)
1335	Tiagabine	(115103-54-3)
1336	Tiapride	(51012-32-9)
1337	Ticagrelor	(274693-27-5)
1338	Ticlopidine	(55142-85-3)
1339	Tiemonium	(144-12-7)
1340	Tiletamine	(14176-49-9)

1341	Tilidine	(51931-66-9)
1342	Timolol	(26839-75-8)
1343	Tinidazole	(19387-91-8)
1344	Tiocarlide	(910-86-1)
1345	Tizanidine	(51322-75-9)
1346	TMA	(1082-23-1)
1347	Tolazamide	(1156-19-0)
1348	Tolazoline	(59-98-3)
1349	Tolbutamide	(64-77-7)
1350	Toliprolol	(2933-94-0)
1351	Tolmetin	(26171-23-3)
1352	Tolnaftate	(2398-96-1)
1353	Tolpropamine	(5632-44-0)
1354	Tolycaine	(3686-58-6)
1355	Topiramate	(97240-79-4)
1356	Topotecan	(123948-87-8)
1357	Torasemide	(56211-40-6)
1358	Toremifene	(89778-26-7)
1359	Tramadol	(27203-92-5)
1360	Trandolapril	(87679-37-6)
1361	Tranexamic acid	(1197-18-8)
1362	Trans-3-methylfentanyl	(na)
1363	Tranylcypromine	(155-09-9)
1364	Trapidil	(15421-84-8)
1365	Trazodone	(19794-93-5)
1366	Trenbolone	(10161-33-8)
1367	Triamcinolone	(124-94-7)
1368	Triamterene	(396-01-0)
1369	Triasulfuron	(82097-50-5)
1370	Triazolam	(28911-01-5)
1371	Trifluoperazine	(117-89-5)
1372	Trifluperidol	(749-13-3)
1373	Triflupromazine	(146-54-3)
1374	Trihexyphenidyl. Benzhexol	(144-11-6)
1375	Trimethobenzamide	(138-56-7)
1376	Trimethoprim	(738-70-5)
1377	Trimipramine	(739-71-9)
1378	Triperiden	(14617-17-5)
1379	Triprolidine	(486-12-4)
1380	Tritoqualine	(14504-73-5)
1381	Tromantadine	(53783-83-8)

1382	Tropatepine	(27574-24-9)
1383	Tropicamide	(1508-75-4)
1384	Tropisetron	(89565-68-4)
1385	Trospium	(10405-02-4)
1386	Tryptamine	(61-54-1)
1387	Tulobuterol	(41570-61-0)
1388	Tyramine	(51-67-2)
1389	U-47700	(82657-23-6)
1390	Udenafil	(268203-93-6)
1391	UR-144	(1199943-44-6)
1392	UR-144 Isomer	(na)
1393	UR-144 N-pentanoic acid metabolite	(1451369-33-7)
1394	UR-144-N-(4-hydroxypentyl)	(1537889-04-5)
1395	Valacyclovir	(124832-26-4)
1396	Valdecoxib	(181695-72-7)
1397	Valsartan	(137862-53-4)
1398	Vardenafil	(224785-90-4)
1399	Varenicline	(249296-44-4. 375815-87-5)
1400	Venlafaxine	(93413-69-5)
1401	Verapamil	(52-53-9)
1402	Vigabatrin	(60643-86-9)
1403	Vildagliptin	(274901-16-59
1404	Viloxazine	(46817-91-8)
1405	Vincamine	(1617-90-9)
1406	Vortioxetine	(508233-74-7)
1407	Warfarin	(81-81-2)
1408	WIN-55-212-2	(131543-22-1)
1409	Xanthinol	(2530-97-4)
1410	Xanthoanthrafil. Benzamidenafil	(1020251-53-9)
1411	Xipamide	(14293-44-8)
1412	XLR-11	(1364933-54-9)
1413	XLR-11 Isomer	(na)
1414	XLR-12	(895155-78-9)
1415	Xylometazoline	(526-36-3)
1416	Yangonin	(500-62-9)
1417	Yohimbine	(146-48-5)
1418	Zaleplon	(151319-34-5)
1419	Ziprasidone	(146939-27-7)
1420	Zolmitriptan	(139264-17-8)
1421	Zolpidem	(82626-48-0)
1422	Zonisamide	(68291-97-4)

1423	Zopiclone	(43200-80-2)
1424	Zotepine	(26615-21-4)
1425	Zuclopenthixol	(53772-83-1)
1426	1-(2-Methoxyphenyl)piperazine	
1427	1-(4-methoxyphenyl)piperazine	
1428	25I-NBF	
1429	2-Aminoindane. 2-AI	
1430	2C-B-FLY	
1431	2C-D	
1432	2-DPMP (desoxypipradrole)	
1433	2-Fluoroethcathinone. 2-FEC	
1434	2-fluoromethcathinone	
1435	2-MEC. 2-Methylethcathinone	
1436	2-MeO-Diphenidine. Methoxphenidine	
1437	2-MMC. 2-Methylmethcathinone	
1438	3.4.5-Trimethoxyamphetamine. TMA	
1439	3.4-Dimethoxyphenethylamine	
1440	3.4-Dimethylethcathinone	
1441	3.4-Methylenedioxy-n-benzylcathinone	
1442	3-4-CTMP	
1443	3-Ethylethcathinone	
1444	3-Fluorophenmetrazine	
1445	3-MEC. 3-Methylethcathinone	
1446	3-MeO-PCP	
1447	3-MMC. 3-Methylmethcathinone	
1448	4-Acetoxy-N-methyl-N-ethyltryptamine	
1449	4-AcO-DMT. Psilacetin	
1450	4-BMC. Brephedrone	
1451	4-CEC	
1452	4-CMC. Clephedrone	
1453	4-Cyano CUMYL-BUTINACA	
1454	4-EMC. 4-Ethylmethcathinone	
1455	4-Ethylethcathinone	
1456	4-Ethylmethcathinone	
1457	4-Ethyl-N.N-Dimethylcathinone	
1458	4F-alpha-PEP. 4F-PV8	
1459	4F-alpha-PVP	
1460	4-Fluoroethcathinone	
1461	4-MeO-PCP	
1462	4-MeTMP. 4-Methylmethylphenidate	
1463	4-MMA (Methylmethamphetamine)	

1464	4-OH MET. Metocin
1465	5-APDB
1466	5-EAPB
1467	5F-AMBICA
1468	5-Fluoro AKB48 N-(4-hydroxypentyl) metabolite
1469	5-Fluoro PB-22 3-carboxyindole metabolite
1470	5-fluoro PY-PICA
1471	5F-PB-22. 5-fluoro QUPIC
1472	5-IT. 5-API
1473	5-MAPB
1474	5-MeO-MIPT
1475	5-MeOPP
1476	6-APDB
1477	6-EAPB
1478	AB-FUBINACA metabolite
1479	AB-PINACA metabolite (amide hydrolysis)
1480	AB-Pinaca N-(5-OH-PENTYL) metabolite
1481	Acetyl-Fentanyl
1482	Acrylfentanyl
1483	ADB-Chminaca
1484	AH-7921
1485	AKB48 N-(5-hydroxypentyl) metabolite
1486	Allylescaline
1487	ALPHA-PBP. alpha-Pyrrolidinobutiophenone
1488	alpha-PEP
1489	alpha-PHP
1490	alpha-PMP
1491	alpha-POP / PV9
1492	ALPHA-PVP metabolite
1493	alpha-PVT
1494	alpha-Pyrrolidinobutiophenone
1495	Alprostadile. Prostaglandin E1
1496	AM 1241
1497	Amitriptylinoxide
1498	Apixaban
1499	Argatroban
1500	Bendamustine
1501	Benodanil
1502	Benzedrone
1503	Bitrex (Denatonium)

1504	bk-2C-B
1505	Bromocriptine
1506	Butaperazine
1507	Butorphanol
1508	Butylscopolamine
1509	Butyryl Fentanyl
1510	Camfetamine
1511	Candesartan
1512	Carfentanyl
1513	Chloramphenicol
1514	Clozapine N-oxide
1515	Coumatetralyl
1516	D2PM (diphenylprolinol)
1517	D3-6-O-Acetylmorphine
1518	D3-Atropin
1519	D3-Benzoylecgonine
1520	D3-Chlorpromazine
1521	D3-Cocaethylene
1522	D3-Cocaine
1523	D3-Codeine
1524	D3-Cotinine
1525	D3-Doxepin
1526	D3-Ecgoninemethylester
1527	D3-EDDP
1528	D3-Hydromorphone
1529	D3-LSD
1530	D3-Morphine
1531	D3-Oxycodone
1532	D3-Trimipramine
1533	D4-Clonazepam
1534	D4-Haloperidol
1535	D4-Lorazepam
1536	D4-Midazolam
1537	D4-N-Desmethylflunitrazepam
1538	D4-Nicotine
1539	D5-Amphetamine
1540	D5-Diazepam
1541	D5-Fentanyl
1542	D5-MBDB
1543	D5-MDA
1544	D5-MDEA

1545	D5-MDMA
1546	D5-Nordiazepam
1547	D5-Oxazepam
1548	D5-Temazepam
1549	D6-Dihydrocodeine
1550	D6-Fluoxetine
1551	D6-Zolpidem
1552	D7-7-Aminoflunitrazepam
1553	D7-Flunitrazepam
1554	D9-Heroin
1555	D9-Methadone
1556	DALT
1557	Deschloroketamine
1558	Desmethoxyyangonin
1559	Desmethyl carbodenafil
1560	Desmethylclobazam
1561	Desmethylnortriptyline
1562	Desmethylpromazine
1563	Dexmedetomidine
1564	Dextrorphan. O-Desmethyldextromethorphan
1565	Digoxin
1566	Dihydrokavain
1567	Dihydromethysticin
1568	Diphenidine
1569	DL-4-methylamphetamine
1570	Domperidone
1571	E-10-Hydroxyamitriptyline
1572	Edoxaban
1573	Emepronium
1574	Etaqualone
1575	Etilefrine
1576	Euthylone. bk-EBDB
1577	Fenoterol
1578	Fluoro methyl phenidate
1579	Frovatriptan
1580	Furanyl fentanyl
1581	Guanethidine
1582	HDMP-28. Methylnaphthidate
1583	Homofenazine
1584	Hydroxybupropion (as (RS.RS)-cyclic Hemiketal)

1585	Ibogaine
1586	Isopropylphenidate
1587	JWH-019-(5-hydroxyindol) metabolite
1588	JWH-073-N-(3-hydroxybutyl) metabolite-D5
1589	JWH-081-N-(5-hydroxypentyl) metabolite
1590	JWH-210-N-(4-hydroxypentyl) metabolite
1591	JWH-250-N-(4-hydroxypentyl) metabolite
1592	JWH-398-N-(5-hydroxypentyl) metabolite
1593	Leflunomide
1594	Levamisole
1595	Lofexidine
1596	Loperamide
1597	Losartan Carboxylic Acid (EXP-3174)
1598	Loxapine
1599	Lurasidone
1600	MAM2201 N-pentanoic acid metabolite
1601	MDPHP
1602	Mebroqualone
1603	Meftetramine
1604	Metamfepramone
1605	Methoxetamine. MXE
1606	Methoxypiperamide
1607	Methysticin
1608	Mexedrone
1609	Mitragynine
1610	MPHP
1611	MPPP (3-desmethylprodine)
1612	MT-45
1613	N-(3-Methylbenzyl)piperazine
1614	N.N-Diisobutyltryptamine
1615	Nalmefene
1616	N-Desmethylolanzapine
1617	Neostigmine
1618	N-Ethylbuphedrone
1619	Nimetazepam
1620	NM-2-AI
1621	N-Methyl Pregabalin
1622	NMT
1623	Norchlorprothixene
1624	Norcocaine
1625	Norfluoxetine

1626	Norketamine
1627	Normephedrone
1628	Norneosildenafil
1629	Ocfentanil
1630	oCpp (1-2 chloropiperazine)
1631	PB-22 3-carboxyindole metabolite
1632	PB-22 N-(5-hydroxypentyl) metabolite
1633	p-FBF
1634	Phenylpiracetam
1635	Piracetam
1636	Pravadoline
1637	Propoxyphenyl sildenafil
1638	Propoxyphenyl-Thiohydroxyhomosildenafil
1639	PV8. alpha-PHPP
1640	Rasagiline
1641	RH-34
1642	Salvinorin A
1643	Stanozolol
1644	Sulfamethazine
1645	Tetracycline
1646	Thebaine
1647	Thiothinone. bk-MPA
1648	TH-PVP
1649	Tiletamine
1650	Topiramate
1651	Tropatepine
1652	Tryptamine
1653	U-47700
1654	UR-144 N-pentanoic acid metabolite
1655	UR-144-N-(4-hydroxypentyl)
1656	Viloxazine
1657	Vortioxetine
1658	Xanthoanthrafil. Benzamidenafil
1659	Yangonin
