# Supplementary material for: Assessment of Xenoestrogens in Jordanian Water System: Activity and Identification
Source: Toxics. 2023 Jan 9;11(1):63. doi: 10.3390/toxics11010063 (PMC9866086; doi:10.3390/toxics11010063)
Supplement: Supplementary file 1 [file toxics-11-00063-s001.zip › Supplementary Material S2.pdf]

Supplementary Material S2

List of Potential veterinary drugs pollutants

	Name	CAS Number
1	2-Acetamido-5-nitrothiazole	(140-40-9)
2	2-Aminoflubendazole	(82050-13-3)
3	3-(Methylamino)propylamine	(6291-84-5)
4	3-Amino-2-oxazolidinone	(80-65-9)
5	4-Epitetracycline	(79-85-6)
6	5-Hydroxyflunixin	(75369-61-8)
7	5-Hydroxymebendazole	(60254-95-7)
8	Albendazole	(54965-21-8)
9	Albendazole oxide	(54029-12-8)
10	Albendazole sulfone	(75184-71-3)
11	Albendazole-2-aminosulfone	(80983-34-2)
12	Altrenogest	(850-52-2)
13	Amoxicillin	(26787-78-0)
14	Ampicillin	(69-53-4)
15	Amprolium	(121-25-5)
16	Arprinocid	(55779-18-5)
17	Azaperol	(2804-05-9)
18	Azaperone	(1649-18-9)
19	Baquiloprim	(102280-35-3)
20	Basic violet 3. Methyl violet	(548-62-9)
21	Betamethasone	(378-44-9)
22	Brombuterol	(41937-02-4)
23	Cabergoline	(81409-90-7)
24	Carazolol	(57775-29-8)
25	Carbadox	(6804-07-5)
26	Carprofen	(53716-49-7)
27	Cefalexin	(15686-71-2)
28	Cefalonium	(5575-21-3)
29	Cefazolin	(25953-19-9)
30	Cefquinome	(84957-30-2)
31	Ceftiofur	(80370-57-6)
32	Chlordimeform	(6164-98-3)
33	Chlormadinone acetate	(302-22-7)
34	Chlorpromazine	(50-53-3)
35	Chlortetracycline	(57-62-5)
36	Cimaterol	(54239-37-1)
37	Cinoxacin	(28657-80-9)
38	Ciprofloxacin	(85721-33-1)

39	Clazuril	(101831-36-1)
40	Clenbuterol	(37148-27-9)
41	Clindamycin	(18323-44-9)
42	Clorsulon	(60200-06-8)
43	Closantel	(57808-65-8)
44	Clostebol	(1093-58-9)
45	Danofloxacin	(112398-08-0)
46	Dapsone. 4.4-Diaminodiphenylsulfone	(80-08-0)
47	Decoquinate	(18507-89-6)
48	Demeclocycline	(127-33-3)
49	Desacetyl cephapirin	(38115-21-8)
50	Desfuroyl ceftiofur cystein disulfide	(158039-15-7)
51	Dexamethasone	(50-02-2)
52	Diaveridine	(5355-16-8)
53	Diclazuril	(101831-37-2)
54	Diclofenac	(15307-86-5)
55	Dicyclanil	(112636-83-6)
56	Diethylstilbestrol	(56-53-1)
57	Difloxacin	(98106-17-3)
58	Dimetridazole	(551-92-8)
59	Dimetridazole-2-hydroxy	(936-05-0)
60	Dinitolmide	(148-01-6)
61	Doramectin	(117704-25-3)
62	Doxycycline	(564-25-0)
63	Enoxacin	(74011-58-8)
64	Enrofloxacin	(93106-60-6)
65	erythro-Isoxsuprine	(579-56-6)
66	erythro-Isoxsuprine	(579-56-6.HCl)
67	Erythromycin A	(114-07-8)
68	Ethopabate	(59-06-3)
69	Fenbendazole	(43210-67-9)
70	Fenbendazole sulfone	(54029-20-8)
71	Fenoterol	(13392-18-2)
72	Firocoxib	(189954-96-9)
73	Fleroxacin	(79660-72-3)
74	Florfenicol	(73231-34-2)
75	Florfenicol amine	(76639-93-5)
76	Flubendazole	(31430-15-6)
77	Flugestone 17-acetate	(2529-45-5)
78	Flumequine	(42835-25-6)
79	Flunixin	(38677-85-9)
80	Fumagillin	(23110-15-8)
81	Furaltadone	(139-91-3)

82	Furazolidone	(67-45-8)
83	Gamithromycin	(145435-72-9)
84	Halofuginone	(55837-20-2)
85	Haloperidol	(52-86-8)
86	Imidocarb	(27885-92-3)
87	Ipronidazole	(14885-29-1)
88	Ipronidazole-hydroxy	(35175-14-5)
89	Ivermectin B1A	(70288-86-7 (70161-11-4 + 70209-81-3))
90	Josamycin	(16846-24-5)
91	Ketoprofen	(22071-15-4)
92	Ketotriclabendazole	(1201920-88-8)
93	Lasalocid	(25999-20-6. Na-salt)
94	Leucocrystal violet	(603-48-5)
95	Leucomalachite green	(129-73-7)
96	Leucomalachite green D6	(1173021-13-0)
97	Leucomycin A5	(18361-45-0)
98	Levamisole	(14769-73-4)
99	Lincomycin	(154-21-2)
100	Lomefloxacin	(98079-51-7)
101	Mabuterol	(56341-08-3)
102	Maduramicin	(79356-08-4)
103	Malachite green	(569-64-2)
104	Marbofloxacin	(115550-35-1)
105	Mebendazole	(31431-39-7)
106	Mebendazole-amine	(52329-60-9)
107	Medroxyprogesterone 17-acetate	(71-58-9)
108	Mefenamic acid	(61-68-7)
109	Melengestrol acetate	(2919-66-6)
110	Meloxicam	(71125-38-7)
111	Meloxicam D3	(942047-63-4)
112	Metacycline	(914-00-1)
113	Methylprednisolone	(83-43-2)
114	Metronidazole	(443-48-1)
115	Metronidazol-hydroxy	(4812-40-2)
116	Monensin	(17090-79-8)
117	Morantel	(20574-50-9)
118	Moxidectin	(113507-06-5)
119	Nalidixic acid	(389-08-2)
120	Narasin	(55134-13-9)
121	Neospiramycin I	(70253-62-2)
122	Nequinate	(13997-19-8)
123	Niclosamide	(50-65-7)
124	Nitrofurantoin	(67-20-9)

125	Nitrofurazone	(59-87-0)
126	Nitrovin. Difuranzone	(804-36-4)
127	Nitroxynil	(1689-89-0)
128	Norfloxacin	(70458-96-7)
129	Norfloxacin D5	(1015856-57-1)
130	Norgestimate	(35189-28-7)
131	Novobiocin	(303-81-1)
132	Ofloxacin	(82419-36-1)
133	Olaquindox	(23696-28-8)
134	Oleandomycin	(3922-90-5)
135	Orbifloxacin	(113617-63-3)
136	Ormetoprim	(6981-18-6)
137	Oxfendazole	(53716-50-0)
138	Oxibendazole	(20559-55-1)
139	Oxolinic acid	(14698-29-4)
140	Oxyclozanide	(2277-92-1)
141	Oxyphenbutazone	(129-20-4)
142	Phenylbutazone	(50-33-9)
143	Piperazine	(110-85-0)
144	Pirlimycin	(79548-73-5)
145	Ponazuril	(69004-04-2)
146	Prednisolone	(50-24-8)
147	Propionylpromazine	(3568-24-9)
148	Pyrimethamine	(58-14-0)
149	Ractopamine	(97825-25-7)
150	Rafoxanide	(22662-39-1)
151	Rifaximin	(80621-81-4)
152	Robenidine	(25875-51-8)
153	Ronidazole	(7681-76-7)
154	Roxithromycin	(80214-83-1)
155	Salbutamol	(18559-94-9)
156	Salicylic acid	(69-72-7)
157	Salinomycin	(53003-10-4)
158	Sparfloxacin	(110871-86-8)
159	Sulfabenzamide	(127-71-9)
160	Sulfacetamide	(144-80-9)
161	Sulfachloropyridazine	(80-32-0)
162	Sulfaclozine	(102-65-8)
163	Sulfadiazine	(68-35-9)
164	Sulfadimethoxine	(122-11-2)
165	Sulfadimidine. Sulfamethazine	(57-68-1)
166	Sulfadoxine	(2447-57-6)
167	Sulfaethoxypyridazine	(963-14-4)

168	Sulfaguanidine	(57-67-0)
169	Sulfamerazine	(127-79-7)
170	Sulfameter. Sulfamethoxydiazine	(651-06-9)
171	Sulfamethizole	(144-82-1)
172	Sulfamethoxazole	(723-46-6)
173	Sulfamethoxazole D4	(1020719-86-1)
174	Sulfamethoxypyridazine	(80-35-3)
175	Sulfamonomethoxine	(1220-83-3)
176	Sulfamoxole	(729-99-7)
177	Sulfanilamide	(63-74-1)
178	Sulfanitran	(122-16-7)
179	Sulfaphenazole	(526-08-9)
180	Sulfapyridine	(144-83-2)
181	Sulfaquinoxaline	(59-40-5)
182	Sulfathiazole	(72-14-0)
183	Sulfatroxazole	(23256-23-7)
184	Sulfisomidine	(515-64-0)
185	Sulfisoxazole. Sulfafurazole	(127-69-5)
186	Sulfisozole	(73247-57-1) (Na.79921-43-0)
187	Terbutaline	(23031-25-6)
188	Testosterone	(58-22-0)
189	Tetracycline	(60-54-8)
190	Thiamphenicol	(15318-45-3)
191	Tiamulin	(55297-95-5)
192	Tilmicosin	(108050-54-0)
193	Tolfenamic acid	(13710-19-5)
194	Toltrazuril	(69004-03-1)
195	Toltrazuril sulfoxide	(69004-15-5)
196	Triclabendazole	(68786-66-3)
197	Trimethoprim	(738-70-5)
198	Tylosin 3-acetate	(63409-10-9)
199	Tylosin A	(1401-69-0)
200	Tylosin B. Desmycosin	(11032-98-7)
201	Valnemulin	(101312-92-9)
202	Vedaprofen	(71109-09-6)
203	Virginiamycin S1. Staphylomycin S1	(23152-29-6)
204	Xylazine	(7361-61-7)
205	Zeranol	(26538-44-3)
206	Zilpaterol	(117827-79-9)
