# Supplementary material for: Assessment of Xenoestrogens in Jordanian Water System: Activity and Identification
Source: Toxics. 2023 Jan 9;11(1):63. doi: 10.3390/toxics11010063 (PMC9866086; doi:10.3390/toxics11010063)
Supplement: Supplementary file 1 [file toxics-11-00063-s001.zip › Supplementary Material S3.pdf]

Supplementary Material S3

List of Potential Insecticides, pesticides and herbicides pollutants

	Name	CAS Number
1	1-(4-isopropylphenyl)-urea	(56046-17-4)
2	1.2.3.6-Tetrahydrophthalimide (cis-)	(1469-48-3)
3	1-Naphthylaceticacid	(86-87-3)
4	2.3.4.6-Tetrachlorophenol	(58-90-2)
5	2.4 Dimethylphenyl-N-methylformamidine (metabolite Amitraz)	(60397-77-5)
6	2.4.6-Trichlorophenol	(88-06-2)
7	2-Aminobenzimidazole	(934-32-7)
8	2-Phenylphenol	(90-43-7)
9	3 5 6-Trichloro-2-pyridinol (TCPy)	(6515-38-4)
10	4-Chlor-2-methylphenol	(1570-64-5)
11	4-Methoxy-1.3-phenylenediamine	(615-05-4)
12	Acephate	(30560-19-1)
13	Acequinocyl	(57960-19-7)
14	Acetamiprid	(135410-20-7)
15	Acetochlor	(34256-82-1)
16	Acibenzolar-S-Methyl	(135158-54-2)
17	Acifluorfen	(50594-66-6)
18	Aclonifen	(74070-46-5)
19	AD-67	(71526-07-3)
20	Alachlor	(15972-60-8)
21	Alanycarb	(83130-01-2)
22	Albendazole	(54965-21-8)
23	Aldicarb	(116-06-3)
24	Aldicarb-sulfone (Aldoxycarb)	(1646-88-4)
25	Aldicarb-sulfoxide	(1646-87-3)
26	Allethrin	(584-79-2)
27	Allidochlor	(93-71-0)
28	Alloxydim	(55634-91-8)
29	Ametoctradin	(865318-97-4)
30	Ametryn	(834-12-8)
31	Amicarbazone	(129909-90-6)
32	Amidosulfuron	(120923-37-7)
33	Aminocarb (Metacil)	(2032-59-9)
34	Aminopyralid	(150114-71-9)
35	Amisulbrom	(348635-87-0)
36	Amitraz	(33089-61-1)

37	Amitrole	(61-82-5)
38	Ancymidol	(12771-68-5)
39	Anilazine (Zinochlor. Dyrene)	(101-05-3)
40	Anilofos	(64249-01-0)
41	Anthraquinone	(84-65-1)
42	Aramite	(140-57-8)
43	Asana (Esfenvalerate)	(66230-04-4)
44	Aspon	(3244-90-4)
45	Asulam	(3337-71-1)
46	Asulam metabolite Acetyl Sulfanilamid	(na)
47	Atraton	(1610-17-9)
48	Atrazine	(1912-24-9)
49	Atrazine 2-Hydroxy	(2163-68-0)
50	Atrazine D5	(163165-75-1)
51	Atrazine-desethyl	(6190-65-4)
52	Atrazine-desethyl D6	(na)
53	Atrazine-desisopropyl	(1007-28-9)
54	AvermectinB1b (Abamectin)	(71751-41-2)
55	Azaconazole	(60207-31-0)
56	Azadirachtin	(11141-17-6)
57	Azamethiphos	(35575-96-3)
58	Azimsulfuron	(120162-55-2)
59	Azinphos-ethyl	(2642-71-9)
60	Azinphos-methyl (Guthion)	(86-50-0)
61	Aziprotryne	(4658-28-0)
62	Azoxystrobin	(131860-33-8)
63	BAC 10	(965-32-2)
64	BAC 12	(139-07-1)
65	BAC 14	(139-08-2)
66	BAC 16	(122-18-9)
67	BAC 18	(122-19-0)
68	Barban (endo) (isomerA)	(101-27-9)
69	Beflubutamid	(113614-08-7)
70	Benalaxyl	(71626-11-4)
71	Benazolin	(3813-05-6)
72	Benazolin-ethyl ester	(25059-80-7)
73	Bendiocarb	(22781-23-3)
74	Benfuracarb	(82560-54-1)
75	Benfuresate	(68505-69-1)
76	Benodanil	(15310-01-7)
77	Benomyl	(17804-35-2)

78	Benoxacor	(98730-04-2)
79	Bensulfuron-methyl	(83055-99-6)
80	Bensulide	(741-58-2)
81	Bensultap	(17606-31-4)
82	Bentazone	(25057-89-0)
83	Benthiavalicarb-isopropyl	(177406-68-7)
84	Benzisothiazol-3(2H)-one 1-2-	(2634-33-5)
85	Benzofenap	(82692-44-2)
86	Benzoic acid 3-6-Dichloro-2-hydroxy- (dicamba metabolite)	(3401-80-7)
87	Benzovindiflupyr	(1072957-71-1)
88	Benzoximate	(29104-30-1)
89	Benzoylprop-ethyl	(22212-55-1)
90	Benzthiazuron	(1929-88-0)
91	Benzylaminopurine 6-	(1214-39-7)
92	Bifenazate	(149877-41-8)
93	Bifenox	(42576-02-3)
94	Bifenthrin	(82657-04-3)
95	Bioallethrin	(584-79-2)
96	Bioresmethrin	(28434-01-7)
97	Bispyribac	(125401-75-4)
98	Bitertanol	(55179-31-2)
99	Bixafen	(581809-46-3)
100	Boscalid	(188425-85-6)
101	Brodifacoum	(56073-10-0)
102	Bromacil	(314-40-9)
103	Bromadiolone	(28772-56-7)
104	Bromfenvinfos	(33399-00-7)
105	Bromobutide	(74712-19-9)
106	Bromophos (Bromophos-methyl)	(2104-96-3)
107	Bromophos-ethyl	(4824-78-6)
108	Bromoxynil	(1689-84-5)
109	Bromuconazole	(116255-48-2)
110	BTS 44596 (metabolite prochloraz)	(na)
111	Bupirimate	(41483-43-6)
112	Buprofezin	(953030-84-7)
113	Butachlor	(23184-66-9)
114	Butafenacil	(134605-64-4)
115	Butamifos	(36335-67-8)
116	Butocarboxim	(34681-10-2)
117	Butocarboxim-sulfoxid	(34681-24-8)

118	Butoxycarboxim	(34681-23-7)
119	Butralin	(33629-47-9)
120	Buturon	(3766-60-7)
121	Butylate	(2008-41-5)
122	Cadusafos	(95465-99-9)
123	Cafenstrole	(125306-83-4)
124	Cambendazole	(26097-80-3)
125	Captafol	(2425-06-1)
126	Captan	(133-06-2)
127	Carbamate Ethyl-N-(3-hydroxyphenyl)-	(7159-96-8)
128	Carbanilide	(102-07-8)
129	Carbanolate	(671-04-5)
130	Carbaryl	(63-25-2)
131	Carbendazim	(10605-21-7)
132	Carbetamide	(16118-49-3)
133	Carbofuran	(1563-66-2)
134	Carbofuran 3-keto-	(16709-30-1)
135	Carbofuran D3	(1007459-98-4)
136	Carbofuran-3-hydroxy	(16655-82-6)
137	Carbophenothion	(786-19-6)
138	Carbophenothion-Methyl	(953-17-3)
139	Carbosulfan	(55285-14-8)
140	Carboxin	(5234-68-4)
141	Carfentrazone-ethyl	(128639-02-1)
142	Carpropamid	(104030-54-8)
143	Carvone	(99-49-0)
144	Cetylpyridinium chloride	(6004-24-6)
145	CGA 321113 (Trifloxystrobin Metabolite)	(252913-85-2)
146	Chinomethionat	(2439-01-2)
147	Chlomethoxyfen	(32861-85-1)
148	Chloramben	(133-90-4)
149	Chlorantraniliprole	(500008-45-7)
150	Chlorbromuron	(13360-45-7)
151	Chlorbufam	(1967-16-4)
152	Chlordimeform	(6164-98-3)
153	Chlorfenapyr	(122453-73-0)
154	Chlorfenprop-methyl	(14437-17-3)
155	Chlorfenson.Ovex.ephirsulfonate.ovatron	(80-33-1)
156	Chlorfenvinphos	(470-90-6)
157	Chlorfluazuron	(71422-67-8)
158	Chloridazon-desphenyl-methyl	(17254-80-7)

159	Chloridazone	(1698-60-8)
160	Chlorimuronethyl	(90982-32-4)
161	Chlormequat	(7003-89-6)
162	Chlorothalonil-4-hydroxy	(na)
163	Chlorotoluron	(15545-48-9)
164	Chloroxuron (Chloroxifenidim)	(1982-47-4)
165	Chlorpropham	(101-21-3)
166	Chlorpyrifos O-analog	(5598-15-2)
167	Chlorpyriphos	(2921-88-2)
168	Chlorpyriphos-methyl	(5598-13-0)
169	Chlorsulfuron	(64902-72-3)
170	Chlorthal-dimethyl (DCPA. Dacthal)	(1861-32-1)
171	Chlorthiamid	(1918-13-4)
172	Chlorthion	(500-28-7)
173	Chlorthiophos	(60238-56-4)
174	Chlozolinate	(72391-46-9)
175	Chromafenozide	(143807-66-3)
176	Cinidon-ethyl	(142891-20-1)
177	Cinosulfuron	(94593-91-6)
178	Clethodim	(99129-21-2)
179	Climbazole	(38083-17-9)
180	Clodinafop acid	(114420-56-3)
181	Clodinafop-propargyl	(105512-06-9)
182	Clofentezine	(74115-24-5)
183	Clomazone (Command)	(81777-89-1)
184	Clomeprop	(84496-56-0)
185	Cloprop	(101-10-0)
186	Clopyralid	(1702-17-6)
187	Cloquintocet-1-methyl-hexyl ester	(99607-70-2)
188	Cloransulam-methyl	(147150-35-4)
189	Clothiandin	(210880-92-5)
190	Coumachlor	(81-82-3)
191	Coumafuryl	(117-52-2)
192	Coumaphos	(56-72-4)
193	Coumatetralyl	(5836-29-3)
194	Crimidine	(535-89-7)
195	Crotoxyphos	(7700-17-6)
196	Crufomate	(299-86-5)
197	Cumyluron	(99485-76-4)
198	Cyanazine	(21725-46-2)
199	Cyanofenphos	(13067-93-1)

200	Cyanophos	(2636-26-2)
201	Cyantraniliprole	(736994-63-1)
202	Cyazofamid	(120116-88-3)
203	Cyclanilide	(113136-77-9)
204	Cycloate	(1134-23-2)
205	Cycloheximide	(66-81-9)
206	Cycloprothrin	(63935-38-6)
207	Cyclosulfamuron	(136849-15-5)
208	Cycloxydim	(101205-02-1)
209	Cycluron	(2163-69-1)
210	Cyenopyrafen	(560121-52-0)
211	Cyflufenamid	(180409-60-3)
212	Cyflumetofen	(400882-07-7)
213	Cyfluthrin (Baythroid)	(68359-37-5)
214	Cyhalofop acid	(122008-78-0)
215	Cyhalofop butyl	(122008-85-9)
216	Cyhalothrin (lambda-)	(91465-08-6)
217	Cymiazole	(61676-87-7)
218	Cymoxanil	(57966-95-7)
219	Cypermethrin	(52315-07-8)
220	Cyphenothrin	(39515-40-7)
221	Cyprazin	(22936-86-3)
222	Cyproconazole	(94361-06-5)
223	Cyprodinil	(121552-61-2)
224	Cyromazine	(66215-27-8)
225	Cythioate	(115-93-5)
226	Daimuron (Dymron)	(42609-52-9)
227	Dalapon	(75-99-0)
228	Dazomet	(533-74-4)
229	DCPMU (1-(3.4-DICHLOROPHENYL)-3-METHYL UREA)(Monomethyldiuron)	(3567-62-2)
230	DCPU (1.3.4-dichlorophenyl-urea)	(2327-02-8)
231	DDA (2.2-bis(4-chlorophenyl)-acetic acid)	(83-05-6)
232	DDAC-C10	(7173-51-5)
233	DDAC-C12	(3401-74-9)
234	DEET (Diethyltoluamide)	(134-62-3)
235	Deltamethrin	(52918-63-5)
236	Demeton-S	(126-75-0)
237	Demeton-S-methyl	(919-86-8)
238	Demeton-S-methylsulfone	(17040-19-6)
239	Demeton-S-methylsulfoxid (Oxydemeton-methyl)	(301-12-2)

240	Desmedipham	(13684-56-5)
241	Desmetryn	(1014-69-3)
242	Diafenthiuron	(80060-09-9)
243	Diafenthiuron metabolite CGA 140408	(na)
244	Diafenthiuron metabolite CGA 177960	(na)
245	Dialifos	(10311-84-9)
246	Diallate	(2303-16-4)
247	Diazinon	(333-41-5)
248	Diazinon-O-analog	(962-58-3)
249	Dibromo-4-hydroxy-benzoic acid 3-5-	(3337-62-0)
250	Dibutylchlorendate	(1770-80-5)
251	Dicamba	(1918-00-9)
252	Dicamba-methyl	(6597-78-0)
253	Dicapthon	(2463-84-5)
254	Dichlofenthion	(97-17-6)
255	Dichlofluanid	(1085-98-9)
256	Dichlormid	(37764-25-3)
257	Dichlorobenzamide	(2008-58-4)
258	Dichlorophen	(97-23-4)
259	Dichlorvos	(62-73-7)
260	Diclobutrazol	(75736-33-3)
261	Diclomezine	(62865-36-5)
262	Dicloran	(99-30-9)
263	Diclosulam	(145701-21-9)
264	Dicofol	(115-32-2)
265	Dicrotophos	(141-66-2)
266	Diethofencarb	(87130-20-9)
267	Difenacoum	(56073-07-5)
268	Difenoconazole	(119446-68-3)
269	Difenoxuron	(14214-32-5)
270	Difenzoquat	(49866-87-7)
271	Difethialone	(104653-34-1)
272	Diflubenzuron	(35367-38-5)
273	Diflufenican	(83164-33-4)
274	Diflufenzopyr	(109293-97-2)
275	Dihydroquinoline 2-2-4-Trimethyl-1-2-	(147-47-7)
276	Dikegulac	(18467-77-1)
277	Dimefuron	(34205-21-5)
278	Dimepiperate	(61432-55-1)
279	Dimethachlor	(50563-36-5)

280	Dimethachlor metabolite CGA 369873	(na)
281	Dimethametryn	(22936-75-0)
282	Dimethenamid	(87674-68-8)
283	Dimethipin	(55290-64-7)
284	Dimethirimol	(5221-53-4)
285	Dimethoate	(60-51-5)
286	Dimethoate metabolite Desmethyl Dimethoate	(na)
287	Dimethoate metabolite Dimethoate carboxylic acid	(1113-01-5)
288	Dimethomorph	(110488-70-5)
289	Dimethylanilin (N.N-)	(121-69-7)
290	Dimethylphthalate	(131-11-3)
291	Dimethylvinphos	(2274-67-1)
292	Dimetilan	(644-64-4)
293	Dimoxystrobin	(149961-52-4)
294	Dinex (2-Cyclohexyl-4.6-dinitrophenol)	(131-89-5)
295	Diniconazole	(83657-24-3)
296	Dinitramine	(29091-05-2)
297	Dinobuton	(973-21-7)
298	Dinocap	(39300-45-3)
299	Dinocap-Meptyl Ester NH4-adduct	(131-72-6 / 6119-92-2)
300	Dinocap-Meptyl Phenol	(3687-22-7)
301	Dinoseb	(88-85-7)
302	Dinotefuran	(165252-70-0)
303	Dinoterb	(1420-07-1)
304	Dioxacarb	(6988-21-2)
305	Dioxathion	(78-34-2)
306	Diphacinone	(82-66-6)
307	Diphenamid	(957-51-7)
308	Diphenylamine	(122-39-4)
309	Diphenylamine N-Nitroso-	(86-30-6)
310	Dipropetryn	(4147-51-7)
311	Diquat	(2764-72-9)
312	Disulfiram	(97-77-8)
313	Disulfoton	(298-04-4)
314	Disulfoton-sulfone	(2497-06-5)
315	Disulfoton-sulfoxid	(2497-07-6)
316	Ditalimfos	(5131-24-8)
317	Dithianon	(3347-22-6)
318	Dithiopyr	(97886-45-8)
319	Diuron	(330-54-1)
320	Diuron D6	(1007536-67-5)

321	DMSA	(4710-17-2)
322	Dodemorph	(1593-77-7)
323	Dodine	(2439-10-3)
324	Doramectin	(117704-25-3)
325	Drazoxolon	(5707-69-7)
326	Edifenphos	(17109-49-8)
327	Emamectin	(119791-41-2)
328	Endosulfan	(115-29-7)
329	Endosulfan-sulfate	(1031-07-8)
330	Endothal	(145-73-3)
331	EPN	(2104-64-5)
332	Epoxiconazole	(133855-98-8)
333	Eprinomectin	(123997-26-2)
334	EPTC	(759-94-4)
335	Esprocarb	(85785-20-2)
336	Etaconazole	(60207-93-4)
337	Ethaboxam	(162650-77-3)
338	Ethametsulfuron-methyl	(97780-06-8)
339	Ethidimuron	(30043-49-3)
340	Ethiofencarb	(29973-13-5)
341	Ethiofencarb-sulfone	(53380-23-7)
342	Ethiofencarb-sulfoxide	(53380-22-6)
343	Ethiolate	(2941-55-1)
344	Ethion	(563-12-2)
345	Ethiprole	(181587-01-9)
346	Ethirimol	(23947-60-6)
347	Ethofumesate	(26225-79-6)
348	Ethoprophos. Ethoprop	(13194-48-4)
349	Ethoxy-1-2-3-4-tetra-hydro-2-2-4-trimethylchinolin 6-	(16489-90-0)
350	Ethoxyquin	(91-53-2)
351	Etobenzanid	(79540-50-4)
352	Etofenprox	(80844-07-1)
353	Etoxazole	(153233-91-1)
354	Etrimfos	(38260-54-7)
355	Famoxadone	(131807-57-3)
356	Famphur	(52-85-7)
357	Fenamidone	(161326-34-7)
358	Fenamiphos	(22224-92-6)
359	Fenamiphos - sulfone	(31972-44-8)
360	Fenamiphos sulfoxide	(31972-43-7)

361	Fenarimol	(60168-88-9)
362	Fenazaquin	(120928-09-8)
363	Fenbuconazole	(114369-43-6)
364	Fenbutatin Oxide	(13356-08-6)
365	Fenclofos.Ronnel	(299-84-3)
366	Fenfuram	(24691-80-3)
367	Fenhexamid	(126833-17-8)
368	Fenitrothion	(122-14-5)
369	Fenobucarb	(3766-81-2)
370	Fenothiocarb	(62850-32-2)
371	Fenoxanil	(115852-48-7)
372	Fenoxaprop-ethyl	(66441-23-4)
373	Fenoxycarb	(79127-80-3)
374	Fenpiclonil	(74738-17-3)
375	Fenpropathrin	(39515-41-8)
376	Fenpropidin	(67306-00-7)
377	Fenpropimorph	(67564-91-4)
378	Fenpropimorph Carboxylic Acid	(121098-45-1)
379	Fenpyrazamine	(473798-59-3)
380	Fenpyroximate	(134098-61-6)
381	Fenson	(80-38-6)
382	Fensulfothion	(115-90-2)
383	Fensulfothion-oxon	(6552-21-2)
384	Fensulfothion-sulfon	(14255-72-2)
385	Fenthion	(55-38-9)
386	Fenthion-oxon	(6552-12-1)
387	Fenthion-oxonsulfone	(14086-35-2)
388	Fenthion-oxonsulfoxide	(6552-13-2)
389	Fenthion-sulfon	(3761-42-0)
390	Fenthion-sulfoxide	(3761-41-9)
391	Fenuron	(101-42-8)
392	Fenvalerate	(51630-58-1)
393	Ferimzone	(89269-64-7)
394	Fipronil	(120068-37-3)
395	Fipronil-Desulfinyl	(205650-65-3)
396	Fipronil-sulfide	(120067-83-6)
397	Fipronilsulfone	(120068-36-2)
398	Flamprop	(58667-63-3)
399	Flamprop-isopropyl	(52756-22-6)
400	Flamprop-methyl	(52756-25-9)
401	Flazasulfuron	(104040-78-0)

402	Flocoumafene	(90035-08-8)
403	Flonicamid	(158062-67-0)
404	Flonicamid metabolite TFNA-AM	(158062-71-6)
405	Flonicamid metabolite TFNG	(207502-65-6)
406	Florasulam	(145701-23-1)
407	Fluacrypyrim	(229977-93-9)
408	Fluazifop-P-Butyl metabolite CGA 142110	(33252-63-0)
409	Fluazinam	(79622-59-6)
410	Fluazuron	(86811-58-7)
411	Flubendiamide	(272451-65-7)
412	Flubenzimine	(37893-02-0)
413	Flucarbazone	(145026-88-6)
414	Fluchloralin	(33245-39-5)
415	Flucycloxuron	(113036-88-7)
416	Flucythrinate	(70124-77-5)
417	Fludioxonil	(131341-86-1)
418	Flufenacet	(142459-58-3)
419	Flufenacet sulfonic acid (FOE 5043-sulfonic acid)	(947601-87-8)
420	Flufenacet thioglycolate sulfoxide (FOE 5043 thioglycolate sulfoxide)	(201668-33-9)
421	Flufenacet-oxalate (FOE 5043-oxalate)	(201668-31-7)
422	Flufenoxuron	(101463-69-8)
423	Flumequine	(42835-25-6)
424	Flumethrin	(69770-45-2)
425	Flumetsulam	(98967-40-9)
426	Flumiclorac-pentyl	(87546-18-7)
427	Flumioxazin	(103361-09-7)
428	Fluometuron	(2164-17-2)
429	Fluopicolide	(239110-15-7)
430	Fluopyram	(658066-35-4)
431	Fluoroglycofen-ethyl	(77501-90-7)
432	Fluotrimazole	(31251-03-3)
433	Fluoxastrobin	(361377-29-9)
434	Flupyradifurone	(951659-40-8)
435	Fluquinconazole	(136426-54-5)
436	Fluridone	(59756-60-4)
437	Flurochloridone	(61213-25-0)
438	Fluroxypyr	(69377-81-7)
439	Flurprimidol	(56425-91-3)
440	Flurtamone	(96525-23-4)
441	Flusilazole	(85509-19-9)

442	Flusulfamide	(106917-52-6)
443	Fluthiacet-methyl	(117337-19-6)
444	Flutolanil	(66332-96-5)
445	Flutriafol	(76674-21-0)
446	Fluvalinate (tau-)	(102851-06-9)
447	Fluxapyroxad	(907204-31-3)
448	Folpet	(133-07-3)
449	Fomesafen	(72178-02-0)
450	Fonofos	(944-22-9)
451	Foramsulfuron	(173159-57-4)
452	Forchlorfenuron	(68157-60-8)
453	Formetanate	(22259-30-9)
454	Formothion	(2540-82-1)
455	Fosthiazate	(98886-44-3)
456	Fuberidazole	(3878-19-1)
457	Furalaxyl	(57646-30-7)
458	Furametpyr	(123572-88-3)
459	Furathiocarb	(65907-30-4)
460	Furilazole	(121776-33-8)
461	Furmecyclox	(60568-05-0)
462	Gibberellic acid	(77-06-5)
463	Glufosinate	(51276-47-2)
464	Griseofulvin	(126-07-8)
465	Halfenprox	(111872-58-3)
466	Halofenozide	(112226-61-6)
467	Halosulfuron-methyl	(100784-20-1)
468	Heptenophos	(23560-59-0)
469	Hexaconazole	(79983-71-4)
470	Hexaflumuron	(86479-06-3)
471	Hexazinone	(51235-04-2)
472	Hexythiazox	(78587-05-0)
473	Homobrassinolide 22(S)-23(S)-	(80483-89-2)
474	Hydramethylnon	(67485-29-4)
475	Hymexazol	(10004-44-1)
476	Icaridin	(119515-38-7)
477	Imazalil	(35554-44-0)
478	Imazamethabenz-methyl	(81405-85-8)
479	Imazamox	(114311-32-9)
480	Imazapic	(104098-48-8)
481	Imazapyr	(81334-34-1)
482	Imazaquin	(81335-37-7)

483	Imazethapyr	(81335-77-5)
484	Imazosulfuron	(122548-33-8)
485	Imibenconazole	(86598-92-7)
486	Imidacloprid	(138261-41-3)
487	Imiprothrin	(72963-72-5)
488	Inabenfide	(82211-24-3)
489	Indanofan	(133220-30-1)
490	Indole-3-butyric acid	(133-32-4)
491	Indolyl-acetic-acid-ethyl-ester 3-	(778-82-5)
492	Indoxacarb	(173584-44-6)
493	Iodocarb	(55406-53-6)
494	Iodofenphos (Jodfenphos)	(18181-70-9)
495	Iodophenoxyacetic acid 4-	(1878-94-0)
496	Iodosulfuron-methyl	(144550-36-7)
497	Ioxynil	(1689-83-4)
498	Ipconazole	(125225-28-7)
499	Iprobenfos	(26087-47-8)
500	Iprodione	(36734-19-7)
501	Iprodione metabolite RP 30228	(na)
502	Iprovalicarb	(140923-17-7)
503	Isazophos	(42509-80-8)
504	Isocarbamid (Azolamide)	(30979-48-7)
505	Isocarbophos	(24353-61-5)
506	Isofenphos	(25311-71-1)
507	Isofenphos-methyl	(99675-03-3)
508	Isofenphos-Oxon	(31120-85-1)
509	Isomethiozin	(57052-04-7)
510	Isoprocarb	(2631-40-5)
511	Isopropalin	(33820-53-0)
512	Isoprothiolane	(50512-35-1)
513	Isoproturon	(34123-59-6)
514	Isoproturon D6	(217487-17-7)
515	Isopyrazam	(881685-58-1)
516	Isothiazolin-3-one 2-Methyl-4-	(2682-20-4)
517	Isothiazolin-3-one 5-Chloro-2-methyl-4-	(26172-55-4)
518	Isotianil	(224049-04-1)
519	Isoxaben	(82558-50-7)
520	Isoxadifen-ethyl	(163520-33-0)
521	Isoxaflutole	(141112-29-0)
522	Isoxathion	(18854-01-8)

523	Ivermectin	(70288-86-7 (70161-11-4 + 70209-81-3))
524	Karbutilate	(4849-32-5)
525	KIF-3535-M-31 (metabolite of Mepanipyrim)	()
526	Kinetin	(525-79-1)
527	Kresoxim-methyl	(143390-89-0)
528	Kresoxim-Methyl metabolite BF 490-1	(137169-29-0)
529	Lactofen	(77501-63-4)
530	Lenacil	(2164-08-1)
531	Leptophos	(21609-90-5)
532	Lethane 384	(112-56-1)
533	Linuron	(330-55-2)
534	Lufenuron	(103055-07-8)
535	Malaoxon	(1634-78-2)
536	Malathion	(121-75-5)
537	Maleic Hydrazide	(123-33-1)
538	Mandestrobin	(173662-97-0)
539	Mandipropamid	(374726-62-2)
540	MCPA-Butoxyethyl-ester	(19480-43-4)
541	Mecarbam	(2595-54-2)
542	Mefenacet	(73250-68-7)
543	Mefenpyr-diethyl	(135590-91-9)
544	Mefluidide	(53780-34-0)
545	Melamine	(108-78-1)
546	Mepanipyrim	(110235-47-7)
547	Mepanipyrim metabolite KIF-3535-M-31	(204571-52-8)
548	Mephosfolan	(950-10-7)
549	Mepiquat 4-Hydroxy (Piperidinium)	(na)
550	Mepronil	(55814-41-0)
551	Mercaptobenzothiazole	(149-30-4)
552	Merphos	(150-50-5)
553	Mesosulfuron-methyl	(208465-21-8)
554	Mesotrione	(104206-82-8)
555	Mesotrione metabolite AMBA	(393085-45-5)
556	Metaflumizone	(139968-49-3)
557	Metalaxyl	(57837-19-1)
558	Metalaxyl metabolite CGA 107955	(104390-55-8)
559	Metalaxyl metabolite CGA 108905	(na)
560	Metalaxyl metabolite CGA 67869	(66637-79-4)
561	Metalaxyl metabolite CGA 94689	(na)
562	Metamitron	(41394-05-2)

563	Metazachlor	(67129-08-2)
564	Metconazole	(125116-23-6)
565	Methabenzthiazuron	(18691-97-9)
566	Methacrifos	(62610-77-9)
567	Methamidophos	(10265-92-6)
568	Methfuroxam	(28730-17-8)
569	Methidathion	(950-37-8)
570	Methiocarb (Mercaptodimethur)	(2032-65-7)
571	Methiocarb-sulfone	(2179-25-1)
572	Methiocarb-sulfoxide	(2635-10-1)
573	Methomyl	(16752-77-5)
574	Methomyl D3	(1398109-07-3)
575	Methoprene	(40596-69-8)
576	Methoprotryne	(841-06-5)
577	Methothrin	(34388-29-9)
578	Methoxyfenozide	(161050-58-4)
579	Metobromuron	(3060-89-7)
580	Metolachlor	(51218-45-2)
581	Metolachlor D6	(1219803-97-0)
582	Metolcarb	(1129-41-5)
583	Metominostrobin	(133408-51-2)
584	Metosulam	(139528-85-1)
585	Metoxuron	(19937-59-8)
586	Metrafenone	(220899-03-6)
587	Metribuzin	(21087-64-9)
588	Metsulfuron-methyl	(74223-64-6)
589	Mevinphos	(26718-65-0)
590	Mexacarbate	(315-18-4)
591	MGK-264	(113-48-4)
592	Milbemectin A4	(51596-11-3)
593	Molinate	(2212-67-1)
594	Monalide	(7287-36-7)
595	Monocrotophos	(6923-22-4)
596	Monolinuron	(1746-81-2)
597	Monuron	(150-68-5)
598	Moxidectin	(113507-06-5)
599	Myclobutanil	(88671-89-0)
600	N.N-Dimethyl-N'-p-tolylsulphamide (DMST. metabolite tolylfluanid)	(66840-71-9)
601	N-2-4-Dimethylphenylformamide (DMF. Metabolite Amitraz)	(60397-77-5)

602	Naled	(300-76-5)
603	Naphthalene acetamide	(86-86-2)
604	Naphthoxyaceticacid (beta-)	(120-23-0)
605	Naproanilide	(52570-16-8)
606	Napropamide	(15299-99-7)
607	Naptalam (N-1-Naphthylphthalamicacid)	(132-66-1)
608	Neburon	(555-37-3)
609	Nicosulfuron	(111991-09-4)
610	Nicotine	(54-11-5)
611	Nitenpyram	(150824-47-8)
612	Nitralin	(4726-14-1)
613	Nitrofen	(1836-75-5)
614	Nitroguaiacol 5-	(na)
615	Nitrothal-isopropyl	(10552-74-6)
616	Norflurazon	(27314-13-2)
617	Norflurazon Desmethyl	(23576-24-1)
618	Novaluron	(116714-46-6)
619	Noviflumuron	(121451-02-3)
620	Nuarimol	(63284-71-9)
621	Octhilinone	(26530-20-1)
622	Ofurace	(58810-48-3)
623	Omethoate	(1113-02-6)
624	Orbencarb	(34622-58-7)
625	Orthosulfamuron	(213464-77-8)
626	Oryzalin	(19044-88-3)
627	Oxabetrinil	(94593-79-0)
628	Oxadiargyl	(39807-15-3)
629	Oxadiazon	(19666-30-9)
630	Oxadixyl	(77732-09-3)
631	Oxamyl	(23135-22-0)
632	Oxamyl oxime	(30558-43-1)
633	Oxasulfuron	(144651-06-9)
634	Oxaziclomefone	(153197-14-9)
635	Oxfendazole	(53716-50-0)
636	Oxycarboxin	(5259-88-1)
637	Oxydemeton Methyl Sulfone	(17040-19-6)
638	Oxyfluorfen	(42874-03-3)
639	p.p-Dichlorobenzophenone	(90-98-2)
640	Paclobutrazole	(76738-62-0)
641	Paraoxon	(311-45-5)
642	Paraoxon-methyl	(950-35-6)

643	Parathion	(56-38-2)
644	Parathion-methyl	(298-00-0)
645	Pebulate	(1114-71-2)
646	Penconazole	(66246-88-6)
647	Pencycuron	(66063-05-6)
648	Pendimethalin	(40487-42-1)
649	Penflufen	(494793-67-8)
650	Penfluron	(35367-31-8)
651	Penoxsulam	(219714-96-2)
652	Pentachlorophenol. PCP	(87-86-5)
653	Pentanochlor	(2307-68-8)
654	Penthiopyrad	(183675-82-3)
655	Pentoxazone	(110956-75-7)
656	Permethrin	(52645-53-1)
657	Permethrin (cis-)	(61949-76-6)
658	Pethoxamid	(106700-29-2)
659	Pethoxamide metabolite MET-42	(na)
660	Phenmedipham	(13684-63-4)
661	Phenmedipham metabolite MHPC	(13683-89-1)
662	Phenothrin	(26002-80-2)
663	Phenthoate	(2597-03-7)
664	Phorate	(298-02-2)
665	Phorate-oxon	(2600-69-3)
666	Phorate-oxon-sulfone	(2588-06-9)
667	Phorate-oxon-sulfoxide	(2588-05-8)
668	Phorate-sulfone	(2588-04-7)
669	Phorate-sulfoxide	(2588-03-6)
670	Phosalone	(2310-17-0)
671	Phosmet	(732-11-6)
672	Phosmet-oxon	(3735-33-9)
673	Phosphamidon (Dimecron)	(13171-21-6)
674	Phoxim	(14816-18-3)
675	Picolinafen	(137641-05-5)
676	Picolinicacid-methyl-ester 6-Chloro-2-	(6636-55-1)
677	Picoxystrobin	(117428-22-5)
678	Pindone	(83-26-1)
679	Pinoxaden	(243973-20-8)
680	Piperonylbutoxide	(51-03-6)
681	Piperophos	(24151-93-7)
682	Pirimicarb	(23103-98-2)
683	Pirimicarb D6	(1015854-66-6)

684	Pirimicarb Desmethyl	(30614-22-3)
685	Pirimicarb Desmethylformamido-	(27218-04-8)
686	Pirimiphos-ethyl	(23505-41-1)
687	Pirimiphos-methyl	(29232-93-7)
688	Prallethrin	(23031-36-9)
689	Pretilachlor	(51218-49-6)
690	Primisulfuron-methyl	(86209-51-0)
691	Probenazole	(27605-76-1)
692	Prochloraz	(67747-09-5)
693	Prochloraz metabolite BTS 44595	(139520-94-8)
694	Prochloraz metabolite BTS 44596	(na)
695	Prochloraz metabolite BTS 9608	(na)
696	Procymidone	(32809-16-8)
697	Profenophos	(41198-08-7)
698	Profoxydim	(139001-49-3)
699	Prohexadione	(88805-35-0)
700	Promecarb	(2631-37-0)
701	Prometon	(1610-18-0)
702	Prometryn (Caparol)	(7287-19-6)
703	Propachlor	(1918-16-7)
704	Propamocarb	(24579-73-5)
705	Propamocarb-N-oxide	(na)
706	Propanil	(709-98-8)
707	Propaphos	(7292-16-2)
708	Propargite	(2312-35-8)
709	Propazine	(139-40-2)
710	Propetamphos	(31218-83-4)
711	Propham	(122-42-9)
712	Propiconazole	(60207-90-1)
713	Propionic acid 3-(3-Indolyl)-	(830-96-6)
714	Propoxur	(114-26-1)
715	Propoxycarbazone	(145026-81-9)
716	Propyzamide (Pronamide)	(23950-58-5)
717	Proquinazid	(189278-12-4)
718	Prosulfocarb	(52888-80-9)
719	Prosulfuron	(94125-34-)
720	Prothioconazole	(178928-70-6)
721	Prothioconazole desthio	(120983-64-4)
722	Prothiofos (Tokuthion)	(34643-46-4)
723	Prothoate	(2275-18-5)
724	Pymetrozine	(123312-89-0)

725	Pyracarbolid	(24691-76-7)
726	Pyraclofos	(77458-01-6)
727	Pyraclostrobin	(175013-18-0)
728	Pyraflufen-ethyl	(129630-19-9)
729	Pyrasulfotole	(365400-11-9)
730	Pyrazolynate	(58011-68-0)
731	Pyrazophos	(13457-18-6)
732	Pyrazoxyfen	(71561-11-0)
733	Pyrethrin	(121-21-1)
734	Pyrethrins: Cinerin	(25402-06-6)
735	Pyrethrins: Jasmolin	(4466-14-2)
736	Pyribenzoxim	(168088-61-7)
737	Pyributicarb	(88678-67-5)
738	Pyridaben	(96489-71-3)
739	Pyridafenthion	(119-12-0)
740	Pyridafol	(40020-01-7)
741	Pyridalyl	(179101-81-6)
742	Pyridate	(55512-33-9)
743	Pyrifenox	(88283-41-4)
744	Pyrifluquinazon	(337458-27-2)
745	Pyriftalid	(135186-78-6)
746	Pyrimethanil	(53112-28-0)
747	Pyrimidifen	(105779-78-0)
748	Pyriminobac-methyl	(136191-64-5)
749	Pyrimisulfan	(221205-90-9)
750	Pyriofenone	(688046-61-9)
751	Pyriproxyfen	(95737-68-1)
752	Pyroquilon	(57369-32-1)
753	Pyroxsulam	(422556-08-9)
754	Quinalphos	(13593-03-8)
755	Quinclorac	(84087-01-4)
756	Quinmerac	(90717-03-6)
757	Quinoclamine	(2797-51-1)
758	Quinolinol 1-2-Dihydro-2-2-4-trimethyl-6-	(na)
759	Quinolinone 2-2-4-Trimethyl-6(2H)-	(na)
760	Quinoxyphen	(124495-18-7)
761	Rabenzazole	(40341-04-6)
762	Resmethrin	(10453-86-8)
763	Rimsulfuron	(122931-48-0)
764	Rotenone	(83-79-4)
765	Saflufenacil	(372137-35-4)

766	Saflufenacil Didesmethyl-	(na)
767	Saflufenacil M800H11	(na)
768	Saflufenacil M800H35	(na)
769	Saflufenacil Metabolite M800H35	(1246768-31-9)
770	Schradan	(152-16-9)
771	Sebuthylazine	(7286-69-3)
772	Secbumeton	(26259-45-0)
773	Sedaxane	(874967-67-6)
774	Sethoxydim	(74051-80-2)
775	Siduron	(1982-49-6)
776	Silafluofen	(105024-66-6)
777	Silthiofam	(175217-20-6)
778	Simazine	(122-34-9)
779	Simazine 2-Hydroxy	(2599-11-3)
780	Simeconazole	(149508-90-7)
781	Simetryn	(1014-70-6)
782	Spinetoram	(187166-40-1)
783	Spinosad A	(168316-95-8. 131929-60-7)
784	Spinosad D	(168316-95-8. 131929-63-0)
785	Spinosyn B or K	(168316-95-8. 131929-63-0 (B))
786	Spirodiclofen	(148477-71-8)
787	Spiromesifen	(283594-90-1)
788	Spirotetramate	(203313-25-1)
789	Spirotetramate-enol	(203312-38-3)
790	Spirotetramate-enol-glucoside	(1172614-86-6)
791	Spirotetramate-keto-hydroxy	(1172134-11-0)
792	Spirotetramate-mono-hydroxy	(1172134-12-1)
793	Spiroxamine	(118134-30-8)
794	Sulcotrione	(99105-77-8)
795	Sulcotrione metabolite CMBA	(na)
796	Sulfanilamide	(63-74-1)
797	Sulfentrazone	(122836-35-5)
798	Sulfometuron-methyl	(74222-97-2)
799	Sulfosulfuron	(141776-32-1)
800	Sulfotepp	(3689-24-5)
801	Sulfoxaflor	(946578-00-3)
802	Sulprofos (Bolstar)	(35400-43-2)
803	SWEP.MCC	(1918-18-9)
804	TBSA Triflusulfuron metabolite	(1869-24-5)
805	TCMTB	(21564-17-0)

806	Tebuconazole	(107534-96-3)
807	Tebuconazole D6	(na)
808	Tebufenozide	(112410-23-8)
809	Tebufenpyrad	(119168-77-3)
810	Tebupirimphos	(96182-53-5)
811	Tebutame	(35256-85-0)
812	Tebuthiuron	(34014-18-1)
813	Tecloftalam	(76280-91-6)
814	Teflubenzuron	(83121-18-0)
815	Tefluthrin	(79538-32-2)
816	Tembotrione	(335104-84-2)
817	Temephos	(3383-96-8)
818	TEPP	(107-49-3)
819	Tepraloxydim	(149979-41-9)
820	Terbacil	(5902-51-2)
821	Terbufos	(13071-79-9)
822	Terbufos-Oxon-sulfone	(na?)
823	Terbufos-Oxon-sulfoxide	(56165-57-2)
824	Terbufossulfone	(56070-16-7)
825	Terbufos-sulfoxid	(10548-10-4)
826	Terbumeton	(33693-04-8)
827	Terbuthylazine	(5915-41-3)
828	Terbuthylazine-Desethyl	(30125-63-4)
829	Terbutryn	(886-50-0)
830	Tetrachlorvinphos (Stirofos)	(22248-79-9)
831	Tetraconazole	(112281-77-3)
832	Tetradifon	(116-29-0)
833	Tetramethrin	(7696-12-0)
834	TFNA (metabolite flonicamid)	(158063-66-2)
835	Thenylchlor	(96491-05-3)
836	Thiabendazole	(148-79-8)
837	Thiabendazole-5-hydroxy	(948-71-0)
838	Thiacloprid	(111988-49-9)
839	Thiamethoxam	(153719-23-4)
840	Thiazafluron	(25366-23-8)
841	Thiazopyr	(117718-60-2)
842	Thidiazuron	(51707-55-2)
843	Thiencarbazone-methyl	(317815-83-1)
844	Thifensulfuron-methyl	(79277-27-3)
845	Thifluzamide	(130000-40-7)
846	Thiobencarb	(28249-77-6)

847	Thiocyclam	(31895-21-3)
848	Thiodicarb	(59669-26-0)
849	Thiofanox	(39196-18-4)
850	Thiofanox sulfone	(39184-59-3)
851	Thiofanox sulfoxide	(39184-27-5)
852	Thiomethon	(640-15-3)
853	Thionazin (Zinophos)	(297-97-2)
854	Thiophanate-methyl	(23564-05-8)
855	Thiophanat-ethyl	(23564-06-9)
856	Thiosultap	(98968-92-4)
857	Thiram (Tetramethylthiuramdisulfide.TMTD)	(137-26-8)
858	Tiocarbazil	(36756-79-3)
859	Tolclofos-methyl	(57018-04-9)
860	Tolfenpyrad	(129558-76-5)
861	Tolyfluanid	(731-27-1)
862	Topramezone	(210631-68-8)
863	Topramezone metabolite M670H05	(223646-24-0)
864	Tralkoxydim	(87820-88-0)
865	Tralomethrin	(66841-25-6)
866	Transfluthrin	(118712-89-3)
867	Triadimefon	(43121-43-3)
868	Triadimenol	(55219-65-3)
869	Triallate	(2303-17-5)
870	Triapenthenol	(76608-88-3)
871	Triasulfuron	(82097-50-5)
872	Triazamate	(112143-82-5)
873	Triazophos	(24017-47-8)
874	Triazoxide	(72459-58-6)
875	Tribenuron methyl	(101200-48-0)
876	Tribufos (Merphos oxide. DEF)	(78-48-8)
877	Trichlamide	(70193-21-4)
878	Trichlorfon (Dylox)	(52-68-6)
879	Triclocarban	(101-20-2)
880	Triclopyr	(55335-06-3)
881	Triclopyr-methylester	(60825-26-5)
882	Triclosan	(3380-34-5)
883	Tricyclazole	(41814-78-2)
884	Tridemorph	(81412-43-3)
885	Trietazine	(1912-26-1)
886	Trifloxystrobin	(141517-21-7)
887	Trifloxysulfuron	(145099-21-4)

888	Triflumizol	(68694-11-1. old:99387-89-0)
889	Triflumizol Metabolite FM-6-1	(131549-75-2)
890	Triflumizole	(99387-89-0)
891	Triflumizole FM-6-1	(109849-99-2)
892	Triflumuron	(64628-44-0)
893	Triflusufuron-m metabolite IN-D8526	(na)
894	Triflusulfuron-methyl	(126535-15-7)
895	Triforine	(26644-46-2)
896	Trimethacarb (2.3.5-)	(12407-86-2)
897	Trinexapac free acid	(104273-73-6)
898	Trinexapac-ethyl	(95266-40-3)
899	Triphenylphosphate	(115-86-6)
900	Triticonazole	(131983-72-7)
901	Tritosulfuron	(142469-14-5)
902	Tritosulfuron metabolite AMTT	(5311-05-7)
903	Uniconazole	(83657-22-1)
904	Uracil	(66-22-8)
905	Valifenalate	(283159-90-0)
906	Vamidothion	(2275-23-2)
907	Vegadex (Sulfallate)	(95-06-7)
908	Vernolate	(1929-77-7)
909	Vinclozolin	(50471-44-8)
910	Warfarin	(81-81-2)
911	XMC	(2655-14-3)
912	Zoxamide	(156052-68-5)
913	3 5 6-Trichloro-2-pyridinol (TCPy)	(6515-38-4)
914	4-Chlor-2-methylphenol	(1570-64-5)
915	AD-67	(71526-07-3)
916	Aminobenzimidazole 2-	(934-32-7)
917	Anilazine (Zinochlor)	(101-05-3)
918	Anthraquinone	(84-65-1)
919	Asulam	(3337-71-1)
920	Asulam metabolite Acetyl Sulfanilamid	(na)
921	BAC 10	(965-32-2)
922	BAC 12	(139-07-1)
923	BAC 14	(139-08-2)
924	BAC 16	(122-18-9)
925	BAC 18	(122-19-0)
926	Benfuresate NH4-adduct	(68505-69-1)
927	Benzisothiazol-3(2H)-one 1-2-	(2634-33-5)

928	Benzoic acid 3-6-Dichloro-2-hydroxy- (dicamba metabolite)	(3401-80-7)
929	Benzovindiflupyr	(1072957-71-1)
930	Benzylaminopurine 6-	(1214-39-7)
931	Carbamate Ethyl-N-(3-hydroxyphenyl)-	(7159-96-8)
932	Carbanilide	(102-07-8)
933	Carbanolate	(671-04-5)
934	Carbophenothion-Methyl	(953-17-3)
935	Cetylpyridinium chloride	(6004-24-6)
936	Chinomethionat	(2439-01-2)
937	Chlorantraniliprole	(500008-45-7)
938	Chlorfluazuron	(71422-67-8)
939	Chloridazon-desphenyl-methyl	(17254-80-7)
940	Chlorothalonil-4-hydroxy	(na)
941	Chlozolinate	(72391-46-9)
942	Cloprop	(101-10-0)
943	Coumafuryl	(117-52-2)
944	Cycloxydim	(101205-02-1)
945	Cyenopyrafen	(560121-52-0)
946	Cyhalofop acid	(122008-78-0)
947	DDAC-C10	(7173-51-5)
948	DDAC-C12	(3401-74-9)
949	Diafenthiuron metabolite CGA 140408	(na)
950	Diafenthiuron metabolite CGA 177960	(na)
951	Dibromo-4-hydroxy-benzoic acid 3-5-	(3337-62-0)
952	Difenacoum	(56073-07-5)
953	Dihydroquinoline 2-2-4-Trimethyl-1-2-	(147-47-7)
954	Dimethachlor metabolite CGA 369873	(na)
955	Dimethoate metabolite Desmethyl Dimethoate	(na)
956	Dimethoate metabolite Dimethoate carboxylic acid	(1113-01-5)
957	Dinocap-Meptyl Ester NH4-adduct	(131-72-6 / 6119-92-2)
958	Dinocap-Meptyl Phenol	(3687-22-7)
959	Diphenylamine N-Nitroso-	(86-30-6)
960	Dithianon	(3347-22-6)
961	DMSA	(4710-17-2)
962	Ethoxy-1-2-3-4-tetra-hydro-2-2-4-trimethylchinolin 6-	(16489-90-0)
963	Fenpropimorph Carboxylic Acid	(121098-45-1)
964	Fensulfothion-oxon	(6552-21-2)
965	Fipronil-desulfinyl	(205650-65-3)

966	Fipronil-sulfide	(120067-83-6)
967	Flonicamid metabolite TFNA-AM	(158062-71-6)
968	Flonicamid metabolite TFNG	(207502-65-6)
969	Florasulam	(145701-23-1)
970	Fluazifop-P-Butyl metabolite CGA 142110	(33252-63-0)
971	Fluazinam	(79622-59-6)
972	Fluchloralin	(33245-39-5)
973	Flufenacet sulfonic acid (FOE 5043-sulfonic acid)	(947601-87-8)
974	Flufenacet thioglycolate sulfoxide (FOE 5043 thioglycolate sulfoxide)	(201668-33-9)
975	Flufenacet-oxalate (FOE 5043-oxalate)	(201668-31-7)
976	Fluometuron	(2164-17-2)
977	Flusulfamide	(106917-52-6)
978	Folpet	(133-07-3)
979	Formothion	(2540-82-1)
980	Halosulfuron-methyl	(100784-20-1)
981	Homobrassinolide 22(S)-23(S)-	(80483-89-2)
982	Imazapic	(104098-48-8)
983	Indole-3-butyric acid	(133-32-4)
984	Indolyl-acetic-acid-ethyl-ester 3-	(778-82-5)
985	Iodocarb	(55406-53-6)
986	Iodophenoxyacetic acid 4-	(1878-94-0)
987	Iprodione metabolite RP 30228	(na)
988	Isofenphos-Oxon	(31120-85-1)
989	Isothiazolin-3-one 2-Methyl-4- Peak 1	(2682-20-4)
990	Isothiazolin-3-one 5-Chloro-2-methyl-4-	(26172-55-4)
991	Isotianil	(224049-04-1)
992	Kinetin	(525-79-1)
993	Kresoxim-Methyl metabolite BF 490-1	(137169-29-0)
994	Mandestrobin	(173662-97-0)
995	Mepanipyrim metabolite KIF-3535-M-31	(204571-52-8)
996	Mepiquat 4-Hydroxy (Piperidinium)	(na)
997	Mesotrione metabolite AMBA	(393085-45-5)
998	Metaflumizone e-isomere	(139968-49-3)
999	Metalaxyl metabolite CGA 107955	(104390-55-8)
1000	Metalaxyl metabolite CGA 108905	(na)
1001	Metalaxyl metabolite CGA 67869	(66637-79-4)
1002	Metalaxyl metabolite CGA 94689	(na)
1003	Milbemectin A4	(51596-11-3)
1004	Naproanilide	(52570-16-8)
1005	Nitroguaiacol 5-	(na)

1006	Noviflumuron	(121451-02-3)
1007	Orthosulfamuron	(213464-77-8)
1008	Penflufen	(494793-67-8)
1009	Pentachlorophenol	(87-86-5)
1010	Penthiopyrad	(183675-82-3)
1011	Pethoxamide metabolite MET-42	(na)
1012	Phenkapton	(2275-14-1)
1013	Phenmedipham metabolite MHPC	(13683-89-1)
1014	Picolinicacid-methyl-ester 6-Chloro-2-	(6636-55-1)
1015	Pindone	(83-26-1)
1016	Prallethrin	(23031-36-9)
1017	Prochloraz metabolite BTS 44595	(139520-94-8)
1018	Prochloraz metabolite BTS 44596	(na)
1019	Prochloraz metabolite BTS 9608	(na)
1020	Prohexadione	(88805-35-0)
1021	Propamocarb-N-oxide	(na)
1022	Propionic acid 3-(3-Indolyl)-	(830-96-6)
1023	Propoxycarbazone	(145026-81-9)
1024	Prothiofos	(34643-46-4)
1025	Prothoate	(2275-18-5)
1026	Pyrasulfotole	(365400-11-9)
1027	Pyribenzoxim	(168088-61-7)
1028	Pyridafenthion	(119-12-0)
1029	Pyridafol	(40020-01-7)
1030	Pyrimisulfan	(221205-90-9)
1031	Pyriofenone	(688046-61-9)
1032	Quinolinol 1-2-Dihydro-2-2-4-trimethyl-6-	(na)
1033	Quinolinone 2-2-4-Trimethyl-6(2H)-	(na)
1034	Saflufenacil Didesmethyl-	(na)
1035	Saflufenacil Metabolite M800H35	(1246768-31-9)
1036	Sedaxane	(874967-67-6)
1037	Sulcotrione metabolite CMBA	(na)
1038	Sulfanilamide	(63-74-1)
1039	Sulfentrazone	(122836-35-5)
1040	Sulfoxaflor	(946578-00-3)
1041	TBSA Triflusulfuron metabolite	(1869-24-5)
1042	Tecloftalam	(76280-91-6)
1043	Terbuthylazine-Desethyl	(30125-63-4)
1044	Thiabendazole-5-hydroxy	(948-71-0)
1045	Thifluzamide	(130000-40-7)
1046	Thiomethon	(640-15-3)

1047	Thionazin	(297-97-2)
1048	Thiosultap	(98968-92-4)
1049	Topramezone	(210631-68-8)
1050	Topramezone metabolite M670H05	(223646-24-0)
1051	Transfluthrin	(118712-89-3)
1052	Triapenthenol	(76608-88-3)
1053	Trichlamide	(70193-21-4)
1054	Triclosan	(3380-34-5)
1055	Triflumizole FM-6-1	(109849-99-2)
1056	Triflusufuron-m metabolite IN-D8526	(na)
1057	Triflusulfuron-methyl	(126535-15-7)
1058	Trinexapac free acid	(104273-73-6)
1059	Tritosulfuron metabolite AMTT	(5311-05-7)
1060	Uracil	(66-22-8)
