# Supplementary material for: Assessment of Xenoestrogens in Jordanian Water System: Activity and Identification
Source: Toxics. 2023 Jan 9;11(1):63. doi: 10.3390/toxics11010063 (PMC9866086; doi:10.3390/toxics11010063)
Supplement: Supplementary file 1 [file toxics-11-00063-s001.zip › Supplementary Material S4.pdf]

Supplementary Material S4

List of Potential Xenoestrogens according to literature

	Compound	Chemical Formula	M.Wt	CAS #	Occurrence and use
**Naturally occurring**					
1	20S-Protopanaxadiol	C30H52O3	460.7	30636-90-9	Gastrointestinal metabolic product of ginsenosides
2	Alpha-zearalenol	C18H24O5	320.4	36455-71-7	Metabolite of zearalenone, used as growth promoter in beef cattle in certain non-European Union countries
3	Apigenin	C15H10O5	270.24	520-36-5	Flavonoid phytoestrogen
4	Ferutinine	C22H30O4	358.5	41743-44-6	Sesquiterpenoid, Umbelliferae family
5	Formononetin	C16H12O4	268.26	485-72-3	Isoflavonoid, in red clover and soy extracts
6	Genistein	C15H10O5	270.24	446-72-0	Isoflavonoid, in red clover and soy extracts
7	Kaempferol	C15H10O6	286.24	520-18-3	Flavonoid phytoestrogen
8	1,5-Dibromo-2-(2,4-dibromophenoxy)-3-methoxybenzene	C13H8Br4O2	515.8	102739-99-1	Occur naturally in marine organisms
9	Zearalenone	C18H22O5	318.4	17924-92-4	Mycotoxin, food contaminant
**Industrial products and pollutants including organohalogens, plastic components, detergents**					
1	3,9-Dihydroxy-benz[a]anthracene	C18H12O2	260.3	56614-97-2	Potential metabolite of benz[a]anthracene
2	3-Methylcholanthrene	C21H16	268.4	56-49-5	PAH, mainly incomplete combustion
3	4-n-Octylphenol	C14H22O	206.32	1806-26-4	Intermediate in production of and degradation product of octylphenol ethoxylates used to in rubber, in pesticides and paints

4	4-Nonylphenol	C15H24O	220.35	104-40-5	Precursor to and degradation product of commercially important detergents
5	4-n-Propylphenol	C9H12O	136.19	645-56-7	Degradation product of surfactants
6	4-Tert-butyl 2-methylphenol	C11H16O	164.24	98-27-1	Industrial product and environmental contaminant
7	4-Tert-octylphenol	C14H22O	206.32	140-66-9	Used in the manufacture of nonionic surfactants
8	7,12-Dimethyl-benz(a)anthracene	C20H15F	274.3	737-22-4	PAH, mainly < incomplete combustion
9	Benzotriazole	C6H5N3	119.12	95-14-7	Anticorrosive agent used in aircraft de-icing, antifreeze fluids, and dishwasher detergents
10	Benzyl butyl phthalate	C19H20O4	312.4	85-68-7	Plasticizer for polyvinyl chloride in vinyl floor tiles, vinyl foam, and carpet backing, in cellulosic resins
11	Bisphenol A	C15H16O2	228.29	80-05-7	Used in the production of polycarbonate plastic and epoxy resins used as coatings on the inside of food and beverage cans
12	Dibutylphthalate	C16H22O4	278.34	84-74-2	Mainly used as plasticiser, also in paints, inks, and cosmetics
13	Dichlorostyrene	C8H6Cl2	173.04	50852-77-2	Intermediate in chemical industry
14	Dodecylphenol	C18H30O	262.4	27193-86-8	Used in phenol resins in adhesives and paints
15	Nonylphenols	C15H24O	220.35	25154-52-3	Precursor to and degradation product of commercially important detergents
16	Polybrominated diphenyl ether	C12H4Br6O3	675.6	80246-27-1	Flame retardants used in plastics, foams, building materials, electronics,

					furnishings, motor vehicles
	Polychlorinated biphenyl	C12H4Cl6	360.9	32774-16-6	Industrial products, used as dielectric and coolant fluids
	**Substances used in personal care products**				
1	3-Benzylidene camphor	C17H20O	240.34	15087-24-8	UV filters, used in sunscreens and cosmetics
2	4,4-Dihydroxy-benzophenone	C13H10O3	214.22	611-99-4	UV filter, used in sunscreens and cosmetics
3	4-Hydroxybenzoic acid n-butyl ester	C11H14O4	210.23	123910-88-3	Preservatives in foods, drugs, and personal products
4	4-hydroxybenzoic acid ethyl ester	C9H10O3	166.17	120-47-8	UV filter, used in sunscreens and cosmetics
5	4-hydroxybenzoic acid propyl ester	C10H12O3	180.2	94-13-3	UV filter, used in sunscreens and cosmetics
6	4-Hydroxybenzophenone	C13H10O2	198.22	1137-42-4	Metabolite of UV filter, used in sunscreens and cosmetics
7	4-Methylbenzylidene camphor	C18H22O	254.4	36861-47-9	UV filters, used in sunscreens and cosmetics
8	Benzophenone-1	C13H10O3	214.22	131-56-6	UV filter, used in sunscreens and cosmetics
9	Benzophenone-2	C13H10O5	246.21	131-55-5	UV filter, used in sunscreens and cosmetics
10	Benzophenone-3	C14H12O3	228.24	131-57-7	UV filter, used in sunscreens and cosmetics
11	Benzophenone-4	C14H12O6S	308.31	4065-45-6	UV filter, used in sunscreens and cosmetics
12	Benzyl salicylate	C14H12O3	228.24	118-58-1	UV filter, used in sunscreens and cosmetics
13	Ethoxylated ethyl 4-amino benzoate				UV filter, used in sunscreens and cosmetics

14	Ethyl-4-aminobenzoate	C9H11NO2	165.19	94-09-7	UV filter, used in sunscreens and cosmetics
15	Eusolex 2292,	C18H26O3	290.4	5466-77-3	UV filter, used in sunscreens and cosmetics
16	Eusolex 6300,	C18H22O	254.4	36861-47-9	UV filter, used in sunscreens and cosmetics
17	Eusolex 6007,	C17H27NO2	277.4	21245-02-3	UV filter, used in sunscreens and cosmetics
18	Eusolex HMS				UV filter, used in sunscreens and cosmetics
19	Galaxolide (HHCB)	C18H26O	258.4	1222-05-5	Fragrance, used in sunscreens and cosmetics
20	Homosalate	C16H22O3	262.34	118-56-9	UV filter, used in sunscreens and cosmetics
21	Isopentyl-4-methoxycinnamate	C15H20O3	248.32	71617-10-2	UV filter, used in sunscreens and cosmetics
22	Octocrylene	C24H27NO2	361.5	6197-30-4	UV filter, used in sunscreens and cosmetics
23	Octyl dimethyl para amino benzoate	C17H27NO2	277.4	58817-05-3	UV filter, used in sunscreens and cosmetics
24	Octyl salicylate	C15H22O3	250.33	6969-49-9	UV filter, used in sunscreens and cosmetics
25	Octyl-methoxycinnamate	C18H26O3	290.4	83834-59-7	UV filter, used in sunscreens and cosmetics
26	Para amino-benzoic acid	C7H7NO2	137.14	150-13-0	UV filter, used in sunscreens and cosmetics
27	Phenyl salicylate	C13H10O3	214.22	118-55-8	UV filter, used in sunscreens and cosmetics
28	Tonalide (AHTN)	C18H26O	258.4	21145-77-7	UV filter, used in sunscreens and cosmetics
	**Pesticides**				

1	2,4,5-Trichloro-phenoxyacetic acid	C8H5Cl3O3	255.5	93-76-5	Herbicide, defoliant used in Agent Orange in Vietnam
2	3-(2,2-Dichlorovinyl)-2,2-dimethylcyclopropene carboxylic acid	C8H10Cl2O2	209.07	55701-05-8	Pyrethroid insecticide metabolite
3	3-Phenoxybenzoic acid	C13H10O3	214.22	3739-38-6	Pyrethroid insecticide metabolite
4	Alachlor	C14H20ClNO2	269.77	15972-60-8	Acid amide herbicide against grasses and broadleaf weeds. Use as herbicide banned in European Union (2006)
5	Aldrin	C12H8Cl6	364.9	309-00-2	Organochlorine insecticide, widely used until 1970s
6	Atrazine	C8H14ClN5	215.68	1912-24-9	Still widely used triazine herbicide, banned in the European Union
7	Azinphos-methyl	C10H12N3O3PS2	317.3	86-50-0	Organophosphate insecticide, banned in the European Union (2006)
8	Benomyl	C14H18N4O3	290.32	17804-35-2	Carbamate fungicide has been used on fruits
9	Bromopropylate	C17H16Br2O3	428.1	18181-80-1	Carbinol acaricide, used on fruits and legumes, in viticulture
10	Carbaryl	C12H11NO2	201.22	63-25-2	Carbamate insecticide, widely used in gardens, agriculture, and forestry
11	Chlomethoxyfen	C13H9Cl2NO4	314.12	32861-85-1	Diphenyl ether herbicide, used on rice
12	Chlordane	C10H6Cl8	409.8	12789-03-6	Organochlorine pesticide, banned in USA in 1988
13	Chlordecone	C10Cl10O	490.6	143-50-0	Organochlorine insecticide and fungicide, banned by the Stockholm Convention on persistent organic pollutants
14	Chlornitrofen	C12H6Cl3NO3	318.5	1836-77-7	Diphenyl ether herbicide

15	Chlorpropham	C10H12ClNO2	213.66	101-21-3	Carbamate herbicide and plant growth regulator
16	Chlorpyriphos	C9H11Cl3NO3PS	350.6	2921-88-2	Organophosphorus insecticide, highly used on corn and fruit trees, still frequently used in Danish greenhouses in 2001
17	Cycloprothrin	C26H21Cl2NO4	482.4	63935-38-6	Pyrethroid insecticide used in agriculture
18	Cyfluthrin	C22H18Cl2FNO3	434.3	68359-37-5	Pyrethroid insecticide, agriculture, home, garden
19	Cyhalothrin	C23H19ClF3NO3	449.8	68085-85-8	pyrethroid insecticide, agriculture, home, garden
20	Cypermethrin	C22H19Cl2NO3	416.3	52315-07-8	Pyrethroid insecticide, in agriculture, home, garden
21	Cyprodinil	C14H15N3	225.29	121552-61-2	Fungicide used in agriculture and on fruit
22	DDE	C14H8Cl4	318	72-55-9	DDT metabolite
23	DDT,	C14H9Cl5	354.5	50-29-3	Organochlorine pesticide. Banned
24	Deltamethrin	C22H19Br2NO3	505.2	52918-63-5	Pyrethroid insecticide, widely used, still frequently used in Danish greenhouses in 2001
25	Dichlorvos	C4H7Cl2O4P	220.97	62-73-7	Organophosphate insecticide widely used to control household pests, also on outdoor fruit, and vegetable crops. Frequently used in Danish greenhouses in 2001
26	Dicofol	C14H9Cl5O	370.5	115-32-2	Organochlorine pesticide for mite control on agricultural crops, ornamentals, and around buildings
27	Dieldrin	C12H8Cl6O	380.9	60-57-1	Organochlorine pesticide, now banned in many countries

28	Dimethomorph	C21H22ClNO4	387.9	110488-70-5	Cinnamic acid fungicide, used in agriculture (potatoes)
29	Endosulfan,	C9H6Cl6O3S	406.9	959-98-8	Organochlorine insecticide, banned by the Stockholm Convention (2011)
30	Endrin	C12H8Cl6O	380.9	72-20-8	Organochlorine pesticide, used in agriculture. Now banned in many countries
31	Ethoxyquin	C14H19NO	217.31	91-53-2	Quinoline-based antioxidant used as a food preservative (E324) and a pesticide
32	Etofenprox	C25H28O3	376.5	80844-07-1	Pyrethroid insecticide used in homes (pet and foggers)
33	Fenarimol	C17H12Cl2N2O	331.2	60168-88-9	Heterocyclic organochlorine fungicide, used on ornamentals, trees, horticulture (frequently used in Danish greenhouses in 2001)
34	Fenbuconazole	C19H17ClN4	336.8	114369-43-6	Triazole fungicide used in agriculture, fruit
35	Fenhexamid	C14H17Cl2NO2	302.2	126833-17-8	Fungicide, used on fruit, ornamentals, trees
36	Fenitrothion	C9H12NO5PS	277.24	122-14-5	Organophosphorus insecticide, agriculture, grain storage
37	Fenthion	C10H15O3PS2	278.3	55-38-9	Organothiophosphate insecticide, avicide, and acaricide, widely used in sugar cane, rice, field corn, beets, pome and stone fruit, citrus fruits, pistachio, cotton, olives, coffee, cocoa, vegetables, and vines
38	Fenvalerate	C25H22ClNO3	419.9	51630-58-1	Pyrethroid pesticide, agriculture, home, garden

39	Fludioxonil	C12H6F2N2O2	248.18	131341-86-1	Fungicide used on sowing-seed,vegetables, fruits, and ornamentals
40	Imazalil	C14H14Cl2N2O	297.2	35554-44-0	Imidazole fungicide: sowing seed, potatoes, and fruits after harvesting
41	Lindane	C6H6Cl6	290.8	319-84-6	Organochlorine insecticide, used to treat lice and scabies
42	Linuron	C9H10Cl2N2O2	249.09	330-55-2	Phenylureum herbicide: horticulture
43	Methiocarb	C11H15NO2S	225.31	2032-65-7	Carbamate insecticide, still frequently used in Danish greenhouses in 2001
44	Methoxychlor	C16H15Cl3O2	345.6	72-43-5	Organochlorine pesticide, used on crops, ornamentals, livestock, and pets against insects. Banned in the USA (2003) and European Union (2002)
45	Metolachlor	C15H22ClNO2	283.79	51218-45-2	Chloroacetanilide herbicide, grass and broadleaf weed control in agriculture
46	O-Phenylphenol	C12H10O	170.21	90-43-7	Fungicide, generally applied after harvest; used for waxing citrus fruits. As a food additive, it has E number E231
47	Pentachlorophenol	C6Cl5OH	266.3	87-86-5	Organochlorine pesticide: herbicide, insecticide, fungicide, antifouling paint, preservation of wood. Use now restricted
48	Permethrin	C21H20Cl2O3	391.3	52645-53-1	Pyrethroid insecticide, agriculture, home, garden
49	Pirimicarb	C11H18N4O2	238.29	23103-98-2	Carbamate insecticide: vegetable, cereal, and orchard crops

50	Pirimiphos-methyl	C11H20N3O3PS	305.34	29232-93-7	Phosphorothioate insecticide: postharvest insecticide used on stored corn and sorghum grain and seed
51	Prochloraz	C15H16Cl3N3O2	376.7	67747-09-5	Imidazole-type foliar fungicide:gardening and agriculture, still frequently used in Danish greenhouses in 2001.
52	Procymidone	C13H11Cl2NO2	284.13	32809-16-8	Dicarboximide fungicide: preharvest spray or postharvest dip of lupins, grapes, stone fruit, strawberries
53	Propamocarb	C9H20N2O2	188.27	24579-73-5	Carbamate fungicide: horticulture, ornamentals, agriculture
54	Pyrazoxyfen	C20H16Cl2N2O3	403.3	71561-11-0	Benzoylpyrazole herbicide
55	Pyrimethanil	C12H13N3	199.25	53112-28-0	Fungicide: fruit,vineyards
56	Quinoxyfen	C15H8Cl2FNO	308.1	124495-18-7	Fungicide:cereals,beets, strawberries, some berries
57	Tebuconazole	C16H22ClN3O	307.82	107534-96-3	Triazole fungicide: agriculture. Assessed by the Swedish Chemicals Agency as potentially toxic;
58	Tetramethrin	C19H25NO4	331.4	7696-12-0	Pyrethroid insecticide: Primarily pressurized aerosols in household, industrial, commercial, and institutional settings
59	Tolclofos-methyl	C9H11Cl2O3PS	301.13	57018-04-9	Organophosphorous fungicide, still frequently used in Danish greenhouses in 2001
60	Toxaphene	C10H8Cl8	411.8	8001-35-2	Organochlorine insecticide (banned in many countries)
61	Trans-nonachlor	C10H5Cl9	444.2	5103-73-1	Organochlorine insecticide: agriculture

62	tribenuron-methyl	C15H17N5O6S	395.4	101200-48-0	Herbicide: grains; still frequently used in Danish greenhouses in 2001
63	Triflumizole	C15H15ClF3N3O	345.75	68694-11-1	Conazole fungicide: plant protection
64	Vinclozolin	C12H9Cl2NO3	286.11	50471-44-8	Dicarboximide fungicide, used on fruits and vegetables, still frequently used in Danish greenhouses in 2001. Use now strictly regulated
