# Supplementary material for: Assessment of Xenoestrogens in Jordanian Water System: Activity and Identification
Source: Toxics. 2023 Jan 9;11(1):63. doi: 10.3390/toxics11010063 (PMC9866086; doi:10.3390/toxics11010063)
Supplement: Supplementary file 1 [file toxics-11-00063-s001.zip › table S1.pdf]

Compound Spectrum List Report

**Analysis Info**	24_August_2021_Dr_ Yazan alakam_2	Acquisition Date	24/08/2021
Method	Kailani _MS/MS	Operator	Demo User
		Instrument	impact II 1825265.10265

Acquisition Parameter

Source Type	ESI	Ion Polarity	Positive	Set Nebulizer	2.0 Bar
Focus	Active	Set Capillary	2500 V	Set Dry Heater	200 °C
Scan Begin	30 m/z	Set End Plate Offset	-500 V	Set Dry Gas	8.0 l/min
Scan End	1000 m/z	Set Charging Voltage	2000 V		

Cmpd 1, Dissect, 1.3 min

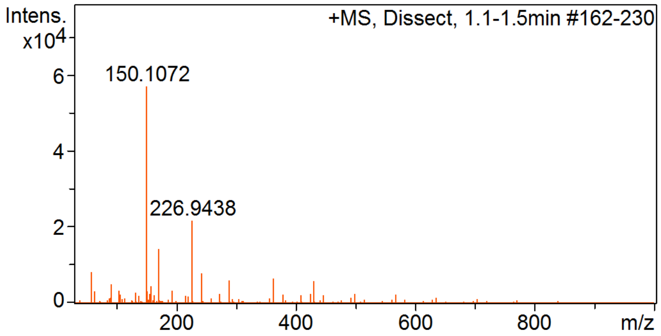

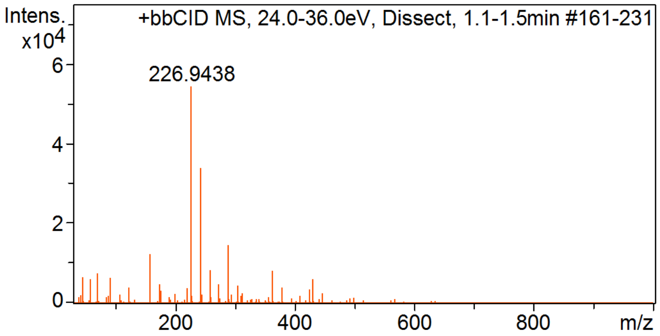

#	m/z	Res.	S/N	I	I %	FWHM
1	58.0629	17235	144.7	8273	14.5	0.0034
2	90.9736	19427	91.1	5208	9.1	0.0047
3	150.1072	27037	998.4	57065	100.0	0.0056
4	158.9586	26619	80.7	4612	8.1	0.0060
5	172.0886	27547	250.6	14326	25.1	0.0062
6	226.9438	31578	382.1	21842	38.3	0.0072
7	242.9173	30799	140.8	8045	14.1	0.0079
8	288.9120	29233	107.0	6115	10.7	0.0099
9	362.9142	33919	115.2	6582	11.5	0.0107
10	430.8992	31478	103.4	5909	10.4	0.0137

#	m/z	Res.	S/N	I	I %	FWHM
1	45.0315	15747	123.2	6708	12.3	0.0029
2	70.0626	18185	139.6	7605	14.0	0.0039
3	90.9737	19692	119.2	6490	11.9	0.0046
4	158.9585	27342	228.0	12416	22.8	0.0058
5	226.9438	31428	998.6	54390	100.0	0.0072
6	242.9172	32344	623.2	33945	62.4	0.0075
7	258.8906	28962	155.0	8442	15.5	0.0089
8	288.9119	30619	270.6	14737	27.1	0.0094
9	362.9141	31292	152.0	8278	15.2	0.0116
10	430.8988	31197	114.1	6215	11.4	0.0138

Compound Spectrum List Report

Cmpd 2, Dissect, 1.4 min

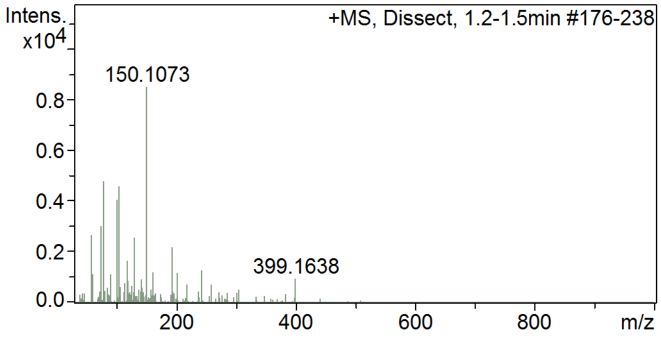

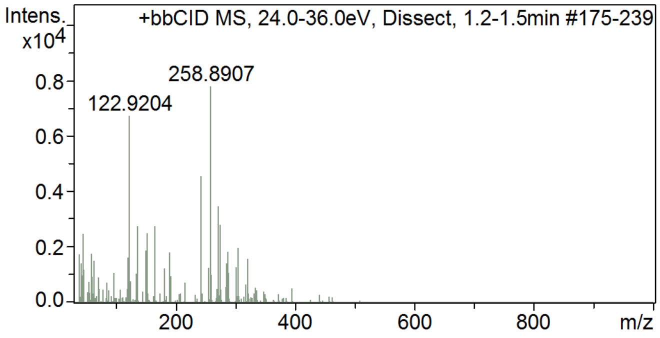

#	m/z	Res.	S/N	I	I %	FWHM
1	58.0629	17045	312.5	2664	31.3	0.0034
2	74.0937	18279	355.0	3026	35.6	0.0041
3	79.0184	18790	560.5	4778	56.2	0.0042
4	101.0564	20167	476.3	4060	47.7	0.0050
5	104.1037	21049	537.2	4579	53.8	0.0049
6	118.0823	23275	195.0	1662	19.5	0.0051
7	130.1044	23115	301.7	2571	30.2	0.0056
8	150.1073	26676	998.0	8507	100.0	0.0056
9	194.1319	27084	257.4	2194	25.8	0.0072
10	242.9173	29638	151.6	1292	15.2	0.0082

#	m/z	Res.	S/N	I	I %	FWHM
1	45.0316	15821	320.4	2495	32.0	0.0028
2	122.9204	23864	862.8	6721	86.3	0.0052
3	135.9723	25622	353.8	2756	35.4	0.0053
4	151.9458	27061	321.4	2503	32.1	0.0056
5	165.9820	27781	354.4	2761	35.4	0.0060
6	242.9173	31877	585.4	4560	58.5	0.0076
7	258.8907	29635	999.8	7788	100.0	0.0087
8	271.9431	32400	445.6	3471	44.6	0.0084
9	274.8642	30420	359.7	2802	36.0	0.0090
10	304.8850	29689	255.6	1991	25.6	0.0103

Cmpd 3, Dissect, 1.5 min

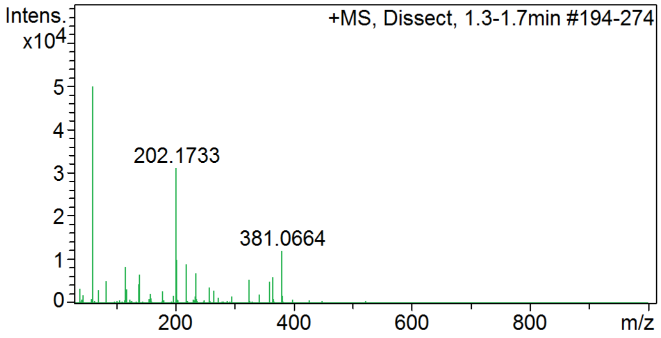

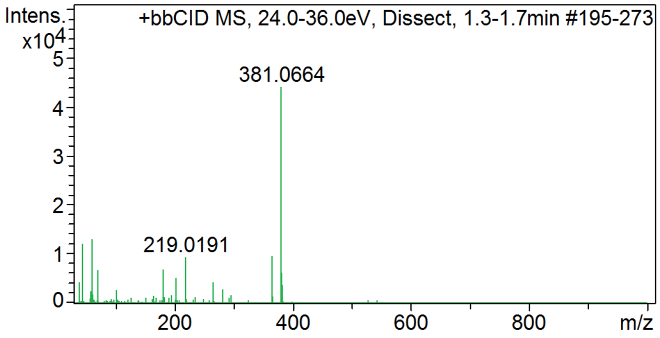

#	m/z	Res.	S/N	I	I %	FWHM
1	61.0373	17231	999.1	49959	100.0	0.0035
2	116.0668	22358	169.1	8455	16.9	0.0052
3	139.0455	25474	133.1	6657	13.3	0.0055
4	202.1733	29776	622.7	31135	62.3	0.0068
5	203.0457	29480	201.1	10055	20.1	0.0069
6	219.0191	29801	180.8	9039	18.1	0.0073
7	236.1415	31336	140.4	7019	14.1	0.0075
8	325.1018	31714	109.1	5455	10.9	0.0103
9	365.0932	32328	122.7	6133	12.3	0.0113
10	381.0664	32204	242.3	12115	24.2	0.0118

#	m/z	Res.	S/N	I	I %	FWHM
1	38.9615	14619	99.3	4385	9.9	0.0027
2	44.0112	15457	276.0	12195	27.6	0.0028
3	61.0374	16750	295.4	13053	29.6	0.0036
4	70.0625	18046	154.6	6830	15.5	0.0039
5	181.9555	27314	158.5	7001	15.9	0.0067
6	203.0457	29124	119.5	5278	12.0	0.0070
7	219.0191	30062	215.0	9500	21.5	0.0073
8	365.0927	31899	219.5	9697	22.0	0.0114
9	381.0664	31927	999.3	44151	100.0	0.0119
10	382.0697	30244	144.0	6362	14.4	0.0126

Compound Spectrum List Report

Cmpd 4, Dissect, 1.6 min

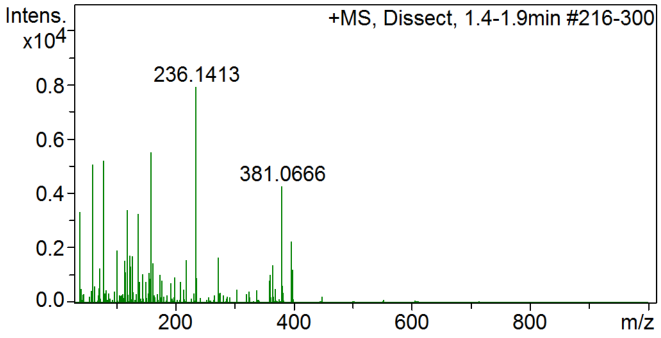

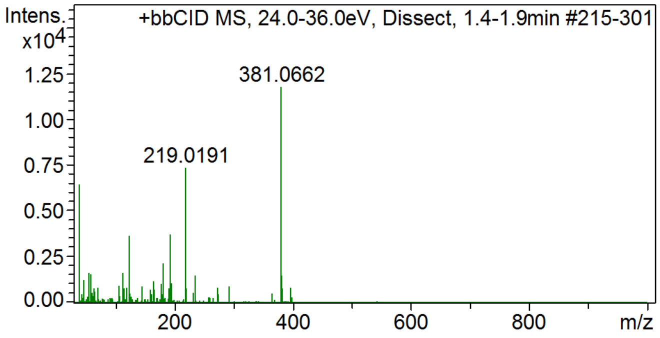

#	m/z	Res.	S/N	I	I %	FWHM
1	38.9614	14569	421.6	3341	42.2	0.0027
2	61.0373	16972	639.1	5065	63.9	0.0036
3	79.0184	18648	657.5	5210	65.8	0.0042
4	102.1244	20303	244.4	1937	24.5	0.0050
5	119.0563	23274	430.5	3411	43.1	0.0051
6	137.0762	25336	412.2	3267	41.2	0.0054
7	159.0574	26246	694.9	5507	69.5	0.0061
8	236.1413	31109	999.7	7922	100.0	0.0076
9	381.0666	32086	539.4	4275	54.0	0.0119
10	397.2578	32707	285.9	2266	28.6	0.0121

#	m/z	Res.	S/N	I	I %	FWHM
1	38.9614	14659	549.6	6462	55.0	0.0027
2	55.0520	17017	141.2	1660	14.1	0.0032
3	58.0629	17261	135.4	1591	13.5	0.0034
4	112.8917	23355	139.4	1639	13.9	0.0048
5	122.9203	24191	313.2	3682	31.3	0.0051
6	181.9553	28314	183.6	2158	18.4	0.0064
7	193.9913	29632	319.3	3754	31.9	0.0065
8	219.0191	29662	625.4	7353	62.5	0.0074
9	381.0662	31938	999.8	11755	100.0	0.0119
10	382.0699	30552	129.0	1516	12.9	0.0125

Cmpd 5, Dissect, 1.7 min

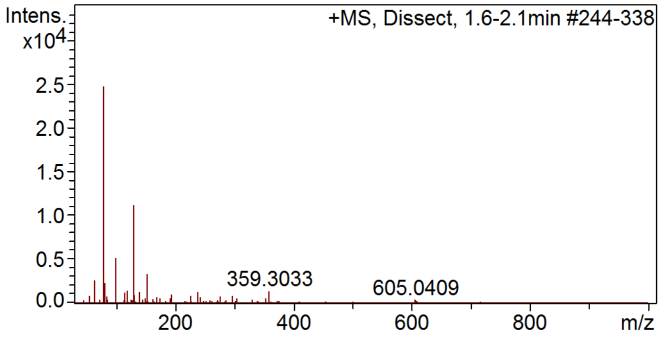

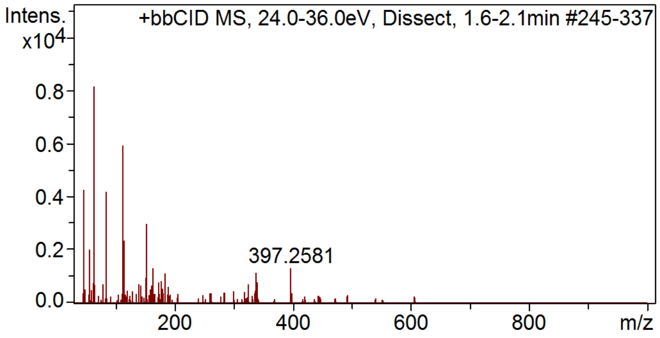

#	m/z	Res.	S/N	I	I %	FWHM
1	63.9955	17360	107.3	2660	10.7	0.0037
2	79.0184	18763	998.5	24753	100.0	0.0042
3	81.0141	19754	95.0	2356	9.5	0.0041
4	100.1089	20782	209.6	5195	21.0	0.0048
5	119.0564	23301	59.2	1467	5.9	0.0051
6	130.0455	25188	451.1	11182	45.2	0.0052
7	140.0882	25225	54.9	1361	5.5	0.0056
8	152.0266	27348	135.6	3361	13.6	0.0056
9	239.1413	29723	54.0	1338	5.4	0.0080
10	359.3033	31514	57.6	1427	5.8	0.0114

#	m/z	Res.	S/N	I	I %	FWHM
1	46.9930	15903	522.7	4263	52.3	0.0030
2	56.0472	16719	248.1	2024	24.8	0.0034
3	63.9955	16934	999.8	8154	100.0	0.0038
4	84.0415	19297	514.9	4199	51.5	0.0044
5	84.0778	20179	196.3	1601	19.6	0.0042
6	112.8917	23116	727.5	5933	72.8	0.0049
7	114.8892	22666	289.8	2363	29.0	0.0051
8	152.0266	26635	367.1	2994	36.7	0.0057
9	164.9149	28177	164.7	1344	16.5	0.0059
10	397.2581	31570	163.9	1336	16.4	0.0126

Compound Spectrum List Report

Cmpd 6, Dissect, 1.8 min

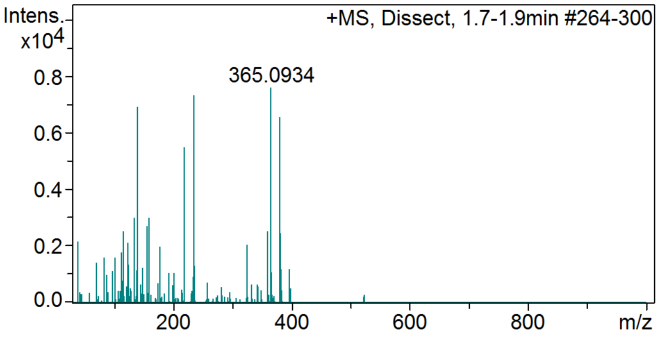

#	m/z	Res.	S/N	I	I %	FWHM
1	116.0666	23560	334.7	2546	33.5	0.0049
2	134.0403	24964	395.9	3012	39.7	0.0054
3	139.0455	25749	907.2	6902	90.9	0.0054
4	156.0217	26404	337.3	2566	33.8	0.0059
5	156.1329	27787	357.0	2716	35.8	0.0056
6	159.0573	26939	394.4	3000	39.5	0.0059
7	219.0194	30375	721.6	5489	72.3	0.0072
8	236.1414	30766	963.4	7329	96.5	0.0077
9	365.0934	34236	998.3	7594	100.0	0.0107
10	381.0663	33740	859.5	6539	86.1	0.0113

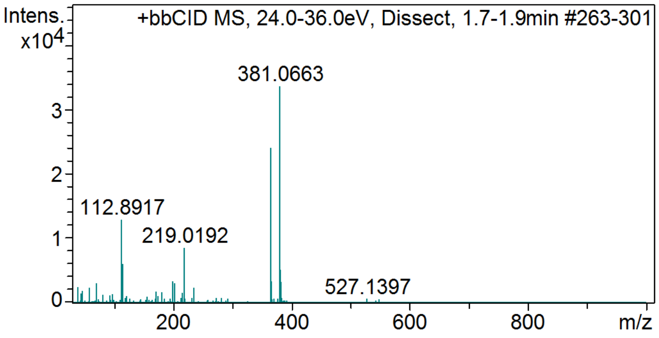

#	m/z	Res.	S/N	I	I %	FWHM
1	112.8917	23098	384.6	12969	38.5	0.0049
2	114.8892	22748	182.1	6141	18.3	0.0051
3	199.9838	28810	100.7	3396	10.1	0.0069
4	203.0458	29283	94.1	3172	9.4	0.0069
5	219.0192	30333	253.2	8537	25.4	0.0072
6	365.0928	32826	713.3	24050	71.5	0.0111
7	366.0966	30812	100.2	3378	10.0	0.0119
8	381.0663	31480	997.9	33648	100.0	0.0121
9	382.0695	30679	155.7	5248	15.6	0.0125
10	383.0665	28707	98.3	3315	9.9	0.0133

Cmpd 7, Dissect, 1.9 min

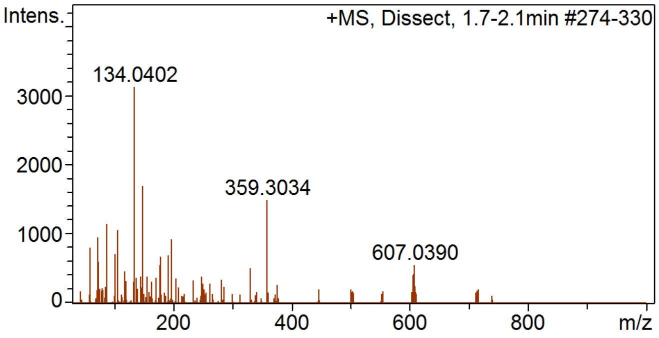

#	m/z	Res.	S/N	I	I %	FWHM
1	60.0422	16421	259.5	811	26.0	0.0037
2	72.0782	17983	305.1	954	30.5	0.0040
3	88.0363	19115	368.5	1152	36.9	0.0046
4	102.0513	21714	228.4	714	22.8	0.0047
5	106.0462	21825	336.9	1054	33.7	0.0049
6	134.0402	24253	999.8	3126	100.0	0.0055
7	148.0555	26234	542.4	1696	54.3	0.0056
8	193.0640	27836	221.8	694	22.2	0.0069
9	198.0906	28143	296.9	928	29.7	0.0070
10	359.3034	31374	477.1	1492	47.7	0.0115

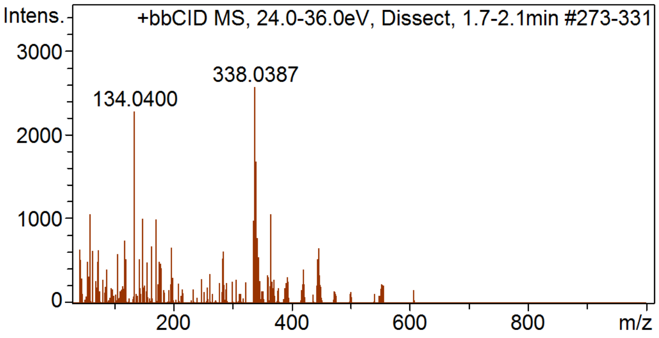

#	m/z	Res.	S/N	I	I %	FWHM
1	60.0421	16856	412.5	1062	41.3	0.0036
2	118.0824	24778	289.5	745	29.0	0.0048
3	134.0400	25504	884.3	2277	88.5	0.0053
4	148.0553	25258	390.2	1005	39.1	0.0059
5	171.9947	30255	386.8	996	38.7	0.0057
6	336.0415	31092	381.2	982	38.2	0.0108
7	338.0387	34198	999.1	2573	100.0	0.0099
8	340.0362	30150	653.7	1683	65.4	0.0113
9	342.9939	31402	303.7	782	30.4	0.0109
10	365.0931	33664	412.3	1062	41.3	0.0108

Compound Spectrum List Report

Cmpd 8, Dissect, 2.0 min

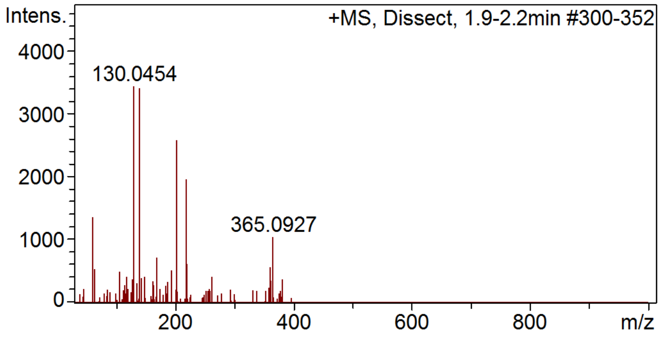

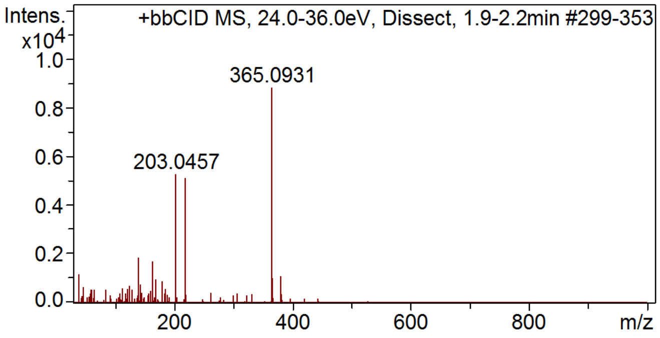

#	m/z	Res.	S/N	I	I %	FWHM
1	61.0085	17509	173.8	598	17.4	0.0035
2	61.0374	16954	396.4	1365	39.7	0.0036
3	130.0454	25049	997.6	3435	100.0	0.0052
4	139.0455	26040	988.9	3405	99.1	0.0053
5	170.0366	27712	210.6	725	21.1	0.0061
6	203.0459	28689	748.1	2576	75.0	0.0071
7	219.0191	28936	570.7	1965	57.2	0.0076
8	221.1095	30033	180.7	622	18.1	0.0074
9	361.2099	32986	167.2	576	16.8	0.0110
10	365.0927	32114	304.7	1049	30.5	0.0114

#	m/z	Res.	S/N	I	I %	FWHM
1	38.9615	14616	134.6	1190	13.5	0.0027
2	139.0452	26133	178.8	1580	17.9	0.0053
3	139.9099	26370	212.8	1881	21.3	0.0053
4	164.9149	27192	193.3	1708	19.4	0.0061
5	170.0365	27156	109.9	971	11.0	0.0063
6	203.0457	28441	597.5	5280	59.8	0.0071
7	219.0191	29753	580.0	5126	58.1	0.0074
8	365.0931	33780	998.4	8824	100.0	0.0108
9	366.0964	32682	117.5	1038	11.8	0.0112
10	381.0662	30201	125.7	1111	12.6	0.0126

Cmpd 9, Dissect, 2.0 min

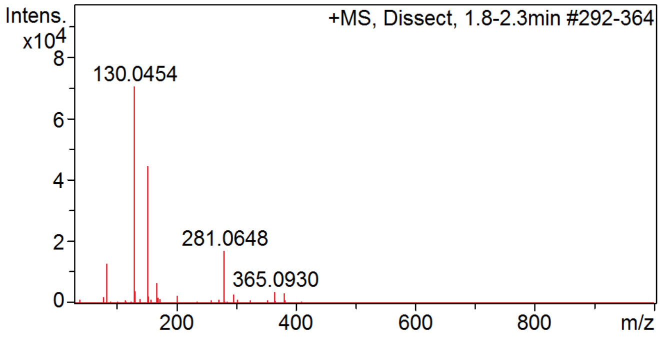

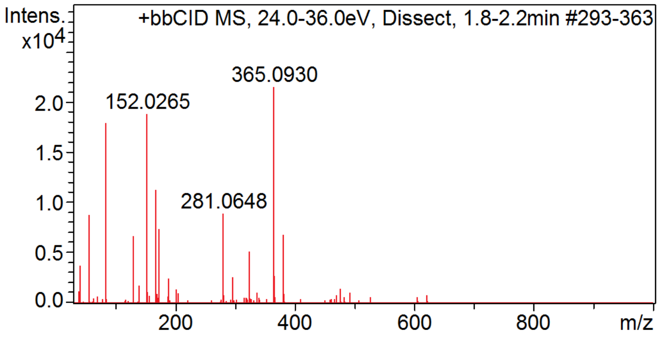

#	m/z	Res.	S/N	I	I %	FWHM
1	84.0416	19264	182.8	12905	18.3	0.0044
2	130.0454	25170	997.7	70440	100.0	0.0052
3	131.0489	23752	56.4	3983	5.7	0.0055
4	152.0265	27242	631.5	44587	63.3	0.0056
5	167.9999	27207	96.0	6779	9.6	0.0062
6	203.0456	28849	32.9	2322	3.3	0.0070
7	281.0648	32025	243.9	17224	24.5	0.0088
8	297.0378	29818	40.2	2836	4.0	0.0100
9	365.0930	31653	53.1	3746	5.3	0.0115
10	381.2842	30603	47.3	3338	4.7	0.0125

#	m/z	Res.	S/N	I	I %	FWHM
1	56.0471	17094	407.9	8780	40.8	0.0033
2	84.0415	19526	830.7	17880	83.1	0.0043
3	130.0455	24550	312.8	6732	31.3	0.0053
4	152.0265	26686	870.7	18742	87.1	0.0057
5	168.0000	27627	522.9	11256	52.3	0.0061
6	174.0077	27660	342.7	7377	34.3	0.0063
7	281.0648	31556	414.7	8926	41.5	0.0089
8	325.0277	32970	241.5	5198	24.2	0.0099
9	365.0930	33203	999.6	21516	100.0	0.0110
10	381.0664	30450	318.4	6854	31.9	0.0125

Compound Spectrum List Report

Cmpd 10, Dissect, 2.2 min

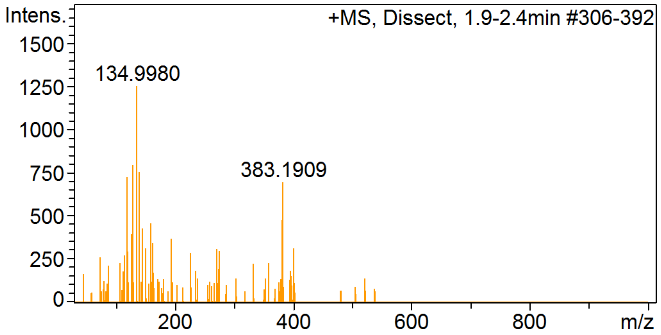

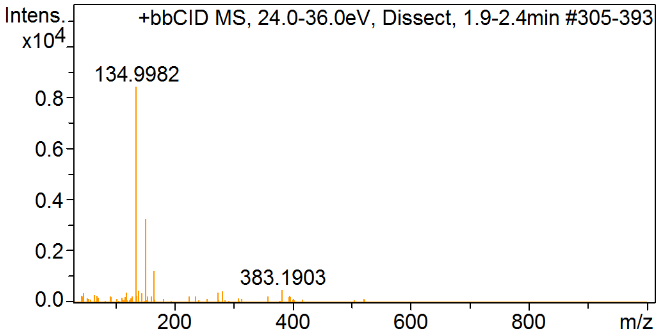

#	m/z	Res.	S/N	I	I %	FWHM
1	119.0563	23697	577.7	726	57.9	0.0050
2	127.0701	23595	316.7	398	31.8	0.0054
3	129.0479	25037	632.0	794	63.4	0.0052
4	134.9980	25850	997.3	1253	100.0	0.0052
5	140.0883	26127	599.6	753	60.1	0.0054
6	145.0597	25990	342.0	430	34.3	0.0056
7	159.0573	26041	364.7	458	36.6	0.0061
8	195.1161	29175	296.1	372	29.7	0.0067
9	381.2840	32586	379.1	476	38.0	0.0117
10	383.1909	29982	554.1	696	55.6	0.0128

#	m/z	Res.	S/N	I	I %	FWHM
1	46.9931	15996	46.0	389	4.6	0.0029
2	119.0563	23606	49.5	418	5.0	0.0050
3	134.9982	25743	998.4	8433	100.0	0.0052
4	139.9099	25472	58.7	496	5.9	0.0055
5	150.9715	26869	386.1	3261	38.7	0.0056
6	166.9450	26877	148.3	1253	14.9	0.0062
7	274.9556	35781	50.1	423	5.0	0.0077
8	275.0426	37247	43.1	364	4.3	0.0074
9	282.0393	30587	53.1	448	5.3	0.0092
10	383.1903	33726	61.6	520	6.2	0.0114

Cmpd 11, Dissect, 2.3 min

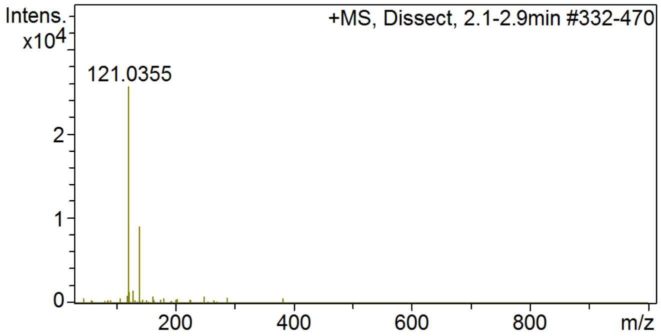

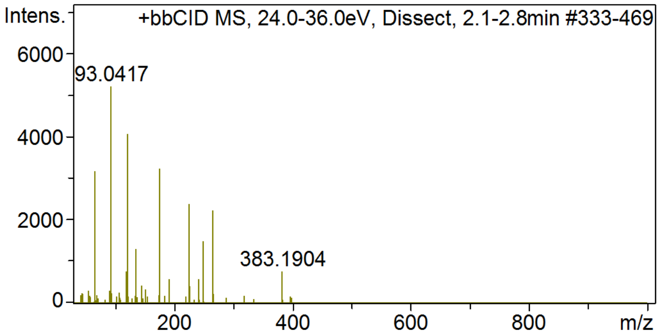

#	m/z	Res.	S/N	I	I %	FWHM
1	107.0669	21791	22.9	590	2.3	0.0049
2	119.0563	23663	32.3	831	3.2	0.0050
3	121.0355	24177	995.5	25630	100.0	0.0050
4	122.0386	22954	55.0	1415	5.5	0.0053
5	129.0480	24201	61.6	1585	6.2	0.0053
6	139.0454	25659	352.1	9066	35.4	0.0054
7	163.1174	26862	29.6	763	3.0	0.0061
8	182.0751	27621	22.3	575	2.2	0.0066
9	250.0713	29823	29.7	764	3.0	0.0084
10	288.9124	29919	24.2	624	2.4	0.0097

#	m/z	Res.	S/N	I	I %	FWHM
1	66.0316	17219	606.9	3162	60.7	0.0038
2	93.0417	19866	999.3	5206	100.0	0.0047
3	119.0563	23268	148.3	773	14.8	0.0051
4	121.0355	23496	780.4	4066	78.1	0.0052
5	134.9982	25412	251.6	1311	25.2	0.0053
6	176.0598	27889	621.3	3237	62.2	0.0063
7	226.0374	29933	457.8	2385	45.8	0.0076
8	250.0712	30799	286.9	1495	28.7	0.0081
9	266.0446	30890	429.0	2235	42.9	0.0086
10	383.1904	31626	149.5	779	15.0	0.0121

Compound Spectrum List Report

Cmpd 12, Dissect, 2.6 min

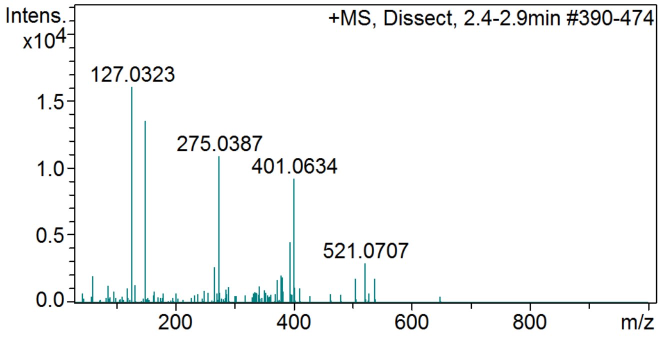

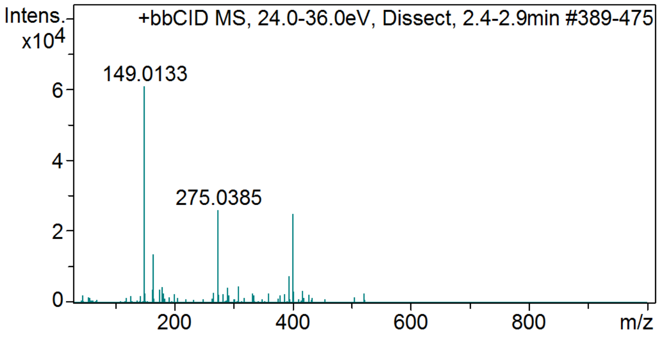

#	m/z	Res.	S/N	I	I %	FWHM
1	61.0084	16533	126.2	2025	12.6	0.0037
2	127.0323	23754	999.4	16040	100.0	0.0053
3	149.0135	25623	840.9	13496	84.1	0.0058
4	267.0498	28207	166.8	2677	16.7	0.0095
5	275.0387	30126	677.1	10868	67.8	0.0091
6	379.0698	33252	129.8	2084	13.0	0.0114
7	381.2844	30855	119.8	1923	12.0	0.0124
8	395.0462	30578	280.7	4506	28.1	0.0129
9	401.0634	31221	574.2	9215	57.5	0.0128
10	521.0707	32155	184.2	2957	18.4	0.0162

#	m/z	Res.	S/N	I	I %	FWHM
1	149.0133	26559	998.3	60840	100.0	0.0056
2	164.9145	27020	63.3	3858	6.3	0.0061
3	164.9867	26903	224.2	13664	22.5	0.0061
4	176.0597	27322	62.2	3793	6.2	0.0064
5	180.9602	27883	74.4	4534	7.5	0.0065
6	275.0385	30817	429.5	26176	43.0	0.0089
7	291.0111	24543	70.8	4316	7.1	0.0119
8	309.0127	30665	78.6	4791	7.9	0.0101
9	395.0462	31373	125.7	7660	12.6	0.0126
10	401.0630	31145	411.5	25077	41.2	0.0129

Cmpd 13, Dissect, 2.9 min

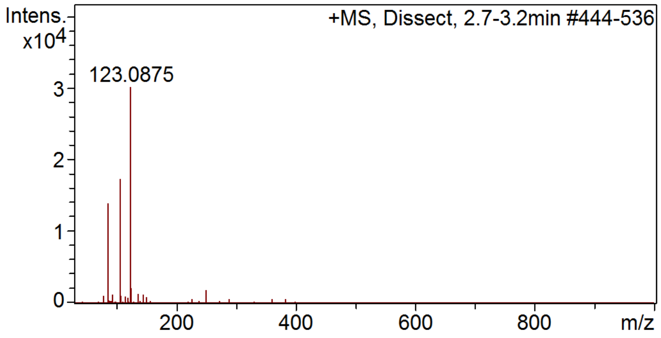

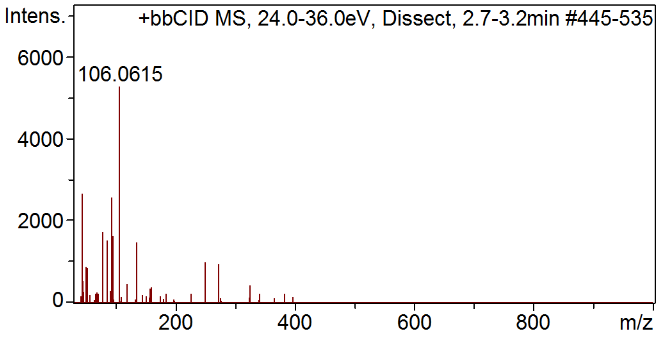

#	m/z	Res.	S/N	I	I %	FWHM
1	79.0184	18413	33.4	1010	3.3	0.0043
2	86.0570	19579	460.9	13934	46.2	0.0044
3	94.0622	20807	41.1	1242	4.1	0.0045
4	106.0615	22099	572.2	17298	57.3	0.0048
5	107.0657	19840	34.8	1052	3.5	0.0054
6	123.0875	24064	998.5	30186	100.0	0.0051
7	124.0909	22680	72.6	2194	7.3	0.0055
8	136.0573	24656	44.0	1329	4.4	0.0055
9	145.0597	26338	40.9	1236	4.1	0.0055
10	251.0553	29244	63.0	1904	6.3	0.0086

#	m/z	Res.	S/N	I	I %	FWHM
1	44.0112	15488	502.9	2660	50.5	0.0028
2	44.0476	15423	188.1	995	18.9	0.0029
3	78.0309	17393	327.5	1732	32.9	0.0045
4	86.0570	20138	289.7	1532	29.1	0.0043
5	94.0622	19661	486.1	2571	48.8	0.0048
6	96.0411	19910	309.0	1634	31.0	0.0048
7	106.0615	21368	996.3	5270	100.0	0.0050
8	134.9981	25815	280.2	1482	28.1	0.0052
9	251.0552	31153	189.7	1004	19.0	0.0081
10	273.0363	32130	180.7	956	18.1	0.0085

Compound Spectrum List Report

Cmpd 14, Dissect, 3.1 min

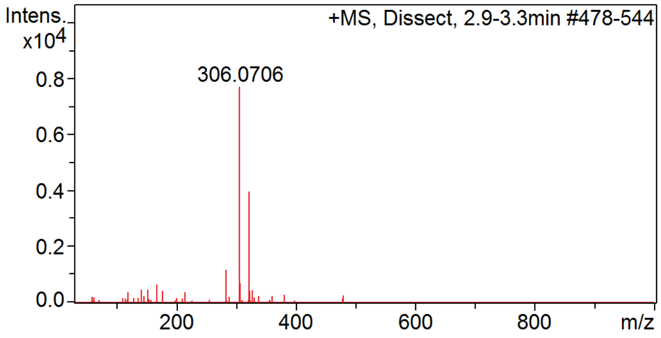

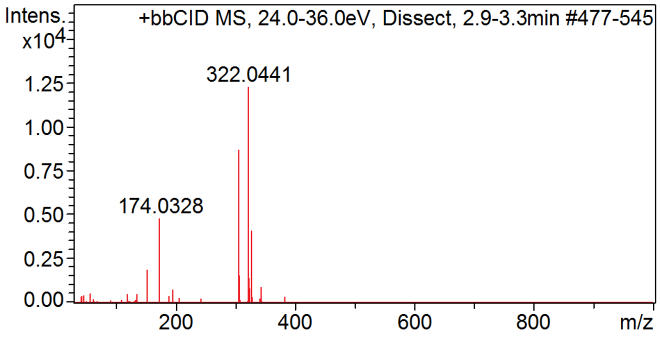

#	m/z	Res.	S/N	I	I %	FWHM
1	142.1176	25625	64.5	498	6.5	0.0055
2	152.0514	26217	62.7	483	6.3	0.0058
3	168.0577	26993	88.0	679	8.8	0.0062
4	284.0896	28740	154.5	1191	15.5	0.0099
5	306.0706	31330	999.9	7709	100.0	0.0098
6	307.0338	34163	68.4	527	6.8	0.0090
7	307.0735	26609	94.0	725	9.4	0.0115
8	322.0436	30474	514.6	3967	51.5	0.0106
9	323.0464	30482	58.3	450	5.8	0.0106
10	328.0518	32032	60.6	467	6.1	0.0102

#	m/z	Res.	S/N	I	I %	FWHM
1	152.0512	26693	153.8	1890	15.4	0.0057
2	174.0328	27742	389.6	4790	39.0	0.0063
3	306.0704	31653	705.5	8674	70.7	0.0097
4	307.0336	31815	86.5	1064	8.7	0.0097
5	307.0729	30223	126.8	1560	12.7	0.0102
6	322.0441	31537	998.5	12277	100.0	0.0102
7	323.0470	28485	115.7	1423	11.6	0.0113
8	324.0450	26787	68.4	841	6.8	0.0121
9	328.0514	33597	335.7	4128	33.6	0.0098
10	344.0256	34435	75.1	924	7.5	0.0100

Cmpd 15, Dissect, 3.1 min

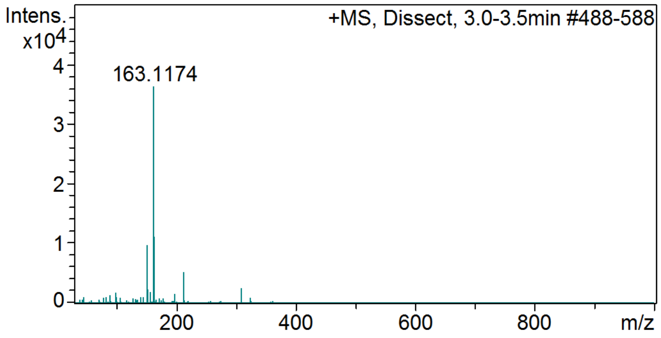

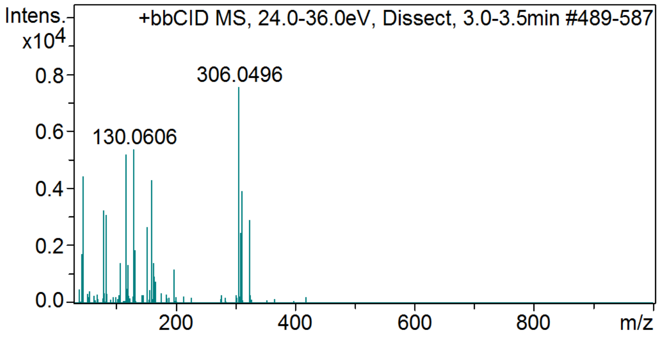

#	m/z	Res.	S/N	I	I %	FWHM
1	99.0411	20040	51.2	1862	5.1	0.0049
2	151.0914	25663	269.4	9790	26.9	0.0059
3	152.0514	27067	67.7	2460	6.8	0.0056
4	157.0782	26079	54.9	1995	5.5	0.0060
5	163.1174	27567	999.9	36342	100.0	0.0059
6	164.1014	27013	307.8	11187	30.8	0.0061
7	164.1206	26094	114.7	4170	11.5	0.0063
8	198.1037	28993	43.8	1593	4.4	0.0068
9	213.0951	28686	144.9	5266	14.5	0.0074
10	309.0841	31266	71.3	2590	7.1	0.0099

#	m/z	Res.	S/N	I	I %	FWHM
1	45.0315	15842	585.0	4425	58.5	0.0028
2	80.0467	19011	428.0	3237	42.8	0.0042
3	84.0778	19114	409.4	3097	41.0	0.0044
4	117.0533	23365	685.7	5187	68.6	0.0050
5	130.0606	24493	709.4	5366	71.0	0.0053
6	161.0129	27477	568.0	4297	56.8	0.0059
7	306.0496	30277	999.6	7561	100.0	0.0101
8	306.0706	31834	411.5	3113	41.2	0.0096
9	312.0575	31514	517.5	3914	51.8	0.0099
10	325.0576	28994	385.6	2916	38.6	0.0112

Compound Spectrum List Report

Cmpd 16, Dissect, 3.3 min

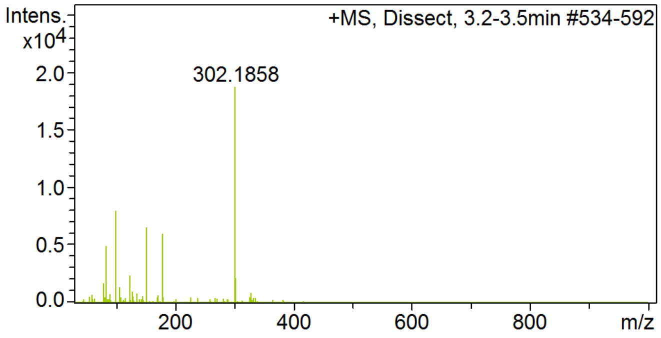

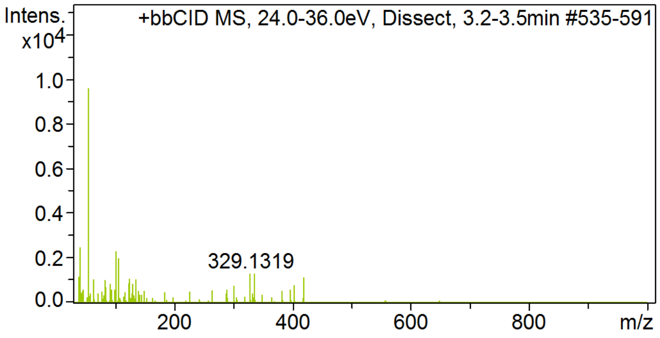

#	m/z	Res.	S/N	I	I %	FWHM
1	79.0184	18490	92.8	1743	9.3	0.0043
2	83.0826	18913	262.8	4937	26.4	0.0044
3	100.1088	20439	427.1	8023	42.8	0.0049
4	106.0616	22310	76.4	1434	7.7	0.0048
5	123.0875	23661	129.3	2430	13.0	0.0052
6	151.0815	26018	349.5	6565	35.1	0.0058
7	151.0907	27514	75.3	1415	7.6	0.0055
8	179.1116	27802	318.8	5988	32.0	0.0064
9	302.1858	32113	996.8	18725	100.0	0.0094
10	303.1892	29179	117.3	2203	11.8	0.0104

#	m/z	Res.	S/N	I	I %	FWHM
1	38.9615	14684	123.3	1186	12.3	0.0027
2	41.0368	15166	258.4	2485	25.8	0.0027
3	55.0520	17031	999.9	9618	100.0	0.0032
4	102.0879	21319	243.0	2337	24.3	0.0048
5	106.0616	22015	209.2	2013	20.9	0.0048
6	123.9364	24784	114.1	1098	11.4	0.0050
7	134.9982	25299	112.6	1083	11.3	0.0053
8	329.1319	36348	139.2	1339	13.9	0.0091
9	336.0603	31507	136.8	1316	13.7	0.0107
10	419.1037	33791	119.5	1149	12.0	0.0124

Cmpd 17, Dissect, 3.4 min

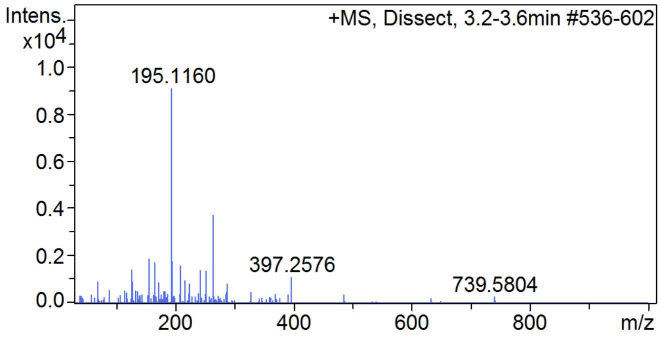

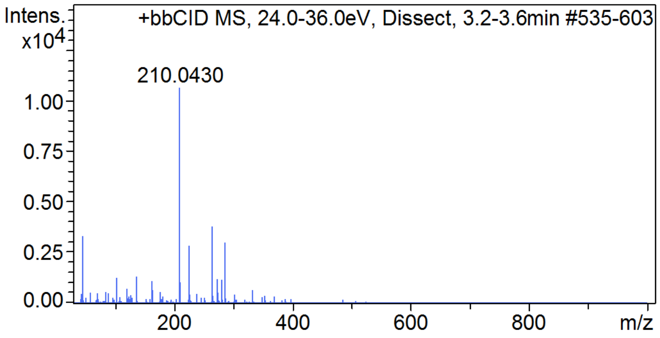

#	m/z	Res.	S/N	I	I %	FWHM
1	127.0456	24192	159.8	1456	16.0	0.0053
2	156.1328	26963	208.8	1903	20.9	0.0058
3	166.0803	26845	191.6	1746	19.2	0.0062
4	195.1160	28912	999.4	9109	100.0	0.0067
5	196.1238	24116	197.0	1796	19.7	0.0081
6	210.0428	28446	177.2	1615	17.7	0.0074
7	243.1750	32334	155.8	1420	15.6	0.0075
8	253.1198	29507	153.3	1397	15.3	0.0086
9	265.0705	30156	411.5	3750	41.2	0.0088
10	397.2576	31832	123.3	1124	12.3	0.0125

#	m/z	Res.	S/N	I	I %	FWHM
1	45.0316	15810	311.5	3317	31.2	0.0028
2	103.0508	22008	119.1	1268	11.9	0.0047
3	136.0571	25109	126.3	1345	12.6	0.0054
4	163.0286	24545	105.2	1121	10.5	0.0066
5	210.0430	29400	999.7	10648	100.0	0.0071
6	226.0165	29584	267.0	2844	26.7	0.0076
7	265.0702	30329	355.6	3788	35.6	0.0087
8	274.0818	31931	113.7	1211	11.4	0.0086
9	281.0439	31744	111.3	1186	11.1	0.0089
10	287.0515	31181	283.3	3017	28.3	0.0092

Compound Spectrum List Report

Cmpd 18, Dissect, 3.4 min

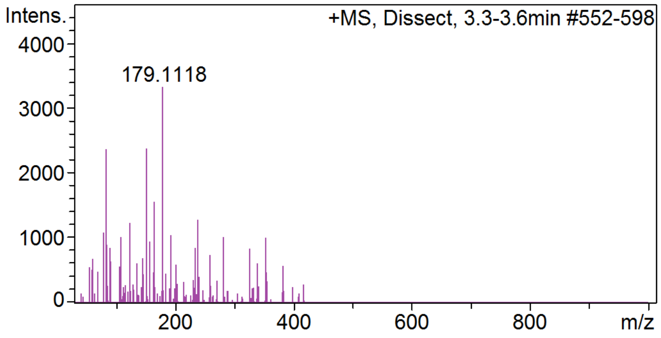

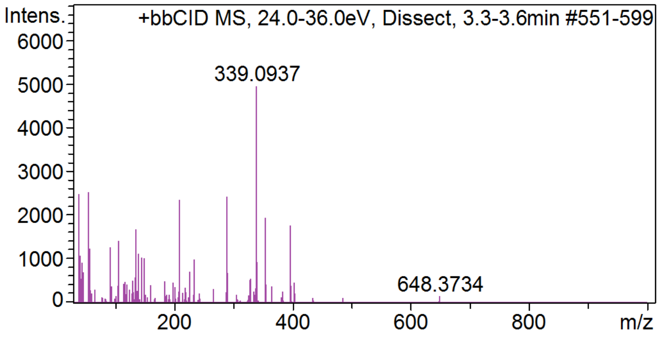

#	m/z	Res.	S/N	I	I %	FWHM
1	79.0184	18635	325.8	1086	32.6	0.0042
2	83.0825	19322	708.2	2361	70.9	0.0043
3	107.9631	23560	304.6	1015	30.5	0.0046
4	123.0874	23945	369.4	1231	37.0	0.0051
5	151.0812	25449	638.7	2129	63.9	0.0059
6	151.0912	25917	710.8	2370	71.1	0.0058
7	165.0967	26416	466.7	1556	46.7	0.0062
8	179.1118	25666	999.4	3332	100.0	0.0070
9	193.1271	25347	314.5	1048	31.5	0.0076
10	239.1409	31143	385.6	1286	38.6	0.0077

#	m/z	Res.	S/N	I	I %	FWHM
1	38.9614	14817	503.1	2491	50.3	0.0026
2	55.0520	16754	510.4	2527	51.0	0.0033
3	92.0463	20021	258.6	1280	25.9	0.0046
4	106.0616	21492	287.8	1425	28.8	0.0049
5	134.9980	25072	341.2	1689	34.1	0.0054
6	210.0431	29131	476.0	2356	47.6	0.0072
7	290.0761	31272	491.9	2435	49.2	0.0093
8	339.0937	32308	1000.0	4951	100.0	0.0105
9	355.0669	28892	393.7	1949	39.4	0.0123
10	397.2576	31887	357.8	1772	35.8	0.0125

Cmpd 19, Dissect, 3.5 min

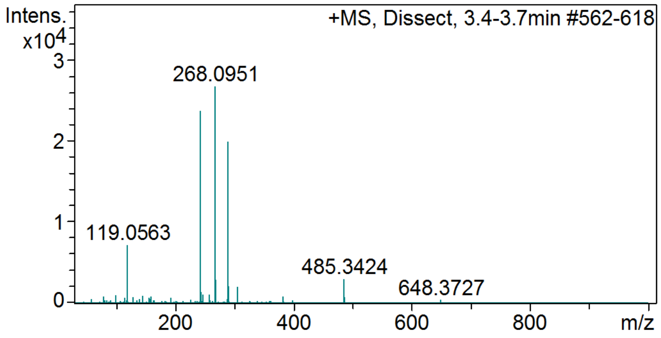

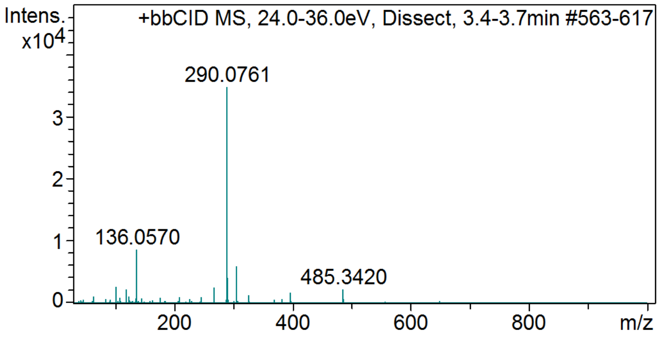

#	m/z	Res.	S/N	I	I %	FWHM
1	119.0563	23533	270.3	7237	27.1	0.0051
2	243.1750	33149	883.5	23654	88.5	0.0073
3	243.6766	31994	268.0	7176	26.8	0.0076
4	244.1783	29918	54.7	1465	5.5	0.0082
5	268.0951	32362	998.4	26730	100.0	0.0083
6	269.0980	26681	110.0	2945	11.0	0.0101
7	290.0763	32318	741.8	19861	74.3	0.0090
8	291.0790	27392	80.1	2144	8.0	0.0106
9	306.0497	31130	78.6	2104	7.9	0.0098
10	485.3424	33895	112.8	3020	11.3	0.0143

#	m/z	Res.	S/N	I	I %	FWHM
1	102.0879	20106	78.7	2775	8.0	0.0051
2	119.0564	23168	67.0	2362	6.8	0.0051
3	136.0570	25593	244.9	8638	24.8	0.0053
4	268.0951	29400	75.6	2667	7.7	0.0091
5	290.0761	31782	987.7	34844	100.0	0.0091
6	291.0791	26936	117.3	4137	11.9	0.0108
7	306.0495	30771	169.6	5982	17.2	0.0099
8	326.3668	32434	36.2	1277	3.7	0.0101
9	397.2577	30766	50.2	1770	5.1	0.0129
10	485.3420	30583	66.0	2328	6.7	0.0159

Compound Spectrum List Report

Cmpd 20, Dissect, 3.6 min

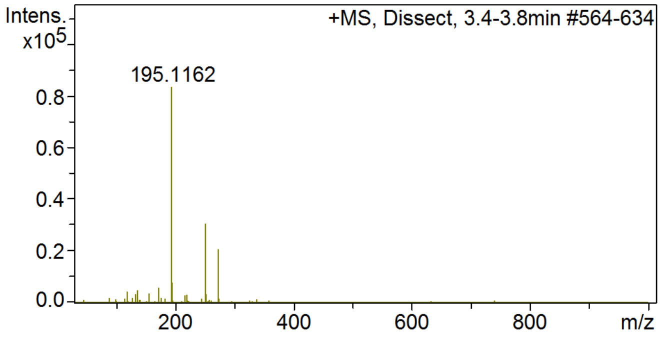

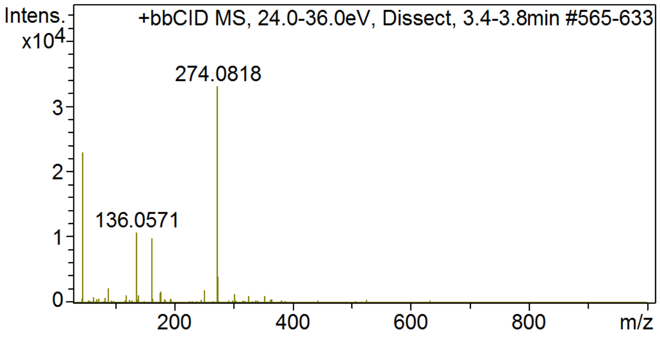

#	m/z	Res.	S/N	I	I %	FWHM
1	119.0564	23503	55.4	4634	5.5	0.0051
2	133.0813	24072	39.4	3297	3.9	0.0055
3	136.0572	24877	61.5	5139	6.2	0.0055
4	156.0965	26360	41.6	3476	4.2	0.0059
5	173.1227	26025	71.9	6013	7.2	0.0067
6	195.1162	29548	999.1	83536	100.0	0.0066
7	196.1195	29089	94.9	7933	9.5	0.0067
8	252.1008	31884	367.2	30703	36.8	0.0079
9	253.1037	24188	39.9	3335	4.0	0.0105
10	274.0819	32023	248.4	20766	24.9	0.0086

#	m/z	Res.	S/N	I	I %	FWHM
1	45.0316	15695	694.0	22933	69.4	0.0029
2	89.0569	18481	68.7	2271	6.9	0.0048
3	136.0571	25265	325.5	10757	32.6	0.0054
4	163.0285	27299	298.6	9867	29.9	0.0060
5	177.0070	28636	49.5	1636	5.0	0.0062
6	179.0020	26691	53.6	1772	5.4	0.0067
7	252.1003	28941	60.6	2003	6.1	0.0087
8	274.0818	31998	999.7	33036	100.0	0.0086
9	275.0849	27848	121.2	4004	12.1	0.0099
10	303.0689	31284	38.6	1277	3.9	0.0097

Cmpd 21, Dissect, 3.7 min

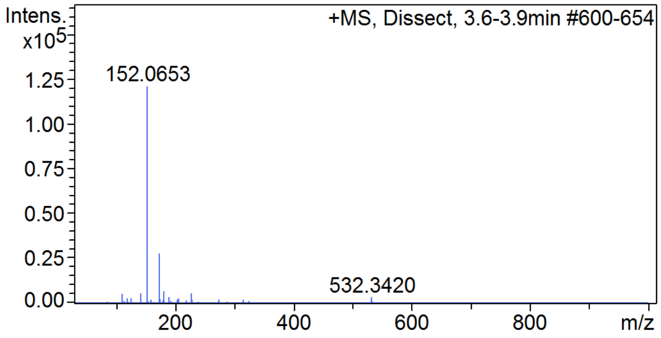

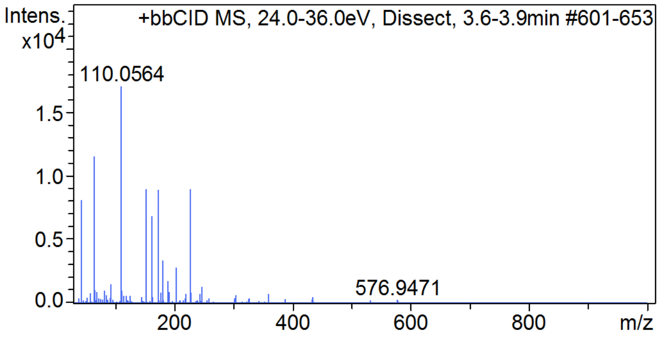

#	m/z	Res.	S/N	I	I %	FWHM
1	110.0562	22999	42.8	5169	4.3	0.0048
2	119.0563	23537	23.5	2838	2.3	0.0051
3	142.0814	25642	47.7	5771	4.8	0.0055
4	152.0653	27129	999.1	120795	100.0	0.0056
5	153.0686	25685	59.5	7194	6.0	0.0060
6	174.0466	28378	230.6	27876	23.1	0.0061
7	182.0724	27253	58.4	7066	5.8	0.0067
8	190.0198	27130	27.2	3286	2.7	0.0070
9	228.1129	28657	49.7	6003	5.0	0.0080
10	532.3420	31372	26.6	3215	2.7	0.0170

#	m/z	Res.	S/N	I	I %	FWHM
1	43.0160	15300	475.7	8107	47.6	0.0028
2	43.0525	15386	116.7	1989	11.7	0.0028
3	65.0363	17110	676.9	11535	67.7	0.0038
4	110.0564	21947	999.4	17031	100.0	0.0050
5	152.0653	26458	525.6	8957	52.6	0.0057
6	163.0286	27162	402.0	6851	40.2	0.0060
7	174.0467	27416	522.1	8898	52.2	0.0063
8	182.0727	27587	198.4	3380	19.8	0.0066
9	204.0537	29261	167.4	2853	16.8	0.0070
10	228.1132	29183	524.8	8944	52.5	0.0078

Compound Spectrum List Report

Cmpd 22, Dissect, 3.7 min

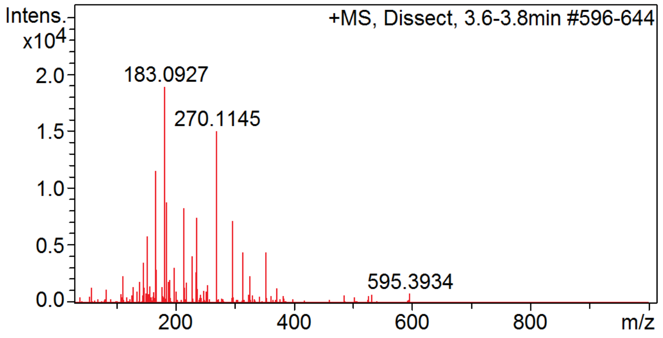

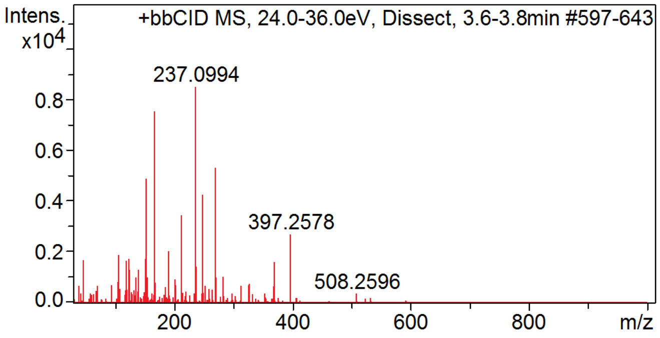

#	m/z	Res.	S/N	I	I %	FWHM
1	153.0687	25789	308.3	5841	30.9	0.0059
2	168.0944	25030	608.2	11522	60.9	0.0067
3	183.0927	28120	998.2	18912	100.0	0.0065
4	186.1063	27045	462.4	8761	46.3	0.0069
5	215.1175	28546	436.3	8266	43.7	0.0075
6	237.0993	29082	392.1	7429	39.3	0.0082
7	270.1145	29607	790.9	14985	79.2	0.0091
8	297.1449	30568	377.1	7145	37.8	0.0097
9	314.1705	30992	233.2	4419	23.4	0.0101
10	353.2542	32729	234.6	4446	23.5	0.0108

#	m/z	Res.	S/N	I	I %	FWHM
1	106.0616	21893	224.0	1906	22.4	0.0048
2	153.0833	24479	573.4	4881	57.4	0.0063
3	168.0939	27444	884.4	7528	88.5	0.0061
4	191.0227	28466	241.5	2056	24.2	0.0067
5	213.0039	27302	406.9	3464	40.7	0.0078
6	213.1014	25650	222.8	1896	22.3	0.0083
7	237.0994	29947	999.8	8511	100.0	0.0079
8	249.0525	27902	499.6	4252	50.0	0.0089
9	270.1151	28908	624.8	5318	62.5	0.0093
10	397.2578	31720	318.4	2710	31.8	0.0125

Cmpd 23, Dissect, 3.8 min

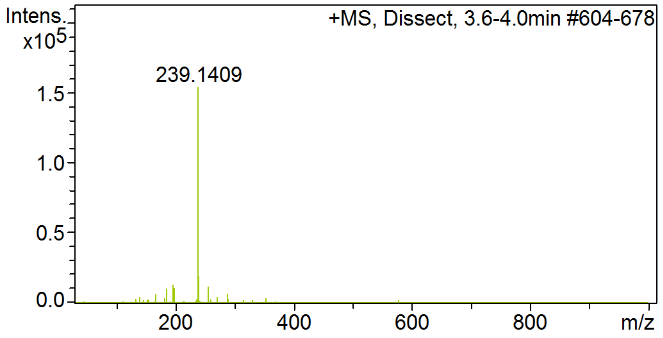

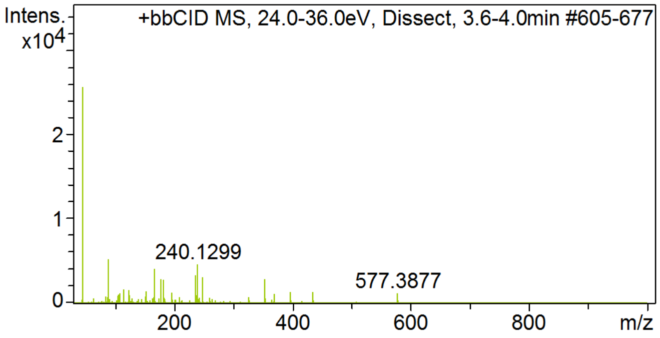

#	m/z	Res.	S/N	I	I %	FWHM
1	168.0945	24340	38.8	5981	3.9	0.0069
2	186.1062	27804	68.1	10507	6.8	0.0067
3	197.1581	28659	88.3	13633	8.9	0.0069
4	199.1161	28683	71.7	11060	7.2	0.0069
5	239.1409	29405	997.6	153966	100.0	0.0081
6	240.1301	28957	49.5	7641	5.0	0.0083
7	240.1440	28991	124.1	19152	12.4	0.0083
8	256.1667	30122	78.5	12118	7.9	0.0085
9	271.1711	29348	27.3	4209	2.7	0.0092
10	289.1972	32349	42.9	6617	4.3	0.0089

#	m/z	Res.	S/N	I	I %	FWHM
1	45.0316	15717	998.7	25675	100.0	0.0029
2	89.0568	18951	204.1	5248	20.4	0.0047
3	115.0826	21863	67.7	1740	6.8	0.0053
4	168.0939	27557	161.8	4159	16.2	0.0061
5	178.1167	27239	113.0	2905	11.3	0.0065
6	183.0975	24226	109.2	2807	10.9	0.0076
7	237.0993	30380	130.6	3357	13.1	0.0078
8	240.1299	28679	180.0	4628	18.0	0.0084
9	249.0527	28928	122.4	3148	12.3	0.0086
10	353.2542	33367	114.1	2934	11.4	0.0106

Compound Spectrum List Report

Cmpd 24, Dissect, 3.9 min

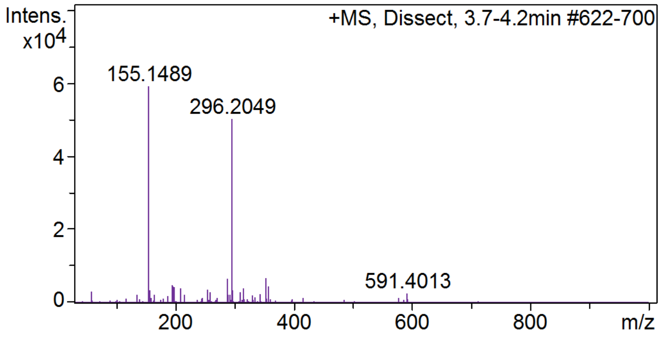

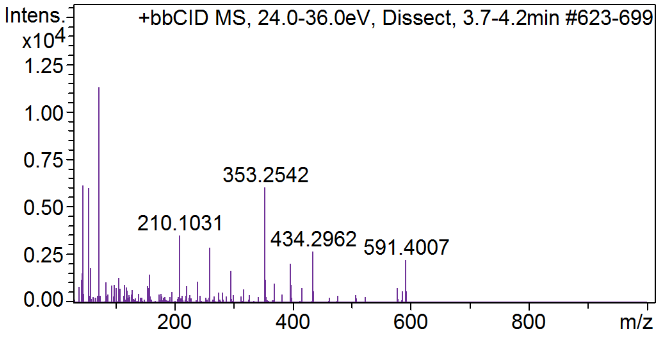

#	m/z	Res.	S/N	I	I %	FWHM
1	155.1489	26697	996.9	59191	100.0	0.0058
2	196.1627	28096	84.5	5016	8.5	0.0070
3	197.1581	27328	78.1	4640	7.8	0.0072
4	199.1163	28891	75.9	4505	7.6	0.0069
5	289.1976	33338	112.8	6697	11.3	0.0087
6	296.1295	31061	84.0	4989	8.4	0.0095
7	296.2049	34843	843.8	50101	84.6	0.0085
8	296.7065	35105	292.1	17341	29.3	0.0085
9	353.2542	32692	114.7	6810	11.5	0.0108
10	358.1951	30899	78.8	4678	7.9	0.0116

#	m/z	Res.	S/N	I	I %	FWHM
1	46.0631	16158	543.8	6152	54.4	0.0029
2	55.0520	17128	530.1	5997	53.0	0.0032
3	59.0467	17627	162.3	1836	16.2	0.0033
4	73.0733	18469	1000.0	11313	100.0	0.0040
5	210.1031	27175	313.6	3547	31.4	0.0077
6	260.0785	29494	256.7	2905	25.7	0.0088
7	353.2542	33925	533.6	6037	53.4	0.0104
8	397.2577	31634	182.5	2064	18.2	0.0126
9	434.2962	29373	239.3	2708	23.9	0.0148
10	591.4007	33245	198.6	2247	19.9	0.0178

Cmpd 25, Dissect, 4.0 min

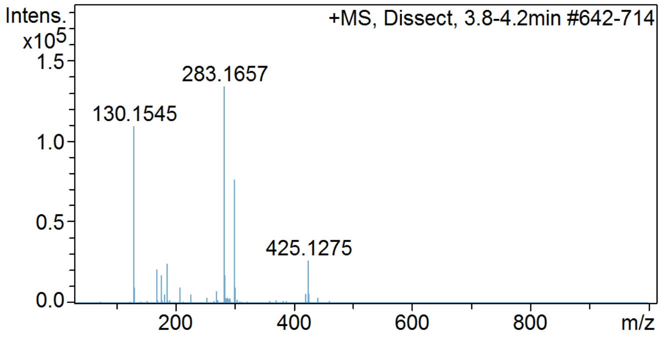

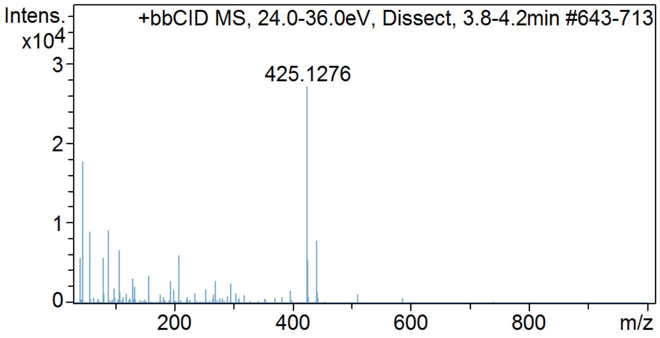

#	m/z	Res.	S/N	I	I %	FWHM
1	130.1545	24415	812.7	108985	81.3	0.0053
2	170.1117	27405	157.4	21106	15.8	0.0062
3	170.1480	27365	77.2	10353	7.7	0.0062
4	177.0961	27409	130.7	17532	13.1	0.0065
5	187.1376	28275	185.3	24844	18.5	0.0066
6	283.1657	31994	999.2	133998	100.0	0.0089
7	284.1688	30238	130.0	17433	13.0	0.0094
8	300.1916	31940	569.2	76333	57.0	0.0094
9	301.1950	29276	74.0	9927	7.4	0.0103
10	425.1275	32787	198.1	26571	19.8	0.0130

#	m/z	Res.	S/N	I	I %	FWHM
1	41.0368	14920	211.7	5754	21.2	0.0028
2	45.0315	15689	652.2	17727	65.3	0.0029
3	57.0676	17032	331.3	9005	33.2	0.0034
4	80.0467	19037	209.9	5705	21.0	0.0042
5	89.0567	18674	336.5	9146	33.7	0.0048
6	107.0455	22130	244.6	6647	24.5	0.0048
7	209.1190	29182	222.5	6048	22.3	0.0072
8	425.1276	31979	999.1	27155	100.0	0.0133
9	426.1307	30874	202.5	5503	20.3	0.0138
10	441.1003	33665	289.2	7859	28.9	0.0131

Compound Spectrum List Report

Cmpd 26, Dissect, 4.1 min

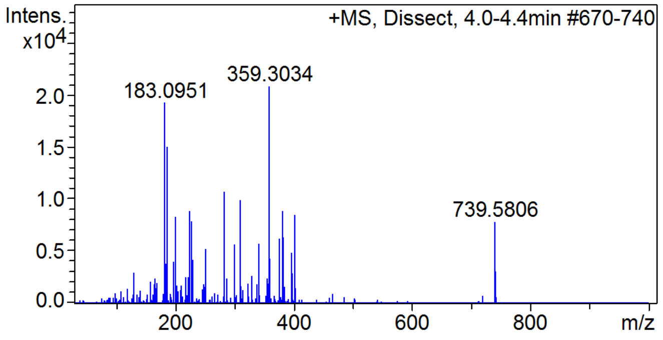

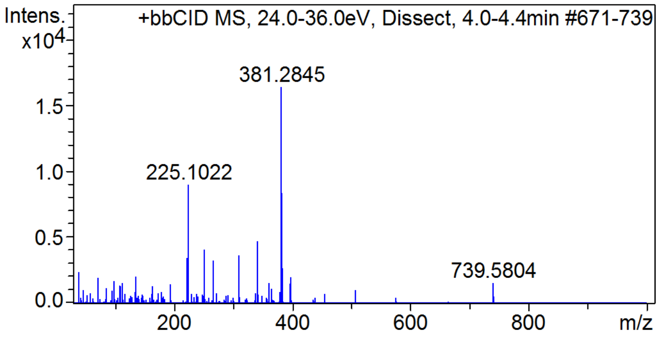

#	m/z	Res.	S/N	I	I %	FWHM
1	183.0951	26954	924.0	19250	92.4	0.0068
2	187.1377	28249	720.5	15010	72.1	0.0066
3	201.1164	27357	399.9	8332	40.0	0.0074
4	225.1022	28154	424.4	8841	42.5	0.0080
5	228.1155	27780	378.5	7887	37.9	0.0082
6	283.1657	31564	513.2	10692	51.3	0.0090
7	310.1448	32490	475.8	9913	47.6	0.0095
8	359.3034	34772	999.5	20823	100.0	0.0103
9	381.2845	33691	426.7	8891	42.7	0.0113
10	402.2193	31239	407.8	8497	40.8	0.0129

#	m/z	Res.	S/N	I	I %	FWHM
1	38.9614	14673	146.6	2408	14.7	0.0027
2	223.0868	28198	211.7	3478	21.2	0.0079
3	225.1022	28527	548.3	9006	54.9	0.0079
4	252.1121	27771	249.6	4099	25.0	0.0091
5	267.2593	31276	198.9	3267	19.9	0.0085
6	310.1445	30965	222.8	3660	22.3	0.0100
7	341.2933	32883	288.3	4735	28.8	0.0104
8	381.2845	33183	999.3	16412	100.0	0.0115
9	382.2879	34618	510.1	8378	51.0	0.0110
10	383.2908	29858	163.1	2678	16.3	0.0128

Cmpd 27, Dissect, 4.2 min

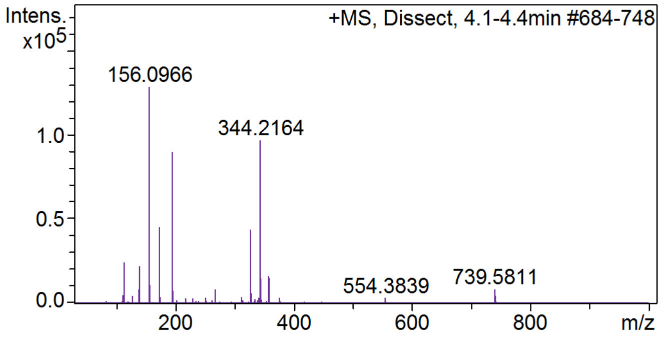

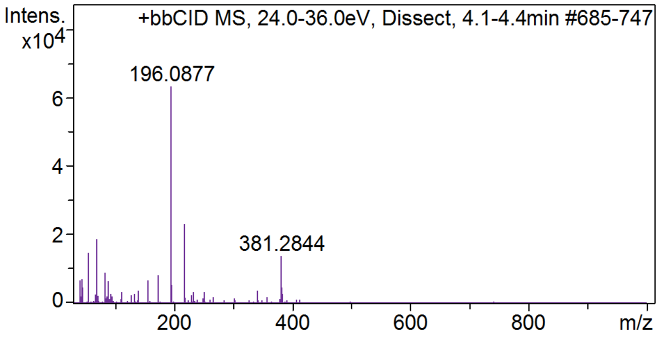

#	m/z	Res.	S/N	I	I %	FWHM
1	114.0875	22851	189.1	24350	19.0	0.0050
2	139.0706	24639	172.3	22185	17.3	0.0056
3	156.0966	27270	998.0	128483	100.0	0.0057
4	174.1065	27852	351.7	45272	35.2	0.0063
5	196.0878	29580	697.7	89816	69.9	0.0066
6	327.1903	34095	339.6	43720	34.0	0.0096
7	344.2164	33493	750.5	96621	75.2	0.0103
8	345.2196	31349	115.5	14865	11.6	0.0110
9	358.0542	33166	128.3	16512	12.9	0.0108
10	359.3034	34638	120.7	15534	12.1	0.0104

#	m/z	Res.	S/N	I	I %	FWHM
1	41.0368	14948	107.2	6788	10.7	0.0027
2	44.0112	15419	112.7	7133	11.3	0.0029
3	55.0520	17093	235.3	14902	23.5	0.0032
4	69.0673	18015	295.3	18697	29.5	0.0038
5	83.0827	19018	141.7	8970	14.2	0.0044
6	156.0965	26696	108.1	6847	10.8	0.0058
7	174.1217	27559	130.6	8269	13.1	0.0063
8	196.0877	29323	999.4	63280	100.0	0.0067
9	218.0689	29342	368.0	23303	36.8	0.0074
10	381.2844	33427	219.8	13920	22.0	0.0114

Compound Spectrum List Report

Cmpd 28, Dissect, 4.3 min

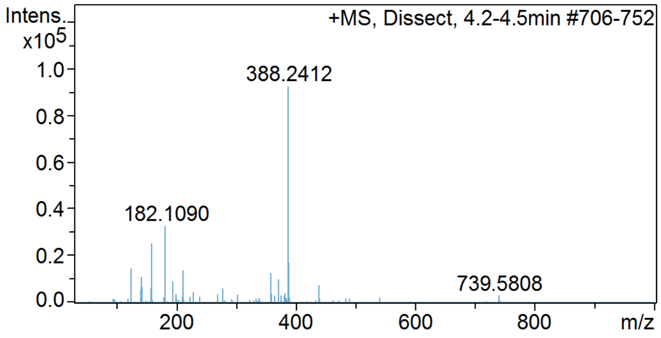

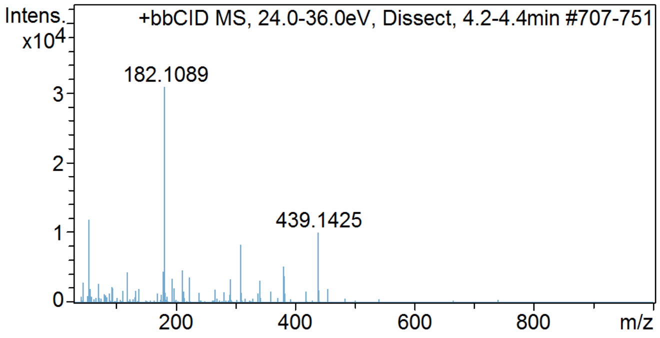

#	m/z	Res.	S/N	I	I %	FWHM
1	125.0919	24023	160.3	14823	16.0	0.0052
2	142.1178	24936	121.7	11259	12.2	0.0057
3	160.1278	26889	275.1	25446	27.5	0.0060
4	182.1090	27992	355.3	32861	35.5	0.0065
5	195.0812	28176	102.5	9481	10.3	0.0069
6	212.1122	17952	150.7	13940	15.1	0.0118
7	359.3037	34356	139.3	12882	13.9	0.0105
8	371.2152	30912	109.6	10138	11.0	0.0120
9	388.2412	34408	999.9	92484	100.0	0.0113
10	389.2446	32008	187.9	17377	18.8	0.0122

#	m/z	Res.	S/N	I	I %	FWHM
1	55.0520	17053	384.8	11903	38.5	0.0032
2	119.0565	22670	143.4	4435	14.3	0.0053
3	180.0936	26745	147.0	4547	14.7	0.0067
4	182.1089	28604	999.3	30912	100.0	0.0064
5	212.1143	16265	153.1	4736	15.3	0.0130
6	224.1182	28416	119.4	3694	11.9	0.0079
7	310.1443	31669	268.9	8318	26.9	0.0098
8	381.2845	33583	168.2	5203	16.8	0.0114
9	382.2877	34554	124.0	3837	12.4	0.0111
10	439.1425	32062	325.0	10055	32.5	0.0137

Cmpd 29, Dissect, 4.3 min

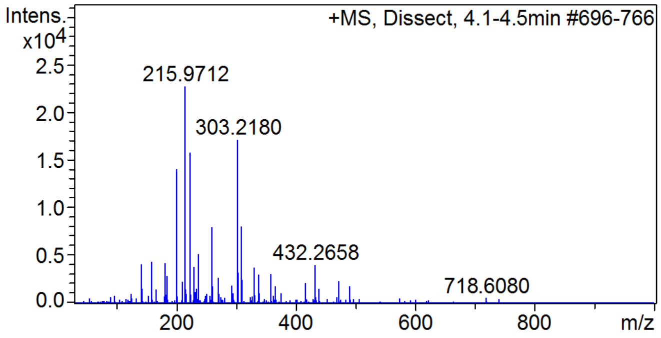

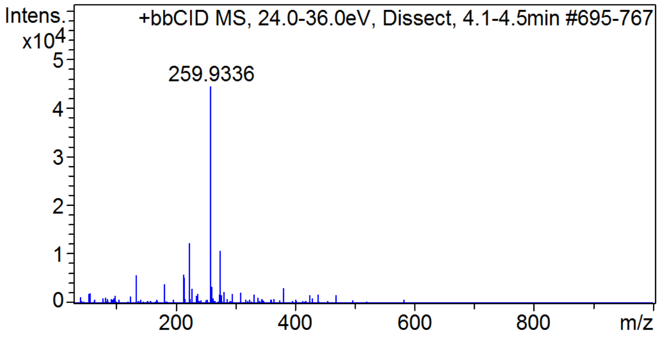

#	m/z	Res.	S/N	I	I %	FWHM
1	142.1178	25444	181.3	4119	18.1	0.0056
2	160.1278	26881	191.9	4359	19.2	0.0060
3	182.1090	27568	188.5	4283	18.9	0.0066
4	202.1371	28467	618.9	14059	61.9	0.0071
5	215.9712	29586	999.8	22712	100.0	0.0073
6	224.1182	29345	692.8	15738	69.3	0.0076
7	237.9526	29653	229.5	5213	23.0	0.0080
8	259.9340	31478	351.0	7972	35.1	0.0083
9	303.2180	31579	753.7	17121	75.4	0.0096
10	310.1448	30974	354.8	8059	35.5	0.0100

#	m/z	Res.	S/N	I	I %	FWHM
1	134.0012	25412	129.7	5766	13.0	0.0053
2	182.1090	28622	89.8	3993	9.0	0.0064
3	214.1368	29666	135.4	6018	13.6	0.0072
4	215.9711	30315	118.8	5283	11.9	0.0071
5	224.1182	27917	277.8	12350	27.8	0.0080
6	259.9336	32626	998.7	44396	100.0	0.0080
7	260.9362	26475	79.1	3516	7.9	0.0099
8	261.9292	29202	78.6	3493	7.9	0.0090
9	275.9068	31922	242.2	10766	24.2	0.0086
10	381.2846	34175	71.0	3154	7.1	0.0112

Compound Spectrum List Report

Cmpd 30, Dissect, 4.5 min

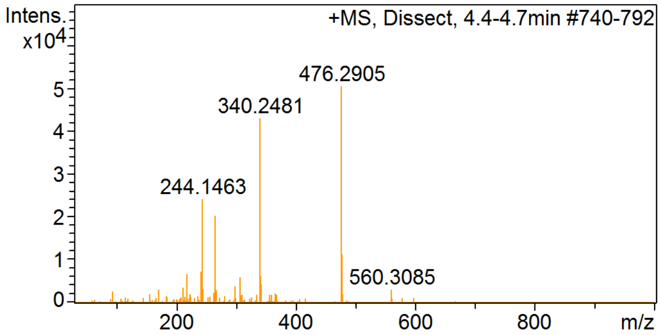

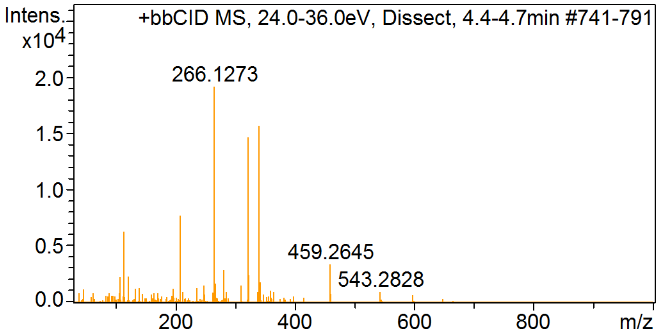

#	m/z	Res.	S/N	I	I %	FWHM
1	219.1057	28681	134.7	6799	13.5	0.0076
2	242.1305	30581	145.6	7349	14.6	0.0079
3	244.1463	30208	478.0	24131	47.9	0.0081
4	266.1275	30233	401.4	20266	40.2	0.0088
5	307.1913	28409	118.3	5971	11.8	0.0108
6	340.2481	33172	848.8	42848	85.0	0.0103
7	341.2512	30589	125.6	6341	12.6	0.0112
8	343.1799	28527	85.6	4322	8.6	0.0120
9	476.2905	32943	998.8	50424	100.0	0.0145
10	477.2938	31198	226.2	11422	22.7	0.0153

#	m/z	Res.	S/N	I	I %	FWHM
1	107.9633	21313	120.4	2311	12.1	0.0051
2	114.0875	22729	328.7	6310	32.9	0.0050
3	121.0972	22506	121.5	2332	12.2	0.0054
4	209.1579	29423	403.8	7752	40.4	0.0071
5	266.1273	30868	998.8	19176	100.0	0.0086
6	282.1005	30493	150.8	2895	15.1	0.0093
7	322.2381	32421	762.5	14640	76.3	0.0099
8	323.2408	31751	128.2	2462	12.8	0.0102
9	340.2481	31902	814.7	15642	81.6	0.0107
10	459.2645	30688	177.4	3406	17.8	0.0150

Cmpd 31, Dissect, 4.6 min

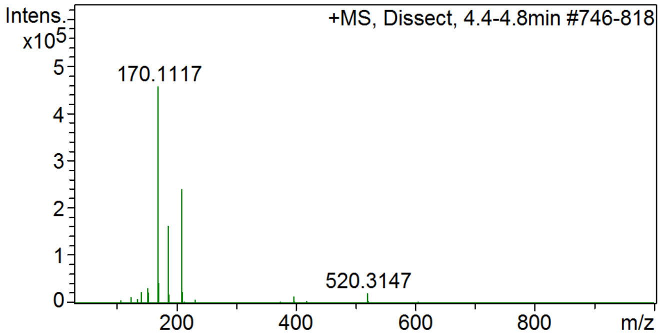

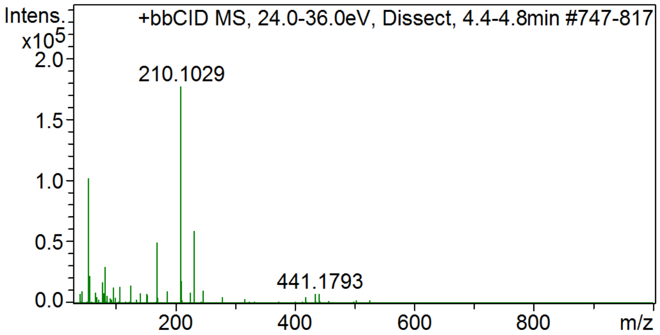

#	m/z	Res.	S/N	I	I %	FWHM
1	142.1177	25411	54.8	25149	5.5	0.0056
2	152.1017	26080	71.4	32810	7.2	0.0058
3	153.0857	26112	50.6	23240	5.1	0.0059
4	170.1117	29284	995.2	457069	100.0	0.0058
5	171.1151	26483	94.0	43182	9.4	0.0065
6	188.1216	29362	356.4	163698	35.8	0.0064
7	189.1250	26914	39.5	18151	4.0	0.0070
8	210.1029	30439	524.7	240983	52.7	0.0069
9	211.1062	28397	53.4	24510	5.4	0.0074
10	520.3147	32166	49.5	22721	5.0	0.0162

#	m/z	Res.	S/N	I	I %	FWHM
1	55.0156	16859	89.5	15845	9.0	0.0033
2	55.0520	17409	576.7	102062	57.7	0.0032
3	57.0676	17099	128.1	22675	12.8	0.0033
4	79.0514	19077	97.4	17242	9.7	0.0041
5	83.0825	19303	170.8	30217	17.1	0.0043
6	125.0919	23558	83.3	14747	8.3	0.0053
7	170.1116	28177	280.2	49594	28.0	0.0060
8	210.1029	29260	999.3	176845	100.0	0.0072
9	211.1061	28388	103.5	18319	10.4	0.0074
10	232.0841	30432	333.6	59034	33.4	0.0076

Compound Spectrum List Report

Cmpd 32, Dissect, 4.7 min

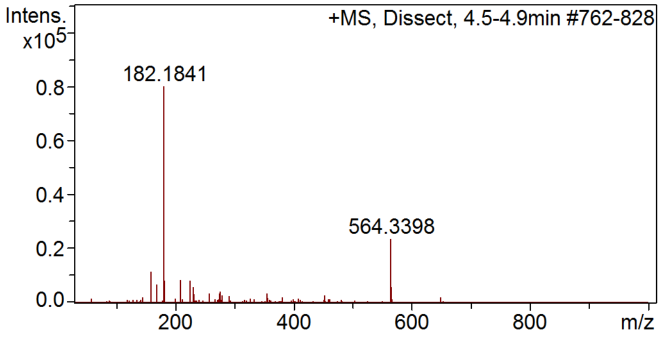

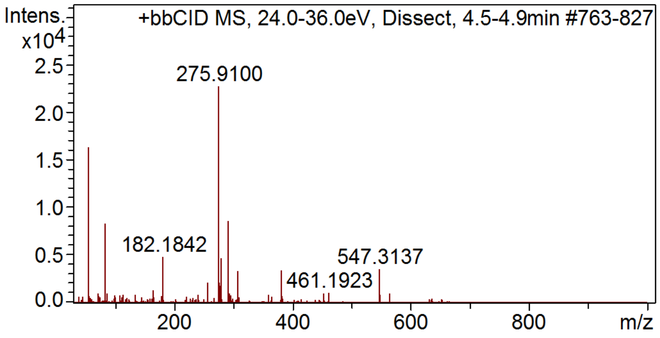

#	m/z	Res.	S/N	I	I %	FWHM
1	159.0593	25289	145.4	11650	14.5	0.0063
2	170.1118	28788	85.8	6871	8.6	0.0059
3	182.1841	28540	999.8	80112	100.0	0.0064
4	183.1875	27374	103.5	8297	10.4	0.0067
5	210.1031	29977	108.6	8704	10.9	0.0070
6	226.1341	30247	105.5	8451	10.5	0.0075
7	231.9480	29067	73.8	5912	7.4	0.0080
8	277.0591	29542	55.8	4474	5.6	0.0094
9	564.3398	30861	296.2	23732	29.6	0.0183
10	565.3421	31899	74.4	5963	7.4	0.0177

#	m/z	Res.	S/N	I	I %	FWHM
1	55.0519	17296	716.3	16289	71.7	0.0032
2	83.0826	19153	367.0	8347	36.8	0.0043
3	182.1842	27173	213.5	4856	21.4	0.0067
4	257.1271	28646	95.2	2164	9.5	0.0090
5	275.9100	31265	998.5	22708	100.0	0.0088
6	280.1423	29473	208.0	4731	20.8	0.0095
7	291.8836	30095	378.0	8596	37.9	0.0097
8	307.8570	31593	147.9	3364	14.8	0.0097
9	381.2843	31845	151.9	3454	15.2	0.0120
10	547.3137	34613	157.1	3573	15.7	0.0158

Cmpd 33, Dissect, 4.7 min

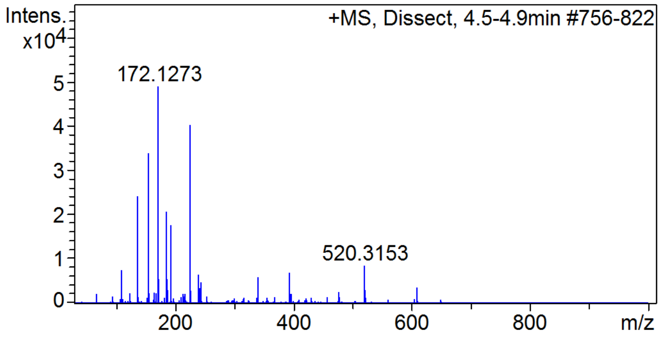

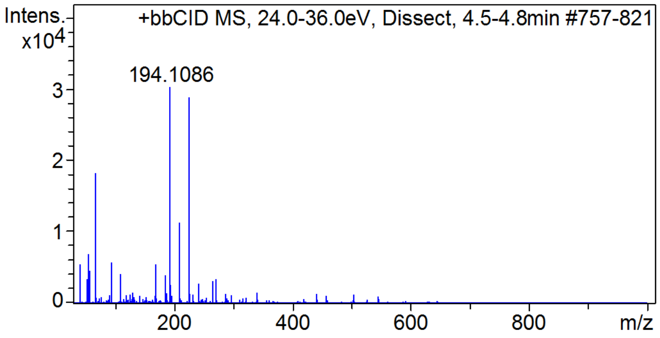

#	m/z	Res.	S/N	I	I %	FWHM
1	109.0975	21768	153.3	7510	15.3	0.0050
2	136.0710	25036	493.0	24151	49.3	0.0054
3	155.1012	26333	691.4	33874	69.2	0.0059
4	172.1273	27616	999.8	48983	100.0	0.0062
5	186.2153	27993	422.3	20690	42.2	0.0067
6	194.1086	28239	360.0	17638	36.0	0.0069
7	226.1340	30457	820.2	40181	82.0	0.0074
8	240.1415	27543	133.0	6518	13.3	0.0087
9	393.2514	32198	142.7	6989	14.3	0.0122
10	520.3153	33170	174.0	8526	17.4	0.0157

#	m/z	Res.	S/N	I	I %	FWHM
1	41.0368	14942	182.1	5528	18.2	0.0027
2	55.0520	17067	228.7	6945	22.9	0.0032
3	57.0676	17209	151.2	4592	15.1	0.0033
4	67.0518	17503	599.1	18191	60.0	0.0038
5	94.0621	19317	190.9	5796	19.1	0.0049
6	170.1117	28075	180.8	5489	18.1	0.0061
7	194.1086	28978	998.9	30332	100.0	0.0067
8	210.0819	28556	201.5	6118	20.2	0.0074
9	210.1031	28606	371.6	11284	37.2	0.0073
10	226.1336	30677	948.4	28796	94.9	0.0074

Compound Spectrum List Report

Cmpd 34, Dissect, 4.8 min

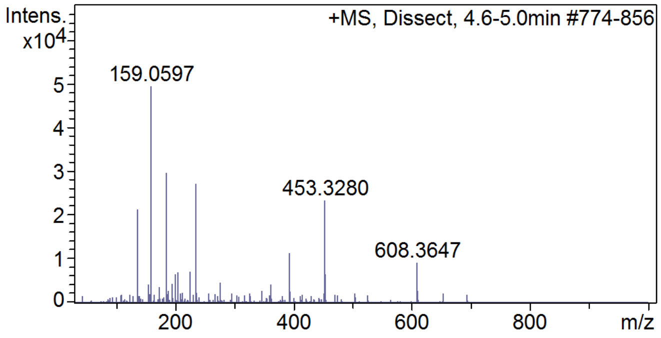

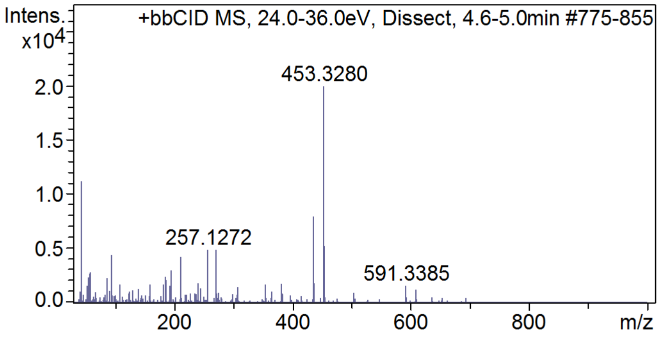

#	m/z	Res.	S/N	I	I %	FWHM
1	136.0711	25068	430.6	21289	43.1	0.0054
2	159.0597	26687	999.9	49435	100.0	0.0060
3	186.2153	28170	598.4	29584	59.8	0.0066
4	205.1003	28154	141.6	6999	14.2	0.0073
5	226.1342	30143	145.8	7210	14.6	0.0075
6	236.1051	29852	549.5	27166	55.0	0.0079
7	393.2516	32284	230.5	11396	23.1	0.0122
8	453.3280	30611	472.8	23374	47.3	0.0148
9	454.3310	31216	133.9	6620	13.4	0.0146
10	608.3647	30200	187.9	9289	18.8	0.0201

#	m/z	Res.	S/N	I	I %	FWHM
1	43.0160	15539	562.2	11212	56.2	0.0028
2	59.0469	17053	143.0	2852	14.3	0.0035
3	94.0621	19472	222.6	4440	22.3	0.0048
4	196.1242	26942	152.2	3035	15.2	0.0073
5	212.1111	27200	212.6	4240	21.3	0.0078
6	257.1272	30126	247.2	4930	24.7	0.0085
7	271.1713	30484	246.7	4920	24.7	0.0089
8	435.3180	29441	399.5	7967	40.0	0.0148
9	453.3280	32826	999.9	19940	100.0	0.0138
10	454.3315	29908	264.8	5280	26.5	0.0152

Cmpd 35, Dissect, 4.9 min

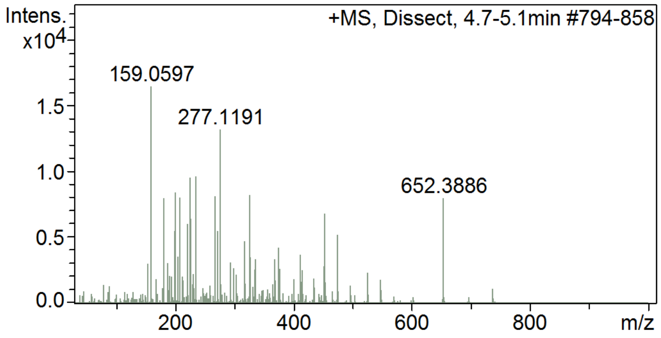

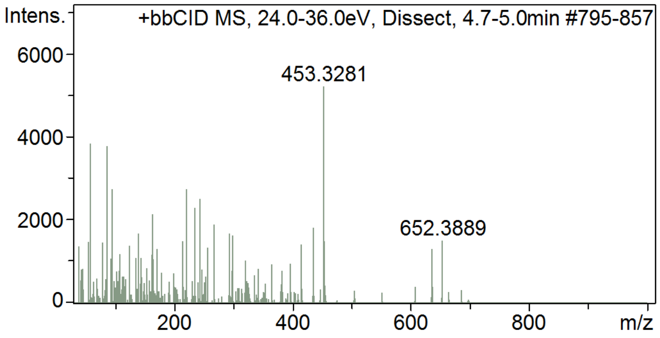

#	m/z	Res.	S/N	I	I %	FWHM
1	159.0597	26330	999.9	16452	100.0	0.0060
2	182.1115	28255	484.6	7974	48.5	0.0064
3	201.0956	28065	510.9	8405	51.1	0.0072
4	209.1100	27942	488.8	8043	48.9	0.0075
5	226.1359	29779	578.3	9514	57.8	0.0076
6	236.1051	29285	584.5	9616	58.5	0.0081
7	268.1432	29637	493.3	8116	49.3	0.0090
8	277.1191	31260	801.1	13181	80.1	0.0089
9	326.6991	34063	500.7	8238	50.1	0.0096
10	652.3886	31360	485.2	7982	48.5	0.0208

#	m/z	Res.	S/N	I	I %	FWHM
1	58.0628	17179	736.4	3839	73.6	0.0034
2	87.0411	19554	725.0	3779	72.5	0.0045
3	95.0824	19713	525.4	2738	52.5	0.0048
4	164.9149	26250	412.2	2149	41.2	0.0063
5	222.1027	27637	524.7	2735	52.5	0.0080
6	236.1174	27555	440.4	2296	44.0	0.0086
7	244.0838	28763	480.6	2505	48.1	0.0085
8	268.1427	27430	362.8	1891	36.3	0.0098
9	435.3180	28529	350.3	1826	35.0	0.0153
10	453.3281	32339	1000.0	5212	100.0	0.0140

Compound Spectrum List Report

Cmpd 36, Dissect, 5.0 min

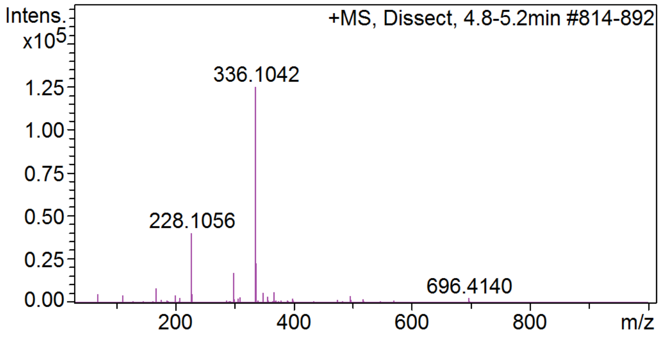

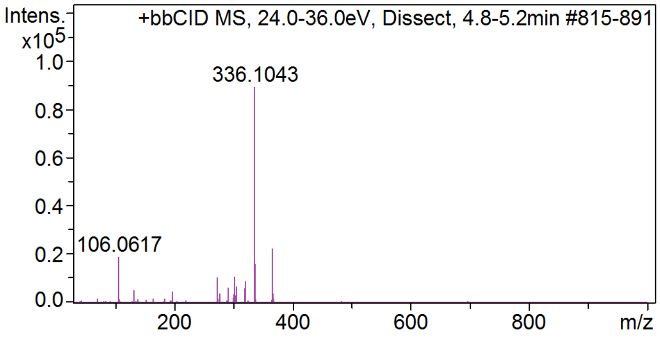

#	m/z	Res.	S/N	I	I %	FWHM
1	69.0673	17911	40.9	5116	4.1	0.0039
2	111.1132	21598	35.0	4375	3.5	0.0051
3	169.1157	24959	68.0	8500	6.8	0.0068
4	228.1056	30528	323.5	40432	32.4	0.0075
5	229.1089	27671	39.6	4953	4.0	0.0083
6	299.0784	32727	140.9	17614	14.1	0.0091
7	336.1042	32952	999.3	124906	100.0	0.0102
8	337.1075	30952	184.7	23084	18.5	0.0109
9	348.7111	33943	49.3	6161	4.9	0.0103
10	368.0752	30990	51.2	6405	5.1	0.0119

#	m/z	Res.	S/N	I	I %	FWHM
1	106.0617	21922	212.5	19020	21.3	0.0048
2	274.0696	30956	119.2	10673	11.9	0.0089
3	292.0795	29214	72.1	6450	7.2	0.0100
4	303.0713	30861	121.7	10895	12.2	0.0098
5	306.0583	29678	78.9	7060	7.9	0.0103
6	320.0737	30627	70.6	6318	7.1	0.0105
7	321.0810	30108	102.1	9136	10.2	0.0107
8	336.1043	34644	999.3	89438	100.0	0.0097
9	337.1081	28300	180.4	16146	18.1	0.0119
10	366.0596	32618	252.1	22562	25.2	0.0112

Cmpd 37, Dissect, 5.1 min

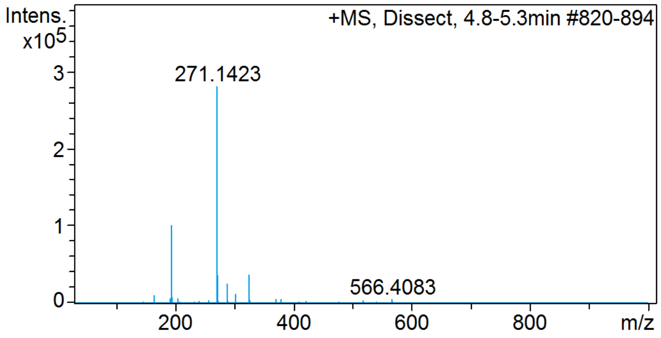

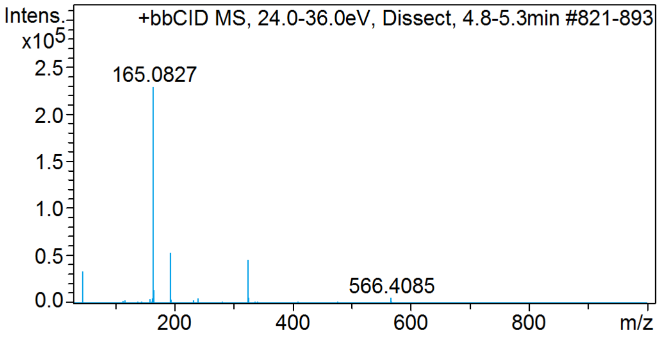

#	m/z	Res.	S/N	I	I %	FWHM
1	165.0830	25740	36.9	10375	3.7	0.0064
2	192.0703	28095	22.3	6270	2.2	0.0068
3	195.0924	29444	360.7	101523	36.1	0.0066
4	196.0950	23294	28.8	8114	2.9	0.0084
5	205.1155	28261	21.8	6127	2.2	0.0073
6	271.1423	32922	998.3	281006	100.0	0.0082
7	272.1451	29132	132.2	37204	13.2	0.0093
8	289.1530	35803	92.6	26078	9.3	0.0081
9	303.1677	32009	44.4	12507	4.5	0.0095
10	325.1489	32479	132.5	37291	13.3	0.0100

#	m/z	Res.	S/N	I	I %	FWHM
1	45.0315	15766	148.8	34111	14.9	0.0029
2	160.0450	26716	18.6	4264	1.9	0.0060
3	164.9148	26111	21.0	4827	2.1	0.0063
4	165.0827	28278	997.7	228787	100.0	0.0058
5	166.0856	21378	63.7	14612	6.4	0.0078
6	195.0923	29418	234.4	53762	23.5	0.0066
7	241.1327	29643	22.8	5228	2.3	0.0081
8	325.1487	32920	201.3	46169	20.2	0.0099
9	326.1511	28178	25.2	5778	2.5	0.0116
10	566.4085	29689	25.9	5947	2.6	0.0191

Compound Spectrum List Report

Cmpd 38, Dissect, 5.2 min

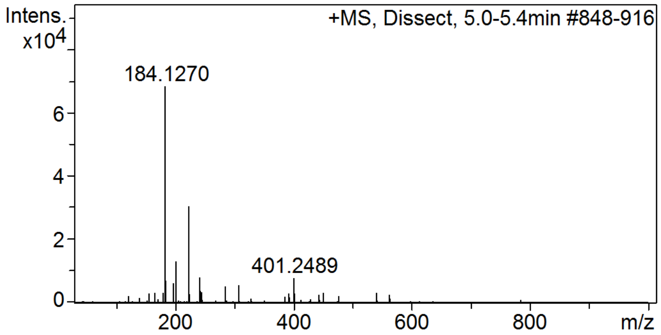

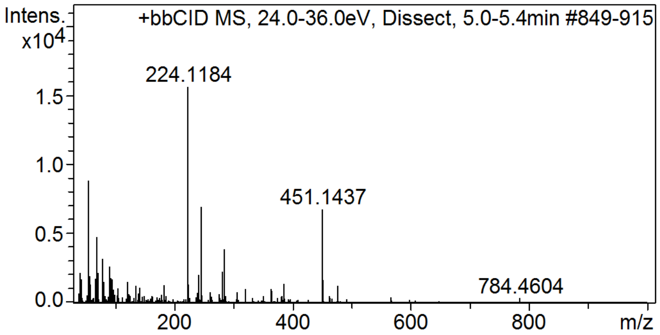

#	m/z	Res.	S/N	I	I %	FWHM
1	184.1270	28342	999.3	68374	100.0	0.0065
2	185.1303	26297	105.3	7205	10.5	0.0070
3	198.1422	28603	91.4	6255	9.1	0.0069
4	202.1371	28703	192.1	13145	19.2	0.0070
5	224.1184	29438	445.2	30460	44.5	0.0076
6	242.1458	29095	120.2	8222	12.0	0.0083
7	242.2761	29745	78.4	5365	7.8	0.0081
8	285.1213	30066	76.7	5249	7.7	0.0095
9	308.2116	30095	83.3	5697	8.3	0.0102
10	401.2489	35760	116.3	7959	11.6	0.0112

#	m/z	Res.	S/N	I	I %	FWHM
1	55.0520	17127	566.0	8844	56.6	0.0032
2	69.0673	17752	307.1	4798	30.7	0.0039
3	71.0466	18363	139.1	2174	13.9	0.0039
4	79.0513	18728	205.6	3213	20.6	0.0042
5	91.0513	19961	169.3	2646	16.9	0.0046
6	224.1184	29090	999.9	15623	100.0	0.0077
7	246.0995	29213	445.8	6966	44.6	0.0084
8	282.1585	29486	146.8	2294	14.7	0.0096
9	285.1213	29807	248.2	3878	24.8	0.0096
10	451.1437	30876	431.8	6746	43.2	0.0146

Cmpd 39, Dissect, 5.2 min

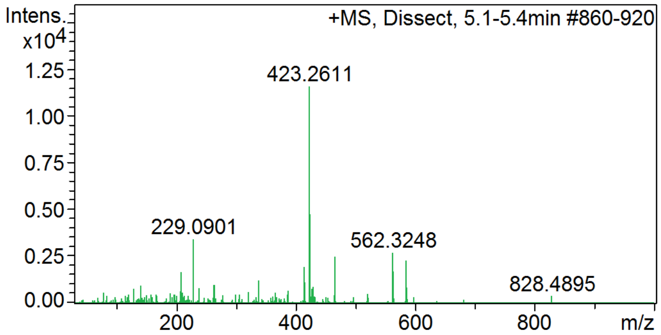

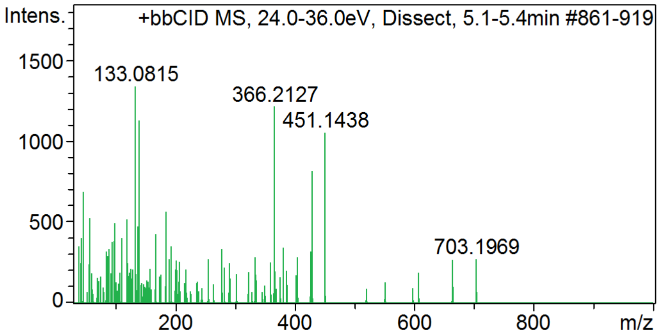

#	m/z	Res.	S/N	I	I %	FWHM
1	229.0901	25961	296.3	3430	29.6	0.0088
2	414.7483	33794	170.6	1974	17.1	0.0123
3	423.2611	34957	999.5	11569	100.0	0.0121
4	423.7625	33067	410.4	4750	41.1	0.0128
5	424.2606	24885	210.6	2437	21.1	0.0170
6	465.2701	35570	216.6	2507	21.7	0.0131
7	562.3248	32762	235.7	2728	23.6	0.0172
8	562.8269	33205	148.4	1717	14.8	0.0169
9	584.3368	31961	197.9	2290	19.8	0.0183
10	584.8394	36675	183.3	2121	18.3	0.0159

#	m/z	Res.	S/N	I	I %	FWHM
1	46.9930	16050	512.9	687	51.3	0.0029
2	57.0677	17111	394.2	528	39.5	0.0033
3	99.0408	20079	370.6	497	37.1	0.0049
4	119.0564	22726	387.3	519	38.8	0.0052
5	133.0815	24107	999.3	1339	100.0	0.0055
6	139.9100	25694	841.1	1127	84.2	0.0054
7	185.0746	26808	422.6	566	42.3	0.0069
8	366.2127	30975	907.2	1215	90.8	0.0118
9	429.2718	31462	609.8	817	61.0	0.0136
10	451.1438	30147	784.4	1051	78.5	0.0150

Compound Spectrum List Report

Cmpd 40, Dissect, 5.4 min

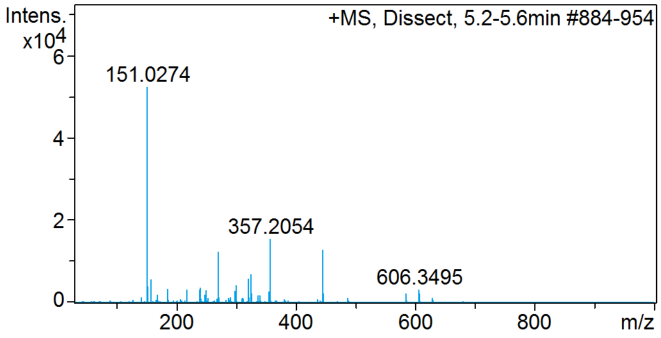

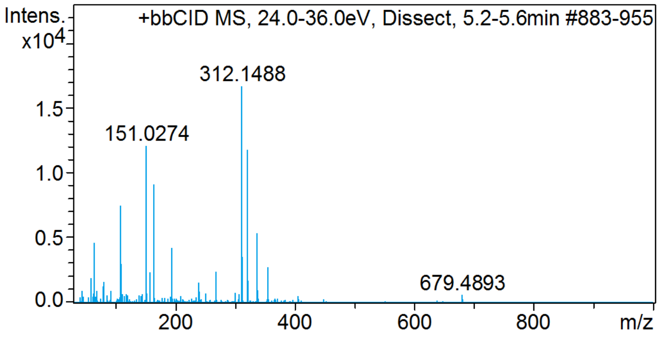

#	m/z	Res.	S/N	I	I %	FWHM
1	151.0274	26026	999.9	52446	100.0	0.0058
2	152.0304	24272	79.2	4154	7.9	0.0063
3	158.0911	25850	110.9	5818	11.1	0.0061
4	271.1423	29418	236.3	12394	23.6	0.0092
5	301.1520	28887	85.0	4456	8.5	0.0104
6	321.1205	33328	113.9	5973	11.4	0.0096
7	325.2167	29592	133.3	6993	13.3	0.0110
8	357.2054	32673	297.1	15583	29.7	0.0109
9	445.2741	35870	244.9	12844	24.5	0.0124
10	445.7760	34258	101.9	5344	10.2	0.0130

#	m/z	Res.	S/N	I	I %	FWHM
1	65.0362	17252	277.0	4624	27.7	0.0038
2	109.0070	21699	447.9	7476	44.8	0.0050
3	151.0274	25716	722.5	12059	72.3	0.0059
4	164.9148	26461	281.4	4698	28.2	0.0062
5	165.0831	25461	546.5	9122	54.7	0.0065
6	195.0908	22264	253.8	4236	25.4	0.0088
7	312.1488	31785	998.9	16673	100.0	0.0098
8	313.1523	29452	214.6	3581	21.5	0.0106
9	321.1201	31436	706.0	11784	70.7	0.0102
10	337.0935	30077	323.7	5404	32.4	0.0112

Cmpd 41, Dissect, 5.4 min

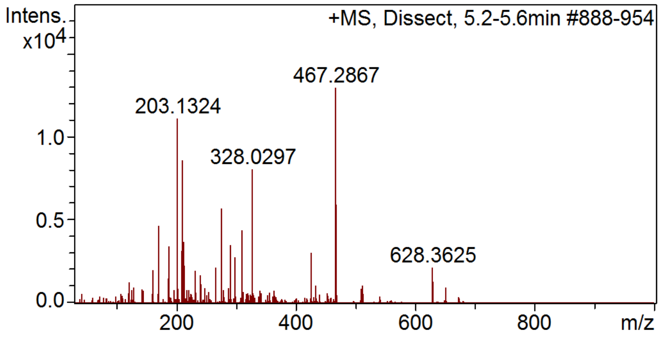

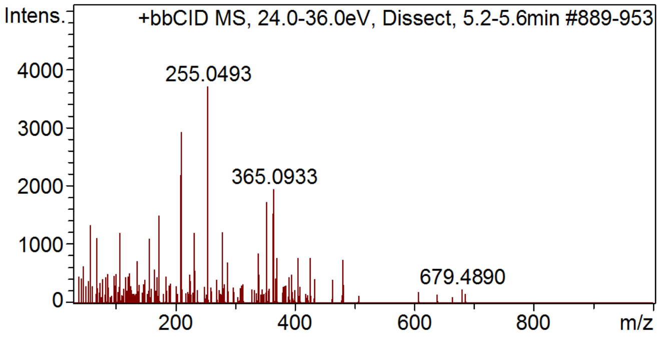

#	m/z	Res.	S/N	I	I %	FWHM
1	171.0959	26346	358.6	4653	35.9	0.0065
2	203.1324	28141	852.7	11063	85.3	0.0072
3	211.0872	27943	661.7	8585	66.2	0.0076
4	213.1047	26955	284.5	3691	28.5	0.0079
5	276.1868	30003	440.5	5715	44.1	0.0092
6	291.1343	32516	270.1	3505	27.0	0.0090
7	311.0036	31671	337.8	4382	33.8	0.0098
8	328.0297	30819	620.2	8047	62.0	0.0106
9	467.2867	33502	999.9	12974	100.0	0.0139
10	467.7883	32448	456.8	5926	45.7	0.0144

#	m/z	Res.	S/N	I	I %	FWHM
1	58.0628	17336	360.6	1336	36.1	0.0033
2	174.1221	26078	403.9	1497	40.4	0.0067
3	210.1398	27600	591.5	2192	59.2	0.0076
4	211.0873	28361	787.0	2916	78.7	0.0074
5	211.1204	26177	396.1	1468	39.6	0.0081
6	255.0493	28729	999.7	3704	100.0	0.0089
7	280.1422	27952	329.8	1222	33.0	0.0100
8	353.1422	27957	468.3	1735	46.8	0.0126
9	364.1974	29605	415.4	1539	41.6	0.0123
10	365.0933	31848	524.7	1944	52.5	0.0115

Compound Spectrum List Report

Cmpd 42, Dissect, 5.5 min

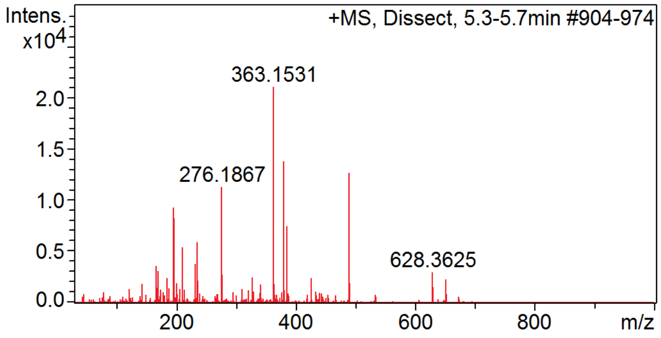

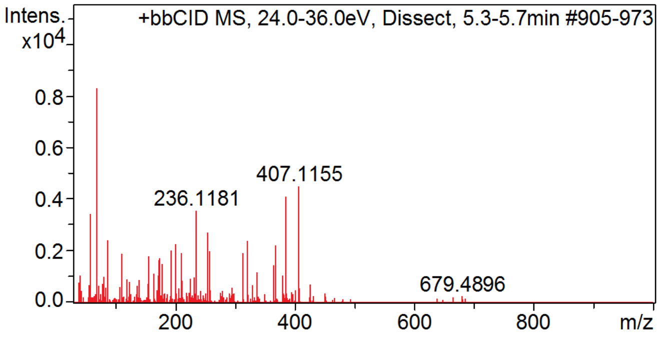

#	m/z	Res.	S/N	I	I %	FWHM
1	196.1266	27860	442.1	9315	44.2	0.0070
2	197.0566	28251	390.0	8218	39.0	0.0070
3	211.0871	26610	257.7	5431	25.8	0.0079
4	236.1177	27490	282.1	5944	28.2	0.0086
5	276.1867	31539	535.5	11284	53.6	0.0088
6	363.1531	32361	999.8	21067	100.0	0.0112
7	380.1786	31029	655.0	13802	65.5	0.0123
8	385.1340	32706	356.5	7512	35.7	0.0118
9	489.2984	34549	601.9	12682	60.2	0.0142
10	489.7997	31195	275.1	5796	27.5	0.0157

#	m/z	Res.	S/N	I	I %	FWHM
1	58.0628	17263	412.5	3432	41.3	0.0034
2	69.0309	17927	999.2	8313	100.0	0.0039
3	69.0673	17771	538.4	4480	53.9	0.0039
4	87.0411	18604	291.7	2427	29.2	0.0047
5	202.0367	28013	275.6	2293	27.6	0.0072
6	236.1181	28470	427.5	3557	42.8	0.0083
7	255.0495	26924	328.2	2730	32.8	0.0095
8	321.1207	31113	289.8	2412	29.0	0.0103
9	385.1341	31394	494.0	4110	49.4	0.0123
10	407.1155	30088	542.0	4509	54.2	0.0135

Cmpd 43, Dissect, 5.6 min

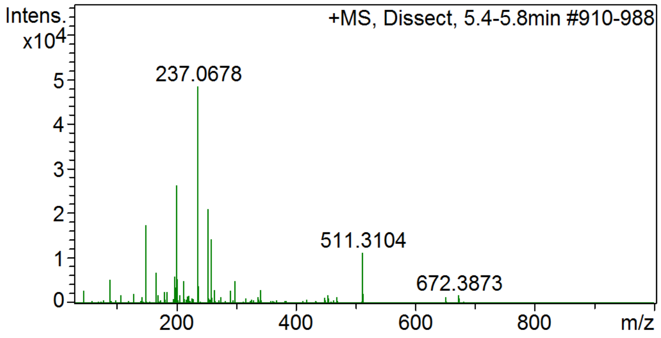

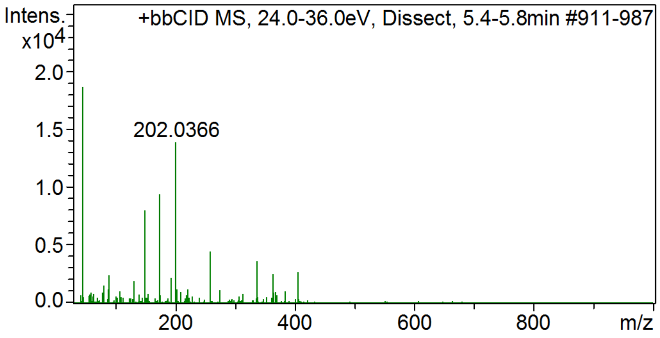

#	m/z	Res.	S/N	I	I %	FWHM
1	89.0567	19251	111.9	5423	11.2	0.0046
2	149.0183	25895	361.9	17529	36.2	0.0058
3	167.1010	26754	141.4	6850	14.2	0.0062
4	198.1422	28688	125.5	6077	12.6	0.0069
5	202.0364	29146	543.9	26347	54.5	0.0069
6	202.1369	28879	114.9	5564	11.5	0.0070
7	237.0678	30576	998.8	48383	100.0	0.0078
8	254.0938	30242	434.5	21046	43.5	0.0084
9	259.0491	31515	294.7	14276	29.5	0.0082
10	511.3104	34741	233.4	11304	23.4	0.0147

#	m/z	Res.	S/N	I	I %	FWHM
1	45.0316	15671	998.0	18696	100.0	0.0029
2	89.0568	19074	131.7	2468	13.2	0.0047
3	149.0183	24658	429.3	8042	43.0	0.0060
4	149.0910	25966	132.1	2474	13.2	0.0057
5	175.0266	26384	500.9	9384	50.2	0.0066
6	202.0366	28567	739.9	13860	74.1	0.0071
7	259.0493	29308	239.6	4488	24.0	0.0088
8	337.1142	28890	195.9	3669	19.6	0.0117
9	364.1970	30837	137.5	2575	13.8	0.0118
10	406.2039	30130	145.6	2728	14.6	0.0135

Compound Spectrum List Report

Cmpd 44, Dissect, 5.7 min

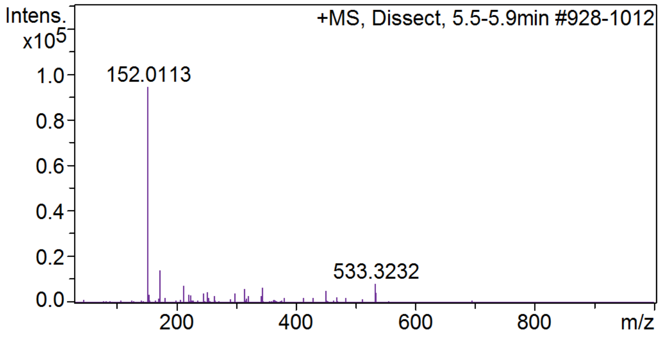

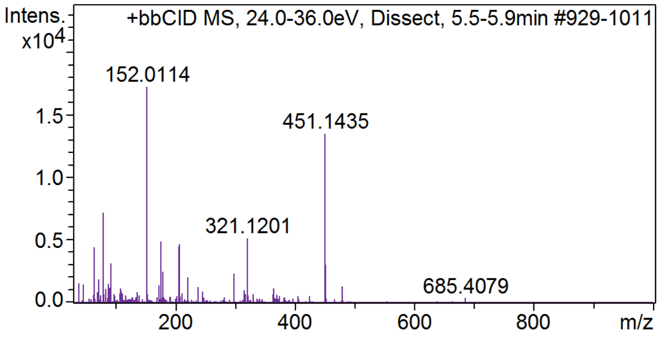

#	m/z	Res.	S/N	I	I %	FWHM
1	152.0113	26771	999.7	94297	100.0	0.0057
2	173.9924	28041	152.7	14401	15.3	0.0062
3	213.1525	28329	81.3	7673	8.1	0.0075
4	246.9888	30635	46.3	4363	4.6	0.0081
5	253.1462	29277	51.8	4886	5.2	0.0086
6	315.1670	29843	66.7	6292	6.7	0.0106
7	345.1765	31204	73.9	6969	7.4	0.0111
8	451.1440	33481	56.3	5309	5.6	0.0135
9	533.3232	34396	89.3	8422	8.9	0.0155
10	533.8244	33176	49.0	4625	4.9	0.0161

#	m/z	Res.	S/N	I	I %	FWHM
1	65.0363	17276	259.5	4469	26.0	0.0038
2	80.0467	18793	419.3	7220	41.9	0.0043
3	92.0465	19552	184.2	3172	18.4	0.0047
4	152.0114	25835	999.8	17216	100.0	0.0059
5	177.0826	27124	286.5	4933	28.7	0.0065
6	207.0922	28024	260.6	4487	26.1	0.0074
7	208.0900	26938	273.1	4702	27.3	0.0077
8	321.1201	31137	299.3	5154	29.9	0.0103
9	451.1435	32196	779.8	13428	78.0	0.0140
10	452.1459	30808	177.7	3060	17.8	0.0147

Cmpd 45, Dissect, 5.8 min

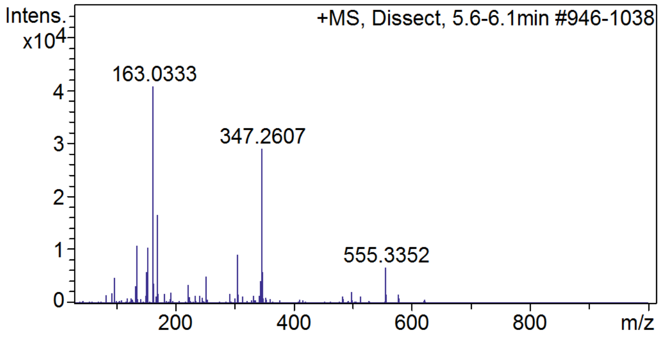

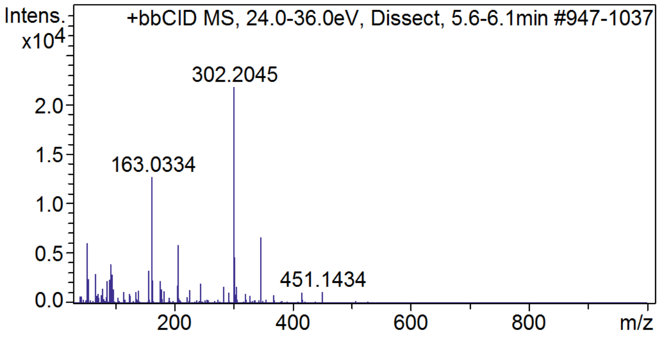

#	m/z	Res.	S/N	I	I %	FWHM
1	135.1121	24353	265.4	10831	26.6	0.0055
2	151.1065	25507	144.9	5915	14.5	0.0059
3	153.1221	25930	256.3	10461	25.6	0.0059
4	163.0333	26796	999.4	40788	100.0	0.0061
5	171.1321	26832	408.8	16685	40.9	0.0064
6	253.1461	28176	125.8	5135	12.6	0.0090
7	306.2112	32339	226.2	9231	22.6	0.0095
8	347.2607	32930	712.5	29080	71.3	0.0105
9	348.2632	29011	144.5	5895	14.5	0.0120
10	555.3352	32145	166.8	6809	16.7	0.0173

#	m/z	Res.	S/N	I	I %	FWHM
1	53.0364	16592	278.9	6084	27.9	0.0032
2	67.0519	17479	137.1	2991	13.7	0.0038
3	93.0668	19771	182.7	3985	18.3	0.0047
4	157.0958	23909	152.4	3324	15.3	0.0066
5	163.0334	26304	583.6	12730	58.4	0.0062
6	163.0677	25032	138.4	3020	13.9	0.0065
7	208.0899	28267	269.3	5875	27.0	0.0074
8	302.2045	31312	998.6	21784	100.0	0.0097
9	303.2068	28790	214.0	4669	21.4	0.0105
10	347.2608	30921	306.2	6679	30.7	0.0112

Compound Spectrum List Report

Cmpd 46, Dissect, 5.9 min

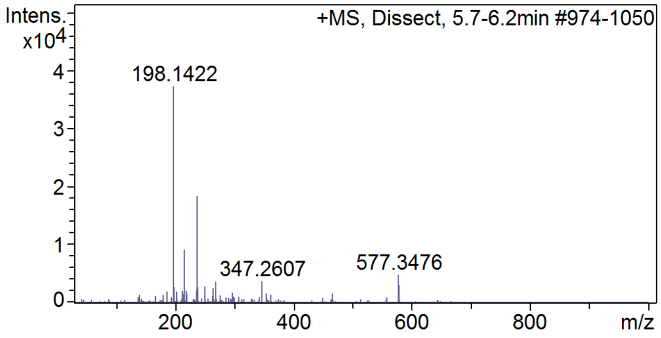

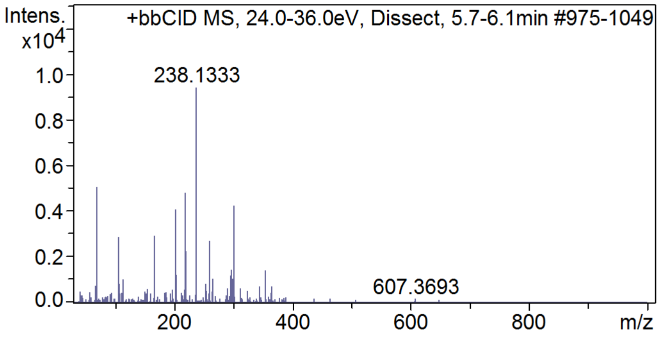

#	m/z	Res.	S/N	I	I %	FWHM
1	198.1422	29373	998.4	37288	100.0	0.0067
2	199.0799	27209	75.7	2826	7.6	0.0073
3	216.1522	29862	245.7	9177	24.6	0.0072
4	238.1334	30160	493.3	18424	49.4	0.0079
5	239.1400	28223	73.7	2754	7.4	0.0085
6	251.1554	30210	77.0	2875	7.7	0.0083
7	269.1654	29131	98.4	3673	9.9	0.0092
8	347.2607	32814	100.5	3754	10.1	0.0106
9	577.3476	33380	129.6	4841	13.0	0.0173
10	577.8488	34665	83.2	3107	8.3	0.0167

#	m/z	Res.	S/N	I	I %	FWHM
1	69.0673	17920	538.2	5068	53.8	0.0039
2	106.0616	21882	306.8	2888	30.7	0.0048
3	167.9878	26855	311.9	2937	31.2	0.0063
4	203.0459	27682	433.1	4078	43.3	0.0073
5	219.0192	28182	320.5	3017	32.1	0.0078
6	219.1669	28030	509.9	4801	51.0	0.0078
7	220.1258	27504	240.7	2266	24.1	0.0080
8	238.1333	30072	999.8	9413	100.0	0.0079
9	260.1146	30545	290.2	2732	29.0	0.0085
10	302.2045	31584	450.4	4241	45.1	0.0096

Cmpd 47, Dissect, 6.0 min

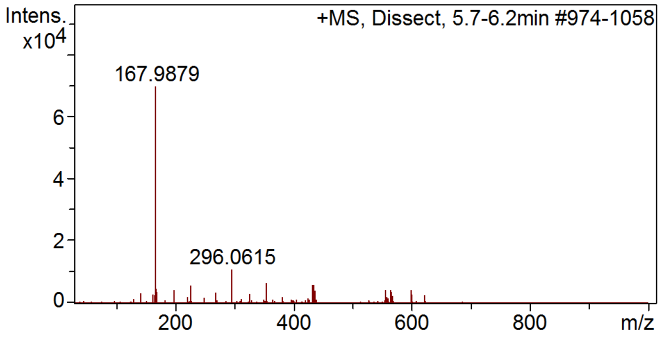

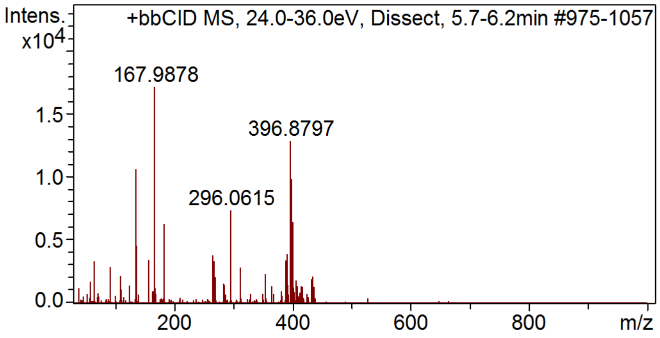

#	m/z	Res.	S/N	I	I %	FWHM
1	167.9879	27540	999.3	69665	100.0	0.0061
2	168.9909	22449	69.9	4870	7.0	0.0075
3	199.0799	27230	64.0	4459	6.4	0.0073
4	227.1465	29123	82.8	5771	8.3	0.0078
5	296.0615	31410	158.5	11047	15.9	0.0094
6	354.9344	31304	95.9	6682	9.6	0.0113
7	432.8553	31288	85.6	5966	8.6	0.0138
8	434.8522	32008	86.9	6056	8.7	0.0136
9	555.8670	38995	63.0	4392	6.3	0.0143
10	563.8599	30856	64.4	4490	6.4	0.0183

#	m/z	Res.	S/N	I	I %	FWHM
1	135.0091	24774	620.6	10629	62.1	0.0054
2	136.0165	22384	268.1	4592	26.8	0.0061
3	167.9878	26772	999.5	17118	100.0	0.0063
4	184.0932	26620	367.4	6292	36.8	0.0069
5	265.8747	30449	223.4	3827	22.4	0.0087
6	296.0615	30876	430.4	7372	43.1	0.0096
7	390.8859	28590	229.0	3921	22.9	0.0137
8	396.8797	32168	749.0	12827	74.9	0.0123
9	398.8766	30229	576.6	9875	57.7	0.0132
10	400.8750	30872	377.3	6461	37.7	0.0130

Compound Spectrum List Report

Cmpd 48, Dissect, 6.1 min

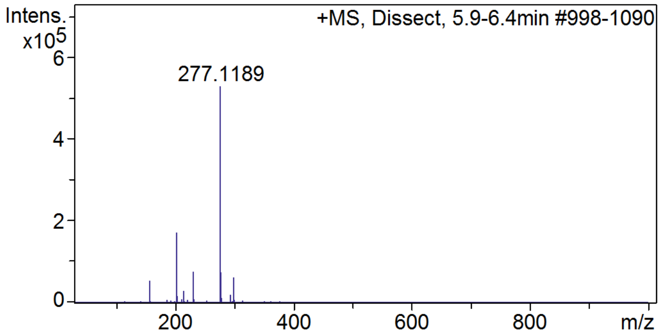

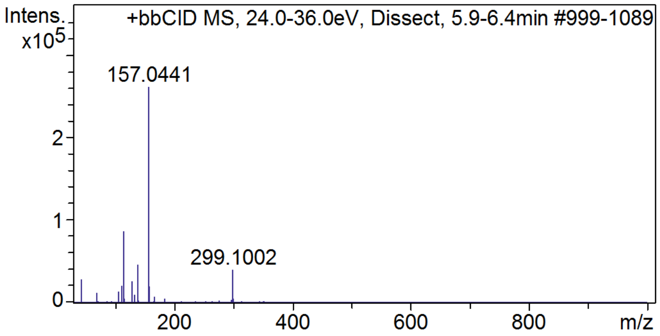

#	m/z	Res.	S/N	I	I %	FWHM
1	157.0442	26435	104.7	55934	10.6	0.0059
2	203.0846	30635	322.7	172470	32.6	0.0066
3	204.0880	28245	30.2	16143	3.1	0.0072
4	215.1107	28312	57.4	30669	5.8	0.0076
5	231.0785	30426	142.9	76377	14.4	0.0076
6	277.1189	34583	989.4	528799	100.0	0.0080
7	278.1225	31329	140.8	75264	14.2	0.0089
8	279.1243	28739	21.7	11625	2.2	0.0097
9	294.1450	32181	37.6	20082	3.8	0.0091
10	299.1003	33199	119.2	63719	12.0	0.0090

#	m/z	Res.	S/N	I	I %	FWHM
1	43.0160	15549	107.5	28666	10.9	0.0028
2	68.9945	17927	46.5	12394	4.7	0.0038
3	106.0616	21936	53.9	14378	5.5	0.0048
4	111.0039	22720	79.4	21173	8.1	0.0049
5	115.0350	23467	324.7	86585	33.1	0.0049
6	129.0139	24433	101.3	27016	10.3	0.0053
7	138.9978	25493	175.7	46848	17.9	0.0055
8	157.0441	28488	981.9	261830	100.0	0.0055
9	158.0475	25987	76.5	20397	7.8	0.0061
10	299.1002	34099	152.8	40749	15.6	0.0088

Cmpd 49, Dissect, 6.3 min

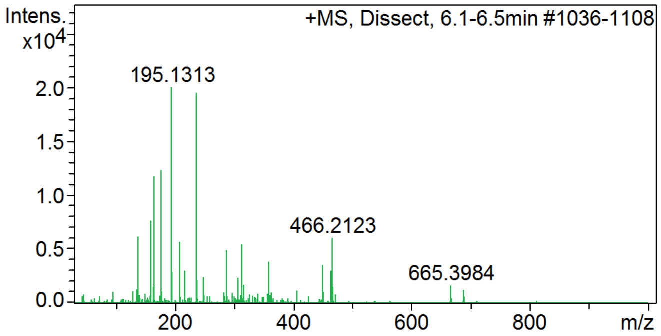

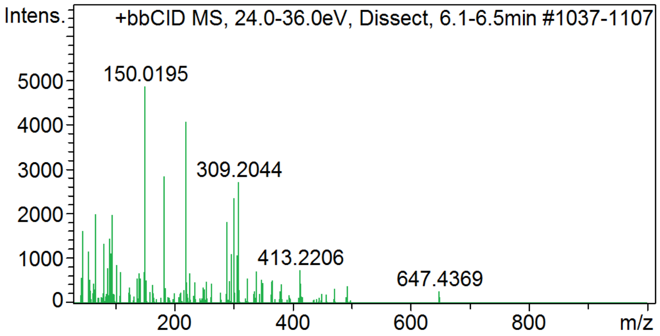

#	m/z	Res.	S/N	I	I %	FWHM
1	137.0915	24436	308.9	6199	30.9	0.0056
2	159.1113	26430	382.7	7680	38.3	0.0060
3	165.0425	26897	584.6	11730	58.5	0.0061
4	177.1214	27386	614.8	12337	61.5	0.0065
5	195.0950	27984	871.3	17484	87.2	0.0070
6	195.1313	28860	999.8	20062	100.0	0.0068
7	209.0738	28971	284.3	5705	28.4	0.0072
8	237.1406	30521	973.6	19535	97.4	0.0078
9	313.2381	30432	273.7	5491	27.4	0.0103
10	466.2123	30429	302.2	6063	30.2	0.0153

#	m/z	Res.	S/N	I	I %	FWHM
1	45.0316	15647	335.5	1632	33.6	0.0029
2	67.0518	17335	410.9	2000	41.1	0.0039
3	91.0512	19713	300.2	1461	30.0	0.0046
4	95.0824	20167	406.6	1978	40.7	0.0047
5	150.0195	24834	999.0	4861	100.0	0.0060
6	184.1634	27963	586.2	2852	58.7	0.0066
7	220.1703	31134	834.4	4060	83.5	0.0071
8	290.2013	32099	374.6	1823	37.5	0.0090
9	302.2044	30286	483.3	2352	48.4	0.0100
10	309.2044	30125	557.3	2712	55.8	0.0103

Compound Spectrum List Report

Cmpd 50, Dissect, 6.4 min

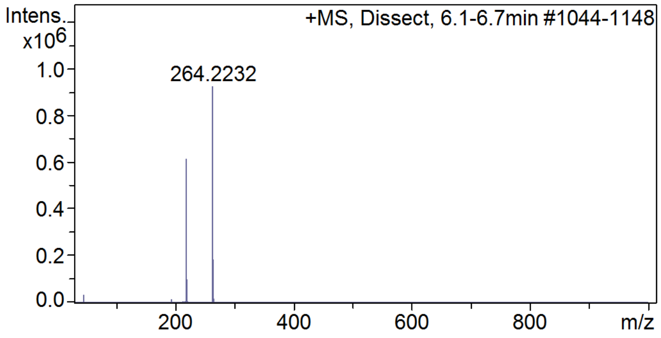

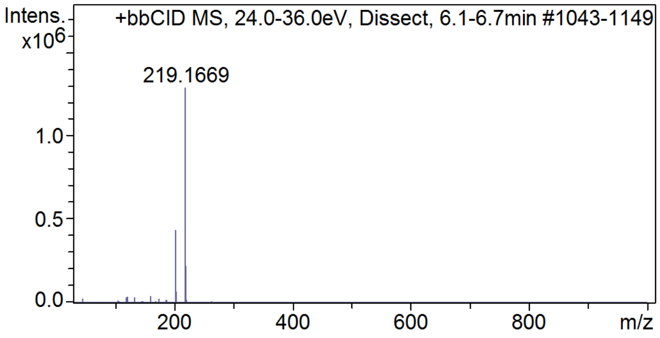

#	m/z	Res.	S/N	I	I %	FWHM
1	46.0632	15789	35.0	32885	3.6	0.0029
2	195.0950	27740	15.7	14779	1.6	0.0070
3	213.9920	29036	8.2	7718	0.8	0.0074
4	217.0761	28532	7.5	7025	0.8	0.0076
5	219.1670	32758	653.4	613352	66.4	0.0067
6	220.1703	29635	109.6	102880	11.1	0.0074
7	221.1734	26818	8.0	7554	0.8	0.0082
8	264.2232	35236	984.2	923913	100.0	0.0075
9	265.2268	31176	198.7	186507	20.2	0.0085
10	266.2298	28434	19.1	17904	1.9	0.0094

#	m/z	Res.	S/N	I	I %	FWHM
1	46.0632	15775	19.1	24809	1.9	0.0029
2	119.0815	23632	23.8	30787	2.4	0.0050
3	121.0970	23381	28.6	37035	2.9	0.0052
4	133.0965	24901	25.9	33574	2.6	0.0053
5	161.1269	27025	30.8	39865	3.1	0.0060
6	175.1420	27616	18.3	23679	1.8	0.0063
7	203.1361	31389	338.6	438882	34.0	0.0065
8	204.1395	29462	54.7	70884	5.5	0.0069
9	219.1669	33828	995.4	1290268	100.0	0.0065
10	220.1703	31065	173.2	224478	17.4	0.0071

Cmpd 51, Dissect, 6.4 min

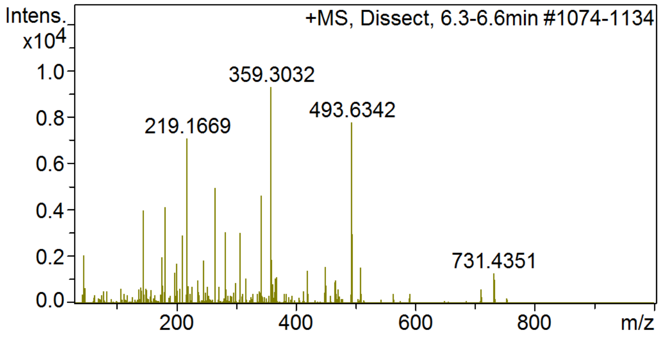

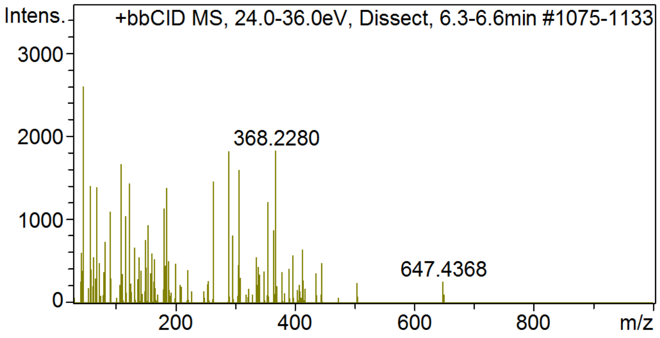

#	m/z	Res.	S/N	I	I %	FWHM
1	145.0599	24943	429.8	4000	43.0	0.0058
2	181.9666	28902	443.8	4131	44.4	0.0063
3	219.1669	32147	759.7	7071	76.0	0.0068
4	265.2267	30856	531.4	4946	53.1	0.0086
5	283.1649	27357	328.5	3057	32.8	0.0104
6	342.2522	29904	496.8	4623	49.7	0.0114
7	359.1916	29919	601.4	5597	60.1	0.0120
8	359.3032	32242	1000.0	9307	100.0	0.0111
9	493.6342	34807	832.9	7752	83.3	0.0142
10	493.9690	36375	616.9	5742	61.7	0.0136

#	m/z	Res.	S/N	I	I %	FWHM
1	46.0632	15767	998.7	2599	100.0	0.0029
2	58.0628	16901	540.6	1407	54.1	0.0034
3	69.0673	18065	533.9	1390	53.5	0.0038
4	109.0612	22121	641.6	1670	64.2	0.0049
5	123.9365	23420	551.4	1435	55.2	0.0053
6	186.1296	26491	532.4	1386	53.3	0.0070
7	264.2232	29804	560.8	1459	56.1	0.0089
8	290.2016	27270	698.8	1819	70.0	0.0106
9	307.2274	30820	613.5	1597	61.4	0.0100
10	368.2280	30057	701.4	1826	70.2	0.0123

Compound Spectrum List Report

Cmpd 52, Dissect, 6.6 min

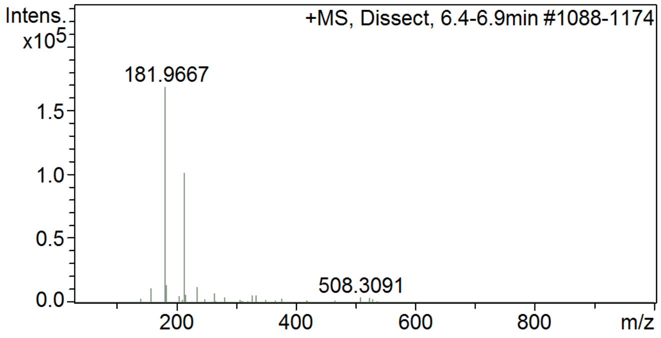

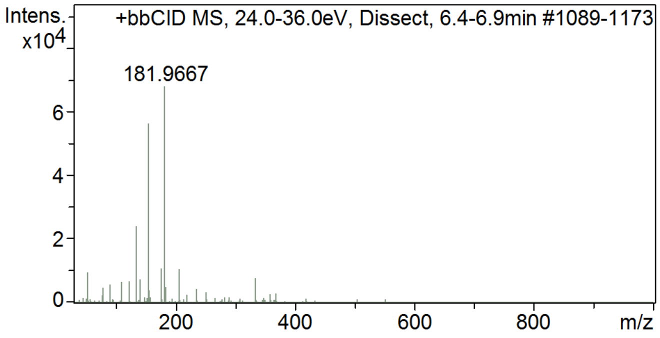

#	m/z	Res.	S/N	I	I %	FWHM
1	158.0217	26692	68.2	11510	6.8	0.0059
2	181.9667	29374	997.9	168528	100.0	0.0062
3	182.9696	24743	81.7	13793	8.2	0.0074
4	183.9625	27592	82.8	13978	8.3	0.0067
5	213.9919	30082	256.5	43310	25.7	0.0071
6	214.0825	31759	600.5	101419	60.2	0.0067
7	215.0855	27659	36.6	6188	3.7	0.0078
8	216.0785	27475	36.8	6219	3.7	0.0079
9	236.0639	30355	76.4	12906	7.7	0.0078
10	264.2233	31727	42.2	7126	4.2	0.0083

#	m/z	Res.	S/N	I	I %	FWHM
1	53.0364	17063	142.1	9725	14.3	0.0031
2	110.0023	22643	96.9	6629	9.7	0.0049
3	122.0017	23758	102.1	6989	10.3	0.0051
4	134.0013	25193	354.2	24244	35.6	0.0053
5	140.9958	25330	109.5	7494	11.0	0.0056
6	153.9727	26717	821.9	56251	82.7	0.0058
7	177.0822	30399	161.0	11020	16.2	0.0058
8	181.9667	28974	993.7	68008	100.0	0.0063
9	207.0919	32165	157.4	10769	15.8	0.0064
10	334.0065	32418	114.6	7846	11.5	0.0103

Cmpd 53, Dissect, 6.7 min

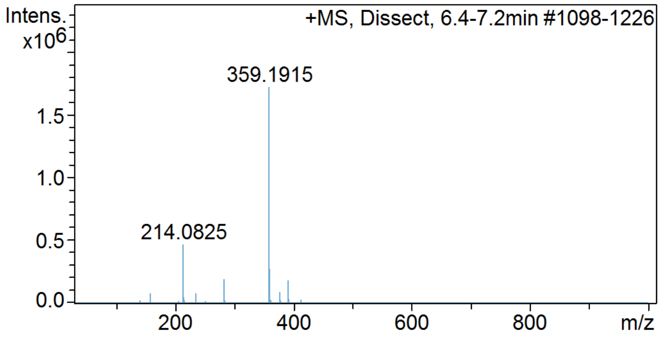

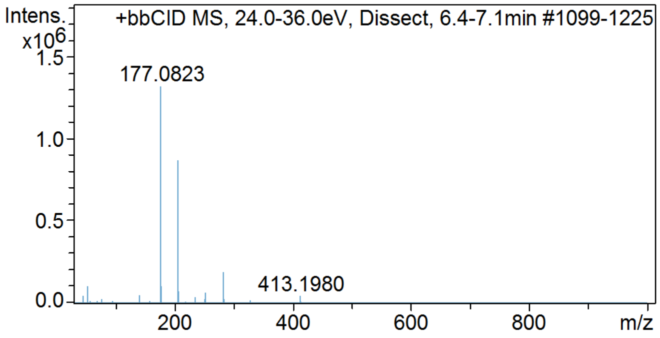

#	m/z	Res.	S/N	I	I %	FWHM
1	158.0217	26844	46.9	81404	4.7	0.0059
2	214.0825	32436	269.8	468695	27.2	0.0066
3	215.0856	27694	28.2	49011	2.8	0.0078
4	236.0638	30777	48.0	83352	4.8	0.0077
5	283.1420	33345	113.6	197303	11.5	0.0085
6	359.1915	41412	991.3	1722256	100.0	0.0087
7	360.1946	34822	160.9	279617	16.2	0.0103
8	377.2026	29720	54.9	95337	5.5	0.0127
9	391.2169	34119	107.0	185936	10.8	0.0115
10	392.2198	30170	19.3	33546	1.9	0.0130

#	m/z	Res.	S/N	I	I %	FWHM
1	45.0316	15684	34.0	46149	3.5	0.0029
2	53.0364	17129	79.7	108269	8.2	0.0031
3	140.9958	25271	36.3	49263	3.7	0.0056
4	177.0823	30881	969.9	1316753	100.0	0.0057
5	178.0852	25962	79.1	107416	8.2	0.0069
6	207.0919	32616	638.1	866244	65.8	0.0063
7	208.0948	26989	56.6	76887	5.8	0.0077
8	253.1324	31463	49.5	67233	5.1	0.0080
9	283.1420	34094	140.8	191169	14.5	0.0083
10	413.1980	32560	34.0	46195	3.5	0.0127

Compound Spectrum List Report

Cmpd 54, Dissect, 6.8 min

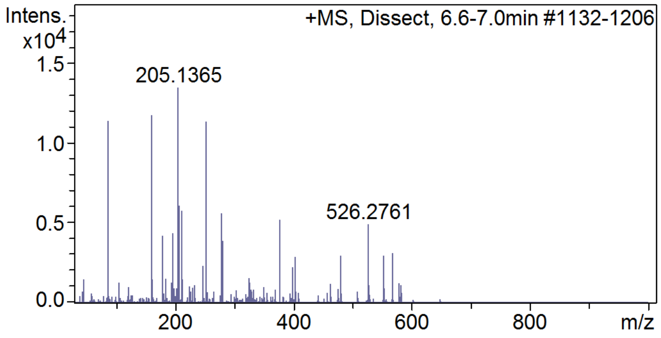

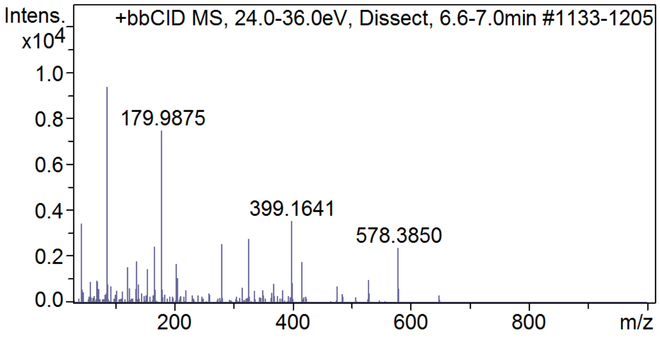

#	m/z	Res.	S/N	I	I %	FWHM
1	87.0411	19500	840.4	11371	84.3	0.0045
2	161.1270	26583	865.9	11716	86.9	0.0061
3	205.1365	28718	996.6	13485	100.0	0.0071
4	207.9817	28436	450.6	6097	45.2	0.0073
5	212.0128	28065	419.5	5676	42.1	0.0076
6	212.1574	29095	425.5	5757	42.7	0.0073
7	253.0381	29948	835.4	11304	83.8	0.0084
8	279.0529	30171	415.7	5624	41.7	0.0092
9	377.2028	32167	385.9	5221	38.7	0.0117
10	526.2761	34422	364.1	4926	36.5	0.0153

#	m/z	Res.	S/N	I	I %	FWHM
1	43.0160	15531	368.4	3453	36.9	0.0028
2	87.0411	19510	999.4	9367	100.0	0.0045
3	136.0170	24711	193.7	1816	19.4	0.0055
4	167.9878	27251	260.5	2441	26.1	0.0062
5	179.9875	27825	795.2	7452	79.6	0.0065
6	281.1058	31978	274.0	2568	27.4	0.0088
7	326.2192	30559	296.4	2778	29.7	0.0107
8	399.1641	29956	381.0	3570	38.1	0.0133
9	416.3379	32321	190.5	1785	19.1	0.0129
10	578.3850	31442	255.4	2393	25.5	0.0184

Cmpd 55, Dissect, 6.9 min

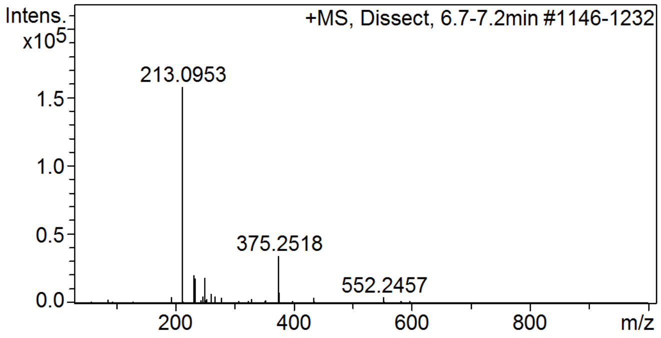

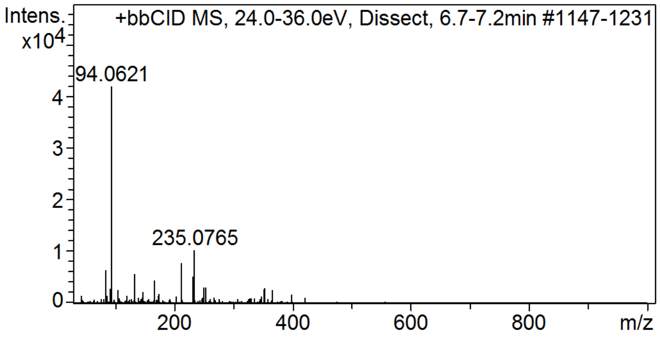

#	m/z	Res.	S/N	I	I %	FWHM
1	213.0953	29911	996.2	157819	100.0	0.0071
2	232.1621	29916	131.0	20750	13.1	0.0078
3	233.1458	30699	89.7	14215	9.0	0.0076
4	235.0764	29309	117.0	18534	11.7	0.0080
5	248.0118	30328	29.6	4686	3.0	0.0082
6	251.1560	30957	117.7	18643	11.8	0.0081
7	262.0270	30105	46.1	7306	4.6	0.0087
8	268.1813	29707	29.9	4735	3.0	0.0090
9	375.2518	33580	218.6	34637	21.9	0.0112
10	376.2549	30121	51.1	8095	5.1	0.0125

#	m/z	Res.	S/N	I	I %	FWHM
1	84.0779	19105	154.0	6468	15.4	0.0044
2	94.0621	19727	998.7	41949	100.0	0.0048
3	133.0967	24540	135.8	5704	13.6	0.0054
4	167.9880	26867	106.8	4485	10.7	0.0063
5	213.0953	28319	188.1	7903	18.8	0.0075
6	232.1622	30884	125.0	5251	12.5	0.0075
7	235.0765	28823	245.4	10309	24.6	0.0082
8	251.0498	30143	75.0	3152	7.5	0.0083
9	254.1643	29158	75.8	3182	7.6	0.0087
10	353.2375	29100	70.9	2979	7.1	0.0121

Compound Spectrum List Report

Cmpd 56, Dissect, 7.1 min

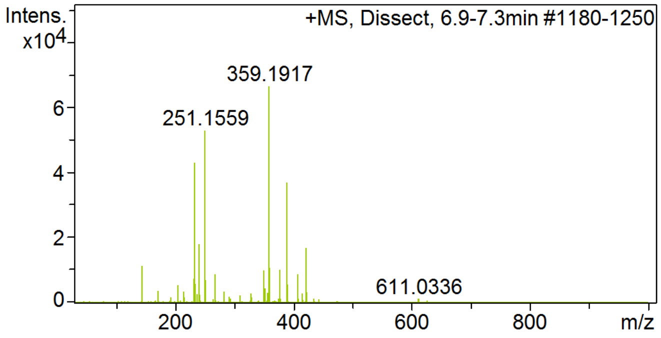

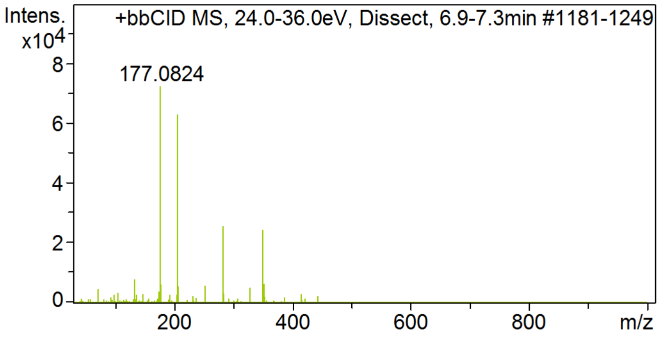

#	m/z	Res.	S/N	I	I %	FWHM
1	144.1334	25594	171.3	11395	17.1	0.0056
2	233.1459	31072	646.3	42996	64.7	0.0075
3	241.1176	30136	272.0	18095	27.2	0.0080
4	251.1559	31037	794.0	52823	79.5	0.0081
5	350.2185	32042	150.7	10028	15.1	0.0109
6	359.1917	33920	999.1	66474	100.0	0.0106
7	360.1944	29488	164.7	10958	16.5	0.0122
8	377.2024	37621	155.6	10351	15.6	0.0100
9	389.2010	33152	556.6	37028	55.7	0.0117
10	421.2266	31890	255.7	17009	25.6	0.0132

#	m/z	Res.	S/N	I	I %	FWHM
1	133.0967	24811	109.1	7893	10.9	0.0054
2	177.0824	28582	999.3	72273	100.0	0.0062
3	178.0851	22724	87.7	6346	8.8	0.0078
4	207.0920	30391	866.7	62682	86.7	0.0068
5	208.0949	24031	79.2	5730	7.9	0.0087
6	253.1322	29974	79.9	5778	8.0	0.0084
7	283.1421	32699	355.6	25719	35.6	0.0087
8	329.1821	30537	71.4	5160	7.1	0.0108
9	350.2184	32704	336.7	24348	33.7	0.0107
10	352.2340	34147	88.9	6431	8.9	0.0103

Cmpd 57, Dissect, 7.1 min

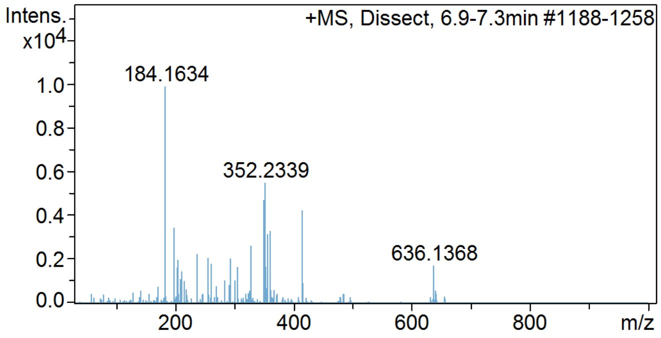

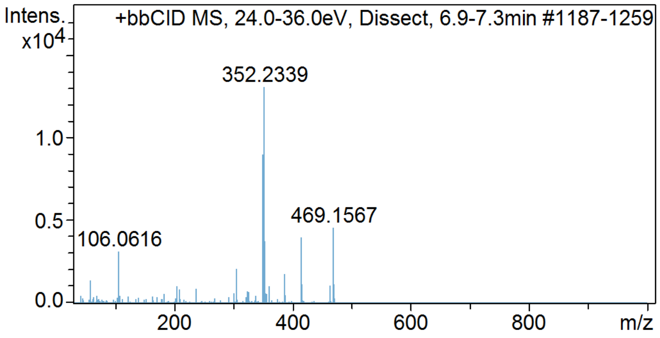

#	m/z	Res.	S/N	I	I %	FWHM
1	184.1634	27426	999.8	9899	100.0	0.0067
2	199.1164	27228	349.2	3457	34.9	0.0073
3	238.1711	28591	229.1	2269	22.9	0.0083
4	256.0821	28977	210.8	2087	21.1	0.0088
5	328.2373	31162	267.8	2651	26.8	0.0105
6	350.2185	32244	477.2	4724	47.7	0.0109
7	352.2339	32768	555.8	5503	55.6	0.0107
8	356.2678	30527	319.9	3167	32.0	0.0117
9	361.2096	30538	334.8	3314	33.5	0.0118
10	415.1876	31702	429.6	4253	43.0	0.0131

#	m/z	Res.	S/N	I	I %	FWHM
1	58.0628	17230	107.9	1411	10.8	0.0034
2	106.0616	22046	240.9	3153	24.1	0.0048
3	306.1939	29909	159.5	2086	15.9	0.0102
4	350.2183	32311	687.4	8995	68.8	0.0108
5	351.2214	30834	174.3	2281	17.4	0.0114
6	352.2339	34128	999.8	13083	100.0	0.0103
7	353.2372	30693	288.0	3769	28.8	0.0115
8	387.1570	28927	138.1	1807	13.8	0.0134
9	415.1877	31664	306.3	4008	30.6	0.0131
10	469.1567	31667	350.5	4586	35.1	0.0148

Compound Spectrum List Report

Cmpd 58, Dissect, 7.2 min

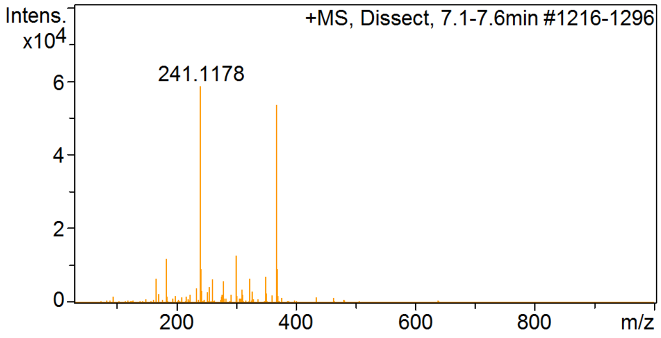

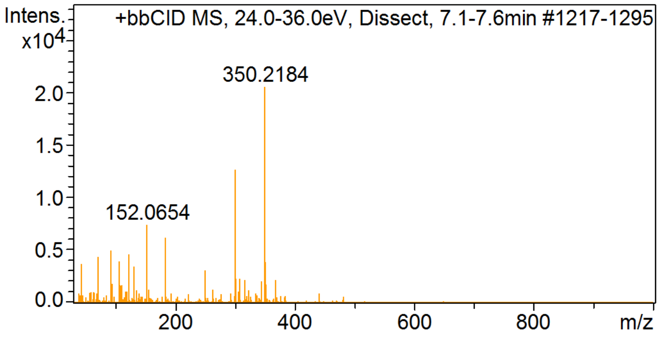

#	m/z	Res.	S/N	I	I %	FWHM
1	167.1011	26626	112.7	6603	11.3	0.0063
2	184.1634	27531	205.2	12028	20.5	0.0067
3	241.1178	31216	999.5	58580	100.0	0.0077
4	242.1210	29301	157.9	9257	15.8	0.0083
5	261.1764	30147	111.5	6532	11.2	0.0087
6	301.1676	30376	219.4	12859	22.0	0.0099
7	323.0778	31737	112.5	6592	11.3	0.0102
8	350.2186	32449	123.4	7233	12.3	0.0108
9	368.0754	33659	913.1	53518	91.4	0.0109
10	369.0784	29913	158.1	9267	15.8	0.0123

#	m/z	Res.	S/N	I	I %	FWHM
1	43.0160	15459	181.8	3743	18.2	0.0028
2	71.0465	17893	214.5	4415	21.5	0.0040
3	92.0465	19871	243.7	5015	24.4	0.0046
4	106.0616	22011	192.1	3955	19.2	0.0048
5	122.0560	23532	226.4	4660	22.7	0.0052
6	152.0654	25581	360.2	7415	36.1	0.0059
7	184.0366	28739	301.8	6213	30.2	0.0064
8	301.1675	30266	615.5	12669	61.6	0.0100
9	350.2184	32188	999.2	20567	100.0	0.0109
10	351.2216	31115	190.6	3923	19.1	0.0113

Cmpd 59, Dissect, 7.4 min

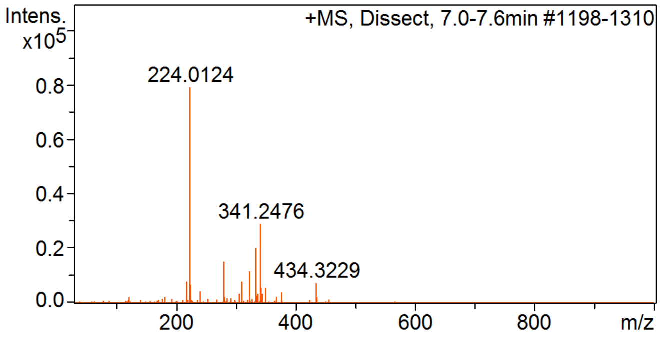

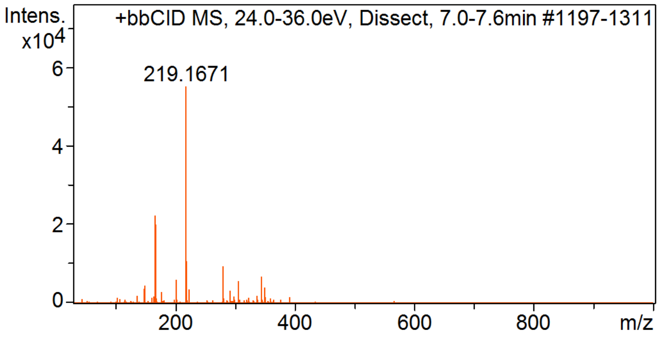

#	m/z	Res.	S/N	I	I %	FWHM
1	219.1670	28655	101.6	8046	10.2	0.0076
2	224.0124	29578	999.3	79108	100.0	0.0076
3	225.0153	27080	87.6	6933	8.8	0.0083
4	281.1056	31610	193.6	15325	19.4	0.0089
5	310.2274	31752	101.6	8045	10.2	0.0098
6	323.0777	31269	149.9	11864	15.0	0.0103
7	334.2265	31877	254.3	20127	25.4	0.0105
8	341.2476	31970	367.6	29100	36.8	0.0107
9	342.2508	30231	72.3	5722	7.2	0.0113
10	434.3229	30874	96.9	7668	9.7	0.0141

#	m/z	Res.	S/N	I	I %	FWHM
1	149.0183	25782	84.1	4641	8.4	0.0058
2	166.9802	26943	405.5	22379	40.6	0.0062
3	167.9878	26296	364.6	20121	36.5	0.0064
4	203.1363	28022	110.6	6102	11.1	0.0072
5	219.1671	29896	999.3	55150	100.0	0.0073
6	220.1704	29012	197.3	10890	19.7	0.0076
7	281.1056	31512	171.1	9440	17.1	0.0089
8	306.1937	30917	104.0	5741	10.4	0.0099
9	345.0587	30979	125.0	6898	12.5	0.0111
10	350.2184	32171	73.8	4075	7.4	0.0109

Compound Spectrum List Report

Cmpd 60, Dissect, 7.4 min

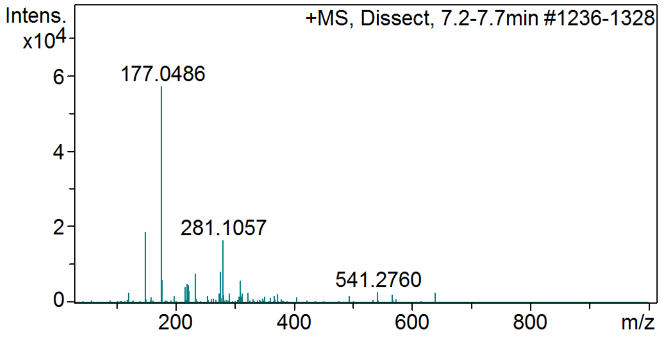

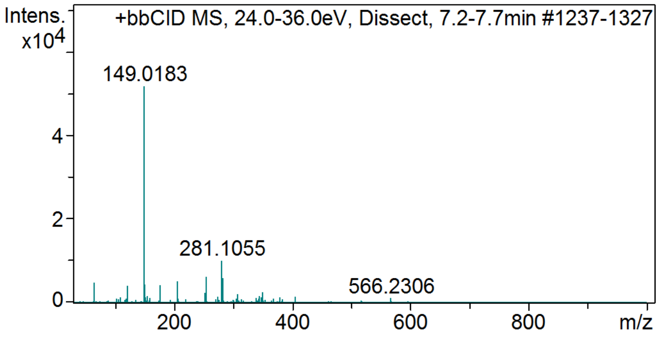

#	m/z	Res.	S/N	I	I %	FWHM
1	149.0183	25524	328.2	18761	32.8	0.0058
2	177.0486	27828	999.7	57154	100.0	0.0064
3	178.0519	27750	106.2	6070	10.6	0.0064
4	217.1515	28092	75.8	4336	7.6	0.0077
5	221.0671	28087	90.3	5165	9.0	0.0079
6	223.0891	28496	84.9	4855	8.5	0.0078
7	235.1614	29847	135.8	7764	13.6	0.0079
8	277.1706	31018	146.7	8390	14.7	0.0089
9	281.1057	32208	290.8	16628	29.1	0.0087
10	310.2274	31466	103.8	5935	10.4	0.0099

#	m/z	Res.	S/N	I	I %	FWHM
1	65.0363	17046	96.9	5014	9.7	0.0038
2	121.0244	22936	81.5	4216	8.2	0.0053
3	149.0183	26138	999.9	51714	100.0	0.0057
4	149.0911	26008	71.1	3679	7.1	0.0057
5	150.0215	25757	85.7	4433	8.6	0.0058
6	177.0826	26256	85.3	4410	8.5	0.0067
7	207.0922	28175	102.6	5308	10.3	0.0074
8	255.0553	28947	122.0	6312	12.2	0.0088
9	281.1055	31875	194.4	10057	19.4	0.0088
10	283.1422	30649	117.5	6078	11.8	0.0092

Cmpd 61, Dissect, 7.6 min

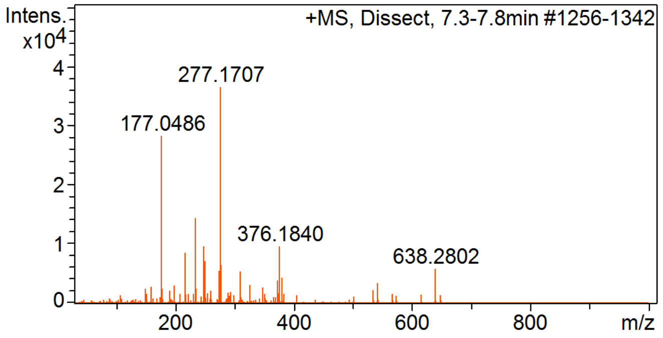

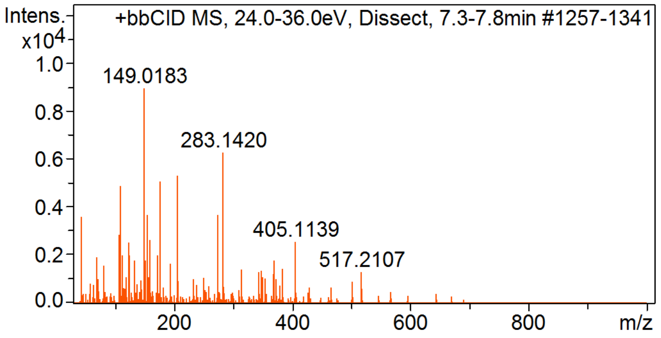

#	m/z	Res.	S/N	I	I %	FWHM
1	177.0486	27426	772.2	28247	77.2	0.0065
2	217.1513	28675	234.9	8594	23.5	0.0076
3	235.1614	30147	394.1	14414	39.4	0.0078
4	249.1403	30551	265.4	9707	26.5	0.0082
5	251.0620	30039	195.5	7152	19.6	0.0084
6	275.1529	30391	152.2	5566	15.2	0.0091
7	277.1707	32057	999.9	36575	100.0	0.0086
8	278.1739	28537	177.9	6508	17.8	0.0097
9	376.1840	31452	265.4	9708	26.5	0.0120
10	638.2802	32072	159.8	5844	16.0	0.0199

#	m/z	Res.	S/N	I	I %	FWHM
1	43.0160	15547	401.2	3598	40.1	0.0028
2	107.0820	21943	319.5	2865	32.0	0.0049
3	109.0612	22318	544.4	4881	54.4	0.0049
4	109.0976	21732	314.9	2824	31.5	0.0050
5	149.0183	25748	999.9	8966	100.0	0.0058
6	155.0649	26320	410.6	3682	41.1	0.0059
7	177.0828	25827	564.7	5064	56.5	0.0069
8	207.0923	27905	592.2	5310	59.2	0.0074
9	275.1528	29778	409.9	3676	41.0	0.0092
10	283.1420	30914	699.0	6268	69.9	0.0092

Compound Spectrum List Report

Cmpd 62, Dissect, 7.6 min

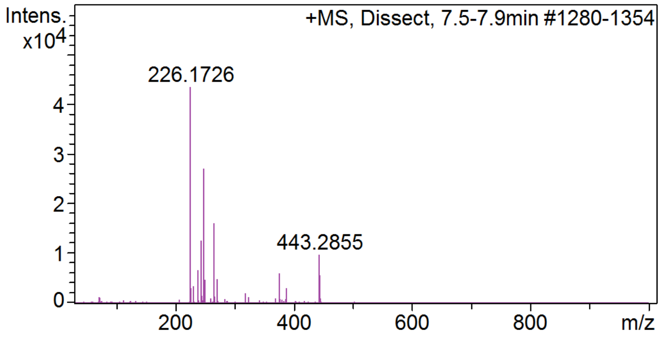

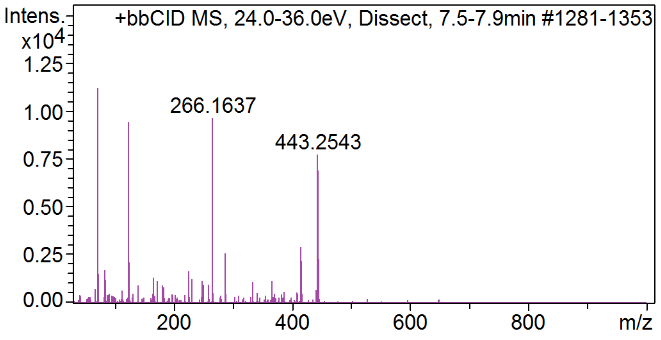

#	m/z	Res.	S/N	I	I %	FWHM
1	226.0280	28856	186.3	8118	18.6	0.0078
2	226.1726	30180	1000.0	43571	100.0	0.0075
3	239.0228	29600	153.5	6690	15.4	0.0081
4	243.9838	29942	146.0	6360	14.6	0.0081
5	244.1826	30092	289.6	12618	29.0	0.0081
6	249.1403	30243	620.8	27049	62.1	0.0082
7	266.1639	30599	371.6	16190	37.2	0.0087
8	376.1841	30985	139.1	6061	13.9	0.0121
9	443.2855	31743	224.3	9773	22.4	0.0140
10	444.2854	29321	132.7	5781	13.3	0.0152

#	m/z	Res.	S/N	I	I %	FWHM
1	72.0416	18854	999.2	11226	100.0	0.0038
2	123.0400	23067	839.8	9436	84.1	0.0053
3	266.1637	30790	857.1	9630	85.8	0.0086
4	443.1253	40756	336.0	3775	33.6	0.0109
5	443.1610	33774	544.0	6112	54.4	0.0131
6	443.2260	31662	663.7	7457	66.4	0.0140
7	443.2543	31740	687.6	7725	68.8	0.0140
8	444.1884	37858	332.6	3736	33.3	0.0117
9	444.2252	36962	614.6	6905	61.5	0.0120
10	444.2581	34246	327.2	3676	32.7	0.0130

Cmpd 63, Dissect, 7.7 min

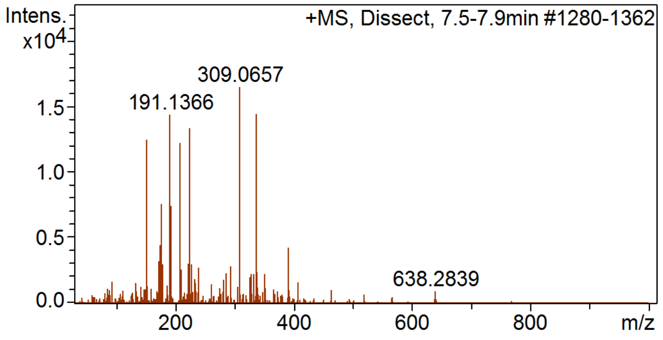

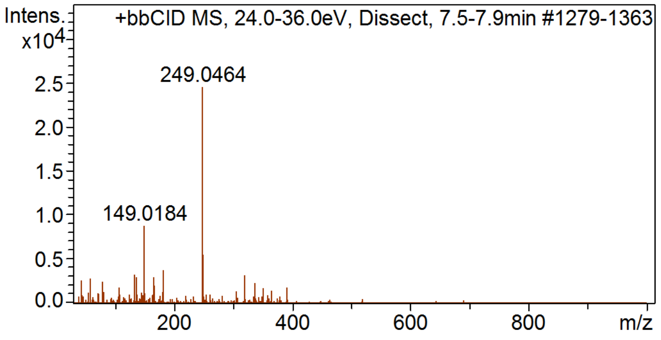

#	m/z	Res.	S/N	I	I %	FWHM
1	151.1066	25591	756.1	12462	75.6	0.0059
2	175.1423	27279	270.7	4463	27.1	0.0064
3	177.0487	26602	458.4	7555	45.9	0.0067
4	191.1366	27847	870.0	14341	87.0	0.0069
5	193.1521	27778	449.4	7408	45.0	0.0070
6	209.1466	29037	740.8	12211	74.1	0.0072
7	225.1310	29693	808.9	13333	80.9	0.0076
8	309.0657	31961	999.6	16477	100.0	0.0097
9	337.0929	31331	874.7	14417	87.5	0.0108
10	391.0991	30154	258.4	4259	25.8	0.0130

#	m/z	Res.	S/N	I	I %	FWHM
1	133.0966	24520	134.3	3308	13.5	0.0054
2	136.0168	24983	120.8	2976	12.1	0.0054
3	149.0184	23966	356.4	8780	35.7	0.0062
4	149.0909	26640	264.0	6504	26.5	0.0056
5	166.0594	26288	121.8	3000	12.2	0.0063
6	183.0855	24943	155.3	3825	15.6	0.0073
7	249.0464	31189	997.6	24576	100.0	0.0080
8	250.0497	29976	225.4	5553	22.6	0.0083
9	250.1698	28788	116.8	2879	11.7	0.0087
10	320.0673	29762	130.1	3204	13.0	0.0108

Compound Spectrum List Report

Cmpd 64, Dissect, 7.7 min

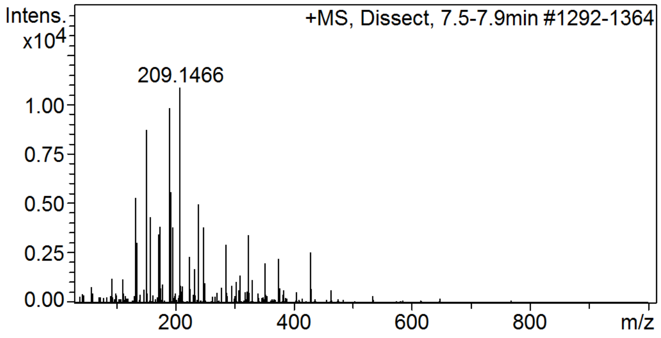

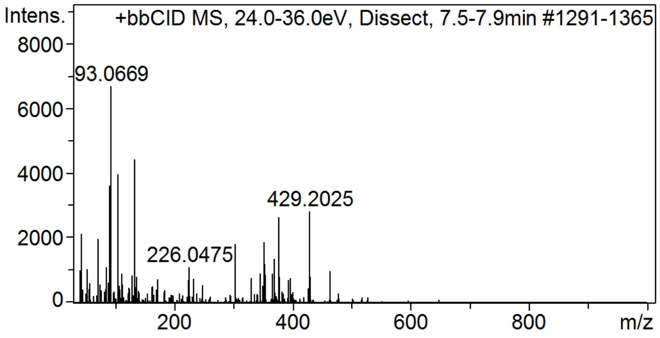

#	m/z	Res.	S/N	I	I %	FWHM
1	133.0967	24467	486.9	5292	48.7	0.0054
2	151.1066	25725	801.2	8708	80.2	0.0059
3	158.1485	26392	397.4	4319	39.8	0.0060
4	175.1422	27516	355.0	3858	35.5	0.0064
5	191.1366	27919	901.2	9796	90.2	0.0068
6	193.1521	27807	512.9	5575	51.3	0.0069
7	197.0895	27977	349.9	3803	35.0	0.0070
8	209.1466	29260	999.3	10861	100.0	0.0071
9	240.1878	30646	456.4	4961	45.7	0.0078
10	249.1402	29162	350.3	3808	35.1	0.0085

#	m/z	Res.	S/N	I	I %	FWHM
1	43.0160	15582	320.0	2140	32.0	0.0028
2	71.0466	18164	295.4	1976	29.5	0.0039
3	91.0513	20111	540.6	3615	54.1	0.0045
4	93.0669	19738	999.8	6686	100.0	0.0047
5	105.0664	21643	595.0	3979	59.5	0.0049
6	133.0966	24519	661.0	4420	66.1	0.0054
7	304.1779	30728	272.2	1820	27.2	0.0099
8	352.2336	31902	282.3	1888	28.2	0.0110
9	377.1801	30938	397.3	2657	39.7	0.0122
10	429.2025	30369	421.6	2820	42.2	0.0141

Cmpd 65, Dissect, 7.8 min

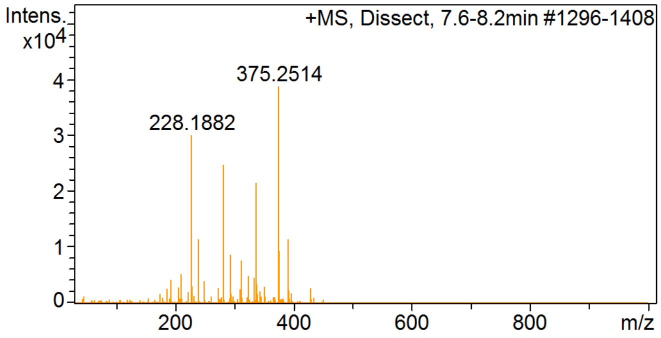

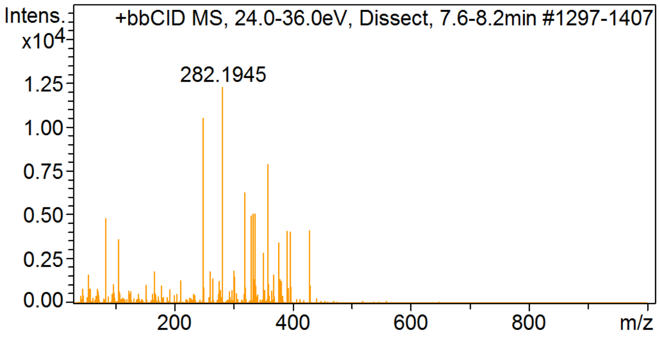

#	m/z	Res.	S/N	I	I %	FWHM
1	211.1621	28776	137.4	5323	13.7	0.0073
2	228.1882	30599	773.2	29959	77.3	0.0075
3	240.1878	30816	296.5	11487	29.7	0.0078
4	282.1949	30532	637.9	24716	63.8	0.0092
5	294.2328	31190	224.3	8689	22.4	0.0094
6	312.2430	30677	199.4	7727	19.9	0.0102
7	337.0927	34247	556.8	21572	55.7	0.0098
8	375.2514	33113	999.8	38736	100.0	0.0113
9	376.2549	30554	242.0	9377	24.2	0.0123
10	391.0988	31748	297.5	11527	29.8	0.0123

#	m/z	Res.	S/N	I	I %	FWHM
1	84.0778	18979	392.9	4825	39.3	0.0044
2	250.1694	28669	853.6	10483	85.4	0.0087
3	282.1945	30665	999.7	12277	100.0	0.0092
4	320.0667	32507	513.4	6305	51.4	0.0098
5	331.2631	30646	404.7	4970	40.5	0.0108
6	334.2240	30483	415.1	5097	41.5	0.0110
7	337.0924	32768	415.4	5101	41.6	0.0103
8	359.0737	31986	642.6	7892	64.3	0.0112
9	391.0983	30171	334.6	4108	33.5	0.0130
10	429.2027	30446	339.5	4170	34.0	0.0141

Compound Spectrum List Report

Cmpd 66, Dissect, 8.0 min

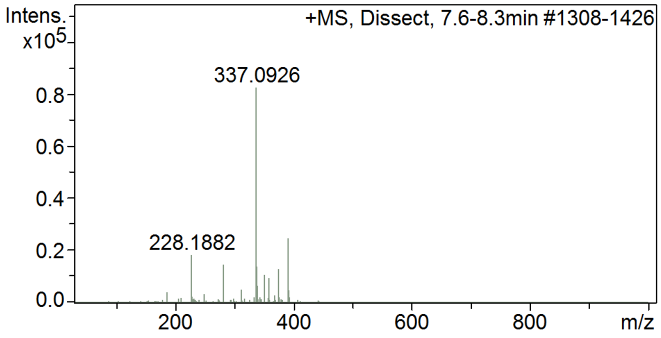

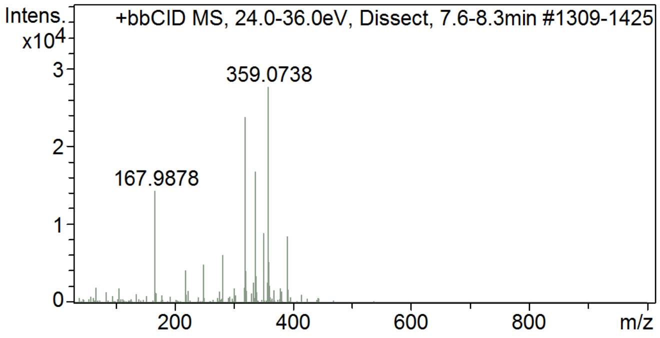

#	m/z	Res.	S/N	I	I %	FWHM
1	228.1882	30199	224.1	18498	22.4	0.0076
2	282.1954	30215	178.9	14769	17.9	0.0093
3	312.2428	30362	64.0	5282	6.4	0.0103
4	337.0926	34356	999.9	82534	100.0	0.0098
5	338.0956	30352	171.2	14131	17.1	0.0111
6	339.0890	26270	80.8	6671	8.1	0.0129
7	351.1077	30864	130.8	10797	13.1	0.0114
8	359.0737	31589	116.4	9604	11.6	0.0114
9	375.2513	31971	159.0	13124	15.9	0.0117
10	391.0987	31809	301.6	24892	30.2	0.0123

#	m/z	Res.	S/N	I	I %	FWHM
1	167.9878	26988	518.8	14382	51.9	0.0062
2	219.1670	28654	153.0	4241	15.3	0.0076
3	250.1696	28598	178.5	4949	17.9	0.0087
4	282.1944	30644	221.4	6139	22.2	0.0092
5	320.0667	32908	857.4	23770	85.8	0.0097
6	337.0926	33673	605.8	16793	60.6	0.0100
7	351.1077	31416	321.8	8921	32.2	0.0112
8	359.0738	32377	999.6	27710	100.0	0.0111
9	360.0764	30606	191.3	5304	19.1	0.0118
10	391.0985	31272	309.1	8568	30.9	0.0125

Cmpd 67, Dissect, 8.1 min

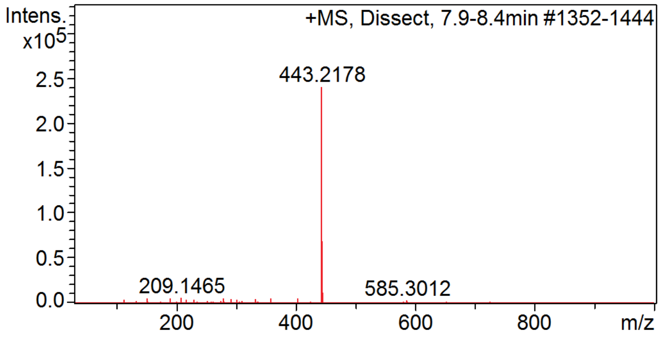

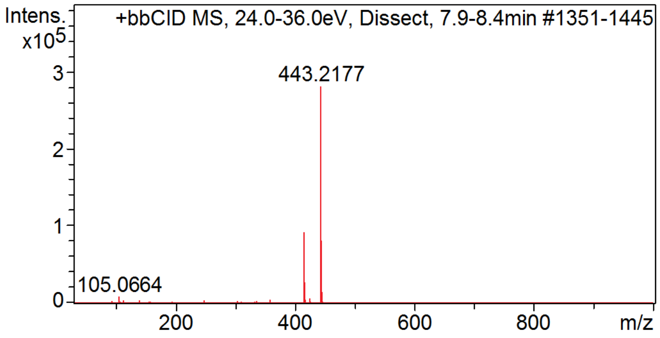

#	m/z	Res.	S/N	I	I %	FWHM
1	151.0913	25501	22.1	5323	2.2	0.0059
2	191.1365	28444	20.8	5009	2.1	0.0067
3	209.1465	28377	24.1	5794	2.4	0.0074
4	279.1950	25019	20.8	5012	2.1	0.0112
5	292.2173	31355	19.8	4769	2.0	0.0093
6	359.0739	31423	22.8	5480	2.3	0.0114
7	403.1878	29796	20.7	4989	2.1	0.0135
8	443.2178	37827	999.9	240498	100.0	0.0117
9	444.2209	32269	288.2	69315	28.8	0.0138
10	445.2239	30653	52.5	12631	5.3	0.0145

#	m/z	Res.	S/N	I	I %	FWHM
1	105.0664	21611	30.6	8610	3.1	0.0049
2	138.9978	25230	14.4	4047	1.4	0.0055
3	359.0737	32023	17.7	4970	1.8	0.0112
4	415.1873	35336	328.6	92482	32.9	0.0117
5	416.1904	32056	98.2	27632	9.8	0.0130
6	417.1935	27495	14.8	4178	1.5	0.0152
7	425.1688	31028	23.2	6520	2.3	0.0137
8	443.2177	38301	997.9	280879	100.0	0.0116
9	444.2208	33715	290.6	81798	29.1	0.0132
10	445.2237	30308	54.2	15251	5.4	0.0147

Compound Spectrum List Report

Cmpd 68, Dissect, 8.1 min

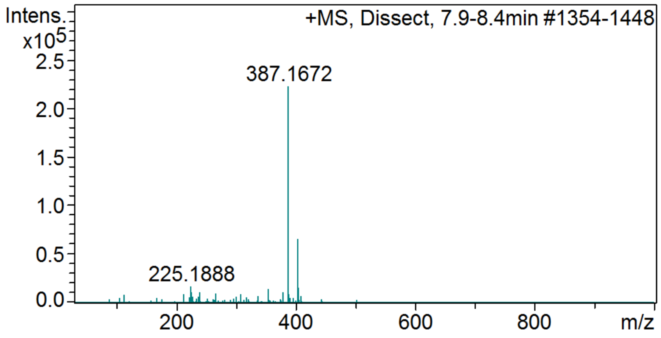

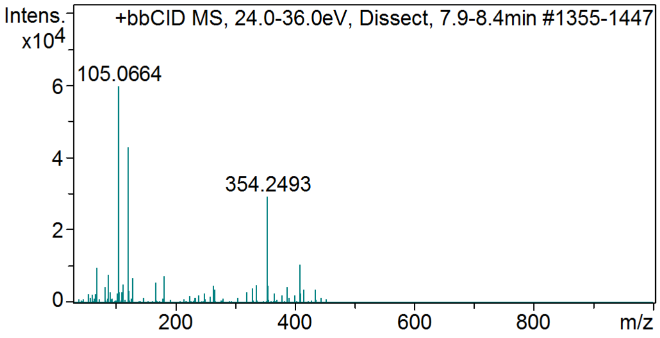

#	m/z	Res.	S/N	I	I %	FWHM
1	225.1888	28309	77.4	17240	7.7	0.0080
2	226.1150	29076	51.4	11462	5.1	0.0078
3	240.0939	29356	51.5	11470	5.2	0.0082
4	267.1139	29407	45.7	10189	4.6	0.0091
5	354.2497	30080	66.2	14744	6.6	0.0118
6	379.0635	31128	51.5	11480	5.2	0.0122
7	387.1672	35403	999.3	222631	100.0	0.0109
8	388.1705	34252	247.3	55102	24.8	0.0113
9	404.1931	33681	296.8	66128	29.7	0.0120
10	405.1964	30616	70.8	15770	7.1	0.0132

#	m/z	Res.	S/N	I	I %	FWHM
1	69.0308	18088	162.8	9723	16.3	0.0038
2	88.0184	19424	130.5	7789	13.0	0.0045
3	105.0664	21627	999.8	59695	100.0	0.0049
4	121.0607	22884	717.5	42840	71.8	0.0053
5	121.0971	22709	112.7	6727	11.3	0.0053
6	129.0496	22199	115.4	6891	11.5	0.0058
7	167.9878	27153	97.2	5801	9.7	0.0062
8	182.0539	27730	126.3	7539	12.6	0.0066
9	354.2493	31567	490.8	29307	49.1	0.0112
10	409.1480	30913	177.5	10595	17.7	0.0132

Cmpd 69, Dissect, 8.3 min

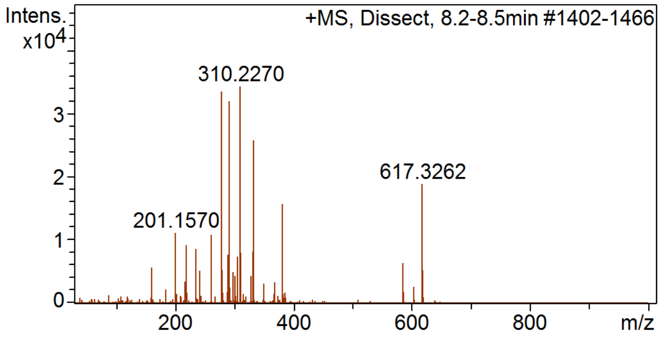

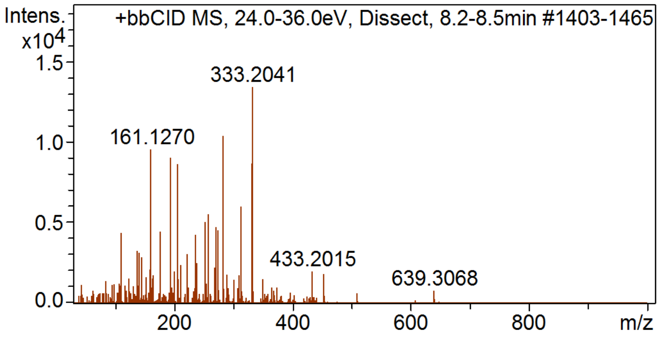

#	m/z	Res.	S/N	I	I %	FWHM
1	201.1570	27888	326.8	11209	32.7	0.0072
2	219.1670	28200	269.1	9231	26.9	0.0078
3	236.1930	29650	253.3	8688	25.3	0.0080
4	262.1714	29963	317.8	10901	31.8	0.0087
5	279.1974	30924	975.8	33472	97.6	0.0090
6	292.2173	32273	930.6	31920	93.1	0.0091
7	310.2270	31659	999.5	34284	100.0	0.0098
8	333.2037	29424	749.3	25701	75.0	0.0113
9	382.0469	31106	457.6	15696	45.8	0.0123
10	617.3262	31474	548.9	18827	54.9	0.0196

#	m/z	Res.	S/N	I	I %	FWHM
1	161.1270	26616	709.9	9536	71.0	0.0061
2	195.0926	26206	671.3	9017	67.2	0.0074
3	207.0921	26017	642.9	8635	64.3	0.0080
4	253.1325	30102	377.2	5067	37.7	0.0084
5	258.1742	29374	413.8	5558	41.4	0.0088
6	271.1420	28602	352.5	4735	35.3	0.0095
7	283.1419	31321	772.9	10382	77.3	0.0090
8	313.1515	29310	446.7	6001	44.7	0.0107
9	332.2084	31742	644.4	8656	64.5	0.0105
10	333.2041	25559	999.6	13426	100.0	0.0130

Compound Spectrum List Report

Cmpd 70, Dissect, 8.4 min

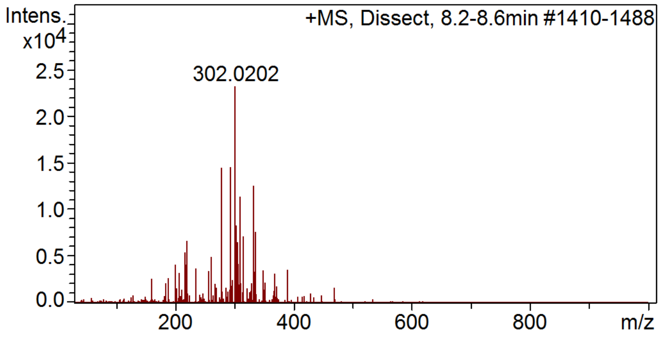

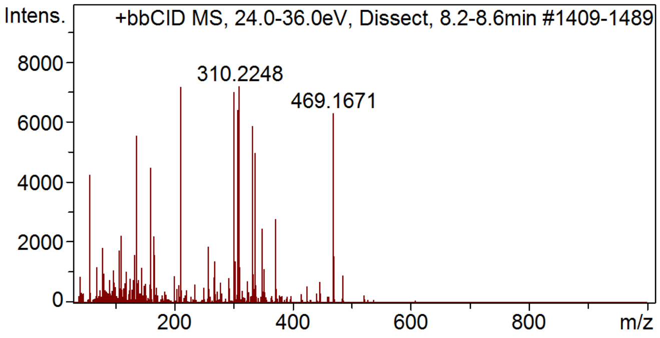

#	m/z	Res.	S/N	I	I %	FWHM
1	221.1463	28817	288.1	6697	28.8	0.0077
2	279.1973	30283	621.9	14458	62.2	0.0092
3	294.2328	31808	626.7	14569	62.7	0.0093
4	302.0202	32273	999.0	23226	100.0	0.0094
5	304.0355	30020	356.5	8288	35.7	0.0101
6	306.0514	31388	280.6	6524	28.1	0.0098
7	310.2269	31257	489.4	11379	49.0	0.0099
8	315.2539	29236	307.5	7149	30.8	0.0108
9	333.2035	28448	539.1	12533	54.0	0.0117
10	336.2396	32235	327.7	7619	32.8	0.0104

#	m/z	Res.	S/N	I	I %	FWHM
1	57.0676	17101	589.3	4238	59.0	0.0033
2	136.0471	25032	770.3	5539	77.1	0.0054
3	161.1270	26325	624.2	4489	62.5	0.0061
4	212.1938	28546	996.8	7168	99.7	0.0074
5	302.0203	30764	973.7	7002	97.4	0.0098
6	308.2098	27935	887.0	6379	88.8	0.0110
7	310.2248	30267	999.4	7187	100.0	0.0102
8	333.2039	25715	813.7	5851	81.4	0.0130
9	337.2411	26596	691.9	4975	69.2	0.0127
10	469.1671	32310	872.6	6275	87.3	0.0145

Cmpd 71, Dissect, 8.5 min

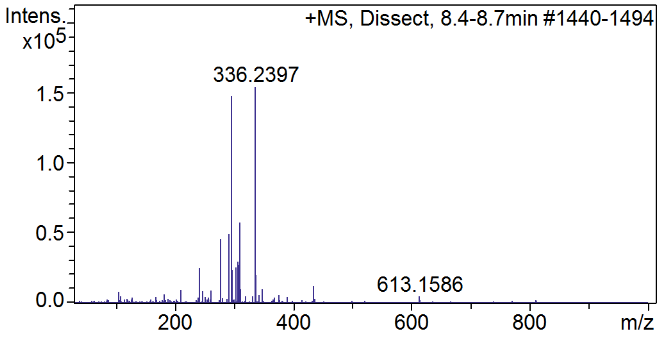

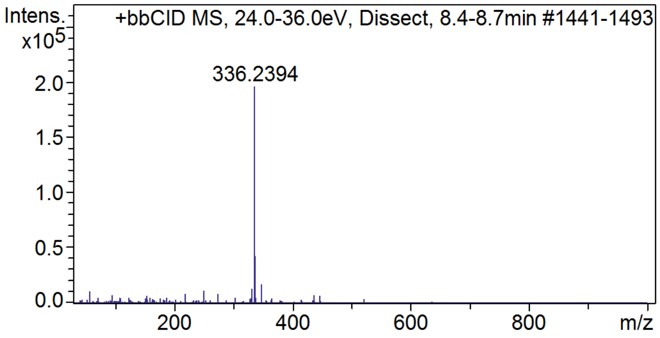

#	m/z	Res.	S/N	I	I %	FWHM
1	242.2761	28275	165.0	25449	16.5	0.0086
2	278.2386	31250	297.7	45910	29.8	0.0089
3	292.2175	31464	319.1	49218	31.9	0.0093
4	296.2486	33394	956.4	147491	95.7	0.0089
5	297.2519	31621	153.5	23673	15.4	0.0094
6	304.0360	31299	167.1	25763	16.7	0.0097
7	307.2166	31979	193.2	29790	19.3	0.0096
8	308.2120	27040	177.6	27382	17.8	0.0114
9	310.2271	31019	372.1	57391	37.2	0.0100
10	336.2397	33340	999.3	154102	100.0	0.0101

#	m/z	Res.	S/N	I	I %	FWHM
1	57.0677	17027	56.5	11066	5.7	0.0034
2	95.0824	19937	37.4	7311	3.7	0.0048
3	219.1670	28677	44.2	8648	4.4	0.0076
4	251.1559	28813	61.3	11990	6.1	0.0087
5	275.1913	30691	43.0	8422	4.3	0.0090
6	332.2084	31791	68.1	13334	6.8	0.0104
7	336.2394	32903	999.9	195703	100.0	0.0102
8	337.2423	30164	221.2	43294	22.1	0.0112
9	348.2029	30560	88.8	17378	8.9	0.0114
10	437.1788	28780	35.4	6930	3.5	0.0152

Compound Spectrum List Report

Cmpd 72, Dissect, 8.6 min

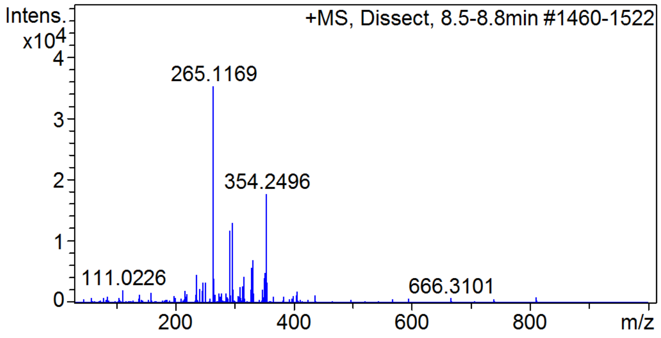

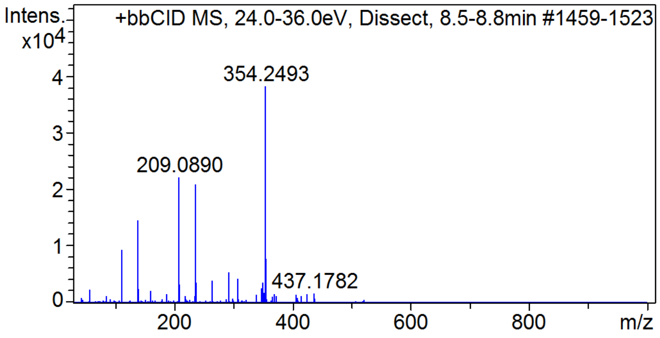

#	m/z	Res.	S/N	I	I %	FWHM
1	237.0832	28501	130.3	4593	13.0	0.0083
2	265.0770	31190	389.7	13739	39.0	0.0085
3	265.1169	30548	999.7	35244	100.0	0.0087
4	293.0710	31717	334.4	11791	33.5	0.0092
5	297.1023	31772	370.5	13063	37.1	0.0094
6	297.2517	30525	167.5	5905	16.8	0.0097
7	330.2527	32832	162.2	5720	16.2	0.0101
8	332.2685	30770	199.4	7029	19.9	0.0108
9	352.2341	32472	138.7	4890	13.9	0.0108
10	354.2496	31046	503.6	17755	50.4	0.0114

#	m/z	Res.	S/N	I	I %	FWHM
1	111.0226	22530	246.6	9439	24.7	0.0049
2	139.0166	24470	379.7	14536	38.0	0.0057
3	209.0890	28787	579.4	22181	58.0	0.0073
4	237.0831	30127	545.4	20879	54.6	0.0079
5	238.0866	28880	96.7	3703	9.7	0.0082
6	265.0771	29081	103.4	3956	10.3	0.0091
7	293.0710	31224	143.5	5494	14.4	0.0094
8	308.2103	28319	113.8	4356	11.4	0.0109
9	354.2493	32136	998.6	38227	100.0	0.0110
10	355.2526	31156	205.7	7874	20.6	0.0114

Cmpd 73, Dissect, 8.8 min

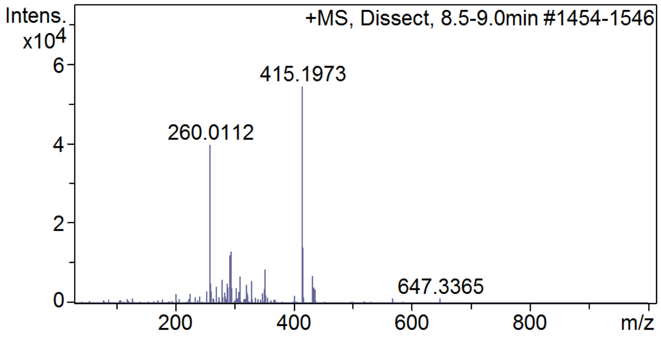

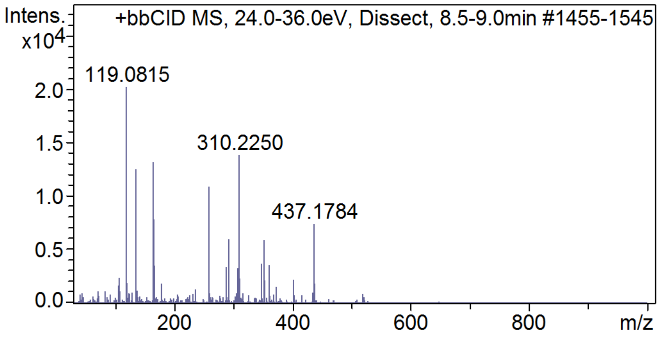

#	m/z	Res.	S/N	I	I %	FWHM
1	260.0112	30982	729.1	39682	73.0	0.0084
2	280.2179	29147	109.9	5981	11.0	0.0096
3	293.0710	31102	222.9	12131	22.3	0.0094
4	295.0836	30092	239.1	13014	23.9	0.0098
5	310.2261	31256	125.5	6829	12.6	0.0099
6	330.2527	31963	105.3	5732	10.5	0.0103
7	352.2341	32501	157.1	8550	15.7	0.0108
8	415.1973	33470	998.7	54352	100.0	0.0124
9	416.2006	31103	256.3	13951	25.7	0.0134
10	432.2231	30573	127.6	6944	12.8	0.0141

#	m/z	Res.	S/N	I	I %	FWHM
1	119.0565	22024	332.6	6722	33.3	0.0054
2	119.0815	23155	999.6	20204	100.0	0.0051
3	135.0759	24413	617.4	12480	61.8	0.0055
4	165.9723	26970	649.6	13130	65.0	0.0062
5	166.9798	24725	387.0	7821	38.7	0.0068
6	260.0112	30471	537.3	10859	53.7	0.0085
7	293.0710	31745	297.1	6004	29.7	0.0092
8	310.2250	30674	683.7	13818	68.4	0.0101
9	352.2338	33160	294.5	5953	29.5	0.0106
10	437.1784	30365	368.4	7446	36.9	0.0144

Compound Spectrum List Report

Cmpd 74, Dissect, 8.9 min

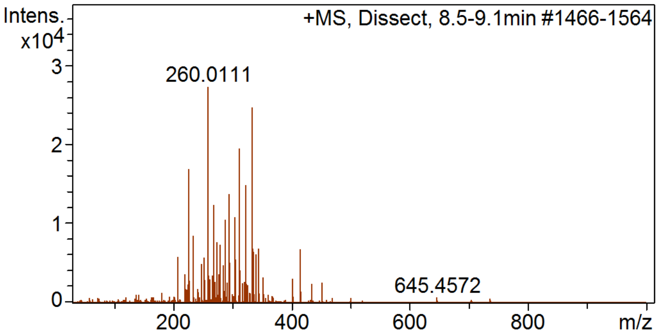

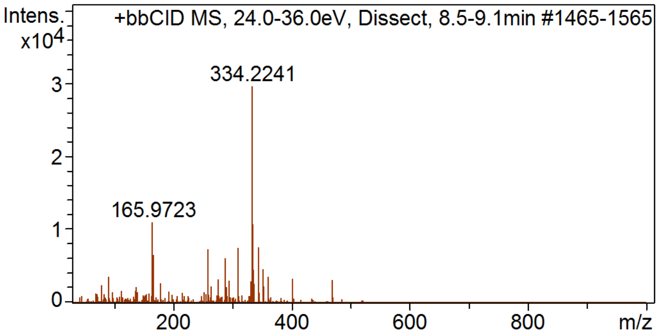

#	m/z	Res.	S/N	I	I %	FWHM
1	227.1563	28159	619.1	16926	61.9	0.0081
2	260.0111	30327	999.5	27329	100.0	0.0086
3	269.1083	30612	453.8	12408	45.4	0.0088
4	288.2800	30663	385.2	10531	38.5	0.0094
5	295.0836	30602	504.5	13793	50.5	0.0096
6	295.2360	30458	445.6	12184	44.6	0.0097
7	305.2007	30050	395.5	10812	39.6	0.0102
8	312.2430	31852	712.1	19469	71.2	0.0098
9	323.2110	31179	543.5	14861	54.4	0.0104
10	334.2241	32963	901.5	24650	90.2	0.0101

#	m/z	Res.	S/N	I	I %	FWHM
1	165.9723	26477	373.0	11065	37.3	0.0063
2	166.9798	24886	196.9	5841	19.7	0.0067
3	167.9879	26651	223.0	6616	22.3	0.0063
4	260.0112	29909	249.6	7406	25.0	0.0087
5	288.2801	29863	208.2	6176	20.8	0.0097
6	310.2251	29986	255.6	7584	25.6	0.0103
7	334.2241	34257	998.9	29636	100.0	0.0098
8	335.2272	31549	363.3	10778	36.4	0.0106
9	345.1921	31382	256.6	7613	25.7	0.0110
10	352.2339	32258	158.8	4712	15.9	0.0109

Cmpd 75, Dissect, 9.0 min

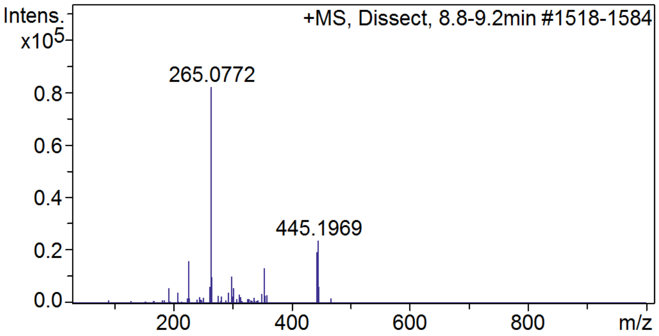

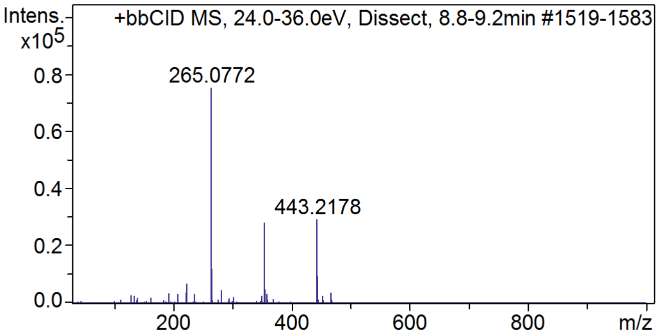

#	m/z	Res.	S/N	I	I %	FWHM
1	227.1563	28171	195.1	16018	19.5	0.0081
2	263.1918	30872	79.3	6507	7.9	0.0085
3	265.0772	32193	999.9	82073	100.0	0.0082
4	266.0804	30465	121.5	9976	12.2	0.0087
5	300.0048	31718	125.7	10319	12.6	0.0095
6	354.2505	29915	164.4	13494	16.4	0.0118
7	443.2177	31587	237.1	19461	23.7	0.0140
8	444.2206	30903	78.7	6463	7.9	0.0144
9	445.1969	32106	291.7	23944	29.2	0.0139
10	446.2000	29550	77.3	6347	7.7	0.0151

#	m/z	Res.	S/N	I	I %	FWHM
1	223.0679	28444	51.2	3868	5.1	0.0078
2	224.0996	28484	94.0	7097	9.4	0.0079
3	265.0772	32399	998.5	75369	100.0	0.0082
4	266.0807	30602	159.8	12064	16.0	0.0087
5	282.1944	30059	62.4	4711	6.3	0.0094
6	354.2494	31400	374.1	28234	37.5	0.0113
7	355.2526	30235	65.8	4965	6.6	0.0117
8	443.2178	32810	389.6	29411	39.0	0.0135
9	444.2209	30684	128.4	9695	12.9	0.0145
10	467.1783	30559	52.6	3974	5.3	0.0153

Compound Spectrum List Report

Cmpd 76, Dissect, 9.1 min

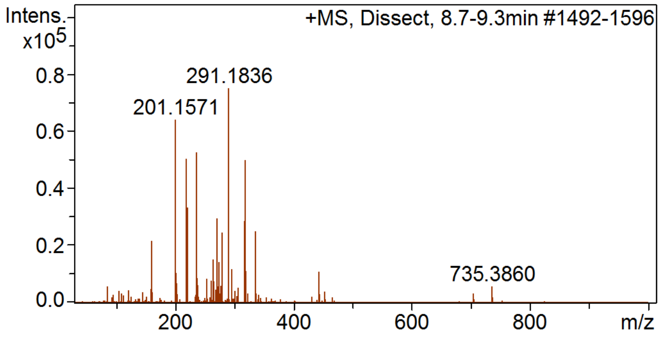

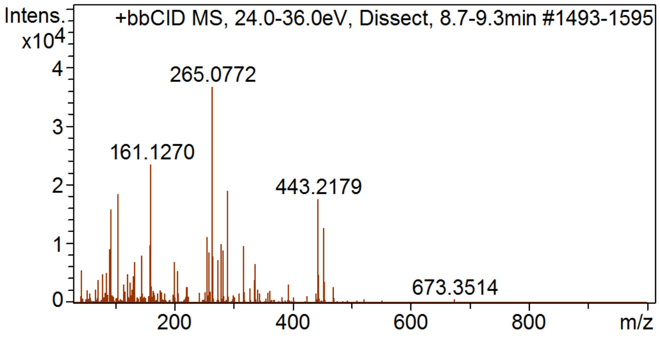

#	m/z	Res.	S/N	I	I %	FWHM
1	201.1571	29080	849.2	63853	85.0	0.0069
2	219.1671	29091	668.3	50250	66.9	0.0075
3	221.1826	28463	444.4	33414	44.5	0.0078
4	237.1771	30971	700.1	52642	70.1	0.0077
5	271.1240	29603	392.4	29503	39.3	0.0092
6	280.1604	29849	327.9	24659	32.8	0.0094
7	291.1836	31968	999.2	75133	100.0	0.0091
8	318.2898	32594	381.4	28678	38.2	0.0098
9	319.2162	32496	663.4	49884	66.4	0.0098
10	336.2402	31795	333.3	25062	33.4	0.0106

#	m/z	Res.	S/N	I	I %	FWHM
1	93.0668	19829	431.4	15847	43.1	0.0047
2	105.0664	21609	503.5	18496	50.4	0.0049
3	159.1114	26658	267.5	9827	26.8	0.0060
4	161.1270	26637	639.7	23497	64.0	0.0060
5	256.2550	30133	306.8	11270	30.7	0.0085
6	265.0772	32157	999.8	36727	100.0	0.0082
7	280.1603	30289	273.6	10050	27.4	0.0092
8	291.1837	30725	517.8	19022	51.8	0.0095
9	443.2179	33183	480.1	17635	48.0	0.0134
10	453.1729	31454	346.5	12727	34.7	0.0144

Cmpd 77, Dissect, 9.3 min

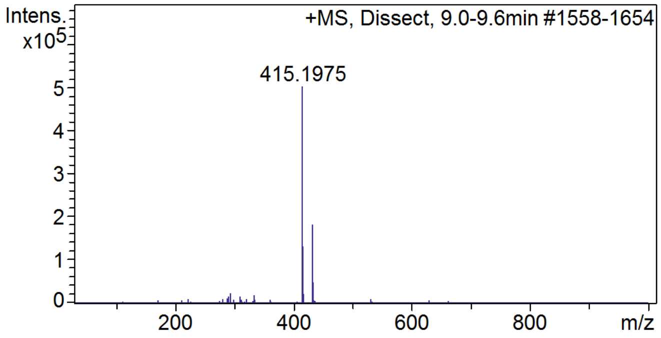

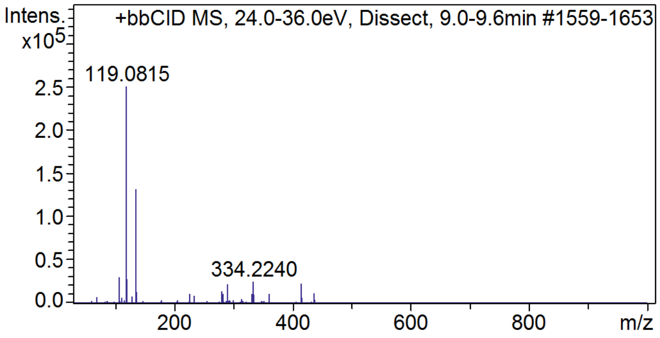

#	m/z	Res.	S/N	I	I %	FWHM
1	288.2802	30090	21.0	10530	2.1	0.0096
2	291.0557	31650	31.2	15671	3.1	0.0092
3	294.2331	32312	49.6	24924	5.0	0.0091
4	310.2275	31018	29.7	14909	3.0	0.0100
5	334.2242	32496	37.1	18652	3.7	0.0103
6	415.1975	38246	999.5	502132	100.0	0.0109
7	416.2010	33786	265.2	133238	26.5	0.0123
8	417.2036	29875	43.4	21788	4.3	0.0140
9	432.2236	34758	365.0	183371	36.5	0.0124
10	433.2267	31956	97.4	48919	9.7	0.0136

#	m/z	Res.	S/N	I	I %	FWHM
1	107.0820	22053	123.5	30955	12.4	0.0049
2	119.0565	22013	59.1	14828	5.9	0.0054
3	119.0815	23955	999.0	250454	100.0	0.0050
4	120.0849	23159	114.2	28639	11.4	0.0052
5	135.0759	25279	524.1	131408	52.5	0.0053
6	136.0793	24082	54.5	13672	5.5	0.0057
7	281.1290	31727	56.4	14142	5.6	0.0089
8	291.0555	31733	89.8	22523	9.0	0.0092
9	334.2240	33593	102.1	25595	10.2	0.0099
10	415.1973	32213	92.5	23185	9.3	0.0129

Compound Spectrum List Report

Cmpd 78, Dissect, 9.4 min

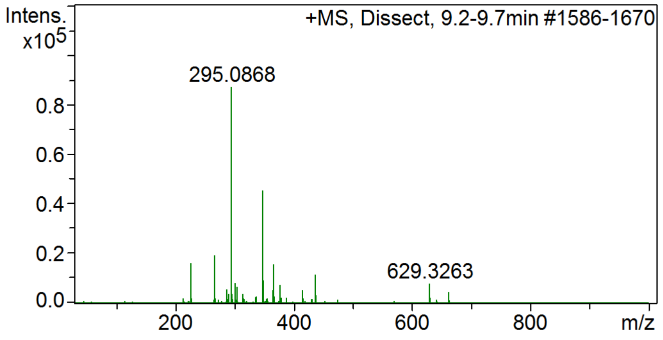

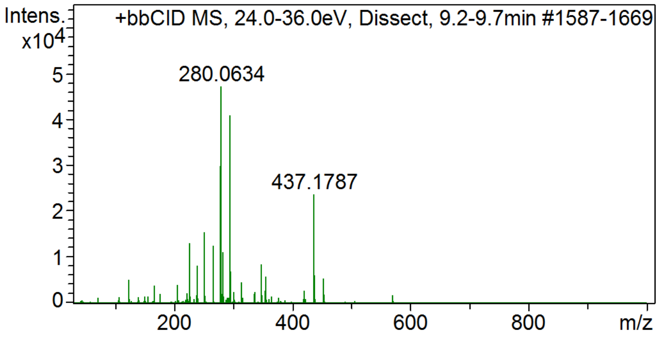

#	m/z	Res.	S/N	I	I %	FWHM
1	227.0562	28921	186.3	16258	18.6	0.0079
2	267.0928	31243	222.3	19395	22.2	0.0085
3	295.0868	32728	1000.0	87259	100.0	0.0090
4	302.0205	31266	94.7	8262	9.5	0.0097
5	348.0971	31927	520.0	45376	52.0	0.0109
6	349.1001	29943	106.9	9328	10.7	0.0117
7	366.1069	31375	181.4	15829	18.1	0.0117
8	377.0229	31637	86.3	7529	8.6	0.0119
9	437.1785	31875	133.4	11641	13.3	0.0137
10	629.3263	32705	93.2	8129	9.3	0.0192

#	m/z	Res.	S/N	I	I %	FWHM
1	227.0563	29571	278.1	13130	27.8	0.0077
2	240.2242	30502	175.3	8275	17.5	0.0079
3	252.0698	30155	327.0	15439	32.7	0.0084
4	267.0928	31183	266.3	12572	26.7	0.0086
5	279.0560	31523	630.5	29768	63.1	0.0089
6	280.0634	31228	999.0	47164	100.0	0.0090
7	283.1421	31493	237.5	11214	23.8	0.0090
8	295.0867	33021	863.5	40769	86.4	0.0089
9	348.0971	31672	181.1	8553	18.1	0.0110
10	437.1787	32952	503.6	23776	50.4	0.0133

Cmpd 79, Dissect, 9.5 min

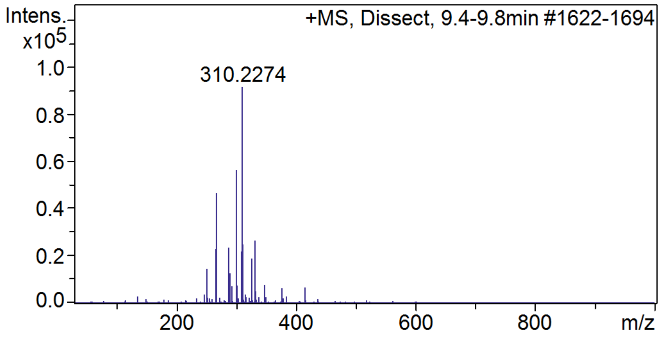

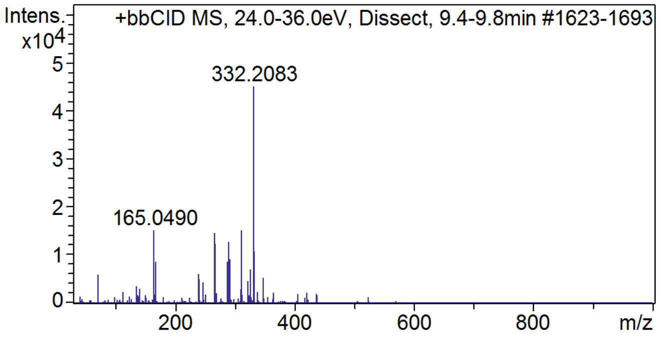

#	m/z	Res.	S/N	I	I %	FWHM
1	252.2239	30988	159.6	14661	16.0	0.0081
2	267.0929	31155	252.1	23158	25.3	0.0086
3	268.2183	32345	506.3	46508	50.8	0.0083
4	288.2802	31535	256.1	23524	25.7	0.0091
5	301.1335	33008	612.9	56303	61.4	0.0091
6	309.0988	32986	239.8	22033	24.0	0.0094
7	310.2274	31912	997.4	91626	100.0	0.0097
8	311.2308	31735	271.0	24900	27.2	0.0098
9	327.0754	30799	208.3	19140	20.9	0.0106
10	332.2086	32346	290.6	26700	29.1	0.0103

#	m/z	Res.	S/N	I	I %	FWHM
1	165.0490	26555	338.6	15253	33.9	0.0062
2	167.9879	27102	193.4	8712	19.3	0.0062
3	267.0927	31840	326.0	14684	32.6	0.0084
4	268.0636	28879	276.9	12476	27.7	0.0093
5	288.2801	31560	192.8	8684	19.3	0.0091
6	290.1994	31367	284.5	12816	28.5	0.0093
7	292.2152	32195	206.4	9297	20.6	0.0091
8	312.0523	31592	337.9	15224	33.8	0.0099
9	332.2083	33953	999.6	45030	100.0	0.0098
10	333.2115	31498	239.8	10804	24.0	0.0106

Compound Spectrum List Report

Cmpd 80, Dissect, 9.7 min

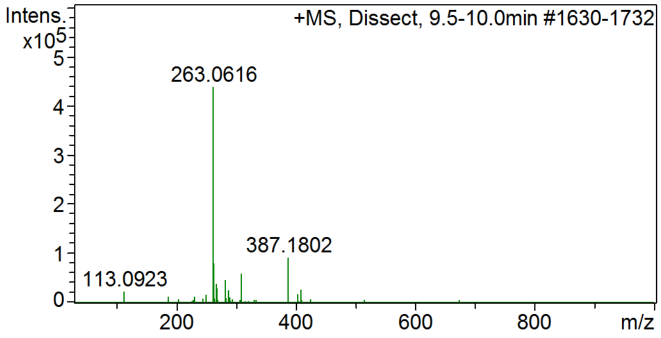

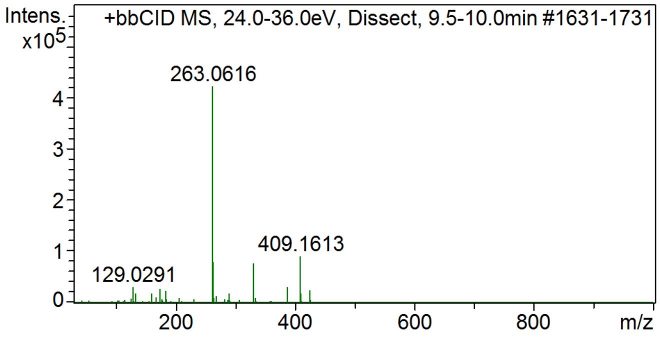

#	m/z	Res.	S/N	I	I %	FWHM
1	113.0923	22498	53.7	23660	5.4	0.0050
2	263.0616	34504	996.5	439220	100.0	0.0076
3	264.0650	31655	182.8	80584	18.3	0.0083
4	268.2184	32456	88.8	39139	8.9	0.0083
5	269.1923	31256	70.4	31045	7.1	0.0086
6	283.1236	31757	107.2	47232	10.8	0.0089
7	288.2801	31365	59.2	26074	5.9	0.0092
8	309.0988	32587	137.7	60680	13.8	0.0095
9	387.1802	33362	211.7	93306	21.2	0.0116
10	409.1612	31654	63.4	27929	6.4	0.0129

#	m/z	Res.	S/N	I	I %	FWHM
1	129.0291	24382	74.5	31500	7.5	0.0053
2	175.0096	28427	65.6	27708	6.6	0.0062
3	184.0933	27447	57.7	24397	5.8	0.0067
4	263.0616	34349	999.0	422223	100.0	0.0077
5	264.0650	31727	190.1	80331	19.0	0.0083
6	290.1995	31742	46.2	19506	4.6	0.0091
7	331.1197	34218	183.3	77493	18.4	0.0097
8	387.1802	32290	74.8	31602	7.5	0.0120
9	409.1613	35113	215.8	91211	21.6	0.0117
10	425.1346	32564	59.6	25190	6.0	0.0131

Cmpd 81, Dissect, 9.8 min

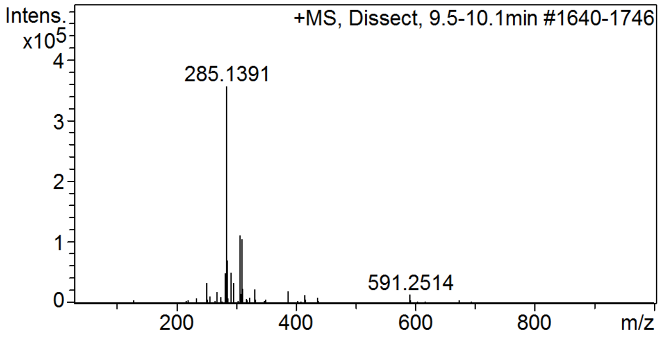

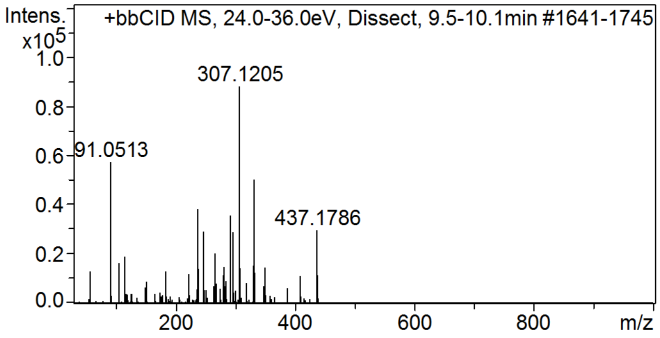

#	m/z	Res.	S/N	I	I %	FWHM
1	252.2239	31250	95.6	34064	9.6	0.0081
2	283.1236	31723	140.6	50090	14.1	0.0089
3	285.1391	34088	999.5	356149	100.0	0.0084
4	286.1425	31734	198.8	70847	19.9	0.0090
5	292.2162	30469	144.0	51324	14.4	0.0096
6	297.1022	31792	95.4	34000	9.5	0.0093
7	307.1204	33071	313.3	111636	31.3	0.0093
8	310.2274	31997	296.0	105473	29.6	0.0097
9	311.2307	31432	69.5	24763	7.0	0.0099
10	332.2085	32074	64.8	23096	6.5	0.0104

#	m/z	Res.	S/N	I	I %	FWHM
1	91.0513	20411	648.8	57206	64.9	0.0045
2	115.0503	22779	217.2	19152	21.7	0.0051
3	238.0909	29942	434.0	38266	43.4	0.0080
4	247.1975	31074	332.1	29279	33.2	0.0080
5	267.1292	30966	229.9	20273	23.0	0.0086
6	292.2153	32597	404.6	35679	40.5	0.0090
7	297.1024	31652	328.0	28920	32.8	0.0094
8	307.1205	34117	999.3	88109	100.0	0.0090
9	332.2085	33870	569.4	50206	57.0	0.0098
10	437.1786	33694	336.5	29673	33.7	0.0130

Compound Spectrum List Report

Cmpd 82, Dissect, 9.9 min

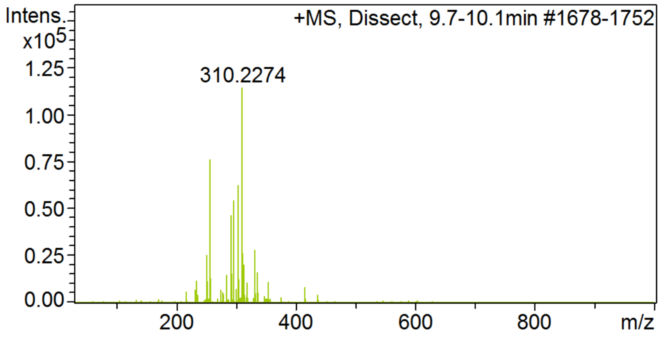

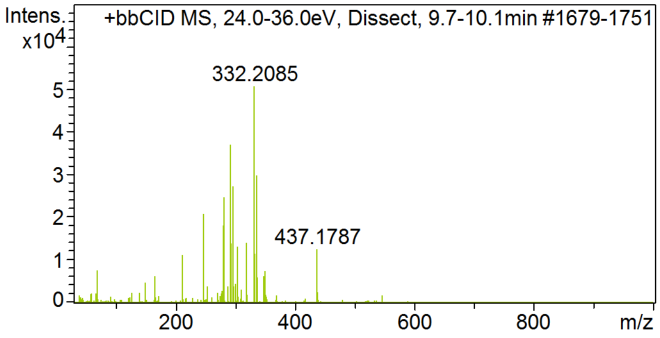

#	m/z	Res.	S/N	I	I %	FWHM
1	252.2239	31530	223.0	25514	22.3	0.0080
2	257.1087	31333	664.5	76015	66.5	0.0082
3	292.2162	30526	406.5	46496	40.7	0.0096
4	297.1024	32405	475.8	54425	47.6	0.0092
5	304.2899	31631	548.3	62718	54.8	0.0096
6	310.2274	31434	999.8	114375	100.0	0.0099
7	311.2306	30516	232.7	26623	23.3	0.0102
8	314.2586	32980	179.1	20485	17.9	0.0095
9	332.2085	32168	247.2	28279	24.7	0.0103
10	336.2397	33445	144.7	16556	14.5	0.0101

#	m/z	Res.	S/N	I	I %	FWHM
1	247.1975	30572	411.3	20845	41.1	0.0081
2	281.0717	30849	357.6	18125	35.8	0.0091
3	282.0788	29270	488.7	24769	48.9	0.0096
4	292.2152	33114	728.2	36909	72.8	0.0088
5	293.0713	33027	274.6	13916	27.5	0.0089
6	297.1023	31457	538.1	27273	53.8	0.0094
7	304.2897	31471	260.3	13193	26.0	0.0097
8	319.0835	30770	279.6	14172	28.0	0.0104
9	332.2085	33928	999.8	50674	100.0	0.0098
10	336.2396	34091	588.7	29839	58.9	0.0099

Cmpd 83, Dissect, 10.0 min

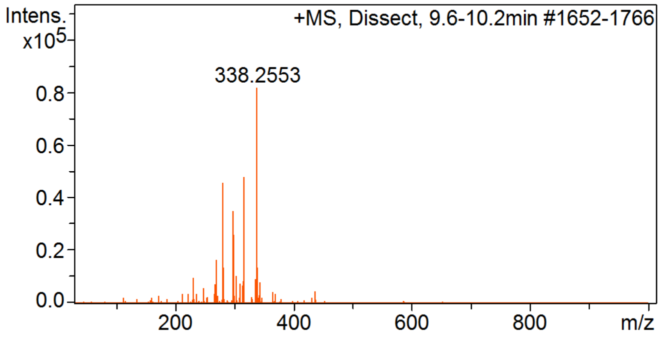

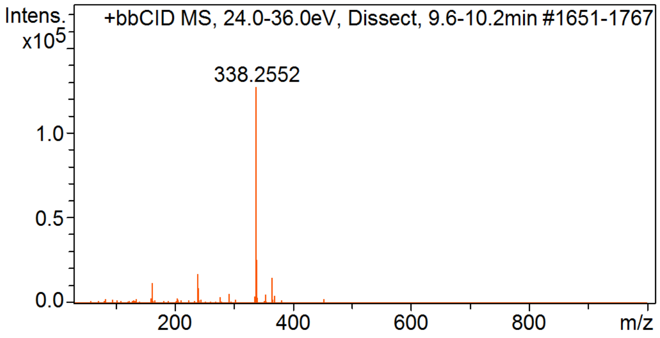

#	m/z	Res.	S/N	I	I %	FWHM
1	270.2338	30847	202.5	16568	20.3	0.0088
2	281.1060	29132	557.6	45607	55.8	0.0096
3	281.1289	27714	171.8	14049	17.2	0.0101
4	281.2381	28222	230.4	18849	23.1	0.0100
5	281.2576	29232	283.4	23179	28.4	0.0096
6	298.2642	31696	429.6	35138	43.0	0.0094
7	299.1180	31020	318.1	26020	31.8	0.0096
8	316.2742	32266	586.3	47955	58.7	0.0098
9	338.2553	33765	999.2	81733	100.0	0.0100
10	339.2585	29764	168.0	13745	16.8	0.0114

#	m/z	Res.	S/N	I	I %	FWHM
1	163.0334	26242	95.7	12167	9.6	0.0062
2	240.1021	25436	75.5	9600	7.6	0.0094
3	240.2241	29970	135.8	17276	13.6	0.0080
4	241.1154	23748	54.9	6985	5.5	0.0102
5	241.1516	25678	71.9	9150	7.2	0.0094
6	241.1869	24698	56.3	7167	5.6	0.0098
7	293.0713	33952	43.7	5556	4.4	0.0086
8	338.2552	34607	998.0	126947	100.0	0.0098
9	339.2586	30629	201.9	25680	20.2	0.0111
10	365.0935	30930	120.3	15307	12.1	0.0118

Compound Spectrum List Report

Cmpd 84, Dissect, 10.1 min

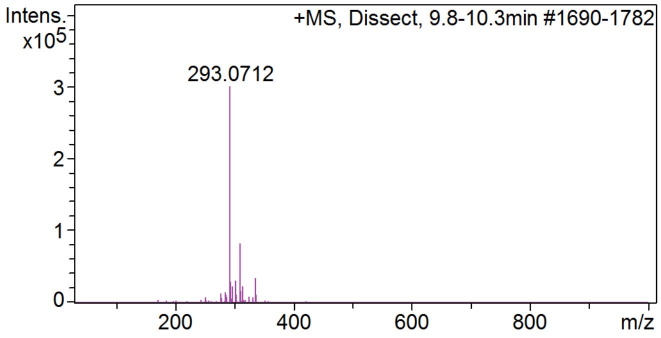

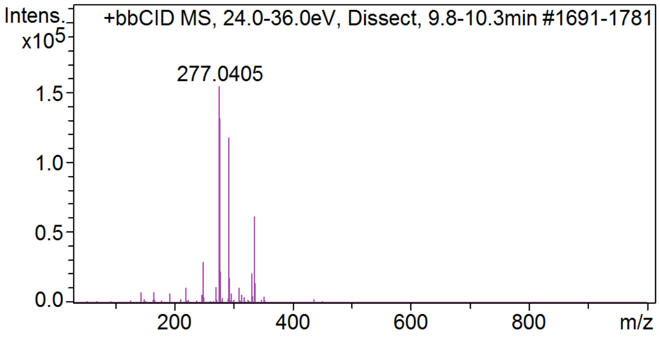

#	m/z	Res.	S/N	I	I %	FWHM
1	285.1389	29098	52.9	15926	5.3	0.0098
2	293.0712	34460	999.0	300678	100.0	0.0085
3	294.0746	32519	99.5	29938	10.0	0.0090
4	297.2520	31280	76.8	23108	7.7	0.0095
5	303.1280	32209	105.9	31875	10.6	0.0094
6	310.0253	31707	274.2	82533	27.4	0.0098
7	310.2275	30310	88.4	26600	8.8	0.0102
8	311.0283	29529	57.5	17293	5.8	0.0105
9	314.2586	33728	79.2	23837	7.9	0.0093
10	336.2397	33765	115.1	34637	11.5	0.0100

#	m/z	Res.	S/N	I	I %	FWHM
1	250.0543	30754	188.9	29153	18.9	0.0081
2	271.1239	30874	75.3	11620	7.5	0.0088
3	277.0405	33902	1000.0	154297	100.0	0.0082
4	278.0477	31278	850.1	131165	85.0	0.0089
5	279.0512	29528	146.6	22616	14.7	0.0095
6	293.0713	34057	761.3	117472	76.1	0.0086
7	294.0746	31794	117.2	18077	11.7	0.0092
8	332.2085	31735	138.0	21297	13.8	0.0105
9	336.2396	34094	400.9	61858	40.1	0.0099
10	337.2430	32035	94.1	14515	9.4	0.0105

Cmpd 85, Dissect, 10.2 min

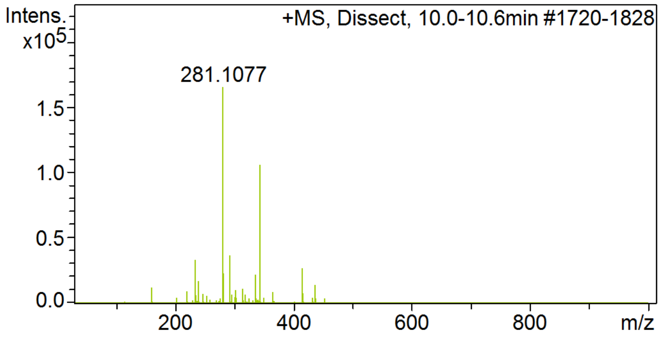

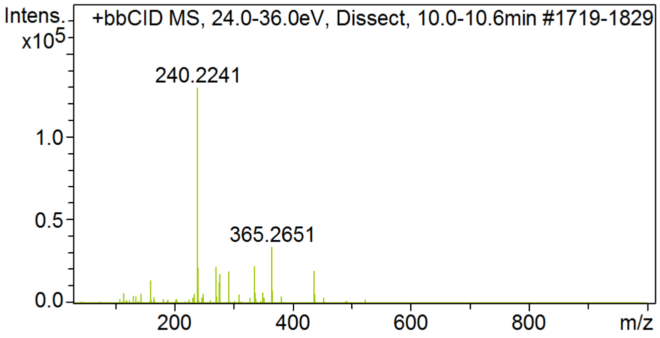

#	m/z	Res.	S/N	I	I %	FWHM
1	235.1614	29878	201.5	33429	20.2	0.0079
2	240.2239	30298	103.9	17226	10.4	0.0079
3	281.1077	35054	998.3	165573	100.0	0.0080
4	282.1111	32014	140.6	23326	14.1	0.0088
5	293.0712	33691	224.2	37183	22.5	0.0087
6	336.2397	33183	135.1	22409	13.5	0.0101
7	343.2839	34166	638.6	105919	64.0	0.0100
8	344.2872	31654	94.9	15738	9.5	0.0109
9	415.1973	32642	163.6	27141	16.4	0.0127
10	437.1785	32002	86.0	14265	8.6	0.0137

#	m/z	Res.	S/N	I	I %	FWHM
1	161.0542	26657	108.8	14135	10.9	0.0060
2	240.2241	32088	997.9	129670	100.0	0.0075
3	241.2275	30784	163.5	21251	16.4	0.0078
4	271.1239	30973	171.0	22222	17.1	0.0088
5	277.0403	33424	99.5	12928	10.0	0.0083
6	278.0476	31093	138.1	17951	13.8	0.0089
7	293.0711	33906	149.8	19465	15.0	0.0086
8	336.2395	33822	173.3	22523	17.4	0.0099
9	365.2651	33487	262.6	34120	26.3	0.0109
10	437.1785	33862	152.9	19863	15.3	0.0129

Compound Spectrum List Report

Cmpd 86, Dissect, 10.3 min

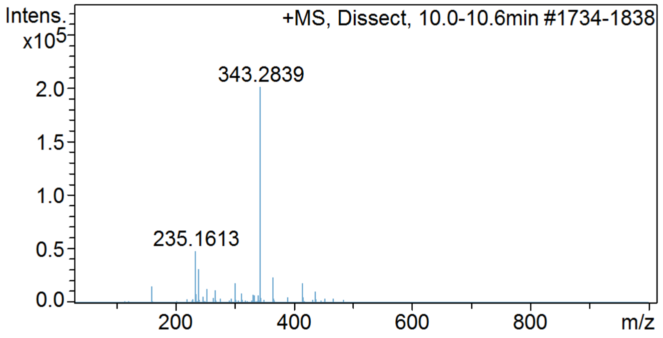

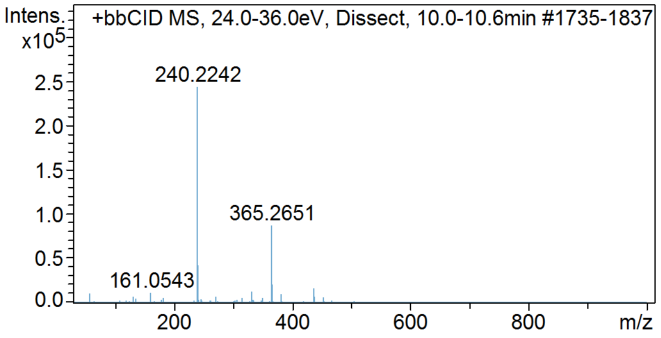

#	m/z	Res.	S/N	I	I %	FWHM
1	161.0540	25759	79.4	16028	8.0	0.0063
2	235.1613	29694	241.3	48692	24.2	0.0079
3	240.2241	30598	158.3	31943	15.9	0.0079
4	254.2393	30083	65.2	13164	6.5	0.0085
5	268.2182	30544	60.3	12170	6.0	0.0088
6	301.2750	30102	93.6	18891	9.4	0.0100
7	343.2839	34335	998.2	201469	100.0	0.0100
8	344.2872	31571	195.8	39507	19.6	0.0109
9	365.2650	30826	118.2	23854	11.8	0.0118
10	415.1972	32050	93.4	18850	9.4	0.0130

#	m/z	Res.	S/N	I	I %	FWHM
1	57.0676	17229	41.7	10229	4.2	0.0033
2	161.0543	26769	48.6	11928	4.9	0.0060
3	240.2242	32145	994.7	244256	100.0	0.0075
4	241.2275	30910	175.2	43011	17.6	0.0078
5	332.2083	31315	52.9	12984	5.3	0.0106
6	365.0930	32407	40.0	9833	4.0	0.0113
7	365.2651	33812	358.1	87931	36.0	0.0108
8	366.2683	30689	87.9	21591	8.8	0.0119
9	381.2382	28997	39.8	9783	4.0	0.0131
10	437.1786	33337	69.7	17106	7.0	0.0131

Cmpd 87, Dissect, 10.3 min

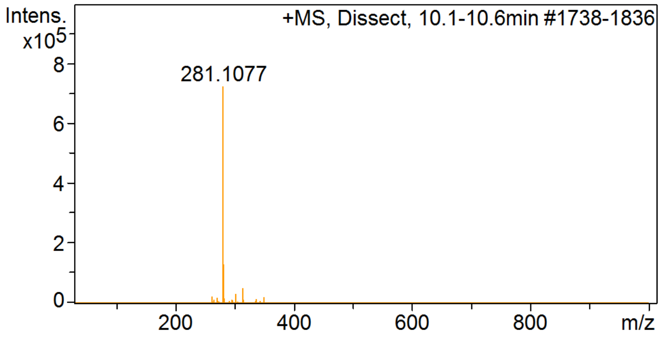

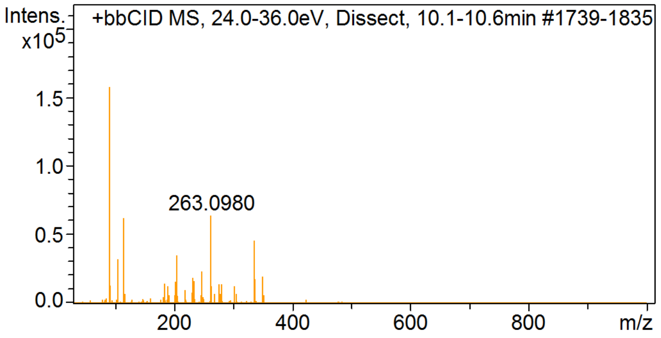

#	m/z	Res.	S/N	I	I %	FWHM
1	263.0979	29791	29.5	21360	3.0	0.0088
2	271.1239	29622	25.3	18347	2.5	0.0092
3	281.1077	36153	998.1	722559	100.0	0.0078
4	282.1112	32957	181.7	131535	18.2	0.0086
5	282.2334	30623	39.6	28683	4.0	0.0092
6	283.1142	27947	22.3	16155	2.2	0.0101
7	303.0891	30757	45.5	32922	4.6	0.0099
8	314.2585	33253	70.9	51310	7.1	0.0095
9	337.2430	31225	18.2	13196	1.8	0.0108
10	350.2184	31876	27.7	20020	2.8	0.0110

#	m/z	Res.	S/N	I	I %	FWHM
1	91.0512	20936	999.6	157732	100.0	0.0043
2	105.0664	21728	206.4	32576	20.7	0.0048
3	115.0504	23034	393.7	62120	39.4	0.0050
4	205.0941	27893	223.8	35308	22.4	0.0074
5	232.0804	28890	119.1	18787	11.9	0.0080
6	248.0748	28612	148.4	23419	14.8	0.0087
7	263.0980	32147	405.9	64042	40.6	0.0082
8	336.2396	33731	289.3	45657	28.9	0.0100
9	337.2428	30978	115.0	18151	11.5	0.0109
10	350.2185	32572	124.3	19607	12.4	0.0108

Compound Spectrum List Report

Cmpd 88, Dissect, 10.4 min

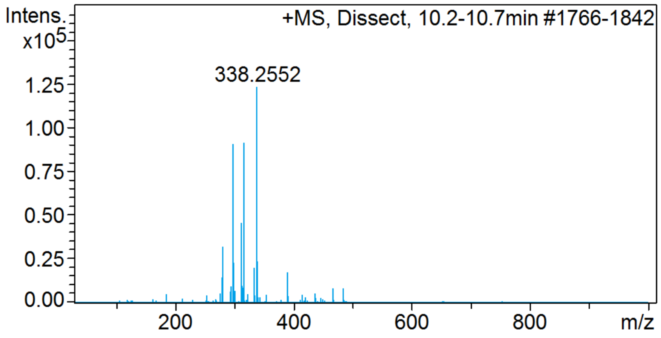

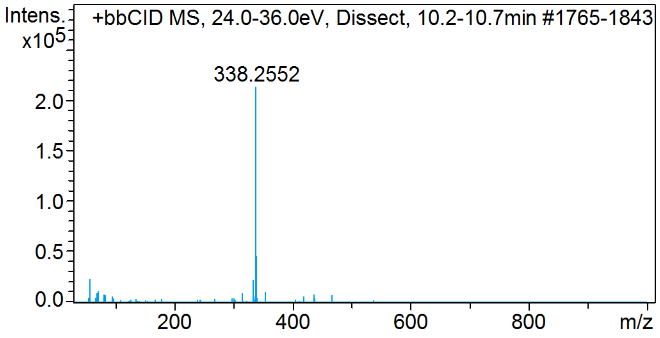

#	m/z	Res.	S/N	I	I %	FWHM
1	281.1078	35871	260.9	32268	26.1	0.0078
2	298.2641	32886	733.2	90677	73.4	0.0091
3	299.2673	30051	186.5	23062	18.7	0.0100
4	312.2429	32143	370.6	45835	37.1	0.0097
5	316.2742	33443	738.4	91313	73.9	0.0095
6	317.2774	30641	146.5	18120	14.7	0.0104
7	334.2242	31225	162.3	20067	16.2	0.0107
8	338.2552	34534	999.0	123545	100.0	0.0098
9	339.2585	30571	194.4	24042	19.5	0.0111
10	390.2871	30508	142.0	17560	14.2	0.0128

#	m/z	Res.	S/N	I	I %	FWHM
1	57.0676	17255	109.8	23554	11.0	0.0033
2	69.0673	18367	46.2	9912	4.6	0.0038
3	71.0829	18520	55.3	11853	5.5	0.0038
4	81.0670	19040	37.0	7929	3.7	0.0043
5	316.2141	31930	42.1	9032	4.2	0.0099
6	334.2241	32508	108.1	23189	10.9	0.0103
7	338.2552	35362	996.2	213649	100.0	0.0096
8	339.2585	32049	217.4	46615	21.8	0.0106
9	354.2284	30750	52.1	11181	5.2	0.0115
10	437.1786	33042	38.2	8191	3.8	0.0132

Cmpd 89, Dissect, 10.5 min

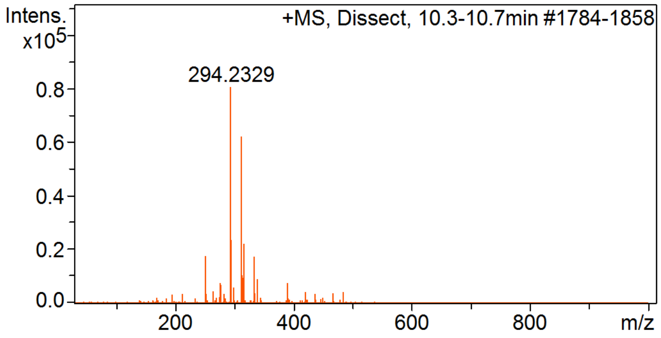

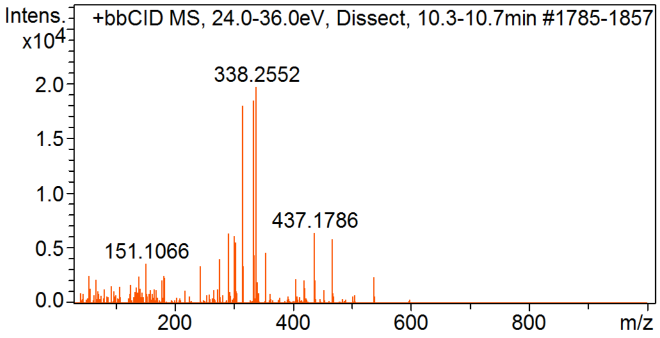

#	m/z	Res.	S/N	I	I %	FWHM
1	252.2237	31105	220.3	17777	22.0	0.0081
2	294.2329	32805	999.5	80657	100.0	0.0090
3	295.2363	31061	293.7	23699	29.4	0.0095
4	312.2429	32309	767.9	61968	76.8	0.0097
5	313.2461	30132	130.6	10541	13.1	0.0104
6	316.2142	30921	120.1	9694	12.0	0.0102
7	316.2741	33142	276.6	22319	27.7	0.0095
8	334.2242	31356	218.3	17620	21.8	0.0107
9	339.2586	30981	113.7	9173	11.4	0.0110
10	390.2870	29863	96.8	7814	9.7	0.0131

#	m/z	Res.	S/N	I	I %	FWHM
1	292.2154	31249	323.3	6370	32.4	0.0094
2	301.1335	30819	310.2	6111	31.0	0.0098
3	304.2147	31440	282.0	5557	28.2	0.0097
4	316.2140	32079	909.9	17928	91.1	0.0099
5	334.2242	32667	933.8	18399	93.5	0.0102
6	335.2272	30627	222.2	4379	22.2	0.0109
7	338.2552	34661	999.1	19685	100.0	0.0098
8	354.2286	29017	233.6	4602	23.4	0.0122
9	437.1786	32718	327.1	6445	32.7	0.0134
10	466.9810	31688	296.0	5832	29.6	0.0147

Compound Spectrum List Report

Cmpd 90, Dissect, 10.6 min

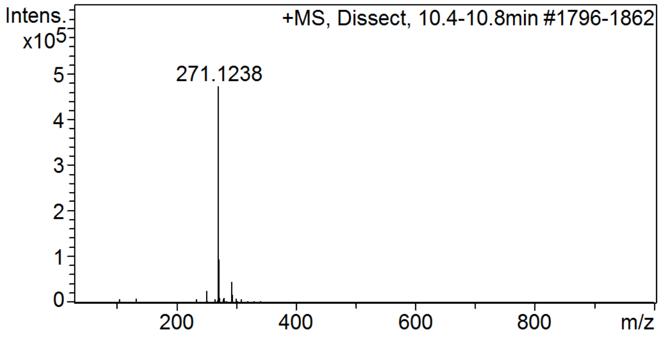

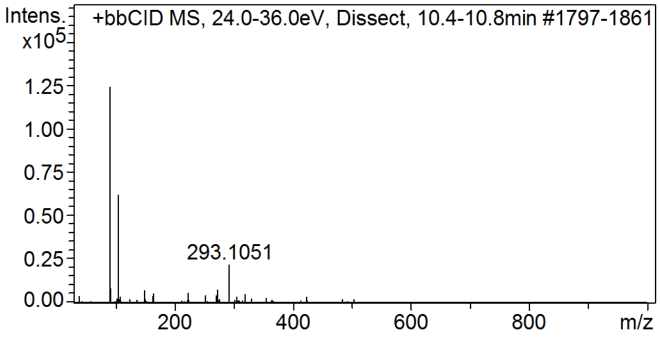

#	m/z	Res.	S/N	I	I %	FWHM
1	133.0603	23932	19.6	9295	2.0	0.0056
2	252.2237	30764	56.3	26683	5.6	0.0082
3	271.1238	34194	996.6	472648	100.0	0.0079
4	271.2372	28741	18.9	8950	1.9	0.0094
5	272.1273	32101	201.5	95573	20.2	0.0085
6	273.1318	15917	23.0	10890	2.3	0.0172
7	280.2535	26000	22.6	10722	2.3	0.0108
8	293.1051	31183	99.4	47131	10.0	0.0094
9	294.2329	32619	36.5	17322	3.7	0.0090
10	301.1334	30490	19.3	9137	1.9	0.0099

#	m/z	Res.	S/N	I	I %	FWHM
1	91.0512	20839	996.3	124308	100.0	0.0044
2	92.0545	19007	67.9	8470	6.8	0.0048
3	105.0664	21870	497.4	62059	49.9	0.0048
4	149.0182	23458	48.3	6027	4.8	0.0064
5	149.0911	25348	57.3	7152	5.8	0.0059
6	149.1274	25852	60.5	7550	6.1	0.0058
7	165.0490	25909	44.8	5594	4.5	0.0064
8	224.0756	26883	49.8	6213	5.0	0.0083
9	273.1394	30235	64.5	8050	6.5	0.0090
10	293.1051	32075	178.3	22250	17.9	0.0091

Cmpd 91, Dissect, 10.7 min

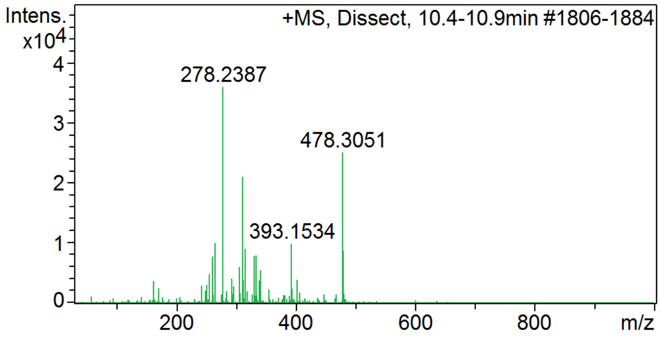

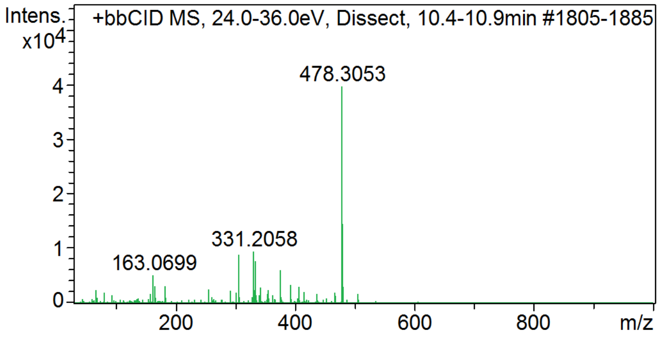

#	m/z	Res.	S/N	I	I %	FWHM
1	261.2127	28647	217.4	7825	21.7	0.0091
2	266.2391	30404	279.8	10070	28.0	0.0088
3	278.2387	31730	999.8	35983	100.0	0.0088
4	312.2431	31065	583.6	21003	58.4	0.0101
5	316.2142	30575	252.9	9101	25.3	0.0103
6	331.2060	30140	220.4	7934	22.0	0.0110
7	334.2241	30094	221.4	7968	22.1	0.0111
8	393.1534	30732	277.4	9983	27.7	0.0128
9	478.3051	31687	695.6	25034	69.6	0.0151
10	479.3088	29148	242.7	8734	24.3	0.0164

#	m/z	Res.	S/N	I	I %	FWHM
1	163.0699	26604	132.0	5237	13.2	0.0061
2	166.0595	27246	79.7	3162	8.0	0.0061
3	183.0853	28521	80.5	3193	8.1	0.0064
4	306.2301	31532	224.2	8898	22.4	0.0097
5	331.2058	31703	241.1	9569	24.1	0.0104
6	334.2241	31620	195.2	7747	19.5	0.0106
7	376.1273	30925	155.9	6188	15.6	0.0122
8	393.1534	26350	86.7	3440	8.7	0.0149
9	478.3053	32545	999.1	39650	100.0	0.0147
10	479.3083	30379	366.1	14528	36.6	0.0158

Compound Spectrum List Report

Cmpd 92, Dissect, 10.7 min

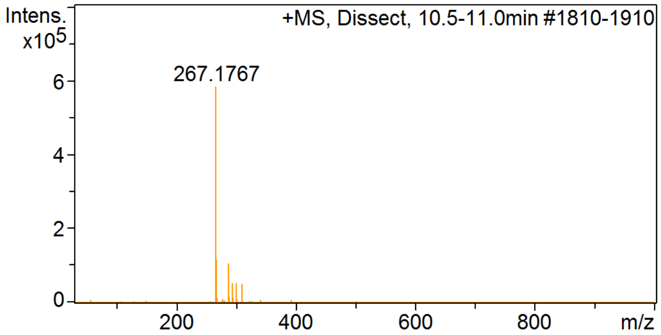

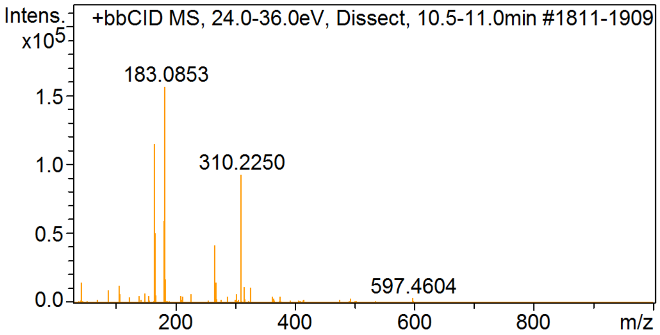

#	m/z	Res.	S/N	I	I %	FWHM
1	267.1767	34363	990.7	584739	100.0	0.0078
2	268.1801	30862	200.2	118194	20.2	0.0087
3	269.1084	29926	18.9	11162	1.9	0.0090
4	269.1843	25427	21.3	12559	2.1	0.0106
5	288.2439	32839	183.6	108361	18.5	0.0088
6	289.2473	29799	28.2	16670	2.9	0.0097
7	294.2330	32892	91.1	53780	9.2	0.0089
8	295.2364	31515	21.3	12589	2.2	0.0094
9	301.1334	31610	93.3	55089	9.4	0.0095
10	310.2250	31669	89.2	52642	9.0	0.0098

#	m/z	Res.	S/N	I	I %	FWHM
1	43.0524	15330	95.7	14949	9.6	0.0028
2	106.0828	21021	81.8	12777	8.2	0.0050
3	166.0595	27587	733.2	114506	73.4	0.0060
4	167.0666	23205	322.7	50398	32.3	0.0072
5	182.0777	28375	378.8	59166	37.9	0.0064
6	183.0853	28876	998.7	155974	100.0	0.0063
7	184.0892	23342	110.0	17182	11.0	0.0079
8	267.1766	31473	268.3	41901	26.9	0.0085
9	269.1082	30380	95.7	14945	9.6	0.0089
10	310.2250	32870	593.6	92708	59.4	0.0094

Cmpd 93, Dissect, 10.9 min

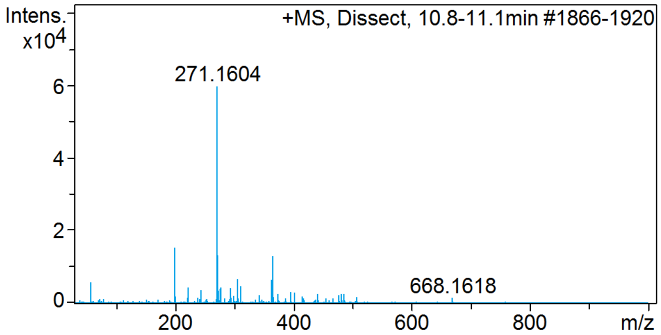

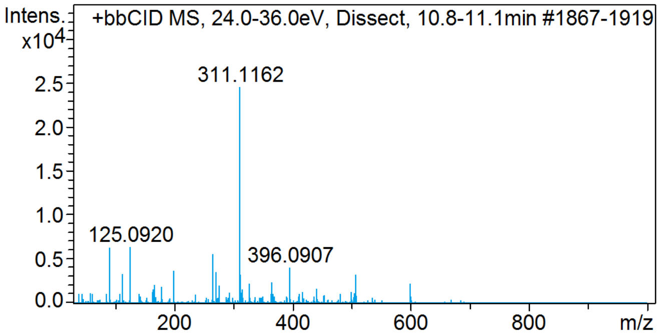

#	m/z	Res.	S/N	I	I %	FWHM
1	57.0676	17166	98.3	5877	9.8	0.0033
2	200.1942	29845	257.7	15404	25.8	0.0067
3	223.0891	29688	76.2	4554	7.6	0.0075
4	271.1604	30593	998.5	59691	100.0	0.0089
5	272.1637	29892	220.8	13198	22.1	0.0091
6	278.2386	30177	75.1	4489	7.5	0.0092
7	306.0879	30311	113.4	6780	11.4	0.0101
8	311.1160	30184	81.2	4855	8.1	0.0103
9	363.0471	32465	110.7	6620	11.1	0.0112
10	365.0449	31361	220.1	13157	22.0	0.0116

#	m/z	Res.	S/N	I	I %	FWHM
1	91.0513	19970	257.5	6333	25.8	0.0046
2	113.0923	22093	137.3	3376	13.7	0.0051
3	125.0920	23625	259.8	6391	26.0	0.0053
4	200.1941	28954	152.6	3754	15.3	0.0069
5	266.1214	28722	228.9	5630	22.9	0.0093
6	271.1604	28762	144.6	3556	14.5	0.0094
7	311.1162	33089	999.3	24580	100.0	0.0094
8	312.1195	30257	133.9	3292	13.4	0.0103
9	396.0907	31669	166.6	4098	16.7	0.0125
10	507.3595	30911	132.9	3269	13.3	0.0164

Compound Spectrum List Report

Cmpd 94, Dissect, 11.0 min

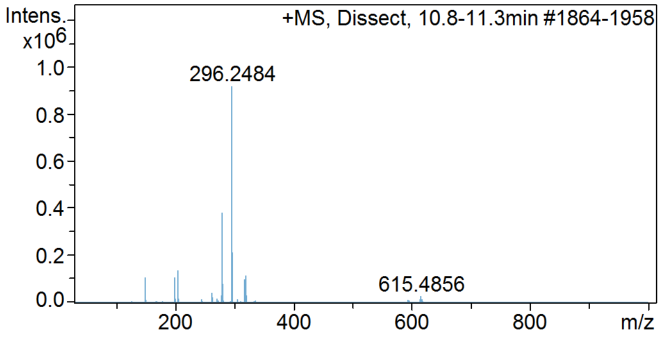

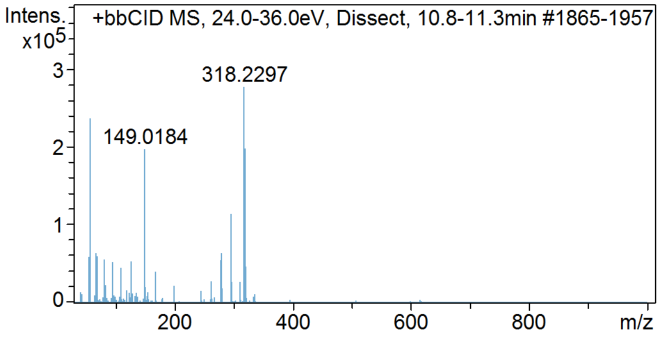

#	m/z	Res.	S/N	I	I %	FWHM
1	149.0184	26253	119.7	109994	12.0	0.0057
2	200.1941	29865	118.8	109171	11.9	0.0067
3	205.0791	30373	152.4	140078	15.3	0.0068
4	263.2282	31786	48.4	44520	4.9	0.0083
5	280.2542	36421	416.2	382525	41.7	0.0077
6	281.2576	32018	88.7	81553	8.9	0.0088
7	296.2484	38077	997.6	916947	100.0	0.0078
8	297.2519	35136	232.8	214016	23.3	0.0085
9	318.2297	34703	110.3	101380	11.1	0.0092
10	320.2453	33384	127.6	117330	12.8	0.0096

#	m/z	Res.	S/N	I	I %	FWHM
1	55.0520	17397	214.3	59649	21.5	0.0032
2	57.0676	17758	849.6	236425	85.2	0.0032
3	67.0518	18267	230.8	64233	23.1	0.0037
4	69.0673	18545	215.3	59906	21.6	0.0037
5	149.0184	27241	708.1	197049	71.0	0.0055
6	280.2544	32966	231.8	64495	23.2	0.0085
7	296.2487	33916	409.8	114051	41.1	0.0087
8	318.2297	36370	997.3	277527	100.0	0.0087
9	319.2331	33717	250.7	69777	25.1	0.0095
10	320.2453	34647	709.9	197563	71.2	0.0092

Cmpd 95, Dissect, 11.1 min

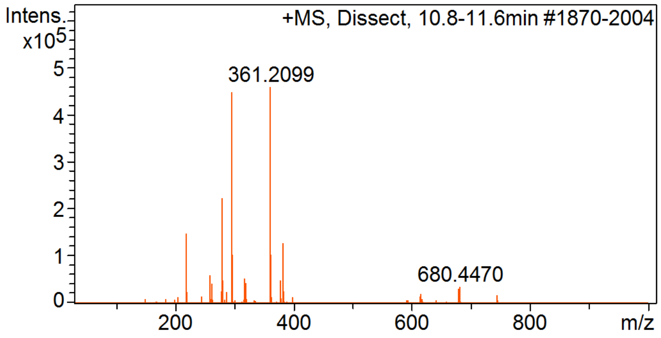

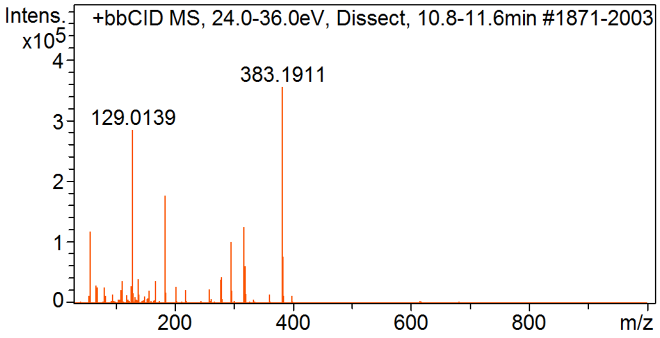

#	m/z	Res.	S/N	I	I %	FWHM
1	219.1670	30074	318.0	148674	32.3	0.0073
2	259.1454	30712	129.2	60405	13.1	0.0084
3	280.2541	36609	477.9	223424	48.6	0.0077
4	281.2575	31788	105.8	49438	10.8	0.0088
5	296.2484	37964	957.9	447810	97.4	0.0078
6	297.2519	35818	220.5	103091	22.4	0.0083
7	318.2296	34768	115.7	54063	11.8	0.0092
8	361.2099	37836	983.5	459739	100.0	0.0095
9	362.2133	33080	221.1	103345	22.5	0.0109
10	383.1911	34776	275.2	128634	28.0	0.0110

#	m/z	Res.	S/N	I	I %	FWHM
1	57.0676	17746	331.4	117985	33.2	0.0032
2	129.0139	25116	796.7	283622	79.8	0.0051
3	138.9979	24847	113.3	40324	11.3	0.0056
4	185.0746	29975	497.9	177257	49.9	0.0062
5	280.2543	32634	122.0	43438	12.2	0.0086
6	296.2486	34217	284.3	101214	28.5	0.0087
7	318.2296	36821	353.3	125782	35.4	0.0086
8	320.2452	34823	171.8	61168	17.2	0.0092
9	383.1911	37613	998.6	355516	100.0	0.0102
10	384.1946	32859	216.3	77002	21.7	0.0117

Compound Spectrum List Report

Cmpd 96, Dissect, 11.2 min

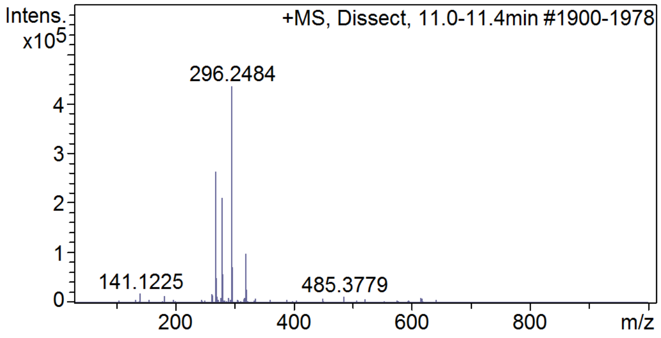

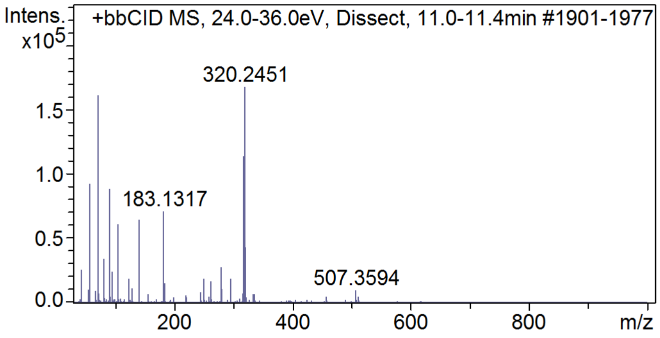

#	m/z	Res.	S/N	I	I %	FWHM
1	141.1225	24940	41.5	18057	4.2	0.0057
2	263.2282	31557	40.1	17478	4.0	0.0083
3	269.1083	32877	606.4	264083	60.7	0.0082
4	270.1117	29278	115.3	50221	11.5	0.0092
5	280.2541	36566	485.2	211313	48.6	0.0077
6	281.2574	31772	132.6	57764	13.3	0.0089
7	296.2484	37545	999.0	435041	100.0	0.0079
8	297.2518	36328	167.3	72862	16.7	0.0082
9	320.2452	33470	227.6	99109	22.8	0.0096
10	321.2486	30361	62.1	27050	6.2	0.0106

#	m/z	Res.	S/N	I	I %	FWHM
1	57.0676	17651	550.4	92682	55.2	0.0032
2	71.0828	19097	958.4	161387	96.2	0.0037
3	81.0670	20002	203.7	34293	20.4	0.0041
4	91.0511	20499	525.3	88445	52.7	0.0044
5	105.0663	21705	363.2	61152	36.4	0.0048
6	141.1227	25115	383.5	64575	38.5	0.0056
7	183.1317	28154	423.0	71230	42.4	0.0065
8	318.2295	37055	676.1	113850	67.8	0.0086
9	320.2451	34735	996.6	167805	100.0	0.0092
10	321.2486	31570	257.6	43369	25.8	0.0102

Cmpd 97, Dissect, 11.2 min

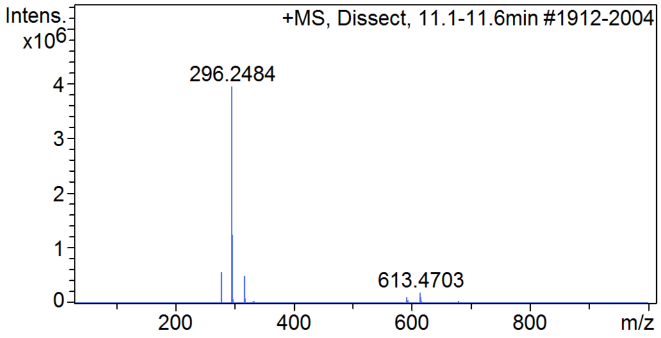

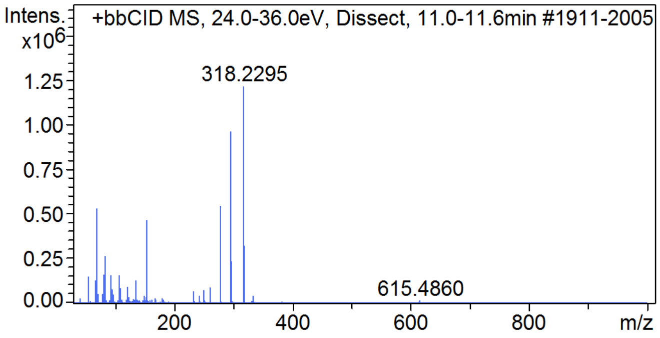

#	m/z	Res.	S/N	I	I %	FWHM
1	279.2226	33820	143.6	571449	14.5	0.0083
2	296.2484	37450	991.7	3947587	100.0	0.0079
3	297.2518	36587	313.4	1247420	31.6	0.0081
4	298.2560	24554	17.7	70653	1.8	0.0121
5	318.2296	35238	126.5	503733	12.8	0.0090
6	319.2328	30812	22.7	90540	2.3	0.0104
7	591.4891	31345	27.5	109614	2.8	0.0189
8	613.4703	32720	52.1	207516	5.3	0.0187
9	614.4735	30870	24.4	97166	2.5	0.0199
10	615.4855	31447	27.6	109835	2.8	0.0196

#	m/z	Res.	S/N	I	I %	FWHM
1	69.0672	19017	435.6	530925	43.7	0.0036
2	81.0670	20081	133.9	163240	13.4	0.0040
3	83.0825	20072	218.3	266021	21.9	0.0041
4	107.0820	21797	130.6	159140	13.1	0.0049
5	153.1222	27145	384.0	467966	38.5	0.0056
6	279.2227	32777	448.4	546464	45.0	0.0085
7	296.2485	35145	788.7	961200	79.1	0.0084
8	297.2519	32617	195.0	237697	19.6	0.0091
9	318.2295	37514	997.3	1215464	100.0	0.0085
10	319.2330	33714	266.6	324961	26.7	0.0095

Compound Spectrum List Report

Cmpd 98, Dissect, 11.3 min

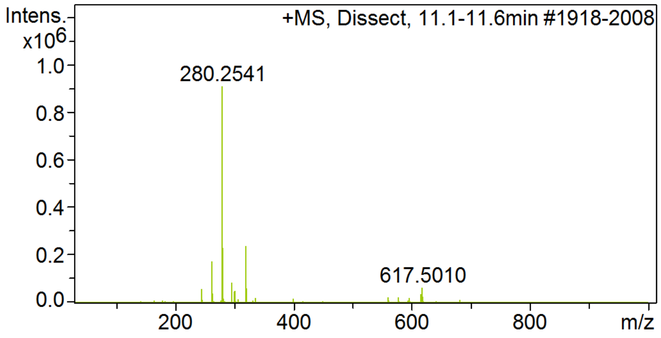

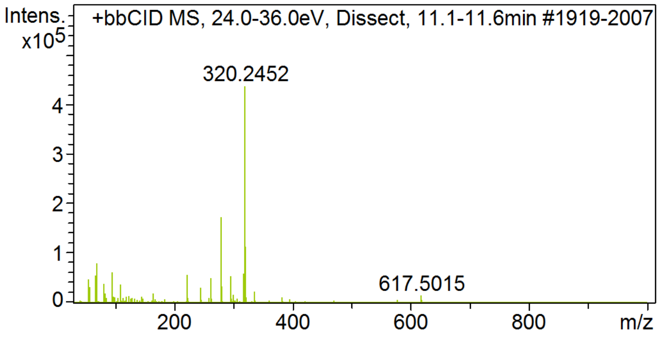

#	m/z	Res.	S/N	I	I %	FWHM
1	245.2181	30205	65.6	59933	6.6	0.0081
2	263.2282	32441	191.5	175077	19.2	0.0081
3	280.2541	37168	995.3	909947	100.0	0.0075
4	281.2574	32793	252.6	230963	25.4	0.0086
5	296.2484	37859	94.3	86195	9.5	0.0078
6	300.9823	31546	55.5	50766	5.6	0.0095
7	301.1487	31653	58.4	53416	5.9	0.0095
8	320.2452	34410	261.8	239326	26.3	0.0093
9	321.2486	31068	68.6	62736	6.9	0.0103
10	617.5010	30042	71.0	64872	7.1	0.0206

#	m/z	Res.	S/N	I	I %	FWHM
1	67.0518	18369	127.4	55682	12.8	0.0037
2	69.0673	19003	183.6	80196	18.4	0.0036
3	95.0823	21123	144.3	63057	14.4	0.0045
4	223.1043	29606	130.4	56966	13.1	0.0075
5	263.2282	30150	116.7	51001	11.7	0.0087
6	280.2542	33160	396.7	173318	39.7	0.0085
7	296.2486	34874	124.5	54405	12.5	0.0085
8	318.2295	37147	137.9	60260	13.8	0.0086
9	320.2452	36357	999.0	436495	100.0	0.0088
10	321.2487	32210	259.8	113496	26.0	0.0100

Cmpd 99, Dissect, 11.4 min

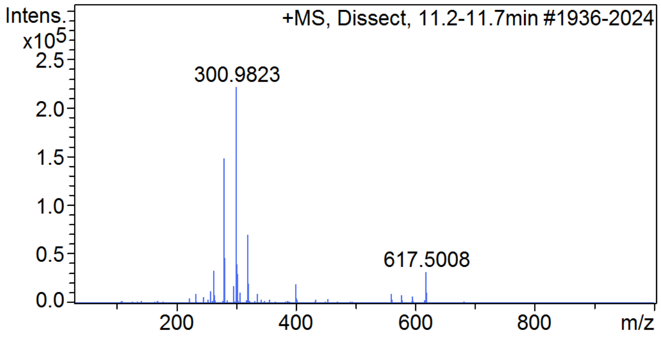

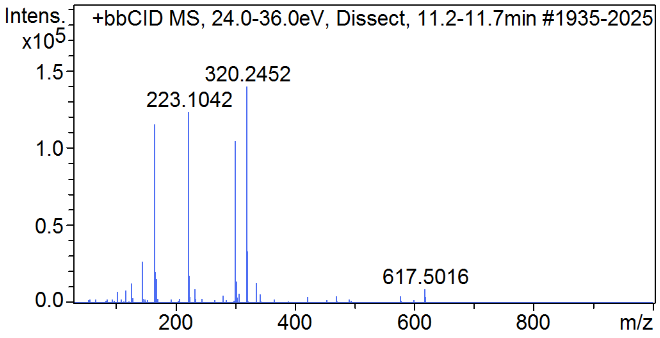

#	m/z	Res.	S/N	I	I %	FWHM
1	263.2282	31325	152.0	33778	15.2	0.0084
2	280.2541	36089	667.6	148334	66.9	0.0078
3	281.2575	31721	210.7	46816	21.1	0.0089
4	300.9823	32215	997.4	221619	100.0	0.0093
5	301.1487	31630	683.7	151920	68.6	0.0095
6	301.9851	28312	182.4	40518	18.3	0.0107
7	302.1521	30274	173.9	38644	17.4	0.0100
8	302.9783	29030	137.5	30562	13.8	0.0104
9	320.2453	33426	316.4	70315	31.7	0.0096
10	617.5008	29690	144.5	32097	14.5	0.0208

#	m/z	Res.	S/N	I	I %	FWHM
1	145.0599	25520	193.5	27057	19.4	0.0057
2	165.9723	27189	822.8	115060	82.3	0.0061
3	166.9790	20220	120.7	16883	12.1	0.0083
4	167.1374	26721	146.6	20498	14.7	0.0063
5	169.1166	27107	113.9	15933	11.4	0.0062
6	223.1042	29983	880.1	123074	88.1	0.0074
7	224.1077	29077	128.0	17906	12.8	0.0077
8	300.9823	31999	745.6	104266	74.6	0.0094
9	320.2452	34937	999.4	139757	100.0	0.0092
10	321.2487	31598	240.4	33614	24.1	0.0102

Compound Spectrum List Report

Cmpd 100, Dissect, 11.5 min

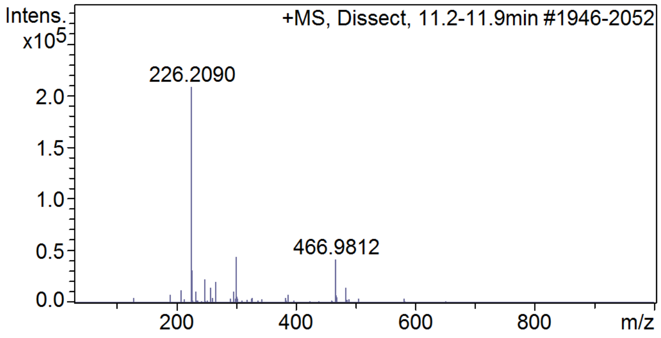

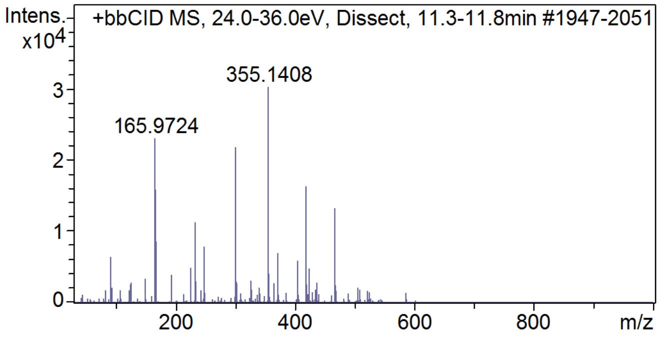

#	m/z	Res.	S/N	I	I %	FWHM
1	226.2090	31551	999.4	208773	100.0	0.0072
2	227.2124	29165	153.0	31964	15.3	0.0078
3	248.1904	29586	110.1	22995	11.0	0.0084
4	248.2501	29848	62.5	13061	6.3	0.0083
5	258.0320	29240	72.4	15127	7.2	0.0088
6	267.0928	30861	97.9	20459	9.8	0.0087
7	300.9823	32141	214.2	44741	21.4	0.0094
8	301.1488	30771	102.4	21391	10.2	0.0098
9	466.9812	34302	201.3	42049	20.1	0.0136
10	484.0070	31449	72.6	15171	7.3	0.0154

#	m/z	Res.	S/N	I	I %	FWHM
1	165.9724	27212	758.8	23008	75.9	0.0061
2	166.9791	20336	246.9	7486	24.7	0.0082
3	167.1374	27074	522.4	15841	52.3	0.0062
4	167.9880	27560	286.0	8673	28.6	0.0061
5	233.2187	30205	372.6	11299	37.3	0.0077
6	248.9723	30173	260.5	7899	26.1	0.0083
7	300.9824	31979	718.7	21793	71.9	0.0094
8	355.1408	32527	999.7	30312	100.0	0.0109
9	418.9794	31900	538.5	16328	53.9	0.0131
10	466.9813	33159	439.5	13327	44.0	0.0141

Cmpd 101, Dissect, 11.6 min

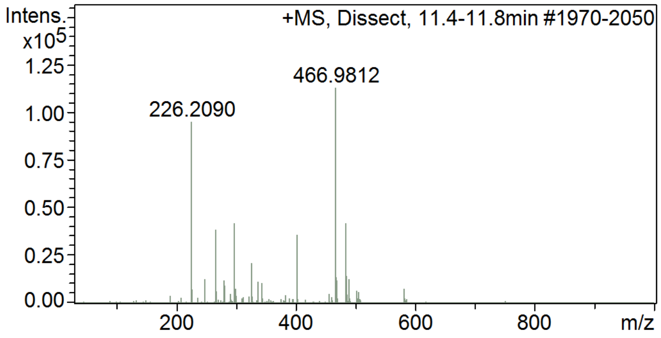

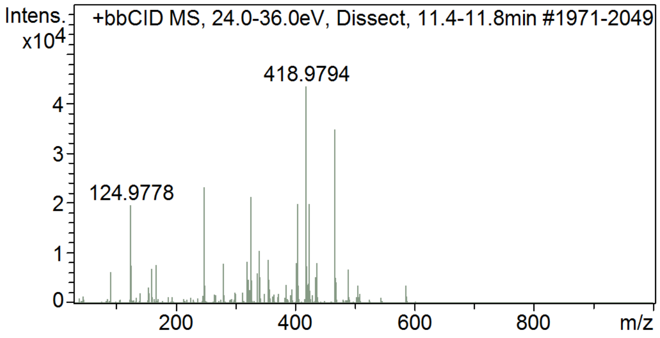

#	m/z	Res.	S/N	I	I %	FWHM
1	226.2090	30877	838.0	94826	83.8	0.0073
2	248.1904	30532	115.2	13030	11.5	0.0081
3	267.0929	30981	340.6	38537	34.1	0.0086
4	298.2641	32704	372.4	42142	37.3	0.0091
5	326.3673	31704	188.4	21319	18.8	0.0103
6	402.1420	38296	317.8	35961	31.8	0.0105
7	466.9812	34605	999.5	113100	100.0	0.0135
8	467.9838	30642	122.7	13887	12.3	0.0153
9	484.0070	31935	372.4	42139	37.3	0.0152
10	485.2731	32698	127.2	14396	12.7	0.0148

#	m/z	Res.	S/N	I	I %	FWHM
1	124.9778	24201	453.0	19696	45.3	0.0052
2	248.9722	30523	535.3	23279	53.6	0.0082
3	320.2453	32973	193.2	8403	19.3	0.0097
4	326.3672	32661	490.2	21318	49.1	0.0100
5	340.9951	31484	241.8	10516	24.2	0.0108
6	355.1409	32620	203.2	8838	20.3	0.0109
7	404.9644	32619	457.4	19891	45.8	0.0124
8	418.9794	32111	999.1	43444	100.0	0.0130
9	424.1231	34032	459.0	19957	45.9	0.0125
10	466.9813	33361	801.0	34829	80.2	0.0140

Compound Spectrum List Report

Cmpd 102, Dissect, 11.7 min

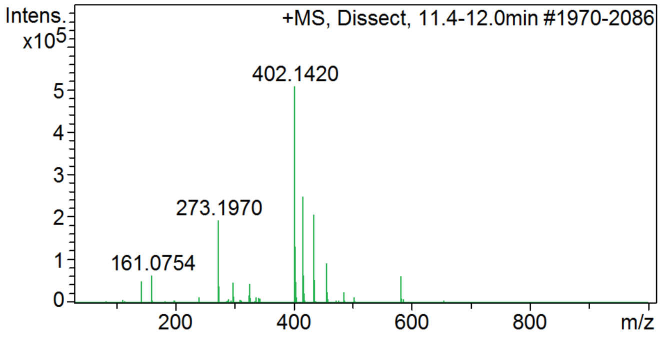

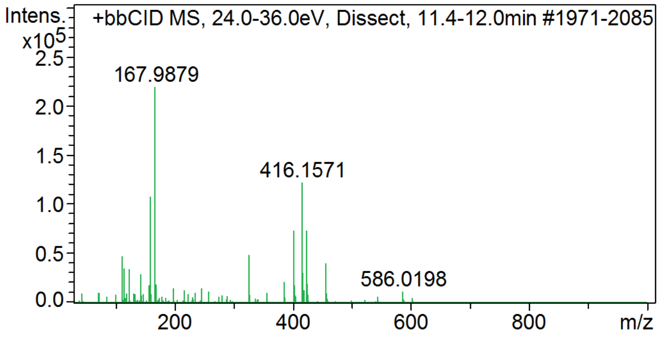

#	m/z	Res.	S/N	I	I %	FWHM
1	161.0754	26636	128.5	65360	12.9	0.0060
2	273.1970	33089	380.2	193381	38.1	0.0083
3	402.1420	38660	998.1	507630	100.0	0.0104
4	403.1452	33763	261.3	132886	26.2	0.0119
5	416.1571	37061	489.8	249142	49.1	0.0112
6	417.1602	32522	127.8	65004	12.8	0.0128
7	434.1671	35156	408.1	207587	40.9	0.0123
8	435.1703	31552	107.2	54513	10.7	0.0138
9	456.1483	33623	183.2	93154	18.4	0.0136
10	581.0646	33254	125.0	63576	12.5	0.0175

#	m/z	Res.	S/N	I	I %	FWHM
1	111.0404	21594	214.9	47357	21.6	0.0051
2	115.0716	22256	159.6	35176	16.0	0.0052
3	123.0763	23624	158.0	34811	15.9	0.0052
4	159.1114	27179	488.9	107739	49.1	0.0059
5	167.9879	28818	996.2	219521	100.0	0.0058
6	326.3672	32962	221.4	48793	22.2	0.0099
7	402.1420	34875	333.6	73517	33.5	0.0115
8	416.1571	37228	554.8	122258	55.7	0.0112
9	424.1231	34160	332.4	73250	33.4	0.0124
10	456.1483	32854	185.1	40786	18.6	0.0139

Cmpd 103, Dissect, 11.8 min

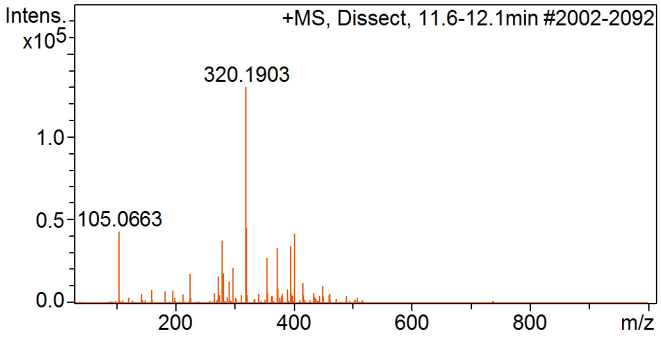

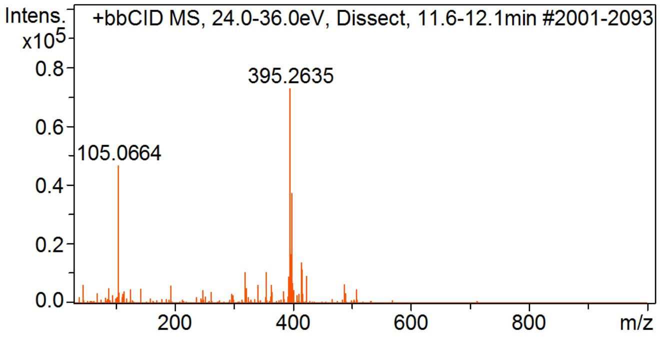

#	m/z	Res.	S/N	I	I %	FWHM
1	105.0663	21756	331.4	43093	33.2	0.0048
2	280.2542	34449	291.6	37918	29.2	0.0081
3	320.1903	33851	998.7	129861	100.0	0.0095
4	320.2454	32339	919.7	119583	92.1	0.0099
5	321.1936	30945	346.6	45063	34.7	0.0104
6	321.2485	30217	261.6	34018	26.2	0.0106
7	355.2724	31097	211.2	27465	21.1	0.0114
8	373.2821	32436	255.1	33164	25.5	0.0115
9	395.2634	31842	264.2	34355	26.5	0.0124
10	402.1420	36940	323.8	42109	32.4	0.0109

#	m/z	Res.	S/N	I	I %	FWHM
1	105.0664	21887	640.3	46797	64.2	0.0048
2	320.2453	33692	146.8	10731	14.7	0.0095
3	355.2723	31370	145.5	10636	14.6	0.0113
4	393.2954	33511	126.5	9244	12.7	0.0117
5	395.2635	33681	997.1	72882	100.0	0.0117
6	396.2668	31047	228.6	16709	22.9	0.0128
7	399.1652	32991	512.5	37457	51.4	0.0121
8	415.1386	32349	190.2	13905	19.1	0.0128
9	416.1571	36222	158.0	11547	15.8	0.0115
10	424.1232	32859	127.6	9330	12.8	0.0129

Compound Spectrum List Report

Cmpd 104, Dissect, 11.8 min

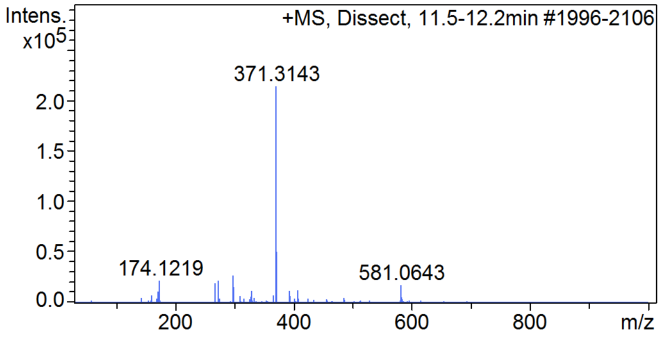

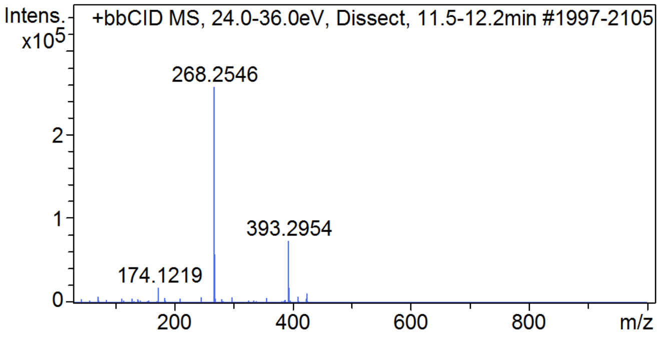

#	m/z	Res.	S/N	I	I %	FWHM
1	174.1219	26524	105.7	22629	10.6	0.0066
2	268.2545	29985	92.8	19875	9.3	0.0089
3	273.1970	32608	104.0	22278	10.4	0.0084
4	298.2640	36170	127.4	27283	12.7	0.0082
5	299.2674	31787	75.5	16164	7.6	0.0094
6	329.3052	31090	58.5	12531	5.9	0.0106
7	371.3143	35562	999.8	214088	100.0	0.0104
8	372.3176	30961	235.9	50522	23.6	0.0120
9	407.1867	31312	61.2	13101	6.1	0.0130
10	581.0643	32676	83.7	17921	8.4	0.0178

#	m/z	Res.	S/N	I	I %	FWHM
1	71.0828	18851	26.8	6902	2.7	0.0038
2	174.1219	26613	72.5	18660	7.3	0.0065
3	268.2546	32870	999.9	257343	100.0	0.0082
4	269.2580	31446	226.5	58282	22.6	0.0086
5	298.2641	32524	26.2	6744	2.6	0.0092
6	393.2954	33344	289.1	74397	28.9	0.0118
7	394.3033	28677	73.3	18856	7.3	0.0137
8	409.2686	29638	29.0	7468	2.9	0.0138
9	425.1261	29841	26.7	6873	2.7	0.0142
10	425.2001	30029	44.8	11521	4.5	0.0142

Cmpd 105, Dissect, 11.9 min

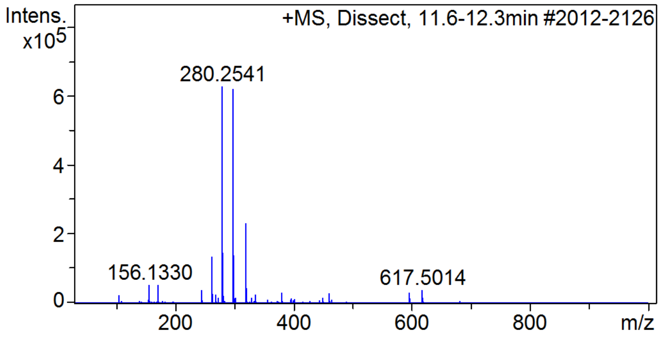

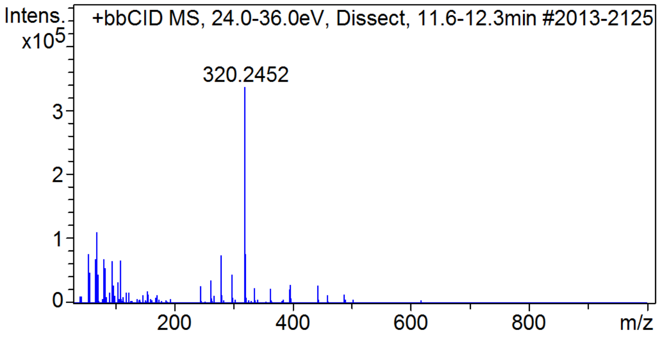

#	m/z	Res.	S/N	I	I %	FWHM
1	156.1330	26676	86.8	54911	8.8	0.0059
2	172.1274	27403	86.4	54599	8.7	0.0063
3	263.2282	31792	214.3	135497	21.6	0.0083
4	280.2541	36269	992.2	627327	100.0	0.0077
5	281.2381	31582	144.9	91600	14.6	0.0089
6	281.2574	31421	231.5	146392	23.3	0.0090
7	298.2639	36887	980.9	620155	98.9	0.0081
8	299.2674	31829	220.9	139660	22.3	0.0094
9	320.1901	35729	226.4	143110	22.8	0.0090
10	320.2453	33757	365.8	231244	36.9	0.0095

#	m/z	Res.	S/N	I	I %	FWHM
1	55.0520	17392	228.0	76971	22.9	0.0032
2	67.0518	18144	203.7	68771	20.4	0.0037
3	69.0672	18644	328.6	110936	32.9	0.0037
4	81.0670	19696	204.5	69055	20.5	0.0041
5	83.0825	19767	163.4	55183	16.4	0.0042
6	95.0824	20755	194.5	65679	19.5	0.0046
7	109.0975	22137	199.1	67211	20.0	0.0049
8	280.2542	32730	221.1	74648	22.2	0.0086
9	320.2452	34949	997.5	336801	100.0	0.0092
10	321.2486	32502	228.4	77125	22.9	0.0099

Compound Spectrum List Report

Cmpd 106, Dissect, 12.1 min

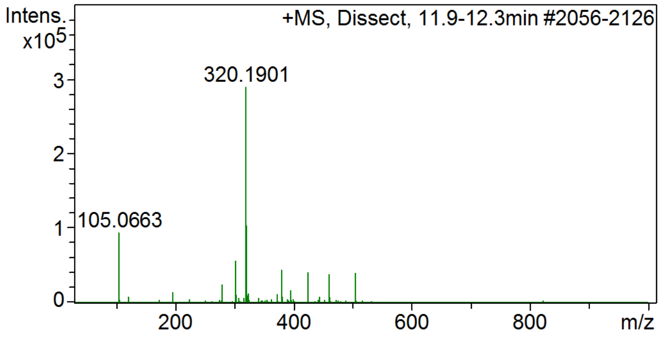

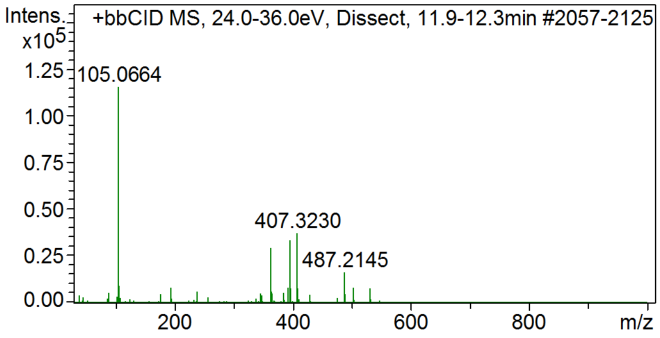

#	m/z	Res.	S/N	I	I %	FWHM
1	105.0663	21910	323.4	94234	32.5	0.0048
2	280.2541	34536	86.8	25296	8.7	0.0081
3	303.1642	31949	196.7	57316	19.8	0.0095
4	320.1901	36701	995.7	290179	100.0	0.0087
5	320.2455	33073	152.6	44459	15.3	0.0097
6	321.1935	33545	357.9	104313	35.9	0.0096
7	380.3240	33042	152.8	44531	15.3	0.0115
8	424.3486	32770	143.5	41807	14.4	0.0129
9	460.2778	32426	135.1	39381	13.6	0.0142
10	504.3025	32386	138.4	40336	13.9	0.0156

#	m/z	Res.	S/N	I	I %	FWHM
1	105.0664	21916	998.9	115638	100.0	0.0048
2	106.0698	21024	80.4	9305	8.0	0.0050
3	195.1159	26159	71.5	8278	7.2	0.0075
4	363.2984	33047	254.6	29471	25.5	0.0110
5	392.3000	30436	72.5	8393	7.3	0.0129
6	395.2634	33028	289.5	33511	29.0	0.0120
7	396.2667	29751	70.4	8145	7.0	0.0133
8	407.3230	34906	322.4	37330	32.3	0.0117
9	487.2145	31270	141.9	16427	14.2	0.0156
10	503.1877	30823	70.9	8210	7.1	0.0163

Cmpd 107, Dissect, 12.1 min

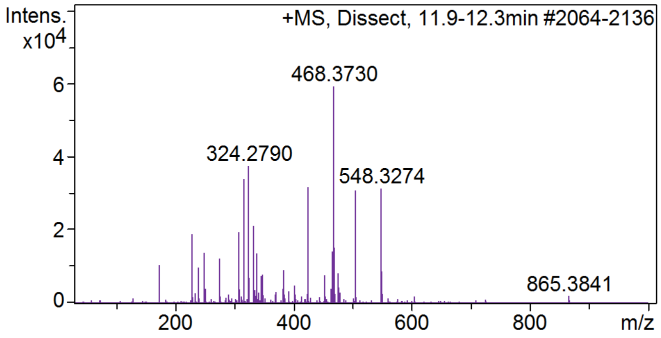

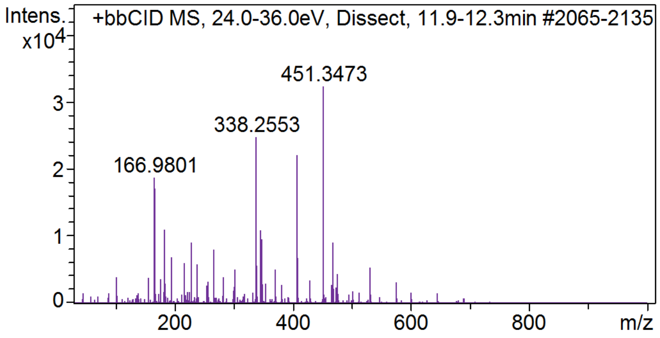

#	m/z	Res.	S/N	I	I %	FWHM
1	229.2279	29019	321.1	19034	32.1	0.0079
2	308.2846	30893	327.5	19411	32.7	0.0100
3	316.2741	31918	574.6	34057	57.5	0.0099
4	324.2790	32826	631.2	37411	63.1	0.0099
5	332.9532	32422	356.9	21153	35.7	0.0103
6	424.3485	32244	535.5	31742	53.6	0.0132
7	468.3730	32530	1000.0	59272	100.0	0.0144
8	469.3764	30437	259.3	15370	25.9	0.0154
9	504.3024	31847	520.2	30835	52.0	0.0158
10	548.3274	31027	530.3	31435	53.0	0.0177

#	m/z	Res.	S/N	I	I %	FWHM
1	166.9801	26796	579.8	18743	58.0	0.0062
2	167.9879	26865	528.3	17076	52.8	0.0063
3	184.0931	25181	341.1	11026	34.1	0.0073
4	229.2278	28250	281.9	9111	28.2	0.0081
5	338.2553	31799	766.1	24765	76.6	0.0106
6	346.2600	33086	338.7	10948	33.9	0.0105
7	348.2756	31225	296.1	9572	29.6	0.0112
8	407.3230	34173	683.5	22095	68.4	0.0119
9	451.3473	32384	1000.0	32324	100.0	0.0139
10	452.3506	32329	322.6	10427	32.3	0.0140

Compound Spectrum List Report

Cmpd 108, Dissect, 12.2 min

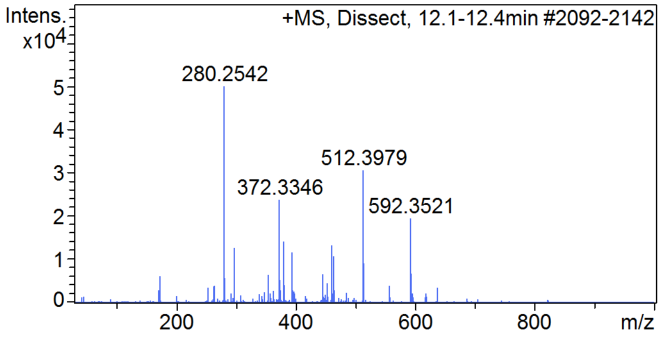

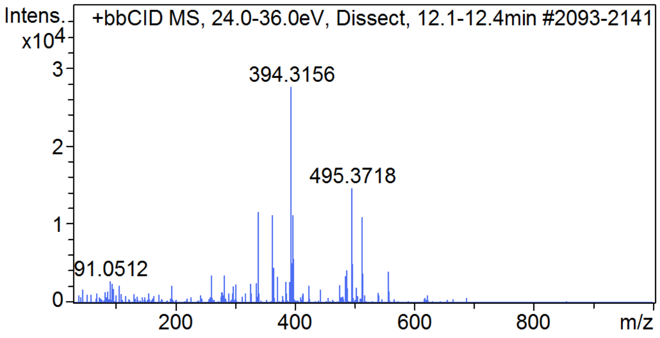

#	m/z	Res.	S/N	I	I %	FWHM
1	280.2542	32134	1000.0	50002	100.0	0.0087
2	298.2641	30233	255.1	12757	25.5	0.0099
3	372.3346	32206	476.4	23823	47.6	0.0116
4	380.3240	29272	285.4	14269	28.5	0.0130
5	394.3158	30866	235.4	11769	23.5	0.0128
6	460.2778	31399	267.7	13385	26.8	0.0147
7	463.2288	31976	215.6	10782	21.6	0.0145
8	512.3979	31531	612.1	30606	61.2	0.0163
9	513.4011	30343	185.3	9266	18.5	0.0169
10	592.3521	31336	390.2	19512	39.0	0.0189

#	m/z	Res.	S/N	I	I %	FWHM
1	339.1463	31757	419.8	11598	42.0	0.0107
2	362.8872	30952	184.1	5088	18.4	0.0117
3	363.2982	31397	405.9	11214	40.6	0.0116
4	394.3156	32787	999.5	27614	100.0	0.0120
5	395.2632	31080	183.0	5057	18.3	0.0127
6	397.2788	30761	407.0	11245	40.7	0.0129
7	398.2823	31051	205.7	5683	20.6	0.0128
8	495.3718	31271	530.7	14663	53.1	0.0158
9	496.3757	31496	180.5	4986	18.1	0.0158
10	512.3982	32378	396.9	10965	39.7	0.0158

Cmpd 109, Dissect, 12.3 min

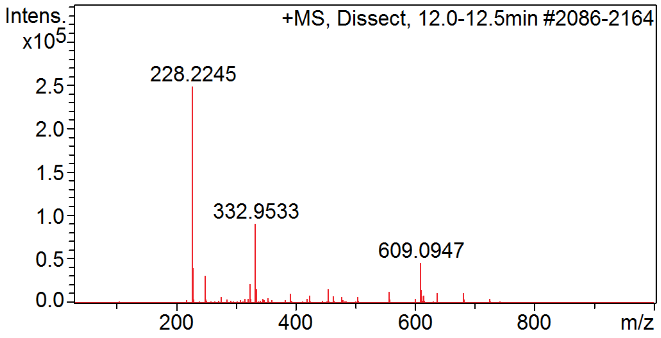

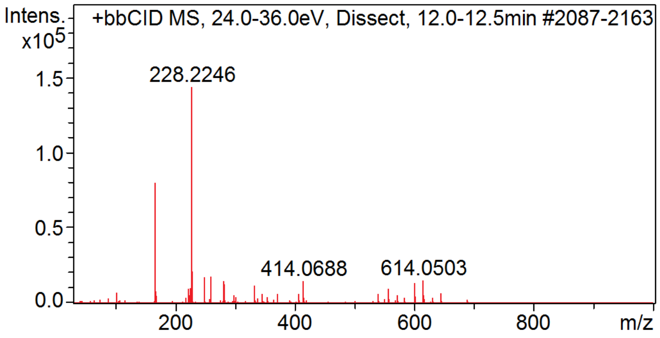

#	m/z	Res.	S/N	I	I %	FWHM
1	228.2245	33563	999.1	248086	100.0	0.0068
2	229.2279	30999	163.1	40497	16.3	0.0074
3	250.2059	31846	127.4	31644	12.8	0.0079
4	324.2789	32767	89.4	22207	9.0	0.0099
5	332.9533	33835	367.9	91354	36.8	0.0098
6	333.9558	29282	57.7	14330	5.8	0.0114
7	334.9493	29882	67.7	16805	6.8	0.0112
8	455.4411	34522	66.3	16460	6.6	0.0132
9	609.0947	32695	187.3	46500	18.7	0.0186
10	610.0983	29843	62.7	15561	6.3	0.0204

#	m/z	Res.	S/N	I	I %	FWHM
1	166.9801	27762	555.7	80071	55.7	0.0060
2	228.2246	32814	996.9	143645	100.0	0.0070
3	229.2279	30217	148.6	21409	14.9	0.0076
4	250.2060	31151	121.1	17445	12.1	0.0080
5	260.0654	31262	125.7	18114	12.6	0.0083
6	282.0463	30410	104.9	15119	10.5	0.0093
7	283.2538	30841	90.1	12978	9.0	0.0092
8	414.0688	31585	105.6	15209	10.6	0.0131
9	600.4479	30811	97.2	14012	9.8	0.0195
10	614.0503	32274	106.6	15365	10.7	0.0190

Compound Spectrum List Report

Cmpd 110, Dissect, 12.4 min

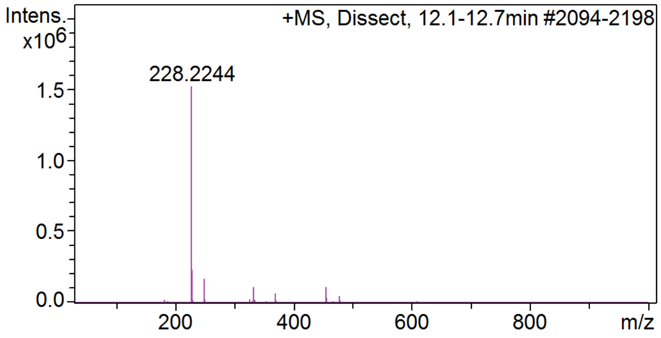

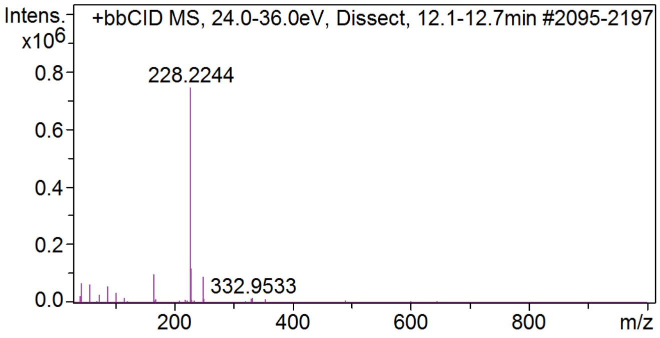

#	m/z	Res.	S/N	I	I %	FWHM
1	228.2244	34267	984.2	1522063	100.0	0.0067
2	229.2278	31655	153.4	237313	15.6	0.0072
3	250.2059	32266	111.4	172269	11.3	0.0078
4	251.2090	28901	17.2	26600	1.7	0.0087
5	326.2581	31032	15.3	23705	1.6	0.0105
6	332.9533	33654	74.7	115505	7.6	0.0099
7	370.2827	34467	42.9	66354	4.4	0.0107
8	455.4412	34618	73.8	114136	7.5	0.0132
9	456.4446	31544	21.6	33395	2.2	0.0145
10	477.4223	32301	29.9	46231	3.0	0.0148

#	m/z	Res.	S/N	I	I %	FWHM
1	41.0368	15047	31.2	23333	3.1	0.0027
2	43.0524	15385	92.5	69186	9.3	0.0028
3	57.0676	17532	87.5	65455	8.8	0.0033
4	74.0573	18765	37.1	27723	3.7	0.0039
5	88.0726	19738	79.2	59211	7.9	0.0045
6	102.0879	20755	48.7	36430	4.9	0.0049
7	166.9801	27734	135.1	101060	13.5	0.0060
8	228.2244	33500	998.7	746757	100.0	0.0068
9	229.2278	31059	161.4	120709	16.2	0.0074
10	250.2059	31515	123.3	92200	12.3	0.0079

Cmpd 111, Dissect, 12.5 min

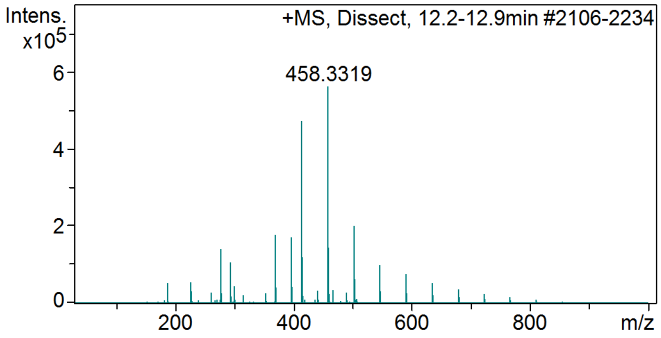

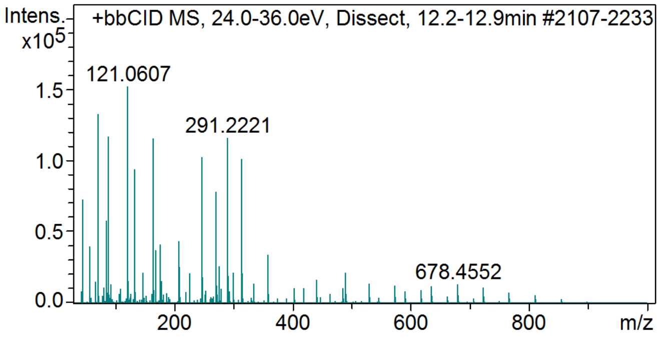

#	m/z	Res.	S/N	I	I %	FWHM
1	278.2386	32935	250.3	141221	25.1	0.0084
2	294.2330	32617	189.7	107057	19.0	0.0090
3	370.2828	34458	317.0	178864	31.8	0.0107
4	397.2815	34289	304.7	171900	30.6	0.0116
5	414.3074	38799	836.5	471984	83.9	0.0107
6	415.3106	33575	213.1	120247	21.4	0.0124
7	458.3319	41201	996.8	562402	100.0	0.0111
8	459.3352	33401	258.2	145709	25.9	0.0138
9	502.3565	35669	357.1	201466	35.8	0.0141
10	546.3812	33324	178.2	100524	17.9	0.0164

#	m/z	Res.	S/N	I	I %	FWHM
1	45.0316	15766	479.5	72962	48.0	0.0029
2	71.0829	19128	871.0	132526	87.2	0.0037
3	89.0566	20403	768.6	116953	76.9	0.0044
4	121.0607	23478	999.3	152047	100.0	0.0052
5	133.0815	24544	616.4	93797	61.7	0.0054
6	165.0854	27326	759.0	115493	76.0	0.0060
7	247.1974	31552	674.4	102615	67.5	0.0078
8	271.1449	31534	515.8	78486	51.6	0.0086
9	291.2221	32769	761.7	115903	76.2	0.0089
10	315.1697	33854	664.8	101161	66.5	0.0093

Compound Spectrum List Report

Cmpd 112, Dissect, 12.6 min

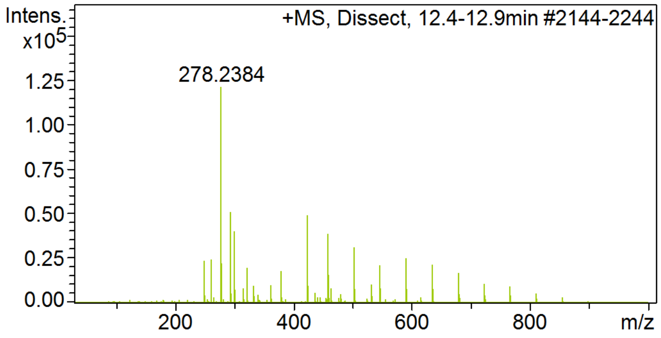

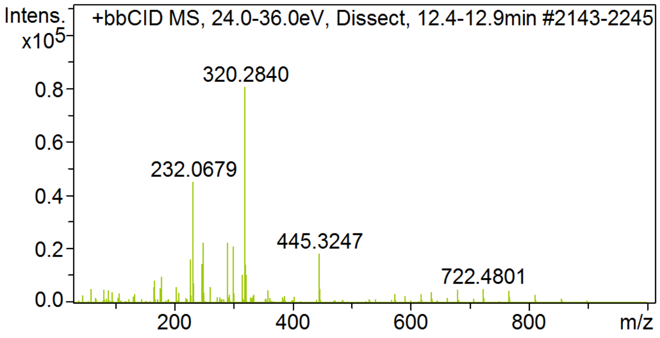

#	m/z	Res.	S/N	I	I %	FWHM
1	250.0778	31964	195.6	23713	19.6	0.0078
2	261.2124	30070	203.5	24674	20.4	0.0087
3	278.2384	33028	999.9	121221	100.0	0.0084
4	279.2418	29844	183.4	22235	18.3	0.0094
5	294.2329	32662	420.7	51002	42.1	0.0090
6	300.2197	32411	331.3	40170	33.1	0.0093
7	423.3434	32054	406.7	49310	40.7	0.0132
8	458.3316	39132	322.2	39061	32.2	0.0117
9	502.3562	35140	258.7	31360	25.9	0.0143
10	590.4056	33443	208.5	25281	20.9	0.0177

#	m/z	Res.	S/N	I	I %	FWHM
1	228.2245	29369	201.8	16265	20.2	0.0078
2	232.0679	32420	559.8	45106	56.0	0.0072
3	247.1973	31478	181.4	14617	18.2	0.0079
4	250.0778	32018	279.5	22520	28.0	0.0078
5	291.2220	32386	279.7	22542	28.0	0.0090
6	300.2198	31472	260.3	20977	26.1	0.0095
7	320.2840	33375	999.3	80522	100.0	0.0096
8	321.2874	31430	179.6	14473	18.0	0.0102
9	322.1185	29571	132.7	10697	13.3	0.0109
10	445.3247	31905	229.9	18523	23.0	0.0140

Cmpd 113, Dissect, 12.7 min

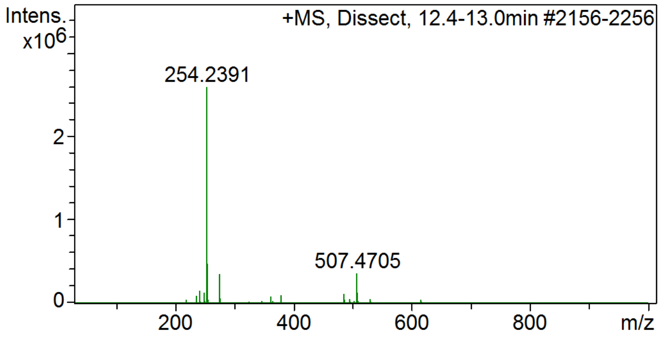

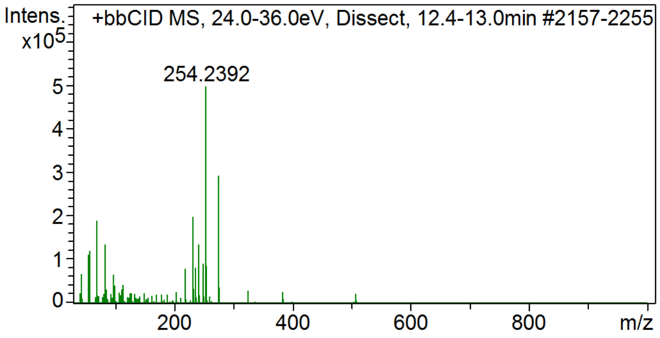

#	m/z	Res.	S/N	I	I %	FWHM
1	237.2133	30884	33.3	88231	3.4	0.0077
2	242.2396	32287	59.5	157807	6.1	0.0075
3	250.0778	32045	51.4	136182	5.2	0.0078
4	254.2391	36054	979.3	2595248	100.0	0.0071
5	255.2426	34155	181.4	480740	18.5	0.0075
6	276.2203	34637	135.8	359900	13.9	0.0080
7	379.2249	32732	35.4	93764	3.6	0.0116
8	485.2726	35985	45.9	121528	4.7	0.0135
9	507.4705	39535	136.0	360533	13.9	0.0128
10	508.4736	35100	50.2	133080	5.1	0.0145

#	m/z	Res.	S/N	I	I %	FWHM
1	55.0519	17558	224.7	112116	22.5	0.0031
2	57.0676	17583	242.6	121066	24.3	0.0032
3	69.0672	18879	379.6	189461	38.1	0.0037
4	83.0825	20245	273.0	136221	27.4	0.0041
5	232.0679	32469	397.7	198474	39.9	0.0071
6	242.2396	31902	272.2	135861	27.3	0.0076
7	250.0779	32069	183.1	91375	18.4	0.0078
8	254.2392	34227	996.5	497301	100.0	0.0074
9	255.2427	31939	173.8	86734	17.4	0.0080
10	276.2205	33843	585.3	292075	58.7	0.0082

Compound Spectrum List Report

Cmpd 114, Dissect, 12.8 min

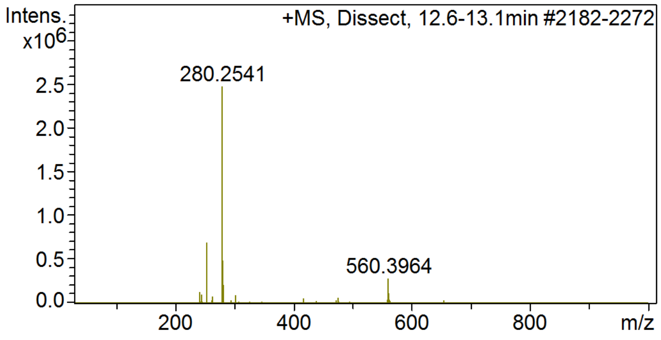

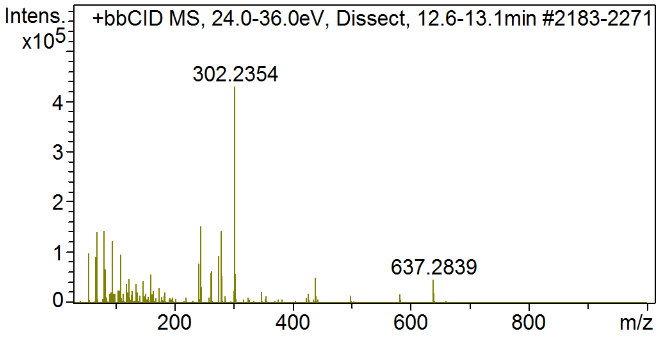

#	m/z	Res.	S/N	I	I %	FWHM
1	242.2397	32306	54.4	134763	5.4	0.0075
2	245.2182	30012	38.7	95720	3.9	0.0082
3	254.2392	34965	281.3	696562	28.1	0.0073
4	280.2541	32648	999.4	2474817	100.0	0.0086
5	281.1058	29009	115.6	286344	11.6	0.0097
6	281.2379	29414	56.4	139662	5.6	0.0096
7	281.2574	29822	198.4	491237	19.8	0.0094
8	282.2685	25852	85.5	211596	8.5	0.0109
9	560.3964	29140	115.3	285387	11.5	0.0192
10	561.4004	28649	49.5	122511	5.0	0.0196

#	m/z	Res.	S/N	I	I %	FWHM
1	55.0520	17361	231.6	99421	23.2	0.0032
2	67.0517	17886	212.5	91214	21.3	0.0037
3	69.0672	18572	328.0	140820	32.8	0.0037
4	81.0669	19352	334.8	143706	33.5	0.0042
5	95.0823	20406	287.5	123414	28.8	0.0047
6	109.0975	22052	226.0	97015	22.6	0.0049
7	245.2181	29462	356.3	152951	35.6	0.0083
8	276.2207	32536	219.2	94097	21.9	0.0085
9	280.2543	31061	335.8	144132	33.6	0.0090
10	302.2354	31010	999.9	429225	100.0	0.0097

Cmpd 115, Dissect, 12.9 min

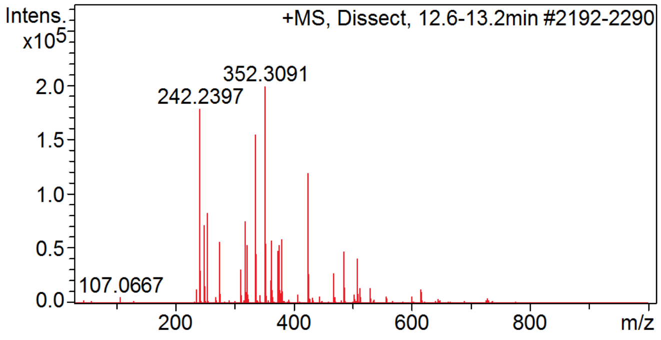

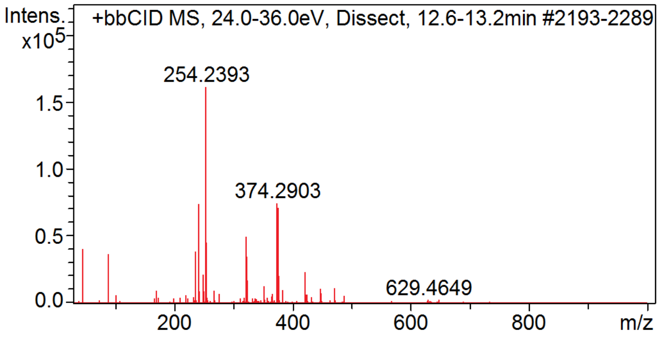

#	m/z	Res.	S/N	I	I %	FWHM
1	242.2397	32190	894.3	177850	89.5	0.0075
2	250.0778	30456	360.1	71603	36.0	0.0082
3	255.2427	31769	417.9	83110	41.8	0.0080
4	275.2490	30869	283.3	56338	28.3	0.0089
5	319.2738	31104	379.3	75429	38.0	0.0103
6	336.3148	33520	777.6	154645	77.8	0.0100
7	352.3091	34402	999.4	198742	100.0	0.0102
8	363.2981	31574	291.1	57896	29.1	0.0115
9	380.3241	31463	294.6	58589	29.5	0.0121
10	425.3519	30235	599.5	119214	60.0	0.0141

#	m/z	Res.	S/N	I	I %	FWHM
1	45.0316	15769	253.0	40753	25.3	0.0029
2	89.0566	19768	229.3	36938	22.9	0.0045
3	237.0831	29449	240.2	38704	24.0	0.0081
4	242.2397	31621	459.1	73967	45.9	0.0077
5	254.2393	31999	999.8	161064	100.0	0.0079
6	255.0930	30152	283.6	45689	28.4	0.0085
7	322.0826	29419	307.8	49593	30.8	0.0109
8	323.2284	21628	218.1	35128	21.8	0.0149
9	374.2903	33826	462.5	74507	46.3	0.0111
10	376.3058	32659	441.8	71181	44.2	0.0115

Compound Spectrum List Report

Cmpd 116, Dissect, 13.0 min

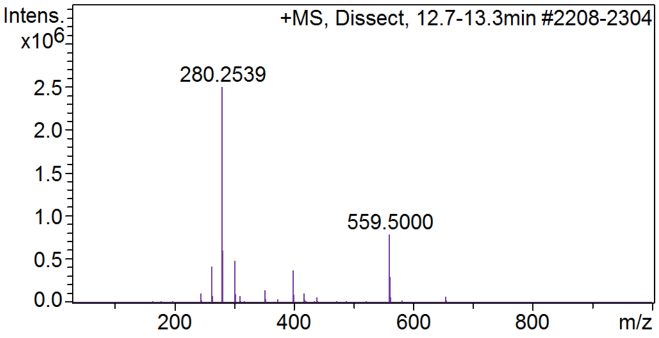

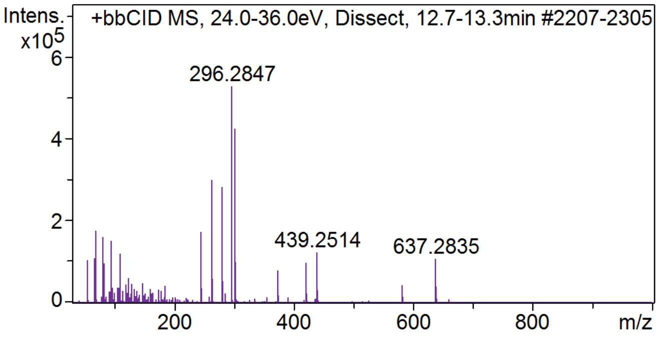

#	m/z	Res.	S/N	I	I %	FWHM
1	245.2180	31002	46.4	116331	4.7	0.0079
2	263.2280	34274	170.3	427244	17.1	0.0077
3	280.2539	35491	996.9	2501729	100.0	0.0079
4	281.2572	35372	242.4	608229	24.3	0.0080
5	302.2351	36472	194.9	489103	19.6	0.0083
6	352.3090	34611	61.6	154582	6.2	0.0102
7	399.3443	38370	150.8	378505	15.1	0.0104
8	417.2702	33120	46.6	116999	4.7	0.0126
9	559.5000	44792	317.0	795382	31.8	0.0125
10	560.5032	39940	123.3	309459	12.4	0.0140

#	m/z	Res.	S/N	I	I %	FWHM
1	69.0672	19003	333.2	176296	33.4	0.0036
2	81.0669	20309	303.2	160452	30.4	0.0040
3	95.0822	21402	285.6	151107	28.6	0.0044
4	109.0974	22482	229.0	121193	23.0	0.0049
5	245.2180	32384	329.0	174109	33.0	0.0076
6	263.2281	33845	566.8	299926	56.8	0.0078
7	280.2540	34541	532.8	281927	53.4	0.0081
8	296.2847	35482	997.9	528067	100.0	0.0084
9	302.2351	35861	800.9	423834	80.3	0.0084
10	439.2514	34131	234.0	123841	23.5	0.0129

Cmpd 117, Dissect, 13.1 min

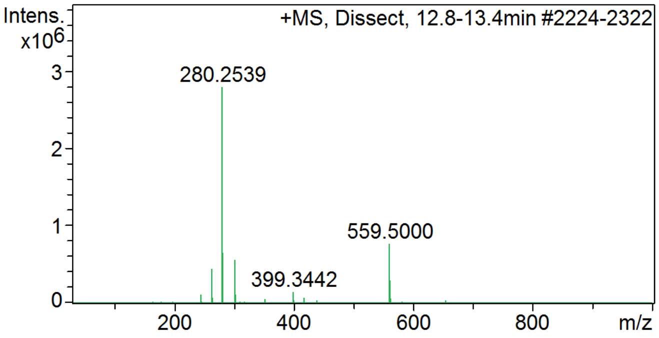

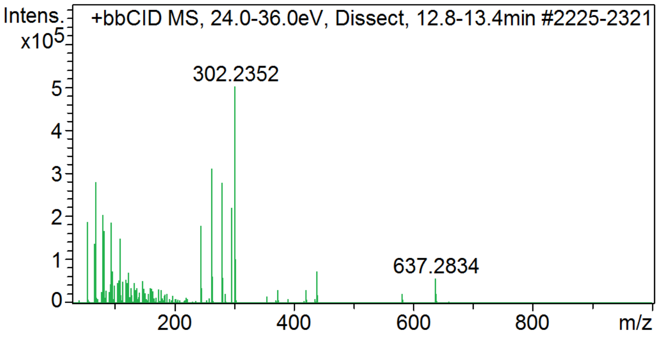

#	m/z	Res.	S/N	I	I %	FWHM
1	245.2179	31181	40.0	113039	4.0	0.0079
2	263.2280	34590	158.9	448893	16.0	0.0076
3	264.2313	31212	26.2	73951	2.6	0.0085
4	280.2539	35621	990.6	2798296	100.0	0.0079
5	281.2572	35714	231.5	653935	23.4	0.0079
6	302.2351	37014	200.5	566275	20.2	0.0082
7	303.2385	32444	39.1	110413	3.9	0.0093
8	399.3442	36433	52.9	149289	5.3	0.0110
9	559.5000	44961	272.8	770727	27.5	0.0124
10	560.5033	39960	107.8	304572	10.9	0.0140

#	m/z	Res.	S/N	I	I %	FWHM
1	55.0520	17694	375.4	189045	37.7	0.0031
2	69.0672	19137	556.2	280080	55.8	0.0036
3	81.0669	20448	405.7	204281	40.7	0.0040
4	83.0824	20451	333.2	167778	33.5	0.0041
5	95.0822	21569	372.4	187537	37.4	0.0044
6	245.2180	32577	358.2	180372	36.0	0.0075
7	263.2281	33999	617.5	310929	62.0	0.0077
8	280.2540	34751	554.3	279099	55.6	0.0081
9	296.2848	34542	439.4	221244	44.1	0.0086
10	302.2352	36244	996.0	501527	100.0	0.0083

Compound Spectrum List Report

Cmpd 118, Dissect, 13.2 min

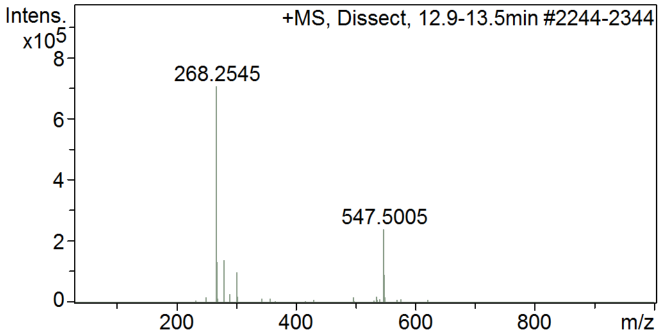

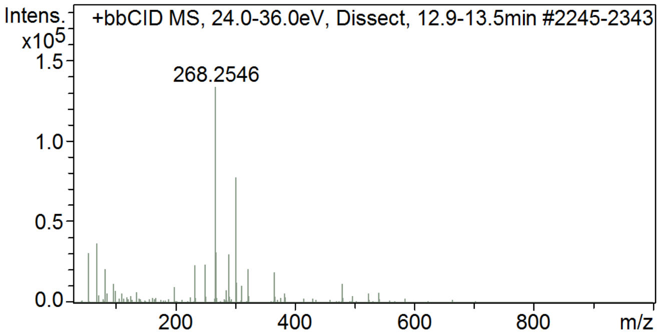

#	m/z	Res.	S/N	I	I %	FWHM
1	268.2545	35750	995.9	705614	100.0	0.0075
2	269.2579	32303	190.4	134865	19.1	0.0083
3	280.2539	37067	196.9	139504	19.8	0.0076
4	290.2358	32333	40.7	28846	4.1	0.0090
5	302.2353	35964	142.7	101094	14.3	0.0084
6	303.2387	31747	26.6	18871	2.7	0.0096
7	535.5009	31791	28.1	19875	2.8	0.0168
8	547.5005	36366	337.9	239432	33.9	0.0151
9	548.5038	32467	131.4	93088	13.2	0.0169
10	549.5072	30120	26.3	18629	2.6	0.0182

#	m/z	Res.	S/N	I	I %	FWHM
1	55.0520	17524	232.1	31133	23.3	0.0031
2	69.0672	18949	273.8	36730	27.5	0.0036
3	83.0825	20151	157.6	21143	15.8	0.0041
4	233.2187	29239	175.6	23554	17.6	0.0080
5	251.2286	29633	178.6	23964	17.9	0.0085
6	268.2546	32019	997.3	133803	100.0	0.0084
7	269.2580	30201	234.7	31493	23.5	0.0089
8	290.2359	31964	226.5	30393	22.7	0.0091
9	302.2353	35108	579.1	77692	58.1	0.0086
10	322.2995	37698	157.4	21118	15.8	0.0085

Cmpd 119, Dissect, 13.2 min

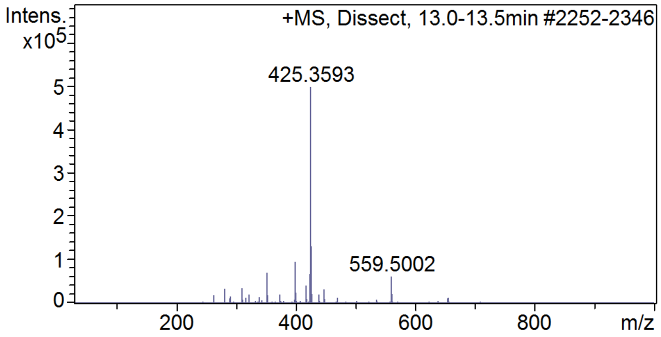

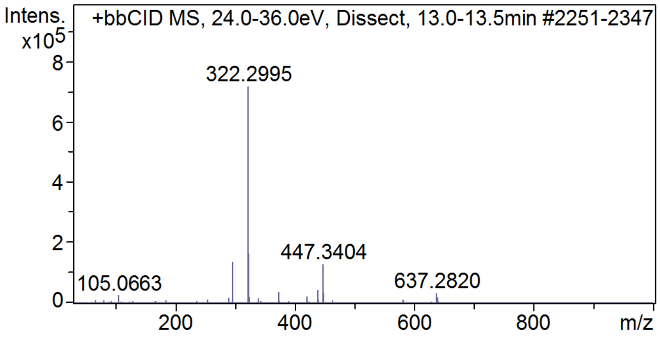

#	m/z	Res.	S/N	I	I %	FWHM
1	281.2574	32766	68.7	34377	6.9	0.0086
2	311.1538	30114	73.3	36662	7.3	0.0103
3	352.3091	30885	143.2	71680	14.4	0.0114
4	399.1641	30514	91.9	45968	9.2	0.0131
5	399.3443	31397	191.6	95877	19.2	0.0127
6	417.2702	30898	84.2	42134	8.4	0.0135
7	424.2491	33277	135.5	67821	13.6	0.0127
8	425.3593	40428	997.3	499037	100.0	0.0105
9	426.3625	34942	262.8	131499	26.4	0.0122
10	559.5002	35891	125.0	62562	12.5	0.0156

#	m/z	Res.	S/N	I	I %	FWHM
1	296.2484	30086	112.9	81103	11.3	0.0098
2	296.2847	30485	192.1	138030	19.2	0.0097
3	297.2877	27892	46.4	33353	4.7	0.0107
4	322.2995	37800	998.2	717261	100.0	0.0085
5	323.3030	32897	231.4	166302	23.2	0.0098
6	374.2901	32251	53.3	38331	5.3	0.0116
7	439.2515	31402	62.1	44610	6.2	0.0140
8	447.3404	34897	180.3	129525	18.1	0.0128
9	448.3436	31210	50.3	36136	5.0	0.0144
10	637.2820	30757	49.2	35381	4.9	0.0207

Compound Spectrum List Report

Cmpd 120, Dissect, 13.3 min

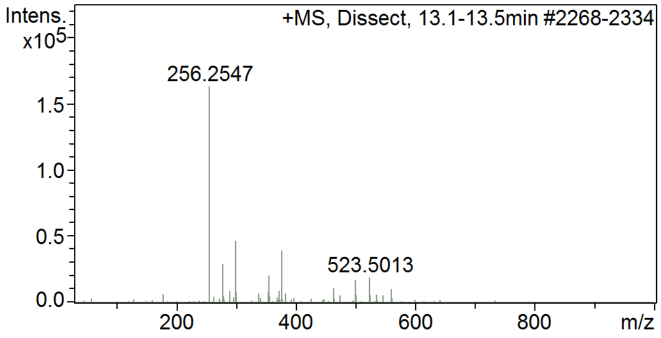

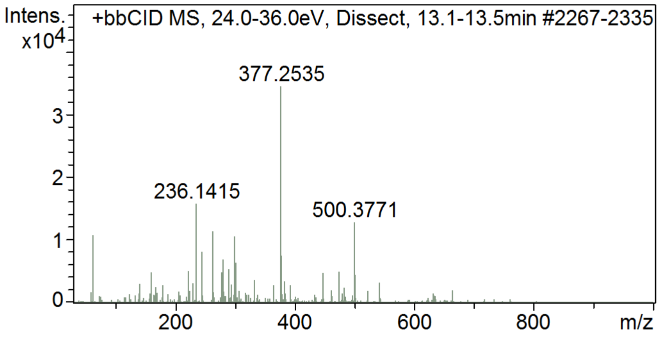

#	m/z	Res.	S/N	I	I %	FWHM
1	256.2547	31833	997.9	163126	100.0	0.0080
2	278.2360	33060	180.4	29484	18.1	0.0084
3	290.2358	32109	57.6	9420	5.8	0.0090
4	300.2797	31638	287.5	46997	28.8	0.0095
5	355.2724	30701	128.8	21057	12.9	0.0116
6	377.2533	32384	243.3	39774	24.4	0.0116
7	463.2287	31539	69.6	11374	7.0	0.0147
8	500.3770	30817	106.1	17337	10.6	0.0162
9	523.5013	31221	119.4	19510	12.0	0.0168
10	559.5001	31588	65.8	10758	6.6	0.0177

#	m/z	Res.	S/N	I	I %	FWHM
1	62.0577	17444	313.1	10808	31.3	0.0036
2	236.1415	29393	459.3	15854	46.0	0.0080
3	245.2181	29407	237.9	8211	23.8	0.0083
4	263.2282	30513	330.7	11415	33.1	0.0086
5	280.2542	31168	201.8	6966	20.2	0.0090
6	300.2795	30754	308.7	10655	30.9	0.0098
7	302.2355	32636	186.7	6443	18.7	0.0093
8	377.2535	31801	999.4	34496	100.0	0.0119
9	378.2566	29395	219.1	7563	21.9	0.0129
10	500.3771	31355	372.5	12859	37.3	0.0160

Cmpd 121, Dissect, 13.4 min

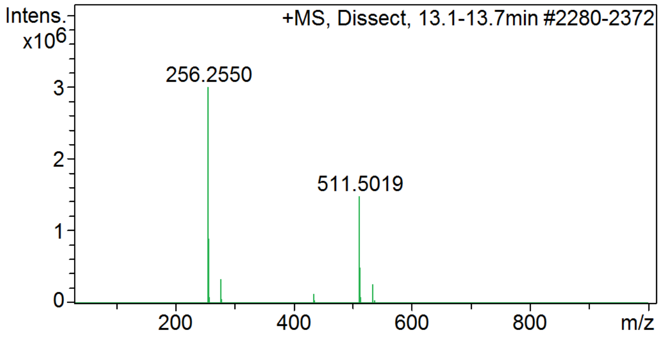

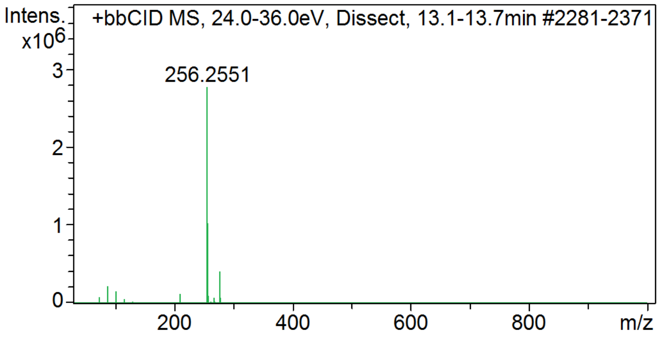

#	m/z	Res.	S/N	I	I %	FWHM
1	256.2550	22941	961.3	3003822	100.0	0.0112
2	257.2581	35734	287.1	896962	29.9	0.0072
3	258.2612	30265	26.3	82270	2.7	0.0085
4	278.2360	35117	109.3	341417	11.4	0.0079
5	434.1567	35999	41.4	129426	4.3	0.0121
6	511.5019	48125	474.6	1482938	49.4	0.0106
7	512.5051	43563	159.5	498303	16.6	0.0118
8	513.5083	33802	28.3	88430	2.9	0.0152
9	533.4831	40017	85.3	266389	8.9	0.0133
10	534.4862	34756	31.3	97722	3.3	0.0154

#	m/z	Res.	S/N	I	I %	FWHM
1	74.0573	19146	28.6	79664	2.9	0.0039
2	88.0725	21057	80.6	224680	8.1	0.0042
3	102.0878	21614	57.3	159761	5.7	0.0047
4	211.1158	31324	43.7	121957	4.4	0.0067
5	256.2551	20837	996.7	2778852	100.0	0.0123
6	257.2581	35915	368.9	1028505	37.0	0.0072
7	258.2612	30864	34.3	95748	3.4	0.0084
8	268.1842	32664	24.4	68020	2.4	0.0082
9	278.2360	35826	147.6	411596	14.8	0.0078
10	279.2393	31519	24.2	67584	2.4	0.0089

Compound Spectrum List Report

Cmpd 122, Dissect, 13.6 min

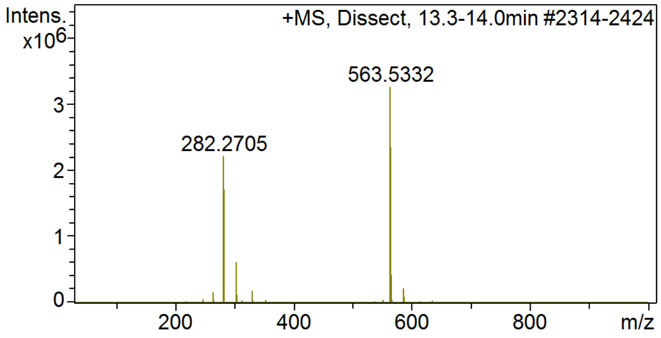

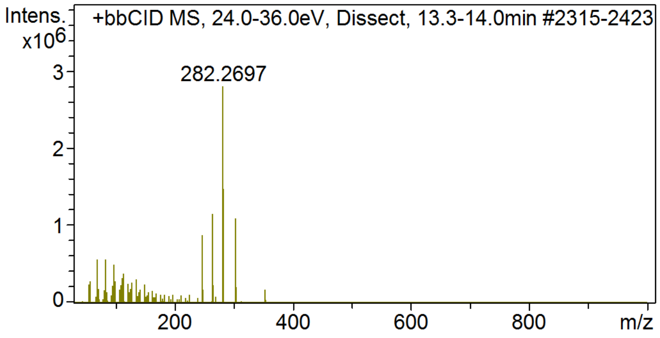

#	m/z	Res.	S/N	I	I %	FWHM
1	265.2436	33196	51.1	168390	5.2	0.0080
2	282.2705	15377	670.8	2210900	67.7	0.0184
3	283.2727	37918	518.9	1710248	52.4	0.0075
4	304.2506	38084	188.4	620762	19.0	0.0080
5	305.2540	32389	36.6	120485	3.7	0.0094
6	331.2729	35154	55.8	183796	5.6	0.0094
7	563.5332	21794	990.6	3264698	100.0	0.0259
8	564.5347	49643	712.1	2346973	71.9	0.0114
9	565.5378	44256	130.5	430148	13.2	0.0128
10	585.5124	39859	68.5	225695	6.9	0.0147

#	m/z	Res.	S/N	I	I %	FWHM
1	69.0672	19429	201.0	567020	20.2	0.0036
2	83.0824	21293	201.5	568444	20.3	0.0039
3	97.0977	22746	177.0	499437	17.8	0.0043
4	111.1130	23552	116.7	329414	11.8	0.0047
5	114.0874	23757	137.4	387645	13.8	0.0048
6	247.2335	35724	310.8	876977	31.3	0.0069
7	265.2435	36693	408.9	1153751	41.2	0.0072
8	282.2697	18140	993.2	2802472	100.0	0.0156
9	283.2727	37968	521.5	1471629	52.5	0.0075
10	304.2506	39076	387.0	1092090	39.0	0.0078

Cmpd 123, Dissect, 13.8 min

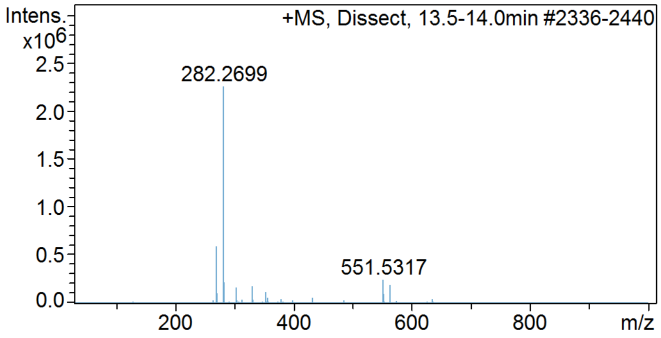

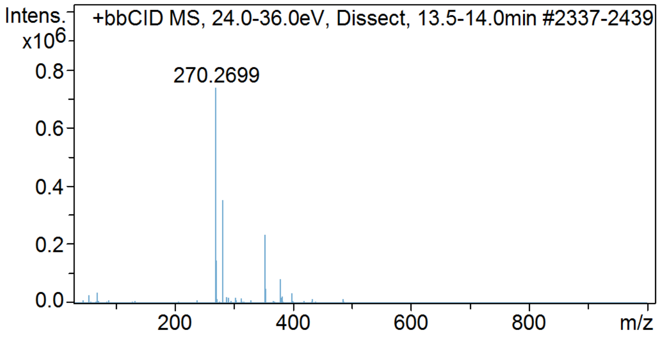

#	m/z	Res.	S/N	I	I %	FWHM
1	270.2700	34170	260.4	594283	26.3	0.0079
2	271.2734	30784	47.9	109380	4.8	0.0088
3	282.2699	22976	988.5	2255915	100.0	0.0123
4	283.2728	36970	96.2	219565	9.7	0.0077
5	304.2507	36386	73.7	168147	7.5	0.0084
6	331.2728	33897	78.9	180034	8.0	0.0098
7	353.2542	33129	54.2	123704	5.5	0.0107
8	551.5317	34928	110.6	252354	11.2	0.0158
9	552.5348	32016	45.4	103539	4.6	0.0173
10	563.5320	30150	85.2	194540	8.6	0.0187

#	m/z	Res.	S/N	I	I %	FWHM
1	55.0519	17591	37.6	27826	3.8	0.0031
2	69.0672	19166	50.5	37394	5.1	0.0036
3	270.2699	34826	997.7	739094	100.0	0.0078
4	271.2734	31805	198.7	147227	19.9	0.0085
5	282.2696	24568	478.1	354159	47.9	0.0115
6	353.2542	36188	317.3	235028	31.8	0.0098
7	354.2576	31673	68.3	50603	6.8	0.0112
8	379.2690	33638	114.3	84654	11.5	0.0113
9	383.1910	32307	31.5	23332	3.2	0.0119
10	399.3120	30837	49.8	36879	5.0	0.0129

Compound Spectrum List Report

Cmpd 124, Dissect, 13.9 min

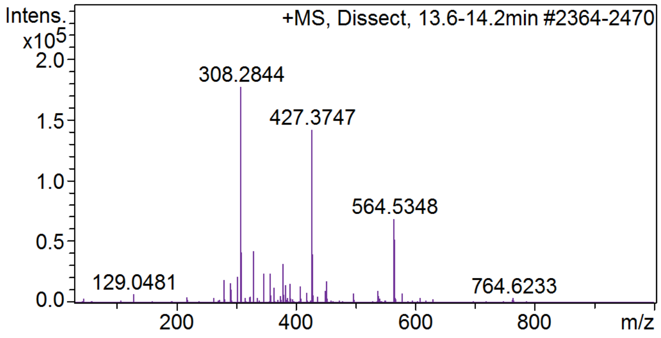

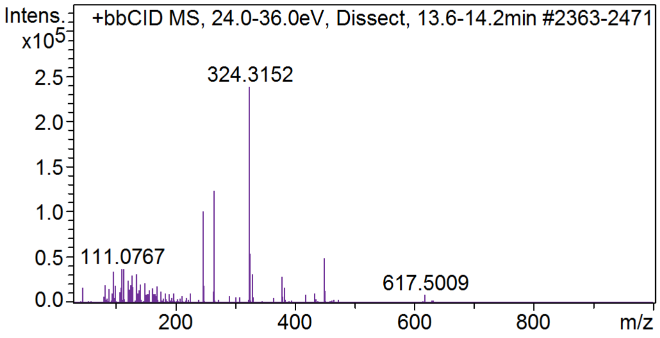

#	m/z	Res.	S/N	I	I %	FWHM
1	308.2844	32951	999.4	177499	100.0	0.0094
2	309.2878	30993	235.2	41775	23.5	0.0100
3	330.2656	30866	240.7	42747	24.1	0.0107
4	347.3037	30478	137.3	24377	13.7	0.0114
5	357.2879	32914	136.6	24263	13.7	0.0109
6	379.2690	32324	181.1	32169	18.1	0.0117
7	427.3747	33935	798.7	141843	79.9	0.0126
8	428.3778	31346	225.0	39951	22.5	0.0137
9	564.5348	33178	386.5	68636	38.7	0.0170
10	565.5449	28587	292.6	51964	29.3	0.0198

#	m/z	Res.	S/N	I	I %	FWHM
1	97.0979	19965	147.4	35135	14.8	0.0049
2	111.0767	21088	157.6	37567	15.8	0.0053
3	111.1131	21596	154.8	36888	15.5	0.0051
4	114.0876	21872	156.7	37342	15.7	0.0052
5	135.1122	24220	135.7	32332	13.6	0.0056
6	247.2338	30128	424.7	101213	42.5	0.0082
7	265.2437	30523	519.5	123801	52.0	0.0087
8	324.3152	34285	999.4	238182	100.0	0.0095
9	325.3186	31180	229.1	54595	22.9	0.0104
10	449.3566	31642	207.7	49504	20.8	0.0142

Cmpd 125, Dissect, 14.0 min

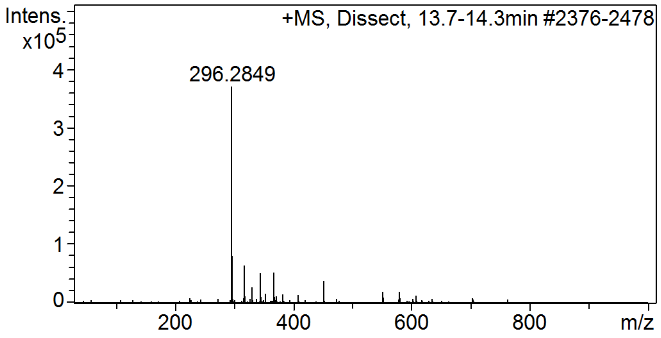

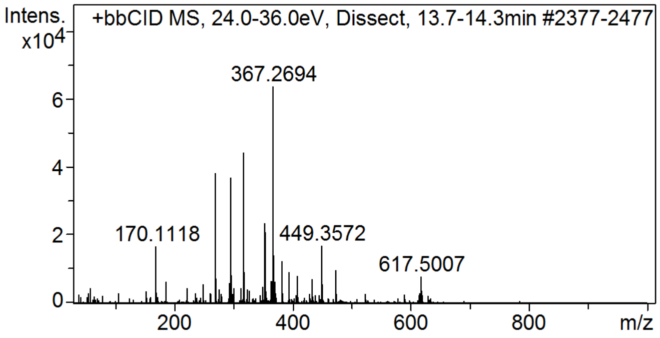

#	m/z	Res.	S/N	I	I %	FWHM
1	296.2485	30215	49.9	18557	5.0	0.0098
2	296.2849	34438	997.4	370732	100.0	0.0086
3	297.2883	31420	217.7	80911	21.8	0.0095
4	318.2662	32312	173.4	64465	17.4	0.0098
5	331.2720	28528	73.7	27406	7.4	0.0116
6	345.2885	32514	138.5	51469	13.9	0.0106
7	367.2695	32893	142.9	53105	14.3	0.0112
8	451.3737	31972	103.8	38589	10.4	0.0141
9	551.5308	30551	53.5	19869	5.4	0.0181
10	579.5619	31431	55.1	20489	5.5	0.0184

#	m/z	Res.	S/N	I	I %	FWHM
1	170.1118	26645	263.1	16754	26.3	0.0064
2	270.2700	32293	598.7	38125	59.9	0.0084
3	296.2482	30938	270.6	17232	27.1	0.0096
4	296.2848	31341	578.9	36860	57.9	0.0095
5	318.2660	32345	693.3	44149	69.3	0.0098
6	353.2544	29625	370.5	23594	37.1	0.0119
7	355.0579	31145	328.4	20914	32.9	0.0114
8	367.2694	34057	999.8	63664	100.0	0.0108
9	368.2728	30629	222.2	14150	22.2	0.0120
10	449.3572	31232	267.2	17014	26.7	0.0144

Compound Spectrum List Report

Cmpd 126, Dissect, 14.2 min

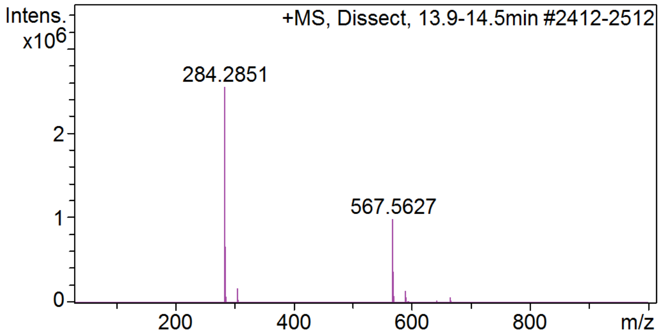

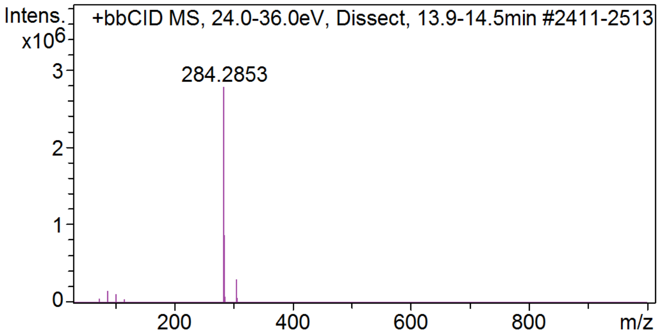

#	m/z	Res.	S/N	I	I %	FWHM
1	284.2851	30659	988.7	2550195	100.0	0.0093
2	285.2885	36661	257.2	663315	26.0	0.0078
3	286.2916	30250	27.4	70709	2.8	0.0095
4	306.2664	34289	67.7	174678	6.8	0.0089
5	567.5627	47406	384.3	991300	38.9	0.0120
6	568.5659	42333	145.5	375164	14.7	0.0134
7	569.5691	32686	29.5	76039	3.0	0.0174
8	589.5438	35751	57.4	147993	5.8	0.0165
9	590.5469	32065	24.0	62023	2.4	0.0184
10	664.5621	32151	26.2	67568	2.6	0.0207

#	m/z	Res.	S/N	I	I %	FWHM
1	74.0572	19057	18.6	51876	1.9	0.0039
2	88.0725	20793	57.8	161117	5.8	0.0042
3	102.0878	21434	41.1	114438	4.1	0.0048
4	116.1030	22726	16.4	45596	1.6	0.0051
5	284.2853	23607	999.4	2784749	100.0	0.0120
6	285.2690	31797	8.6	23927	0.9	0.0090
7	285.2884	37395	313.0	872126	31.3	0.0076
8	286.2916	30986	28.5	79420	2.9	0.0092
9	306.2663	36327	111.4	310460	11.1	0.0084
10	307.2696	32030	22.2	61883	2.2	0.0096

Cmpd 127, Dissect, 14.3 min

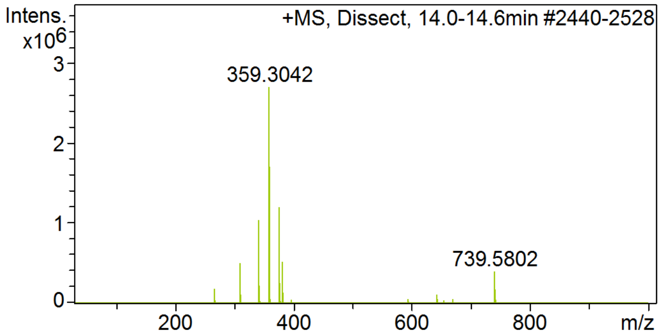

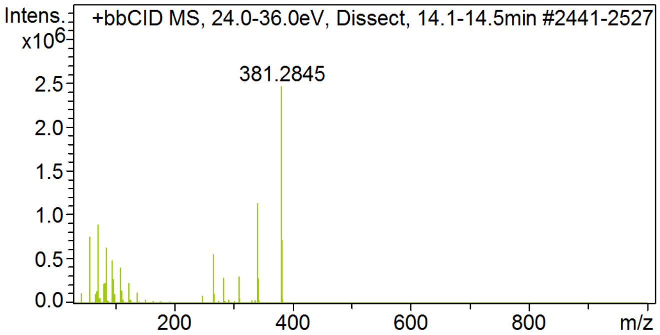

#	m/z	Res.	S/N	I	I %	FWHM
1	267.2589	33445	67.6	186951	6.9	0.0080
2	310.2997	36668	184.4	509783	18.8	0.0085
3	341.2931	40055	378.1	1045285	38.6	0.0085
4	342.2966	36225	82.6	228499	8.4	0.0094
5	359.3042	18075	978.4	2704928	100.0	0.0199
6	360.3064	41612	617.0	1705751	63.1	0.0087
7	376.3288	42112	434.2	1200404	44.4	0.0089
8	377.3323	36962	94.4	261042	9.7	0.0102
9	381.2841	40771	189.3	523348	19.3	0.0094
10	739.5802	42029	145.7	402712	14.9	0.0176

#	m/z	Res.	S/N	I	I %	FWHM
1	57.0675	17957	309.0	760844	30.9	0.0032
2	71.0828	19950	364.4	897097	36.4	0.0036
3	85.0980	21741	256.7	632021	25.7	0.0039
4	95.0821	22418	198.8	489327	19.9	0.0042
5	109.0974	23549	165.3	406878	16.5	0.0046
6	267.2590	36733	228.2	561821	22.8	0.0073
7	310.2999	35530	125.5	308957	12.6	0.0087
8	341.2933	38272	460.7	1134230	46.1	0.0089
9	381.2845	38063	999.7	2461302	100.0	0.0100
10	382.2878	41254	292.9	721148	29.3	0.0093

Compound Spectrum List Report

Cmpd 128, Dissect, 14.3 min

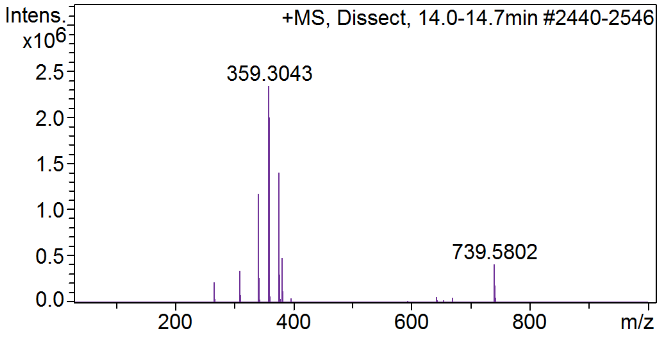

#	m/z	Res.	S/N	I	I %	FWHM
1	267.2589	33544	93.8	223498	9.6	0.0080
2	310.2996	35505	146.3	348868	14.9	0.0087
3	341.2931	40166	491.5	1171608	50.1	0.0085
4	342.2966	36346	113.0	269351	11.5	0.0094
5	359.3043	17402	980.2	2336720	100.0	0.0206
6	360.3064	41764	835.7	1992159	85.3	0.0086
7	376.3288	42297	587.5	1400579	59.9	0.0089
8	377.3323	37168	129.3	308350	13.2	0.0102
9	381.2841	40778	202.9	483647	20.7	0.0094
10	739.5802	42801	175.4	418131	17.9	0.0173

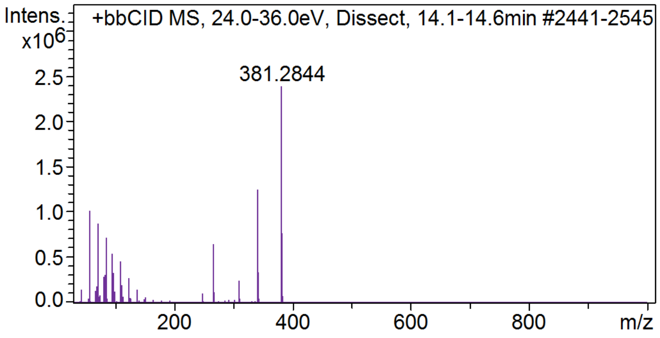

#	m/z	Res.	S/N	I	I %	FWHM
1	57.0675	17961	425.7	1017026	42.6	0.0032
2	71.0828	19993	368.1	879468	36.8	0.0036
3	85.0980	21814	302.0	721551	30.2	0.0039
4	95.0821	22457	228.0	544621	22.8	0.0042
5	109.0973	23609	192.7	460464	19.3	0.0046
6	267.2589	36907	271.6	648897	27.2	0.0072
7	341.2932	38544	522.3	1247811	52.3	0.0089
8	342.2966	39449	142.1	339521	14.2	0.0087
9	381.2844	38061	999.1	2386885	100.0	0.0100
10	382.2877	41459	323.7	773288	32.4	0.0092

Cmpd 129, Dissect, 14.4 min

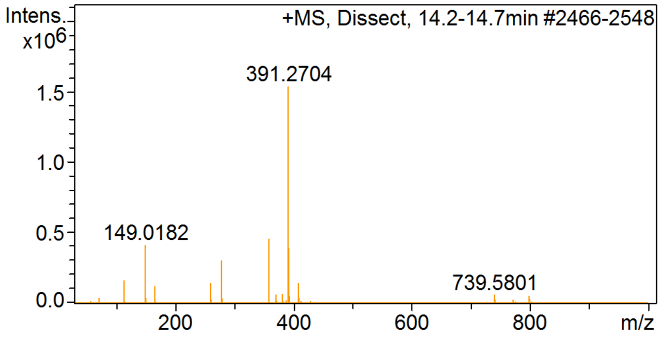

#	m/z	Res.	S/N	I	I %	FWHM
1	113.1285	22135	106.7	164018	10.7	0.0051
2	149.0182	27067	268.4	412498	26.9	0.0055
3	167.0281	27163	81.3	124953	8.1	0.0061
4	261.1394	32430	94.8	145714	9.5	0.0081
5	279.1494	34285	197.8	304000	19.8	0.0081
6	359.3037	20570	300.0	461087	30.0	0.0175
7	381.2841	40569	42.4	65169	4.2	0.0094
8	391.2704	43199	998.6	1534966	100.0	0.0091
9	392.2738	39438	255.4	392587	25.6	0.0099
10	408.2963	35100	94.8	145725	9.5	0.0116

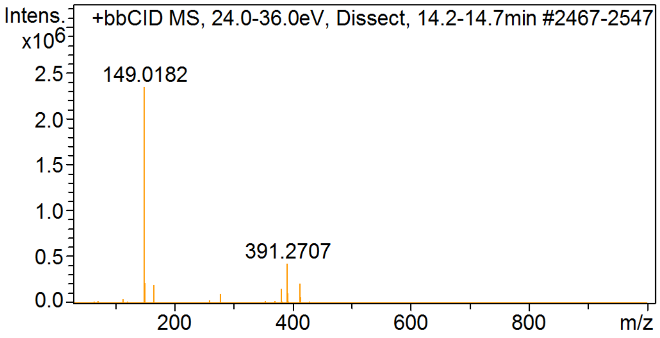

#	m/z	Res.	S/N	I	I %	FWHM
1	113.1285	22077	18.3	46860	2.0	0.0051
2	149.0182	28245	914.1	2346830	100.0	0.0053
3	150.0215	26949	88.6	227397	9.7	0.0056
4	167.0282	28223	79.2	203279	8.7	0.0059
5	279.1496	31877	38.0	97432	4.2	0.0088
6	381.2843	39985	62.4	160249	6.8	0.0095
7	391.2707	39701	168.5	432496	18.4	0.0099
8	392.2740	35208	43.2	110965	4.7	0.0111
9	413.2518	37304	84.1	215977	9.2	0.0111
10	414.2552	34023	24.3	62404	2.7	0.0122

Compound Spectrum List Report

Cmpd 130, Dissect, 14.5 min

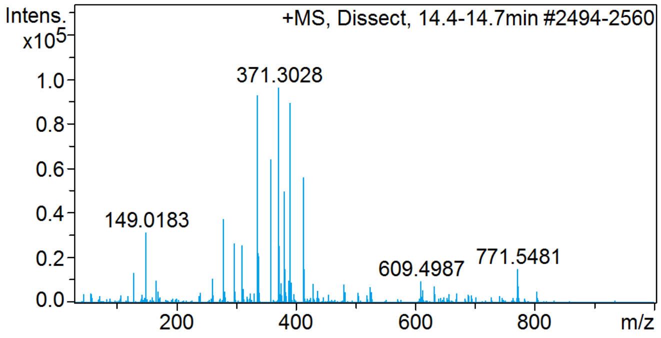

#	m/z	Res.	S/N	I	I %	FWHM
1	149.0183	26935	326.5	31414	32.7	0.0055
2	279.1496	33685	390.8	37603	39.1	0.0083
3	298.3005	30742	277.2	26670	27.7	0.0097
4	310.2998	30944	268.9	25875	26.9	0.0100
5	336.3147	33743	965.3	92881	96.5	0.0100
6	359.3032	33224	664.8	63971	66.5	0.0108
7	371.3028	34248	999.8	96205	100.0	0.0108
8	381.2843	38427	517.8	49826	51.8	0.0099
9	391.2707	41857	927.4	89233	92.8	0.0093
10	413.2519	32387	584.3	56223	58.4	0.0128

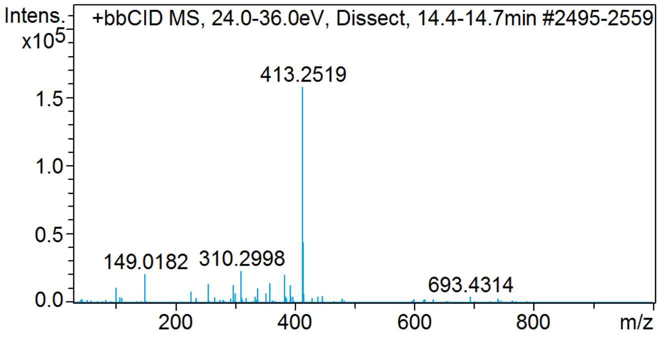

#	m/z	Res.	S/N	I	I %	FWHM
1	101.0562	21101	73.3	11565	7.3	0.0048
2	149.0182	28364	133.9	21128	13.4	0.0053
3	256.2548	29749	89.8	14168	9.0	0.0086
4	298.3002	31855	83.3	13145	8.3	0.0094
5	310.2998	31252	148.0	23363	14.8	0.0099
6	358.2957	30688	94.1	14851	9.4	0.0117
7	383.1909	33308	131.7	20780	13.2	0.0115
8	393.2830	28867	84.9	13402	8.5	0.0136
9	413.2519	37276	997.5	157432	100.0	0.0111
10	414.2553	34577	280.8	44314	28.1	0.0120

Cmpd 131, Dissect, 14.6 min

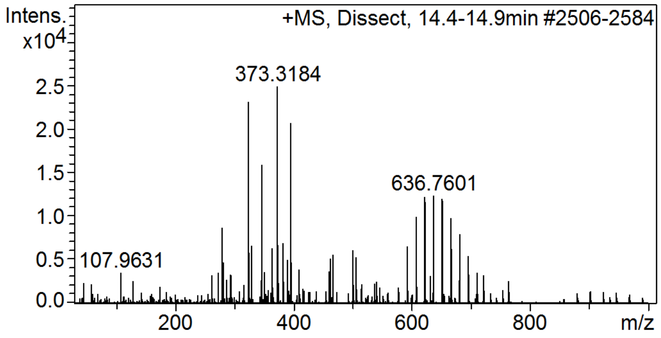

#	m/z	Res.	S/N	I	I %	FWHM
1	324.3152	31012	927.7	23106	92.8	0.0105
2	347.1492	38181	638.4	15900	63.9	0.0091
3	373.3184	31111	999.4	24892	100.0	0.0120
4	395.2996	30493	828.1	20626	82.9	0.0130
5	621.7508	35684	489.8	12199	49.0	0.0174
6	622.0856	35345	468.5	11669	46.9	0.0176
7	636.4260	33816	481.3	11988	48.2	0.0188
8	636.7601	34414	496.6	12369	49.7	0.0185
9	651.1004	36055	481.0	11981	48.1	0.0181
10	651.4353	33668	473.8	11801	47.4	0.0193

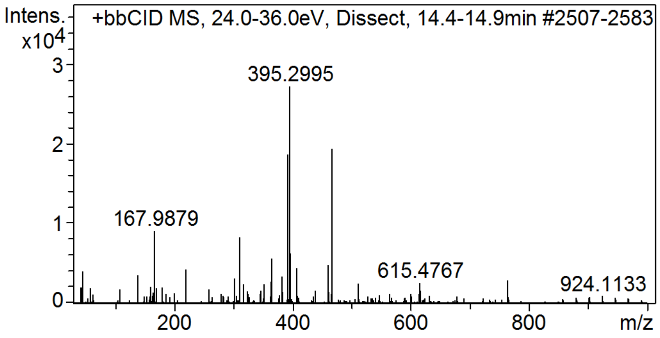

#	m/z	Res.	S/N	I	I %	FWHM
1	167.9879	26654	335.0	9117	33.5	0.0063
2	311.2840	29794	305.0	8300	30.5	0.0104
3	365.0930	31631	207.4	5644	20.7	0.0115
4	392.1941	31675	684.5	18629	68.5	0.0124
5	395.2995	32021	999.9	27213	100.0	0.0123
6	396.3031	29868	230.2	6264	23.0	0.0133
7	407.3491	31634	162.6	4426	16.3	0.0129
8	460.3602	30948	178.0	4843	17.8	0.0149
9	466.5181	31522	713.0	19405	71.3	0.0148
10	467.5216	30669	223.0	6068	22.3	0.0152

Compound Spectrum List Report

Cmpd 132, Dissect, 14.7 min

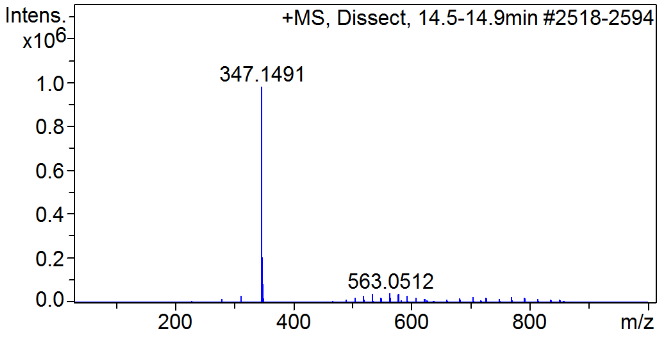

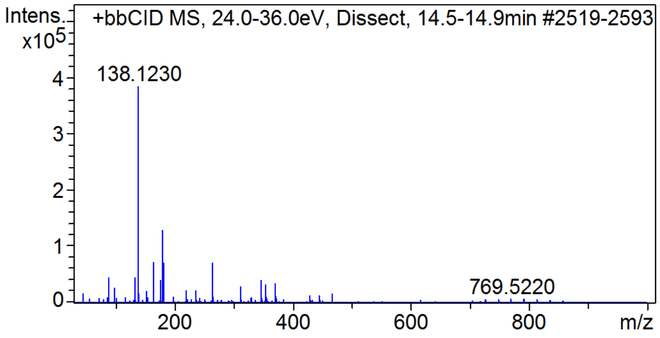

#	m/z	Res.	S/N	I	I %	FWHM
1	347.1491	39316	999.6	979662	100.0	0.0088
2	348.1523	32782	211.9	207677	21.2	0.0106
3	349.1458	26573	87.0	85266	8.7	0.0131
4	519.0262	36871	32.3	31645	3.2	0.0141
5	533.7012	36142	38.9	38138	3.9	0.0148
6	534.0354	36125	34.4	33707	3.4	0.0148
7	563.0512	35304	43.2	42367	4.3	0.0159
8	563.3855	36193	41.0	40177	4.1	0.0156
9	577.7261	35970	37.4	36693	3.7	0.0161
10	578.0605	37197	38.8	38051	3.9	0.0155

#	m/z	Res.	S/N	I	I %	FWHM
1	89.0565	20160	117.9	45371	11.8	0.0044
2	133.0814	24600	120.7	46416	12.1	0.0054
3	138.1230	26402	999.4	384425	100.0	0.0052
4	165.9723	27458	189.4	72843	18.9	0.0060
5	177.1062	28338	108.2	41626	10.8	0.0062
6	180.1685	29088	337.9	129969	33.8	0.0062
7	181.1760	27135	261.6	100628	26.2	0.0067
8	182.9983	28882	187.0	71942	18.7	0.0063
9	265.0738	32701	188.1	72363	18.8	0.0081
10	347.1492	32261	108.5	41720	10.9	0.0108

Cmpd 133, Dissect, 14.8 min

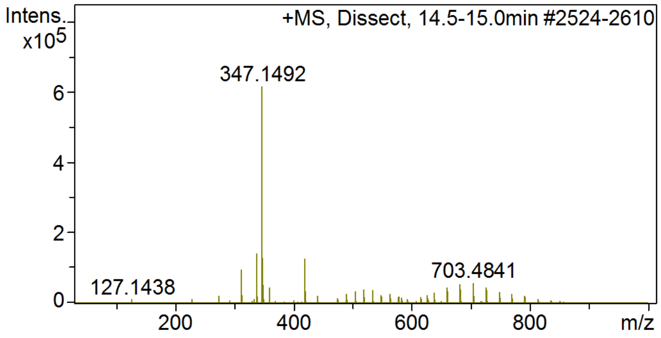

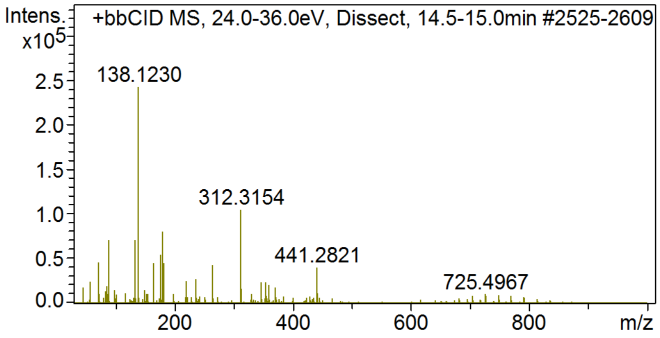

#	m/z	Res.	S/N	I	I %	FWHM
1	312.3156	32386	158.3	97501	15.8	0.0096
2	338.3302	37366	231.3	142469	23.1	0.0091
3	347.1492	38076	999.7	615659	100.0	0.0091
4	348.1523	31763	209.8	129226	21.0	0.0110
5	349.1458	26330	87.6	53970	8.8	0.0133
6	419.3011	35603	207.4	127734	20.7	0.0118
7	659.4591	34684	76.4	47058	7.6	0.0190
8	681.4718	33378	90.5	55727	9.1	0.0204
9	703.4841	33986	96.3	59284	9.6	0.0207
10	725.4962	31556	76.4	47043	7.6	0.0230

#	m/z	Res.	S/N	I	I %	FWHM
1	71.0828	19367	191.6	46491	19.2	0.0037
2	89.0565	20349	292.8	71035	29.3	0.0044
3	133.0814	24551	293.8	71276	29.4	0.0054
4	138.1230	26023	1000.0	242600	100.0	0.0053
5	165.9723	27275	187.6	45506	18.8	0.0061
6	177.1062	28284	224.6	54498	22.5	0.0063
7	180.1685	28555	332.2	80604	33.2	0.0063
8	181.1759	26850	250.4	60738	25.0	0.0067
9	182.9983	28675	187.0	45372	18.7	0.0064
10	312.3154	33020	433.2	105089	43.3	0.0095

Compound Spectrum List Report

Cmpd 134, Dissect, 14.8 min

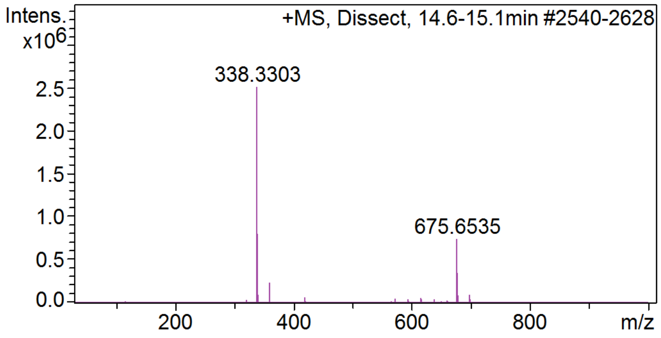

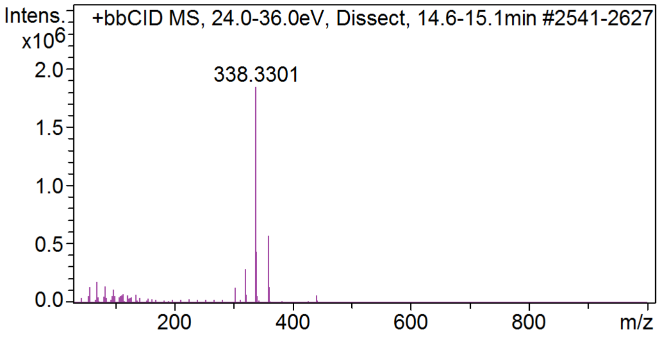

#	m/z	Res.	S/N	I	I %	FWHM
1	338.3303	37155	997.0	2516248	100.0	0.0091
2	339.3337	39080	318.8	804630	32.0	0.0087
3	340.3370	32618	37.5	94737	3.8	0.0104
4	360.3115	36346	94.2	237792	9.5	0.0099
5	419.3012	36318	24.2	61163	2.4	0.0115
6	615.4352	33798	21.9	55233	2.2	0.0182
7	675.6535	46282	295.6	746072	29.7	0.0146
8	676.6567	40294	140.2	353897	14.1	0.0168
9	677.6597	31814	33.5	84476	3.4	0.0213
10	697.6344	31994	36.2	91400	3.6	0.0218

#	m/z	Res.	S/N	I	I %	FWHM
1	57.0676	17745	76.2	140851	7.6	0.0032
2	69.0672	18938	98.8	182691	9.9	0.0036
3	83.0825	20472	78.7	145524	7.9	0.0041
4	97.0979	21082	63.9	118166	6.4	0.0046
5	303.2943	32880	72.6	134356	7.3	0.0092
6	321.3044	35331	156.6	289667	15.7	0.0091
7	338.3301	40246	998.6	1846800	100.0	0.0084
8	339.3336	36976	238.2	440584	23.9	0.0092
9	360.3114	38690	312.4	577709	31.3	0.0093
10	361.3146	33367	73.9	136746	7.4	0.0108

Cmpd 135, Dissect, 14.9 min

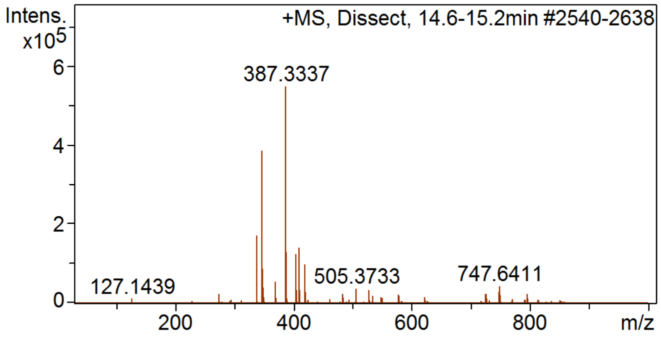

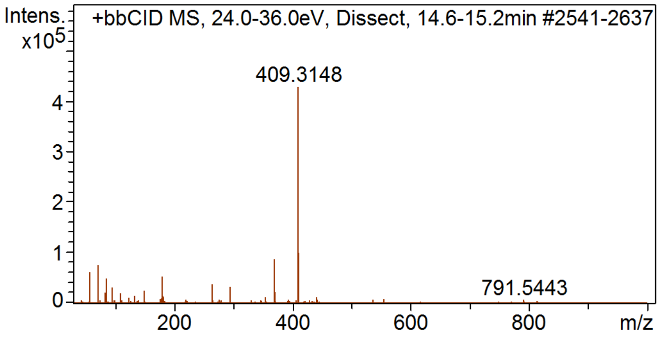

#	m/z	Res.	S/N	I	I %	FWHM
1	338.3303	37862	315.0	172582	31.5	0.0089
2	347.1495	32713	702.7	384978	70.3	0.0106
3	348.1522	30255	159.5	87375	15.9	0.0115
4	369.3237	32085	101.8	55757	10.2	0.0115
5	387.3337	39427	999.9	547816	100.0	0.0098
6	388.3370	33852	238.4	130638	23.8	0.0115
7	404.3596	33697	227.6	124721	22.8	0.0120
8	409.3148	35469	257.8	141226	25.8	0.0115
9	419.3013	36273	181.8	99594	18.2	0.0116
10	747.6411	30233	80.5	44095	8.0	0.0247

#	m/z	Res.	S/N	I	I %	FWHM
1	57.0676	17729	146.2	62751	14.6	0.0032
2	71.0828	19462	178.2	76497	17.8	0.0037
3	85.0981	20015	115.0	49382	11.5	0.0043
4	95.0823	20908	75.3	32338	7.5	0.0045
5	181.1525	27871	125.3	53774	12.5	0.0065
6	265.2436	30211	90.6	38886	9.1	0.0088
7	295.2895	31921	76.8	32957	7.7	0.0093
8	369.3235	34843	203.6	87411	20.4	0.0106
9	409.3148	39495	999.7	429150	100.0	0.0104
10	410.3182	34432	235.1	100912	23.5	0.0119

Compound Spectrum List Report

Cmpd 136, Dissect, 15.0 min

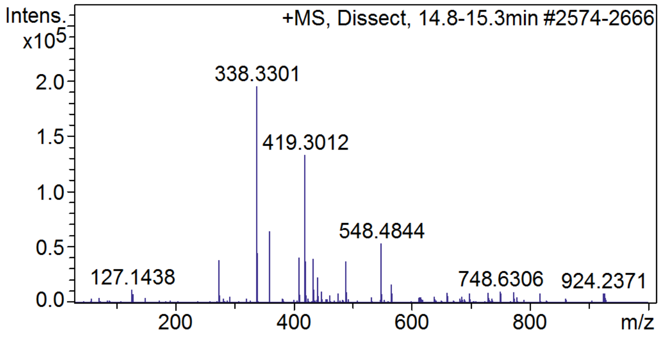

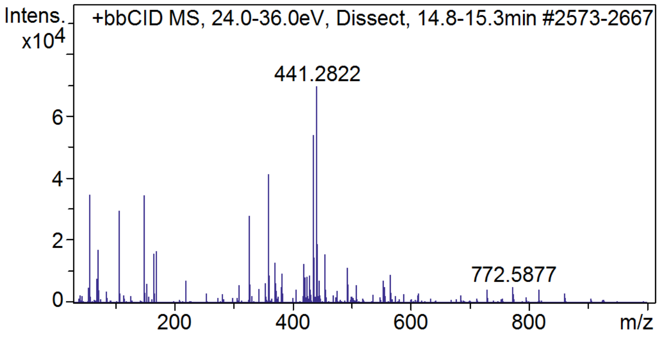

#	m/z	Res.	S/N	I	I %	FWHM
1	275.1549	31685	198.1	38675	19.8	0.0087
2	338.3301	39259	999.9	195204	100.0	0.0086
3	339.3337	36148	231.5	45198	23.2	0.0094
4	360.3110	33663	331.9	64798	33.2	0.0107
5	409.3147	34804	210.0	40998	21.0	0.0118
6	419.3012	35636	681.3	132994	68.1	0.0118
7	420.3044	31658	191.5	37382	19.2	0.0133
8	433.3161	33458	205.3	40072	20.5	0.0130
9	488.3774	30666	192.9	37663	19.3	0.0159
10	548.4844	33304	274.4	53576	27.4	0.0165

#	m/z	Res.	S/N	I	I %	FWHM
1	57.0676	17530	498.7	34725	49.9	0.0033
2	71.0828	19179	245.0	17056	24.5	0.0037
3	107.0455	22150	424.8	29575	42.5	0.0048
4	149.0183	26182	495.5	34497	49.6	0.0057
5	327.2420	31196	322.2	22430	32.2	0.0105
6	327.3260	31396	401.9	27984	40.2	0.0104
7	360.3114	35876	594.9	41419	59.5	0.0100
8	435.3793	33813	772.1	53755	77.2	0.0129
9	441.2822	33152	999.7	69604	100.0	0.0133
10	442.2854	31654	273.6	19052	27.4	0.0140

Cmpd 137, Dissect, 15.0 min

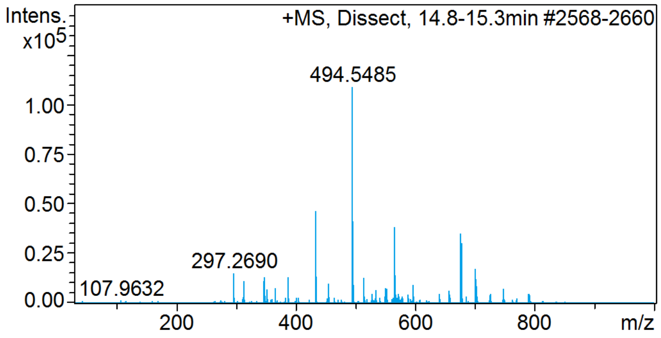

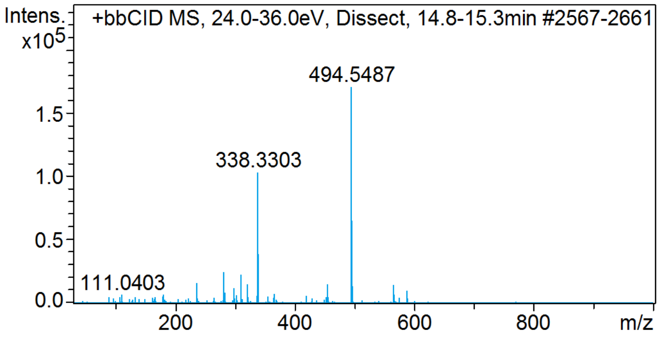

#	m/z	Res.	S/N	I	I %	FWHM
1	433.3162	33855	425.8	46417	42.6	0.0128
2	494.5485	33941	999.8	108982	100.0	0.0146
3	495.5518	32166	380.3	41450	38.0	0.0154
4	565.5466	34284	353.4	38521	35.3	0.0165
5	675.4462	33701	323.1	35214	32.3	0.0200
6	675.6518	35671	314.6	34287	31.5	0.0189
7	676.5049	36442	248.6	27101	24.9	0.0186
8	676.5809	36520	215.9	23538	21.6	0.0185
9	676.6524	34194	185.1	20173	18.5	0.0198
10	677.4532	36289	279.8	30498	28.0	0.0187

#	m/z	Res.	S/N	I	I %	FWHM
1	237.0714	29581	96.9	16566	9.7	0.0080
2	282.2696	33040	146.8	25104	14.7	0.0085
3	310.3000	33400	134.5	22996	13.5	0.0093
4	321.3043	31078	89.7	15339	9.0	0.0103
5	338.3303	32991	604.2	103316	60.5	0.0103
6	339.2777	31745	228.9	39134	22.9	0.0107
7	339.3337	31267	149.1	25492	14.9	0.0109
8	455.2973	32674	89.1	15240	8.9	0.0139
9	494.5487	35880	999.0	170821	100.0	0.0138
10	495.5519	33273	383.1	65503	38.3	0.0149

Compound Spectrum List Report

Cmpd 138, Dissect, 15.2 min

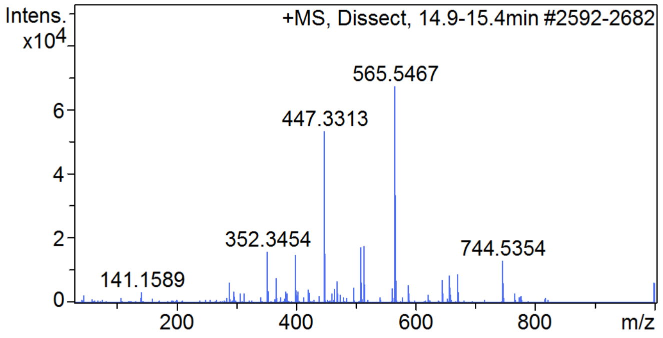

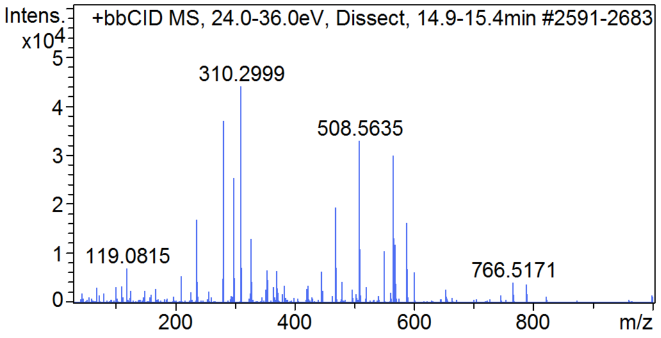

#	m/z	Res.	S/N	I	I %	FWHM
1	352.3454	31081	237.3	15976	23.7	0.0113
2	399.3334	30283	221.2	14890	22.1	0.0132
3	447.3313	34133	790.4	53211	79.1	0.0131
4	448.3346	32207	230.0	15482	23.0	0.0139
5	508.5633	31340	258.7	17420	25.9	0.0162
6	514.2626	31246	262.7	17685	26.3	0.0165
7	565.5467	34059	999.8	67310	100.0	0.0166
8	566.5499	32013	497.2	33477	49.7	0.0177
9	670.4438	29302	133.5	8991	13.4	0.0229
10	744.5354	28996	195.9	13186	19.6	0.0257

#	m/z	Res.	S/N	I	I %	FWHM
1	237.0714	29753	384.5	16947	38.5	0.0080
2	282.2695	32762	839.2	36990	83.9	0.0086
3	299.2956	32335	576.4	25406	57.7	0.0093
4	310.2999	33274	999.7	44064	100.0	0.0093
5	327.3260	32516	295.5	13026	29.6	0.0101
6	469.3125	31581	440.4	19408	44.0	0.0149
7	508.5635	31942	745.4	32851	74.6	0.0159
8	565.5467	33153	679.2	29937	67.9	0.0171
9	566.5500	31700	300.4	13241	30.1	0.0179
10	587.5283	30579	369.2	16273	36.9	0.0192

Cmpd 139, Dissect, 15.2 min

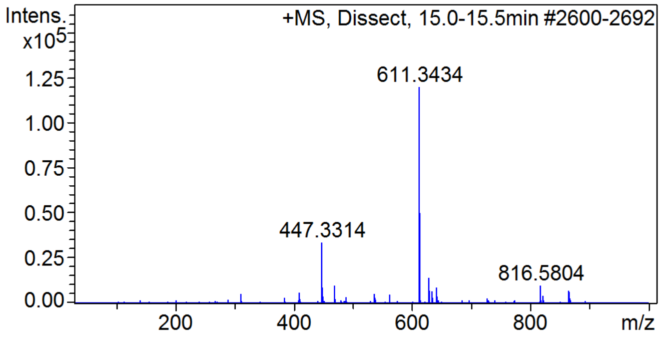

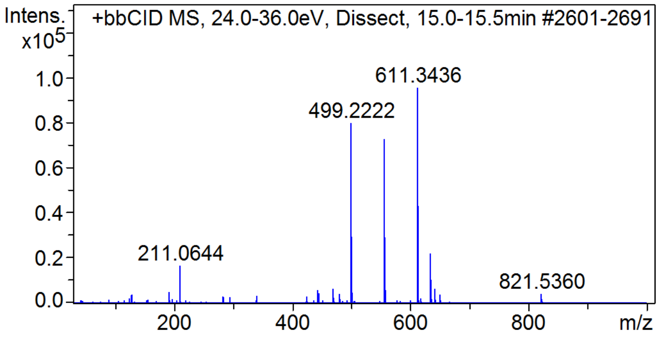

#	m/z	Res.	S/N	I	I %	FWHM
1	447.3314	34574	281.7	33744	28.2	0.0129
2	448.3347	32447	76.6	9181	7.7	0.0138
3	469.3124	31854	84.2	10081	8.4	0.0147
4	611.3434	34848	999.6	119732	100.0	0.0175
5	612.3468	31822	418.9	50180	41.9	0.0192
6	613.3504	30530	107.9	12921	10.8	0.0201
7	628.3693	29160	119.4	14307	11.9	0.0215
8	640.5647	31368	75.5	9045	7.6	0.0204
9	816.5804	29284	83.1	9954	8.3	0.0279
10	864.2365	28985	62.1	7433	6.2	0.0298

#	m/z	Res.	S/N	I	I %	FWHM
1	211.0644	28265	175.9	16807	17.6	0.0075
2	499.2222	33646	833.1	79617	83.3	0.0148
3	500.2254	32473	309.4	29570	30.9	0.0154
4	555.2829	32428	761.6	72783	76.2	0.0171
5	556.2861	31873	306.4	29282	30.6	0.0175
6	611.3436	34028	999.8	95549	100.0	0.0180
7	612.3467	32579	453.2	43311	45.3	0.0188
8	613.3498	30507	105.3	10063	10.5	0.0201
9	633.3242	31203	232.3	22205	23.2	0.0203
10	634.3282	31124	112.1	10708	11.2	0.0204
