# Supplementary material for: Assessment of Xenoestrogens in Jordanian Water System: Activity and Identification
Source: Toxics. 2023 Jan 9;11(1):63. doi: 10.3390/toxics11010063 (PMC9866086; doi:10.3390/toxics11010063)
Supplement: Supplementary file 1 [file toxics-11-00063-s001.zip › table S2.pdf]

Compound Spectrum List Report

Analysis Info 24_August_2021_Dr_ Yazan alakam_818

Acquisition Date 24/08/2021

Method Kailani _MS/MS

Operator Demo User
Instrument impact II 1825265.10265

Acquisition Parameter

Source Type	ESI	Ion Polarity	Positive	Set Nebulizer	2.0 Bar
Focus	Active	Set Capillary	2500 V	Set Dry Heater	200 °C
Scan Begin	30 m/z	Set End Plate Offset	-500 V	Set Dry Gas	8.0 l/min
Scan End	1000 m/z	Set Charging Voltage	2000 V		

Cmpd 1, Dissect, 1.3 min

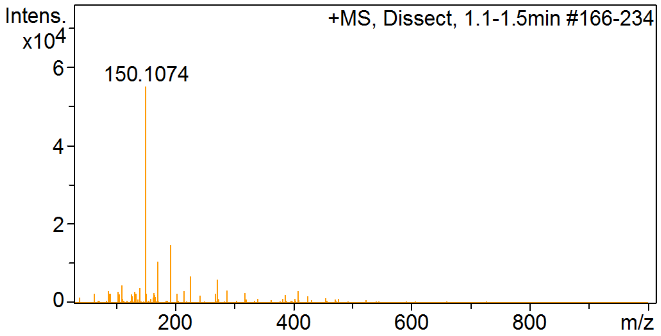

#	m/z	Res.	S/N	I	I %	FWHM
1	88.0730	19475	56.0	3111	5.7	0.0045
2	110.0050	23135	84.7	4704	8.5	0.0048
3	141.1088	25035	72.6	4032	7.3	0.0056
4	150.1074	26797	990.3	55026	100.0	0.0056
5	172.0888	27659	189.8	10545	19.2	0.0062
6	194.1322	29162	267.4	14855	27.0	0.0067
7	216.1132	29457	56.8	3157	5.7	0.0073
8	226.9440	29782	122.8	6824	12.4	0.0076
9	272.9354	31539	110.4	6132	11.1	0.0087
10	288.9105	26880	59.2	3291	6.0	0.0107

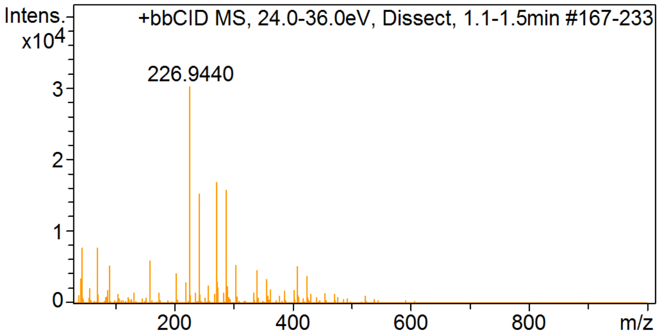

#	m/z	Res.	S/N	I	I %	FWHM
1	45.0315	15870	258.7	7815	25.9	0.0028
2	70.0625	18033	257.5	7779	25.7	0.0039
3	90.9738	20076	176.3	5327	17.6	0.0045
4	158.9585	27357	200.0	6042	20.0	0.0058
5	226.9440	31109	999.9	30210	100.0	0.0073
6	242.9174	31068	506.1	15290	50.6	0.0078
7	272.9353	31624	558.2	16865	55.8	0.0086
8	288.9100	29401	522.2	15778	52.2	0.0098
9	304.8833	28283	179.4	5421	17.9	0.0108
10	408.9047	29592	172.1	5199	17.2	0.0138

Compound Spectrum List Report

Cmpd 2, Dissect, 1.4 min

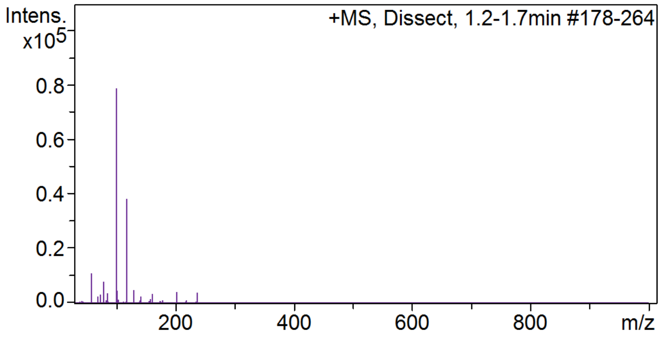

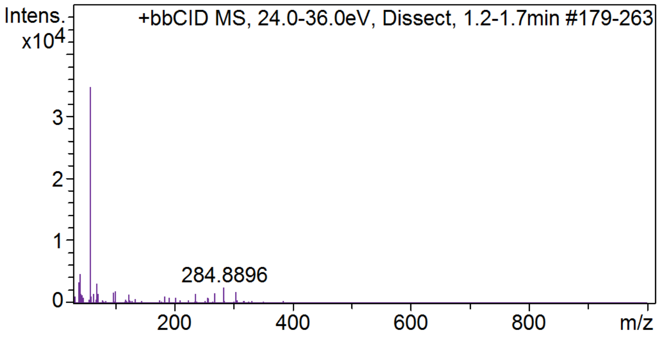

#	m/z	Res.	S/N	I	I %	FWHM
1	59.0470	17209	141.3	11219	14.2	0.0034
2	79.0185	18846	100.9	8011	10.2	0.0042
3	86.0571	18790	48.9	3880	4.9	0.0046
4	101.0565	21385	992.1	78741	100.0	0.0047
5	102.0598	20497	61.3	4864	6.2	0.0050
6	118.0824	23203	480.4	38127	48.4	0.0051
7	130.1045	23657	62.0	4923	6.3	0.0055
8	162.1436	26315	44.8	3556	4.5	0.0062
9	203.0460	29448	53.6	4251	5.4	0.0069
10	238.1575	29142	51.7	4102	5.2	0.0082

#	m/z	Res.	S/N	I	I %	FWHM
1	38.9615	14728	98.0	3414	9.8	0.0026
2	41.0369	15275	138.2	4813	13.8	0.0027
3	59.0106	17230	76.8	2676	7.7	0.0034
4	59.0470	17096	998.0	34754	100.0	0.0035
5	69.0311	17007	91.6	3191	9.2	0.0041
6	97.0046	19324	51.6	1798	5.2	0.0050
7	101.0565	20704	57.9	2015	5.8	0.0049
8	268.9161	32671	48.1	1674	4.8	0.0082
9	284.8896	30705	76.0	2645	7.6	0.0093
10	304.8832	28167	55.5	1933	5.6	0.0108

Cmpd 3, Dissect, 1.4 min

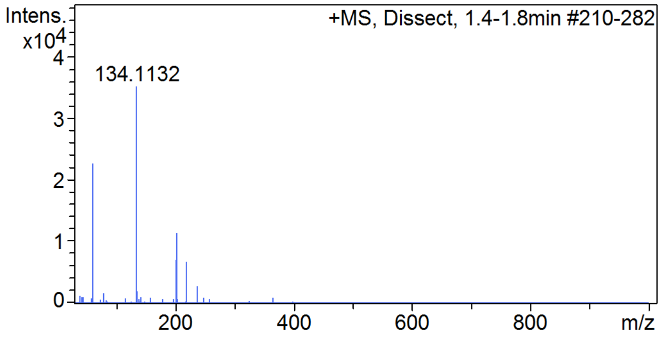

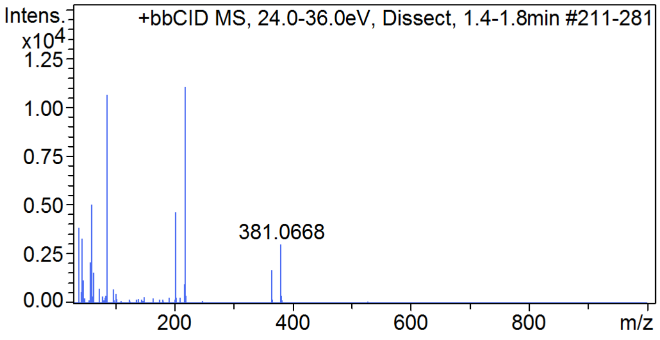

#	m/z	Res.	S/N	I	I %	FWHM
1	61.0083	16961	48.2	1702	4.8	0.0036
2	61.0374	17040	643.2	22687	64.5	0.0036
3	79.0185	18890	48.1	1698	4.8	0.0042
4	134.1132	25038	996.9	35162	100.0	0.0054
5	135.1165	23415	56.6	1995	5.7	0.0058
6	202.0798	29417	67.0	2362	6.7	0.0069
7	202.1734	29324	201.2	7098	20.2	0.0069
8	203.0459	29459	325.7	11488	32.7	0.0069
9	219.0192	28987	191.5	6754	19.2	0.0076
10	238.1576	30232	81.3	2867	8.2	0.0079

#	m/z	Res.	S/N	I	I %	FWHM
1	38.9615	14845	348.8	3853	34.9	0.0026
2	44.0113	15065	299.3	3305	30.0	0.0029
3	58.0630	17028	189.5	2093	19.0	0.0034
4	61.0374	16746	454.9	5024	45.5	0.0036
5	63.9956	16682	144.3	1593	14.4	0.0038
6	86.0936	18852	963.6	10643	96.5	0.0046
7	203.0461	29308	420.0	4638	42.0	0.0069
8	219.0195	29768	999.0	11034	100.0	0.0074
9	365.0935	32023	154.3	1704	15.4	0.0114
10	381.0668	32101	273.3	3018	27.4	0.0119

Compound Spectrum List Report

Cmpd 4, Dissect, 1.5 min

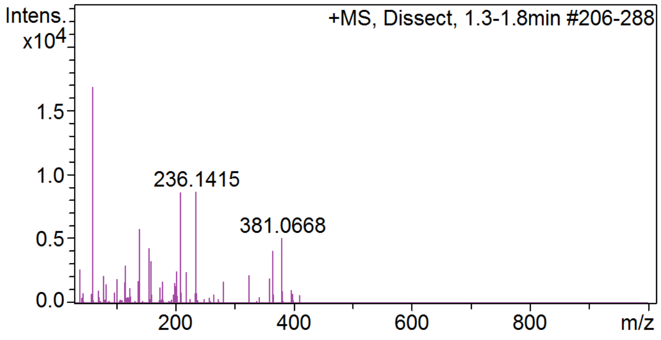

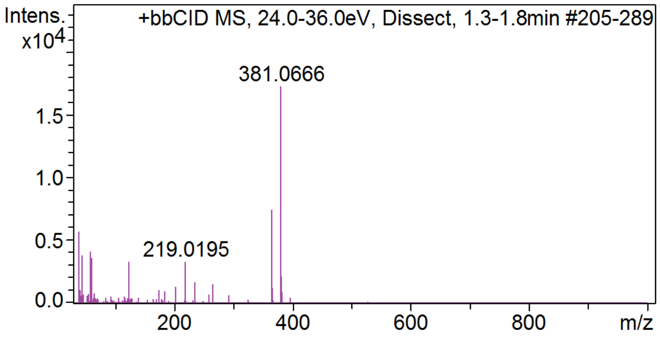

#	m/z	Res.	S/N	I	I %	FWHM
1	38.9615	14617	159.1	2686	15.9	0.0027
2	61.0374	16790	998.9	16868	100.0	0.0036
3	116.0669	23418	175.3	2961	17.6	0.0050
4	139.0456	25562	342.7	5787	34.3	0.0054
5	156.1330	25860	253.6	4282	25.4	0.0060
6	159.0575	26843	196.2	3314	19.6	0.0059
7	210.1057	30150	511.0	8629	51.2	0.0070
8	236.1415	31051	513.2	8666	51.4	0.0076
9	365.0934	31845	244.3	4126	24.5	0.0115
10	381.0668	32259	302.7	5111	30.3	0.0118

#	m/z	Res.	S/N	I	I %	FWHM
1	38.9615	14763	329.5	5704	33.1	0.0026
2	44.0113	15404	220.6	3818	22.1	0.0029
3	58.0630	17162	239.8	4152	24.1	0.0034
4	61.0375	16493	211.1	3655	21.2	0.0037
5	122.9204	23229	167.0	2891	16.8	0.0053
6	123.0401	23138	191.6	3317	19.2	0.0053
7	219.0195	29177	192.4	3331	19.3	0.0075
8	365.0933	31164	430.2	7447	43.2	0.0117
9	381.0666	31909	997.0	17258	100.0	0.0119
10	382.0701	32701	124.7	2159	12.5	0.0117

Cmpd 5, Dissect, 1.7 min

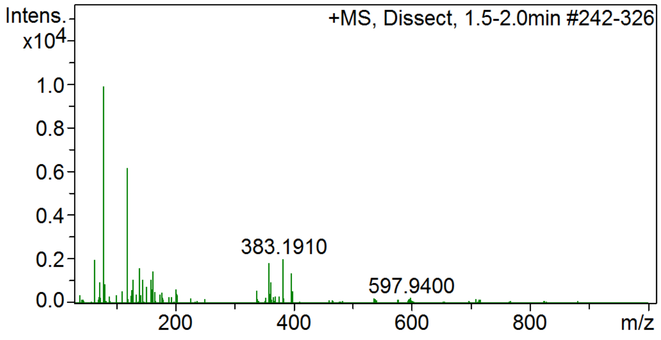

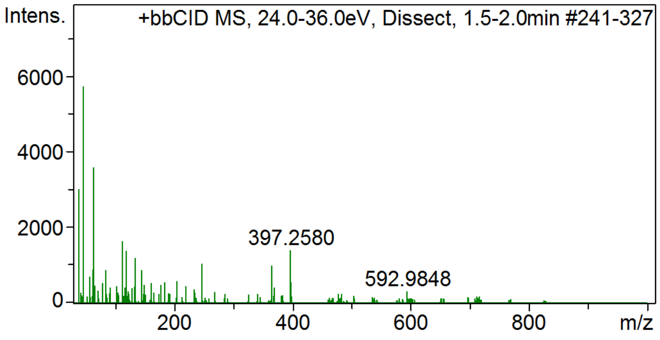

#	m/z	Res.	S/N	I	I %	FWHM
1	63.9956	16687	201.7	1998	20.2	0.0038
2	79.0185	18907	999.1	9900	100.0	0.0042
3	119.0565	23672	624.3	6186	62.5	0.0050
4	129.0482	23999	111.1	1101	11.1	0.0054
5	140.0884	25151	163.6	1621	16.4	0.0056
6	145.0599	25944	111.1	1101	11.1	0.0056
7	163.1174	27341	149.6	1482	15.0	0.0060
8	359.3032	31918	188.4	1867	18.9	0.0113
9	383.1910	32600	203.7	2018	20.4	0.0118
10	397.2581	31551	140.8	1396	14.1	0.0126

#	m/z	Res.	S/N	I	I %	FWHM
1	38.9615	14721	524.7	3007	52.5	0.0026
2	46.9930	16011	999.7	5729	100.0	0.0029
3	62.9877	16946	157.3	901	15.7	0.0037
4	63.9956	16462	625.0	3582	62.5	0.0039
5	112.8919	23196	286.2	1640	28.6	0.0049
6	119.0564	23571	243.4	1395	24.3	0.0051
7	134.0402	26445	210.2	1204	21.0	0.0051
8	248.0136	32277	184.0	1055	18.4	0.0077
9	365.0933	30646	176.5	1012	17.7	0.0119
10	397.2580	30656	244.7	1402	24.5	0.0130

Compound Spectrum List Report

Cmpd 6, Dissect, 1.8 min

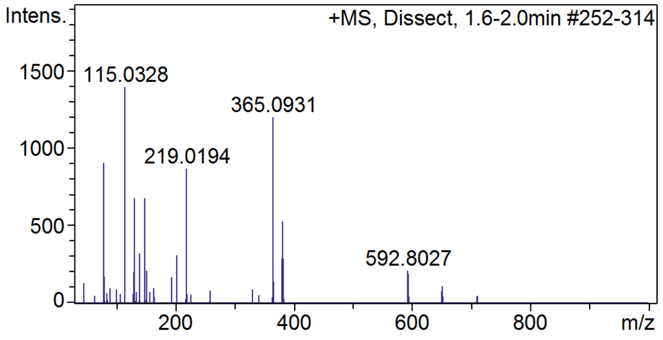

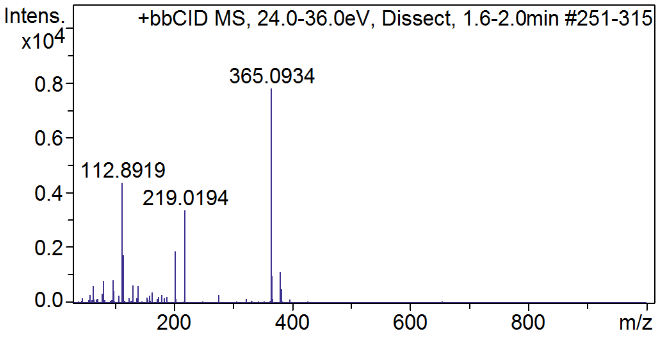

#	m/z	Res.	S/N	I	I %	FWHM
1	79.0185	19018	647.1	905	64.9	0.0042
2	115.0328	22750	997.7	1395	100.0	0.0051
3	131.0060	24151	483.8	676	48.5	0.0054
4	139.0456	25133	230.8	323	23.1	0.0055
5	148.0556	24892	485.2	678	48.6	0.0059
6	203.0460	29511	223.5	312	22.4	0.0069
7	219.0194	29754	620.3	867	62.2	0.0074
8	365.0931	32086	856.9	1198	85.9	0.0114
9	381.2842	32226	379.9	531	38.1	0.0118
10	383.1913	29901	208.5	292	20.9	0.0128

#	m/z	Res.	S/N	I	I %	FWHM
1	80.9451	19320	106.7	832	10.7	0.0042
2	96.9188	19686	109.1	851	10.9	0.0049
3	112.8919	23274	558.5	4358	55.9	0.0049
4	114.8892	22423	225.7	1761	22.6	0.0051
5	115.0325	24047	155.1	1210	15.5	0.0048
6	203.0461	29925	242.9	1895	24.3	0.0068
7	219.0194	29942	432.8	3378	43.3	0.0073
8	365.0934	30714	1000.0	7803	100.0	0.0119
9	366.0968	33273	128.0	999	12.8	0.0110
10	381.0667	29624	147.5	1151	14.8	0.0129

Cmpd 7, Dissect, 1.9 min

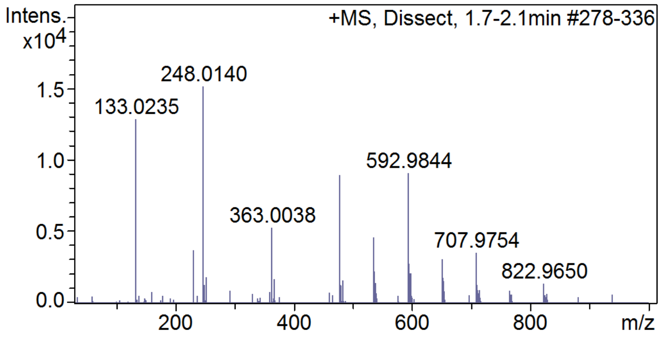

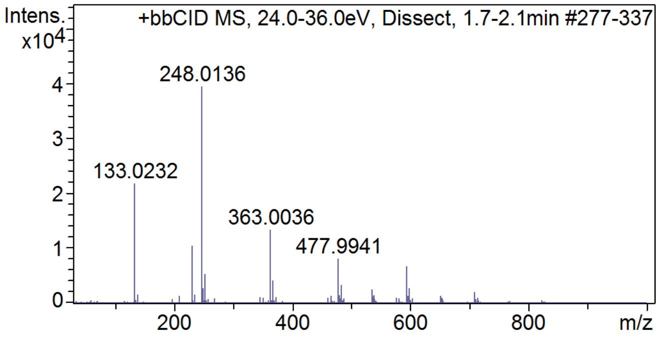

#	m/z	Res.	S/N	I	I %	FWHM
1	133.0235	25398	847.1	12853	84.9	0.0052
2	230.9877	31314	244.6	3711	24.5	0.0074
3	248.0140	30940	998.0	15142	100.0	0.0080
4	363.0038	33010	348.4	5287	34.9	0.0110
5	477.9941	34689	589.2	8940	59.0	0.0138
6	535.4891	37661	304.8	4624	30.5	0.0142
7	592.9844	33449	598.7	9083	60.0	0.0177
8	593.9852	38676	182.9	2775	18.3	0.0154
9	650.4801	34002	205.2	3114	20.6	0.0191
10	707.9754	30952	234.2	3553	23.5	0.0229

#	m/z	Res.	S/N	I	I %	FWHM
1	133.0232	25298	551.6	21831	55.3	0.0053
2	230.9875	30017	265.4	10505	26.6	0.0077
3	248.0136	32343	996.8	39451	100.0	0.0077
4	252.9688	30634	136.1	5385	13.6	0.0083
5	363.0036	33344	340.3	13467	34.1	0.0109
6	367.9588	30290	108.6	4297	10.9	0.0121
7	477.9941	34141	207.0	8194	20.8	0.0140
8	482.9490	30628	86.1	3406	8.6	0.0158
9	592.9850	36090	173.2	6854	17.4	0.0164
10	597.9397	31175	71.6	2836	7.2	0.0192

Compound Spectrum List Report

Cmpd 8, Dissect, 2.0 min

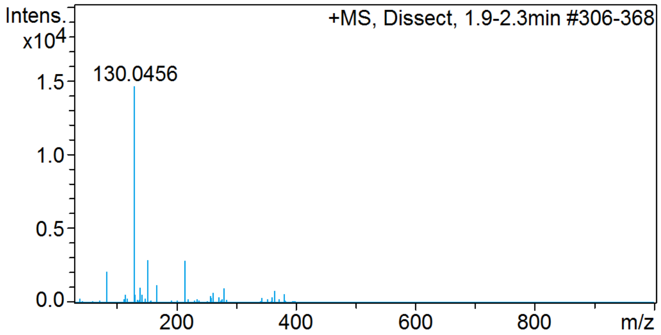

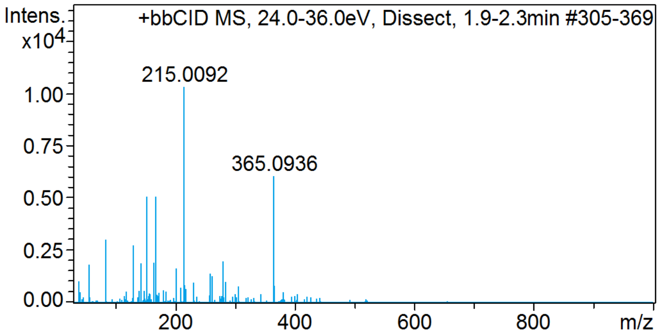

#	m/z	Res.	S/N	I	I %	FWHM
1	84.0417	18294	146.5	2146	14.7	0.0046
2	130.0456	25156	999.0	14632	100.0	0.0052
3	139.0454	24866	72.2	1057	7.2	0.0056
4	152.0267	26024	200.0	2930	20.0	0.0058
5	168.0004	26807	82.5	1209	8.3	0.0063
6	215.0094	30210	195.4	2862	19.6	0.0071
7	262.8794	31229	48.0	702	4.8	0.0084
8	281.0652	29018	68.2	999	6.8	0.0097
9	365.0932	30708	57.9	848	5.8	0.0119
10	381.2846	28370	39.8	583	4.0	0.0134

#	m/z	Res.	S/N	I	I %	FWHM
1	56.0474	17285	178.1	1837	17.8	0.0032
2	84.0417	19004	294.7	3039	29.5	0.0044
3	130.0454	24986	267.3	2757	26.8	0.0052
4	142.9336	25998	184.3	1901	18.4	0.0055
5	152.0266	26783	491.5	5070	49.2	0.0057
6	164.9152	27135	188.8	1947	18.9	0.0061
7	168.0002	26785	492.1	5075	49.2	0.0063
8	215.0092	29411	999.2	10306	100.0	0.0073
9	281.0651	33013	193.8	1999	19.4	0.0085
10	365.0936	30758	585.4	6038	58.6	0.0119

Cmpd 9, Dissect, 2.2 min

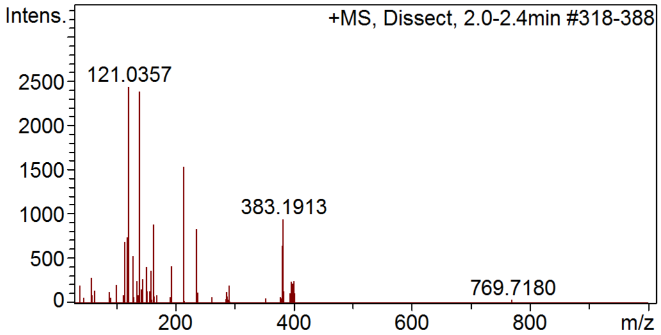

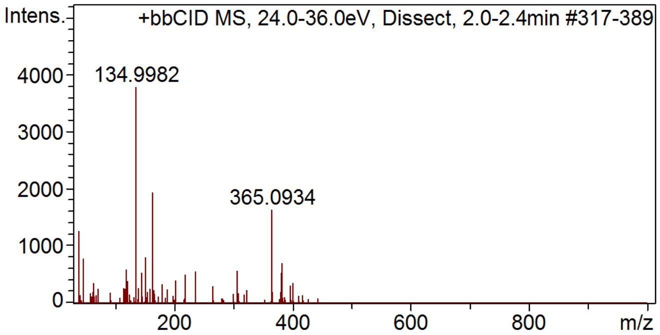

#	m/z	Res.	S/N	I	I %	FWHM
1	115.0329	23223	282.9	691	28.4	0.0050
2	119.0565	23684	306.2	748	30.7	0.0050
3	121.0357	23964	997.7	2436	100.0	0.0051
4	129.0481	23841	220.5	538	22.1	0.0054
5	139.0454	25538	975.9	2383	97.8	0.0054
6	164.9151	27637	364.9	891	36.6	0.0060
7	215.0092	29913	629.9	1538	63.1	0.0072
8	236.9904	30614	344.3	841	34.5	0.0077
9	381.2845	31874	267.2	652	26.8	0.0120
10	383.1913	29907	388.2	948	38.9	0.0128

#	m/z	Res.	S/N	I	I %	FWHM
1	38.9615	14901	335.7	1272	33.6	0.0026
2	46.9931	15952	208.4	790	20.9	0.0029
3	119.0565	23326	157.4	597	15.7	0.0051
4	134.9982	25726	999.6	3788	100.0	0.0052
5	150.9718	25258	214.4	812	21.4	0.0060
6	164.9150	27372	510.9	1936	51.1	0.0060
7	236.9908	29569	150.2	569	15.0	0.0080
8	306.8416	32628	151.0	572	15.1	0.0094
9	365.0934	31841	432.1	1637	43.2	0.0115
10	383.1909	33409	188.3	713	18.8	0.0115

Compound Spectrum List Report

Cmpd 10, Dissect, 2.5 min

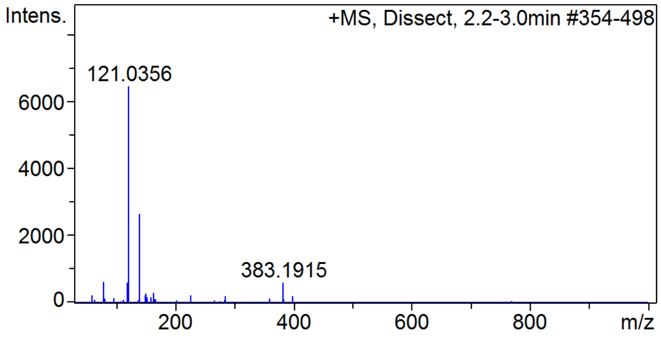

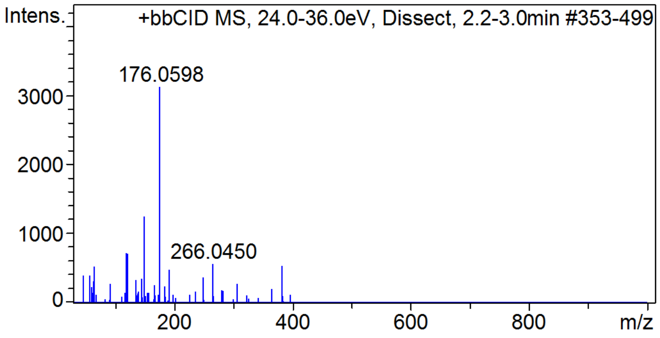

#	m/z	Res.	S/N	I	I %	FWHM
1	79.0185	18861	98.2	642	10.0	0.0042
2	119.0564	23762	95.4	623	9.7	0.0050
3	121.0356	23897	987.2	6450	100.0	0.0051
4	121.0612	21624	40.3	263	4.1	0.0056
5	139.0456	25138	405.2	2647	41.0	0.0055
6	140.0884	25340	86.9	568	8.8	0.0055
7	149.0138	24836	34.5	226	3.5	0.0060
8	150.0863	26546	40.8	267	4.1	0.0057
9	164.9150	27506	48.4	316	4.9	0.0060
10	383.1915	31335	95.6	625	9.7	0.0122

#	m/z	Res.	S/N	I	I %	FWHM
1	46.9931	15877	129.2	404	12.9	0.0030
2	57.0677	16910	128.2	401	12.8	0.0034
3	65.0364	17126	170.3	532	17.0	0.0038
4	119.0564	23574	231.5	724	23.2	0.0051
5	121.0357	23565	227.3	711	22.7	0.0051
6	149.0135	26209	401.0	1254	40.1	0.0057
7	176.0598	27804	999.7	3126	100.0	0.0063
8	192.0332	29032	155.2	485	15.5	0.0066
9	266.0450	33581	182.3	570	18.2	0.0079
10	383.1914	31380	171.8	537	17.2	0.0122

Cmpd 11, Dissect, 2.7 min

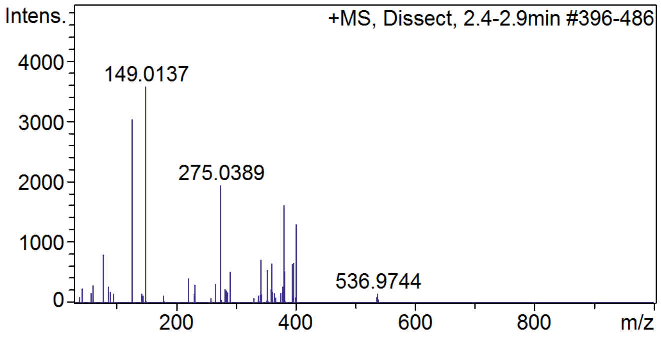

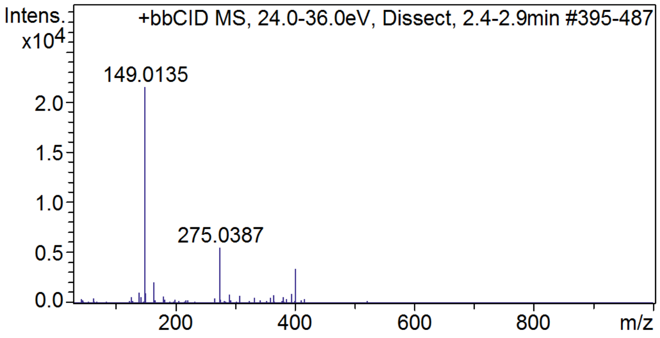

#	m/z	Res.	S/N	I	I %	FWHM
1	79.0185	18910	226.1	808	22.6	0.0042
2	127.0324	23896	846.5	3026	84.7	0.0053
3	149.0137	24750	999.2	3572	100.0	0.0060
4	275.0389	30849	544.0	1945	54.4	0.0089
5	343.0237	33805	200.5	717	20.1	0.0101
6	361.2101	32162	182.8	654	18.3	0.0112
7	381.2846	32177	451.1	1612	45.1	0.0118
8	395.0465	31364	181.4	648	18.2	0.0126
9	397.2580	30500	185.7	664	18.6	0.0130
10	401.0634	34342	364.3	1302	36.5	0.0117

#	m/z	Res.	S/N	I	I %	FWHM
1	139.9099	25674	51.6	1112	5.2	0.0054
2	149.0135	26457	998.0	21529	100.0	0.0056
3	150.0169	25399	48.4	1044	4.8	0.0059
4	164.9148	27526	54.4	1173	5.4	0.0060
5	164.9869	27566	98.3	2120	9.8	0.0060
6	275.0387	31488	258.6	5579	25.9	0.0087
7	291.0099	26139	40.5	874	4.1	0.0111
8	365.0931	32161	37.8	815	3.8	0.0114
9	395.0466	33073	45.0	971	4.5	0.0119
10	401.0636	30104	159.7	3444	16.0	0.0133

Compound Spectrum List Report

Cmpd 12, Dissect, 2.9 min

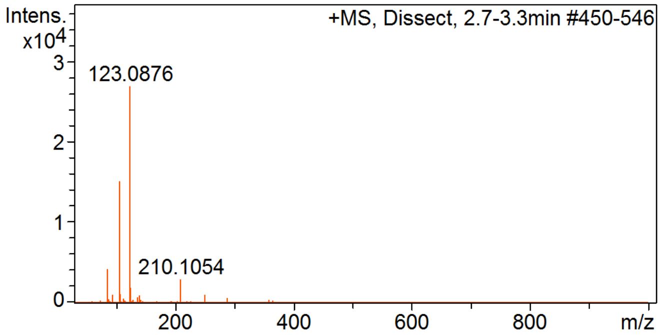

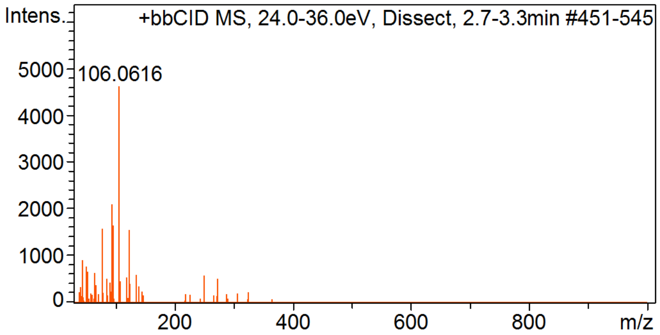

#	m/z	Res.	S/N	I	I %	FWHM
1	86.0571	19002	157.1	4237	15.7	0.0045
2	94.0620	19088	35.9	969	3.6	0.0049
3	106.0616	22386	560.4	15118	56.1	0.0047
4	107.0657	20096	39.6	1068	4.0	0.0053
5	123.0876	23451	998.2	26929	100.0	0.0052
6	124.0908	22424	71.2	1920	7.1	0.0055
7	136.0569	24676	25.8	696	2.6	0.0055
8	140.0883	25309	34.6	934	3.5	0.0055
9	210.1054	29905	111.8	3016	11.2	0.0070
10	251.0564	30949	37.9	1021	3.8	0.0081

#	m/z	Res.	S/N	I	I %	FWHM
1	44.0113	15499	198.3	916	19.9	0.0028
2	44.0476	15368	154.7	715	15.5	0.0029
3	51.0210	16601	170.2	786	17.0	0.0031
4	53.0365	17057	146.6	677	14.7	0.0031
5	65.0364	17056	140.8	651	14.1	0.0038
6	78.0311	18767	343.3	1586	34.4	0.0042
7	94.0622	19765	454.7	2101	45.5	0.0048
8	96.0415	19939	357.5	1652	35.8	0.0048
9	106.0616	21847	998.7	4614	100.0	0.0049
10	123.0401	24379	336.9	1556	33.7	0.0050

Cmpd 13, Dissect, 3.2 min

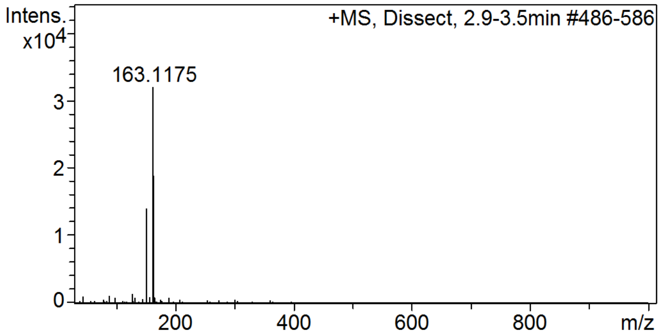

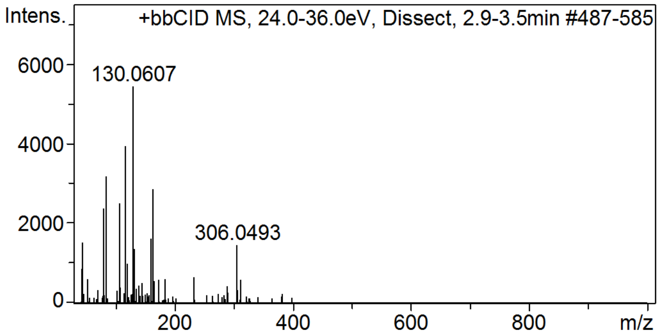

#	m/z	Res.	S/N	I	I %	FWHM
1	45.0316	15850	32.3	1037	3.2	0.0028
2	89.0568	20035	33.3	1069	3.3	0.0044
3	128.1392	23736	44.9	1443	4.5	0.0054
4	151.0913	26170	437.4	14052	43.8	0.0058
5	157.0785	26104	27.0	867	2.7	0.0060
6	163.1175	27695	998.1	32065	100.0	0.0059
7	164.1014	27688	587.4	18873	58.9	0.0059
8	164.1207	26238	123.9	3982	12.4	0.0063
9	164.9150	26361	30.9	993	3.1	0.0063
10	165.1046	27771	27.7	891	2.8	0.0059

#	m/z	Res.	S/N	I	I %	FWHM
1	45.0316	15593	282.0	1537	28.3	0.0029
2	80.0467	18769	436.7	2381	43.8	0.0043
3	84.0779	19629	582.7	3177	58.4	0.0043
4	107.0693	21914	460.1	2509	46.1	0.0049
5	117.0533	23116	722.6	3940	72.5	0.0051
6	130.0607	25104	997.1	5436	100.0	0.0052
7	161.0131	27195	299.3	1632	30.0	0.0059
8	164.1014	27293	525.7	2866	52.7	0.0060
9	164.9150	27098	297.0	1619	29.8	0.0061
10	306.0493	30889	269.8	1471	27.1	0.0099

Compound Spectrum List Report

Cmpd 14, Dissect, 3.3 min

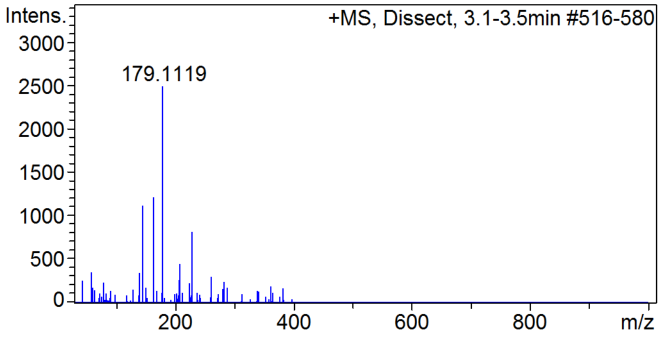

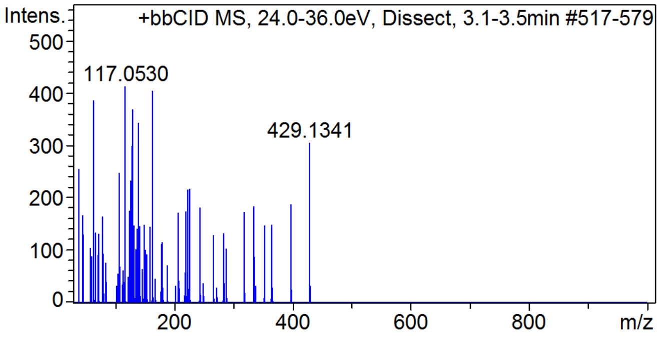

#	m/z	Res.	S/N	I	I %	FWHM
1	43.0159	15908	103.1	257	10.3	0.0027
2	58.0630	17176	141.7	353	14.2	0.0034
3	140.0884	25069	140.5	350	14.1	0.0056
4	145.0599	25888	448.1	1117	44.9	0.0056
5	164.1014	27549	488.0	1216	48.9	0.0060
6	179.1119	27295	998.8	2489	100.0	0.0066
7	207.1421	30082	106.8	266	10.7	0.0069
8	209.0952	29262	180.8	451	18.1	0.0071
9	229.1472	29314	327.0	815	32.7	0.0078
10	261.1722	30567	123.7	308	12.4	0.0085

#	m/z	Res.	S/N	I	I %	FWHM
1	38.9615	14677	616.6	254	61.7	0.0027
2	63.9955	16496	934.8	386	93.5	0.0039
3	107.0694	21175	600.8	248	60.1	0.0051
4	117.0530	23384	999.9	413	100.0	0.0050
5	129.0139	24637	723.7	299	72.4	0.0052
6	130.0608	25458	893.5	369	89.4	0.0051
7	139.9099	25219	828.9	342	82.9	0.0055
8	164.1012	27670	739.6	305	74.0	0.0059
9	164.9150	27508	981.1	405	98.1	0.0060
10	429.1341	39664	739.0	305	73.9	0.0108

Cmpd 15, Dissect, 3.3 min

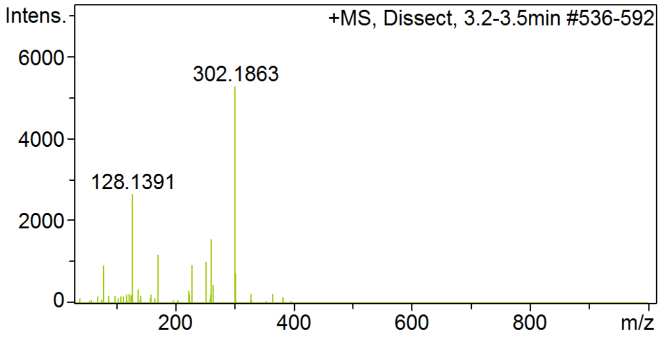

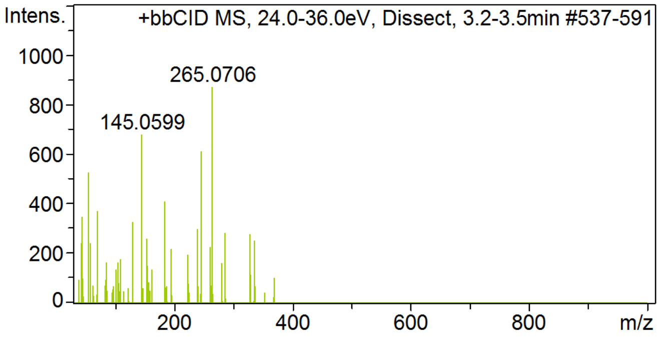

#	m/z	Res.	S/N	I	I %	FWHM
1	79.0185	18998	175.0	923	17.5	0.0042
2	128.1391	23835	503.6	2656	50.4	0.0054
3	172.0909	27495	225.2	1188	22.5	0.0063
4	172.1640	28596	85.7	452	8.6	0.0060
5	229.1472	30294	179.1	944	17.9	0.0076
6	253.1196	29688	191.9	1012	19.2	0.0085
7	261.1724	30131	295.1	1556	29.5	0.0087
8	265.0705	32160	86.2	455	8.6	0.0082
9	302.1863	31804	999.5	5271	100.0	0.0095
10	303.1894	30473	138.9	733	13.9	0.0099

#	m/z	Res.	S/N	I	I %	FWHM
1	44.0476	15644	400.6	349	40.1	0.0028
2	55.0521	17280	602.7	526	60.3	0.0032
3	70.0626	18466	426.5	372	42.7	0.0038
4	130.0608	25576	377.5	329	37.8	0.0051
5	145.0599	25689	777.3	678	77.7	0.0056
6	185.0746	28141	472.2	412	47.2	0.0066
7	240.0552	29772	343.0	299	34.3	0.0081
8	246.0631	29187	700.6	611	70.1	0.0084
9	265.0706	31633	999.9	872	100.0	0.0084
10	265.1069	27962	729.7	636	73.0	0.0095

Compound Spectrum List Report

Cmpd 16, Dissect, 3.4 min

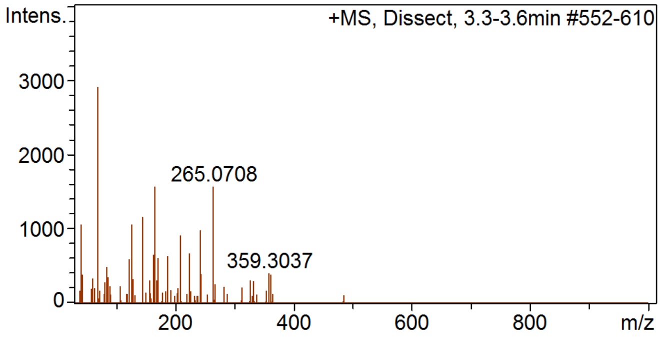

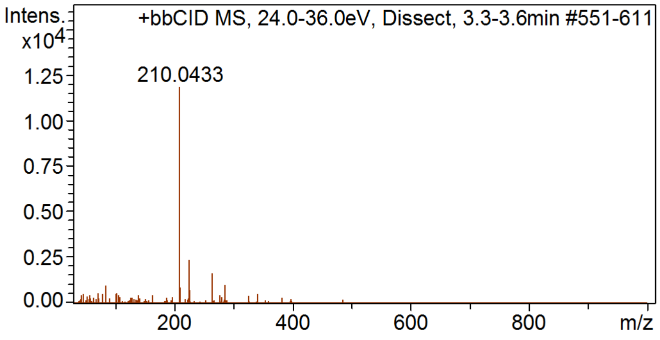

#	m/z	Res.	S/N	I	I %	FWHM
1	41.0368	15240	363.3	1060	36.4	0.0027
2	69.0675	17646	998.9	2914	100.0	0.0039
3	127.0695	22411	363.1	1059	36.3	0.0057
4	145.0600	26388	398.4	1162	39.9	0.0055
5	164.1016	27454	226.6	661	22.7	0.0060
6	166.0805	26531	537.7	1569	53.8	0.0063
7	210.0435	27916	313.1	914	31.3	0.0075
8	225.1024	27280	230.9	674	23.1	0.0083
9	243.1752	32801	338.2	987	33.9	0.0074
10	265.0708	31772	538.9	1572	54.0	0.0083

#	m/z	Res.	S/N	I	I %	FWHM
1	46.9931	16024	47.2	558	4.7	0.0029
2	71.0828	19294	48.5	575	4.9	0.0037
3	84.0780	18925	84.5	1001	8.5	0.0044
4	103.0509	22211	48.2	570	4.8	0.0046
5	210.0433	29543	999.0	11830	100.0	0.0071
6	211.0466	26100	75.6	895	7.6	0.0081
7	226.0166	30903	203.0	2404	20.3	0.0073
8	226.9438	28892	64.9	768	6.5	0.0079
9	265.0706	30286	140.7	1666	14.1	0.0088
10	287.0522	30910	86.2	1020	8.6	0.0093

Cmpd 17, Dissect, 3.6 min

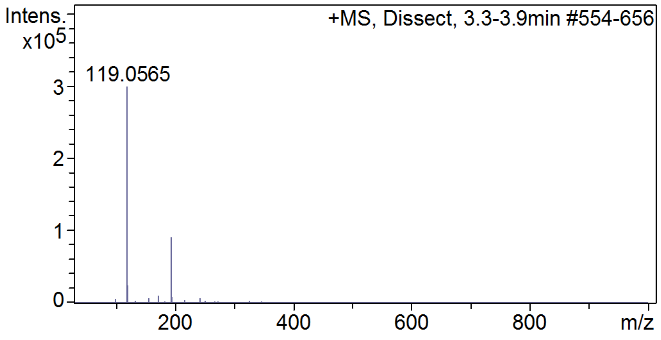

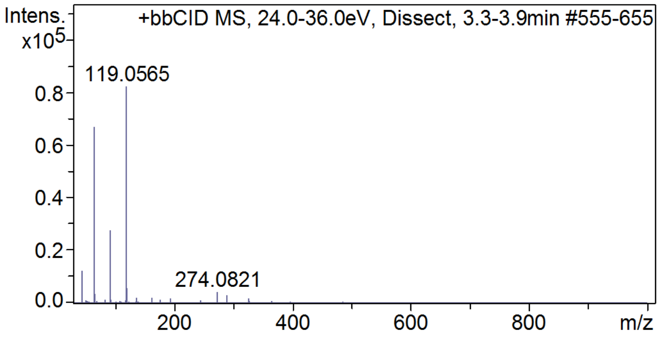

#	m/z	Res.	S/N	I	I %	FWHM
1	100.0725	20812	19.7	6107	2.0	0.0048
2	119.0565	24336	965.8	299107	100.0	0.0049
3	120.0598	22965	79.9	24733	8.3	0.0052
4	156.0967	27217	21.7	6724	2.2	0.0057
5	156.1330	26759	16.0	4956	1.7	0.0058
6	173.1227	28202	33.4	10342	3.5	0.0061
7	195.1163	28764	295.4	91499	30.6	0.0068
8	196.1197	28034	28.1	8718	2.9	0.0070
9	217.0977	29187	11.6	3608	1.2	0.0074
10	243.1752	31902	22.1	6842	2.3	0.0076

#	m/z	Res.	S/N	I	I %	FWHM
1	45.0316	15946	151.0	12524	15.2	0.0028
2	65.0364	17422	804.7	66763	81.2	0.0037
3	66.0397	16979	46.7	3871	4.7	0.0039
4	92.0466	19524	333.3	27652	33.6	0.0047
5	119.0565	23466	990.9	82206	100.0	0.0051
6	120.0598	22758	71.0	5891	7.2	0.0053
7	136.0573	25802	25.3	2095	2.5	0.0053
8	163.0288	26799	24.6	2040	2.5	0.0061
9	274.0821	31132	53.7	4457	5.4	0.0088
10	290.0770	30341	36.7	3042	3.7	0.0096

Compound Spectrum List Report

Cmpd 18, Dissect, 3.7 min

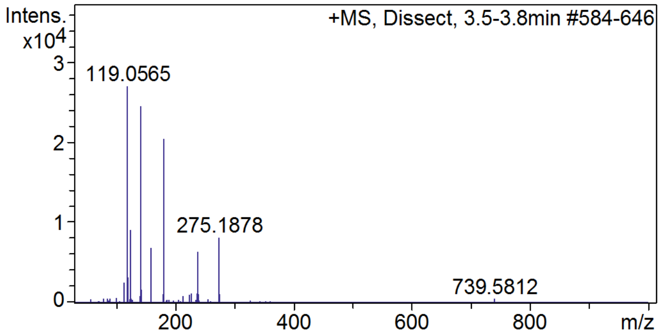

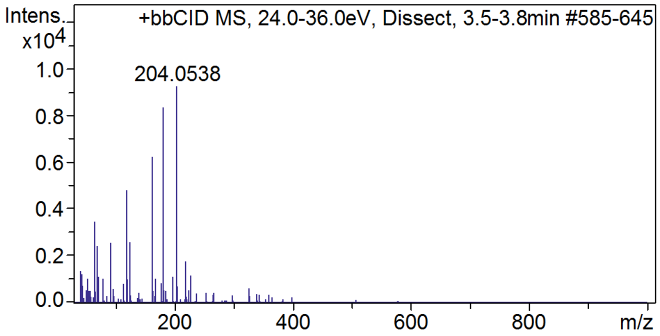

#	m/z	Res.	S/N	I	I %	FWHM
1	114.0875	22381	96.1	2597	9.6	0.0051
2	119.0565	24296	998.5	26992	100.0	0.0049
3	120.0598	22897	118.1	3191	11.8	0.0052
4	125.0557	23369	335.9	9079	33.6	0.0054
5	142.0815	25629	906.3	24498	90.8	0.0055
6	142.1178	25357	118.4	3201	11.9	0.0056
7	160.0915	26389	253.7	6858	25.4	0.0061
8	182.0728	28105	755.4	20419	75.6	0.0065
9	239.1410	30861	235.9	6378	23.6	0.0077
10	275.1878	31767	301.6	8153	30.2	0.0087

#	m/z	Res.	S/N	I	I %	FWHM
1	41.0368	15404	149.5	1383	15.0	0.0027
2	65.0363	17477	376.7	3485	37.7	0.0037
3	69.0673	17705	263.2	2435	26.3	0.0039
4	92.0466	19272	278.7	2579	27.9	0.0048
5	119.0565	23282	521.1	4821	52.1	0.0051
6	125.0553	24576	281.9	2608	28.2	0.0051
7	163.0287	26822	672.5	6222	67.3	0.0061
8	182.0727	27873	900.0	8326	90.0	0.0065
9	204.0538	28342	999.7	9249	100.0	0.0072
10	220.0275	28371	191.5	1772	19.2	0.0078

Cmpd 19, Dissect, 3.7 min

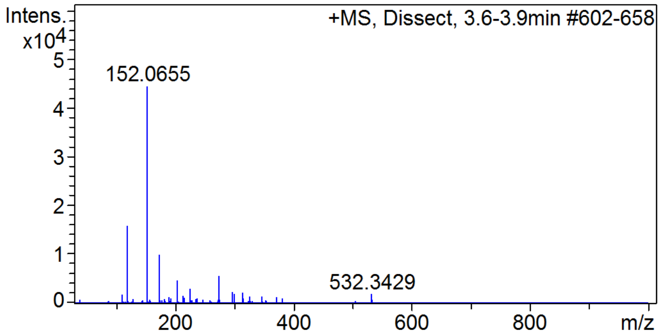

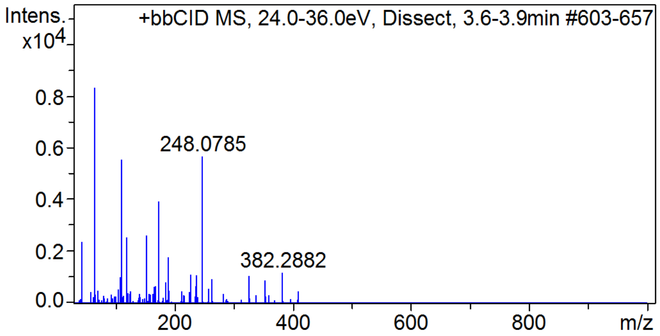

#	m/z	Res.	S/N	I	I %	FWHM
1	119.0565	24124	359.2	15965	35.9	0.0049
2	152.0655	26743	999.2	44412	100.0	0.0057
3	153.0687	26014	59.0	2623	5.9	0.0059
4	174.0466	27846	225.0	10001	22.5	0.0063
5	204.1165	27971	107.7	4788	10.8	0.0073
6	226.0975	29764	69.2	3076	6.9	0.0076
7	275.1875	31679	129.6	5759	13.0	0.0087
8	297.1452	28537	53.2	2363	5.3	0.0104
9	300.1921	30629	45.1	2003	4.5	0.0098
10	314.1702	30580	52.7	2343	5.3	0.0103

#	m/z	Res.	S/N	I	I %	FWHM
1	43.0161	15372	285.2	2376	28.6	0.0028
2	43.0524	15107	141.9	1182	14.2	0.0028
3	65.0363	17365	998.6	8319	100.0	0.0037
4	110.0565	21177	665.1	5541	66.6	0.0052
5	119.0565	23255	307.9	2565	30.8	0.0051
6	152.0655	26796	314.8	2623	31.5	0.0057
7	174.0467	28067	473.7	3947	47.4	0.0062
8	190.0199	28319	213.4	1778	21.4	0.0067
9	248.0785	29687	680.4	5669	68.1	0.0084
10	382.2882	33054	144.6	1204	14.5	0.0116

Compound Spectrum List Report

Cmpd 20, Dissect, 3.8 min

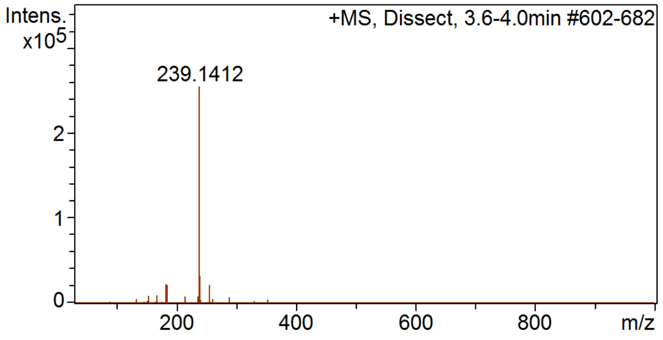

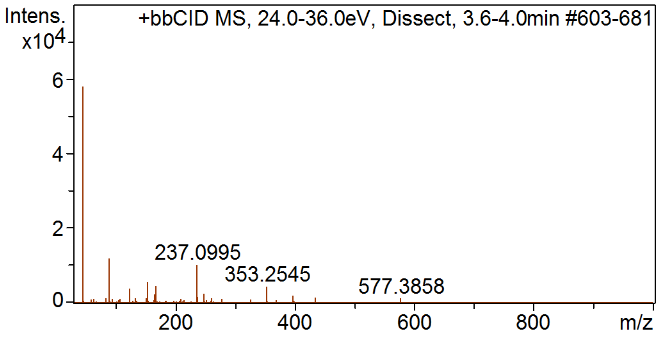

#	m/z	Res.	S/N	I	I %	FWHM
1	153.0834	25443	34.6	8848	3.5	0.0060
2	168.0951	24962	35.7	9128	3.6	0.0067
3	183.0929	28810	90.2	23092	9.1	0.0064
4	186.1064	28465	85.9	21997	8.6	0.0065
5	215.1180	29490	31.8	8144	3.2	0.0073
6	237.0993	29316	30.8	7887	3.1	0.0081
7	239.1412	30556	995.8	254856	100.0	0.0078
8	240.1446	31692	127.2	32545	12.8	0.0076
9	256.1671	32761	85.2	21800	8.6	0.0078
10	289.1976	34532	28.8	7367	2.9	0.0084

#	m/z	Res.	S/N	I	I %	FWHM
1	45.0316	16004	997.7	57971	100.0	0.0028
2	89.0569	18635	207.2	12037	20.8	0.0048
3	123.0764	23155	70.1	4070	7.0	0.0053
4	153.0834	25898	97.7	5678	9.8	0.0059
5	166.0783	27191	40.0	2323	4.0	0.0061
6	168.0942	27930	79.6	4625	8.0	0.0060
7	237.0995	30299	177.2	10294	17.8	0.0078
8	249.0527	28753	46.6	2710	4.7	0.0087
9	353.2545	32249	76.4	4441	7.7	0.0110
10	397.2578	31411	34.4	1998	3.4	0.0126

Cmpd 21, Dissect, 3.9 min

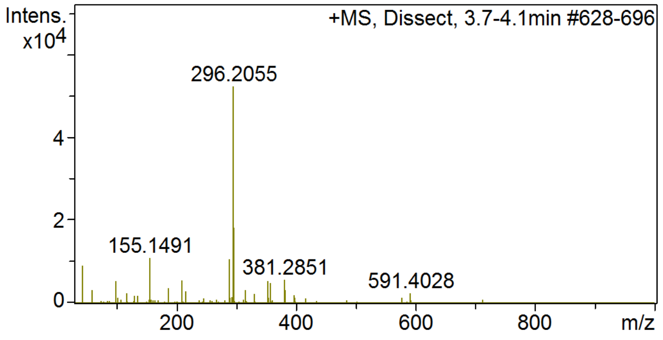

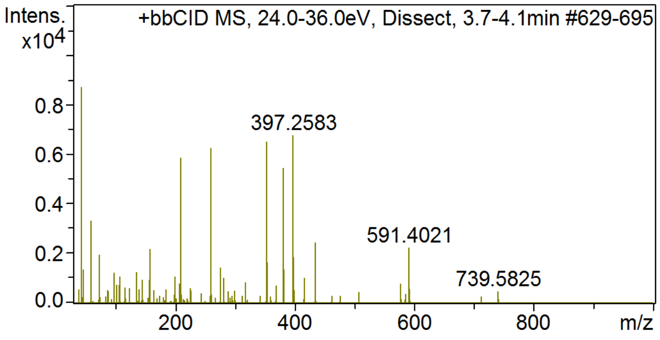

#	m/z	Res.	S/N	I	I %	FWHM
1	43.0161	15577	173.9	9102	17.4	0.0028
2	99.0774	20682	104.6	5476	10.5	0.0048
3	155.1491	25954	211.9	11086	21.2	0.0060
4	210.1037	27705	107.2	5608	10.7	0.0076
5	289.1987	32876	204.6	10706	20.5	0.0088
6	296.2055	34300	998.8	52265	100.0	0.0086
7	296.7070	34638	349.4	18285	35.0	0.0086
8	353.2546	32744	103.8	5432	10.4	0.0108
9	358.1956	33099	95.0	4969	9.5	0.0108
10	381.2851	35216	111.5	5835	11.2	0.0108

#	m/z	Res.	S/N	I	I %	FWHM
1	43.0161	15354	999.4	8707	100.0	0.0028
2	43.0524	15441	255.4	2225	25.6	0.0028
3	59.0469	17276	381.8	3326	38.2	0.0034
4	210.1032	29098	672.5	5859	67.3	0.0072
5	260.0784	29940	716.5	6242	71.7	0.0087
6	353.2546	30990	746.8	6506	74.7	0.0114
7	381.2847	36191	625.5	5449	62.6	0.0105
8	397.2583	33405	776.3	6763	77.7	0.0119
9	434.2970	30310	282.7	2463	28.3	0.0143
10	591.4021	31572	257.5	2244	25.8	0.0187

Compound Spectrum List Report

Cmpd 22, Dissect, 4.0 min

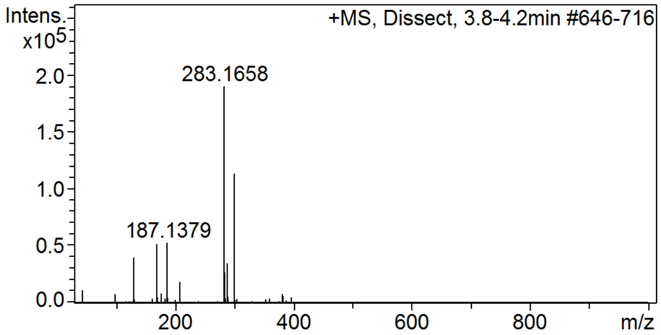

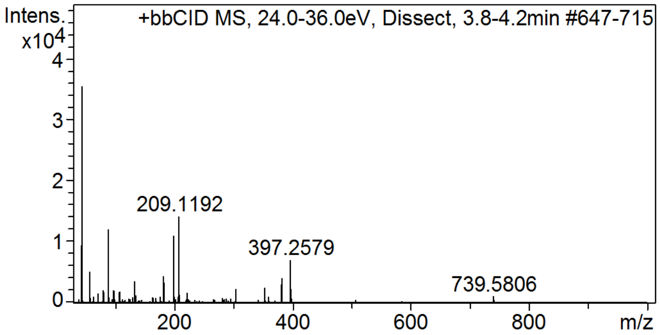

#	m/z	Res.	S/N	I	I %	FWHM
1	130.1546	24278	210.7	40199	21.2	0.0054
2	170.1118	27729	271.2	51738	27.2	0.0061
3	170.1482	26677	63.5	12120	6.4	0.0064
4	187.1379	29027	278.8	53173	28.0	0.0064
5	209.1192	29348	99.1	18904	10.0	0.0071
6	283.1658	31767	995.6	189915	100.0	0.0089
7	284.1692	32130	142.3	27152	14.3	0.0088
8	289.2026	32356	182.2	34759	18.3	0.0089
9	300.1919	32534	595.3	113554	59.8	0.0092
10	301.1951	32822	83.3	15889	8.4	0.0092

#	m/z	Res.	S/N	I	I %	FWHM
1	43.0160	15492	268.0	9505	26.8	0.0028
2	45.0316	15928	999.2	35431	100.0	0.0028
3	57.0676	17158	146.9	5208	14.7	0.0033
4	89.0568	19351	339.3	12030	34.0	0.0046
5	133.0815	24126	101.6	3604	10.2	0.0055
6	183.0952	27218	126.2	4476	12.6	0.0067
7	200.1215	29387	310.6	11015	31.1	0.0068
8	209.1192	29701	399.5	14167	40.0	0.0070
9	382.2882	33733	117.3	4158	11.7	0.0113
10	397.2579	32282	197.7	7010	19.8	0.0123

Cmpd 23, Dissect, 4.2 min

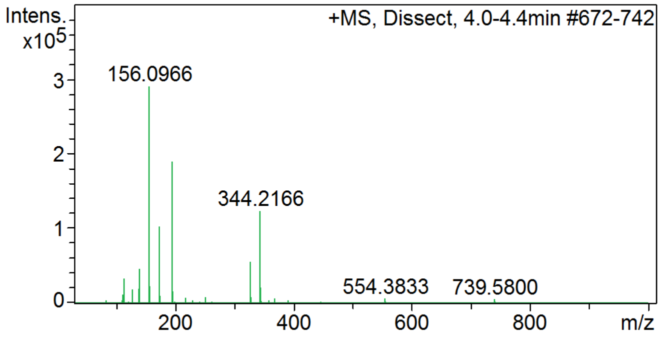

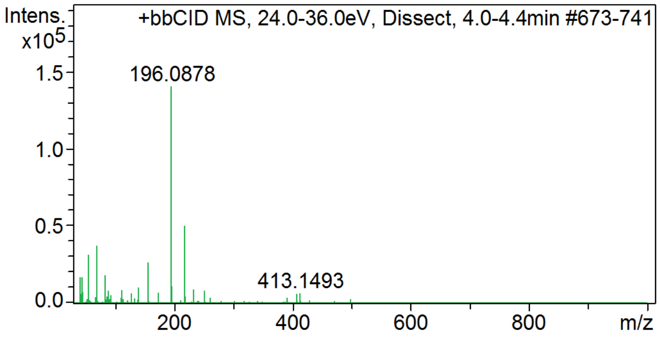

#	m/z	Res.	S/N	I	I %	FWHM
1	114.0876	22462	114.7	33532	11.5	0.0051
2	138.0866	25065	68.0	19889	6.8	0.0055
3	139.0707	25226	158.9	46464	16.0	0.0055
4	156.0966	28106	993.5	290555	100.0	0.0056
5	157.0999	25524	80.7	23608	8.1	0.0062
6	174.1067	28201	353.9	103493	35.6	0.0062
7	196.0879	29563	648.5	189662	65.3	0.0066
8	327.1907	32940	191.7	56066	19.3	0.0099
9	344.2166	33408	423.9	123958	42.7	0.0103
10	345.2203	30720	74.3	21721	7.5	0.0112

#	m/z	Res.	S/N	I	I %	FWHM
1	41.0368	15166	121.5	17083	12.2	0.0027
2	44.0112	15276	121.0	17006	12.1	0.0029
3	55.0520	16989	226.4	31836	22.6	0.0032
4	69.0673	17954	266.1	37412	26.6	0.0038
5	83.0827	19251	130.3	18324	13.0	0.0043
6	139.0707	25351	75.8	10659	7.6	0.0055
7	156.0966	26977	189.1	26581	18.9	0.0058
8	196.0878	28944	999.8	140567	100.0	0.0068
9	197.0912	28513	81.4	11443	8.1	0.0069
10	218.0691	30724	359.2	50506	35.9	0.0071

Compound Spectrum List Report

Cmpd 24, Dissect, 4.3 min

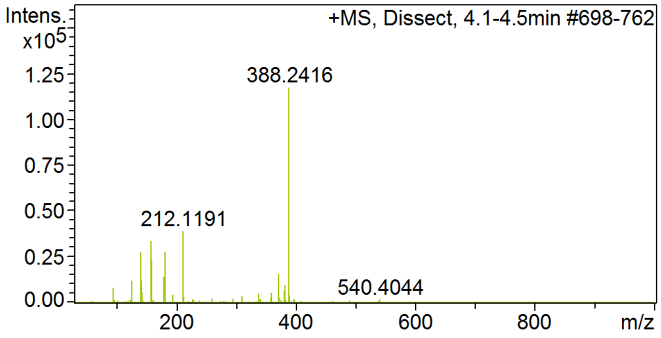

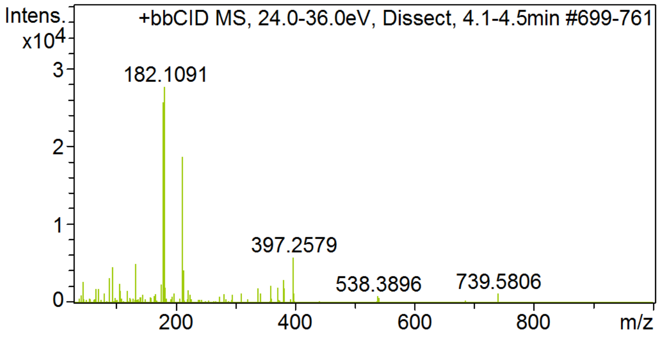

#	m/z	Res.	S/N	I	I %	FWHM
1	141.0863	25206	234.5	27562	23.5	0.0056
2	142.1180	25505	106.3	12494	10.6	0.0056
3	158.1123	26767	287.5	33795	28.8	0.0059
4	160.1280	26926	200.5	23564	20.1	0.0059
5	180.0937	27775	121.9	14326	12.2	0.0065
6	182.1092	27829	237.0	27858	23.7	0.0065
7	212.1191	28170	331.6	38976	33.2	0.0075
8	371.2156	31537	135.4	15915	13.6	0.0118
9	388.2416	34780	998.8	117410	100.0	0.0112
10	389.2448	31980	183.4	21563	18.4	0.0122

#	m/z	Res.	S/N	I	I %	FWHM
1	45.0316	16030	99.1	2749	9.9	0.0028
2	89.0569	18794	117.5	3260	11.8	0.0047
3	95.0825	19953	167.8	4654	16.8	0.0048
4	133.0815	24278	181.5	5035	18.2	0.0055
5	180.0934	28295	922.8	25600	92.4	0.0064
6	182.1091	28692	998.9	27709	100.0	0.0063
7	212.1186	29489	672.4	18654	67.3	0.0072
8	214.1367	30769	152.3	4224	15.2	0.0070
9	381.2847	35374	107.7	2989	10.8	0.0108
10	397.2579	32582	210.7	5846	21.1	0.0122

Cmpd 25, Dissect, 4.3 min

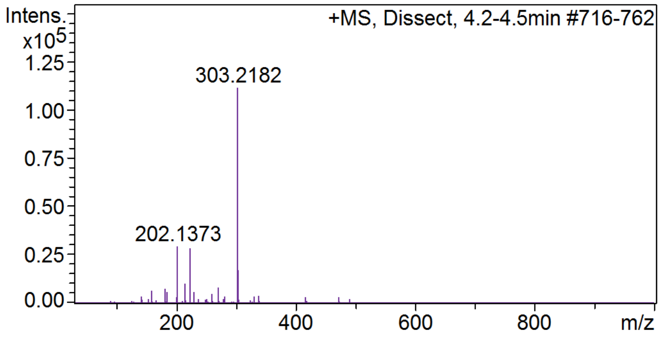

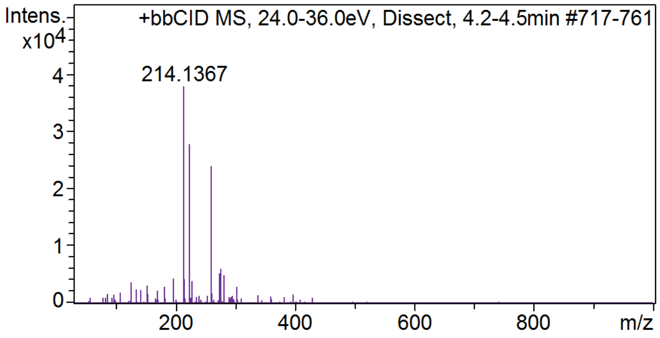

#	m/z	Res.	S/N	I	I %	FWHM
1	160.1279	26537	60.1	6737	6.0	0.0060
2	182.1093	27484	68.3	7661	6.9	0.0066
3	185.1111	27628	55.0	6168	5.5	0.0067
4	202.1373	29227	262.3	29413	26.4	0.0069
5	215.9715	29255	94.2	10562	9.5	0.0074
6	224.1186	30462	255.3	28628	25.7	0.0074
7	230.1314	29020	54.6	6118	5.5	0.0079
8	271.1562	30260	76.4	8562	7.7	0.0090
9	303.2182	33449	994.4	111500	100.0	0.0091
10	304.2215	31079	155.5	17430	15.6	0.0098

#	m/z	Res.	S/N	I	I %	FWHM
1	125.0917	23884	97.4	3697	9.8	0.0052
2	197.1106	28117	116.6	4427	11.7	0.0070
3	214.1367	30728	996.8	37849	100.0	0.0070
4	215.1398	26637	114.6	4351	11.5	0.0081
5	224.1184	30693	729.8	27711	73.2	0.0073
6	228.1518	29464	105.5	4006	10.6	0.0077
7	259.9338	31932	629.8	23913	63.2	0.0081
8	274.0934	29312	139.6	5302	14.0	0.0094
9	275.9071	30762	159.3	6049	16.0	0.0090
10	282.1219	30617	130.9	4971	13.1	0.0092

Compound Spectrum List Report

Cmpd 26, Dissect, 4.4 min

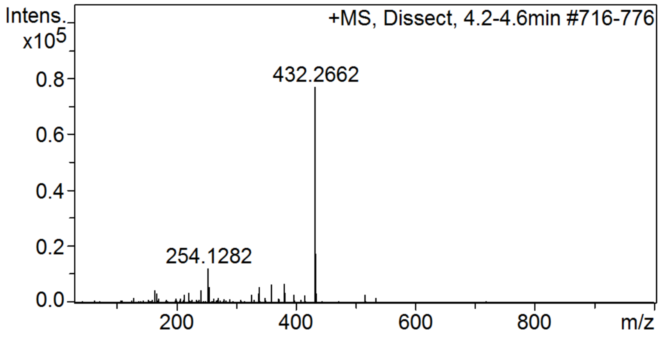

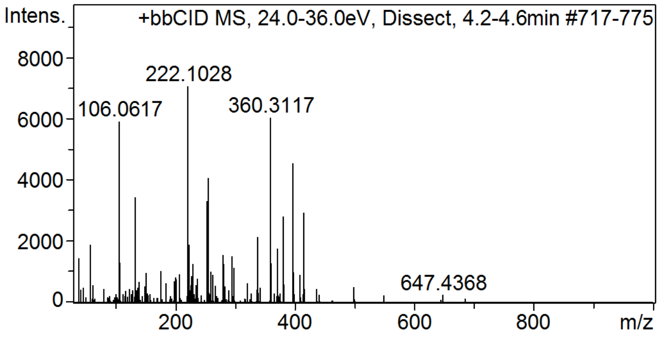

#	m/z	Res.	S/N	I	I %	FWHM
1	165.0857	27162	60.8	4693	6.1	0.0061
2	222.1030	27215	50.0	3863	5.0	0.0082
3	242.1307	29932	60.3	4654	6.0	0.0081
4	254.1282	30541	159.9	12342	16.0	0.0083
5	256.1439	30277	74.3	5733	7.4	0.0085
6	339.3343	33126	76.5	5907	7.7	0.0102
7	360.3101	31651	86.8	6700	8.7	0.0114
8	381.2849	35828	89.0	6874	8.9	0.0106
9	432.2662	34807	997.4	77008	100.0	0.0124
10	433.2695	33796	229.2	17693	23.0	0.0128

#	m/z	Res.	S/N	I	I %	FWHM
1	106.0617	21893	833.7	5895	83.6	0.0048
2	133.0816	24324	488.0	3450	48.9	0.0055
3	222.1028	28742	997.8	7055	100.0	0.0077
4	254.1285	29589	468.0	3309	46.9	0.0086
5	256.1436	28071	574.2	4060	57.5	0.0091
6	338.3307	32842	303.1	2143	30.4	0.0103
7	360.3117	33566	850.8	6016	85.3	0.0107
8	381.2851	35747	399.9	2828	40.1	0.0107
9	397.2584	31607	643.8	4552	64.5	0.0126
10	415.2403	31812	414.9	2934	41.6	0.0131

Cmpd 27, Dissect, 4.5 min

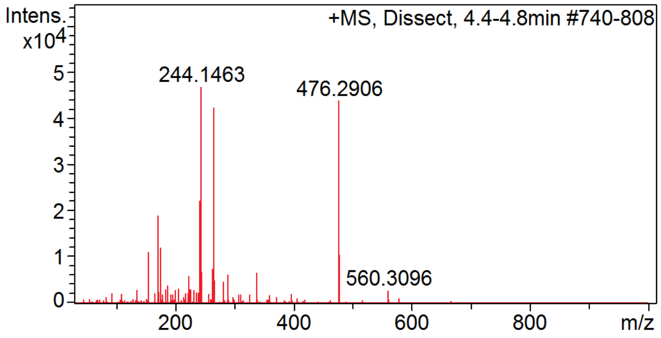

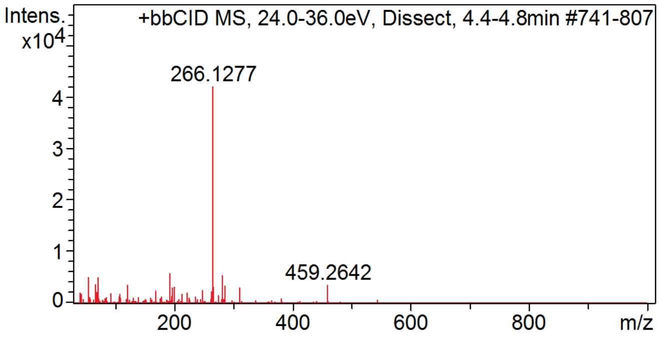

#	m/z	Res.	S/N	I	I %	FWHM
1	155.1014	26309	238.2	11188	23.9	0.0059
2	172.1274	27706	403.7	18961	40.5	0.0062
3	176.1223	27422	257.2	12078	25.8	0.0064
4	242.1306	30577	470.9	22119	47.2	0.0079
5	244.1463	31910	997.4	46845	100.0	0.0077
6	245.1493	28739	146.4	6877	14.7	0.0085
7	264.1120	30826	159.9	7508	16.0	0.0086
8	266.1276	32775	898.9	42222	90.1	0.0081
9	476.2906	33041	930.8	43719	93.3	0.0144
10	477.2935	31601	223.9	10514	22.4	0.0151

#	m/z	Res.	S/N	I	I %	FWHM
1	55.0520	17037	122.4	5148	12.2	0.0032
2	67.0519	17014	90.9	3823	9.1	0.0039
3	72.0418	18631	121.0	5089	12.1	0.0039
4	121.0972	22679	87.4	3675	8.7	0.0053
5	194.1088	28951	139.0	5844	13.9	0.0067
6	266.1277	32758	999.9	42051	100.0	0.0081
7	267.1308	29360	79.0	3324	7.9	0.0091
8	282.1010	27906	129.8	5459	13.0	0.0101
9	286.0933	30253	85.0	3574	8.5	0.0095
10	459.2642	32659	87.3	3671	8.7	0.0141

Compound Spectrum List Report

Cmpd 28, Dissect, 4.6 min

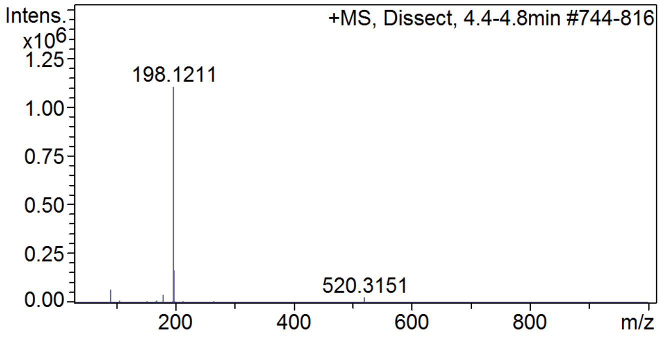

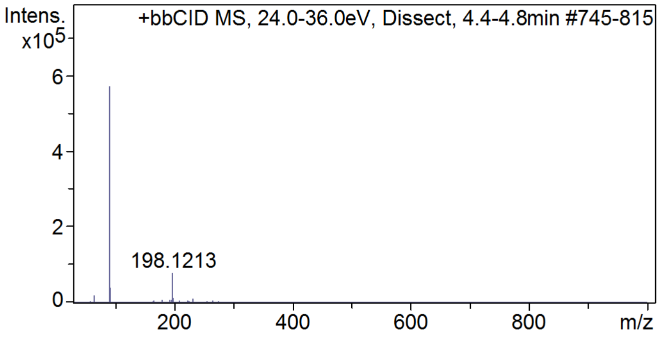

#	m/z	Res.	S/N	I	I %	FWHM
1	91.0513	20015	63.9	71423	6.5	0.0045
2	106.0617	21378	10.1	11313	1.0	0.0050
3	169.1279	27620	8.2	9165	0.8	0.0061
4	170.1118	29380	9.0	10051	0.9	0.0058
5	181.0951	27602	35.6	39757	3.6	0.0066
6	198.1211	31581	985.9	1102508	100.0	0.0063
7	199.1245	29028	149.8	167540	15.2	0.0069
8	214.1368	29004	8.2	9215	0.8	0.0074
9	266.1277	32125	6.6	7401	0.7	0.0083
10	520.3151	32259	24.4	27236	2.5	0.0161

#	m/z	Res.	S/N	I	I %	FWHM
1	65.0364	17148	32.3	18578	3.3	0.0038
2	91.0513	21642	993.8	571183	100.0	0.0042
3	92.0547	19825	72.9	41894	7.3	0.0046
4	181.0951	26621	14.6	8371	1.5	0.0068
5	194.1089	28787	12.3	7056	1.2	0.0067
6	197.1086	27284	12.6	7230	1.3	0.0072
7	198.1213	29739	138.8	79791	14.0	0.0067
8	199.1246	26879	20.7	11916	2.1	0.0074
9	210.1033	30190	11.5	6628	1.2	0.0070
10	232.0845	31157	19.8	11380	2.0	0.0074

Cmpd 29, Dissect, 4.6 min

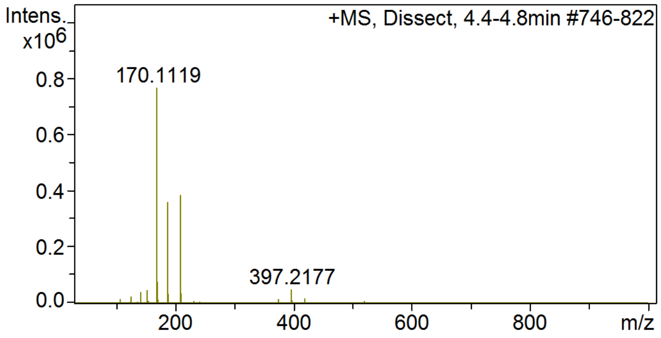

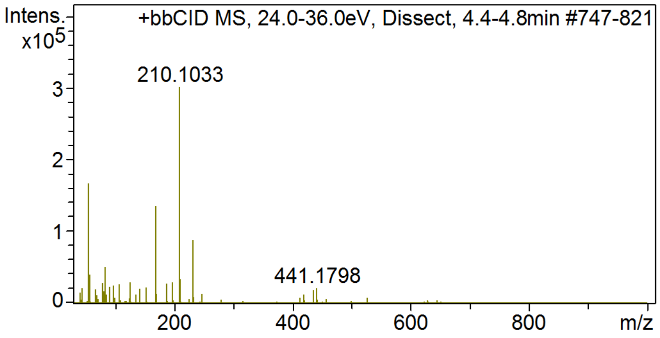

#	m/z	Res.	S/N	I	I %	FWHM
1	142.1179	25528	53.9	41624	5.4	0.0056
2	152.1018	26181	64.3	49651	6.5	0.0058
3	153.0859	25909	50.1	38737	5.0	0.0059
4	170.1119	29536	993.6	767663	100.0	0.0058
5	171.1152	27524	100.9	77993	10.2	0.0062
6	188.1219	30195	467.4	361103	47.0	0.0062
7	189.1253	26764	46.2	35689	4.6	0.0071
8	210.1032	31404	498.8	385360	50.2	0.0067
9	211.1066	27318	48.9	37806	4.9	0.0077
10	397.2177	33715	66.2	51131	6.7	0.0118

#	m/z	Res.	S/N	I	I %	FWHM
1	55.0521	17638	553.0	166962	55.3	0.0031
2	57.0677	17139	133.3	40237	13.3	0.0033
3	79.0514	19220	97.1	29311	9.7	0.0041
4	83.0827	19550	168.6	50899	16.9	0.0042
5	125.0920	23755	100.2	30255	10.0	0.0053
6	170.1120	28284	449.1	135606	44.9	0.0060
7	198.1213	29773	98.7	29786	9.9	0.0067
8	210.1033	30200	999.8	301863	100.0	0.0070
9	211.1065	28319	114.2	34496	11.4	0.0075
10	232.0845	31227	292.2	88211	29.2	0.0074

Compound Spectrum List Report

Cmpd 30, Dissect, 4.7 min

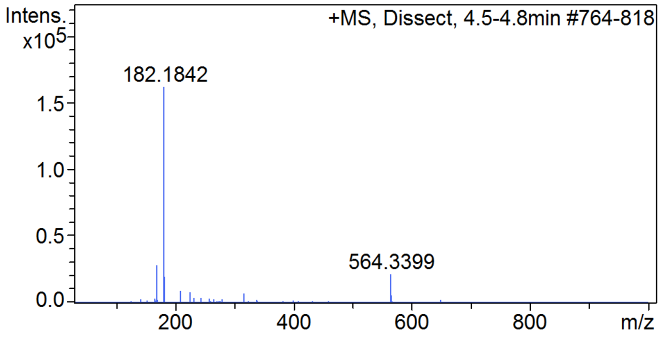

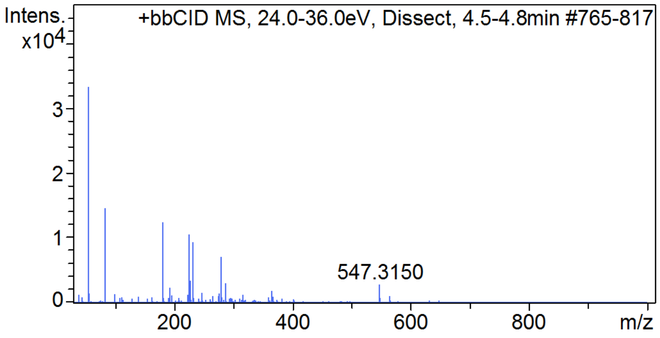

#	m/z	Res.	S/N	I	I %	FWHM
1	170.1119	29187	175.0	28436	17.6	0.0058
2	182.1842	29074	996.9	161963	100.0	0.0063
3	183.1876	26384	122.4	19879	12.3	0.0069
4	210.1032	30768	58.3	9464	5.8	0.0068
5	226.1342	29645	51.6	8385	5.2	0.0076
6	232.0844	29678	22.9	3713	2.3	0.0078
7	244.1463	30884	22.8	3697	2.3	0.0079
8	317.2333	31551	47.1	7654	4.7	0.0101
9	564.3399	31601	135.2	21965	13.6	0.0179
10	565.3442	29659	34.8	5656	3.5	0.0191

#	m/z	Res.	S/N	I	I %	FWHM
1	55.0156	16834	103.1	3445	10.3	0.0033
2	55.0521	17448	998.9	33360	100.0	0.0032
3	83.0827	19297	436.7	14585	43.7	0.0043
4	182.1843	28650	371.9	12420	37.2	0.0064
5	226.1340	29665	317.1	10591	31.7	0.0076
6	228.1519	28379	104.2	3479	10.4	0.0080
7	232.0846	31005	280.3	9362	28.1	0.0075
8	280.1429	30679	213.1	7116	21.3	0.0091
9	288.1089	30533	92.3	3082	9.2	0.0094
10	547.3150	32401	87.9	2935	8.8	0.0169

Cmpd 31, Dissect, 4.7 min

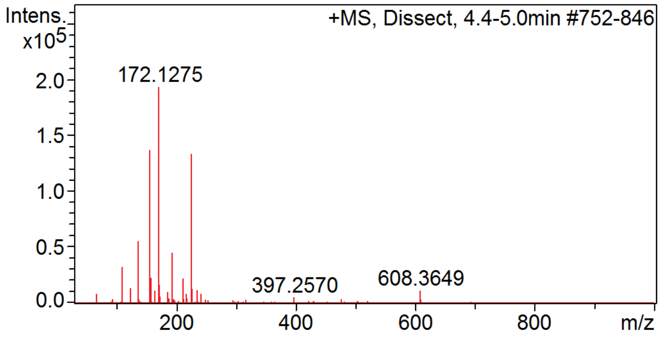

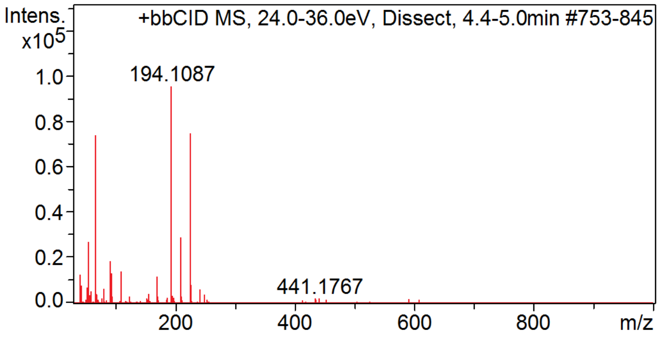

#	m/z	Res.	S/N	I	I %	FWHM
1	109.0976	21978	168.6	32589	16.9	0.0050
2	123.0764	23577	71.0	13711	7.1	0.0052
3	136.0712	24171	287.6	55584	28.8	0.0056
4	155.1015	27139	707.1	136646	70.7	0.0057
5	159.0599	26090	120.8	23335	12.1	0.0061
6	172.1275	28711	999.9	193213	100.0	0.0060
7	173.1116	28044	86.2	16657	8.6	0.0062
8	194.1088	28727	235.7	45550	23.6	0.0068
9	212.1365	28711	117.2	22646	11.7	0.0074
10	226.1341	29758	689.1	133155	68.9	0.0076

#	m/z	Res.	S/N	I	I %	FWHM
1	41.0368	15144	133.8	12781	13.4	0.0027
2	55.0521	17260	282.6	26993	28.3	0.0032
3	67.0519	17789	772.4	73779	77.4	0.0038
4	91.0514	19754	197.0	18816	19.7	0.0046
5	94.0622	19802	139.9	13366	14.0	0.0048
6	109.0976	21523	148.2	14160	14.8	0.0051
7	194.1087	29584	998.3	95353	100.0	0.0066
8	210.0822	29789	305.6	29190	30.6	0.0071
9	210.1032	29348	287.6	27471	28.8	0.0072
10	226.1339	29622	781.4	74642	78.3	0.0076

Compound Spectrum List Report

Cmpd 32, Dissect, 4.8 min

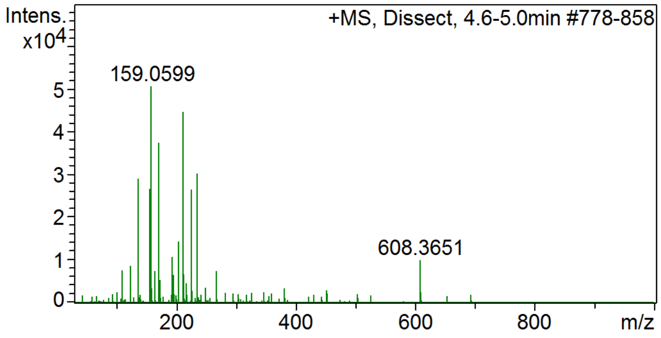

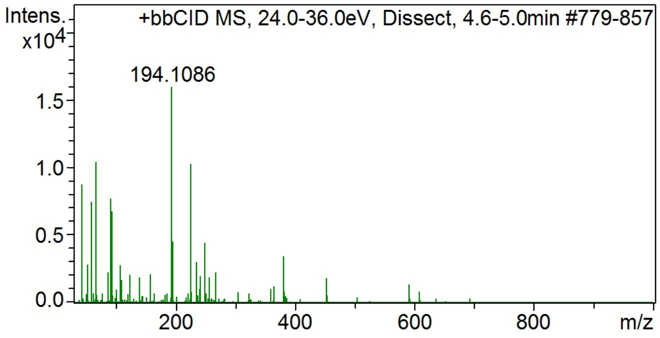

#	m/z	Res.	S/N	I	I %	FWHM
1	136.0712	24329	574.6	29078	57.5	0.0056
2	155.1015	26789	527.1	26676	52.7	0.0058
3	159.0599	26732	999.6	50588	100.0	0.0060
4	172.1275	28515	738.8	37389	73.9	0.0060
5	194.1088	28617	213.7	10816	21.4	0.0068
6	205.1003	28779	284.8	14414	28.5	0.0071
7	212.1365	29401	878.6	44465	87.9	0.0072
8	226.1344	29976	523.7	26504	52.4	0.0075
9	236.1053	30886	596.6	30192	59.7	0.0076
10	608.3651	31826	197.5	9997	19.8	0.0191

#	m/z	Res.	S/N	I	I %	FWHM
1	43.0161	15604	549.2	8773	54.9	0.0028
2	59.0470	17174	467.2	7463	46.7	0.0034
3	67.0519	17802	650.7	10395	65.1	0.0038
4	91.0514	20075	484.6	7740	48.5	0.0045
5	94.0622	20053	423.1	6759	42.3	0.0047
6	194.1086	29303	999.7	15970	100.0	0.0066
7	196.1242	27368	285.3	4558	28.5	0.0072
8	226.1339	30214	642.5	10263	64.3	0.0075
9	250.1332	30382	278.0	4440	27.8	0.0082
10	381.2846	30706	216.8	3462	21.7	0.0124

Cmpd 33, Dissect, 4.9 min

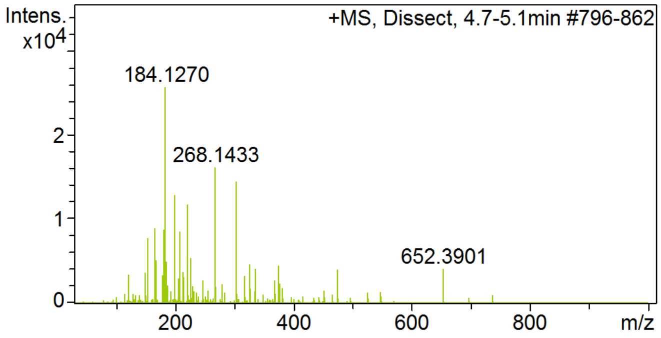

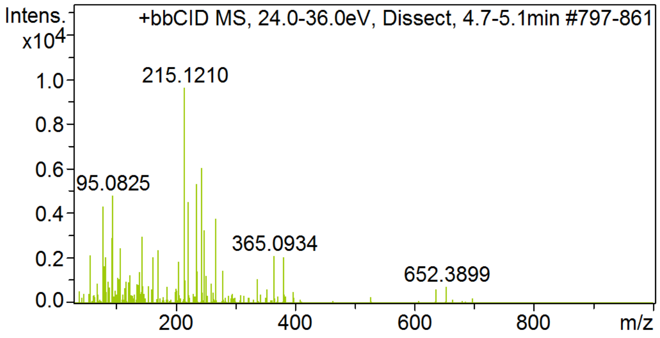

#	m/z	Res.	S/N	I	I %	FWHM
1	154.1176	25844	302.2	7781	30.2	0.0060
2	167.1011	27259	348.1	8964	34.8	0.0061
3	182.1115	27566	340.9	8778	34.1	0.0066
4	184.1270	28512	999.8	25744	100.0	0.0065
5	200.0642	29117	501.3	12909	50.1	0.0069
6	209.1102	28229	333.3	8583	33.3	0.0074
7	222.1028	29438	455.4	11726	45.5	0.0075
8	227.1203	30620	210.9	5431	21.1	0.0074
9	268.1433	31533	626.6	16135	62.7	0.0085
10	304.2022	32389	562.7	14489	56.3	0.0094

#	m/z	Res.	S/N	I	I %	FWHM
1	79.0515	19125	447.7	4315	44.8	0.0041
2	93.0670	18431	302.8	2919	30.3	0.0050
3	95.0825	19953	500.4	4823	50.0	0.0048
4	144.0759	25099	310.4	2992	31.0	0.0057
5	215.1210	28739	999.8	9637	100.0	0.0075
6	222.1029	28278	468.0	4510	46.8	0.0079
7	236.1180	28931	551.9	5319	55.2	0.0082
8	244.0841	29917	627.6	6049	62.8	0.0082
9	248.1177	28075	338.8	3265	33.9	0.0088
10	268.1431	29765	392.5	3783	39.3	0.0090

Compound Spectrum List Report

Cmpd 34, Dissect, 5.0 min

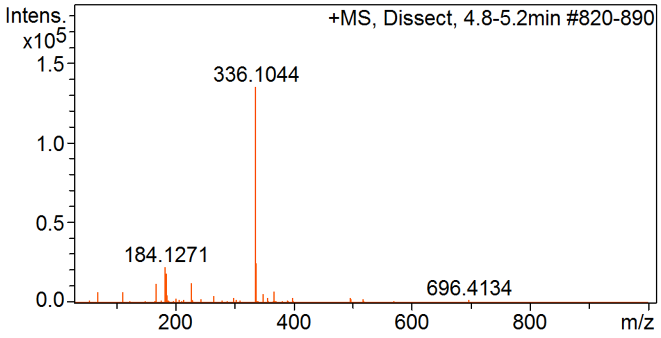

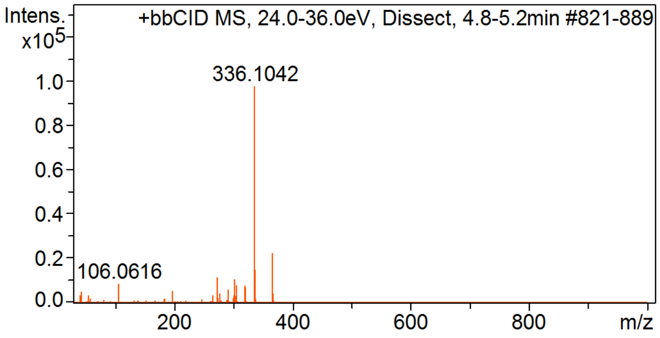

#	m/z	Res.	S/N	I	I %	FWHM
1	69.0673	17944	50.1	6794	5.0	0.0038
2	111.1132	22353	51.3	6963	5.2	0.0050
3	169.1160	25903	90.1	12219	9.1	0.0065
4	184.1271	28163	166.4	22561	16.7	0.0065
5	186.1427	28756	135.8	18416	13.6	0.0065
6	228.1056	30268	93.0	12605	9.3	0.0075
7	336.1044	33056	995.4	134972	100.0	0.0102
8	337.1079	31164	183.0	24808	18.4	0.0108
9	348.7114	34283	39.2	5313	3.9	0.0102
10	368.0751	32219	53.2	7219	5.3	0.0114

#	m/z	Res.	S/N	I	I %	FWHM
1	106.0616	22043	89.1	8713	8.9	0.0048
2	274.0696	31200	117.9	11529	11.8	0.0088
3	292.0795	31126	64.4	6303	6.5	0.0094
4	303.0711	30376	110.1	10772	11.0	0.0100
5	306.0585	30909	84.0	8218	8.4	0.0099
6	320.0734	32422	79.5	7779	8.0	0.0099
7	321.0809	31215	74.0	7241	7.4	0.0103
8	336.1042	34556	997.2	97524	100.0	0.0097
9	337.1088	27286	155.0	15159	15.5	0.0124
10	366.0594	31639	231.7	22658	23.2	0.0116

Cmpd 35, Dissect, 5.1 min

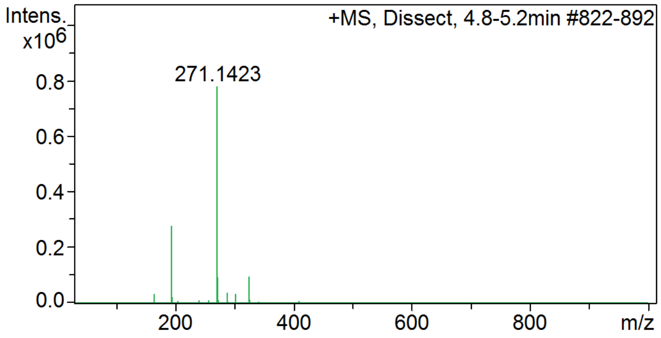

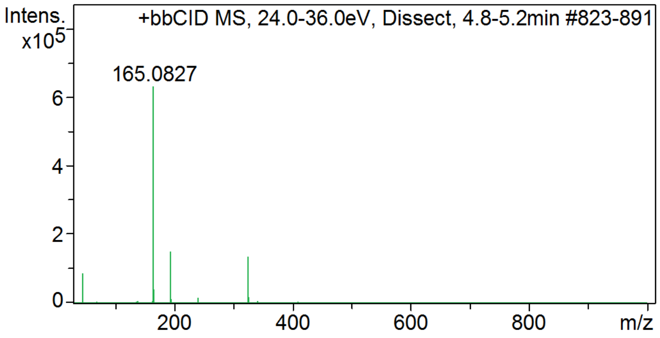

#	m/z	Res.	S/N	I	I %	FWHM
1	165.0829	26786	41.7	32739	4.2	0.0062
2	195.0924	30439	355.4	278937	35.8	0.0064
3	196.0953	23304	26.4	20753	2.7	0.0084
4	241.1334	29530	12.6	9923	1.3	0.0082
5	271.1423	35644	992.8	779146	100.0	0.0076
6	272.1452	29293	120.0	94158	12.1	0.0093
7	289.1532	29742	52.6	41275	5.3	0.0097
8	303.1678	32978	41.0	32148	4.1	0.0092
9	325.1489	33364	124.8	97960	12.6	0.0097
10	326.1517	27430	15.3	11971	1.5	0.0119

#	m/z	Res.	S/N	I	I %	FWHM
1	45.0316	15961	139.8	88229	14.0	0.0028
2	138.0726	24522	10.6	6672	1.1	0.0056
3	165.0827	29073	999.7	631127	100.0	0.0057
4	166.0857	23539	67.2	42440	6.7	0.0071
5	195.0924	30523	239.0	150879	23.9	0.0064
6	196.0953	23941	19.2	12105	1.9	0.0082
7	241.1327	30916	26.0	16399	2.6	0.0078
8	325.1488	33959	216.2	136465	21.6	0.0096
9	326.1517	29857	28.1	17763	2.8	0.0109
10	341.1222	30315	11.5	7269	1.2	0.0113

Compound Spectrum List Report

Cmpd 36, Dissect, 5.1 min

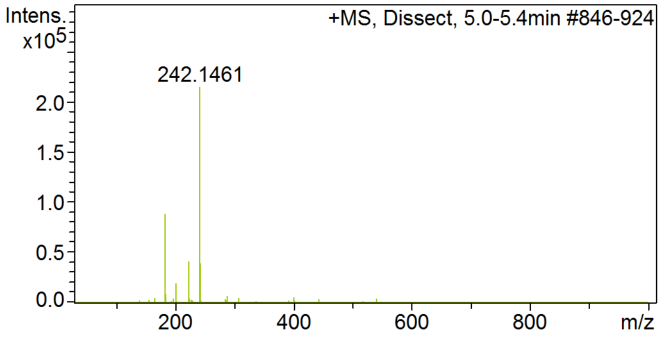

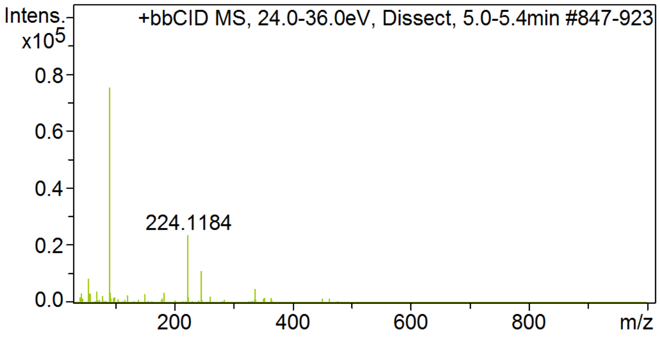

#	m/z	Res.	S/N	I	I %	FWHM
1	166.1171	26801	22.1	4765	2.2	0.0062
2	184.1271	29166	410.7	88561	41.1	0.0063
3	185.1305	26840	39.1	8441	3.9	0.0069
4	202.1371	29163	93.9	20247	9.4	0.0069
5	224.1185	30675	193.0	41623	19.3	0.0073
6	242.1461	31289	998.2	215256	100.0	0.0077
7	243.1495	31197	183.8	39631	18.4	0.0078
8	289.1532	32201	31.1	6709	3.1	0.0090
9	308.2120	30745	22.8	4910	2.3	0.0100
10	401.2494	34495	26.3	5669	2.6	0.0116

#	m/z	Res.	S/N	I	I %	FWHM
1	43.0161	15645	42.0	3162	4.2	0.0027
2	55.0521	17243	114.8	8652	11.5	0.0032
3	57.0677	17167	47.4	3568	4.7	0.0033
4	69.0675	17917	53.6	4041	5.4	0.0039
5	91.0514	20233	999.6	75313	100.0	0.0045
6	92.0547	18735	50.1	3771	5.0	0.0049
7	184.1272	28190	47.7	3595	4.8	0.0065
8	224.1184	31019	314.5	23698	31.5	0.0072
9	246.0997	29971	150.7	11356	15.1	0.0082
10	337.1140	29162	68.1	5130	6.8	0.0116

Cmpd 37, Dissect, 5.2 min

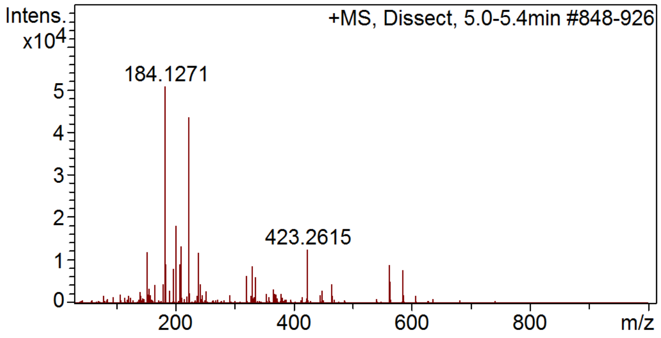

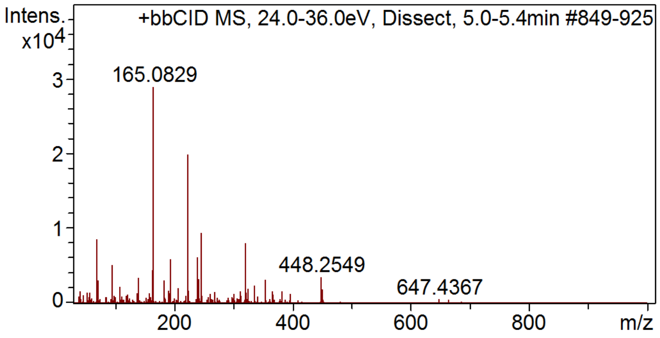

#	m/z	Res.	S/N	I	I %	FWHM
1	153.0859	26648	238.7	12113	23.9	0.0057
2	184.1271	29243	999.3	50710	100.0	0.0063
3	185.1304	27071	180.5	9161	18.1	0.0068
4	202.1371	29074	357.6	18149	35.8	0.0070
5	209.1099	27337	179.8	9125	18.0	0.0076
6	211.1259	28906	265.2	13458	26.5	0.0073
7	224.1185	30723	857.1	43498	85.8	0.0073
8	240.1495	29250	232.9	11821	23.3	0.0082
9	423.2615	34386	249.4	12658	25.0	0.0123
10	562.3249	33118	178.8	9074	17.9	0.0170

#	m/z	Res.	S/N	I	I %	FWHM
1	69.0675	17758	297.0	8592	29.7	0.0039
2	95.0463	19860	179.9	5203	18.0	0.0048
3	164.9150	27757	154.3	4463	15.4	0.0059
4	165.0829	27516	999.9	28921	100.0	0.0060
5	195.0924	29287	205.9	5957	20.6	0.0067
6	224.1184	31035	686.9	19867	68.7	0.0072
7	240.0916	28141	138.5	4006	13.9	0.0085
8	240.1492	29906	213.2	6168	21.3	0.0080
9	246.0997	30089	327.2	9463	32.7	0.0082
10	321.1203	31691	278.1	8044	27.8	0.0101

Compound Spectrum List Report

Cmpd 38, Dissect, 5.4 min

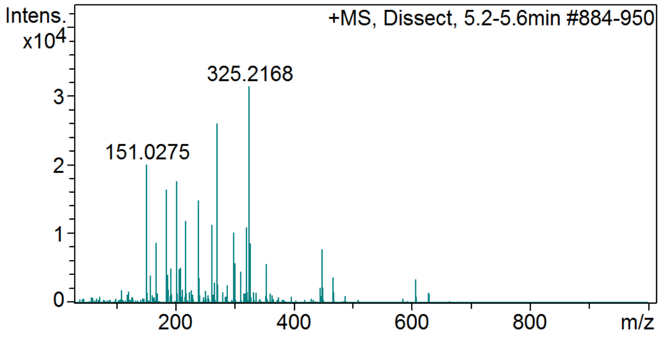

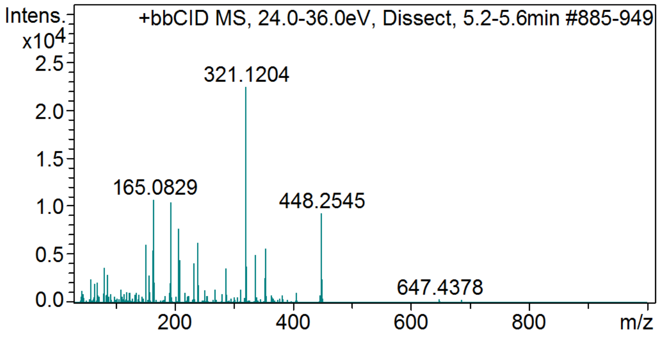

#	m/z	Res.	S/N	I	I %	FWHM
1	151.0275	26129	638.5	20040	63.9	0.0058
2	186.1428	28100	522.2	16390	52.2	0.0066
3	203.1325	28823	560.6	17594	56.1	0.0070
4	218.2044	29026	379.2	11902	37.9	0.0075
5	240.1492	29937	472.9	14840	47.3	0.0080
6	263.1040	31467	359.8	11292	36.0	0.0084
7	271.1425	31715	825.2	25900	82.6	0.0085
8	299.1374	28903	325.2	10208	32.5	0.0103
9	321.1203	32076	348.2	10929	34.8	0.0100
10	325.2168	33041	999.6	31372	100.0	0.0098

#	m/z	Res.	S/N	I	I %	FWHM
1	151.0275	26142	270.4	6080	27.1	0.0058
2	164.9150	27801	243.7	5479	24.4	0.0059
3	165.0829	27208	475.6	10693	47.6	0.0061
4	195.0925	28692	466.0	10476	46.7	0.0068
5	208.1241	28588	342.7	7705	34.3	0.0073
6	240.1492	30296	280.0	6295	28.0	0.0079
7	321.1204	31793	998.3	22445	100.0	0.0101
8	337.0937	29948	223.7	5029	22.4	0.0113
9	355.1581	30174	252.6	5678	25.3	0.0118
10	448.2545	32683	413.5	9297	41.4	0.0137

Cmpd 39, Dissect, 5.5 min

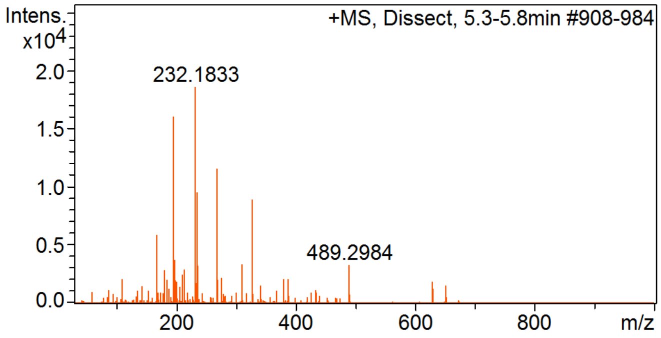

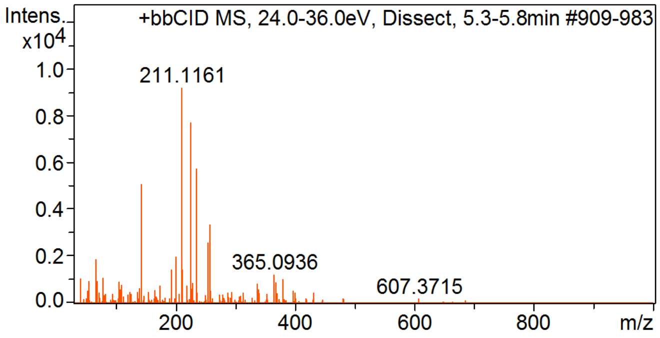

#	m/z	Res.	S/N	I	I %	FWHM
1	168.1329	26768	316.8	5894	31.7	0.0063
2	196.1268	27760	859.7	15996	86.0	0.0071
3	198.1425	29379	203.4	3785	20.3	0.0067
4	232.1833	30250	999.6	18601	100.0	0.0077
5	236.1184	29545	513.5	9555	51.4	0.0080
6	237.0681	30688	174.6	3249	17.5	0.0077
7	269.1927	31062	622.0	11574	62.2	0.0087
8	311.0040	31824	182.8	3401	18.3	0.0098
9	328.0299	33476	480.0	8932	48.0	0.0098
10	489.2984	33823	178.3	3317	17.8	0.0145

#	m/z	Res.	S/N	I	I %	FWHM
1	67.0519	17419	204.4	1883	20.5	0.0038
2	143.1019	24525	551.4	5078	55.2	0.0058
3	202.0366	29289	218.5	2013	21.9	0.0069
4	211.0869	28846	162.2	1493	16.2	0.0073
5	211.1161	29662	999.5	9204	100.0	0.0071
6	212.1194	24779	158.9	1464	15.9	0.0086
7	226.1391	29904	835.1	7690	83.5	0.0076
8	236.1181	30369	624.4	5750	62.5	0.0078
9	255.0494	29823	283.1	2607	28.3	0.0086
10	258.0993	29431	365.7	3367	36.6	0.0088

Compound Spectrum List Report

Cmpd 40, Dissect, 5.6 min

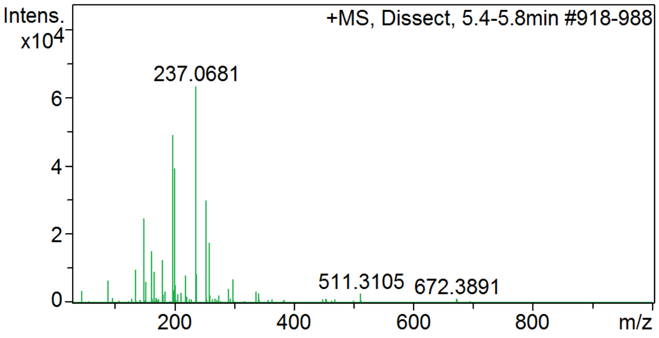

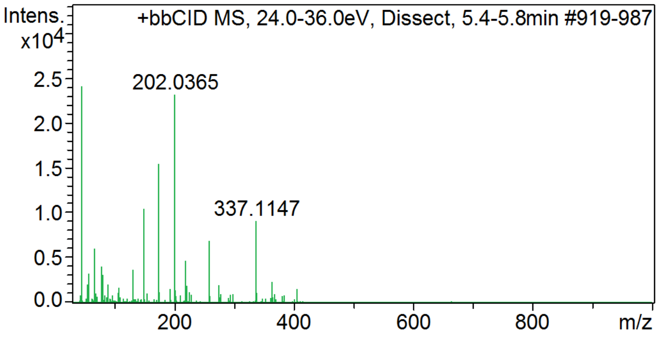

#	m/z	Res.	S/N	I	I %	FWHM
1	135.1124	24428	154.3	9804	15.5	0.0055
2	149.0184	26093	389.0	24720	39.0	0.0057
3	163.1275	26916	238.1	15132	23.9	0.0061
4	167.1010	27194	145.2	9226	14.6	0.0061
5	181.1164	26965	196.9	12511	19.8	0.0067
6	198.1424	29445	771.8	49053	77.5	0.0067
7	202.0368	29762	618.0	39276	62.0	0.0068
8	237.0681	30888	996.4	63327	100.0	0.0077
9	254.0943	31334	472.5	30031	47.4	0.0081
10	259.0494	30905	278.2	17681	27.9	0.0084

#	m/z	Res.	S/N	I	I %	FWHM
1	45.0317	15831	998.6	24099	100.0	0.0028
2	67.0519	17459	251.9	6079	25.2	0.0038
3	79.0515	18908	168.3	4063	16.9	0.0042
4	131.0560	24125	154.4	3726	15.5	0.0054
5	149.0184	25625	435.1	10502	43.6	0.0058
6	175.0267	27732	639.0	15422	64.0	0.0063
7	202.0365	29611	962.0	23216	96.3	0.0068
8	220.1238	29045	196.9	4752	19.7	0.0076
9	259.0493	30712	286.5	6914	28.7	0.0084
10	337.1147	31873	378.6	9137	37.9	0.0106

Cmpd 41, Dissect, 5.7 min

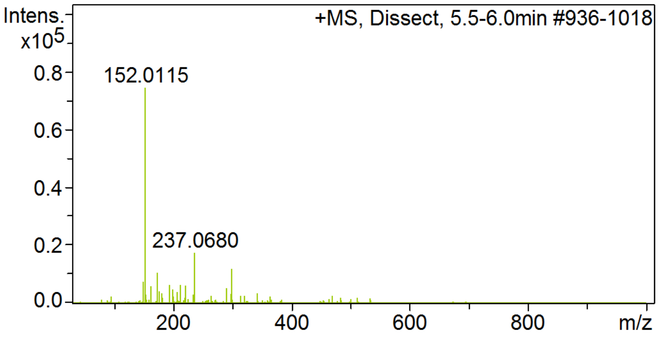

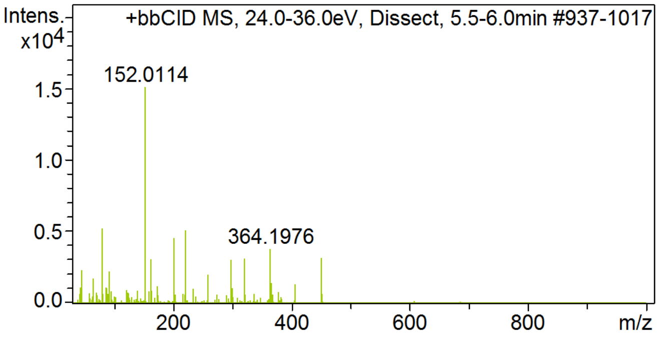

#	m/z	Res.	S/N	I	I %	FWHM
1	149.0186	25950	102.2	7633	10.2	0.0057
2	152.0115	26752	999.4	74647	100.0	0.0057
3	152.1100	25672	81.9	6116	8.2	0.0059
4	163.1275	26703	81.6	6093	8.2	0.0061
5	173.9926	27267	142.1	10612	14.2	0.0064
6	195.1316	28111	86.7	6474	8.7	0.0069
7	213.1416	28599	88.4	6604	8.8	0.0075
8	222.1394	27391	84.5	6315	8.5	0.0081
9	237.0680	29823	235.5	17594	23.6	0.0079
10	299.2233	32397	160.6	11999	16.1	0.0092

#	m/z	Res.	S/N	I	I %	FWHM
1	45.0316	15793	155.5	2350	15.6	0.0029
2	80.0468	19243	346.4	5235	34.7	0.0042
3	152.0114	25897	999.8	15108	100.0	0.0059
4	163.0674	26395	204.6	3092	20.5	0.0062
5	202.0366	29621	301.5	4556	30.2	0.0068
6	222.1391	28624	338.7	5118	33.9	0.0078
7	298.9113	31575	202.4	3058	20.2	0.0095
8	321.1203	31181	207.7	3139	20.8	0.0103
9	364.1976	30602	252.6	3818	25.3	0.0119
10	451.1434	30931	211.7	3199	21.2	0.0146

Compound Spectrum List Report

Cmpd 42, Dissect, 5.7 min

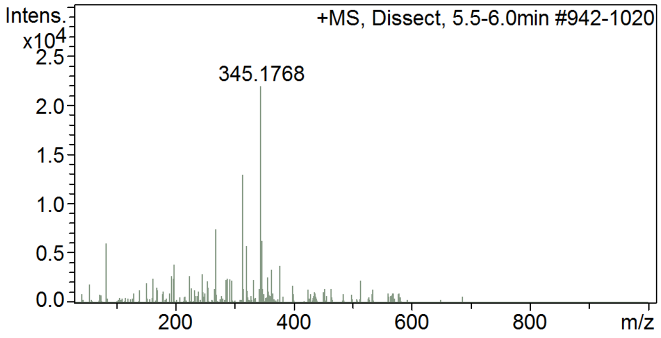

#	m/z	Res.	S/N	I	I %	FWHM
1	83.0463	19048	275.6	6048	27.6	0.0044
2	199.1260	25704	177.6	3897	17.8	0.0077
3	199.1403	22489	179.2	3933	17.9	0.0089
4	269.1271	29531	323.3	7095	32.4	0.0091
5	269.1658	28875	338.9	7437	33.9	0.0093
6	315.1673	31713	590.4	12957	59.1	0.0099
7	321.1205	31231	264.3	5801	26.5	0.0103
8	345.1768	31509	998.5	21914	100.0	0.0110
9	347.2609	33855	287.9	6319	28.8	0.0103
10	377.2023	31983	171.9	3773	17.2	0.0118

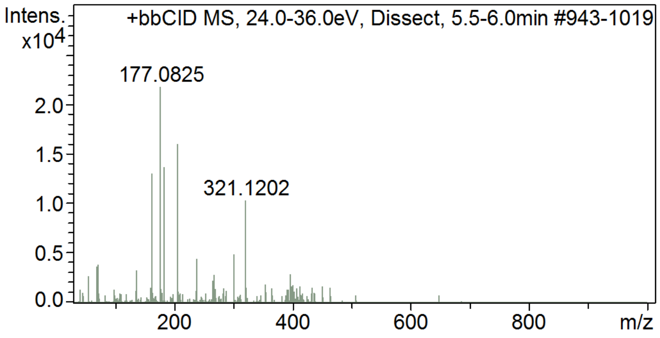

#	m/z	Res.	S/N	I	I %	FWHM
1	69.0673	17929	170.1	3712	17.0	0.0039
2	72.0418	18778	179.8	3923	18.0	0.0038
3	163.0334	26097	546.6	11925	54.7	0.0062
4	163.0673	26491	597.0	13025	59.7	0.0062
5	177.0825	27575	999.7	21809	100.0	0.0064
6	184.0933	27363	628.2	13705	62.8	0.0067
7	207.0921	29472	732.1	15971	73.2	0.0070
8	239.1174	28817	206.5	4504	20.7	0.0083
9	302.2046	32143	226.1	4932	22.6	0.0094
10	321.1202	31136	473.5	10330	47.4	0.0103

Cmpd 43, Dissect, 5.8 min

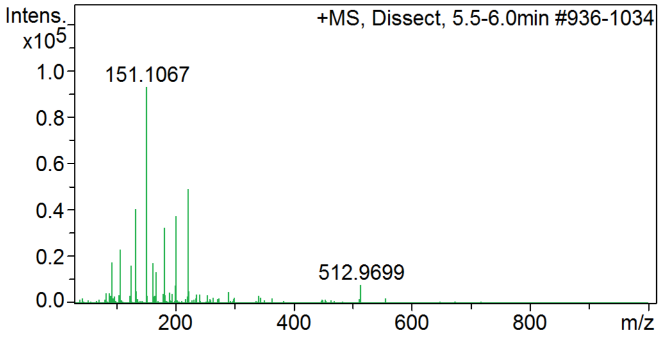

#	m/z	Res.	S/N	I	I %	FWHM
1	93.0669	18907	189.3	17625	19.0	0.0049
2	107.0822	21666	250.6	23328	25.1	0.0049
3	125.0919	23590	176.5	16428	17.7	0.0053
4	133.0968	24186	435.7	40554	43.7	0.0055
5	151.1067	26654	997.7	92870	100.0	0.0057
6	163.0335	26990	186.8	17383	18.7	0.0060
7	169.1167	26754	144.9	13491	14.5	0.0063
8	183.1318	28066	350.7	32645	35.2	0.0065
9	202.1736	28638	401.5	37374	40.2	0.0071
10	223.1233	30298	526.7	49026	52.8	0.0074

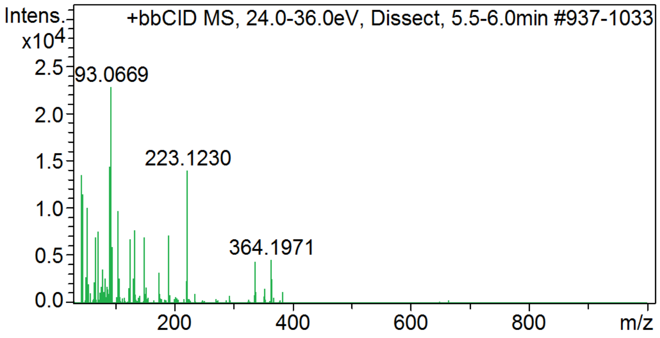

#	m/z	Res.	S/N	I	I %	FWHM
1	43.0160	15552	590.7	13494	59.1	0.0028
2	45.0316	15868	503.9	11510	50.4	0.0028
3	53.0364	16649	441.3	10081	44.1	0.0032
4	71.0466	18259	331.9	7582	33.2	0.0039
5	91.0512	19606	630.7	14407	63.1	0.0046
6	93.0669	19485	999.8	22840	100.0	0.0048
7	105.0665	21144	426.2	9737	42.6	0.0050
8	133.0967	24145	337.7	7715	33.8	0.0055
9	191.0981	27686	314.1	7176	31.4	0.0069
10	223.1230	29846	612.7	13996	61.3	0.0075

Compound Spectrum List Report

Cmpd 44, Dissect, 5.8 min

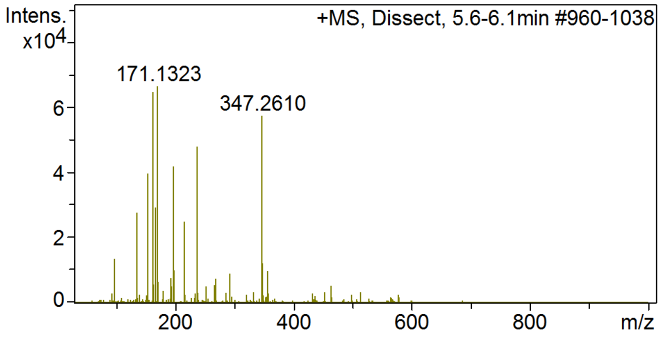

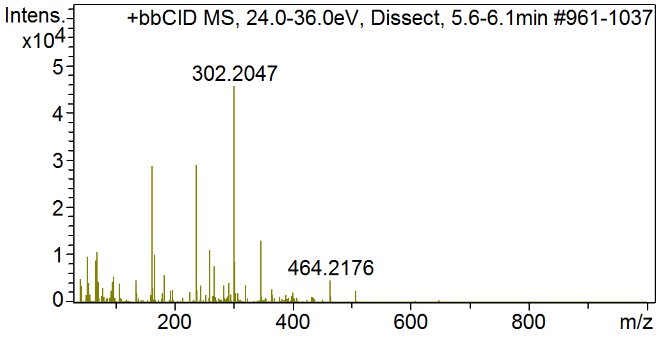

#	m/z	Res.	S/N	I	I %	FWHM
1	135.1124	24506	416.1	27704	41.7	0.0055
2	153.1223	26152	597.0	39745	59.8	0.0059
3	163.0336	26916	973.2	64797	97.5	0.0061
4	167.9880	27662	440.1	29302	44.1	0.0061
5	168.1326	27231	262.1	17451	26.2	0.0062
6	171.1323	27939	998.5	66480	100.0	0.0061
7	198.1424	29320	628.6	41854	63.0	0.0068
8	216.1525	29566	375.8	25018	37.6	0.0073
9	238.1340	30271	719.2	47885	72.0	0.0079
10	347.2610	34445	860.4	57286	86.2	0.0101

#	m/z	Res.	S/N	I	I %	FWHM
1	53.0365	16720	212.2	9693	21.2	0.0032
2	67.0519	17219	194.8	8895	19.5	0.0039
3	69.0674	17730	234.1	10692	23.4	0.0039
4	163.0335	26570	627.7	28669	62.8	0.0061
5	167.9880	27201	222.8	10176	22.3	0.0062
6	238.1336	30833	635.6	29029	63.6	0.0077
7	260.1150	30225	242.7	11086	24.3	0.0086
8	302.2047	32723	999.8	45665	100.0	0.0092
9	303.2069	30275	188.7	8617	18.9	0.0100
10	347.2610	33367	286.2	13070	28.6	0.0104

Cmpd 45, Dissect, 5.9 min

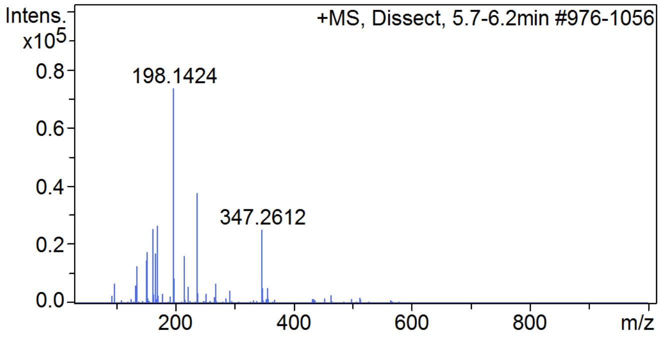

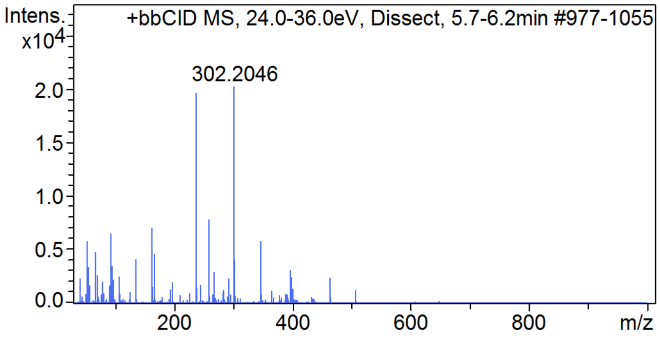

#	m/z	Res.	S/N	I	I %	FWHM
1	135.1124	24322	173.2	12752	17.3	0.0056
2	151.1068	25987	200.1	14730	20.0	0.0058
3	153.1223	26222	240.2	17689	24.0	0.0058
4	163.0336	26412	345.2	25419	34.5	0.0062
5	167.9880	28015	234.2	17243	23.4	0.0060
6	171.1323	27585	361.0	26579	36.1	0.0062
7	198.1424	29691	999.7	73603	100.0	0.0067
8	216.1525	29467	220.6	16245	22.1	0.0073
9	238.1338	30931	514.0	37843	51.4	0.0077
10	347.2612	33680	344.8	25389	34.5	0.0103

#	m/z	Res.	S/N	I	I %	FWHM
1	53.0365	16886	286.9	5807	28.7	0.0031
2	67.0519	17099	235.8	4773	23.6	0.0039
3	93.0670	19826	321.9	6515	32.2	0.0047
4	135.0093	24679	203.8	4125	20.4	0.0055
5	163.0336	26507	350.1	7086	35.0	0.0062
6	167.9881	27183	227.5	4606	22.8	0.0062
7	238.1336	31292	972.4	19682	97.3	0.0076
8	260.1148	30886	386.1	7815	38.6	0.0084
9	302.2046	32587	999.6	20234	100.0	0.0093
10	347.2609	32095	287.7	5823	28.8	0.0108

Compound Spectrum List Report

Cmpd 46, Dissect, 6.0 min

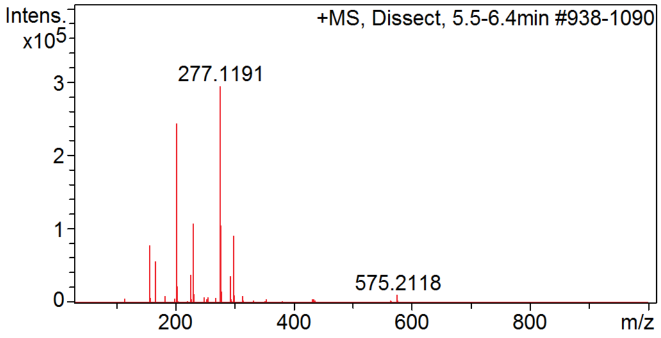

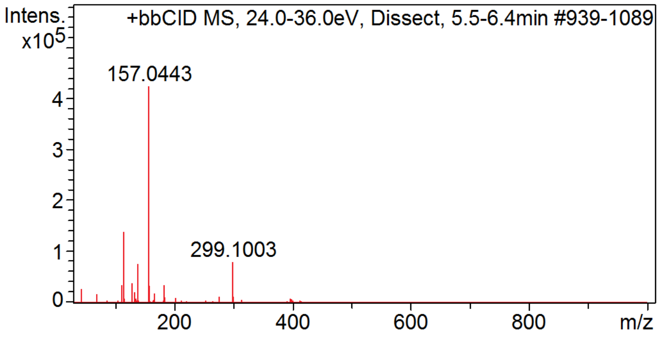

#	m/z	Res.	S/N	I	I %	FWHM
1	157.0444	27003	266.5	78739	26.8	0.0058
2	167.9881	27777	193.0	57036	19.4	0.0060
3	203.0847	31669	822.5	243008	82.6	0.0064
4	204.0882	28447	78.9	23312	7.9	0.0072
5	227.1469	30406	130.2	38459	13.1	0.0075
6	231.0788	31775	365.4	107958	36.7	0.0073
7	277.1191	36730	995.6	294161	100.0	0.0075
8	278.1227	33041	357.9	105753	36.0	0.0084
9	294.1452	32718	125.3	37032	12.6	0.0090
10	299.1004	33974	308.8	91226	31.0	0.0088

#	m/z	Res.	S/N	I	I %	FWHM
1	43.0161	15725	66.5	28399	6.7	0.0027
2	111.0040	23051	84.1	35925	8.5	0.0048
3	115.0351	24085	324.6	138682	32.7	0.0048
4	129.0140	24484	91.9	39260	9.3	0.0053
5	133.0455	22949	49.9	21319	5.0	0.0058
6	138.9980	26246	178.4	76226	18.0	0.0053
7	157.0443	29115	991.5	423617	100.0	0.0054
8	158.0477	27016	80.7	34462	8.1	0.0059
9	184.0934	28620	84.5	36101	8.5	0.0064
10	299.1003	32645	187.9	80265	18.9	0.0092

Cmpd 47, Dissect, 6.1 min

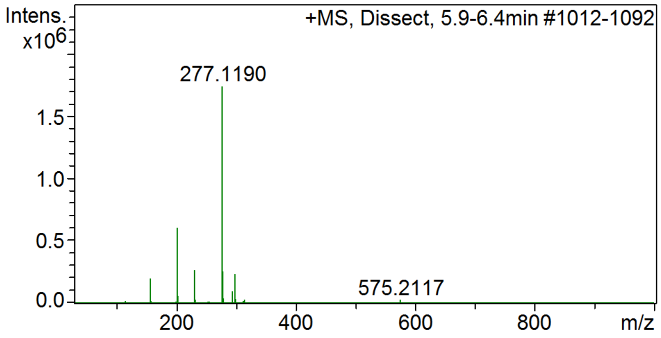

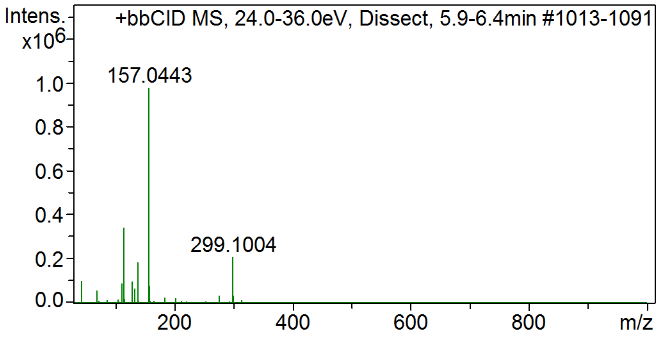

#	m/z	Res.	S/N	I	I %	FWHM
1	157.0444	27173	108.0	203257	11.7	0.0058
2	203.0847	32088	323.3	608396	34.9	0.0063
3	204.0881	28520	31.2	58672	3.4	0.0072
4	231.0787	32244	144.2	271351	15.6	0.0072
5	277.1190	37747	925.1	1740808	100.0	0.0073
6	278.1226	33440	137.9	259479	14.9	0.0083
7	279.1243	28796	21.4	40209	2.3	0.0097
8	294.1452	32906	52.1	98100	5.6	0.0089
9	299.1004	33929	125.7	236546	13.6	0.0088
10	300.1038	31913	16.5	31016	1.8	0.0094

#	m/z	Res.	S/N	I	I %	FWHM
1	43.0161	15935	103.8	102455	10.5	0.0027
2	68.9946	18247	60.3	59587	6.1	0.0038
3	111.0040	22955	92.7	91540	9.4	0.0048
4	115.0351	24218	348.5	344081	35.2	0.0047
5	129.0140	24558	101.0	99696	10.2	0.0053
6	133.0455	22998	70.1	69247	7.1	0.0058
7	138.9980	26337	189.4	186962	19.1	0.0053
8	157.0443	29362	989.1	976597	100.0	0.0053
9	158.0477	27161	82.1	81029	8.3	0.0058
10	299.1004	32726	211.4	208681	21.4	0.0091

Compound Spectrum List Report

Cmpd 48, Dissect, 6.2 min

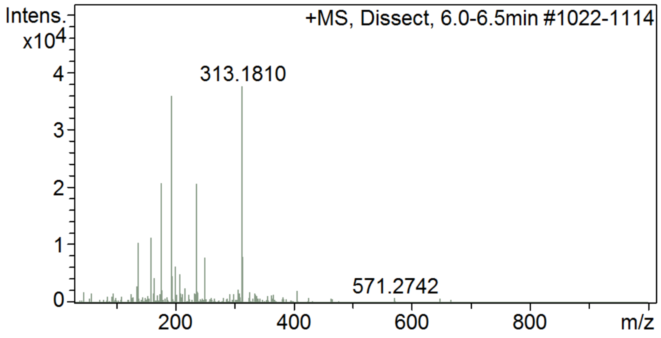

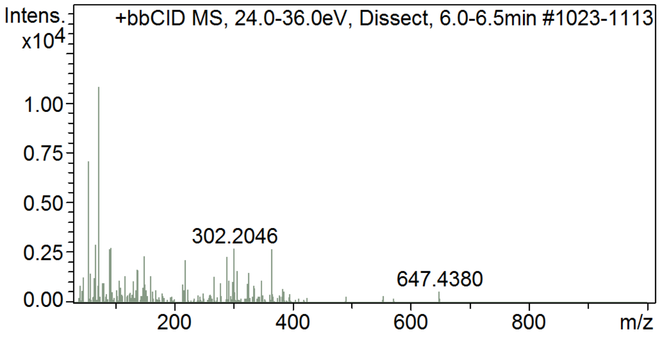

#	m/z	Res.	S/N	I	I %	FWHM
1	137.0916	24960	277.8	10447	27.8	0.0055
2	159.1115	26420	300.6	11306	30.1	0.0060
3	177.1214	27615	551.5	20741	55.2	0.0064
4	195.0952	28507	494.5	18597	49.5	0.0068
5	195.1315	28697	951.8	35791	95.3	0.0068
6	237.1409	29632	549.7	20671	55.0	0.0080
7	251.1560	29910	209.4	7875	21.0	0.0084
8	313.1810	33185	998.7	37556	100.0	0.0094
9	313.2386	31088	327.5	12316	32.8	0.0101
10	314.1843	31458	214.2	8055	21.4	0.0100

#	m/z	Res.	S/N	I	I %	FWHM
1	55.0521	17091	654.2	7082	65.4	0.0032
2	67.0519	17806	271.2	2936	27.1	0.0038
3	72.0782	18253	999.7	10823	100.0	0.0039
4	91.0514	19531	250.5	2712	25.1	0.0047
5	93.0670	19094	255.1	2762	25.5	0.0049
6	149.0547	25791	217.0	2349	21.7	0.0058
7	219.1672	30571	200.0	2165	20.0	0.0072
8	290.2019	29198	213.1	2307	21.3	0.0099
9	302.2046	32053	252.7	2736	25.3	0.0094
10	365.0934	32480	249.0	2696	24.9	0.0112

Cmpd 49, Dissect, 6.4 min

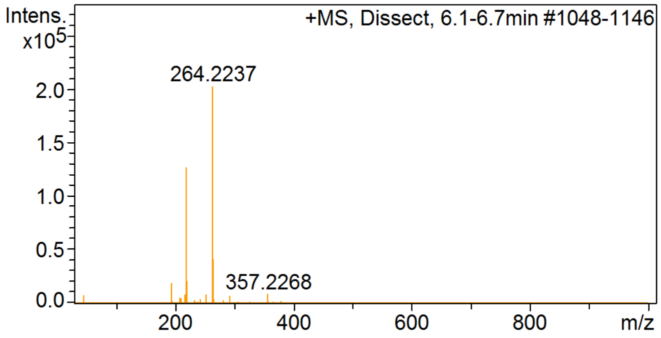

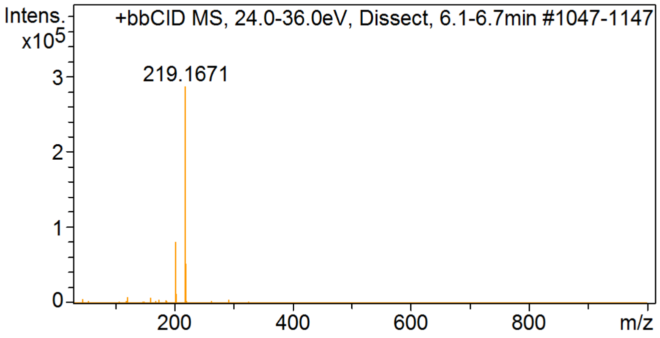

#	m/z	Res.	S/N	I	I %	FWHM
1	46.0632	15867	36.7	7422	3.7	0.0029
2	195.0952	28994	96.5	19539	9.7	0.0067
3	195.1313	27561	40.2	8141	4.0	0.0071
4	217.0764	29220	40.8	8264	4.1	0.0074
5	219.1673	30605	626.0	126713	62.6	0.0072
6	220.1706	29323	103.7	20987	10.4	0.0075
7	253.1717	29313	38.6	7815	3.9	0.0086
8	264.2237	31244	999.6	202358	100.0	0.0085
9	265.2271	31941	206.2	41742	20.6	0.0083
10	357.2268	31117	43.5	8804	4.4	0.0115

#	m/z	Res.	S/N	I	I %	FWHM
1	46.0632	15960	19.2	5504	1.9	0.0029
2	121.0971	23745	26.5	7611	2.7	0.0051
3	161.0906	26663	16.5	4726	1.6	0.0060
4	161.1270	26833	25.6	7343	2.6	0.0060
5	175.1422	27333	14.9	4264	1.5	0.0064
6	203.1363	30253	284.1	81555	28.4	0.0067
7	204.1396	27598	41.9	12018	4.2	0.0074
8	219.1671	30640	999.2	286810	100.0	0.0072
9	220.1705	30700	184.1	52848	18.4	0.0072
10	293.1627	31245	15.8	4536	1.6	0.0094

Compound Spectrum List Report

Cmpd 50, Dissect, 6.4 min

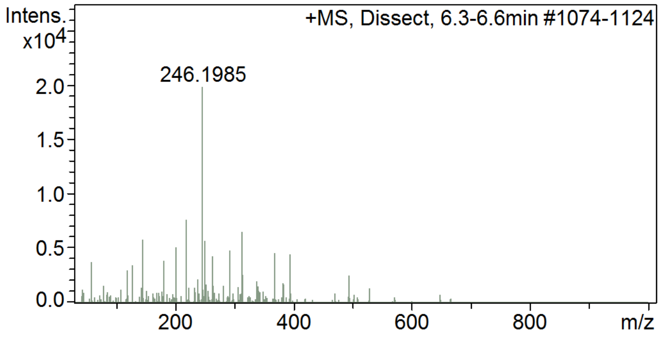

#	m/z	Res.	S/N	I	I %	FWHM
1	145.0599	25174	294.5	5843	29.5	0.0058
2	202.0797	28806	257.6	5111	25.8	0.0070
3	219.1673	30563	386.2	7662	38.6	0.0072
4	246.1985	30650	999.9	19838	100.0	0.0080
5	251.1565	29253	288.9	5733	28.9	0.0086
6	293.1633	31358	243.7	4835	24.4	0.0093
7	313.1808	27203	248.2	4924	24.8	0.0115
8	313.2398	28104	328.3	6513	32.8	0.0111
9	368.2284	31498	231.9	4601	23.2	0.0117
10	394.1944	29230	226.9	4503	22.7	0.0135

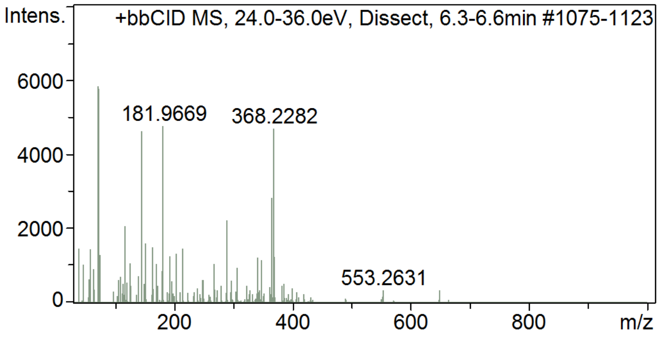

#	m/z	Res.	S/N	I	I %	FWHM
1	71.9494	18220	400.5	2339	40.1	0.0039
2	72.0418	18784	999.3	5836	100.0	0.0038
3	72.0782	18328	988.1	5771	98.9	0.0039
4	117.0660	22873	354.6	2071	35.5	0.0051
5	145.0599	25727	791.5	4623	79.2	0.0056
6	145.0962	25690	550.4	3214	55.1	0.0056
7	181.9669	27962	815.4	4762	81.6	0.0065
8	290.2019	29529	381.0	2225	38.1	0.0098
9	365.0934	31395	484.3	2829	48.5	0.0116
10	368.2282	31137	804.4	4698	80.5	0.0118

Cmpd 51, Dissect, 6.6 min

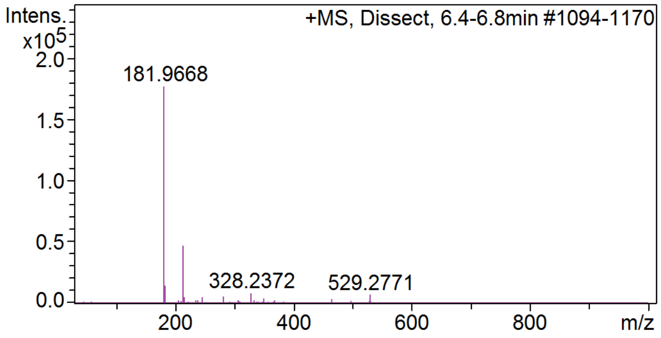

#	m/z	Res.	S/N	I	I %	FWHM
1	181.9668	29115	995.6	176982	100.0	0.0062
2	182.9696	24838	78.4	13934	7.9	0.0074
3	183.9627	27463	84.0	14927	8.4	0.0067
4	213.9920	30732	148.7	26435	14.9	0.0070
5	214.0826	31827	266.5	47371	26.8	0.0067
6	215.9877	28594	27.1	4810	2.7	0.0076
7	246.1983	29628	28.2	5016	2.8	0.0083
8	282.1966	30271	30.8	5469	3.1	0.0093
9	328.2372	31756	47.7	8487	4.8	0.0103
10	529.2771	31576	40.4	7182	4.1	0.0168

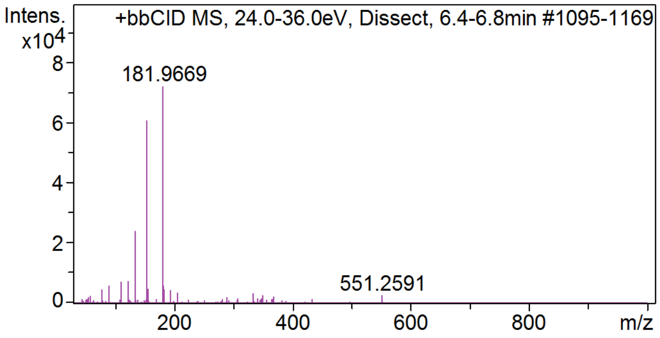

#	m/z	Res.	S/N	I	I %	FWHM
1	78.0312	18522	65.0	4695	6.5	0.0042
2	90.0311	19262	83.5	6037	8.4	0.0047
3	110.0022	22334	102.6	7415	10.3	0.0049
4	122.0019	23321	104.4	7544	10.5	0.0052
5	134.0014	25033	332.5	24022	33.4	0.0054
6	153.9728	26577	838.7	60597	84.2	0.0058
7	155.9686	26163	69.5	5024	7.0	0.0060
8	181.9669	28542	996.3	71985	100.0	0.0064
9	182.9700	23186	84.7	6118	8.5	0.0079
10	183.9628	27110	67.5	4878	6.8	0.0068

Compound Spectrum List Report

Cmpd 52, Dissect, 6.8 min

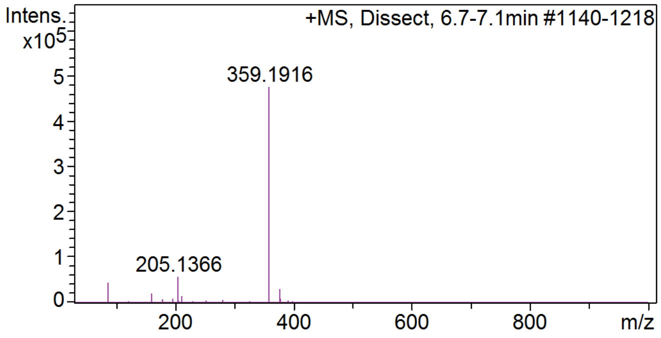

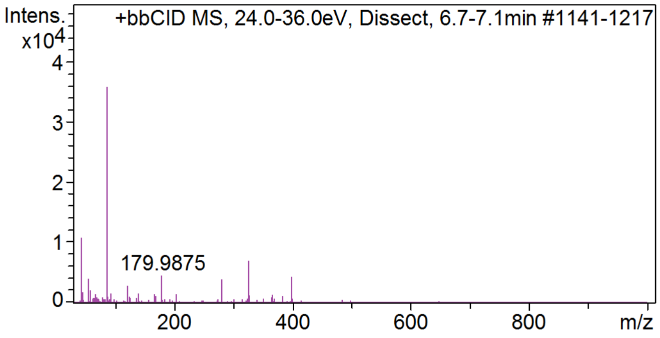

#	m/z	Res.	S/N	I	I %	FWHM
1	87.0411	19754	93.4	45617	9.6	0.0044
2	161.1271	26330	46.0	22455	4.7	0.0061
3	179.1370	26576	15.7	7662	1.6	0.0067
4	197.1469	27968	18.7	9159	1.9	0.0070
5	205.1366	29142	119.2	58250	12.2	0.0070
6	212.1575	28340	29.8	14534	3.1	0.0075
7	281.1056	29463	13.0	6327	1.3	0.0095
8	359.1916	40492	975.4	476466	100.0	0.0089
9	377.2029	30075	65.5	31995	6.7	0.0125
10	378.2059	36351	18.4	9008	1.9	0.0104

#	m/z	Res.	S/N	I	I %	FWHM
1	43.0160	15593	302.2	10848	30.3	0.0028
2	43.0525	15324	54.3	1951	5.4	0.0028
3	55.0520	17076	113.7	4082	11.4	0.0032
4	58.0629	17367	60.5	2173	6.1	0.0033
5	87.0411	19562	998.2	35838	100.0	0.0044
6	121.0973	22476	81.3	2920	8.1	0.0054
7	179.9875	28371	127.0	4560	12.7	0.0063
8	281.1056	30091	111.9	4017	11.2	0.0093
9	326.2191	30619	194.9	6998	19.5	0.0107
10	399.1649	31298	123.5	4432	12.4	0.0128

Cmpd 53, Dissect, 6.9 min

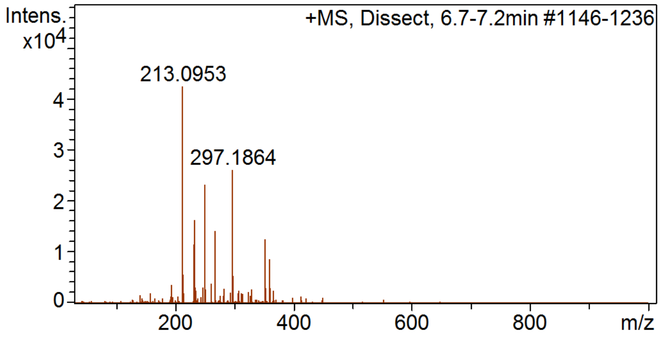

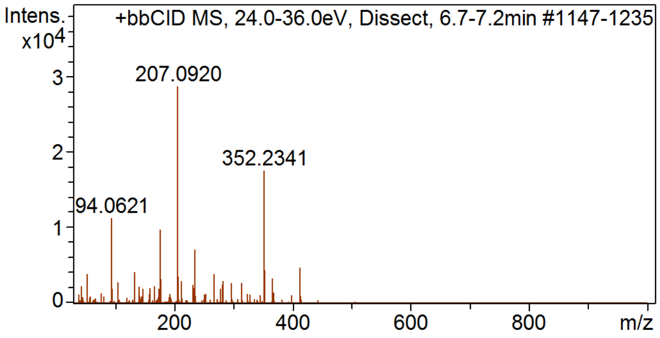

#	m/z	Res.	S/N	I	I %	FWHM
1	213.0953	29846	999.8	42418	100.0	0.0071
2	214.1731	28738	133.4	5658	13.3	0.0075
3	232.1619	29531	272.4	11556	27.2	0.0079
4	233.1459	29961	385.5	16356	38.6	0.0078
5	251.1560	31525	546.9	23201	54.7	0.0080
6	268.1805	30744	334.1	14175	33.4	0.0087
7	297.1864	31923	616.0	26134	61.6	0.0093
8	298.1898	29116	127.9	5424	12.8	0.0102
9	352.2341	32402	297.3	12614	29.7	0.0109
10	360.1948	29507	205.2	8706	20.5	0.0122

#	m/z	Res.	S/N	I	I %	FWHM
1	53.0364	16686	137.7	3950	13.8	0.0032
2	94.0621	19774	392.2	11252	39.2	0.0048
3	133.0967	24134	145.9	4187	14.6	0.0055
4	177.0825	28503	339.2	9732	33.9	0.0062
5	207.0920	30132	999.2	28668	100.0	0.0069
6	236.1545	30489	248.5	7129	24.9	0.0077
7	268.1797	28774	138.0	3960	13.8	0.0093
8	352.2341	32178	611.2	17536	61.2	0.0109
9	353.2375	31510	155.6	4464	15.6	0.0112
10	413.1991	27492	167.1	4793	16.7	0.0150

Compound Spectrum List Report

Cmpd 54, Dissect, 7.2 min

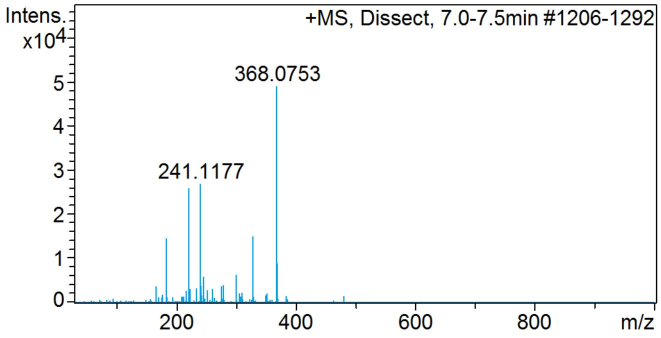

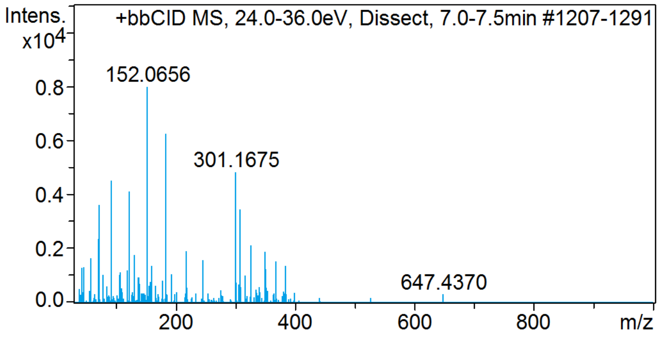

#	m/z	Res.	S/N	I	I %	FWHM
1	184.1634	28029	299.7	14693	30.0	0.0066
2	222.1779	30436	530.1	25995	53.0	0.0073
3	241.1177	30146	548.7	26904	54.9	0.0080
4	246.2346	30355	122.0	5984	12.2	0.0081
5	279.1864	28920	84.8	4157	8.5	0.0097
6	301.1678	29107	132.5	6495	13.3	0.0103
7	329.1575	32186	308.7	15136	30.9	0.0102
8	368.0753	34144	999.6	49016	100.0	0.0108
9	368.2285	31054	189.5	9292	19.0	0.0119
10	369.0784	29970	184.6	9051	18.5	0.0123

#	m/z	Res.	S/N	I	I %	FWHM
1	71.0466	18351	298.2	2380	29.8	0.0039
2	72.0782	18239	454.5	3627	45.5	0.0040
3	92.0466	19518	565.2	4511	56.5	0.0047
4	122.0560	22794	516.3	4120	51.6	0.0054
5	152.0656	26124	999.8	7979	100.0	0.0058
6	184.0366	27850	781.3	6235	78.1	0.0066
7	184.1636	27769	278.1	2219	27.8	0.0066
8	301.1675	29447	603.6	4817	60.4	0.0102
9	308.2098	30490	434.0	3463	43.4	0.0101
10	326.2194	29985	266.8	2129	26.7	0.0109

Cmpd 55, Dissect, 7.3 min

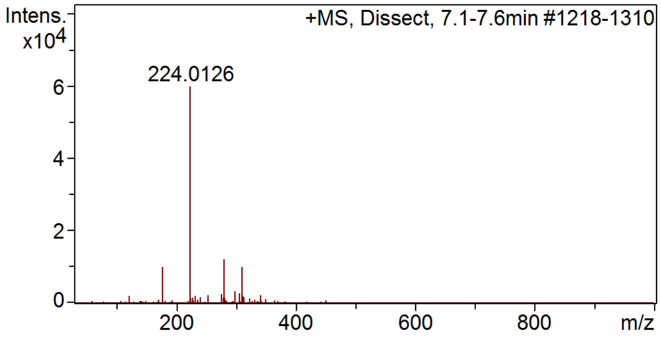

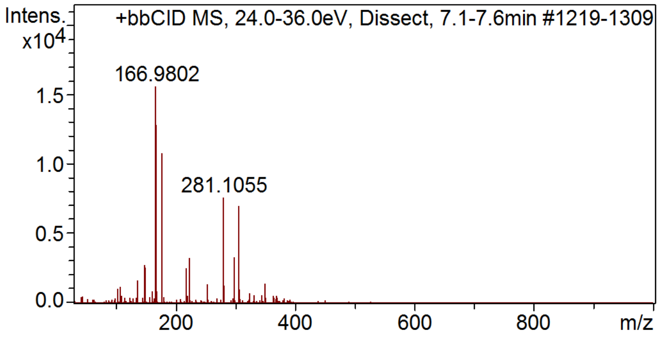

#	m/z	Res.	S/N	I	I %	FWHM
1	178.1167	27219	170.7	10223	17.1	0.0065
2	224.0126	30585	998.5	59810	100.0	0.0073
3	232.1832	29269	34.5	2065	3.5	0.0079
4	254.1647	28345	37.9	2270	3.8	0.0090
5	276.0059	29253	46.1	2760	4.6	0.0094
6	281.1057	31439	204.9	12275	20.5	0.0089
7	298.9113	31119	56.9	3407	5.7	0.0096
8	306.1944	31065	47.8	2863	4.8	0.0099
9	310.2276	32773	168.7	10102	16.9	0.0095
10	341.2471	29023	36.9	2212	3.7	0.0118

#	m/z	Res.	S/N	I	I %	FWHM
1	148.0167	26182	177.3	2765	17.7	0.0057
2	149.0185	25706	166.5	2597	16.7	0.0058
3	166.9802	26978	999.1	15586	100.0	0.0062
4	167.9879	26276	820.2	12796	82.1	0.0064
5	178.1167	27095	690.5	10772	69.1	0.0066
6	219.1670	30404	164.3	2564	16.4	0.0072
7	224.0125	30371	211.9	3305	21.2	0.0074
8	281.1055	31530	487.4	7603	48.8	0.0089
9	298.9114	31265	214.5	3346	21.5	0.0096
10	306.1940	30909	449.7	7016	45.0	0.0099

Compound Spectrum List Report

Cmpd 56, Dissect, 7.4 min

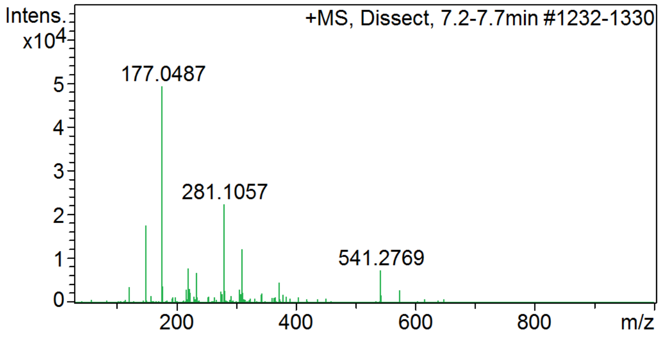

#	m/z	Res.	S/N	I	I %	FWHM
1	121.0608	23212	76.0	3745	7.6	0.0052
2	149.0184	25588	358.5	17671	35.9	0.0058
3	177.0487	27778	999.7	49281	100.0	0.0064
4	178.1166	27050	78.5	3872	7.9	0.0066
5	221.0671	29076	160.0	7885	16.0	0.0076
6	235.1616	29985	138.6	6834	13.9	0.0078
7	281.1057	32356	455.0	22431	45.5	0.0087
8	310.2274	33368	250.4	12344	25.0	0.0093
9	373.2071	30243	96.8	4773	9.7	0.0123
10	541.2769	31650	150.5	7420	15.1	0.0171

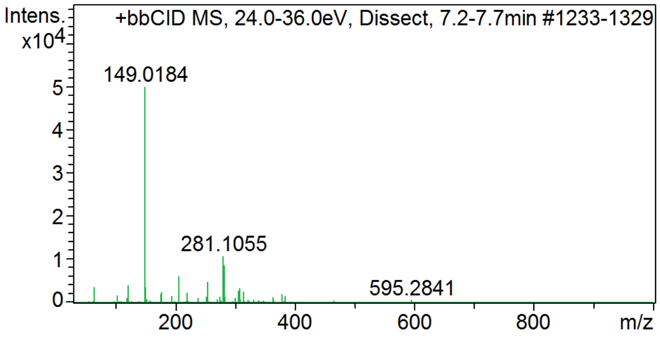

#	m/z	Res.	S/N	I	I %	FWHM
1	65.0363	16971	76.2	3811	7.6	0.0038
2	121.0244	23783	83.4	4170	8.3	0.0051
3	149.0184	26487	999.5	49970	100.0	0.0056
4	150.0217	25113	74.4	3717	7.4	0.0060
5	207.0921	28734	124.2	6209	12.4	0.0072
6	255.0554	30149	99.0	4951	9.9	0.0085
7	281.1055	31113	218.0	10901	21.8	0.0090
8	283.1420	32355	176.3	8815	17.6	0.0088
9	306.1939	30746	57.9	2894	5.8	0.0100
10	308.2094	30922	69.9	3494	7.0	0.0100

Cmpd 57, Dissect, 7.6 min

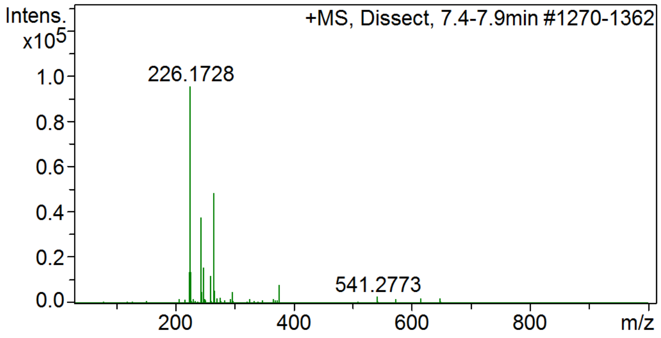

#	m/z	Res.	S/N	I	I %	FWHM
1	225.1313	29790	145.1	14017	14.7	0.0076
2	226.0282	29994	74.0	7149	7.5	0.0075
3	226.1348	28249	111.0	10722	11.2	0.0080
4	226.1728	31280	987.4	95407	100.0	0.0072
5	227.1760	29099	144.7	13977	14.6	0.0078
6	244.1828	31086	391.6	37841	39.7	0.0079
7	249.1402	31070	163.6	15810	16.6	0.0080
8	260.2134	31581	125.9	12162	12.7	0.0082
9	266.1640	32184	500.7	48380	50.7	0.0083
10	376.1837	32698	84.4	8157	8.5	0.0115

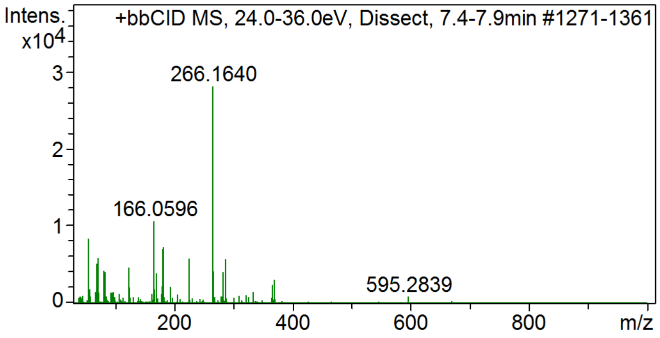

#	m/z	Res.	S/N	I	I %	FWHM
1	55.0520	17102	297.9	8377	29.8	0.0032
2	69.0674	17968	185.3	5212	18.6	0.0038
3	72.0418	18213	210.8	5927	21.1	0.0040
4	166.0596	27068	379.9	10684	38.0	0.0061
5	167.0666	24412	185.7	5221	18.6	0.0068
6	182.0777	28153	252.7	7105	25.3	0.0065
7	183.0853	25361	262.0	7369	26.2	0.0072
8	226.1726	31548	208.8	5873	20.9	0.0072
9	266.1640	31867	999.1	28096	100.0	0.0084
10	288.1452	30421	205.0	5766	20.5	0.0095

Compound Spectrum List Report

Cmpd 58, Dissect, 7.7 min

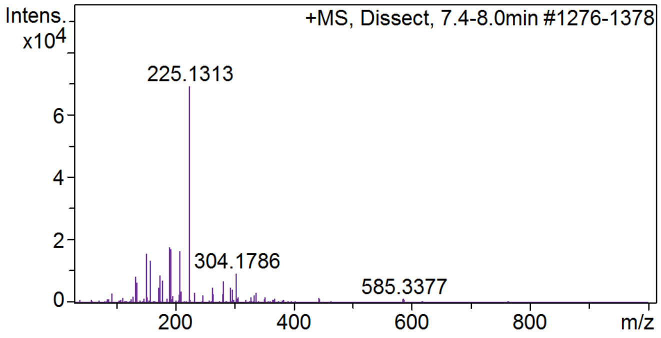

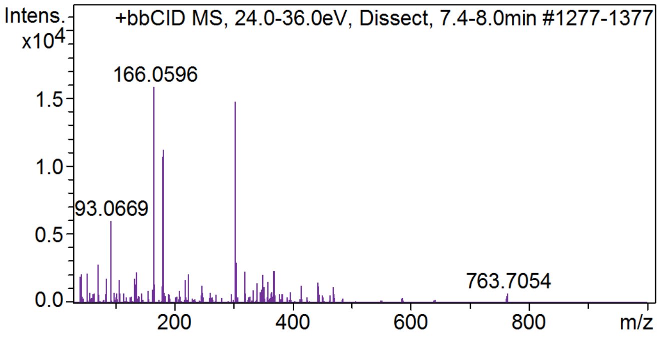

#	m/z	Res.	S/N	I	I %	FWHM
1	133.0968	23562	123.0	8509	12.3	0.0056
2	151.1067	26014	228.6	15812	22.9	0.0058
3	158.1486	26236	195.7	13539	19.6	0.0060
4	175.1423	26242	126.6	8761	12.7	0.0067
5	179.0581	24650	105.4	7292	10.6	0.0073
6	191.1367	28679	256.2	17723	25.7	0.0067
7	193.1524	28684	248.5	17190	24.9	0.0067
8	209.1467	27901	238.7	16510	23.9	0.0075
9	225.1313	29948	998.6	69077	100.0	0.0075
10	304.1786	28672	136.0	9406	13.6	0.0106

#	m/z	Res.	S/N	I	I %	FWHM
1	71.0466	18420	177.7	2813	17.8	0.0039
2	93.0669	19181	380.7	6026	38.1	0.0049
3	166.0596	27100	999.9	15826	100.0	0.0061
4	167.0666	24377	791.5	12528	79.2	0.0069
5	182.0777	27899	674.1	10670	67.4	0.0065
6	183.0853	25484	706.6	11185	70.7	0.0072
7	304.1784	30247	929.3	14709	92.9	0.0101
8	305.1816	30241	166.0	2628	16.6	0.0101
9	306.1939	30053	188.4	2982	18.8	0.0102
10	370.2438	31253	149.6	2369	15.0	0.0118

Cmpd 59, Dissect, 7.8 min

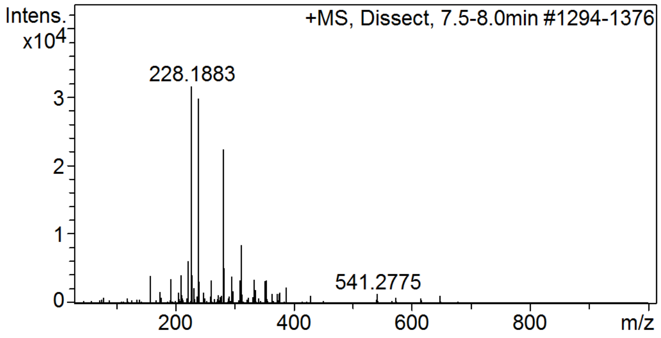

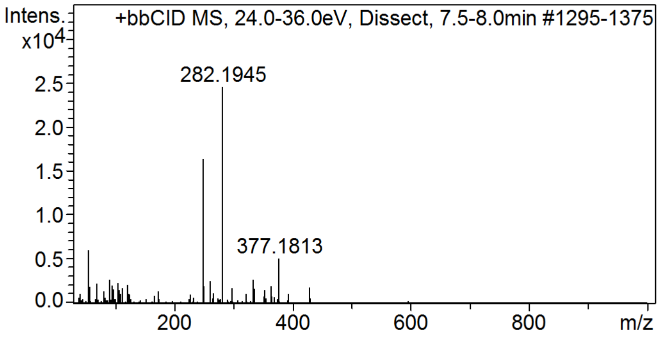

#	m/z	Res.	S/N	I	I %	FWHM
1	158.1486	26296	126.6	4006	12.7	0.0060
2	211.1621	29045	129.9	4110	13.0	0.0073
3	223.1618	28889	195.9	6200	19.7	0.0077
4	228.1883	30366	996.5	31533	100.0	0.0075
5	229.1916	28501	130.6	4133	13.1	0.0080
6	240.1880	31306	937.5	29666	94.1	0.0077
7	282.1953	32329	707.4	22384	71.0	0.0087
8	283.1984	29617	164.1	5194	16.5	0.0096
9	296.2487	29650	125.4	3968	12.6	0.0100
10	312.2429	32363	269.3	8521	27.0	0.0096

#	m/z	Res.	S/N	I	I %	FWHM
1	55.0520	17110	247.4	6085	24.8	0.0032
2	69.0673	17921	93.3	2294	9.3	0.0039
3	91.0513	19715	110.2	2710	11.0	0.0046
4	105.0664	21404	97.0	2385	9.7	0.0049
5	121.0973	22797	87.7	2157	8.8	0.0053
6	250.1696	31590	664.6	16344	66.5	0.0079
7	262.1693	29660	105.6	2596	10.6	0.0088
8	282.1945	32552	999.0	24568	100.0	0.0087
9	334.2240	31621	109.5	2693	11.0	0.0106
10	377.1813	29865	207.1	5094	20.7	0.0126

Compound Spectrum List Report

Cmpd 60, Dissect, 7.9 min

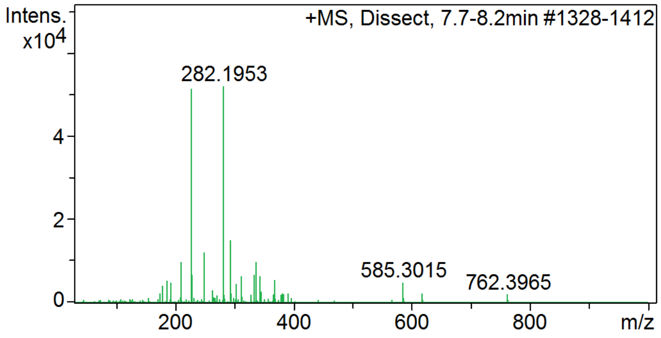

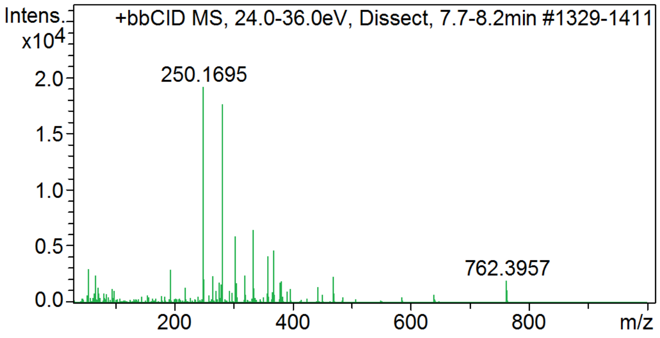

#	m/z	Res.	S/N	I	I %	FWHM
1	211.1622	29203	188.6	9806	18.9	0.0072
2	228.1883	30692	987.7	51369	98.9	0.0074
3	229.1916	29096	131.4	6832	13.2	0.0079
4	250.1696	30494	233.1	12123	23.3	0.0082
5	282.1953	32435	998.8	51945	100.0	0.0087
6	294.2331	32101	289.2	15041	29.0	0.0092
7	312.2429	32207	126.4	6574	12.7	0.0097
8	334.2240	31541	131.6	6844	13.2	0.0106
9	337.0929	31330	188.9	9822	18.9	0.0108
10	344.2314	29811	124.7	6488	12.5	0.0115

#	m/z	Res.	S/N	I	I %	FWHM
1	55.0520	17209	159.0	3048	15.9	0.0032
2	67.0519	17520	129.4	2480	12.9	0.0038
3	195.0927	26899	154.6	2964	15.5	0.0073
4	250.1695	31574	999.7	19159	100.0	0.0079
5	282.1945	32519	918.0	17595	91.8	0.0087
6	304.1783	30162	308.2	5906	30.8	0.0101
7	320.0673	29491	127.7	2447	12.8	0.0109
8	334.2241	31329	339.0	6498	33.9	0.0107
9	359.0737	31456	216.7	4153	21.7	0.0114
10	368.2283	31583	242.5	4648	24.3	0.0117

Cmpd 61, Dissect, 8.1 min

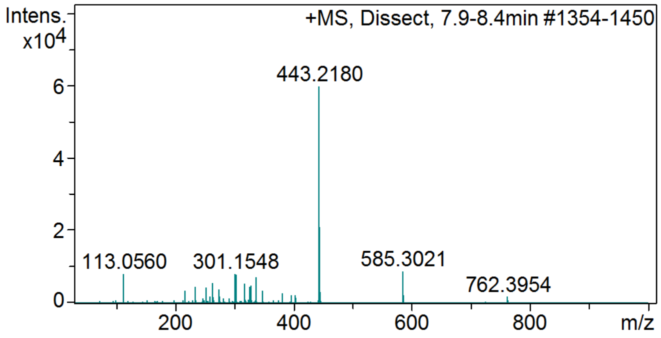

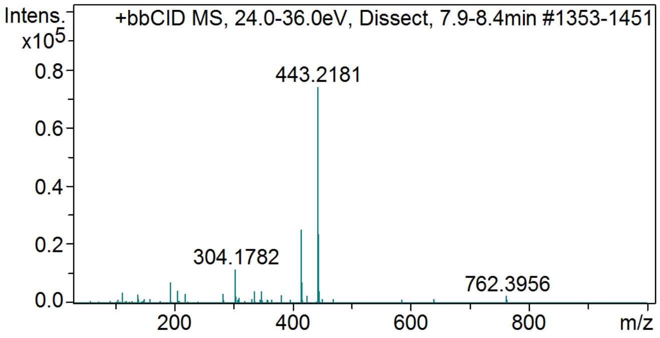

#	m/z	Res.	S/N	I	I %	FWHM
1	113.0560	22645	137.0	8204	13.7	0.0050
2	264.1857	29243	96.2	5761	9.6	0.0090
3	301.1548	32543	138.0	8261	13.8	0.0093
4	304.1785	29488	133.4	7988	13.4	0.0103
5	318.1806	31104	92.6	5543	9.3	0.0102
6	328.2376	32013	85.0	5088	8.5	0.0103
7	337.0927	31098	121.6	7281	12.2	0.0108
8	443.2180	32811	998.8	59798	100.0	0.0135
9	444.2212	31467	353.0	21132	35.3	0.0141
10	585.3021	30377	148.9	8913	14.9	0.0193

#	m/z	Res.	S/N	I	I %	FWHM
1	195.0925	27535	100.1	7422	10.0	0.0071
2	207.0922	27622	60.1	4454	6.0	0.0075
3	304.1782	31784	158.4	11739	15.8	0.0096
4	336.2397	31831	56.2	4163	5.6	0.0106
5	348.2031	30395	59.0	4370	5.9	0.0115
6	415.1877	31418	339.8	25180	34.0	0.0132
7	416.1910	30876	99.2	7355	9.9	0.0135
8	443.2181	33804	999.6	74079	100.0	0.0131
9	444.2214	32676	319.2	23654	31.9	0.0136
10	445.2239	30704	56.7	4202	5.7	0.0145

Compound Spectrum List Report

Cmpd 62, Dissect, 8.1 min

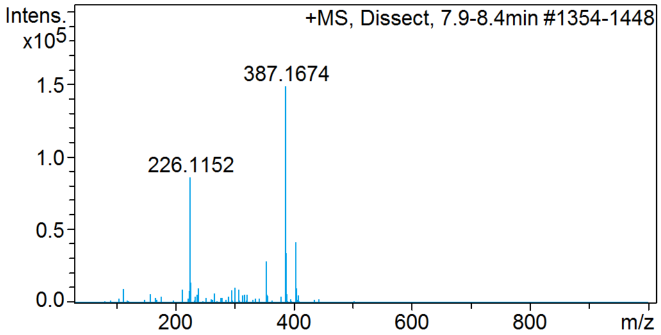

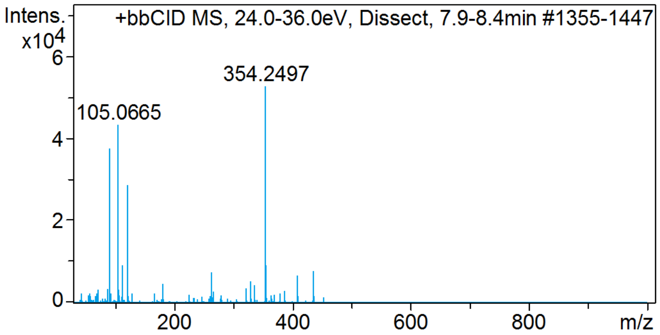

#	m/z	Res.	S/N	I	I %	FWHM
1	113.0560	22614	64.9	9666	6.5	0.0050
2	226.1152	30720	578.5	86164	58.0	0.0074
3	227.1188	29148	94.8	14123	9.5	0.0078
4	240.1879	30438	68.5	10197	6.9	0.0079
5	301.1547	32554	73.1	10890	7.3	0.0093
6	354.2497	32369	191.7	28555	19.2	0.0109
7	387.1674	35312	997.3	148533	100.0	0.0110
8	388.1708	32886	231.2	34431	23.2	0.0118
9	404.1933	33577	279.9	41681	28.1	0.0120
10	405.1965	30573	69.3	10318	6.9	0.0133

#	m/z	Res.	S/N	I	I %	FWHM
1	91.0514	19944	711.5	37501	71.2	0.0046
2	105.0665	21470	821.1	43282	82.2	0.0049
3	113.0561	22331	174.1	9177	17.4	0.0051
4	121.0609	23318	542.7	28605	54.3	0.0052
5	121.0974	22688	124.8	6577	12.5	0.0053
6	264.1848	30292	143.3	7553	14.3	0.0087
7	354.2497	33974	999.6	52686	100.0	0.0104
8	355.2529	30334	173.9	9167	17.4	0.0117
9	409.1487	30919	127.2	6703	12.7	0.0132
10	435.2204	30149	148.7	7839	14.9	0.0144

Cmpd 63, Dissect, 8.3 min

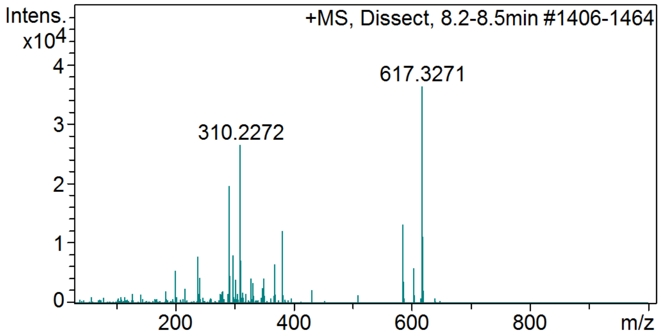

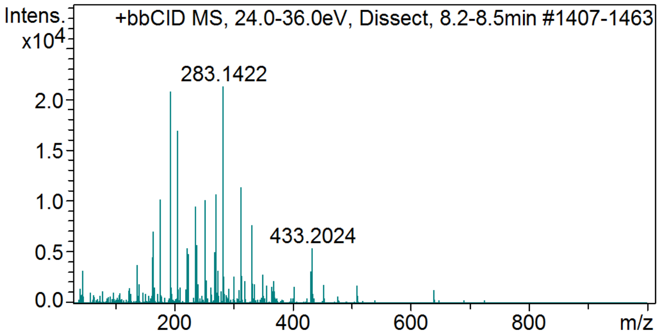

#	m/z	Res.	S/N	I	I %	FWHM
1	239.1179	31217	215.2	7818	21.5	0.0077
2	292.2176	31710	541.2	19665	54.1	0.0092
3	298.2281	31096	222.3	8076	22.2	0.0096
4	310.2272	33178	729.0	26487	72.9	0.0094
5	311.2306	30112	198.9	7228	19.9	0.0103
6	368.2289	32031	180.1	6543	18.0	0.0115
7	382.0475	32140	333.2	12108	33.3	0.0119
8	585.3019	29999	365.0	13261	36.5	0.0195
9	617.3271	32099	999.9	36330	100.0	0.0192
10	618.3299	30419	306.6	11141	30.7	0.0203

#	m/z	Res.	S/N	I	I %	FWHM
1	165.0834	25168	330.7	7028	33.1	0.0066
2	177.0826	26435	479.1	10182	47.9	0.0067
3	195.0925	28713	976.6	20756	97.7	0.0068
4	207.0921	28454	793.5	16864	79.4	0.0073
5	237.1019	29447	445.8	9475	44.6	0.0081
6	253.1325	30147	477.6	10150	47.8	0.0084
7	271.1425	29969	502.2	10674	50.2	0.0090
8	283.1422	31546	999.7	21247	100.0	0.0090
9	313.1517	31095	534.6	11362	53.5	0.0101
10	332.2086	31032	360.2	7654	36.0	0.0107

Compound Spectrum List Report

Cmpd 64, Dissect, 8.4 min

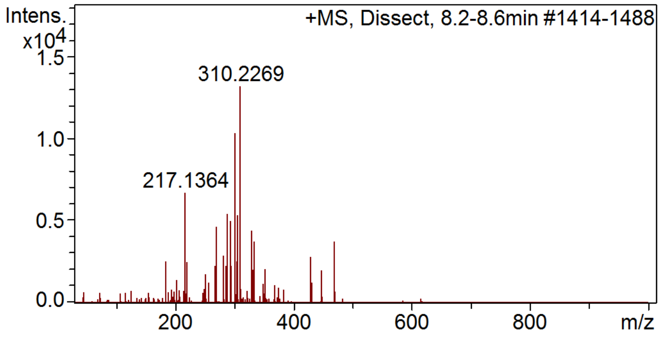

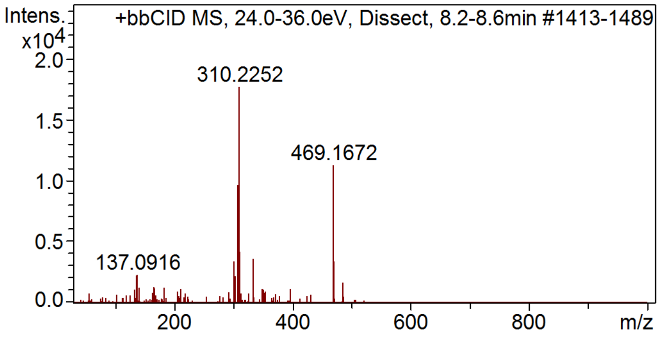

#	m/z	Res.	S/N	I	I %	FWHM
1	217.1364	28378	508.2	6701	50.9	0.0077
2	270.2341	29838	351.8	4639	35.2	0.0091
3	288.2437	29249	410.4	5412	41.1	0.0099
4	294.2330	31583	378.0	4984	37.8	0.0093
5	302.0204	30909	782.4	10316	78.3	0.0098
6	306.0517	31459	404.5	5334	40.5	0.0097
7	310.2269	32952	999.1	13174	100.0	0.0094
8	330.2531	32152	334.2	4407	33.4	0.0103
9	334.2242	31631	283.2	3734	28.3	0.0106
10	469.1672	31353	284.6	3753	28.5	0.0150

#	m/z	Res.	S/N	I	I %	FWHM
1	136.0472	23846	128.4	2274	12.8	0.0057
2	137.0916	24828	130.9	2318	13.1	0.0055
3	302.0206	31341	195.0	3455	19.5	0.0096
4	304.1783	30331	126.6	2243	12.7	0.0100
5	308.2096	31239	546.2	9675	54.6	0.0099
6	310.2252	31081	999.5	17704	100.0	0.0100
7	311.2281	31124	239.1	4234	23.9	0.0100
8	334.2240	31813	204.5	3623	20.5	0.0105
9	469.1672	31307	635.7	11259	63.6	0.0150
10	470.1705	29505	193.1	3420	19.3	0.0159

Cmpd 65, Dissect, 8.4 min

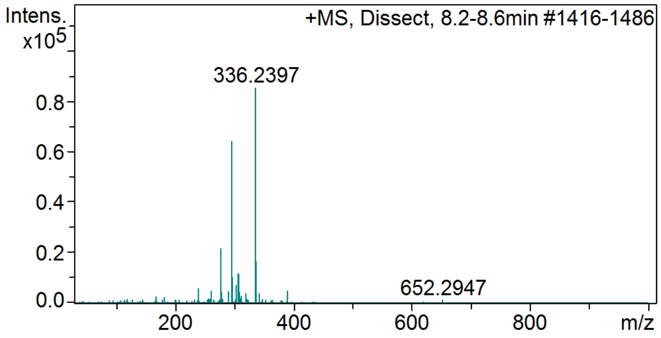

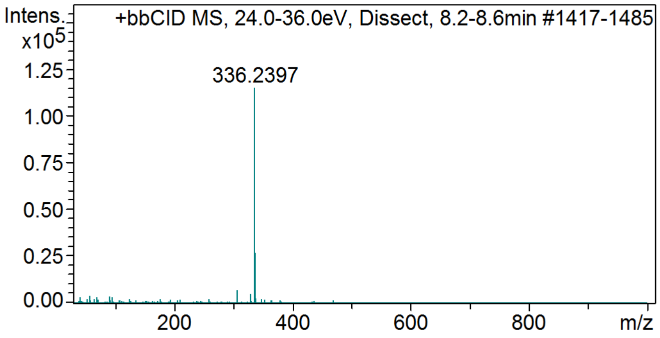

#	m/z	Res.	S/N	I	I %	FWHM
1	240.1878	30012	73.6	6298	7.4	0.0080
2	278.2388	31262	256.4	21931	25.7	0.0089
3	296.2487	32454	749.8	64145	75.0	0.0091
4	297.2518	31209	121.7	10412	12.2	0.0095
5	304.0359	30464	88.3	7552	8.8	0.0100
6	307.2166	30192	141.5	12104	14.2	0.0102
7	308.2110	30471	137.7	11778	13.8	0.0101
8	336.1044	30304	73.7	6305	7.4	0.0111
9	336.2397	33721	999.2	85479	100.0	0.0100
10	337.2429	29997	196.4	16804	19.7	0.0112

#	m/z	Res.	S/N	I	I %	FWHM
1	41.0368	15159	29.4	3388	2.9	0.0027
2	57.0677	17061	34.5	3972	3.5	0.0033
3	69.0673	17978	27.8	3210	2.8	0.0038
4	91.0514	19413	30.9	3566	3.1	0.0047
5	95.0825	19794	27.2	3133	2.7	0.0048
6	307.2169	30337	63.8	7353	6.4	0.0101
7	330.1931	30025	43.2	4981	4.3	0.0110
8	336.1043	29587	38.6	4452	3.9	0.0114
9	336.2397	33903	998.7	115130	100.0	0.0099
10	337.2428	30688	234.0	26981	23.4	0.0110

Compound Spectrum List Report

Cmpd 66, Dissect, 8.6 min

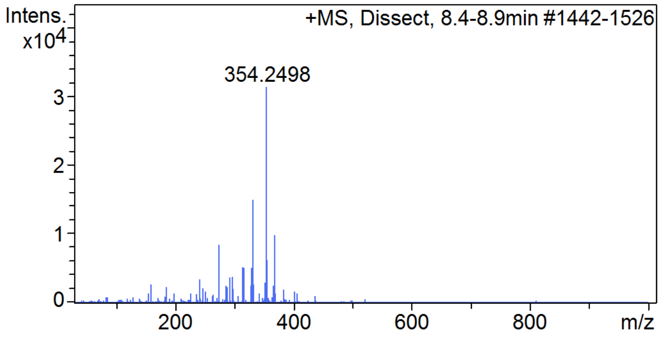

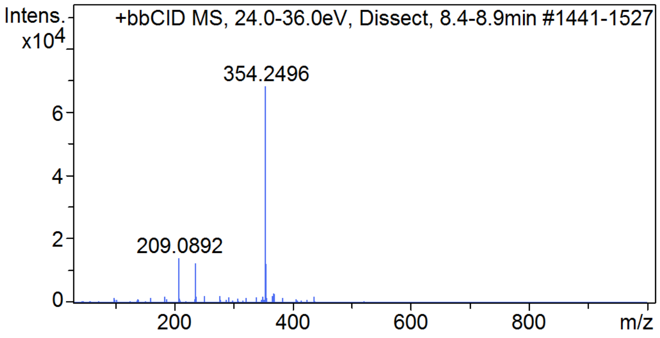

#	m/z	Res.	S/N	I	I %	FWHM
1	274.2288	30252	270.3	8501	27.1	0.0091
2	297.1025	31142	123.5	3883	12.4	0.0095
3	314.2585	30199	167.0	5253	16.7	0.0104
4	317.1620	30120	164.1	5161	16.4	0.0105
5	330.2532	32672	163.8	5151	16.4	0.0101
6	332.2087	30158	138.8	4363	13.9	0.0110
7	332.2686	32363	475.3	14947	47.6	0.0103
8	354.2498	32801	998.7	31407	100.0	0.0108
9	355.2527	28930	199.3	6267	20.0	0.0123
10	368.2287	32436	313.3	9853	31.4	0.0114

#	m/z	Res.	S/N	I	I %	FWHM
1	185.1474	28541	29.3	1994	2.9	0.0065
2	209.0892	28856	207.5	14138	20.8	0.0072
3	237.0832	30123	184.8	12592	18.5	0.0079
4	252.1125	29011	30.5	2080	3.1	0.0087
5	278.1637	30067	31.1	2122	3.1	0.0093
6	354.2496	34505	999.6	68108	100.0	0.0103
7	355.2530	29841	182.4	12430	18.3	0.0119
8	366.2124	29991	31.4	2141	3.1	0.0122
9	368.2283	32337	45.9	3128	4.6	0.0114
10	370.2233	26157	38.5	2626	3.9	0.0142

Cmpd 67, Dissect, 8.8 min

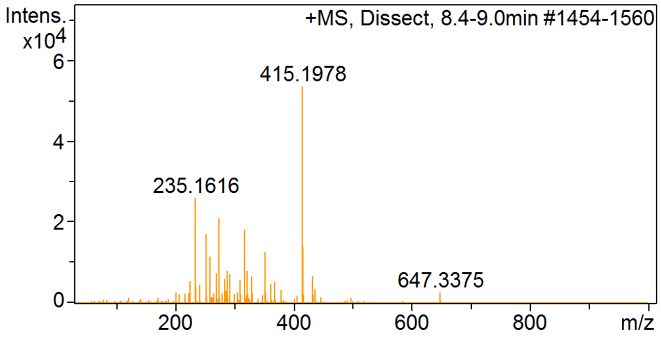

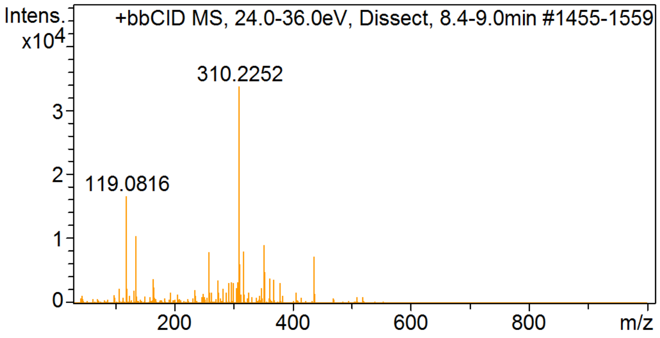

#	m/z	Res.	S/N	I	I %	FWHM
1	235.1616	29740	484.8	25952	48.5	0.0079
2	253.1716	29805	320.1	17137	32.0	0.0085
3	260.0114	29486	217.7	11655	21.8	0.0088
4	275.1528	31811	394.0	21093	39.4	0.0086
5	288.2440	30614	152.3	8152	15.2	0.0094
6	318.2898	31581	341.1	18259	34.1	0.0101
7	322.1901	29972	152.0	8137	15.2	0.0107
8	352.2343	32860	237.2	12701	23.7	0.0107
9	415.1978	32378	999.6	53514	100.0	0.0128
10	416.2009	30961	264.2	14145	26.4	0.0134

#	m/z	Res.	S/N	I	I %	FWHM
1	119.0566	22601	208.9	7045	20.9	0.0053
2	119.0816	23080	494.8	16686	49.5	0.0052
3	135.0760	24375	312.0	10521	31.2	0.0055
4	259.1605	29629	237.7	8015	23.8	0.0087
5	310.2252	31724	1000.0	33718	100.0	0.0098
6	311.2281	30328	182.3	6147	18.2	0.0103
7	318.2899	31704	239.7	8081	24.0	0.0100
8	352.2341	33380	268.9	9068	26.9	0.0106
9	353.2374	30759	145.4	4902	14.5	0.0115
10	437.1791	30885	217.6	7336	21.8	0.0142

Compound Spectrum List Report

Cmpd 68, Dissect, 8.9 min

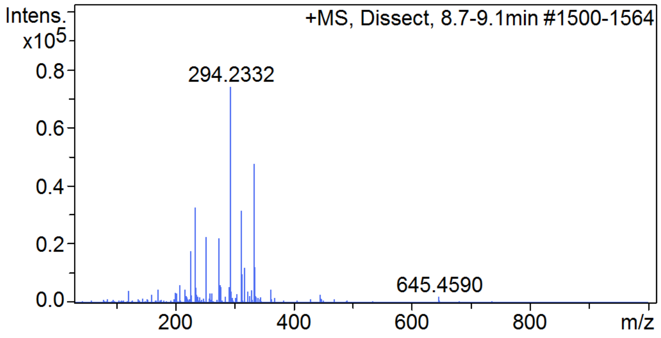

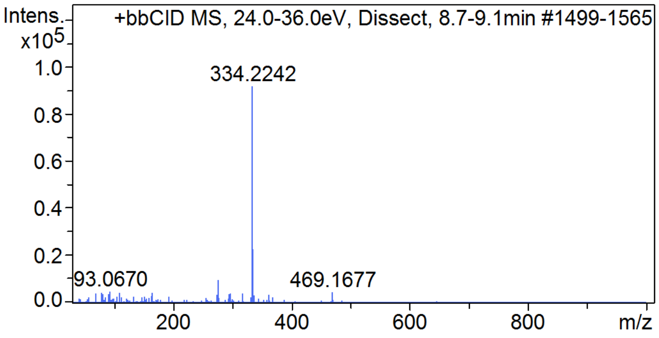

#	m/z	Res.	S/N	I	I %	FWHM
1	227.1567	29019	242.0	17933	24.2	0.0078
2	235.1615	29518	440.3	32625	44.0	0.0080
3	253.1716	29455	307.0	22753	30.7	0.0086
4	275.1529	31064	298.3	22108	29.8	0.0089
5	294.2332	31697	999.5	74064	100.0	0.0093
6	312.2432	33115	426.2	31579	42.6	0.0094
7	313.2464	30471	134.5	9965	13.5	0.0103
8	318.2899	31163	163.1	12088	16.3	0.0102
9	334.2244	33734	643.4	47676	64.4	0.0099
10	335.2274	30239	166.8	12362	16.7	0.0111

#	m/z	Res.	S/N	I	I %	FWHM
1	69.0674	18171	44.8	4109	4.5	0.0038
2	79.0515	18981	50.1	4591	5.0	0.0042
3	93.0670	19170	54.0	4951	5.4	0.0049
4	109.0976	21826	49.8	4568	5.0	0.0050
5	165.1219	26223	47.3	4338	4.7	0.0063
6	277.2072	31679	106.7	9787	10.7	0.0088
7	318.2900	31482	44.6	4088	4.5	0.0101
8	334.2242	33587	999.8	91661	100.0	0.0100
9	335.2275	31379	248.5	22786	24.9	0.0107
10	469.1677	30382	51.9	4758	5.2	0.0154

Cmpd 69, Dissect, 9.0 min

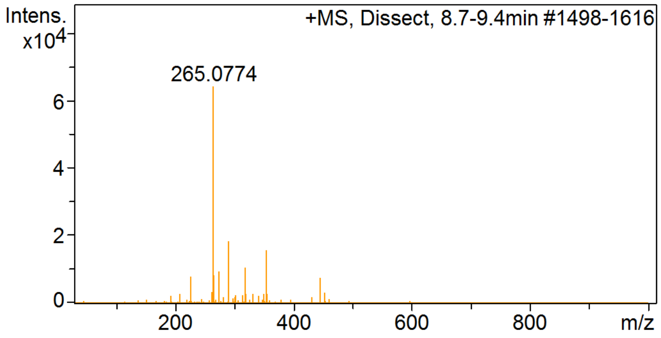

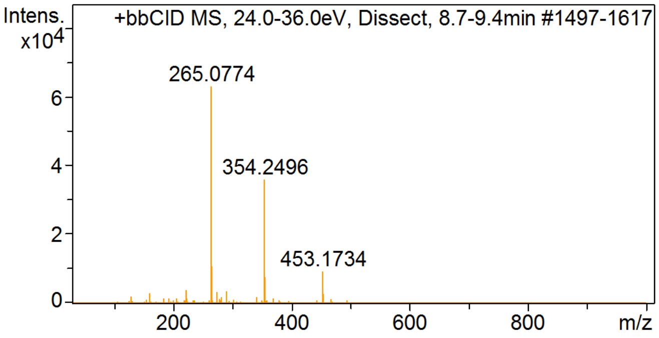

#	m/z	Res.	S/N	I	I %	FWHM
1	227.1567	28813	125.0	8042	12.5	0.0079
2	263.1921	29863	54.7	3522	5.5	0.0088
3	265.0774	31927	997.7	64203	100.0	0.0083
4	266.0808	30721	130.9	8425	13.1	0.0087
5	274.2652	31060	149.6	9628	15.0	0.0088
6	291.1837	32971	287.5	18499	28.8	0.0088
7	319.1647	31560	115.5	7432	11.6	0.0101
8	319.2165	31203	166.0	10683	16.6	0.0102
9	354.2502	30732	245.5	15800	24.6	0.0115
10	445.1974	29690	117.9	7588	11.8	0.0150

#	m/z	Res.	S/N	I	I %	FWHM
1	161.1272	27169	48.2	3032	4.8	0.0059
2	223.0680	29937	63.8	4017	6.4	0.0075
3	265.0774	32412	1000.0	62944	100.0	0.0082
4	266.0809	30727	171.3	10781	17.1	0.0087
5	274.2653	32485	54.9	3454	5.5	0.0084
6	291.1838	32744	56.5	3559	5.7	0.0089
7	354.2496	32989	569.6	35850	57.0	0.0107
8	355.2528	30169	121.8	7664	12.2	0.0118
9	453.1734	31296	149.4	9403	14.9	0.0145
10	454.1766	31548	46.3	2911	4.6	0.0144

Compound Spectrum List Report

Cmpd 70, Dissect, 9.1 min

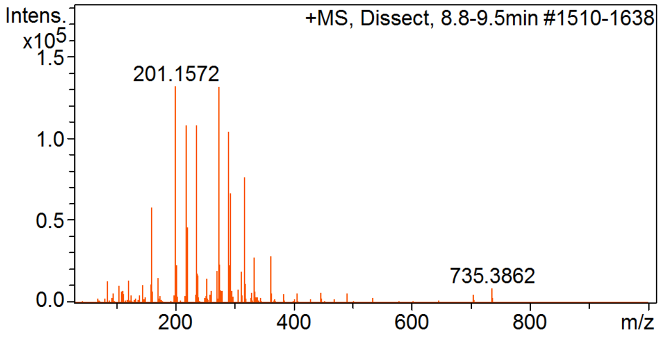

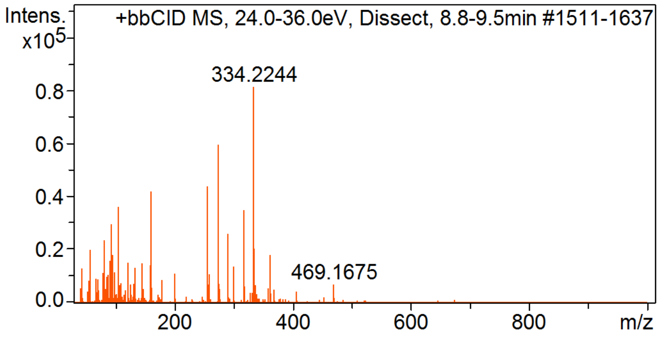

#	m/z	Res.	S/N	I	I %	FWHM
1	161.1272	26984	439.6	57966	44.0	0.0060
2	201.1572	29598	999.4	131773	100.0	0.0068
3	219.1673	30534	817.4	107776	81.8	0.0072
4	221.1828	29577	348.1	45899	34.8	0.0075
5	237.1772	31303	816.6	107673	81.7	0.0076
6	274.2652	31226	996.6	131408	99.7	0.0088
7	291.1837	33028	786.8	103739	78.7	0.0088
8	294.2333	31522	505.1	66595	50.5	0.0093
9	318.2900	33090	578.4	76265	57.9	0.0096
10	362.3147	32708	215.1	28364	21.5	0.0111

#	m/z	Res.	S/N	I	I %	FWHM
1	81.0671	19360	290.6	23719	29.1	0.0042
2	93.0669	19579	363.6	29675	36.4	0.0048
3	105.0665	21565	444.0	36236	44.5	0.0049
4	161.1272	27165	512.6	41833	51.4	0.0059
5	256.2552	32067	538.7	43969	54.0	0.0080
6	274.2652	32569	729.5	59540	73.1	0.0084
7	291.1838	33001	319.2	26048	32.0	0.0088
8	318.2900	33950	429.3	35034	43.0	0.0094
9	334.2244	34504	998.1	81462	100.0	0.0097
10	335.2276	31293	251.4	20516	25.2	0.0107

Cmpd 71, Dissect, 9.2 min

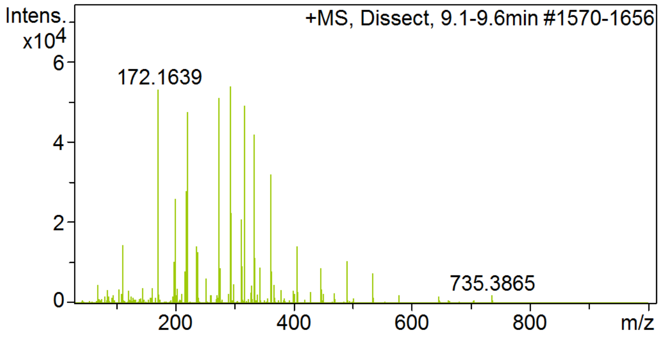

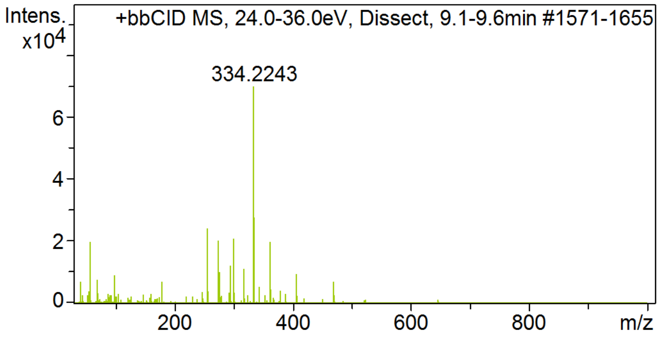

#	m/z	Res.	S/N	I	I %	FWHM
1	172.1639	27137	984.6	53050	98.5	0.0063
2	201.1573	29096	482.4	25994	48.2	0.0069
3	219.1673	30205	516.4	27823	51.6	0.0073
4	221.1829	29477	879.0	47362	87.9	0.0075
5	274.2653	31575	944.8	50904	94.5	0.0087
6	294.2333	31584	999.9	53877	100.0	0.0093
7	295.2366	31117	418.2	22532	41.8	0.0095
8	318.2900	33303	909.4	48996	90.9	0.0096
9	334.2245	33857	775.2	41768	77.5	0.0099
10	362.3147	33072	593.5	31976	59.3	0.0110

#	m/z	Res.	S/N	I	I %	FWHM
1	57.0677	17297	285.6	19976	28.6	0.0033
2	256.2551	31944	346.4	24232	34.7	0.0080
3	274.2651	32386	289.9	20283	29.0	0.0085
4	277.2073	31826	144.7	10119	14.5	0.0087
5	295.2172	31708	175.7	12288	17.6	0.0093
6	300.2798	31357	298.1	20855	29.8	0.0096
7	318.2900	33933	159.1	11132	15.9	0.0094
8	334.2243	34476	998.8	69868	100.0	0.0097
9	335.2277	31873	396.0	27699	39.6	0.0105
10	362.3145	33440	285.6	19979	28.6	0.0108

Compound Spectrum List Report

Cmpd 72, Dissect, 9.3 min

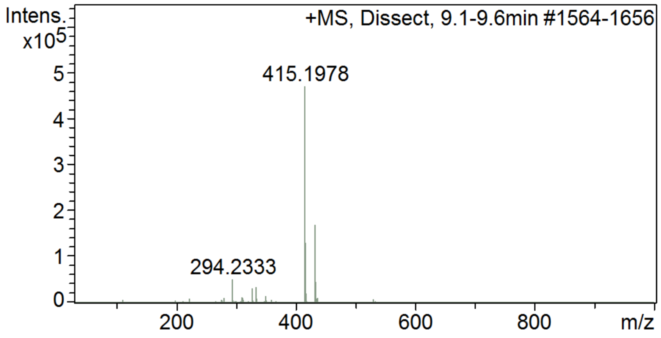

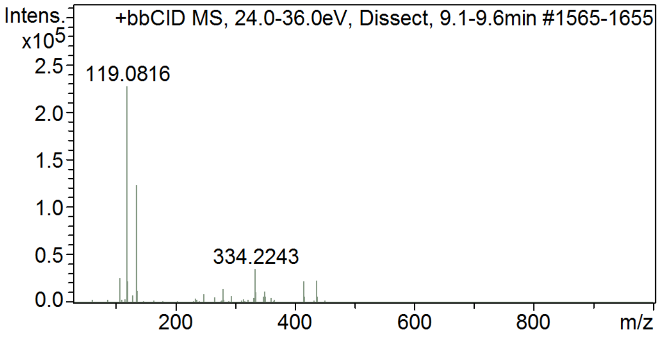

#	m/z	Res.	S/N	I	I %	FWHM
1	294.2333	31755	110.7	52609	11.2	0.0093
2	310.2277	32968	25.2	11967	2.5	0.0094
3	328.2376	32861	68.5	32531	6.9	0.0100
4	334.2244	33340	73.6	34946	7.4	0.0100
5	350.2187	31808	29.5	14024	3.0	0.0110
6	415.1978	37094	990.3	470456	100.0	0.0112
7	416.2012	34508	275.5	130860	27.8	0.0121
8	417.2039	29451	45.1	21448	4.6	0.0142
9	432.2237	35800	358.7	170408	36.2	0.0121
10	433.2270	32422	98.0	46551	9.9	0.0134

#	m/z	Res.	S/N	I	I %	FWHM
1	107.0820	22052	117.0	26632	11.7	0.0049
2	119.0566	22314	66.5	15128	6.7	0.0053
3	119.0816	23927	997.2	226976	100.0	0.0050
4	120.0850	22791	101.1	23004	10.1	0.0053
5	135.0760	25163	542.9	123565	54.4	0.0054
6	136.0793	24059	57.0	12984	5.7	0.0057
7	281.1291	30626	66.3	15084	6.6	0.0092
8	334.2243	34278	157.2	35789	15.8	0.0098
9	415.1977	31391	102.4	23297	10.3	0.0132
10	437.1788	31570	105.5	24012	10.6	0.0138

Cmpd 73, Dissect, 9.4 min

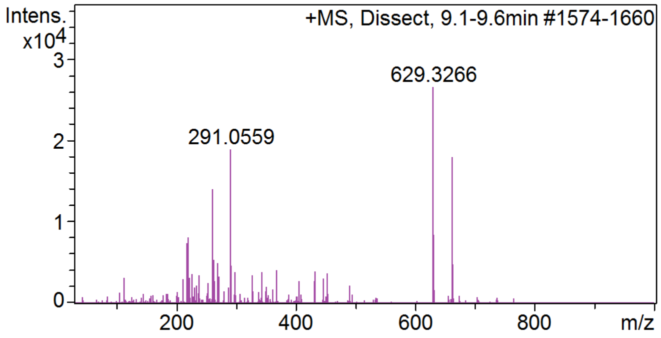

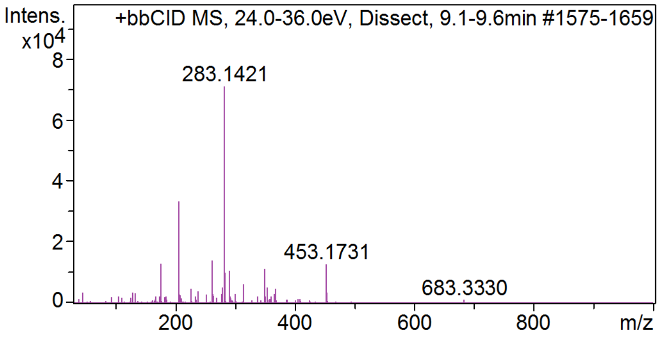

#	m/z	Res.	S/N	I	I %	FWHM
1	219.1672	29107	280.1	7451	28.0	0.0075
2	221.1828	28309	304.1	8090	30.4	0.0078
3	261.1299	31616	526.7	14012	52.7	0.0083
4	263.1921	28325	202.9	5397	20.3	0.0093
5	270.1959	28784	186.5	4962	18.6	0.0094
6	291.0559	31150	709.1	18866	70.9	0.0093
7	629.3266	31475	1000.0	26606	100.0	0.0200
8	630.3289	30888	318.1	8463	31.8	0.0204
9	661.3515	30679	673.2	17912	67.3	0.0216
10	662.3544	28675	182.5	4854	18.2	0.0231

#	m/z	Res.	S/N	I	I %	FWHM
1	177.0826	26637	184.2	13074	18.4	0.0066
2	207.0921	29127	469.2	33307	46.9	0.0071
3	263.0617	29788	198.6	14097	19.9	0.0088
4	283.1421	32852	999.6	70966	100.0	0.0086
5	284.1451	27996	142.3	10102	14.2	0.0101
6	291.0557	30771	152.2	10802	15.2	0.0095
7	315.1658	31384	89.8	6372	9.0	0.0100
8	350.2187	32512	159.2	11304	15.9	0.0108
9	354.2493	30113	75.4	5356	7.5	0.0118
10	453.1731	30916	182.0	12921	18.2	0.0147

Compound Spectrum List Report

Cmpd 74, Dissect, 9.4 min

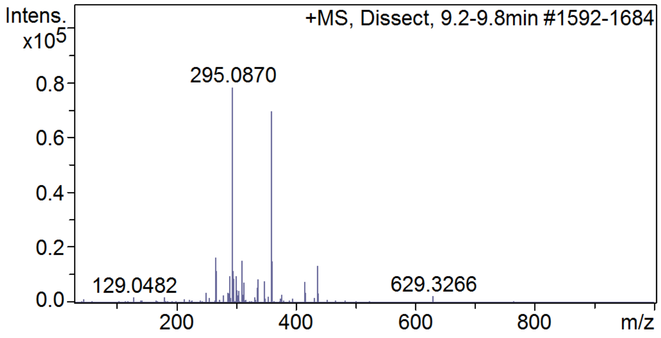

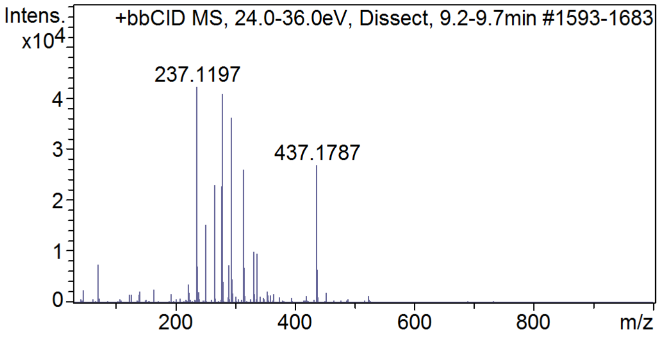

#	m/z	Res.	S/N	I	I %	FWHM
1	267.0929	30340	213.2	16665	21.3	0.0088
2	268.2184	32141	148.4	11600	14.8	0.0083
3	290.1998	30670	125.9	9840	12.6	0.0095
4	295.0870	31894	999.9	78176	100.0	0.0093
5	295.2365	30171	150.0	11731	15.0	0.0098
6	301.1336	31092	127.0	9927	12.7	0.0097
7	310.2276	33236	196.1	15332	19.6	0.0093
8	360.2205	33637	888.8	69487	88.9	0.0107
9	361.2230	26595	195.2	15258	19.5	0.0136
10	437.1788	30609	172.4	13479	17.2	0.0143

#	m/z	Res.	S/N	I	I %	FWHM
1	237.1197	30800	999.8	42248	100.0	0.0077
2	252.0699	30514	361.0	15254	36.1	0.0083
3	267.0929	30529	544.6	23015	54.5	0.0087
4	279.0560	30465	539.8	22810	54.0	0.0092
5	280.0636	30502	966.6	40844	96.7	0.0092
6	295.0870	32259	854.8	36120	85.5	0.0091
7	315.1653	31803	614.6	25969	61.5	0.0099
8	332.2087	32269	236.6	9999	23.7	0.0103
9	337.2429	30923	227.8	9628	22.8	0.0109
10	437.1787	31729	636.5	26898	63.7	0.0138

Cmpd 75, Dissect, 9.6 min

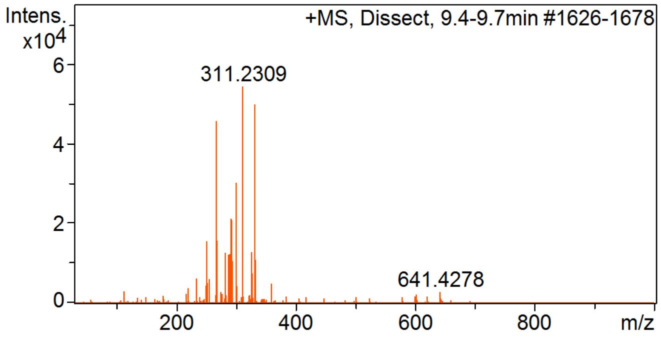

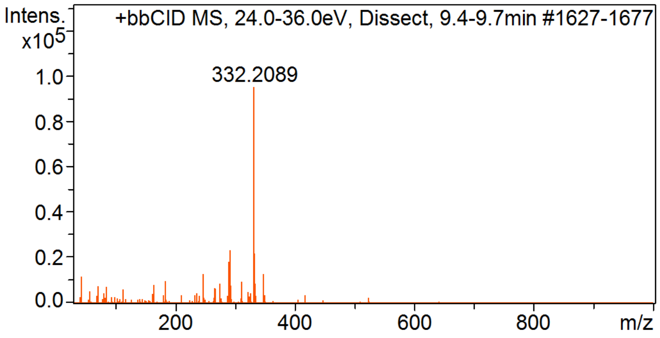

#	m/z	Res.	S/N	I	I %	FWHM
1	252.2240	31187	287.4	15658	28.8	0.0081
2	268.2184	32608	837.1	45610	83.8	0.0082
3	269.1924	32466	290.1	15808	29.0	0.0083
4	283.0873	31268	233.2	12706	23.3	0.0091
5	292.2165	31643	391.2	21318	39.2	0.0092
6	293.2016	31601	384.5	20953	38.5	0.0093
7	301.1337	30855	556.1	30300	55.7	0.0098
8	311.2309	33097	999.0	54436	100.0	0.0094
9	327.0759	31094	236.8	12904	23.7	0.0105
10	332.2088	32461	915.2	49869	91.6	0.0102

#	m/z	Res.	S/N	I	I %	FWHM
1	43.0524	15388	124.2	11837	12.4	0.0028
2	184.0935	29422	105.9	10094	10.6	0.0063
3	247.1976	30591	136.7	13030	13.7	0.0081
4	275.1917	30220	94.1	8970	9.4	0.0091
5	290.1998	31952	192.8	18371	19.3	0.0091
6	292.2155	32266	247.1	23554	24.7	0.0091
7	312.0526	30842	102.0	9718	10.2	0.0101
8	332.2089	33415	999.2	95237	100.0	0.0099
9	333.2120	32665	232.1	22121	23.2	0.0102
10	348.1820	32223	137.7	13125	13.8	0.0108

Compound Spectrum List Report

Cmpd 76, Dissect, 9.7 min

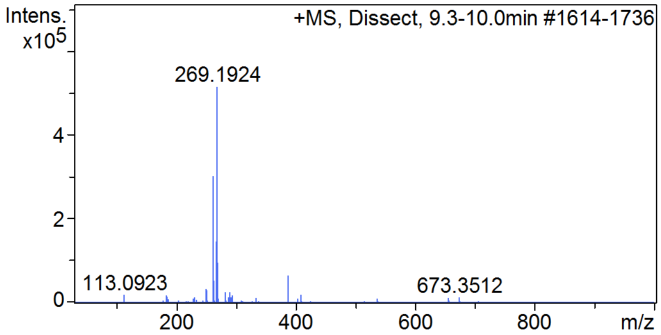

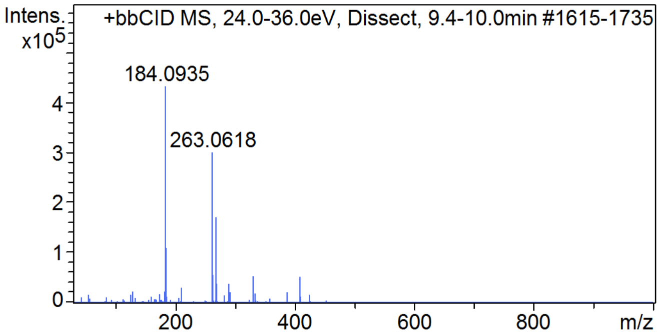

#	m/z	Res.	S/N	I	I %	FWHM
1	251.1924	29778	67.5	34845	6.8	0.0084
2	252.2241	31295	61.7	31876	6.2	0.0081
3	263.0618	33373	585.2	302293	58.6	0.0079
4	264.0652	30227	104.8	54124	10.5	0.0087
5	268.1844	29642	51.4	26556	5.1	0.0090
6	268.2185	33489	285.3	147355	28.6	0.0080
7	269.1924	35053	998.4	515739	100.0	0.0077
8	269.2212	28684	59.1	30531	5.9	0.0094
9	270.1958	31345	187.6	96906	18.8	0.0086
10	387.1804	33578	128.4	66301	12.9	0.0115

#	m/z	Res.	S/N	I	I %	FWHM
1	184.0935	30748	988.2	432285	100.0	0.0060
2	185.0995	22681	250.9	109759	25.4	0.0082
3	211.1161	29036	70.7	30911	7.2	0.0073
4	263.0618	33237	686.2	300185	69.4	0.0079
5	264.0653	30754	128.7	56316	13.0	0.0086
6	269.1926	31554	391.7	171335	39.6	0.0085
7	270.1959	30938	87.9	38453	8.9	0.0087
8	290.1999	31803	87.9	38469	8.9	0.0091
9	331.1199	32419	123.8	54144	12.5	0.0102
10	409.1616	32420	120.0	52484	12.1	0.0126

Cmpd 77, Dissect, 9.8 min

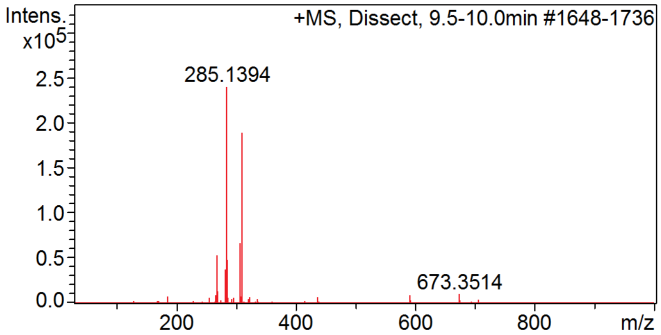

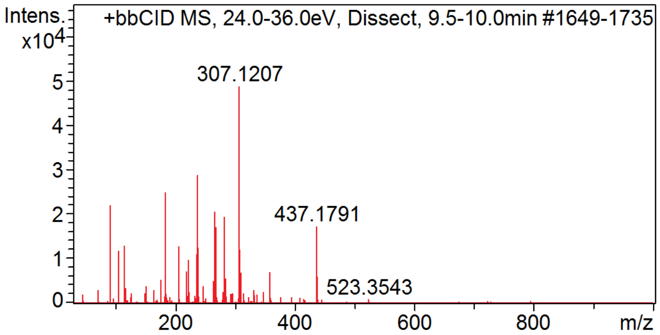

#	m/z	Res.	S/N	I	I %	FWHM
1	267.1770	29401	37.1	8948	3.7	0.0091
2	269.1925	34934	221.8	53444	22.3	0.0077
3	270.1959	31506	56.3	13555	5.7	0.0086
4	283.1239	31794	157.6	37972	15.8	0.0089
5	285.1394	33343	994.7	239660	100.0	0.0086
6	286.1428	31686	202.2	48727	20.3	0.0090
7	307.1206	31465	276.4	66589	27.8	0.0098
8	310.2277	34748	783.9	188878	78.8	0.0089
9	591.2520	33127	35.8	8617	3.6	0.0178
10	673.3514	31041	43.4	10465	4.4	0.0217

#	m/z	Res.	S/N	I	I %	FWHM
1	91.0513	20214	451.9	22096	45.3	0.0045
2	115.0505	22365	266.4	13024	26.7	0.0051
3	184.0935	30721	510.1	24942	51.1	0.0060
4	207.0923	28608	263.6	12889	26.4	0.0072
5	238.0912	28131	589.5	28823	59.1	0.0085
6	267.1294	31268	422.5	20658	42.4	0.0085
7	269.1926	31305	352.3	17224	35.3	0.0086
8	283.1423	31894	398.9	19505	40.0	0.0089
9	307.1207	32102	997.3	48765	100.0	0.0096
10	437.1791	32165	354.9	17355	35.6	0.0136

Compound Spectrum List Report

Cmpd 78, Dissect, 9.8 min

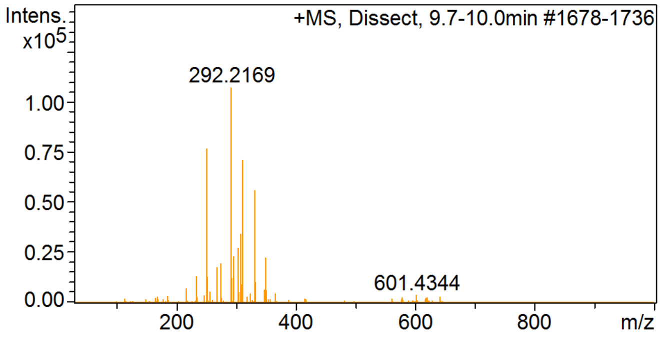

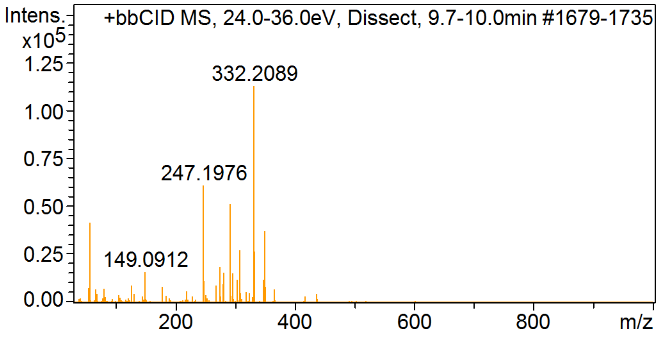

#	m/z	Res.	S/N	I	I %	FWHM
1	252.2241	31544	714.3	76599	71.5	0.0080
2	269.1925	33783	167.5	17965	16.8	0.0080
3	275.1916	30306	184.6	19791	18.5	0.0091
4	292.2169	32011	999.2	107150	100.0	0.0091
5	297.1025	31034	216.8	23246	21.7	0.0096
6	304.2900	32889	256.7	27533	25.7	0.0093
7	308.2104	31335	320.8	34405	32.1	0.0098
8	311.2310	32823	661.8	70969	66.2	0.0095
9	332.2089	32355	523.4	56130	52.4	0.0103
10	350.2188	31361	212.0	22737	21.2	0.0112

#	m/z	Res.	S/N	I	I %	FWHM
1	57.0677	17271	368.8	41753	37.0	0.0033
2	149.0912	26061	141.4	16012	14.2	0.0057
3	247.1976	31538	539.2	61045	54.1	0.0078
4	275.1916	30727	166.4	18840	16.7	0.0090
5	282.0790	29881	139.1	15749	13.9	0.0094
6	292.2155	32899	453.8	51379	45.5	0.0089
7	308.2097	32544	242.2	27419	24.3	0.0095
8	332.2089	33103	997.1	112895	100.0	0.0100
9	333.2120	32154	235.7	26681	23.6	0.0104
10	350.2188	32009	329.7	37330	33.1	0.0109

Cmpd 79, Dissect, 10.0 min

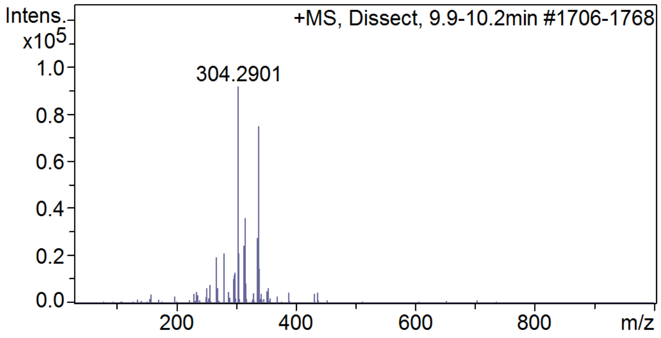

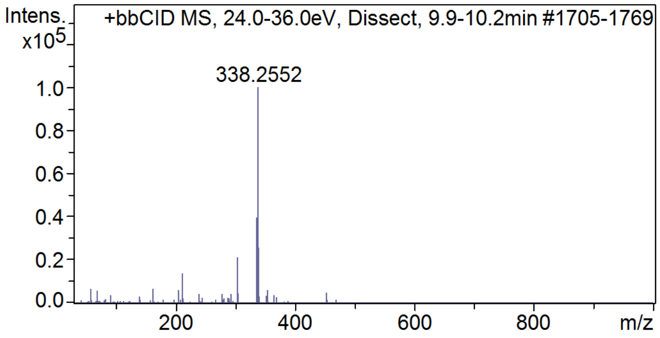

#	m/z	Res.	S/N	I	I %	FWHM
1	268.2185	32071	213.7	19614	21.4	0.0084
2	280.2544	31717	232.0	21294	23.2	0.0088
3	299.1182	30579	141.6	12995	14.2	0.0098
4	304.2901	33209	999.9	91764	100.0	0.0092
5	305.2932	31645	231.5	21250	23.2	0.0096
6	314.2589	32969	266.4	24446	26.6	0.0095
7	316.2744	33311	393.5	36110	39.4	0.0095
8	336.2401	32990	300.6	27585	30.1	0.0102
9	338.2556	33196	813.6	74673	81.4	0.0102
10	339.2588	31264	161.9	14855	16.2	0.0109

#	m/z	Res.	S/N	I	I %	FWHM
1	58.0629	17280	69.4	6962	6.9	0.0034
2	69.0673	18217	59.7	5989	6.0	0.0038
3	163.0335	26178	68.5	6866	6.9	0.0062
4	206.1085	28247	63.2	6334	6.3	0.0073
5	212.2302	29648	139.9	14025	14.0	0.0072
6	304.2897	32367	213.1	21370	21.3	0.0094
7	336.2398	34469	396.8	39797	39.7	0.0098
8	338.2552	33411	998.9	100175	100.0	0.0101
9	339.2586	32868	257.6	25830	25.8	0.0103
10	354.2284	29283	63.6	6381	6.4	0.0121

Compound Spectrum List Report

Cmpd 80, Dissect, 10.1 min

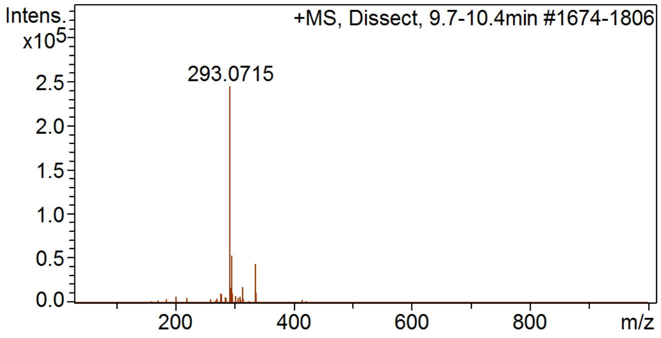

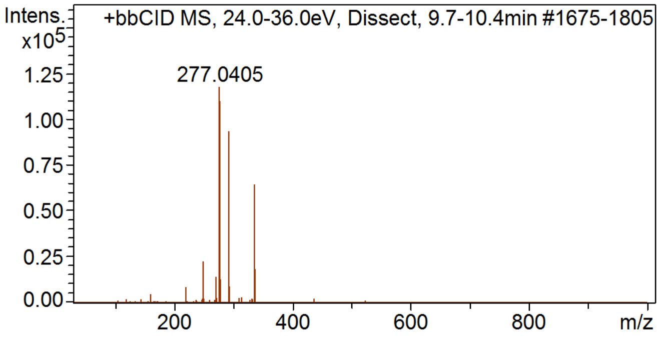

#	m/z	Res.	S/N	I	I %	FWHM
1	278.2389	30526	43.9	10761	4.4	0.0091
2	279.2229	30840	39.3	9641	3.9	0.0091
3	293.0715	33526	998.5	244707	100.0	0.0087
4	293.2203	28955	48.7	11929	4.9	0.0101
5	294.0749	32007	70.8	17354	7.1	0.0092
6	296.2489	32691	218.7	53607	21.9	0.0091
7	297.2522	31109	44.2	10824	4.4	0.0096
8	314.2589	32818	73.6	18042	7.4	0.0096
9	336.2401	32882	182.2	44645	18.2	0.0102
10	337.2433	32158	48.8	11960	4.9	0.0105

#	m/z	Res.	S/N	I	I %	FWHM
1	250.0543	30889	192.4	22659	19.3	0.0081
2	271.1242	31107	121.8	14350	12.2	0.0087
3	277.0405	33023	998.4	117589	100.0	0.0084
4	277.2073	30404	94.6	11146	9.5	0.0091
5	278.0477	30881	931.1	109658	93.3	0.0090
6	279.0513	29255	110.7	13043	11.1	0.0095
7	293.0714	33350	793.0	93400	79.4	0.0088
8	294.0747	31592	77.0	9064	7.7	0.0093
9	336.2398	34557	548.9	64643	55.0	0.0097
10	337.2431	32734	157.0	18491	15.7	0.0103

Cmpd 81, Dissect, 10.3 min

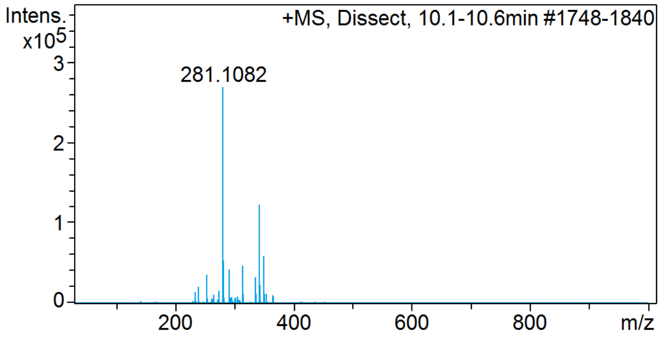

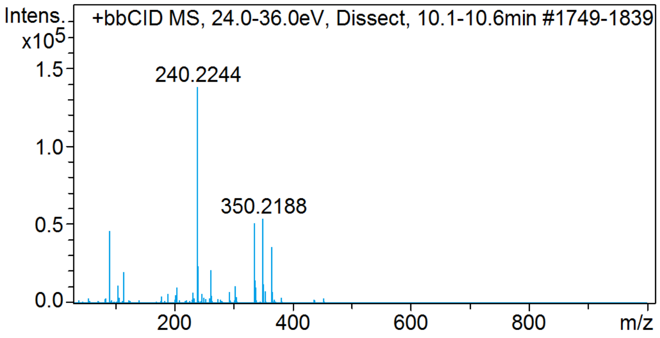

#	m/z	Res.	S/N	I	I %	FWHM
1	254.2396	29598	133.1	35894	13.3	0.0086
2	281.1082	32462	999.6	269592	100.0	0.0087
3	282.1116	30534	176.4	47571	17.6	0.0092
4	282.2337	31142	198.0	53403	19.8	0.0091
5	292.2174	31335	158.4	42710	15.8	0.0093
6	314.2588	32401	175.6	47351	17.6	0.0097
7	336.2400	33027	122.8	33127	12.3	0.0102
8	343.2843	32799	457.1	123285	45.7	0.0105
9	344.2875	30553	88.3	23826	8.8	0.0113
10	350.2189	32083	219.1	59099	21.9	0.0109

#	m/z	Res.	S/N	I	I %	FWHM
1	91.0513	20225	334.6	46167	33.5	0.0045
2	115.0505	22179	145.4	20053	14.5	0.0052
3	240.2244	31361	999.4	137869	100.0	0.0077
4	241.2277	29610	172.5	23790	17.3	0.0081
5	263.0982	31205	155.6	21462	15.6	0.0084
6	336.2400	33380	372.2	51352	37.2	0.0101
7	337.2432	32289	106.5	14695	10.7	0.0104
8	350.2188	33127	390.8	53915	39.1	0.0106
9	351.2219	31378	90.8	12524	9.1	0.0112
10	365.2655	32471	261.6	36086	26.2	0.0112

Compound Spectrum List Report

Cmpd 82, Dissect, 10.4 min

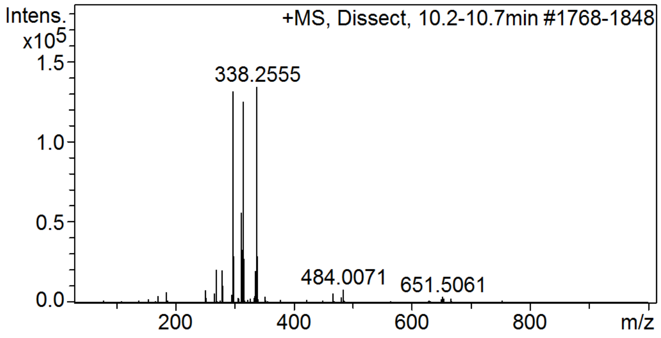

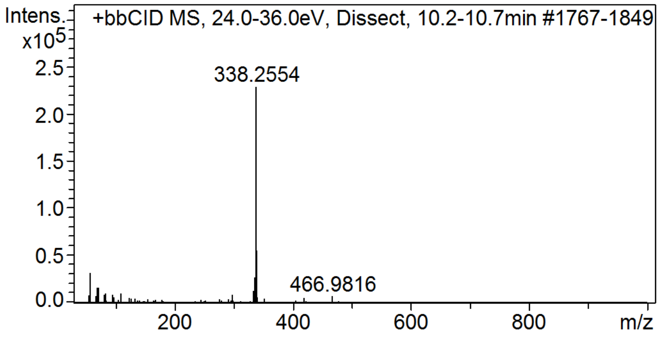

#	m/z	Res.	S/N	I	I %	FWHM
1	270.2341	30908	155.3	20898	15.5	0.0087
2	280.2544	31084	151.6	20404	15.2	0.0090
3	298.2644	31941	976.9	131484	97.7	0.0093
4	299.2678	30535	218.1	29356	21.8	0.0098
5	312.2433	33526	416.6	56073	41.7	0.0093
6	314.2588	32346	246.5	33171	24.7	0.0097
7	316.2744	33076	927.3	124802	92.8	0.0096
8	317.2778	32093	205.7	27679	20.6	0.0099
9	338.2555	33532	999.6	134542	100.0	0.0101
10	339.2588	31943	217.7	29305	21.8	0.0106

#	m/z	Res.	S/N	I	I %	FWHM
1	57.0677	17332	139.7	32058	14.0	0.0033
2	69.0673	18246	71.4	16389	7.2	0.0038
3	71.0829	18475	70.9	16275	7.1	0.0038
4	81.0671	19327	37.5	8601	3.8	0.0042
5	83.0827	19336	42.6	9781	4.3	0.0043
6	109.0977	21835	44.4	10198	4.5	0.0050
7	334.2243	33958	57.0	13074	5.7	0.0098
8	336.2400	33232	118.3	27155	11.9	0.0101
9	338.2554	34239	997.4	228921	100.0	0.0099
10	339.2587	33148	241.6	55462	24.2	0.0102

Cmpd 83, Dissect, 10.5 min

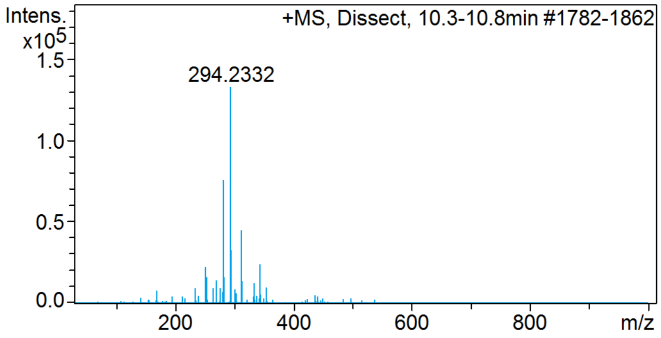

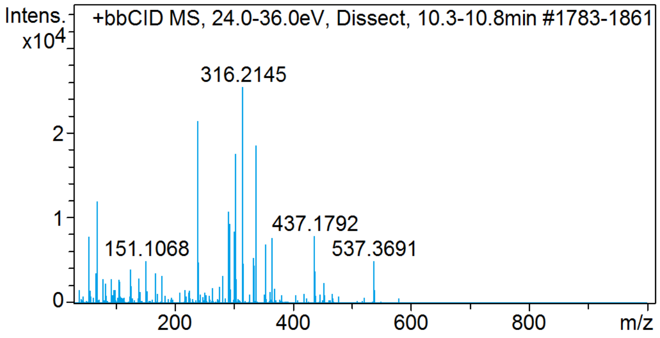

#	m/z	Res.	S/N	I	I %	FWHM
1	252.2240	31010	168.5	22430	16.9	0.0081
2	254.2395	28892	122.8	16337	12.3	0.0088
3	270.2341	31367	106.8	14216	10.7	0.0086
4	282.2337	31911	569.0	75722	57.0	0.0088
5	283.2369	30505	122.0	16242	12.2	0.0093
6	294.2332	31871	998.2	132847	100.0	0.0092
7	295.2365	31709	245.8	32711	24.6	0.0093
8	312.2433	33432	337.3	44894	33.8	0.0093
9	313.2465	30586	105.0	13978	10.5	0.0102
10	343.2844	32128	182.4	24274	18.3	0.0107

#	m/z	Res.	S/N	I	I %	FWHM
1	55.0521	17265	308.8	7857	30.9	0.0032
2	69.0674	18320	472.1	12010	47.2	0.0038
3	240.2245	30812	841.3	21405	84.2	0.0078
4	292.2154	32029	424.9	10811	42.5	0.0091
5	294.2325	31267	366.6	9327	36.7	0.0094
6	301.1338	30355	331.4	8433	33.2	0.0099
7	304.2149	31319	688.7	17522	68.9	0.0097
8	316.2145	31892	999.5	25429	100.0	0.0099
9	338.2555	33969	726.7	18490	72.7	0.0100
10	437.1792	31712	311.8	7934	31.2	0.0138

Compound Spectrum List Report

Cmpd 84, Dissect, 10.6 min

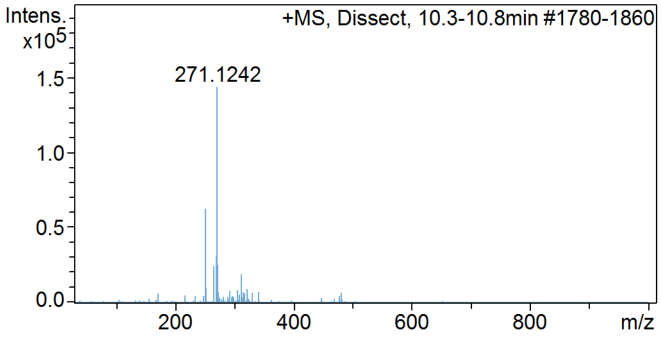

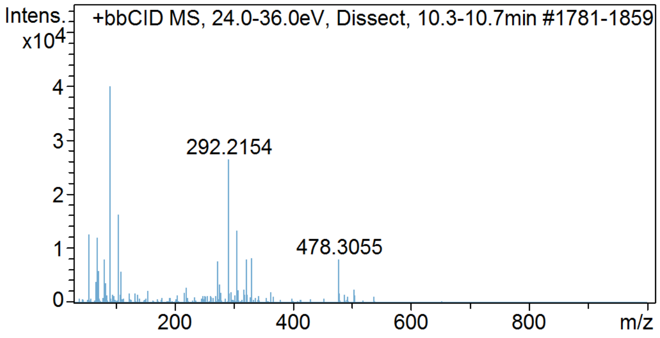

#	m/z	Res.	S/N	I	I %	FWHM
1	252.2240	31258	433.7	62466	43.5	0.0081
2	253.2079	29817	68.0	9794	6.8	0.0085
3	253.2273	29211	72.0	10364	7.2	0.0087
4	266.2392	30519	173.9	25049	17.4	0.0087
5	270.2341	31438	217.7	31353	21.8	0.0086
6	271.1242	32327	998.1	143764	100.0	0.0084
7	271.2375	30402	109.1	15710	10.9	0.0089
8	272.1275	30543	177.1	25507	17.7	0.0089
9	312.2433	33351	134.0	19301	13.4	0.0094
10	322.2247	29870	66.0	9505	6.6	0.0108

#	m/z	Res.	S/N	I	I %	FWHM
1	55.0520	17266	317.4	12690	31.7	0.0032
2	69.0674	18274	301.5	12054	30.1	0.0038
3	81.0671	19376	202.3	8088	20.2	0.0042
4	91.0513	20181	1000.0	39979	100.0	0.0045
5	105.0665	21262	408.4	16327	40.8	0.0049
6	292.2154	32146	661.4	26442	66.1	0.0091
7	306.2303	30942	335.1	13397	33.5	0.0099
8	322.2248	30625	202.6	8100	20.3	0.0105
9	331.2060	31073	206.7	8264	20.7	0.0107
10	478.3055	30342	201.2	8044	20.1	0.0158

Cmpd 85, Dissect, 10.7 min

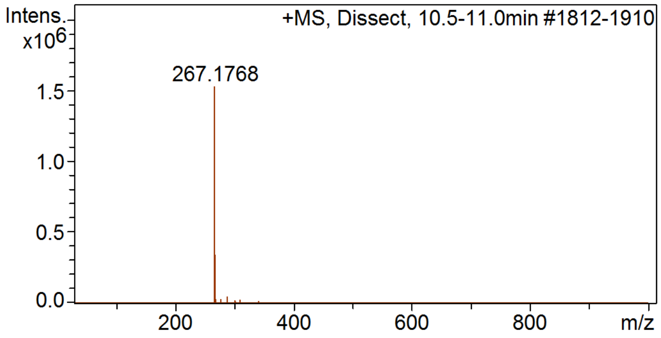

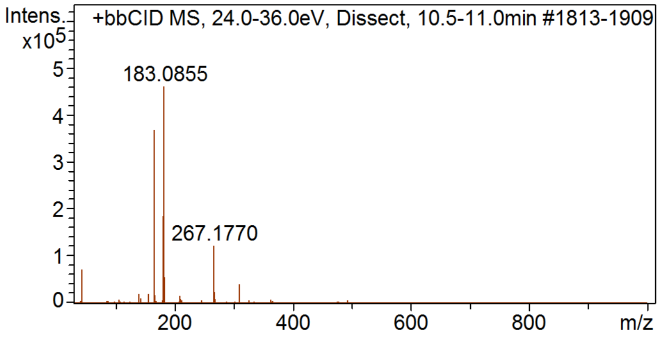

#	m/z	Res.	S/N	I	I %	FWHM
1	267.1768	36825	987.2	1529121	100.0	0.0073
2	268.1803	33416	223.6	346326	22.6	0.0080
3	269.1842	25472	17.8	27568	1.8	0.0106
4	278.2389	31053	18.2	28230	1.8	0.0090
5	288.2440	31466	31.6	48902	3.2	0.0092
6	289.2473	30156	5.6	8621	0.6	0.0096
7	301.1334	30144	13.2	20462	1.3	0.0100
8	302.2955	30051	6.4	9970	0.7	0.0101
9	310.2254	30439	15.6	24144	1.6	0.0102
10	341.2323	31362	9.3	14476	0.9	0.0109

#	m/z	Res.	S/N	I	I %	FWHM
1	43.0524	15453	156.2	72323	15.7	0.0028
2	156.0756	26017	46.3	21448	4.7	0.0060
3	166.0596	29169	794.6	367925	79.8	0.0057
4	167.0668	25502	361.4	167352	36.3	0.0066
5	182.0778	29741	401.5	185932	40.3	0.0061
6	183.0855	30410	995.5	460969	100.0	0.0060
7	184.0892	25992	121.8	56407	12.2	0.0071
8	267.1770	31967	264.5	122454	26.6	0.0084
9	268.1803	30900	55.5	25678	5.6	0.0087
10	310.2254	31699	90.4	41868	9.1	0.0098

Compound Spectrum List Report

Cmpd 86, Dissect, 10.9 min

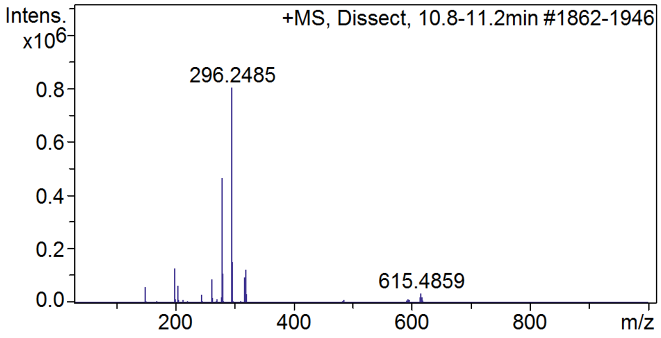

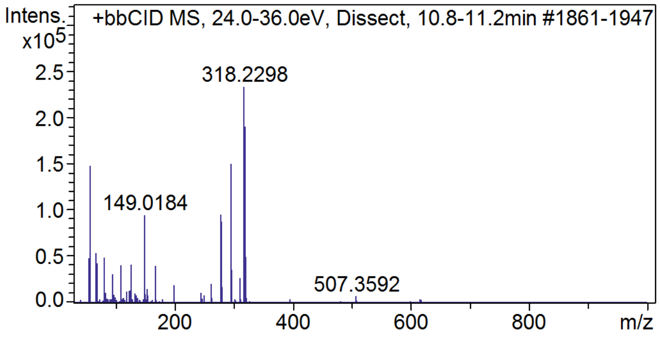

#	m/z	Res.	S/N	I	I %	FWHM
1	149.0185	26243	74.4	59800	7.4	0.0057
2	200.1942	29469	160.0	128635	16.0	0.0068
3	205.0791	30103	82.2	66061	8.2	0.0068
4	263.2283	32628	111.0	89263	11.1	0.0081
5	280.2541	36762	579.4	465758	58.0	0.0076
6	281.2577	32935	137.1	110201	13.7	0.0085
7	296.2485	38196	999.3	803286	100.0	0.0078
8	297.2519	35634	190.0	152748	19.0	0.0083
9	318.2299	34687	120.6	96927	12.1	0.0092
10	320.2454	35162	155.5	124985	15.6	0.0091

#	m/z	Res.	S/N	I	I %	FWHM
1	57.0677	17718	631.7	147493	63.2	0.0032
2	67.0518	18421	232.5	54284	23.3	0.0036
3	81.0670	20110	211.6	49400	21.2	0.0040
4	149.0184	27052	404.3	94395	40.4	0.0055
5	279.2228	33384	409.6	95646	41.0	0.0084
6	280.2544	33088	375.2	87618	37.5	0.0085
7	296.2487	34369	642.3	149987	64.2	0.0086
8	318.2298	36947	999.9	233466	100.0	0.0086
9	320.2454	35874	812.5	189718	81.3	0.0089
10	321.2489	32935	214.8	50157	21.5	0.0098

Cmpd 87, Dissect, 11.1 min

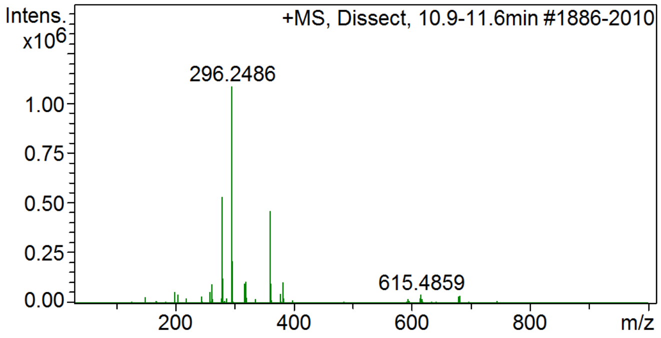

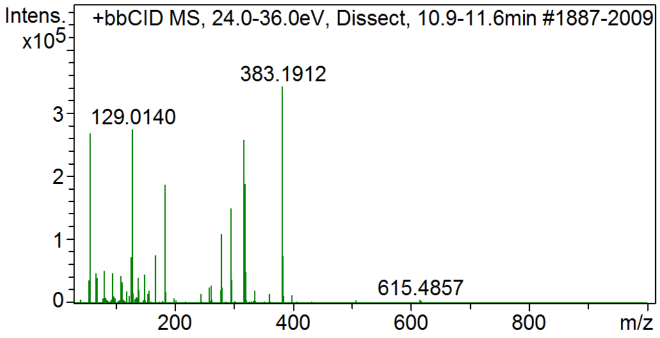

#	m/z	Res.	S/N	I	I %	FWHM
1	263.2283	32567	86.6	98500	9.1	0.0081
2	280.2541	37235	467.4	531404	49.0	0.0075
3	281.2577	33154	111.1	126292	11.6	0.0085
4	296.2486	34519	954.6	1085254	100.0	0.0086
5	297.2519	36927	186.8	212360	19.6	0.0080
6	318.2298	35373	88.0	100057	9.2	0.0090
7	320.2454	35038	98.3	111796	10.3	0.0091
8	361.2101	39007	407.0	462687	42.6	0.0093
9	362.2135	32892	87.4	99401	9.2	0.0110
10	383.1911	33625	93.4	106211	9.8	0.0114

#	m/z	Res.	S/N	I	I %	FWHM
1	57.0677	17748	781.0	267326	78.1	0.0032
2	129.0140	25133	800.2	273889	80.1	0.0051
3	169.1166	27554	223.5	76493	22.4	0.0061
4	185.0747	30213	545.9	186863	54.6	0.0061
5	280.2542	33529	319.7	109446	32.0	0.0084
6	296.2486	35911	438.6	150142	43.9	0.0082
7	318.2298	38310	751.6	257262	75.2	0.0083
8	320.2453	36159	550.5	188444	55.1	0.0089
9	383.1912	37560	999.5	342136	100.0	0.0102
10	384.1947	33370	220.4	75431	22.0	0.0115

Compound Spectrum List Report

Cmpd 88, Dissect, 11.2 min

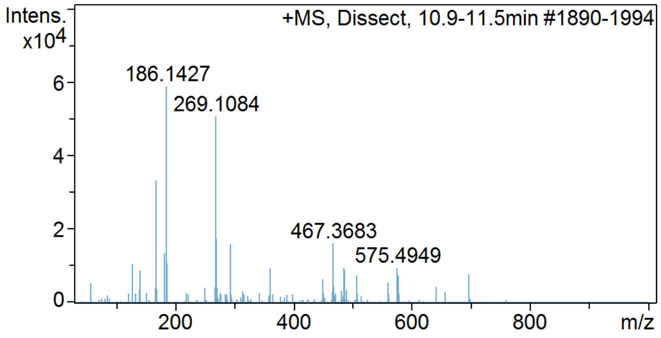

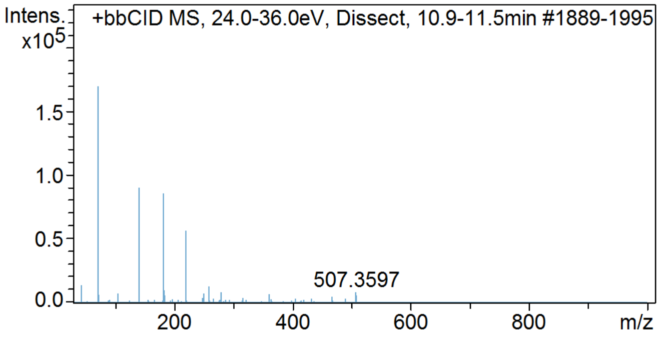

#	m/z	Res.	S/N	I	I %	FWHM
1	128.1391	23086	180.2	10605	18.0	0.0056
2	169.1166	26839	565.5	33284	56.6	0.0063
3	183.1318	26701	230.1	13543	23.0	0.0069
4	186.1427	28092	1000.0	58849	100.0	0.0066
5	187.1267	27511	185.0	10885	18.5	0.0068
6	269.1084	30669	859.2	50565	85.9	0.0088
7	270.1117	29401	295.5	17390	29.6	0.0092
8	294.2329	28468	272.1	16014	27.2	0.0103
9	467.3683	30512	276.0	16243	27.6	0.0153
10	575.4949	31695	164.0	9650	16.4	0.0182

#	m/z	Res.	S/N	I	I %	FWHM
1	43.0525	15305	82.7	14031	8.3	0.0028
2	71.0465	18470	139.5	23648	13.9	0.0038
3	71.0829	19374	999.8	169537	100.0	0.0037
4	141.1226	25377	531.7	90155	53.2	0.0056
5	183.1318	28102	505.8	85771	50.6	0.0065
6	184.1346	22944	60.1	10192	6.0	0.0080
7	220.1236	29448	333.7	56579	33.4	0.0075
8	259.1454	30687	78.5	13307	7.8	0.0084
9	280.2542	34044	52.8	8955	5.3	0.0082
10	507.3597	30343	51.8	8788	5.2	0.0167

Cmpd 89, Dissect, 11.2 min

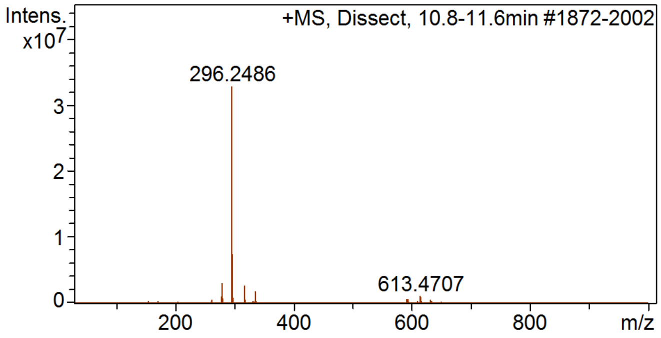

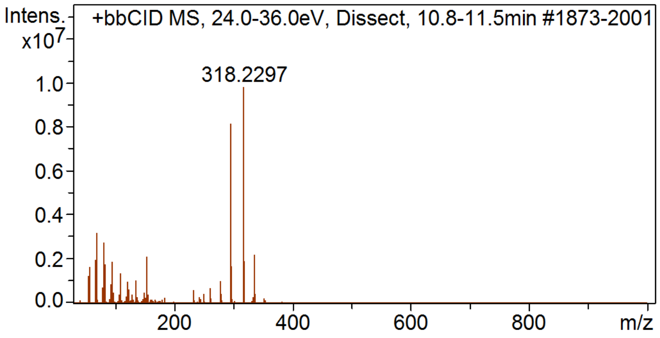

#	m/z	Res.	S/N	I	I %	FWHM
1	279.2226	35047	29.4	969135	3.0	0.0080
2	280.2541	37361	94.4	3112075	9.5	0.0075
3	281.2576	33355	22.8	751353	2.3	0.0084
4	296.2486	30121	995.7	32822248	100.0	0.0098
5	297.2518	37381	228.2	7521699	22.9	0.0080
6	298.2555	27284	25.1	827846	2.5	0.0109
7	318.2298	35608	84.7	2790508	8.5	0.0089
8	336.2397	33624	56.7	1869422	5.7	0.0100
9	613.4707	33022	33.0	1088617	3.3	0.0186
10	615.4856	31042	27.1	894035	2.7	0.0198

#	m/z	Res.	S/N	I	I %	FWHM
1	67.0518	18706	201.8	1984970	20.2	0.0036
2	69.0673	19110	325.0	3196413	32.6	0.0036
3	81.0670	20383	281.3	2767126	28.2	0.0040
4	83.0825	20200	182.1	1791128	18.2	0.0041
5	95.0824	21261	194.1	1908846	19.4	0.0045
6	153.1222	27003	217.4	2138231	21.8	0.0057
7	296.2485	36969	827.6	8140231	82.9	0.0080
8	318.2297	38735	997.9	9814987	100.0	0.0082
9	319.2332	33384	196.0	1927499	19.6	0.0096
10	336.2398	34554	226.2	2224745	22.7	0.0097

Compound Spectrum List Report

Cmpd 90, Dissect, 11.3 min

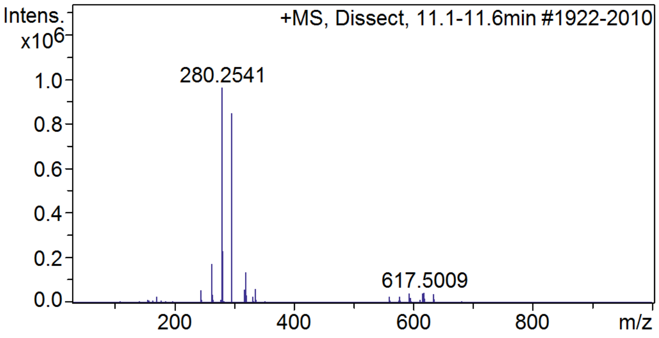

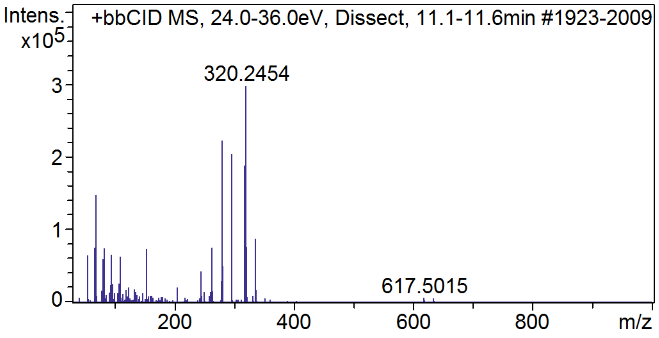

#	m/z	Res.	S/N	I	I %	FWHM
1	245.2183	29771	59.4	57661	6.0	0.0082
2	263.2283	33376	182.1	176658	18.3	0.0079
3	280.2541	37584	992.8	963285	100.0	0.0075
4	281.2576	33937	239.3	232202	24.1	0.0083
5	296.2485	33834	871.8	845855	87.8	0.0088
6	297.2518	36692	157.4	152738	15.9	0.0081
7	318.2298	35004	63.5	61629	6.4	0.0091
8	320.2453	35525	143.5	139277	14.5	0.0090
9	336.2398	33603	65.6	63624	6.6	0.0100
10	617.5009	29728	48.6	47190	4.9	0.0208

#	m/z	Res.	S/N	I	I %	FWHM
1	67.0518	18634	255.6	76199	25.6	0.0036
2	69.0673	19151	497.2	148236	49.8	0.0036
3	83.0825	20330	252.8	75370	25.3	0.0041
4	263.2284	31819	255.2	76095	25.6	0.0083
5	280.2543	34312	747.1	222742	74.8	0.0082
6	296.2486	36281	685.0	204245	68.6	0.0082
7	318.2298	37904	630.0	187835	63.1	0.0084
8	320.2454	37185	998.1	297592	100.0	0.0086
9	321.2488	33222	257.2	76689	25.8	0.0097
10	336.2398	34067	296.4	88383	29.7	0.0099

Cmpd 91, Dissect, 11.4 min

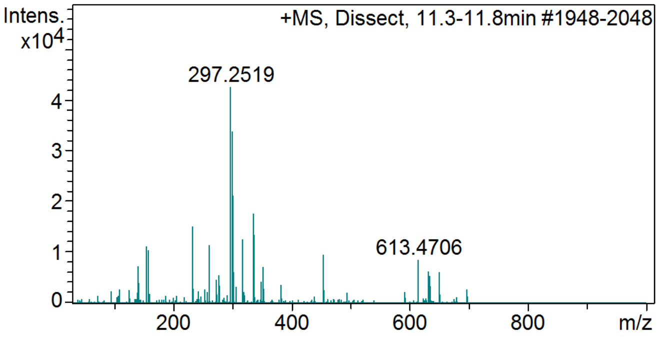

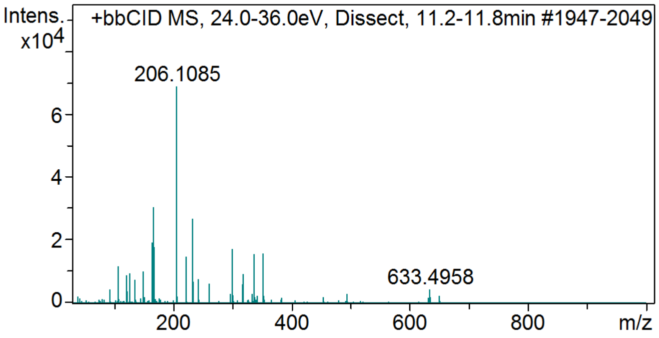

#	m/z	Res.	S/N	I	I %	FWHM
1	155.1015	25658	263.8	11254	26.4	0.0060
2	158.1123	25825	245.2	10462	24.5	0.0061
3	233.2187	29152	356.0	15188	35.6	0.0080
4	261.2127	30179	269.6	11500	27.0	0.0087
5	297.2519	35197	999.0	42619	100.0	0.0084
6	300.9825	31087	791.6	33772	79.2	0.0097
7	301.1487	30611	496.1	21165	49.7	0.0098
8	318.2299	33521	295.2	12596	29.6	0.0095
9	336.2398	33560	415.4	17723	41.6	0.0100
10	337.2431	30787	315.9	13475	31.6	0.0110

#	m/z	Res.	S/N	I	I %	FWHM
1	107.0820	21993	172.1	11846	17.2	0.0049
2	165.9725	27183	282.6	19454	28.3	0.0061
3	167.1374	27423	442.1	30437	44.2	0.0061
4	169.1167	27376	259.6	17869	26.0	0.0062
5	206.1085	29394	999.6	68816	100.0	0.0070
6	223.1044	28204	216.6	14911	21.7	0.0079
7	233.2187	30451	389.0	26783	38.9	0.0077
8	300.9824	31395	251.0	17279	25.1	0.0096
9	337.2431	31631	226.9	15620	22.7	0.0107
10	352.2132	31795	231.7	15950	23.2	0.0111

Compound Spectrum List Report

Cmpd 92, Dissect, 11.5 min

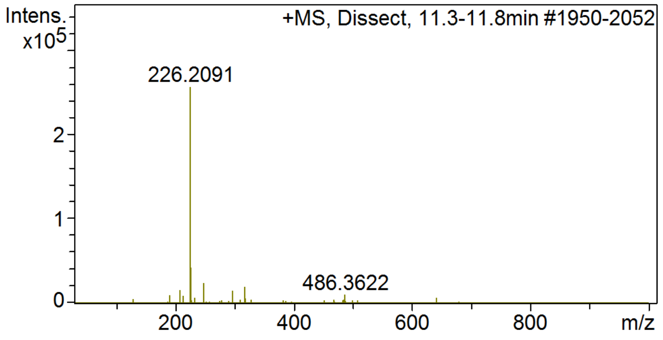

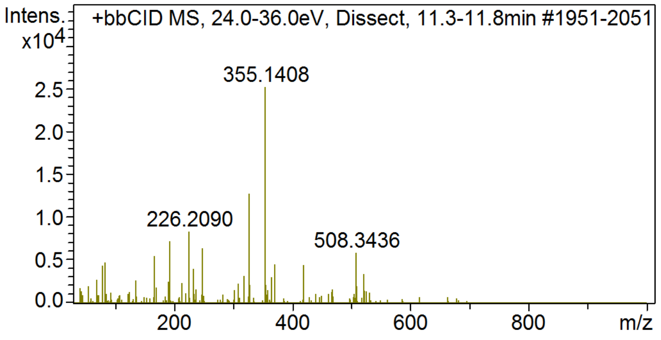

#	m/z	Res.	S/N	I	I %	FWHM
1	191.1731	28241	38.2	9802	3.8	0.0068
2	209.1831	28193	63.1	16215	6.3	0.0074
3	214.2096	28073	33.0	8479	3.3	0.0076
4	226.2091	31778	999.0	256581	100.0	0.0071
5	227.2124	29378	166.1	42651	16.6	0.0077
6	248.1904	29175	94.6	24302	9.5	0.0085
7	248.2502	29071	31.9	8201	3.2	0.0085
8	297.2520	34721	59.7	15338	6.0	0.0086
9	318.2299	33177	77.0	19789	7.7	0.0096
10	486.3622	31146	38.8	9960	3.9	0.0156

#	m/z	Res.	S/N	I	I %	FWHM
1	83.0826	19949	191.5	4831	19.2	0.0042
2	167.1374	27435	218.6	5514	21.9	0.0061
3	194.1088	27995	289.2	7294	28.9	0.0069
4	226.2090	29589	332.6	8389	33.3	0.0076
5	248.1904	28312	256.1	6460	25.6	0.0088
6	327.2172	32070	507.6	12804	50.8	0.0102
7	355.1408	31690	999.5	25212	100.0	0.0112
8	371.1141	30541	182.0	4590	18.2	0.0122
9	418.9798	32368	179.7	4532	18.0	0.0129
10	508.3436	30676	234.9	5925	23.5	0.0166

Cmpd 93, Dissect, 11.6 min

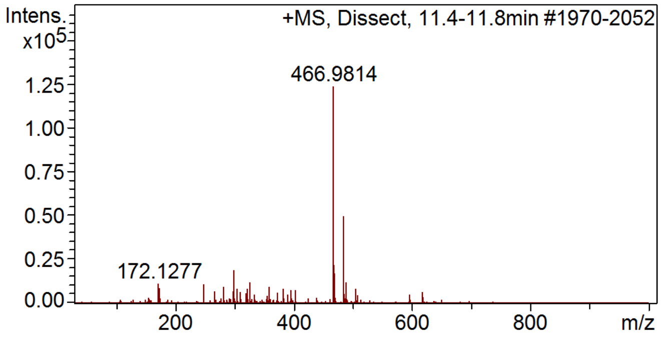

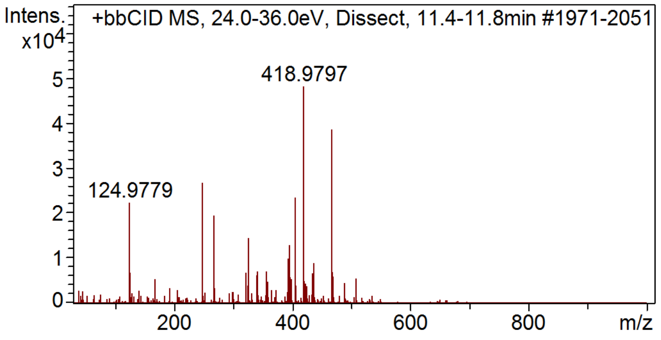

#	m/z	Res.	S/N	I	I %	FWHM
1	172.1277	27048	91.7	11359	9.2	0.0064
2	248.1905	30093	88.5	10954	8.8	0.0082
3	299.2676	30620	155.2	19212	15.5	0.0098
4	326.3674	32481	98.9	12248	9.9	0.0100
5	359.2437	31152	77.7	9619	7.8	0.0115
6	466.9814	34362	999.9	123791	100.0	0.0136
7	467.9842	31169	179.2	22187	17.9	0.0150
8	468.9779	28595	139.8	17312	14.0	0.0164
9	484.0075	32676	403.2	49915	40.3	0.0148
10	488.9628	30704	98.4	12179	9.8	0.0159

#	m/z	Res.	S/N	I	I %	FWHM
1	124.9779	24493	463.7	22351	46.4	0.0051
2	248.9722	29871	555.0	26750	55.5	0.0083
3	268.0314	30863	404.4	19491	40.5	0.0087
4	326.3674	33553	300.9	14501	30.1	0.0097
5	393.2479	31819	195.9	9443	19.6	0.0124
6	393.3024	31177	208.6	10055	20.9	0.0126
7	395.2635	30249	269.2	12975	26.9	0.0131
8	404.9646	32219	488.3	23534	48.9	0.0126
9	418.9797	32364	999.4	48169	100.0	0.0129
10	466.9815	32699	801.2	38616	80.2	0.0143

Compound Spectrum List Report

Cmpd 94, Dissect, 11.6 min

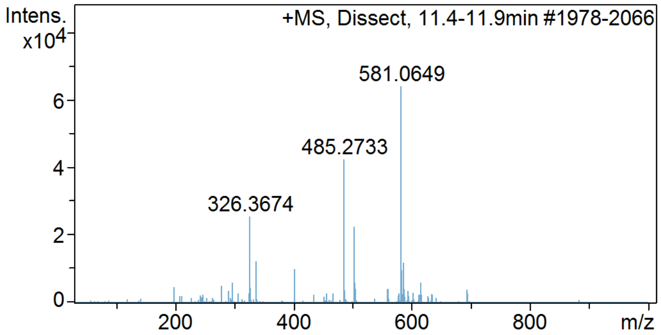

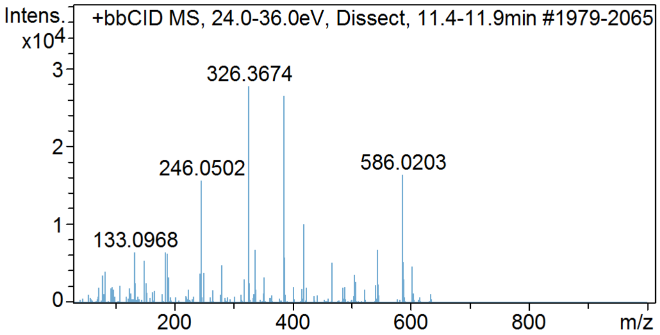

#	m/z	Res.	S/N	I	I %	FWHM
1	326.3674	32651	400.1	25645	40.1	0.0100
2	337.1325	29854	192.8	12355	19.3	0.0113
3	402.1422	31220	158.4	10152	15.9	0.0129
4	485.0099	29891	136.1	8721	13.6	0.0162
5	485.2733	31788	661.6	42407	66.3	0.0153
6	502.2991	31785	352.9	22620	35.3	0.0158
7	581.0649	32501	998.6	64002	100.0	0.0179
8	582.0677	32169	326.9	20951	32.7	0.0181
9	583.0630	26645	152.7	9789	15.3	0.0219
10	586.0200	31118	186.9	11979	18.7	0.0188

#	m/z	Res.	S/N	I	I %	FWHM
1	133.0968	24203	234.5	6501	23.4	0.0055
2	186.2153	28154	233.0	6462	23.3	0.0066
3	189.1059	26666	228.0	6321	22.8	0.0071
4	246.0502	29057	565.1	15672	56.5	0.0085
5	326.3674	33663	999.9	27728	100.0	0.0097
6	337.1323	31700	247.3	6858	24.7	0.0106
7	386.0388	31850	955.5	26496	95.6	0.0121
8	418.9796	32207	363.1	10068	36.3	0.0130
9	544.0327	32129	245.4	6805	24.5	0.0169
10	586.0203	30343	591.5	16404	59.2	0.0193

Cmpd 95, Dissect, 11.7 min

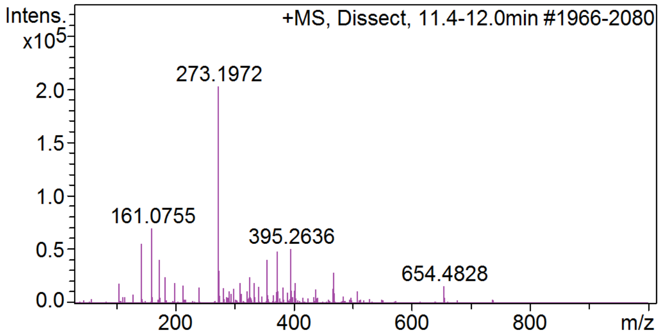

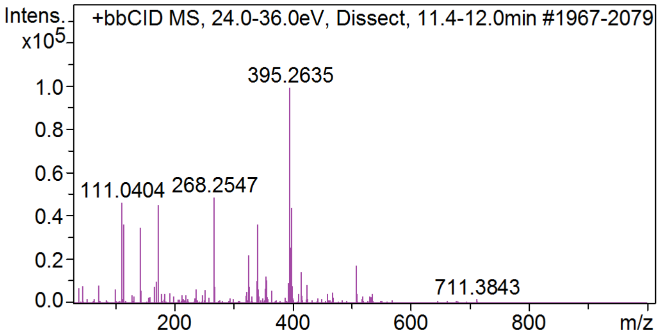

#	m/z	Res.	S/N	I	I %	FWHM
1	143.0656	25232	275.7	55797	27.6	0.0057
2	161.0755	26992	345.8	69976	34.6	0.0060
3	174.1220	27557	200.9	40658	20.1	0.0063
4	273.1972	32166	999.7	202332	100.0	0.0085
5	274.2006	30983	151.8	30715	15.2	0.0088
6	326.3674	32481	121.4	24570	12.1	0.0100
7	355.2727	32084	201.5	40774	20.2	0.0111
8	373.2825	31957	240.1	48590	24.0	0.0117
9	395.2636	31631	252.2	51032	25.2	0.0125
10	468.3525	31681	142.7	28888	14.3	0.0148

#	m/z	Res.	S/N	I	I %	FWHM
1	111.0404	22117	467.5	46417	46.8	0.0050
2	115.0716	22520	365.2	36263	36.5	0.0051
3	143.0656	25073	351.2	34868	35.1	0.0057
4	174.1219	27434	453.3	45004	45.3	0.0063
5	268.2547	32041	491.5	48799	49.2	0.0084
6	326.3674	33464	223.5	22190	22.4	0.0098
7	341.2323	32943	364.8	36218	36.5	0.0104
8	395.2635	32798	999.6	99248	100.0	0.0121
9	396.2669	31727	258.8	25700	25.9	0.0125
10	399.1652	31537	443.6	44042	44.4	0.0127

Compound Spectrum List Report

Cmpd 96, Dissect, 11.9 min

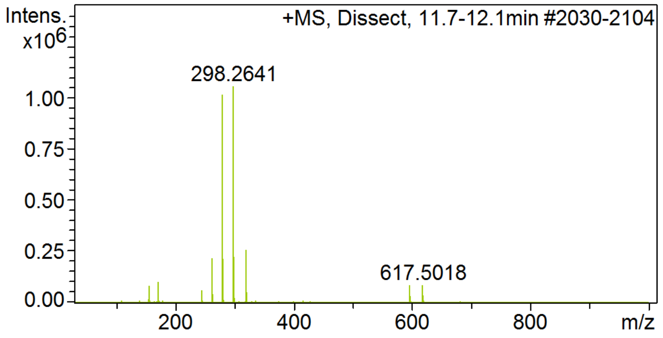

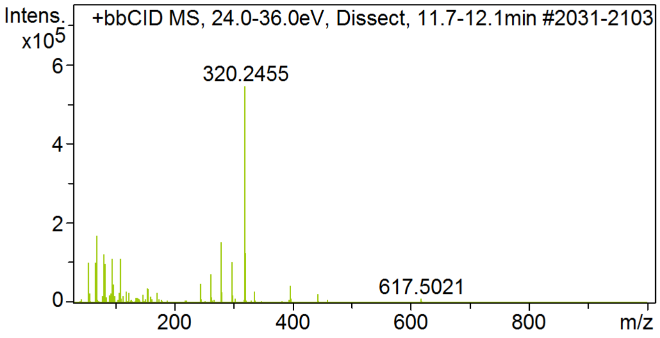

#	m/z	Res.	S/N	I	I %	FWHM
1	172.1275	27894	96.8	103479	9.8	0.0062
2	263.2283	33306	204.3	218335	20.7	0.0079
3	280.2541	37161	950.8	1016296	96.3	0.0075
4	281.2382	32975	123.6	132112	12.5	0.0085
5	281.2576	33047	203.5	217489	20.6	0.0085
6	298.2641	37982	987.2	1055183	100.0	0.0079
7	299.2676	33819	210.9	225399	21.4	0.0088
8	320.2455	36101	243.4	260113	24.7	0.0089
9	595.5206	33386	81.7	87358	8.3	0.0178
10	617.5018	33707	81.9	87520	8.3	0.0183

#	m/z	Res.	S/N	I	I %	FWHM
1	55.0520	17717	186.6	101728	18.7	0.0031
2	67.0518	18437	185.8	101319	18.6	0.0036
3	69.0673	18880	309.7	168851	31.0	0.0037
4	81.0670	20023	225.9	123199	22.6	0.0040
5	95.0824	20968	204.8	111692	20.5	0.0045
6	109.0976	22213	205.5	112059	20.6	0.0049
7	280.2544	33156	281.7	153604	28.2	0.0085
8	298.2645	32286	190.6	103927	19.1	0.0092
9	320.2455	37371	999.5	544971	100.0	0.0086
10	321.2489	34084	232.3	126673	23.2	0.0094

Cmpd 97, Dissect, 12.0 min

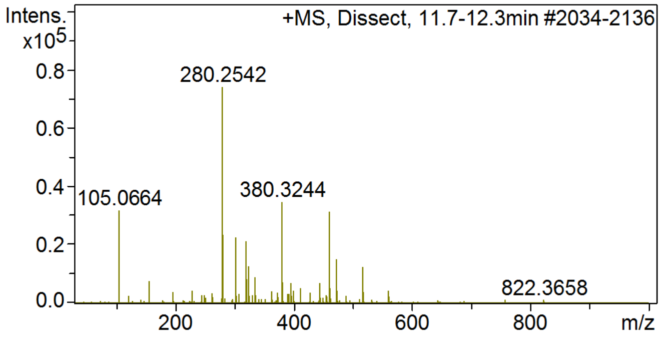

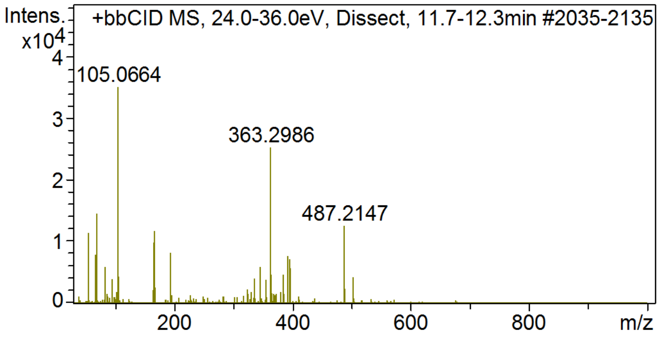

#	m/z	Res.	S/N	I	I %	FWHM
1	105.0664	21593	427.0	31753	42.9	0.0049
2	280.2542	36497	995.6	74037	100.0	0.0077
3	281.2577	32489	315.6	23466	31.7	0.0087
4	303.1644	31432	303.3	22555	30.5	0.0096
5	320.1905	35102	286.6	21314	28.8	0.0091
6	320.2457	35193	192.7	14328	19.4	0.0091
7	324.2792	32342	173.1	12871	17.4	0.0100
8	380.3244	31919	465.7	34628	46.8	0.0119
9	460.2784	31031	423.1	31464	42.5	0.0148
10	472.3111	30202	204.7	15221	20.6	0.0156

#	m/z	Res.	S/N	I	I %	FWHM
1	55.0520	17592	325.8	11444	32.6	0.0031
2	67.0518	18307	226.5	7953	22.7	0.0037
3	69.0673	18740	415.4	14590	41.6	0.0037
4	105.0664	21401	999.6	35108	100.0	0.0049
5	167.1010	27915	281.9	9901	28.2	0.0060
6	167.1374	26982	334.8	11760	33.5	0.0062
7	195.1161	27322	234.6	8241	23.5	0.0071
8	363.2986	32005	717.5	25200	71.8	0.0114
9	392.3003	32331	221.2	7770	22.1	0.0121
10	487.2147	31504	359.9	12639	36.0	0.0155

Compound Spectrum List Report

Cmpd 98, Dissect, 12.1 min

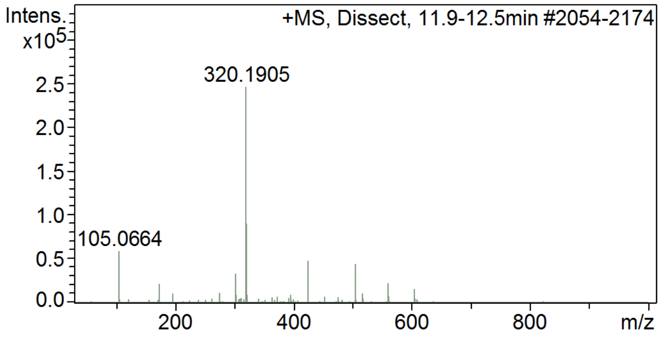

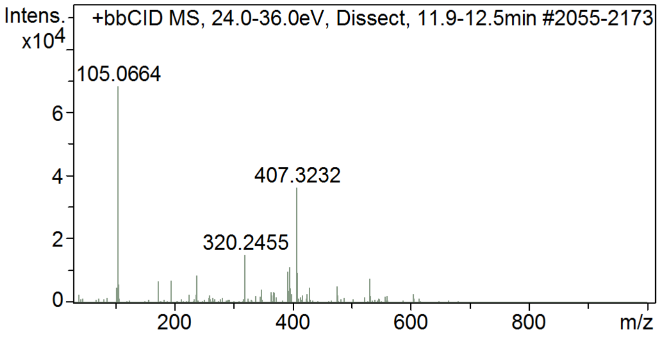

#	m/z	Res.	S/N	I	I %	FWHM
1	105.0664	21637	240.8	59456	24.1	0.0049
2	174.1067	27637	91.2	22523	9.1	0.0063
3	303.1645	31462	138.3	34161	13.9	0.0096
4	320.1905	35199	997.6	246346	100.0	0.0091
5	320.2459	33625	202.5	49993	20.3	0.0095
6	321.1940	32961	366.0	90388	36.7	0.0097
7	424.3491	32534	194.7	48087	19.5	0.0130
8	504.3034	31813	182.2	44994	18.3	0.0159
9	560.3606	31846	94.1	23231	9.4	0.0176
10	604.3854	30997	65.5	16178	6.6	0.0195

#	m/z	Res.	S/N	I	I %	FWHM
1	105.0664	21523	999.7	68231	100.0	0.0049
2	174.1068	26659	100.2	6842	10.0	0.0065
3	196.0881	27926	103.7	7081	10.4	0.0070
4	239.1411	27987	128.1	8742	12.8	0.0085
5	320.2455	34553	223.8	15277	22.4	0.0093
6	392.3004	31593	146.8	10018	14.7	0.0124
7	395.2636	32826	167.1	11403	16.7	0.0120
8	407.3232	33150	531.8	36295	53.2	0.0123
9	408.3265	32376	139.7	9534	14.0	0.0126
10	531.2397	31834	113.5	7744	11.3	0.0167

Cmpd 99, Dissect, 12.1 min

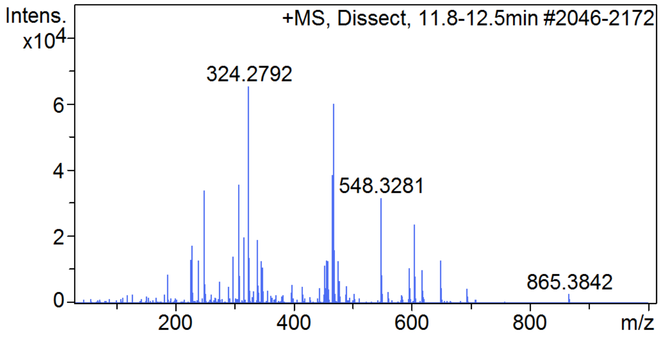

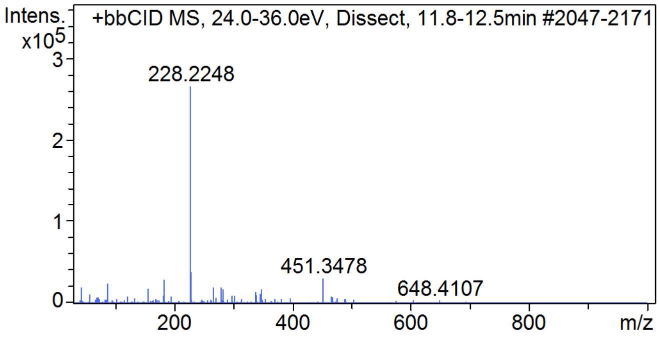

#	m/z	Res.	S/N	I	I %	FWHM
1	250.2060	29754	518.6	33857	51.9	0.0084
2	308.2848	31729	546.5	35679	54.7	0.0097
3	316.2743	32247	305.8	19962	30.6	0.0098
4	324.2792	33607	999.6	65260	100.0	0.0096
5	339.1465	29935	278.3	18170	27.8	0.0113
6	339.2587	27273	291.9	19055	29.2	0.0124
7	466.1461	31337	590.3	38536	59.0	0.0149
8	468.3735	32657	917.9	59925	91.8	0.0143
9	548.3281	30645	483.4	31559	48.4	0.0179
10	604.3853	30735	362.3	23656	36.2	0.0197

#	m/z	Res.	S/N	I	I %	FWHM
1	43.0161	15470	74.9	19946	7.5	0.0028
2	43.0524	15304	73.1	19468	7.3	0.0028
3	88.0726	19613	91.6	24370	9.2	0.0045
4	156.0968	26032	68.6	18254	6.9	0.0060
5	184.0933	27051	110.0	29289	11.0	0.0068
6	228.2248	30617	998.1	265631	100.0	0.0075
7	229.2283	27516	146.6	39016	14.7	0.0083
8	267.1770	30350	74.9	19946	7.5	0.0088
9	280.2545	32763	76.0	20227	7.6	0.0086
10	451.3478	31388	114.8	30548	11.5	0.0144

Compound Spectrum List Report

Cmpd 100, Dissect, 12.2 min

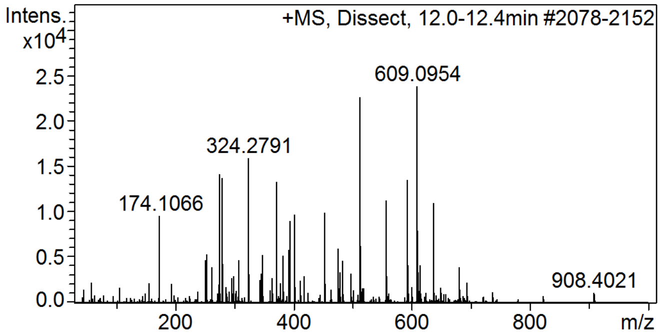

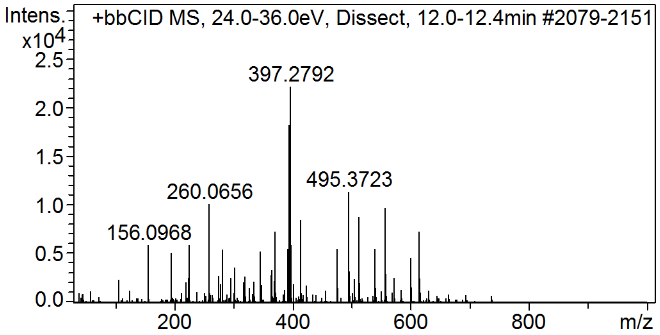

#	m/z	Res.	S/N	I	I %	FWHM
1	276.2230	30315	593.7	14138	59.4	0.0091
2	280.2544	32624	575.0	13693	57.5	0.0086
3	324.2791	33284	668.3	15914	66.8	0.0097
4	372.3349	31217	558.6	13303	55.9	0.0119
5	453.3533	32812	416.7	9923	41.7	0.0138
6	512.3981	31085	947.5	22563	94.8	0.0165
7	556.4228	30975	472.8	11259	47.3	0.0180
8	592.3525	31445	568.3	13533	56.8	0.0188
9	609.0954	32033	1000.0	23813	100.0	0.0190
10	636.3775	31571	460.6	10967	46.1	0.0202

#	m/z	Res.	S/N	I	I %	FWHM
1	260.0656	30824	455.1	10098	45.5	0.0084
2	371.1504	31380	329.0	7300	32.9	0.0118
3	394.3160	31239	818.2	18153	81.9	0.0126
4	397.2792	31776	999.3	22171	100.0	0.0125
5	398.2824	30729	268.4	5955	26.9	0.0130
6	414.0688	31755	383.1	8500	38.3	0.0130
7	495.3723	31894	512.4	11369	51.3	0.0155
8	512.3984	31393	396.0	8787	39.6	0.0163
9	556.4230	31053	437.6	9708	43.8	0.0179
10	614.0502	30305	328.6	7291	32.9	0.0203

Cmpd 101, Dissect, 12.3 min

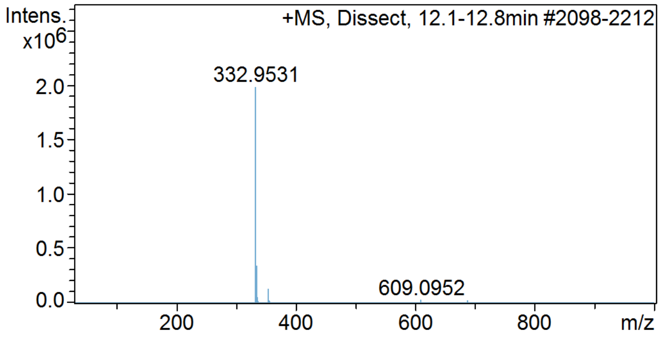

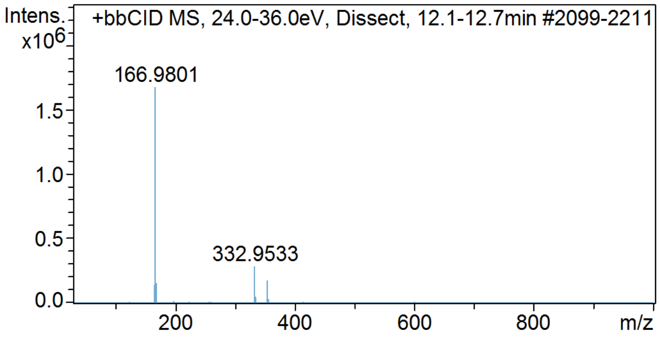

#	m/z	Res.	S/N	I	I %	FWHM
1	332.9531	40402	991.5	1983143	100.0	0.0082
2	333.9559	34805	172.7	345333	17.4	0.0096
3	334.9493	35190	175.5	350930	17.7	0.0095
4	335.9518	29288	28.8	57519	2.9	0.0115
5	336.9454	26440	11.6	23264	1.2	0.0127
6	354.9345	35235	67.7	135500	6.8	0.0101
7	355.9370	30024	11.7	23442	1.2	0.0119
8	356.9302	30219	11.2	22370	1.1	0.0118
9	609.0952	31771	15.3	30674	1.5	0.0192
10	686.8804	30676	13.0	26010	1.3	0.0224

#	m/z	Res.	S/N	I	I %	FWHM
1	165.9725	28008	82.0	143954	8.6	0.0059
2	166.9801	30686	954.9	1676024	100.0	0.0054
3	167.9831	25182	80.9	142021	8.5	0.0067
4	168.9760	28409	92.6	162610	9.7	0.0059
5	332.9533	37715	165.7	290921	17.4	0.0088
6	333.9559	31868	29.3	51474	3.1	0.0105
7	334.9493	32258	27.7	48545	2.9	0.0104
8	354.9346	35080	102.3	179564	10.7	0.0101
9	355.9371	31408	19.2	33774	2.0	0.0113
10	356.9306	30639	18.9	33198	2.0	0.0116

Compound Spectrum List Report

Cmpd 102, Dissect, 12.4 min

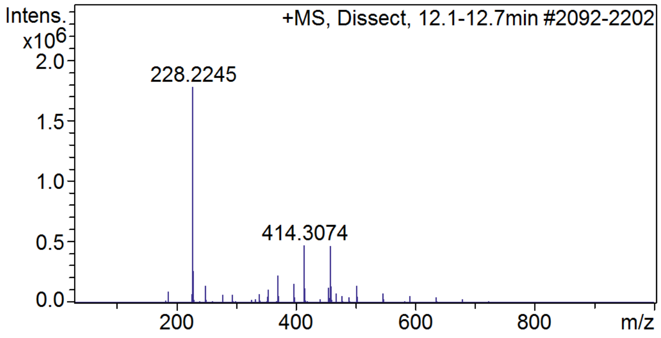

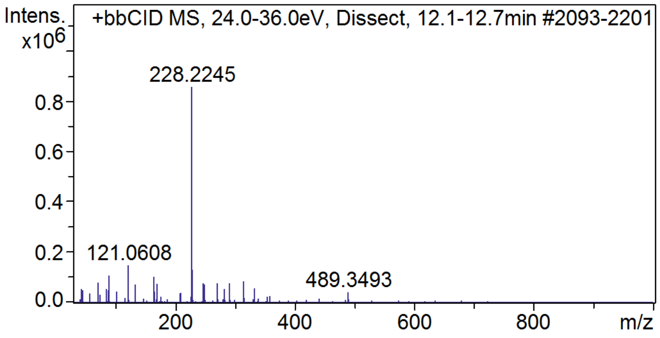

#	m/z	Res.	S/N	I	I %	FWHM
1	228.2245	34198	997.7	1777883	100.0	0.0067
2	229.2279	31565	149.2	265796	15.0	0.0073
3	250.2058	31782	81.4	145053	8.2	0.0079
4	370.2829	34434	127.9	227943	12.8	0.0108
5	397.2815	33411	90.8	161751	9.1	0.0119
6	414.3074	38260	266.9	475516	26.7	0.0108
7	455.4415	34202	73.4	130707	7.4	0.0133
8	458.3321	39052	264.6	471425	26.5	0.0117
9	459.3355	34557	77.6	138254	7.8	0.0133
10	502.3569	36153	81.6	145423	8.2	0.0139

#	m/z	Res.	S/N	I	I %	FWHM
1	71.0829	19133	94.0	81038	9.4	0.0037
2	89.0567	20090	128.7	110947	12.9	0.0044
3	121.0608	23834	174.4	150384	17.5	0.0051
4	165.0855	27462	121.9	105124	12.3	0.0060
5	228.2245	33842	995.1	857884	100.0	0.0067
6	229.2279	30389	153.1	131977	15.4	0.0075
7	247.1975	31185	92.3	79537	9.3	0.0079
8	271.1451	32106	92.4	79635	9.3	0.0084
9	291.2223	31337	93.8	80896	9.4	0.0093
10	315.1698	33687	102.3	88222	10.3	0.0094

Cmpd 103, Dissect, 12.5 min

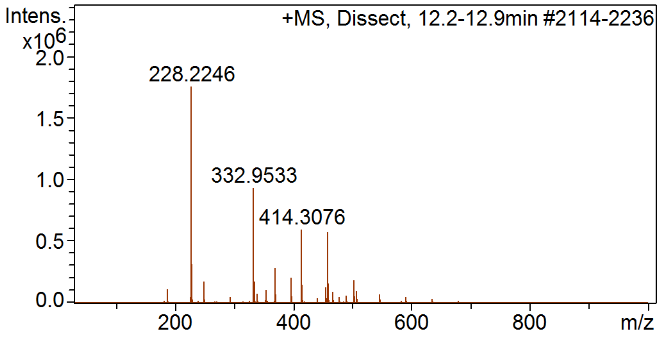

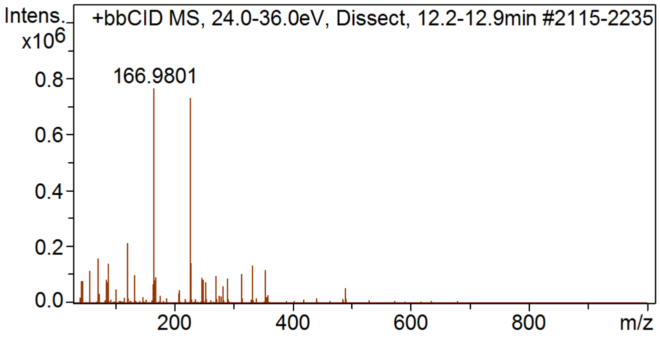

#	m/z	Res.	S/N	I	I %	FWHM
1	228.2246	33838	998.2	1754684	100.0	0.0067
2	229.2280	30862	179.5	315536	18.0	0.0074
3	250.2059	31247	101.8	178918	10.2	0.0080
4	332.9533	38854	532.5	936051	53.3	0.0086
5	334.9493	33294	101.3	178155	10.2	0.0101
6	370.2831	34510	163.6	287677	16.4	0.0107
7	397.2817	34690	120.4	211738	12.1	0.0115
8	414.3076	39319	339.1	596097	34.0	0.0105
9	458.3323	40935	328.8	577949	32.9	0.0112
10	502.3571	37600	105.9	186234	10.6	0.0134

#	m/z	Res.	S/N	I	I %	FWHM
1	57.0676	17496	153.7	117970	15.4	0.0033
2	71.0829	19238	207.8	159532	20.8	0.0037
3	89.0567	20343	184.4	141597	18.5	0.0044
4	121.0608	23935	279.2	214365	28.0	0.0051
5	166.9801	29947	997.8	766020	100.0	0.0056
6	228.2246	33184	949.6	729000	95.2	0.0069
7	229.2280	29898	189.4	145420	19.0	0.0077
8	315.1700	34777	138.0	105945	13.8	0.0091
9	332.9534	35751	176.2	135255	17.7	0.0093
10	354.9346	34069	156.6	120192	15.7	0.0104

Compound Spectrum List Report

Cmpd 104, Dissect, 12.5 min

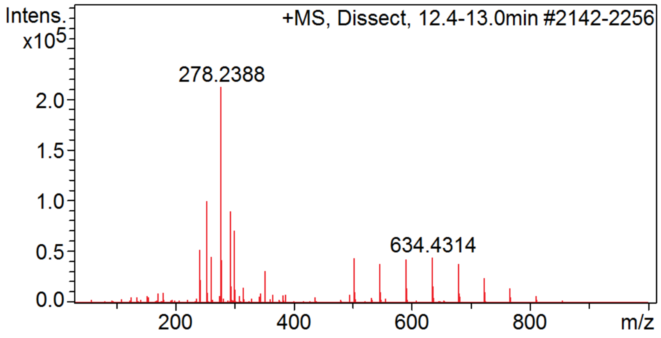

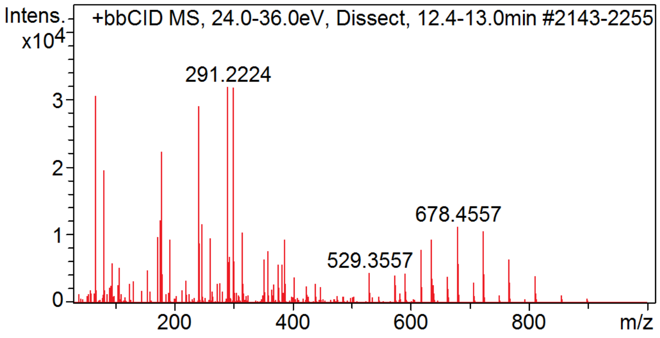

#	m/z	Res.	S/N	I	I %	FWHM
1	242.2399	32326	247.2	52513	24.8	0.0075
2	254.2393	33914	469.8	99815	47.0	0.0075
3	261.2129	30245	214.1	45479	21.4	0.0086
4	278.2388	32320	998.7	212169	100.0	0.0086
5	279.2422	30277	200.3	42549	20.1	0.0092
6	294.2332	31837	424.5	90177	42.5	0.0092
7	300.2200	32375	333.8	70921	33.4	0.0093
8	502.3570	37171	207.6	44107	20.8	0.0135
9	590.4066	33539	203.3	43196	20.4	0.0176
10	634.4314	32509	212.3	45101	21.3	0.0195

#	m/z	Res.	S/N	I	I %	FWHM
1	67.0518	18002	954.3	30455	95.4	0.0037
2	81.0670	19653	612.8	19555	61.3	0.0041
3	177.1063	28020	381.8	12183	38.2	0.0063
4	179.1371	27485	699.5	22323	70.0	0.0065
5	242.2398	32524	907.8	28970	90.8	0.0074
6	247.1976	31612	365.1	11653	36.5	0.0078
7	291.2224	32149	999.9	31908	100.0	0.0091
8	300.2200	31483	996.6	31805	99.7	0.0095
9	678.4557	30965	351.4	11213	35.1	0.0219
10	722.4805	30799	331.6	10582	33.2	0.0235

Cmpd 105, Dissect, 12.7 min

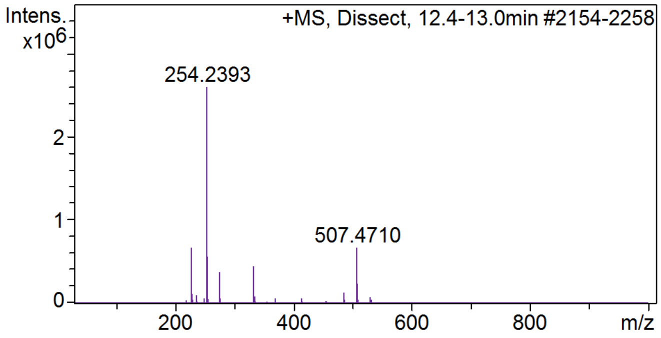

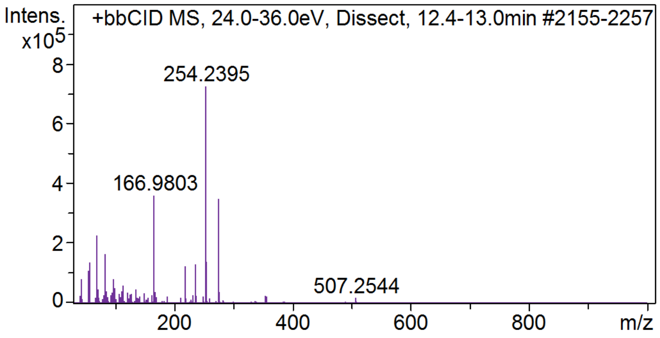

#	m/z	Res.	S/N	I	I %	FWHM
1	228.1883	29044	59.4	154943	6.0	0.0079
2	228.2247	29319	258.7	674473	25.9	0.0078
3	254.2393	34140	997.6	2601384	100.0	0.0074
4	255.2427	34517	216.6	564740	21.7	0.0074
5	276.2206	34814	146.5	382146	14.7	0.0079
6	332.9532	31032	173.1	451417	17.4	0.0107
7	333.2887	30012	67.4	175800	6.8	0.0111
8	485.2731	34332	52.1	135779	5.2	0.0141
9	507.4710	43603	257.6	671667	25.8	0.0116
10	508.4742	37828	92.2	240419	9.2	0.0134

#	m/z	Res.	S/N	I	I %	FWHM
1	55.0520	17783	153.0	110851	15.3	0.0031
2	57.0676	17598	189.1	136960	18.9	0.0032
3	69.0673	19051	314.5	227795	31.5	0.0036
4	83.0825	20387	228.8	165706	22.9	0.0041
5	166.9803	27834	497.7	360498	49.8	0.0060
6	219.2035	30512	171.9	124537	17.2	0.0072
7	237.2135	31565	180.0	130408	18.0	0.0075
8	254.2395	34714	999.9	724257	100.0	0.0073
9	255.2428	31981	192.9	139730	19.3	0.0080
10	276.2207	33674	481.3	348606	48.1	0.0082

Compound Spectrum List Report

Cmpd 106, Dissect, 12.7 min

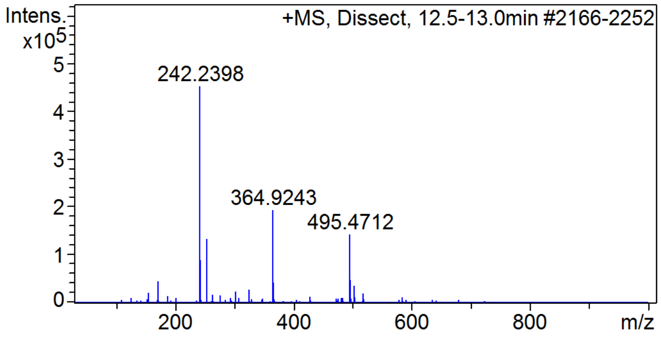

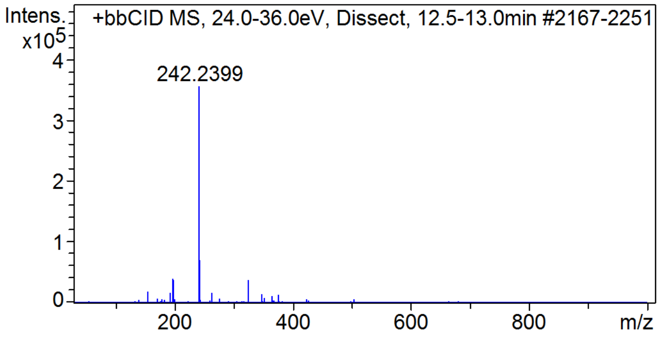

#	m/z	Res.	S/N	I	I %	FWHM
1	172.1275	27531	101.0	45817	10.1	0.0063
2	242.2398	33642	997.1	452306	100.0	0.0072
3	243.2432	29424	197.3	89505	19.8	0.0083
4	254.2393	34433	296.4	134451	29.7	0.0074
5	364.9243	35524	427.3	193846	42.9	0.0103
6	365.9267	30101	78.5	35618	7.9	0.0122
7	366.9202	30324	95.7	43418	9.6	0.0121
8	495.4712	33348	315.1	142923	31.6	0.0149
9	496.4746	31333	108.2	49065	10.8	0.0158
10	502.2990	33367	80.5	36523	8.1	0.0151

#	m/z	Res.	S/N	I	I %	FWHM
1	155.1015	25658	55.6	19852	5.6	0.0060
2	194.1088	27781	48.8	17409	4.9	0.0070
3	197.9436	28946	112.8	40289	11.3	0.0068
4	198.9511	27423	107.0	38219	10.7	0.0073
5	242.2399	33028	996.9	355992	100.0	0.0073
6	243.2432	29384	197.2	70431	19.8	0.0083
7	264.2218	27573	48.2	17203	4.8	0.0096
8	325.2243	32424	108.7	38802	10.9	0.0100
9	348.2758	31682	40.9	14608	4.1	0.0110
10	376.3062	32709	36.4	13012	3.7	0.0115

Cmpd 107, Dissect, 12.9 min

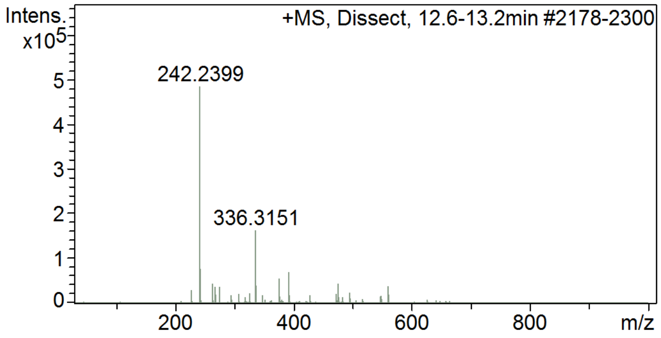

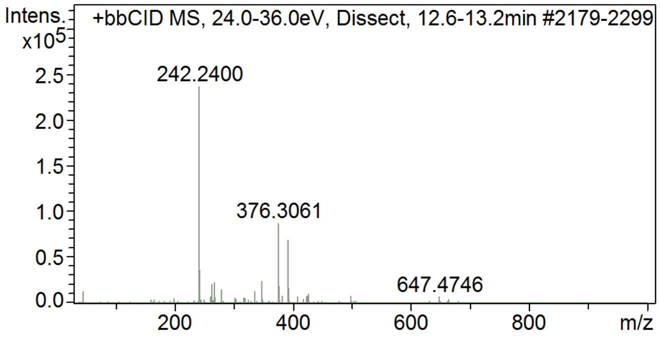

#	m/z	Res.	S/N	I	I %	FWHM
1	242.2399	33184	996.4	484798	100.0	0.0073
2	243.2433	29345	160.6	78158	16.1	0.0083
3	264.2215	28730	93.6	45561	9.4	0.0092
4	275.2492	30818	78.9	38391	7.9	0.0089
5	336.3151	32618	336.9	163910	33.8	0.0103
6	337.3184	30864	83.3	40513	8.4	0.0109
7	376.3061	32689	115.7	56279	11.6	0.0115
8	392.3005	33214	144.9	70504	14.5	0.0118
9	476.1187	31922	92.1	44809	9.2	0.0149
10	559.5008	41822	80.7	39267	8.1	0.0134

#	m/z	Res.	S/N	I	I %	FWHM
1	242.2400	31725	997.7	236451	100.0	0.0076
2	243.2433	29139	154.8	36687	15.5	0.0083
3	264.2237	27405	90.3	21404	9.1	0.0096
4	268.1844	28488	97.1	23021	9.7	0.0094
5	280.2544	32420	64.8	15349	6.5	0.0086
6	348.2758	31081	103.5	24533	10.4	0.0112
7	376.3061	33970	368.7	87372	37.0	0.0111
8	377.3092	30070	82.0	19442	8.2	0.0125
9	392.3004	33103	293.1	69454	29.4	0.0119
10	393.3033	29370	73.5	17427	7.4	0.0134

Compound Spectrum List Report

Cmpd 108, Dissect, 12.9 min

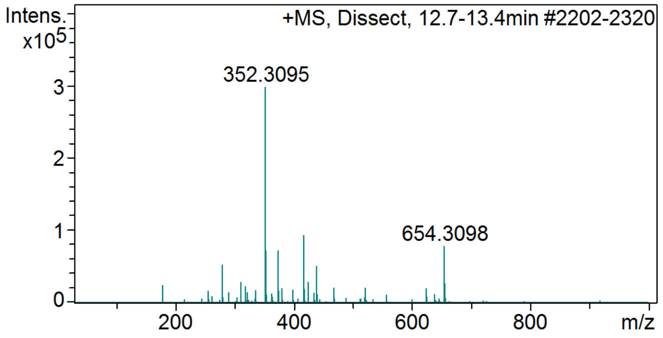

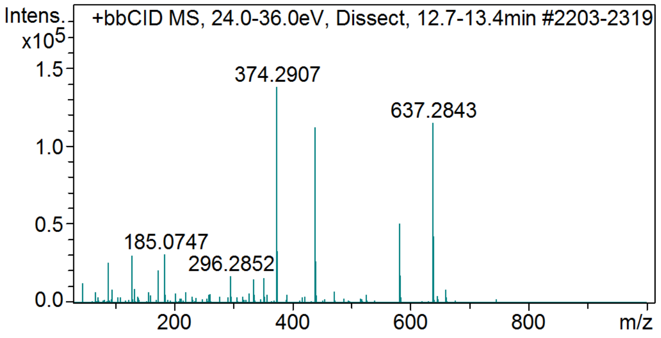

#	m/z	Res.	S/N	I	I %	FWHM
1	280.2542	34971	178.8	53483	17.9	0.0080
2	311.1540	31459	98.4	29439	9.9	0.0099
3	352.3095	35894	998.7	298649	100.0	0.0098
4	353.3128	32408	242.9	72624	24.3	0.0109
5	374.2905	33342	244.2	73036	24.5	0.0112
6	417.2708	33612	314.6	94072	31.5	0.0124
7	424.3491	31634	99.2	29677	9.9	0.0134
8	439.2518	32967	171.7	51349	17.2	0.0133
9	654.3098	32843	264.8	79176	26.5	0.0199
10	655.3132	31088	93.8	28044	9.4	0.0211

#	m/z	Res.	S/N	I	I %	FWHM
1	89.0567	19534	186.7	25799	18.7	0.0046
2	129.0140	24384	220.7	30493	22.1	0.0053
3	185.0747	28775	227.2	31388	22.7	0.0064
4	374.2907	35122	999.3	138070	100.0	0.0107
5	375.2939	32010	241.9	33428	24.2	0.0117
6	439.2520	35205	811.2	112082	81.2	0.0125
7	440.2553	31382	193.2	26699	19.3	0.0140
8	581.2234	32614	367.9	50837	36.8	0.0178
9	637.2843	34701	832.0	114949	83.3	0.0184
10	638.2875	31337	307.7	42516	30.8	0.0204

Cmpd 109, Dissect, 13.1 min

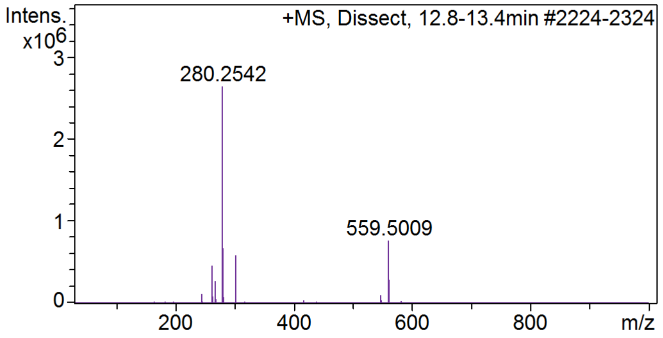

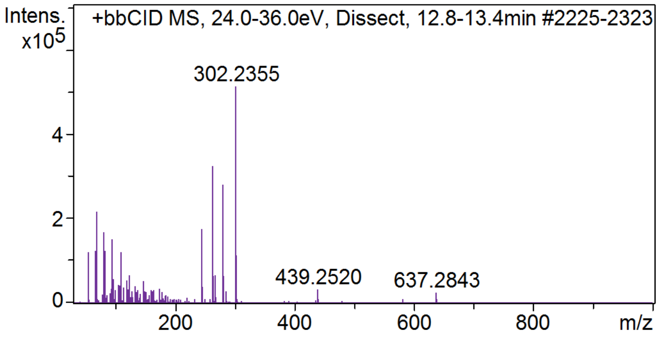

#	m/z	Res.	S/N	I	I %	FWHM
1	245.2184	30605	41.4	110119	4.2	0.0080
2	263.2284	34872	174.4	463881	17.5	0.0075
3	268.2546	35831	104.6	278198	10.5	0.0075
4	280.2542	35077	993.9	2643205	100.0	0.0080
5	281.2577	36119	253.3	673729	25.5	0.0078
6	302.2355	36773	220.5	586465	22.2	0.0082
7	303.2390	32793	42.9	113996	4.3	0.0092
8	547.5010	36370	36.9	98062	3.7	0.0151
9	559.5009	46269	288.7	767671	29.0	0.0121
10	560.5041	38801	109.1	290098	11.0	0.0144

#	m/z	Res.	S/N	I	I %	FWHM
1	55.0520	17913	236.1	121654	23.7	0.0031
2	67.0518	18563	241.0	124142	24.2	0.0036
3	69.0673	19192	421.2	217014	42.3	0.0036
4	81.0670	20432	327.0	168453	32.8	0.0040
5	83.0826	20494	243.1	125232	24.4	0.0041
6	95.0824	21458	296.3	152629	29.7	0.0044
7	245.2184	31938	343.2	176801	34.5	0.0077
8	263.2283	34385	630.3	324730	63.3	0.0077
9	280.2543	34861	543.4	279968	54.6	0.0080
10	302.2355	35669	996.1	513186	100.0	0.0085

Compound Spectrum List Report

Cmpd 110, Dissect, 13.2 min

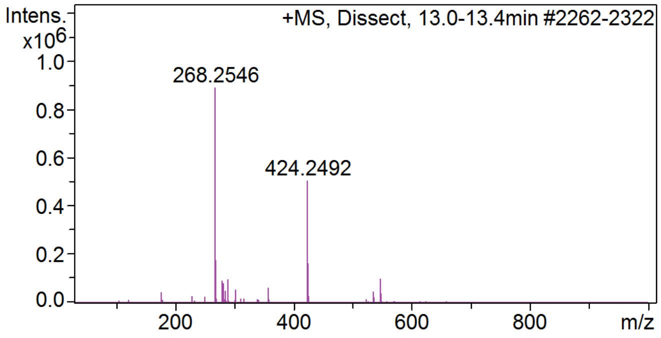

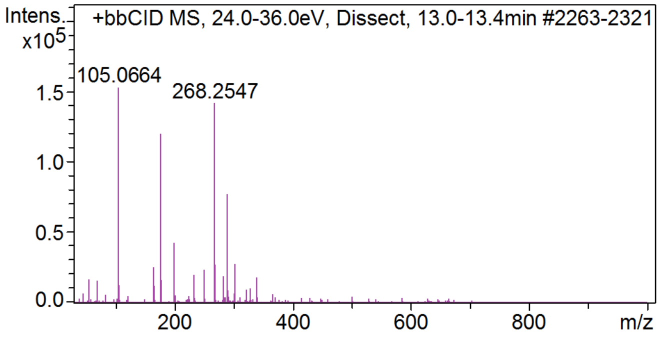

#	m/z	Res.	S/N	I	I %	FWHM
1	268.2546	36155	997.1	891385	100.0	0.0074
2	269.2580	32827	198.1	177111	19.9	0.0082
3	280.2541	36906	104.7	93638	10.5	0.0076
4	282.0347	31325	93.1	83242	9.3	0.0090
5	290.2359	32844	111.9	100075	11.2	0.0088
6	302.2355	33978	64.5	57706	6.5	0.0089
7	358.2621	32252	71.7	64062	7.2	0.0111
8	424.2492	39696	564.7	504787	56.6	0.0107
9	425.2525	35470	185.0	165356	18.6	0.0120
10	547.5010	35491	115.0	102816	11.5	0.0154

#	m/z	Res.	S/N	I	I %	FWHM
1	105.0664	21888	998.5	153033	100.0	0.0048
2	165.9725	27016	165.6	25381	16.6	0.0061
3	177.1215	28477	780.6	119627	78.2	0.0062
4	199.9591	28984	280.0	42916	28.0	0.0069
5	233.2187	29920	131.6	20171	13.2	0.0078
6	251.2287	30466	154.5	23674	15.5	0.0082
7	268.2547	33096	922.7	141420	92.4	0.0081
8	269.2580	30882	177.7	27238	17.8	0.0087
9	290.2360	33460	504.8	77362	50.6	0.0087
10	302.2355	32743	180.8	27710	18.1	0.0092

Cmpd 111, Dissect, 13.4 min

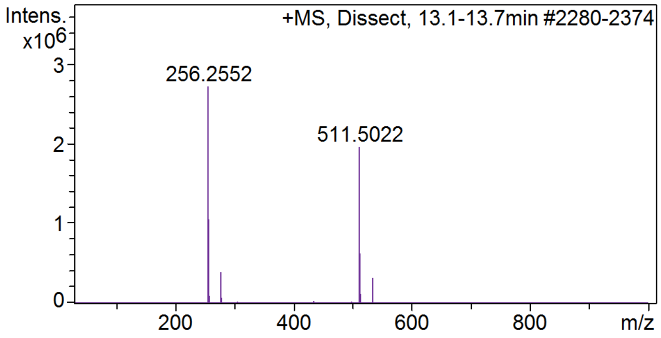

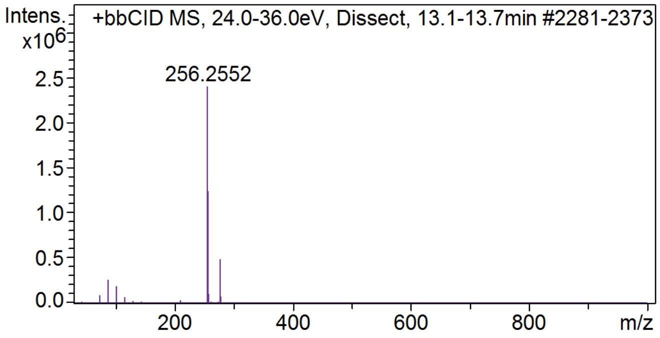

#	m/z	Res.	S/N	I	I %	FWHM
1	256.2552	21800	993.7	2722522	100.0	0.0118
2	257.2583	35916	384.2	1052705	38.7	0.0072
3	258.2614	30310	34.3	94046	3.5	0.0085
4	278.2362	34997	143.8	393877	14.5	0.0080
5	279.2396	29695	24.3	66561	2.4	0.0094
6	511.5022	48588	715.6	1960440	72.0	0.0105
7	512.5056	43083	229.3	628320	23.1	0.0119
8	513.5086	33922	41.5	113820	4.2	0.0151
9	533.4834	40458	119.6	327668	12.0	0.0132
10	534.4866	33530	42.0	114972	4.2	0.0159

#	m/z	Res.	S/N	I	I %	FWHM
1	74.0573	19153	37.2	91375	3.8	0.0039
2	88.0725	21240	109.3	268398	11.2	0.0041
3	102.0879	21966	79.4	195161	8.1	0.0046
4	116.1031	22703	26.4	64918	2.7	0.0051
5	211.1159	30177	11.9	29338	1.2	0.0070
6	256.2552	19049	977.5	2401392	100.0	0.0135
7	257.2582	36173	506.2	1243561	51.8	0.0071
8	258.2613	30548	45.7	112242	4.7	0.0085
9	278.2361	36062	200.4	492285	20.5	0.0077
10	279.2395	30834	31.5	77314	3.2	0.0091

Compound Spectrum List Report

Cmpd 112, Dissect, 13.5 min

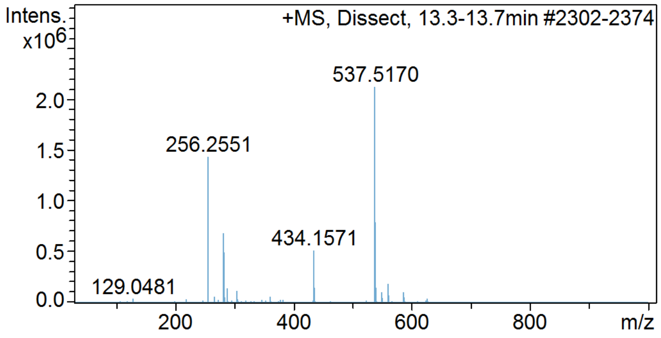

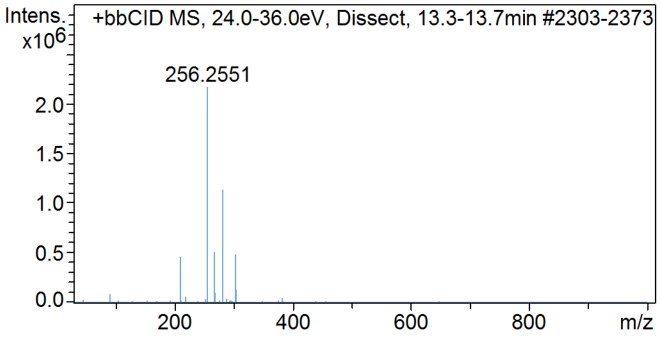

#	m/z	Res.	S/N	I	I %	FWHM
1	256.2551	25888	672.3	1429099	67.4	0.0099
2	282.2700	25126	322.3	684969	32.3	0.0112
3	283.2730	36528	233.8	496868	23.4	0.0078
4	288.1653	30824	68.7	145973	6.9	0.0093
5	434.1571	35503	243.8	518213	24.4	0.0122
6	435.1600	31276	70.8	150440	7.1	0.0139
7	537.5170	44639	997.5	2120344	100.0	0.0120
8	538.5202	38128	372.0	790776	37.3	0.0141
9	539.5234	31125	71.4	151708	7.2	0.0173
10	559.4978	32095	91.2	193903	9.1	0.0174

#	m/z	Res.	S/N	I	I %	FWHM
1	91.0511	20120	40.9	88894	4.1	0.0045
2	211.1159	30462	214.7	467253	21.5	0.0069
3	256.2551	23074	998.6	2172795	100.0	0.0111
4	268.1844	32061	238.7	519479	23.9	0.0084
5	268.2546	33024	237.1	515804	23.7	0.0081
6	269.1878	29115	48.7	105961	4.9	0.0092
7	269.2579	29710	43.4	94435	4.3	0.0091
8	282.2698	29271	524.8	1141847	52.6	0.0096
9	304.2509	37431	225.9	491516	22.6	0.0081
10	305.2543	33743	62.5	136050	6.3	0.0090

Cmpd 113, Dissect, 13.6 min

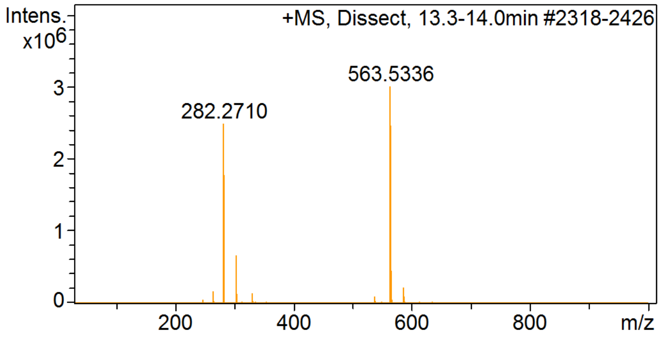

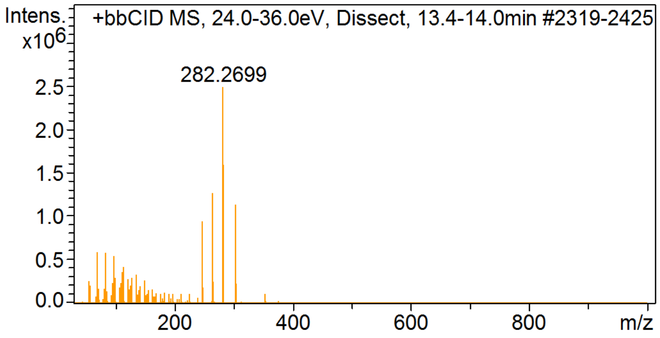

#	m/z	Res.	S/N	I	I %	FWHM
1	265.2436	32789	55.2	170274	5.7	0.0081
2	282.2710	15361	806.4	2485974	82.7	0.0184
3	283.2728	37822	577.1	1779024	59.2	0.0075
4	304.2507	38230	215.8	665431	22.1	0.0080
5	305.2541	33220	43.1	132761	4.4	0.0092
6	331.2730	36824	46.6	143542	4.8	0.0090
7	563.5336	21538	975.1	3006015	100.0	0.0262
8	564.5350	48862	796.5	2455626	81.7	0.0116
9	565.5381	42834	148.0	456194	15.2	0.0132
10	585.5127	38468	74.7	230168	7.7	0.0152

#	m/z	Res.	S/N	I	I %	FWHM
1	69.0672	19497	236.0	590068	23.7	0.0035
2	83.0824	21657	233.1	582772	23.4	0.0038
3	97.0978	22632	217.9	544808	21.8	0.0043
4	111.1130	23586	144.3	360852	14.5	0.0047
5	114.0874	23798	169.6	423985	17.0	0.0048
6	247.2336	36022	379.7	949378	38.1	0.0069
7	265.2436	36982	506.4	1266024	50.8	0.0072
8	282.2699	17937	997.4	2493632	100.0	0.0157
9	283.2729	38204	636.8	1592028	63.8	0.0074
10	304.2507	39063	454.8	1136934	45.6	0.0078

Compound Spectrum List Report

Cmpd 114, Dissect, 13.7 min

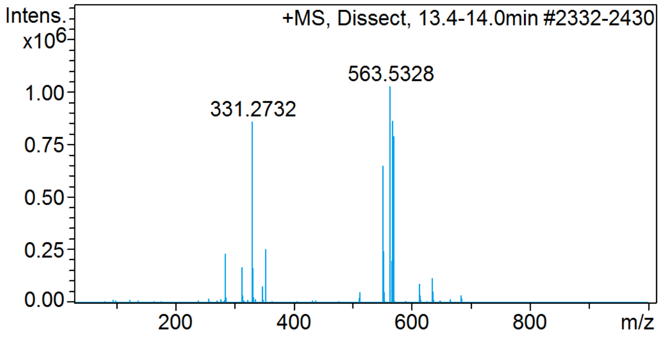

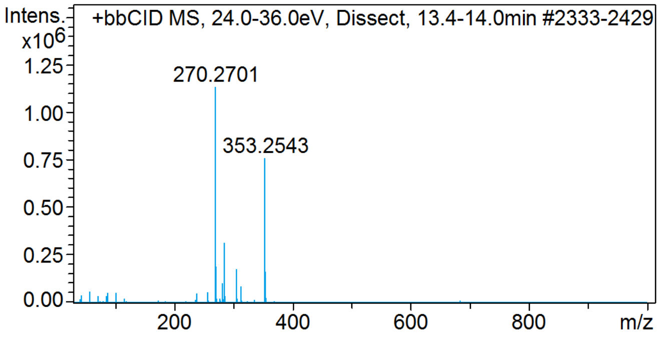

#	m/z	Res.	S/N	I	I %	FWHM
1	285.2838	24729	226.5	235153	22.9	0.0115
2	331.2732	36632	826.2	857619	83.5	0.0090
3	353.2543	34385	245.8	255196	24.9	0.0103
4	551.5322	38841	624.1	647841	63.1	0.0142
5	552.5354	33034	236.3	245268	23.9	0.0167
6	563.5328	25615	989.1	1026692	100.0	0.0220
7	567.4157	26499	205.8	213666	20.8	0.0214
8	567.4589	21720	351.9	365247	35.6	0.0261
9	567.5468	24979	828.2	859736	83.7	0.0227
10	569.5397	28555	758.4	787224	76.7	0.0199

#	m/z	Res.	S/N	I	I %	FWHM
1	57.0676	17772	55.7	63581	5.6	0.0032
2	257.2387	29604	51.9	59277	5.2	0.0087
3	270.2701	36060	991.6	1132874	100.0	0.0075
4	271.2734	32110	168.4	192389	17.0	0.0084
5	282.2698	20313	90.9	103895	9.2	0.0139
6	285.2852	25625	277.1	316543	27.9	0.0111
7	306.2579	25590	157.8	180239	15.9	0.0120
8	313.2631	31265	78.9	90163	8.0	0.0100
9	353.2543	37942	662.9	757272	66.8	0.0093
10	354.2577	32264	146.5	167335	14.8	0.0110

Cmpd 115, Dissect, 13.8 min

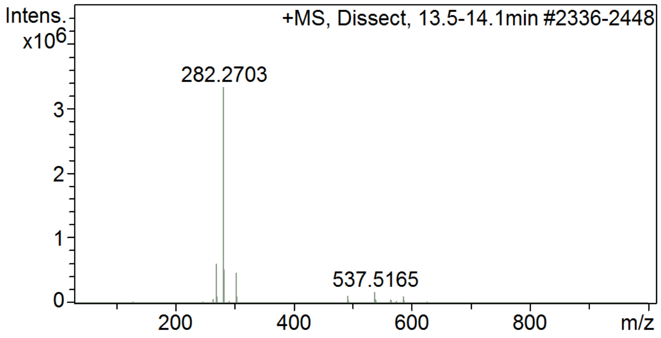

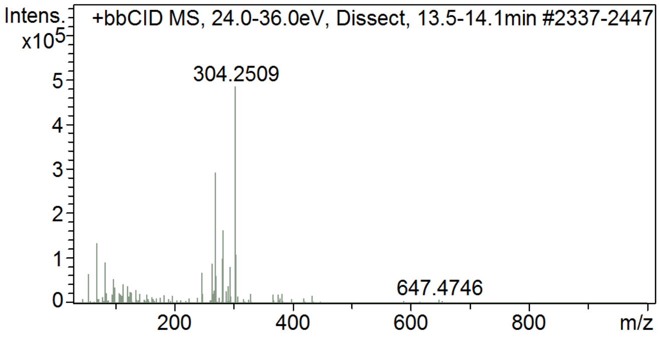

#	m/z	Res.	S/N	I	I %	FWHM
1	265.2438	32069	18.6	62433	1.9	0.0083
2	270.2702	34924	182.9	614756	18.4	0.0077
3	271.2736	31981	30.2	101681	3.0	0.0085
4	282.2703	22577	992.5	3336231	100.0	0.0125
5	283.2730	36854	157.9	530771	15.9	0.0077
6	304.2510	36865	141.2	474755	14.2	0.0083
7	305.2543	32232	30.0	100978	3.0	0.0095
8	492.2765	32646	32.3	108422	3.2	0.0151
9	537.5165	30935	52.9	177969	5.3	0.0174
10	585.5129	35468	30.7	103221	3.1	0.0165

#	m/z	Res.	S/N	I	I %	FWHM
1	69.0672	19268	279.6	135466	28.0	0.0036
2	83.0825	21132	191.6	92854	19.2	0.0039
3	247.2338	34716	144.2	69880	14.4	0.0071
4	265.2437	35943	183.5	88934	18.4	0.0074
5	270.2701	35659	604.3	292783	60.4	0.0076
6	282.2698	23282	209.1	101312	20.9	0.0121
7	283.2730	36989	338.8	164154	33.9	0.0077
8	295.1629	31160	169.0	81880	16.9	0.0095
9	304.2509	37832	999.9	484505	100.0	0.0080
10	305.2543	33864	225.8	109410	22.6	0.0090

Compound Spectrum List Report

Cmpd 116, Dissect, 13.9 min

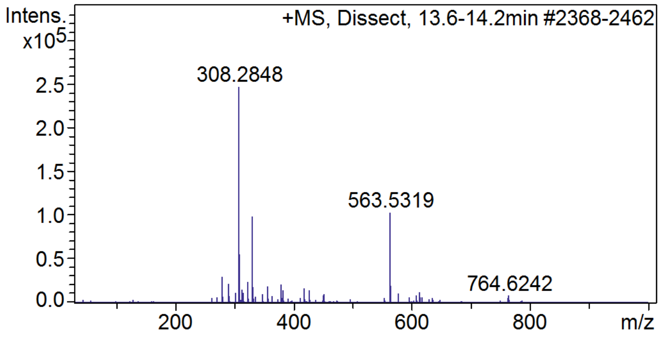

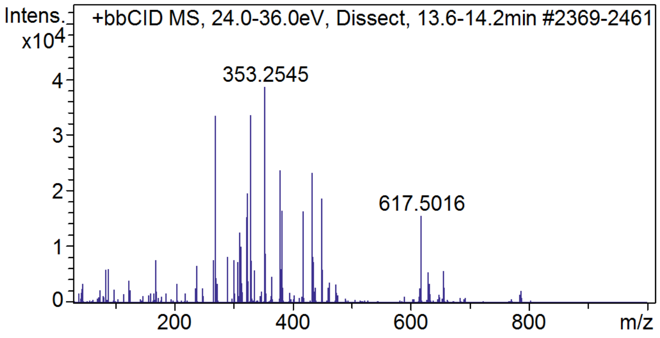

#	m/z	Res.	S/N	I	I %	FWHM
1	280.2544	32147	123.5	30568	12.4	0.0087
2	291.2587	31238	90.5	22404	9.1	0.0093
3	308.2848	33699	997.7	246945	100.0	0.0091
4	309.2881	31944	225.6	55841	22.6	0.0097
5	323.1535	31170	97.5	24139	9.8	0.0104
6	331.2717	29084	399.5	98869	40.0	0.0114
7	357.2881	30572	77.6	19205	7.8	0.0117
8	379.2691	30941	87.2	21589	8.7	0.0123
9	563.5319	39473	416.1	102984	41.7	0.0143
10	564.5351	34197	79.9	19764	8.0	0.0165

#	m/z	Res.	S/N	I	I %	FWHM
1	270.2702	34233	862.0	33354	86.2	0.0079
2	324.3153	30876	505.6	19565	50.6	0.0105
3	330.2659	31902	867.4	33562	86.7	0.0104
4	353.2545	33797	999.9	38693	100.0	0.0105
5	379.2692	31540	612.7	23708	61.3	0.0120
6	383.1913	33323	424.9	16442	42.5	0.0115
7	418.3151	30905	424.8	16439	42.5	0.0135
8	433.3642	32032	600.6	23241	60.1	0.0135
9	449.3583	31630	483.3	18702	48.3	0.0142
10	617.5016	29672	403.0	15593	40.3	0.0208

Cmpd 117, Dissect, 14.0 min

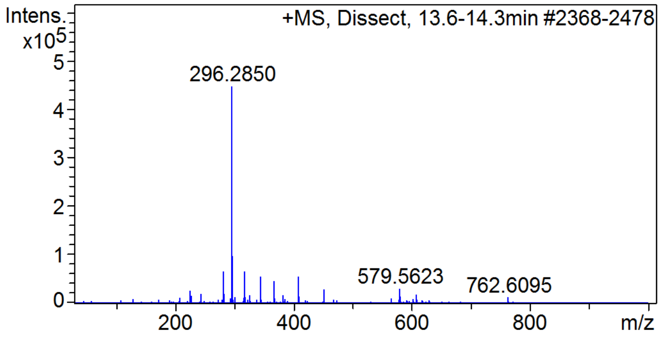

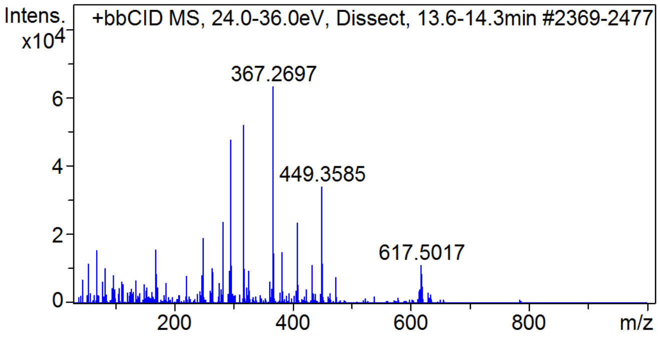

#	m/z	Res.	S/N	I	I %	FWHM
1	282.2699	32077	149.7	67081	15.0	0.0088
2	296.2486	30144	64.9	29068	6.5	0.0098
3	296.2850	35776	998.3	447205	100.0	0.0083
4	297.2885	31934	217.8	97580	21.8	0.0093
5	318.2663	31240	149.9	67172	15.0	0.0102
6	345.2884	32626	123.9	55497	12.4	0.0106
7	367.2697	31962	104.4	46757	10.5	0.0115
8	408.2947	33859	125.4	56160	12.6	0.0121
9	451.3739	31465	65.4	29296	6.6	0.0143
10	579.5623	31490	69.0	30901	6.9	0.0184

#	m/z	Res.	S/N	I	I %	FWHM
1	170.1120	27121	250.4	15848	25.1	0.0063
2	250.1696	29790	302.4	19143	30.3	0.0084
3	283.2539	29634	376.1	23806	37.6	0.0096
4	283.2731	29021	291.4	18449	29.2	0.0098
5	296.2487	30990	388.2	24575	38.9	0.0096
6	296.2852	32937	752.3	47620	75.3	0.0090
7	318.2664	32240	820.7	51952	82.1	0.0099
8	367.2697	32495	999.1	63243	100.0	0.0113
9	408.2948	32043	374.1	23679	37.4	0.0127
10	449.3585	31503	538.8	34109	53.9	0.0143

Compound Spectrum List Report

Cmpd 118, Dissect, 14.1 min

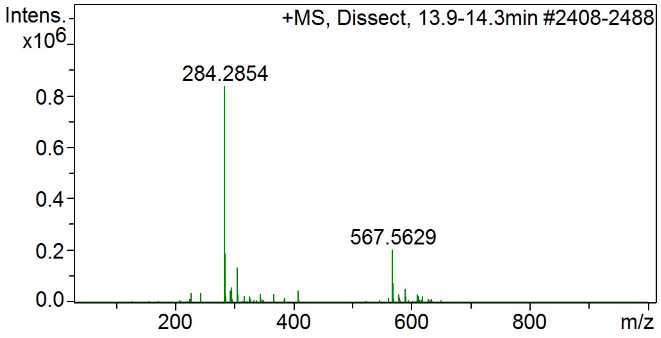

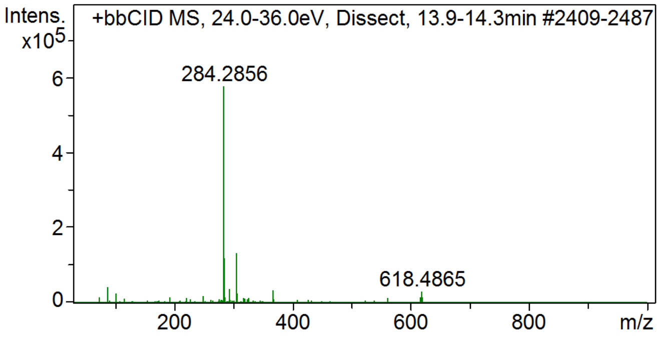

#	m/z	Res.	S/N	I	I %	FWHM
1	244.1827	30169	44.0	36942	4.4	0.0081
2	284.2854	29618	996.5	836956	100.0	0.0096
3	285.2887	36099	228.7	192050	22.9	0.0079
4	294.2330	30312	57.1	47989	5.7	0.0097
5	296.2851	34916	71.3	59873	7.2	0.0085
6	306.2666	33893	161.9	136006	16.3	0.0090
7	408.2946	33463	57.3	48115	5.7	0.0122
8	567.5629	47966	245.9	206573	24.7	0.0118
9	568.5662	41718	92.6	77738	9.3	0.0136
10	589.5442	34966	64.8	54409	6.5	0.0169

#	m/z	Res.	S/N	I	I %	FWHM
1	88.0726	20657	74.2	42888	7.4	0.0043
2	102.0879	21590	45.9	26539	4.6	0.0047
3	250.1697	29463	30.3	17516	3.0	0.0085
4	284.2856	23569	998.0	577016	100.0	0.0121
5	285.2887	36923	208.2	120365	20.9	0.0077
6	294.2332	30904	66.7	38565	6.7	0.0095
7	306.2666	35599	231.3	133726	23.2	0.0086
8	307.2700	31368	44.6	25807	4.5	0.0098
9	367.2697	32000	61.6	35613	6.2	0.0115
10	618.4865	26346	55.3	31964	5.5	0.0235

Cmpd 119, Dissect, 14.2 min

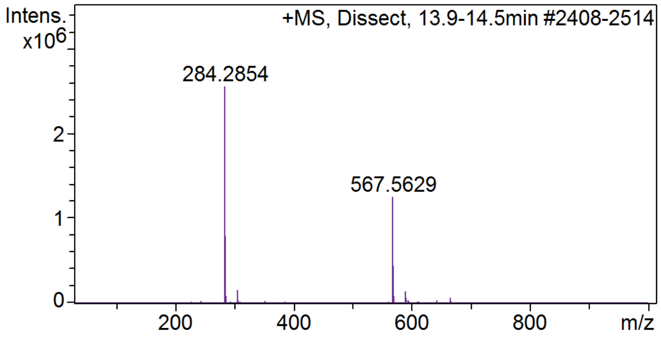

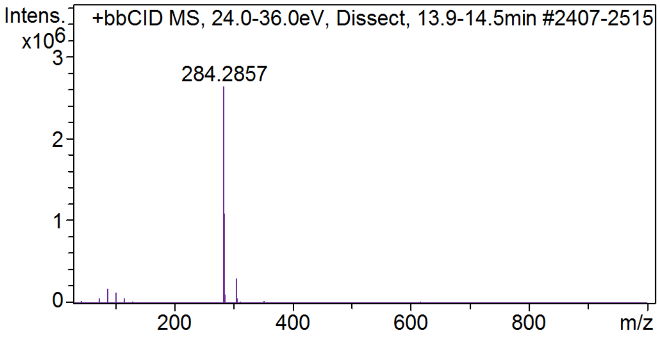

#	m/z	Res.	S/N	I	I %	FWHM
1	284.2854	26692	998.4	2553507	100.0	0.0107
2	285.2886	37223	309.3	790975	31.0	0.0077
3	286.2918	31322	33.7	86303	3.4	0.0091
4	306.2665	34529	63.4	162149	6.4	0.0089
5	567.5629	49766	491.7	1257736	49.3	0.0114
6	568.5663	43233	175.0	447592	17.5	0.0132
7	569.5692	33487	35.2	89953	3.5	0.0170
8	589.5443	36261	58.2	148961	5.8	0.0163
9	590.5474	32975	24.0	61478	2.4	0.0179
10	664.5624	32750	24.1	61601	2.4	0.0203

#	m/z	Res.	S/N	I	I %	FWHM
1	74.0573	19235	21.7	58707	2.2	0.0039
2	88.0725	21007	68.8	185884	7.1	0.0042
3	102.0879	21754	50.7	137180	5.2	0.0047
4	116.1031	22830	21.9	59325	2.3	0.0051
5	284.2857	20517	974.4	2633888	100.0	0.0139
6	285.2690	32125	10.5	28472	1.1	0.0089
7	285.2886	37730	403.8	1091577	41.4	0.0076
8	286.2918	32640	39.0	105454	4.0	0.0088
9	306.2665	36610	113.6	306998	11.7	0.0084
10	307.2699	31854	22.4	60446	2.3	0.0096

Compound Spectrum List Report

Cmpd 120, Dissect, 14.3 min

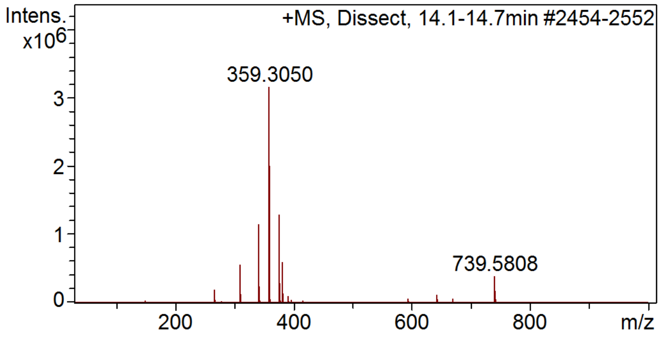

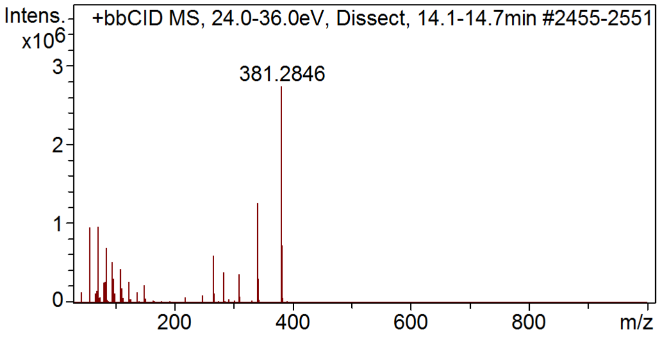

#	m/z	Res.	S/N	I	I %	FWHM
1	267.2592	32824	61.4	197262	6.2	0.0081
2	310.2999	37031	175.4	563668	17.8	0.0084
3	341.2933	40313	359.5	1155480	36.5	0.0085
4	342.2968	36090	77.1	247896	7.8	0.0095
5	359.3050	17242	983.7	3161572	100.0	0.0208
6	360.3067	41584	623.0	2002363	63.3	0.0087
7	376.3292	42298	402.5	1293423	40.9	0.0089
8	377.3325	38207	91.9	295347	9.3	0.0099
9	381.2845	41209	186.8	600256	19.0	0.0093
10	739.5808	41193	124.3	399612	12.6	0.0180

#	m/z	Res.	S/N	I	I %	FWHM
1	57.0676	17814	345.9	953754	34.9	0.0032
2	71.0828	20463	348.5	961180	35.1	0.0035
3	85.0981	21486	253.1	697918	25.5	0.0040
4	95.0822	22580	187.3	516526	18.9	0.0042
5	109.0974	23718	155.6	429218	15.7	0.0046
6	267.2592	37223	218.2	601849	22.0	0.0072
7	284.2852	31016	142.1	391747	14.3	0.0092
8	341.2935	37569	458.9	1265463	46.3	0.0091
9	381.2846	38799	991.9	2735424	100.0	0.0098
10	382.2879	40547	264.3	728836	26.6	0.0094

Cmpd 121, Dissect, 14.3 min

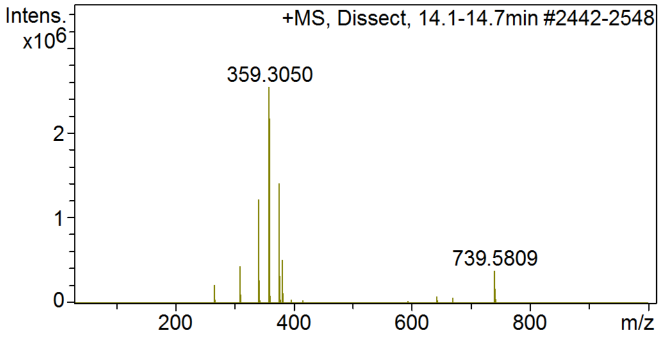

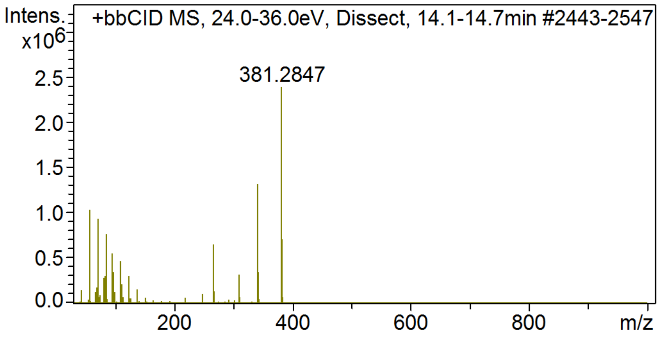

#	m/z	Res.	S/N	I	I %	FWHM
1	267.2592	32805	80.5	222325	8.7	0.0081
2	310.2999	35863	157.8	435920	17.1	0.0087
3	341.2933	40273	440.7	1217836	47.9	0.0085
4	342.2968	36068	100.4	277446	10.9	0.0095
5	359.3050	16632	920.7	2544099	100.0	0.0216
6	360.3067	41599	783.4	2164653	85.1	0.0087
7	376.3292	42236	508.3	1404731	55.2	0.0089
8	377.3326	38132	118.3	326960	12.9	0.0099
9	381.2845	41058	186.2	514635	20.2	0.0093
10	739.5809	41631	139.2	384705	15.1	0.0178

#	m/z	Res.	S/N	I	I %	FWHM
1	57.0676	17822	432.1	1033848	43.2	0.0032
2	71.0828	20496	390.5	934266	39.1	0.0035
3	85.0980	21489	320.6	767029	32.1	0.0040
4	95.0822	22620	232.9	557209	23.3	0.0042
5	97.0978	22210	147.0	351694	14.7	0.0044
6	109.0974	23694	195.5	467644	19.5	0.0046
7	267.2592	37221	273.9	655294	27.4	0.0072
8	341.2935	37650	550.2	1316263	55.0	0.0091
9	381.2847	38221	999.9	2392213	100.0	0.0100
10	382.2879	40558	297.0	710668	29.7	0.0094

Compound Spectrum List Report

Cmpd 122, Dissect, 14.4 min

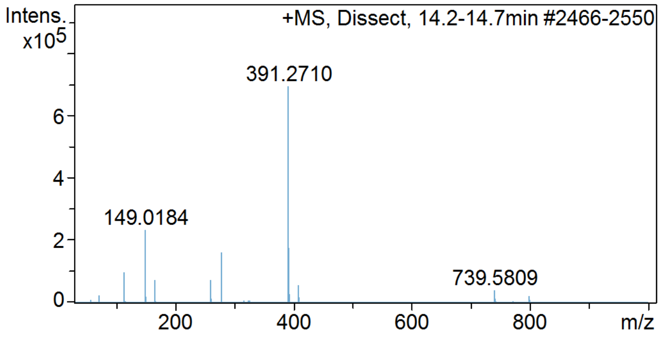

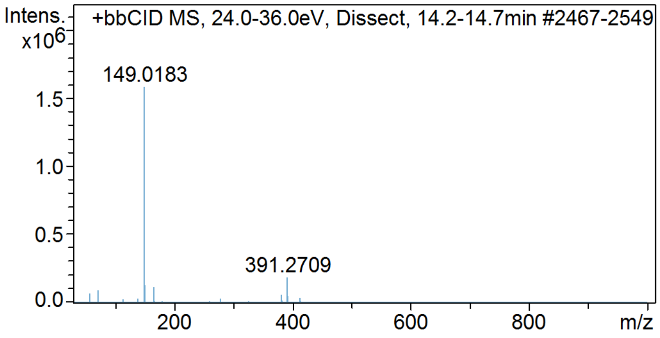

#	m/z	Res.	S/N	I	I %	FWHM
1	113.1287	21555	142.0	99596	14.3	0.0052
2	149.0184	26475	333.7	234076	33.7	0.0056
3	167.0284	27286	105.5	74020	10.7	0.0061
4	261.1398	30227	106.7	74851	10.8	0.0086
5	279.1498	32923	230.5	161693	23.3	0.0085
6	391.2710	40502	989.5	694078	100.0	0.0097
7	392.2743	35001	253.3	177696	25.6	0.0112
8	393.2777	29326	38.3	26895	3.9	0.0134
9	408.2967	32435	83.9	58834	8.5	0.0126
10	739.5809	41264	60.1	42124	6.1	0.0179

#	m/z	Res.	S/N	I	I %	FWHM
1	57.0676	17847	43.7	69434	4.4	0.0032
2	71.0828	20454	59.3	94339	6.0	0.0035
3	149.0183	27977	996.1	1583981	100.0	0.0053
4	150.0217	25972	84.5	134404	8.5	0.0058
5	167.0284	27484	75.8	120579	7.6	0.0061
6	279.1497	31090	20.4	32467	2.0	0.0090
7	381.2847	38550	35.4	56294	3.6	0.0099
8	391.2709	34701	118.5	188382	11.9	0.0113
9	392.2743	31629	32.0	50827	3.2	0.0124
10	413.2521	34988	21.2	33640	2.1	0.0118

Cmpd 123, Dissect, 14.5 min

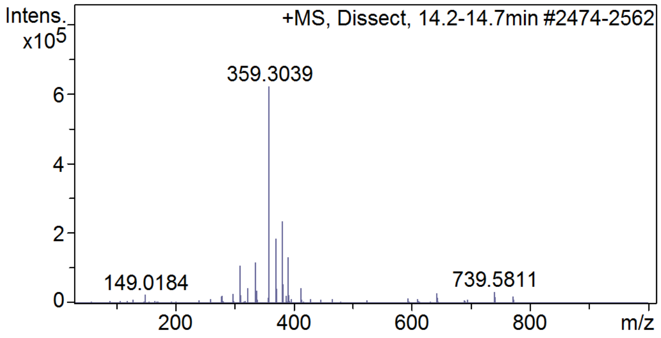

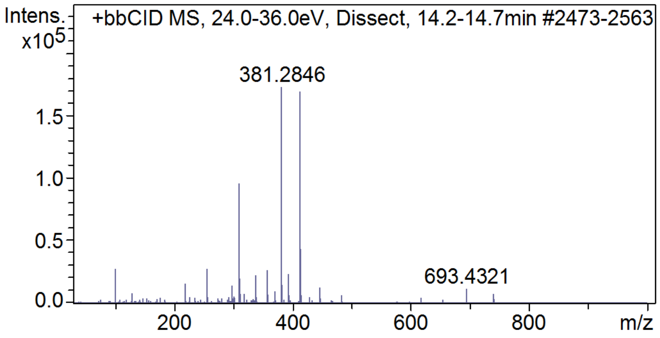

#	m/z	Res.	S/N	I	I %	FWHM
1	310.3001	32153	175.0	109115	17.5	0.0097
2	323.2473	30874	72.7	45317	7.3	0.0105
3	336.3149	32850	188.6	117590	18.9	0.0102
4	359.3039	23487	997.4	621866	100.0	0.0153
5	371.3031	35050	297.9	185708	29.9	0.0106
6	372.3066	30548	68.7	42820	6.9	0.0122
7	381.2847	39908	376.5	234717	37.7	0.0096
8	382.2880	33186	90.5	56403	9.1	0.0115
9	391.2711	39860	213.9	133340	21.4	0.0098
10	413.2521	32282	70.9	44230	7.1	0.0128

#	m/z	Res.	S/N	I	I %	FWHM
1	101.0563	21196	159.8	27606	16.0	0.0048
2	256.2549	29843	160.4	27719	16.0	0.0086
3	310.3000	32596	553.1	95576	55.3	0.0095
4	311.3033	31420	115.5	19954	11.5	0.0099
5	338.3303	30497	132.4	22880	13.2	0.0111
6	358.2961	31416	154.9	26770	15.5	0.0114
7	381.2846	39984	1000.0	172798	100.0	0.0095
8	393.2833	30025	137.0	23673	13.7	0.0131
9	413.2523	35568	978.7	169118	97.9	0.0116
10	414.2555	31829	251.9	43535	25.2	0.0130

Compound Spectrum List Report

Cmpd 124, Dissect, 14.5 min

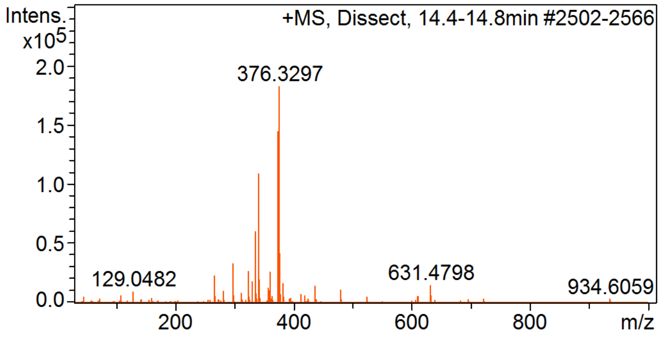

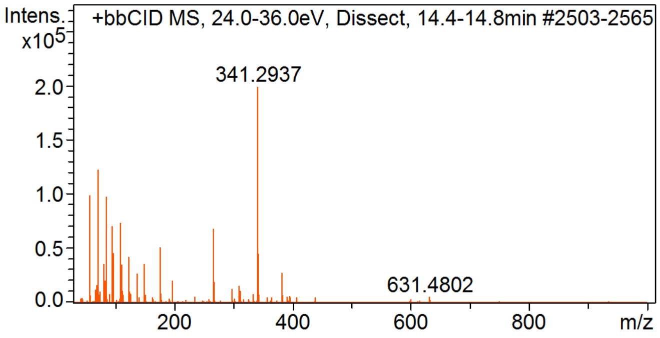

#	m/z	Res.	S/N	I	I %	FWHM
1	267.2595	30193	127.9	23379	12.8	0.0089
2	298.3005	32354	183.0	33445	18.3	0.0092
3	324.3151	31317	149.4	27308	15.0	0.0104
4	336.3149	33146	332.4	60755	33.3	0.0101
5	341.2935	33036	595.6	108866	59.6	0.0103
6	361.2100	34571	146.4	26764	14.7	0.0104
7	374.2351	34717	791.4	144657	79.2	0.0108
8	376.2414	29347	139.0	25408	13.9	0.0128
9	376.3297	32605	999.3	182661	100.0	0.0115
10	377.3328	29427	230.7	42168	23.1	0.0128

#	m/z	Res.	S/N	I	I %	FWHM
1	57.0676	17472	496.0	98973	49.7	0.0033
2	71.0828	19220	614.9	122687	61.6	0.0037
3	85.0981	19721	489.9	97750	49.1	0.0043
4	95.0823	20323	355.2	70862	35.6	0.0047
5	97.0979	20306	230.1	45920	23.0	0.0048
6	109.0975	22172	370.7	73962	37.1	0.0049
7	177.1215	28245	258.3	51545	25.9	0.0063
8	267.2594	30380	343.8	68594	34.4	0.0088
9	341.2937	34968	998.6	199250	100.0	0.0098
10	342.2968	31836	228.9	45675	22.9	0.0108

Cmpd 125, Dissect, 14.7 min

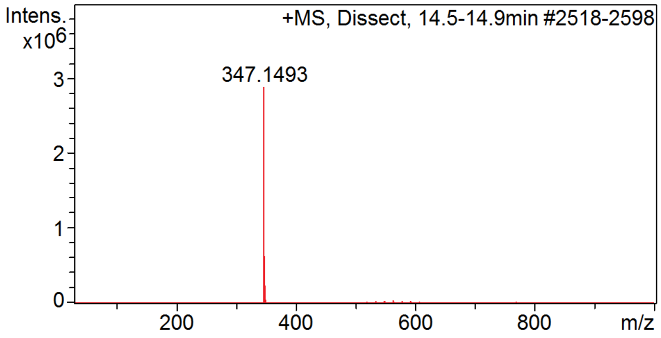

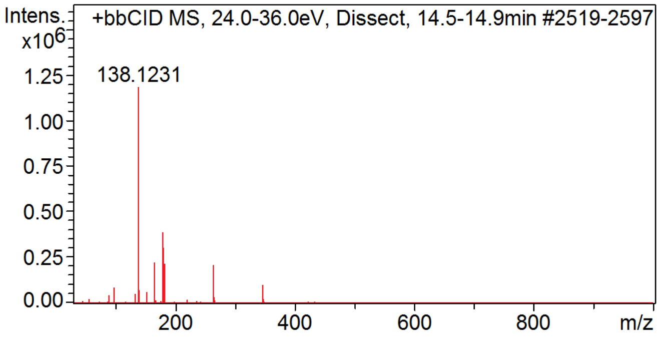

#	m/z	Res.	S/N	I	I %	FWHM
1	347.1493	40925	993.7	2888012	100.0	0.0085
2	348.1523	38167	217.5	632122	21.9	0.0091
3	349.1460	31015	84.4	245427	8.5	0.0113
4	350.1484	28902	17.5	50964	1.8	0.0121
5	533.7014	36327	10.2	29738	1.0	0.0147
6	548.3765	36285	11.6	33645	1.2	0.0151
7	548.7108	36929	10.4	30091	1.0	0.0149
8	563.0512	37791	12.4	36125	1.3	0.0149
9	563.3855	37202	11.3	32749	1.1	0.0151
10	578.0605	37047	10.2	29670	1.0	0.0156

#	m/z	Res.	S/N	I	I %	FWHM
1	98.0931	21013	75.0	88997	7.5	0.0047
2	138.1231	26899	996.7	1182473	100.0	0.0051
3	139.1265	23715	62.0	73549	6.2	0.0059
4	152.1383	25788	55.1	65310	5.5	0.0059
5	165.9724	28975	189.7	224999	19.0	0.0057
6	180.1686	30256	330.0	391461	33.1	0.0060
7	181.1759	28933	258.8	307081	26.0	0.0063
8	182.9983	29678	183.3	217417	18.4	0.0062
9	265.0739	34052	177.2	210182	17.8	0.0078
10	347.1495	33998	85.5	101489	8.6	0.0102

Compound Spectrum List Report

Cmpd 126, Dissect, 14.8 min

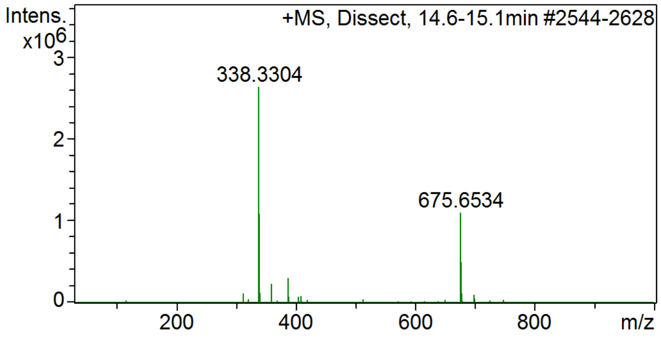

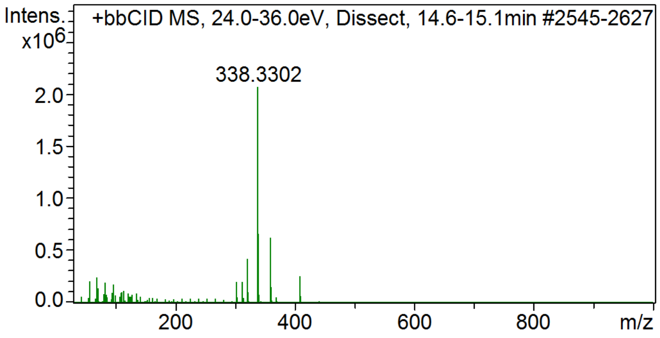

#	m/z	Res.	S/N	I	I %	FWHM
1	312.3158	34045	45.1	119751	4.5	0.0092
2	338.3304	27850	992.4	2637028	100.0	0.0121
3	339.3337	40200	408.0	1084205	41.1	0.0084
4	340.3370	33012	48.3	128290	4.9	0.0103
5	360.3114	36322	89.5	237914	9.0	0.0099
6	387.3337	38833	116.5	309453	11.7	0.0100
7	675.6534	49657	415.1	1103050	41.8	0.0136
8	676.6567	43981	190.5	506230	19.2	0.0154
9	677.6597	34276	46.1	122626	4.7	0.0198
10	697.6345	33458	36.2	96302	3.7	0.0209

#	m/z	Res.	S/N	I	I %	FWHM
1	57.0676	17708	102.9	213514	10.3	0.0032
2	69.0672	19154	119.4	247820	12.0	0.0036
3	83.0824	20784	95.7	198708	9.6	0.0040
4	303.2944	34633	99.6	206688	10.0	0.0088
5	312.3156	35567	99.3	206022	9.9	0.0088
6	321.3043	37205	203.7	422765	20.4	0.0086
7	338.3302	33142	997.9	2071313	100.0	0.0102
8	339.3335	39304	320.1	664426	32.1	0.0086
9	360.3113	39618	301.9	626606	30.3	0.0091
10	409.3148	38859	125.4	260240	12.6	0.0105

Cmpd 127, Dissect, 15.0 min

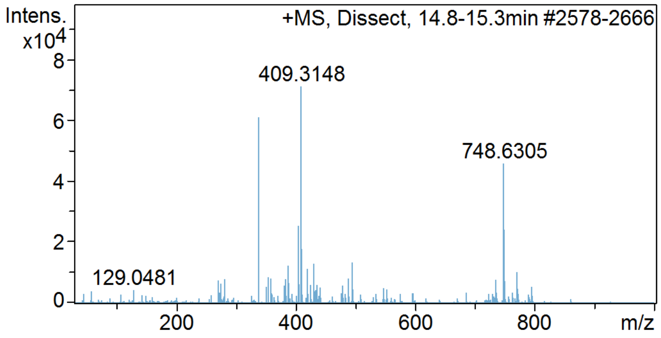

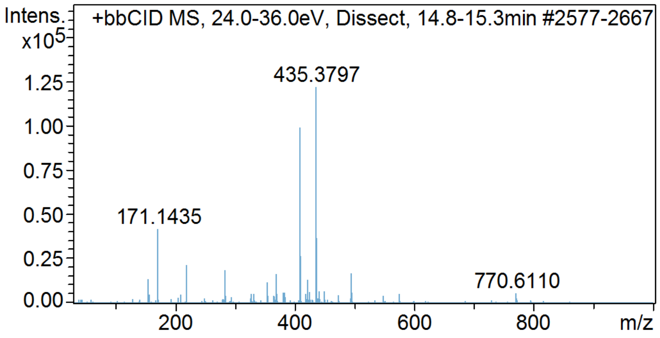

#	m/z	Res.	S/N	I	I %	FWHM
1	338.3302	35563	856.4	60935	85.7	0.0095
2	387.3337	37354	175.6	12494	17.6	0.0104
3	404.3597	33280	358.9	25537	35.9	0.0122
4	409.3148	34620	999.9	71142	100.0	0.0118
5	410.3182	30181	250.8	17842	25.1	0.0136
6	419.3012	32226	161.7	11505	16.2	0.0130
7	430.2957	31727	184.7	13142	18.5	0.0136
8	494.5485	31735	190.0	13516	19.0	0.0156
9	748.6305	29481	645.2	45906	64.5	0.0254
10	749.6336	29136	339.3	24139	33.9	0.0257

#	m/z	Res.	S/N	I	I %	FWHM
1	154.1175	26044	113.9	13911	11.4	0.0059
2	171.1435	28128	344.6	42081	34.5	0.0061
3	220.1235	29099	179.6	21928	18.0	0.0076
4	284.2853	30225	156.4	19094	15.6	0.0094
5	369.3238	33000	136.6	16680	13.7	0.0112
6	409.3150	38055	811.2	99054	81.1	0.0108
7	410.3184	32398	220.8	26963	22.1	0.0127
8	435.3797	35645	999.9	122096	100.0	0.0122
9	436.3828	32291	303.6	37077	30.4	0.0135
10	494.5487	31544	140.0	17100	14.0	0.0157

Compound Spectrum List Report

Cmpd 128, Dissect, 15.1 min

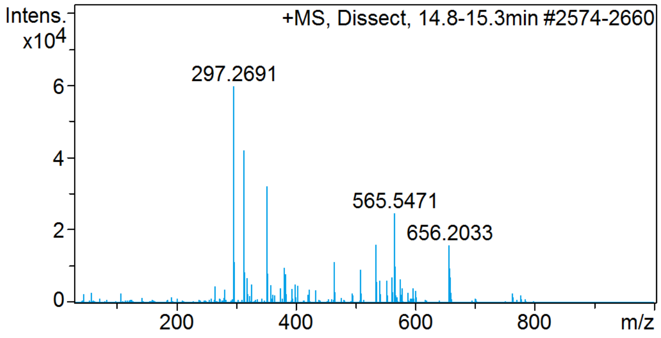

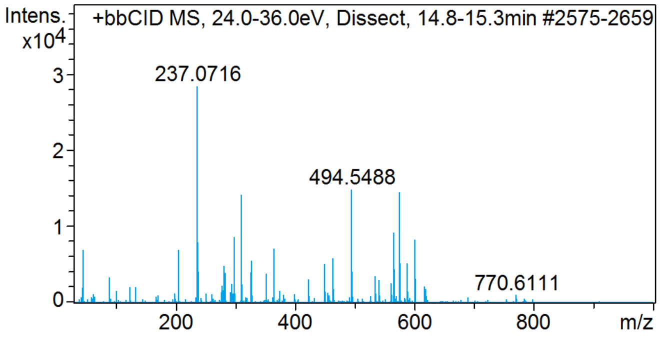

#	m/z	Res.	S/N	I	I %	FWHM
1	297.2691	33085	999.7	59677	100.0	0.0090
2	298.2721	28282	188.8	11273	18.9	0.0105
3	314.2950	32339	703.1	41971	70.3	0.0097
4	352.3456	31289	538.0	32116	53.8	0.0113
5	381.2847	31449	164.6	9828	16.5	0.0121
6	464.4149	30650	189.8	11333	19.0	0.0152
7	534.4695	31500	269.1	16067	26.9	0.0170
8	565.5471	31520	415.1	24781	41.5	0.0179
9	566.5505	30785	170.3	10164	17.0	0.0184
10	656.2033	31082	267.9	15994	26.8	0.0211

#	m/z	Res.	S/N	I	I %	FWHM
1	45.0316	15804	246.2	6990	24.6	0.0028
2	237.0716	30442	999.5	28375	100.0	0.0078
3	238.0725	25766	281.3	7987	28.1	0.0092
4	299.2957	30762	304.1	8633	30.4	0.0097
5	310.3002	30798	501.1	14225	50.1	0.0101
6	365.0932	31936	251.9	7151	25.2	0.0114
7	494.5488	32071	523.6	14865	52.4	0.0154
8	565.5472	31074	325.6	9243	32.6	0.0182
9	574.4612	30792	511.3	14515	51.2	0.0187
10	600.4758	30117	293.3	8328	29.3	0.0199

Cmpd 129, Dissect, 15.2 min

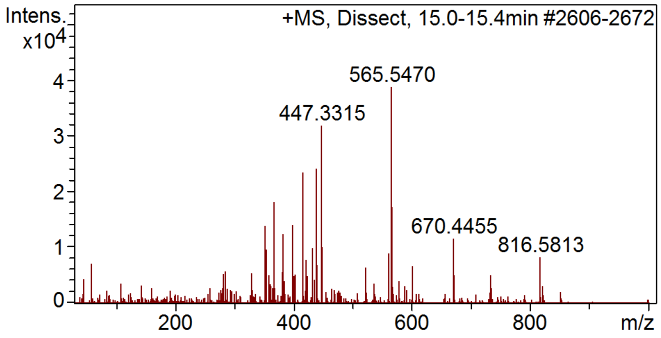

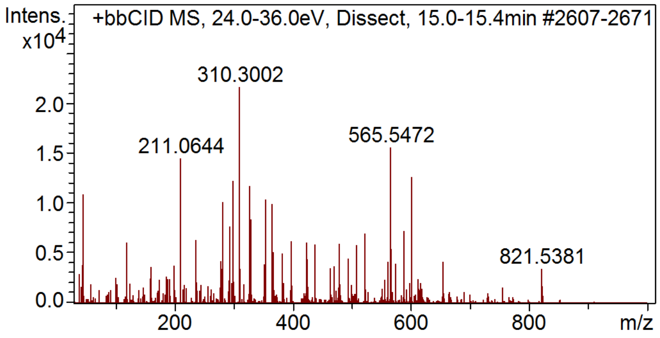

#	m/z	Res.	S/N	I	I %	FWHM
1	352.3458	31912	358.5	13924	35.9	0.0110
2	367.3237	32354	382.8	14867	38.3	0.0114
3	367.3641	32147	469.0	18217	46.9	0.0114
4	383.1913	33657	321.5	12487	32.2	0.0114
5	399.3337	28985	361.7	14050	36.2	0.0138
6	416.3700	30825	602.9	23419	60.3	0.0135
7	439.3410	30442	619.9	24075	62.0	0.0144
8	447.3315	32936	817.4	31748	81.7	0.0136
9	565.5470	32459	1000.0	38839	100.0	0.0174
10	566.5502	31349	443.6	17231	44.4	0.0181

#	m/z	Res.	S/N	I	I %	FWHM
1	45.0316	15754	502.7	10888	50.3	0.0029
2	211.0644	28355	669.8	14507	67.0	0.0074
3	282.2698	31204	467.5	10126	46.8	0.0090
4	299.2958	30578	566.6	12273	56.7	0.0098
5	310.3002	30574	1000.0	21660	100.0	0.0101
6	327.3263	30795	540.6	11710	54.1	0.0106
7	354.3611	30939	478.4	10363	47.8	0.0115
8	365.0933	31730	458.6	9934	45.9	0.0115
9	565.5472	32286	719.4	15582	71.9	0.0175
10	600.4759	29368	582.0	12606	58.2	0.0204

Compound Spectrum List Report
